# Diastereoselective Synthesis of *cis*-α,α′-Disubstituted Cyclic Ethers via Borane-Catalyzed Reductive Cyclization of Diketones

**DOI:** 10.1021/acscatal.5c08127

**Published:** 2026-01-02

**Authors:** Nikolay V. Shcherbakov, Nathaniel Potin, Josep Mas-Roselló

**Affiliations:** Laboratorium für Organische Chemie, ETH Zürich, D-CHAB, Zürich 8093, Switzerland

**Keywords:** boranes, frustrated Lewis pairs, homogeneous catalysis, hydrogenation, oxygen heterocycles

## Abstract

Cyclic ethers, particularly *cis*-α,α′-disubstituted tetrahydrofurans (THFs) and tetrahydropyrans (THPs), are prevalent motifs in bioactive compounds. However, efficient stereoselective methods for accessing such motifs remain limited. Herein, we report a direct catalytic approach to *cis*-2,5- and *cis*-2,6-disubstituted THFs and THPs via reductive cycloetherification of 1,4- and 1,5-diketones. The transformation uses a simple triarylborane catalyst and dihydrogen as a clean reductant, producing water as the sole byproduct. A broad range of products were obtained in high yields (up to 97%) and with high *cis*-selectivity (up to >20:1 dr). The method enabled streamlined access to medicinally relevant scaffolds and was scalable to gram quantities. Mechanistic studies support oxocarbenium ion reduction as the stereodetermining step, while density functional theory calculations suggest that weak, noncovalent interactions between the catalyst and substrate contribute to the observed diastereoselectivity. This work introduces a borane-catalyzed hydrogenative cyclization strategy that may inspire future metal-free syntheses of stereodefined heterocycles.

## Graphical abstract

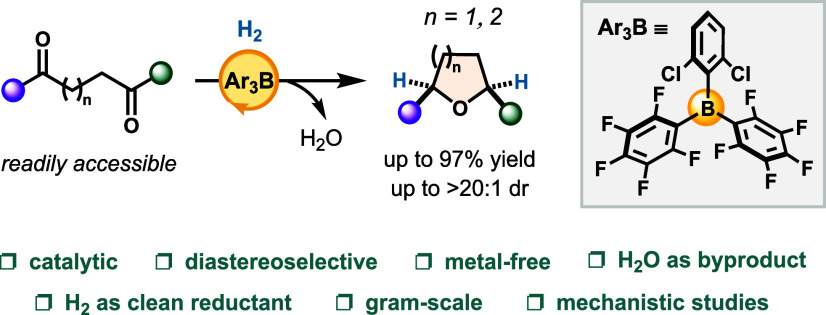

## Introduction

Cyclic ethers such as tetrahydrofurans (THFs) and tetrahydropyrans (THPs) are ubiquitous structural motifs found in diverse natural products (e.g., lignans and macrolides) and pharmaceuticals.^1^ Notably, THFs and THPs rank among the top 10 single-ring systems present in FDA-approved drugs.^2^ An important subclass comprises *cis*-configured α,α′-disubstituted THFs and THPs, which are common in numerous bioactive molecules (Scheme 1A).^3–7^ Despite their prevalence, the stereoselective synthesis of these motifs—especially *cis*-2,5-THFs—remains a significant challenge. Traditional approaches typically involve intramolecular S_N_2 reactions between a hydroxyl and a leaving group (e.g., halide or epoxide),^1,8,9^ requiring multistep sequences to install these functionalities and predefine stereochemistry. Alternative stereoselective methods often rely on cyclization of prefunctionalized olefin substrates, but these are mainly applicable to six-membered THPs.^10–17^ In contrast, stereoselective approaches to *cis*-2,5-THFs are less developed, with reported methods including hydrogenation of furans using transition-metal catalysts^18–22^ and cycloaddition of activated cyclopropane substrates.^23–25^

**Scheme 1:**
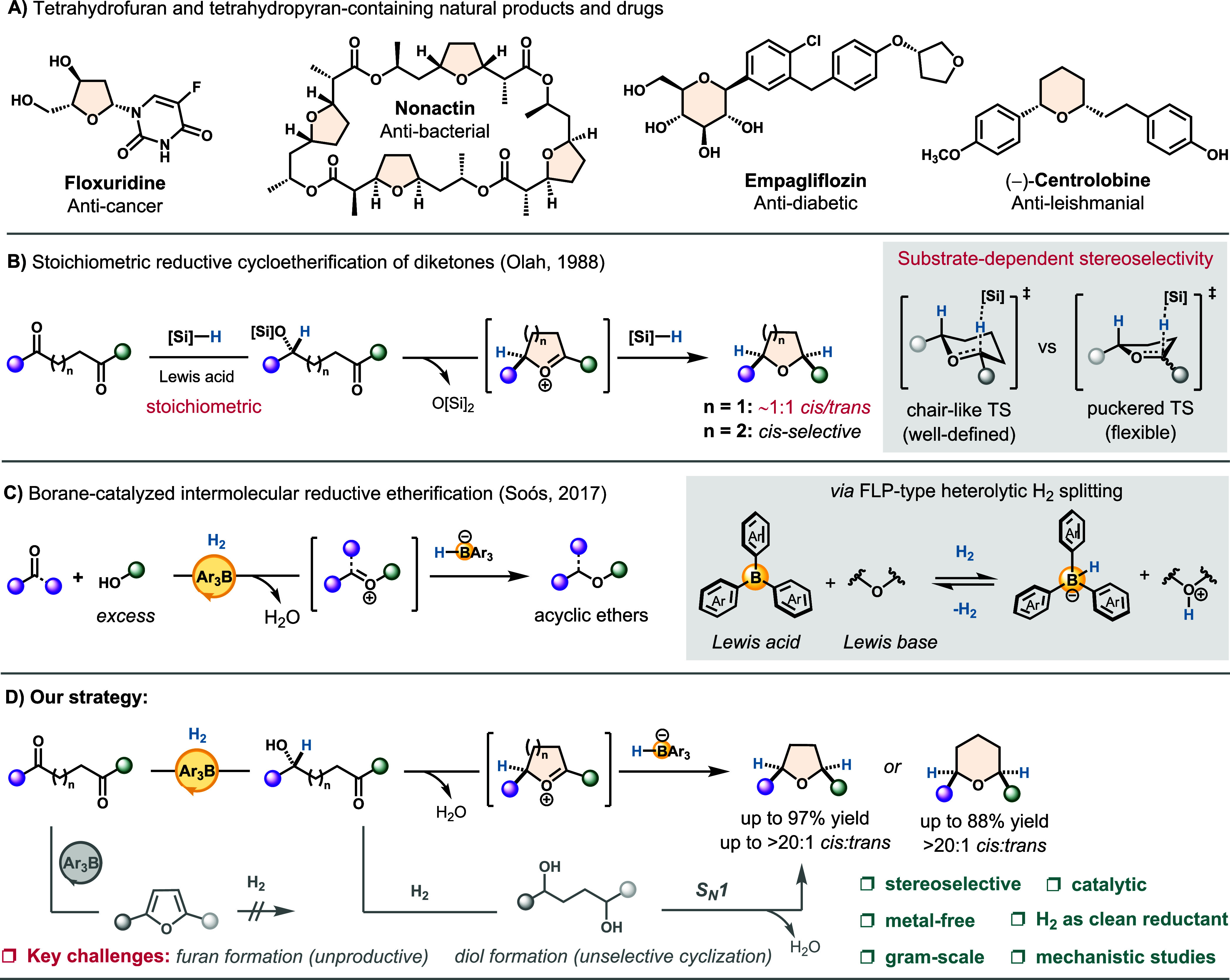
(A–D) Biological Importance of Tetrahydrofuran and Tetrahydropyran Rings and Relevant Synthetic Strategies Involving Reductive Etherification

In this context, we sought to develop a metal-free, broadly applicable catalytic strategy for the efficient and stereoselective synthesis of both *cis*-disubstituted THF and THP rings. We were inspired by the reductive cycloetherification of diketones, a straightforward approach to cyclic ethers first reported by Olah and co-workers in 1988 (Scheme 1B).^26^ This transformation employs a stoichiometric Lewis acid (e.g., TMSOTf or BF_3_·Et_2_O) and a silane to promote a tandem sequence involving ketone reduction, cyclization to an oxocarbenium ion, and subsequent hydride delivery. However, while THPs typically form with high *cis*-selectivity, THFs are often obtained as ∼1:1 *cis*/*trans* mixtures.^26–28^ This disparity arises from conformational differences in the stereodetermining oxocarbenium ion reduction step: THP rings can adopt rigid, chairlike transition states, whereas the greater flexibility of THFs complicates stereocontrol.^26^ Only in specific cases, substitution at C3 or C4 has been shown to favor *cis*-2,5 selectivity.^29–31^ Nevertheless, achieving stereocontrol in purely 2,5-disubstituted THFs remains challenging. Recently, Niu et al. achieved selective oxocarbenium ion reduction to *cis*-2,5-THFs using a bulky Hantzsch ester as the reductant.^32^ Despite this remarkable advance, the reliance on stoichiometric reagents, including silver salts, results in substantial formation of byproducts, which may limit scalability.

We envisioned a catalytic cycloetherification strategy using dihydrogen (H_2_) gas as a clean, cost-effective reductant. A suitable catalyst would need to fulfill three key criteria: (i) activate H_2_, (ii) exhibit strong Lewis acidity to promote oxocarbenium ion formation, and (iii) possess sufficient steric bulk to enforce facial selectivity during hydride delivery to the oxocarbenium ion. With these requirements in mind, we turned our attention to triarylborane Lewis acids.^33–35^ Seminal work by Stephan and co-workers demonstrated that highly Lewis acidic triarylboranes, in combination with ether solvents, can heterolytically split H_2_ via a frustrated Lewis pair (FLP) mechanism, generating a bulky borohydride species [Ar_3_BH]^−^ and a strongly Brønsted acidic oxonium ion.^36^ This unique reactivity^37–39^ has enabled metal-free catalytic hydrogenations of carbonyls.^40–44^ Furthermore, Soós and co-workers showed that triarylboranes can catalyze reductive etherifications of carbonyl compounds with alcohols in excess via borohydride-mediated reduction of oxocarbenium ion intermediates, affording acyclic ethers (Scheme 1C).^45^

Building on these precedents, herein, we report a borane-catalyzed reductive cycloetherification of diketones that directly affords *cis*-2,5- and *cis*-2,6-disubstituted THFs and THPs in high yields and with high diastereoselectivity from readily available diketone precursors (Scheme 1D). This transformation integrates carbonyl reduction, cyclodehydration, and oxocarbenium ion reduction into a single catalytic operation, generating water as the sole byproduct. Compared to related acyclic reductive etherifications,^45^ our system entails greater mechanistic complexity, featuring an additional reduction step, ring formation, and stereoselective installation of multiple stereocenters. It also presents unique challenges, such as the need to suppress competing pathways like Paal–Knorr-type cyclization to furans^46^ and to avoid premature reduction of both carbonyl groups, which can lead to unselective S_N_1-type cyclization (*vide infra*).

## Methods

### General Procedure for Borane-Catalyzed Reductive Cyclization of Diketones

A flame-dried 4 mL glass vial (screw cap with PTFE/silicone septum) equipped with a magnetic stirring bar was charged with 4 Å molecular sieves (∼150 mg/mmol substrate), borane catalyst (0.02 mmol, 10 mol %), diketone substrate (0.2 mmol), and CPME (0.6 mL) under an atmosphere of argon. The mixture was stirred for 2 min, and then, the vial was pierced with a needle and placed in an autoclave. The system was sealed and purged three times with nitrogen (6 bar) and four times with hydrogen (3 bar) and then pressurized to 100 bar with hydrogen gas. The reaction mixture was heated to 80 °C and stirred for 18 h. After the reaction completed, the autoclave was allowed to cool to room temperature and slowly depressurized. The reaction mixture was filtered through a short silica plug and eluted with ethyl acetate. The filtrate was concentrated under reduced pressure, and the crude product was purified by flash column chromatography on silica gel to give the desired cyclic ether product.

## Results and Discussion

### Reaction Development

We initiated our studies using commercially available 1,4-diketone **1a** as a model substrate to investigate its reductive cyclization to the THF product **2a** (Table 1, see the Supporting Information for full details). Using tris­(pentafluorophenyl)­borane (**B1**)—the prototypical FLP Lewis acid component^47^—in *i*Pr_2_O as the Lewis basic solvent,^40^ **2a** was obtained in 66% yield with a 6:1 *cis:trans* diastereomeric ratio (entry 1). The *cis*-configuration was confirmed by the NOE analysis. Despite this promising result, a significant 31% of undesired furan side product **3a** also formed, likely due to nonreductive Paal–Knorr-type cyclization catalyzed by the strongly Lewis acidic borane.^46^ We thus hypothesized that reducing the Lewis acidity of the catalyst could help suppress this side pathway.^41,48^ To assess Lewis acidity, we calculated the fluoride ion affinity (FIA) of the boranes—a common measure to quantify Lewis acidity^49^—obtaining a value of 467 kJ/mol for **B1**. Notably, Soós’ catalyst **B2**, which is ∼12% less Lewis acidic (FIA = 409 kJ/mol), suppressed furan formation and improved diastereoselectivity (11:1 dr), likely due to its increased steric bulk from *ortho*-chlorine substituents.^42^ However, this came at the expense of activity, affording only 30% of the product (entry 2).

**Table 1:**
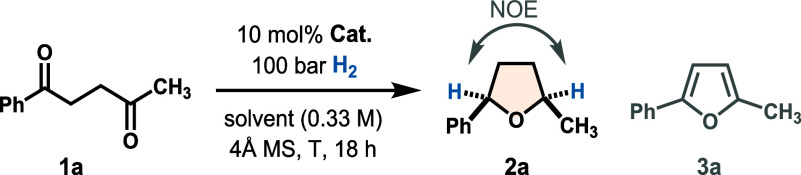

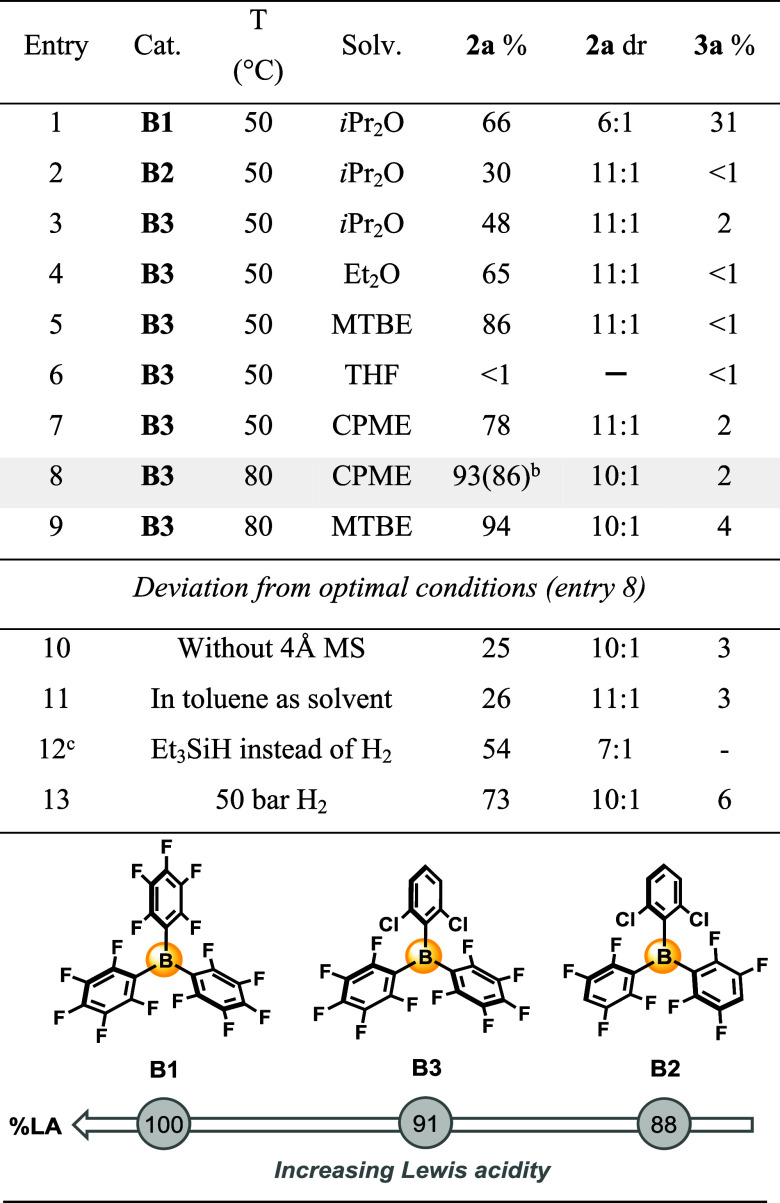
Reaction Design and Optimization^a^

a Reaction conditions (unless otherwise stated): **1a** (0.1 mmol), 10 mol % catalyst, 4Å MS (150 mg), 0.3 mL of the solvent, 100 bar H_2_, 18 h. Yields and diastereomeric ratios (dr) were determined by ^1^H NMR spectroscopy.

b Isolated yield.

c 2 equiv of Et_3_SiH in CH_2_Cl_2_ at rt, 18 h. %LA = relative Lewis acidity normalized to the fluoride ion affinity of **B1** (set as 100%).

The best overall performance was achieved with catalyst **B3**,^50^ which has intermediate Lewis acidity (FIA = 423 kJ/mol). It delivered **2a** in 48% yield with 11:1 dr and only a trace formation of furan (entry 3). These results corroborate our initial hypothesis that balancing the borane’s Lewis acidity is key to catalytic performance, with **B3** providing the best compromise between reactivity and product selectivity. Using **B3**, we next evaluated a range of ether solvents (Table 1, entries 3–6). We found no clear correlation between solvent Lewis basicity^51^ and reaction outcome. Both more coordinating diethyl ether (entry 4) and less coordinating methyl *tert*-butyl ether (entry 5) outperformed *i*Pr_2_O. In contrast, THF completely suppressed the product formation (entry 6). Notably, cyclopentyl methyl ether (CPME)—an industrially attractive solvent^52^—further improved the reaction, affording 78% of **2a** (entry 7). Finally, raising the temperature to 80 °C furnished **2a** in 93% yield (86% isolated) with a 10:1 dr (entry 8). While MTBE gave a comparable outcome (entry 9), we occasionally observed significant solvent loss due to its higher volatility, which resulted in poor mass balance. To avoid this issue, we continued the studies using CPME as the solvent.

Control experiments highlighted additional key parameters. Omission of molecular sieves led to a significantly reduced yield of 25% (entry 10), underlining the importance of removing water to prevent catalyst deactivation. Conducting the reaction in toluene, a nonbasic solvent, still afforded the product, albeit in a lower 26% yield (entry 11). This suggests that Lewis basic components other than the solvent, such as sieves,^44^ the substrate, or the product, may also form effective FLPs. However, their activity appears to be low as an ethereal solvent is required for high yield. While product-induced autocatalysis cannot be ruled out entirely, it seems to be minor under the optimized conditions. Replacing H_2_ with triethylsilane as the reductant lowered both yield and diastereoselectivity (entry 12). The low yield was due to the formation of a linear deoxygenated side product (see the SI).^53,54^ This result demonstrates the advantages of using H_2_: not only is it cleaner and more cost-effective than silanes, but H_2_ also provides superior selectivity in this system. On a practical note, the H_2_ pressure can be lowered if necessary; for example, at 50 bar, only a slight decrease in product yield was observed (73%), while the diastereoselectivity remained unchanged (entry 13).

### Reaction Scope and Streamlined Syntheses

With the optimized conditions in hand, we next evaluated the generality of the method. A broad range of diketones **1** was converted to the corresponding α,α′-disubstituted cyclic ethers **2** in generally high yields and with high *cis*-selectivity (Scheme 2). We first examined 1-arylpentane-1,4-diones, which provided arylmethyl-substituted THFs **2a**–**l**. A gram-scale synthesis of **2a** proceeded smoothly, demonstrating good scalability. A variety of substituents on the aryl ring were tolerated, including chloro (**2b**), methyl ester (**2c**), and boronic ester (**2f**) groups, which may serve as handles for further derivatization. Notably, polar Lewis basic groups such as nitrile (**2g**), secondary amide (**2h**), and primary alcohol (**2i**), which could coordinate to and potentially deactivate the Lewis acid catalyst, were compatible, albeit requiring higher catalyst loadings for better yield. Beyond phenyl groups, naphthyl (**2j**) and heteroarenes such as thiophene (**2k**) and *N*-tosyl indole (**2l**) were also tolerated. In general, electron-rich arenes gave a slightly lower diastereoselectivity (**2e** and **2l**).

**Scheme 2:**
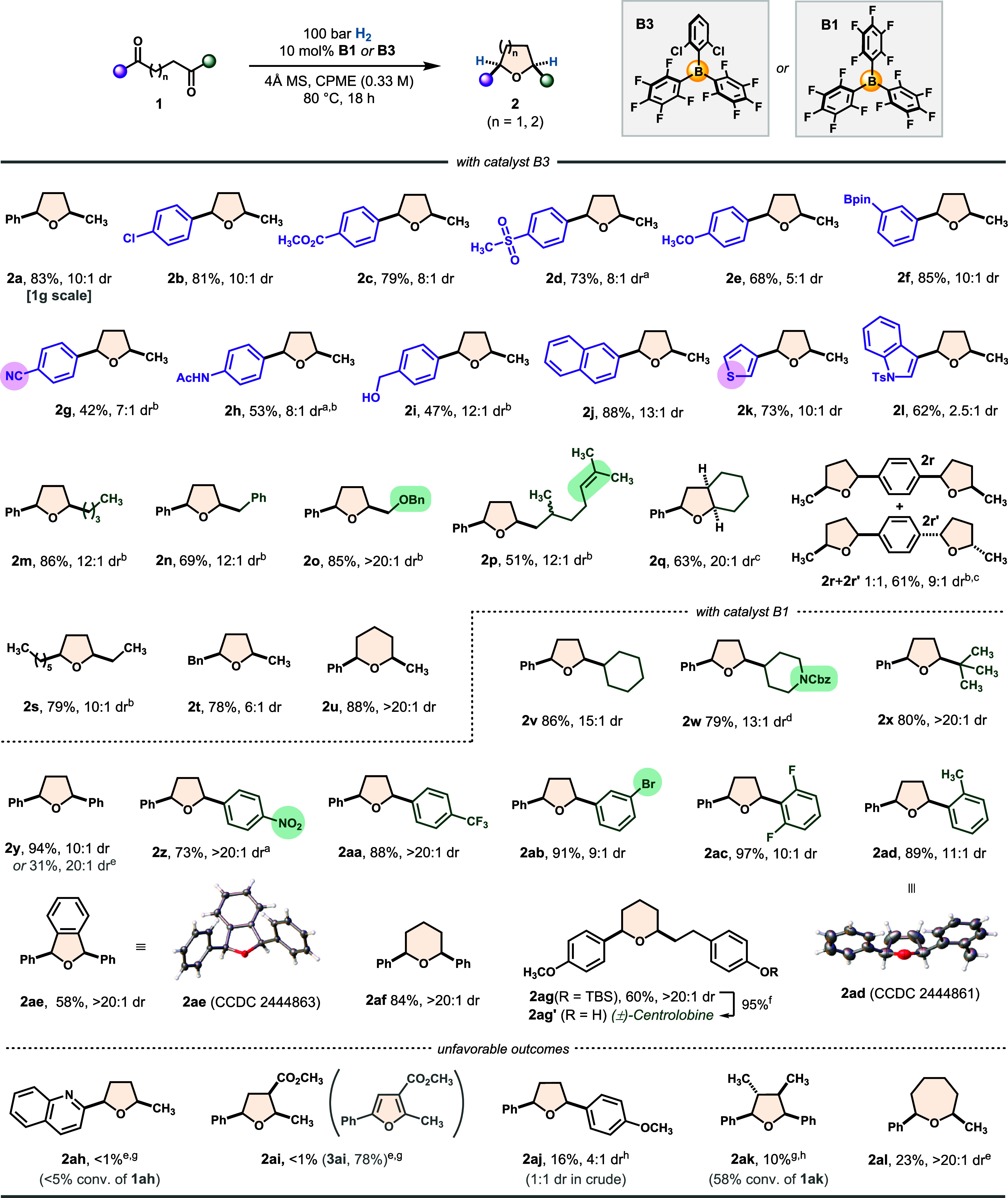
Reaction Scope

Reaction conditions: **1** (0.2 mmol), 10 mol % **B3** or **B1**, 4 Å MS (300 mg), 0.6 mL of CPME, 100 bar H_2_, 18 h. Isolated yields are given. Diastereomeric ratios (dr) were determined by ^1^H NMR spectroscopy.
a 3:1 CPME:1,2-DCE as a solvent.
b 20 mol % **B3** used as a catalyst.
c All minor diastereomers are combined in a single number.
d 20 mol % **B1** used as a catalyst.
e 10 mol % **B3** used as a catalyst.
f Conditions: 1.3 equiv of TBAF in THF, rt, 2 h.
g Determined by crude ^1^H NMR using 1,3,5-trimethoxybenzene as an internal standard.
h 10 mol % **B1** used as a catalyst. In X-ray structures, disorder was removed for clarity.

Different alkyl substituents were well-tolerated, with bulkier groups generally affording higher diastereoselectivity but a somewhat reduced reactivity. For primary alkyl substrates (**2m**–**p**), 20 mol % **B3** was used to ensure high yields. A bicyclic octahydrobenzofuran scaffold (**2q**) bearing three stereocenters was obtained with excellent all-*cis* selectivity. In addition, a double reductive cyclization furnished bis-THFs **2r** and **2r**′ in good combined yield with high *cis*-selectivity. This transformation forges two rings and four stereocenters in a single operation, illustrating the method’s capacity for rapid buildup of molecular complexity. Beyond aryl systems, the method also provided access to dialkyl-substituted THFs such as **2s**, a five-membered analog of (±)-*cis*-lauthisan,^55^ and **2t**, both obtained in high yield and selectivity despite the nonstabilized nature of their corresponding oxocarbenium intermediates. Substrates bearing secondary (Cy in **2v**, *N*-Cbz-piperidine in **2w**) and even tertiary (*t*Bu in **2x**) alkyl groups were also well-tolerated. For these sterically demanding substrates, the less bulky catalyst **B1** provided higher yields. A similar trend was observed for diaryl substrates: diphenyl product **2y** formed in 31% yield and 20:1 dr with **B3**, versus 94% yield and 10:1 dr with **B1**. Using catalyst **B1**, arenes bearing *para*- (**2z** and **2aa**), *meta*- (**2ab**), and *ortho*-substituents (**2ac** and **2ad**) also reacted with high yields and diastereoselectivity. The structure of **2ad** was unambiguously confirmed by X-ray crystal structure analysis, validating the *cis*-stereochemistry. Additionally, a 1,3-dihydroisobenzofuran fused-ring system (**2ae**) was also obtained as a single *cis*-isomer, confirmed by X-ray crystal structure analysis.

While our scope focused primarily on THF rings since five-membered rings are more challenging to access with high *cis*-selectivity than six-membered THPs (*vide supra*), a few THP products (**2u**, **2af**, and **2ag**) were also successfully prepared as single *cis*-diastereomers. Desilylation of **2ag** afforded (±)-centrolobine.^4^ In addition to being applicable to both THFs and THPs, another notable feature of the method is its compatibility with several functionalities often problematic under classical metal-catalyzed hydrogenation: olefin (**2p**), nitro (**2z**), cyano (**2g**), and aryl bromide (**2ab**) groups, which are typically reduced, remained intact under our conditions. Likewise, groups prone to cleavage such as benzyl ether (**2o**) or Cbz carbamate (**2w**) were preserved, highlighting the high chemoselectivity of the method toward carbonyl reduction. Furthermore, whereas sulfur-containing heteroarenes often poison metal catalysts, thiophene (**2k**) was well-tolerated by our system. Nevertheless, a few substrates proved to be challenging. Quinoline-containing **1ah** did not react, likely due to its nitrogen basicity quenching FLP reactivity. Ester-substituted **1ai** yielded mostly furan **3ai** (78% by NMR), presumably due to resonance-stabilized enolization favoring nonreductive Paal–Knorr cyclization.^56^ Electron-rich diaryl substrate **1aj** gave a low product yield (16%) and poor diastereoselectivity (4:1 dr, 1:1 dr by crude NMR). Tetrasubstituted diketone **1ak** gave only 10% of product **2ak** with 42% of the starting material remaining, likely due to steric hindrance hampering reactivity. Lastly, seven-membered oxepane **2al** was obtained with excellent *cis*-selectivity but in low yield (23%), likely due to the difficult cyclization, as several linear side products were observed in the crude.

To further evaluate the synthetic utility of our method, we applied it to the synthesis of two pharmaceutically relevant THF derivatives (Scheme 3). The first, **2am**′, a known intermediate in the synthesis of an antimalarial compound,^57^ was obtained as a single *cis*-isomer *via* a concise three-step sequence: a Stetter reaction to generate the 1,4-diketone precursor (**1am**), catalytic reductive cycloetherification, and final *N*-phthalimide deprotection. Compared to the previously reported five-step route, our approach is both shorter and delivers the target compound in higher overall yield (68% vs 45%), while featuring two catalytic transformations that enhance atom-economy. Using a similar strategy, we synthesized compound **2an**′, which has been reported to exhibit antidepressant activity.^58^ Our five-step route provides the target compound in 31% overall yield and a 5:1 dr, representing an improvement over the previously reported seven-step synthesis, which required chromatographic separation of *cis*/*trans*-isomers and afforded the product in a lower 5% overall yield. Thus, our approach not only reduces the step count but also improves sustainability by incorporating stereoselective catalysis and avoiding isomer separation steps.

**Scheme 3:**
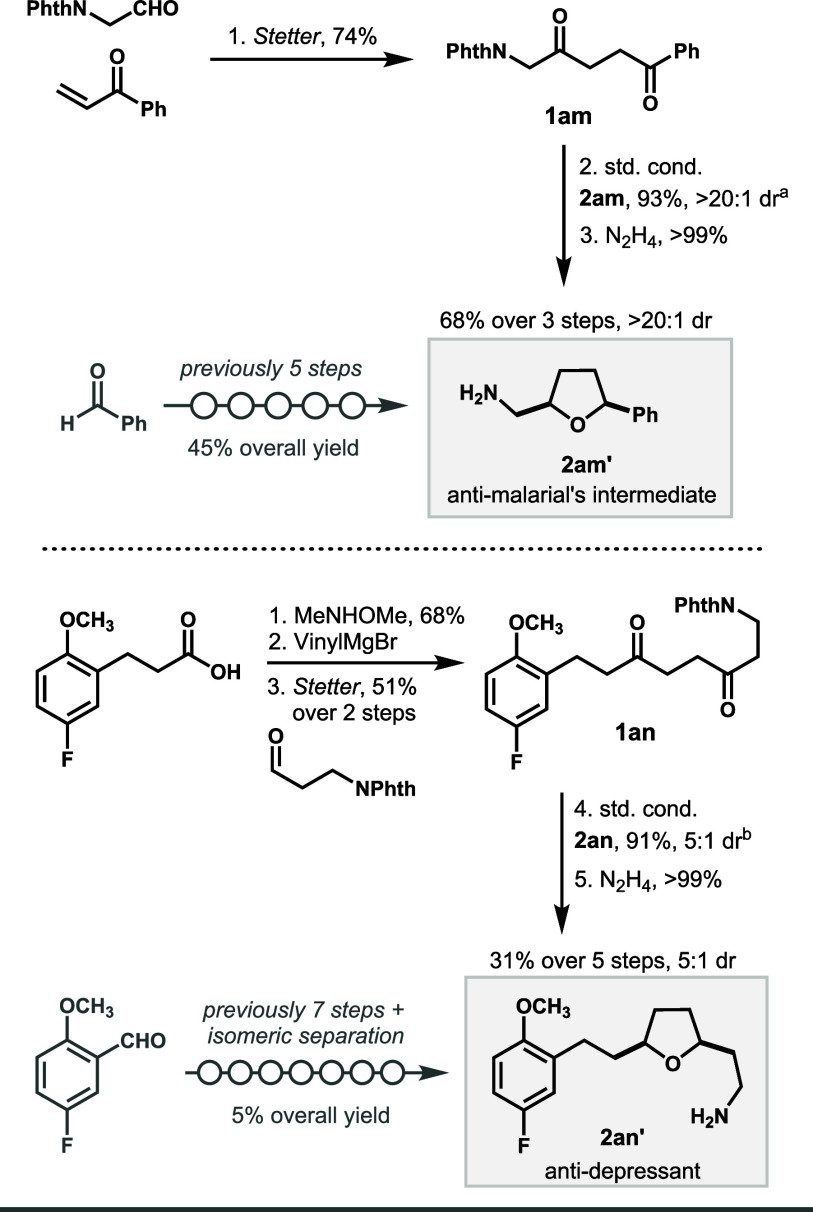
Streamlined Synthesis of Medicinally Relevant Compounds

Full synthesis details included in the Supporting Information. Standard conditions (std. cond.): **1** (0.2 mmol), 10 mol % **B3**, 4 Å MS (300 mg), 0.6 mL of 3:1 CPME:1,2-DCE, 100 bar H_2_, 18 h. Isolated yields are given. Diastereomeric ratios (dr) were determined by ^1^H NMR spectroscopy.
a 20 mol % **B3** used as a catalyst.
b 20 mol % **B1** used as a catalyst.

### Mechanistic Investigations

To gain insight into the reaction mechanism, we conducted deuterium labeling and a series of control experiments (Scheme 4). Subjecting **1a** to 20 bar of D_2_ under otherwise standard conditions led to deuterium incorporation at the α-, α′-, and β-positions relative to the oxygen atom (90%, 90%, and 30%, respectively; Scheme 4, eq 1). The β-deuteration is consistent with the formation of an oxocarbenium ion intermediate (**Int-1a**), which reversibly isomerizes to an enol ether (**Int-2a**) prior to reduction. Additionally, negligible deuteration at the β′-position suggests that initial carbonyl reduction occurs preferentially at the methyl ketone, thereby suppressing enolization at this site. This implies that a stereocenter adjacent to the methyl ketone could remain configurationally stable during the reaction and potentially transfer its chiral information.

**Scheme 4:**
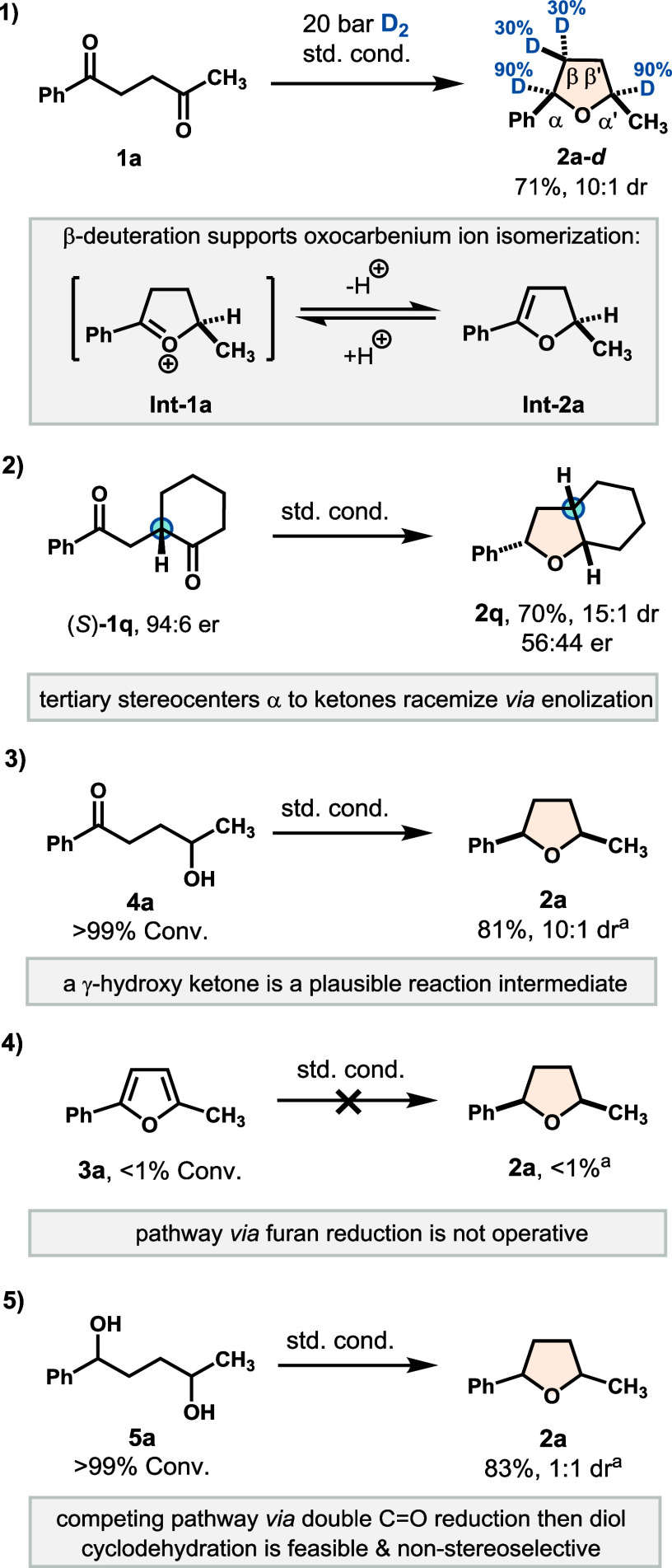
Deuterium Labeling and Control Experiments

Standard conditions (std. cond.): substrate (0.2 mmol), 100 bar H_2_, 10 mol % **B3**, 4 Å MS (300 mg), 0.6 mL of CPME, 18 h.
a Determined by ^1^H NMR analysis of the crude reaction mixture.

However, the enantioenriched substrate (*S*)-**1q** reacted to product **2q** with nearly complete racemization under standard conditions, indicating that enolization at the tertiary stereocenter took place (Scheme 4, eq 2; see the SI for details). Next, to interrogate the involvement of a hydroxy ketone intermediate, we subjected a preformed γ-hydroxy ketone (**4a**) to the standard reaction conditions (Scheme 4, eq 3). Smooth reductive cyclization occurred to afford the corresponding THF product **2a** in 81% yield with a 10:1 dr, closely matching the outcome obtained from corresponding diketone **1a**. This result further supports the primary pathway shown in Scheme 1D. An alternative reaction pathway could involve an initial Lewis acid-promoted cyclization to a furan intermediate, followed by hydrogenation. To probe this possibility, we subjected preformed furan **3a** to the standard reaction conditions (Scheme 4, eq 4). However, no conversion to the THF product was observed, thereby clearly ruling out this pathway. This result highlights the importance of suppressing furan formation, as it constitutes a nonproductive pathway.

A third possible pathway involves reduction of both ketones to a diol intermediate, followed by cycloetherification (Scheme 1D). Given that highly Lewis acidic boranes can promote S_N_1-type dehydrative diol cyclizations,^59^ we tested this possibility using preformed diol **5a** (Scheme 4, eq 5). Under our standard reaction conditions, the corresponding THF product **2a** formed in 83% yield but without diastereoselectivity (1:1 *cis*/*trans*). While this pathway is feasible, the absence of stereocontrol excludes it as the primary pathway, although it may compete in certain cases. This could explain why substrates bearing electron-rich aryl groups (**2e**, **2l**, and **2aj**) were obtained with lower dr, perhaps due to enhanced stabilization of carbocation intermediates favoring S_N_1-type cyclization. This experiment also suggests that α-epimerization of the product does not take place. While triarylboranes are capable of abstracting hydrides at the α-position to oxygen,^60^ this does not seem to occur in our system, as one would expect enrichment of the *cis*-product over time if it did.

Taken together, these results support the primary pathway shown in Scheme 1D, involving (i) FLP-catalyzed reduction of one ketone to a secondary alcohol, (ii) cyclodehydration to generate an oxocarbenium ion intermediate, and (iii) a stereodetermining, irreversible hydride transfer to form the cyclic ether product. Noteworthily, cyclization of the hydroxy ketone intermediate to the oxocarbenium ion species can be promoted both by the Lewis acidic triarylborane catalyst, consistent with prior reports,^61,62^ and by the strongly Brønsted acidic protonated ethers that are generated *in situ* upon H_2_ activation.^45^ In any case, both cyclization mechanisms ultimately originate from the borane catalyst and should not influence the stereochemical outcome of the reaction. Consequently, we focused our efforts on investigating the stereoselectivity-determining oxocarbenium reduction step.

To gain a deeper understanding of the origin of diastereoselectivity, we carried out DFT calculations using the THF derivative **2y** (Figure 1). This compound, bearing identical 2,5-diPh substituents, was chosen to simplify the analysis. All free energies were computed at 80 °C to match experimental conditions. The transition state leading to the *cis* product (TS_*cis*_) was predicted to be 2.1 kcal/mol lower in energy than the one leading to the *trans* product (TS_*trans*_), which is in excellent agreement with experiments (ΔΔ*G* ^‡^ = 2.1 kcal/mol; 20:1 dr, both calculated and observed). The barrier for reduction from the oxocarbenium intermediate **Int-1y** to TS_*cis*_ is relatively low (Δ*G* ^‡^ = 5.1 kcal/mol), consistent with a facile process. The *cis* and *trans* products are nearly isoenergetic (ΔΔ*G* = 0.3 kcal/mol), indicating that stereoselectivity is kinetically controlled by the catalyst. In contrast, for the six-membered THP product **2af**, both kinetics and thermodynamics favor the *cis* isomer (ΔΔ*G* = 2.6 kcal/mol; ΔΔ*G* ^‡^ = 4.1 kcal/mol; details in the SI). This comparison further validates the stereochemical model and underscores the importance of catalyst control, especially for THFs where the thermodynamic bias is minimal.

**Figure 1:**
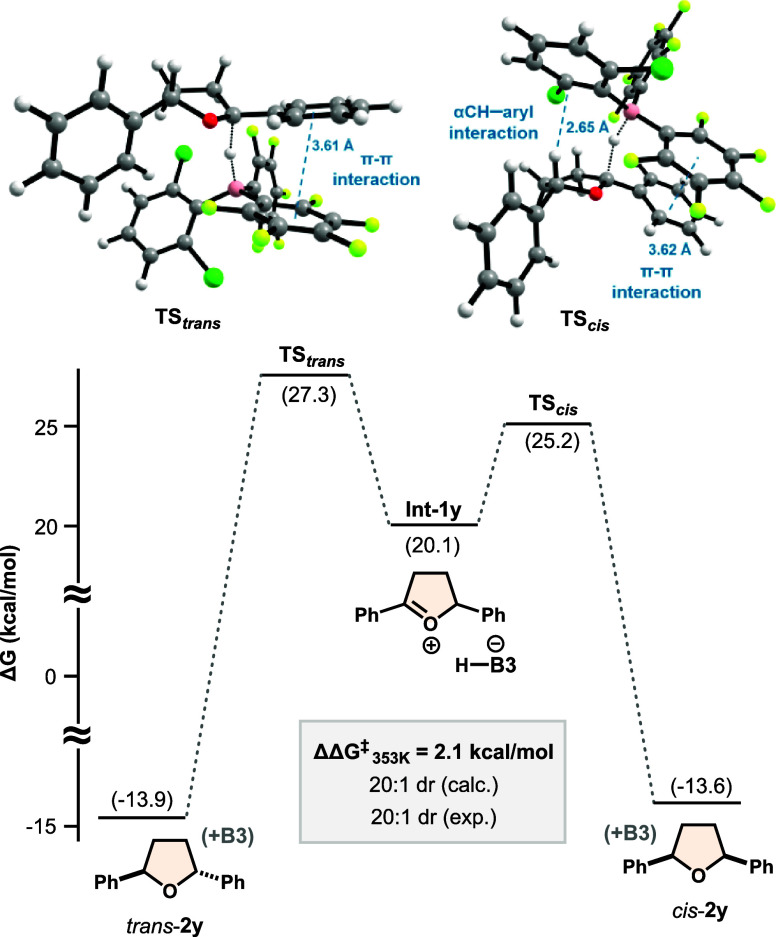
Calculated free energies of intermediates, products, and structures of the transition states. Level of theory: wB97X-D3/def2-TZVP/CPCM­(diethyl ether)/353.15K. The zero-energy level corresponds to **B3** + **1y** + 2H_2_; H_2_O is included in all other energy points but is not depicted for simplicity.

Noncovalent interaction (NCI) analysis^63^ revealed multiple weak van der Waals interactions between the catalyst and substrate **1y** (key interactions are highlighted in Figure 1; full details are provided in the SI). Both TS_*cis*_ and TS_*trans*_ exhibit offset arene–perfluoroarene π–π stacking (centroid separations ∼3.6 Å), which may assist in orienting the oxocarbenium intermediate. However, because high selectivity was also observed for products lacking aromatic groups (e.g., **2s**), this interaction alone cannot account for stereocontrol. A more general and consistent feature is a weak C–H···aryl edge interaction between the α-hydrogen of the substrate and an aryl ring of the catalyst, present in TS_*cis*_ (H···Csp^2^ = 2.65 Å) but absent in TS_*trans*_. A similar interaction was identified for TS_*cis*_ with catalyst **B1** (see the SI), supporting its broader relevance.^64,65^ These findings suggest that, in addition to steric effects, subtle noncovalent interactions may also play a role in stereocontrol.

## Conclusions

In summary, we have developed a general catalytic strategy for the synthesis of *cis*-2,5- and *cis*-2,6-disubstituted tetrahydrofurans and tetrahydropyrans via the reductive cycloetherification of diketones. This transformation is enabled by a simple triarylborane catalyst and employs dihydrogen as a clean, sustainable reductant. The method is straightforward, exhibits a broad substrate scope, and delivers products in high yields and diastereoselectivity. We demonstrated its synthetic utility through concise syntheses of pharmaceutically relevant cyclic ethers including a gram-scale application. Mechanistic studies support a pathway involving ketone reduction, cyclodehydration, and stereodetermining oxocarbenium ion reduction. DFT calculations revealed that noncovalent CH···aryl interactions between the catalyst and the substrate contribute to the observed stereoselectivity, offering insights that may guide future catalyst design. In a broader context, this work presents a pioneering example of a single metal-free catalyst orchestrating a tandem hydrogenation/cyclization reaction without the need for a transition-metal cocatalyst^62^ or exogenous acids,^66,67^ underscoring the versatility of main-group catalysis. We anticipate that the mechanistic insights presented herein will aid the development of enantioselective variants and inspire further metal-free hydrogenative synthesis of heterocycles.

## Supplementary Material



## Notes

Experimental procedures, optimization studies, mechanistic studies, DFT calculations, X-ray crystal data, characterization data, and NMR spectra of new compounds (PDF)

## Notes

CCDC 2444861 (**2ad**) and 2444863 (**2ae**) contain the supplementary crystallographic data for this paper. These data can be obtained free of charge via www.ccdc.cam.ac.uk/data_request/cif, or by emailing data_request@ccdc.cam.ac.uk, or by contacting The Cambridge Crystallographic Data Centre, 12 Union Road, Cambridge CB2 1EZ, UK; fax: + 44 1223 336033.

## Abbreviations

CPME: cyclopentyl methyl ether
MS: molecular sieves
NPhth: phthalimide
MTBE: *tert*-butyl methyl ether
std. cond.: standard conditions
