# Supplementary material for: Diastereoselective Synthesis of cis-α,α′-Disubstituted Cyclic Ethers via Borane-Catalyzed Reductive Cyclization of Diketones
Source: ACS Catal. 2026 Jan 2;16(2):1665–74. doi: 10.1021/acscatal.5c08127 (PMC12813991; doi:10.1021/acscatal.5c08127)
Supplement: Supplementary file 1 [file cs5c08127_si_001.pdf]

Supporting Information for

Diastereoselective Synthesis of *cis*-α,α′-Disubstituted Cyclic Ethers via Borane-Catalyzed Reductive Cyclization of Diketones

Nikolay V. Shcherbakov,[a] Nathaniel Potin,[a] Josep Mas-Roselló,[a]*

[a]Laboratorium für Organische Chemie, ETH Zürich, D-CHAB, Zürich 8093, Switzerland.

E-mail: josep.masrosello@org.chem.ethz.ch (J.M.R.)

Table of Contents

1. General information S2
2. Reaction optimization S3
3. Synthesis and characterization of diketone substrates S6
4. Synthesis and characterization of cyclic ether products S23
5. Gram-scale reaction S47
6. Mechanistic experiments S47
7. Computational details S55
7.1. General information S55
7.2. Calculation of the Lewis acidity of the boranes S56
7.3. Calculations on the oxocarbenium ion reduction S57
7.4. Cartesian coordinates of the calculated structures S64
8. Crystallographic data S101
9. NMR spectra of diketone substrates and cyclic ether products S104
10. References S190

1. General information

NMR spectra were recorded at ambient temperature with a Bruker DRX 400, a Bruker AV III 400 (400 MHz/100 MHz), a Bruker AVN 400 (400 MHz/101 MHz) and a Bruker AVN 500 (500 MHz/126 MHz) in $CDCl_3$. Chemical shifts (δ) are given in parts per million (ppm) relative to resonances of the solvent (^{1}H: δ = 7.26 for residual $CHCl_3$ peak; ^{13}C: δ = 77.2 for $CDCl_3$ peak). Mass-spectra were recorded on a Bruker maXis HRMS-ESI-QTOF instrument. Flash chromatography and plug filtrations were either performed manually using silica gel or automated using a Teledyne Isco CombiFlash MPLC system with prepacked silica columns. Solvents for extraction and chromatography were of HPLC grade or of technical quality and distilled before use. Analytical TLC was performed on unmodified Merck ready-to-use plates (TLC silica gel 60 F254); detection was achieved with a UV lamp (254 and 310 nm) and $KMnO_4$ stain. Melting points were recorded on a Büchi Melting Point M560. HPLC analyses were performed on a Dionex Ultimate 3000 analytical HPLC system with a diode array detector and chiral stationary phase columns. The different species were detected by UV (λ = 210 nm or 254 nm). Samples were dissolved in *n*-hexane/*i*PrOH (4:1) and filtered through an Acrodisc® CR 4 mm syringe filter with a 0.45 µm PTFE membrane prior to injection. The solvents used for hydrogenation reactions were dried over CaH_2, distilled and then stored over activated 4 Å molecular sieves before use. Tris(pentafluorophenyl)borane (**B1**) was purchased from Apollo Scientific Ltd. and used as received. Boranes **B2**[1] and **B3**[2] were synthesized following reported procedures. All boranes were stored in pre-dried Schlenk flasks under an argon atmosphere and kept in the freezer (-20 °C). Diketone substrates **1a**, **1y, 1ae,** and **1af** were purchased from commercial suppliers and used as received. Unless otherwise stated, the rest of chemical reagents were purchased from commercial suppliers and used as received.

2. Reaction optimization

Table S1. Solvent screening

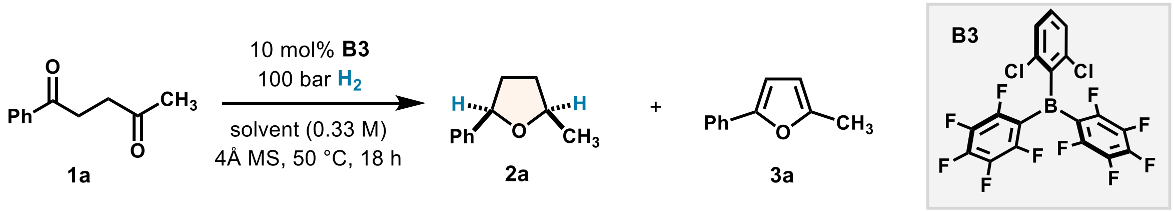

Entry	Solvent	2a %	2a dr	3a
1	*i*Pr_2O	48	11:1	2
2	Et_2O	65	11:1	<1
3	MTBE	86	11:1	<1
4	THF	<1	-	<1
5	CPME	78	11:1	2
6	2-MeTHF	5	11:1	<1
7	1,4-Dioxane	37	11:1	<1
8	Toluene	6	11:1	8
9	1,2-DCE	17	11:1	7
10	*n*-Hexane	13	11:1	3

Reaction conditions: **1a** (0.1 mmol), 10 mol% **B3**, 4Å MS (150 mg), 0.3 mL of the corresponding solvent, 100 bar H_2, 50 °C for 18 h. Yields and diastereomeric ratios (dr) were determined by ^{1}H NMR spectroscopy.

Table S2. Temperature screening

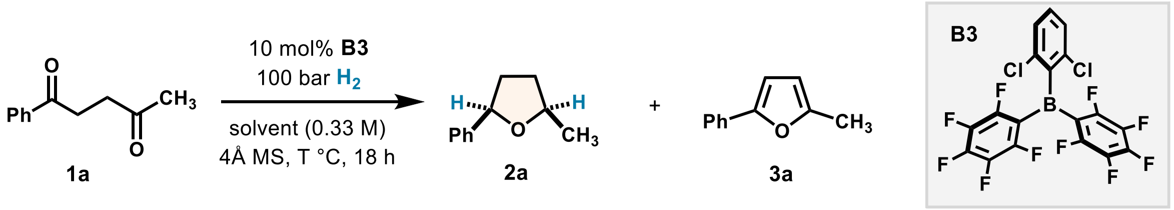

Entry	Temperature (°C)	Solvent	**2a** %	**2a** dr	**3a**
1	40	MTBE	48	11:1	2
2	50	MTBE	86	11:1	<1
3	50	CPME	78	11:1	2
4	60	MTBE	87	11:1	1
5	70	MTBE	89	-	2
6	80	MTBE	94	10:1	4
7	80	CPME	93	10:1	2

Reaction conditions: **1a** (0.1 mmol), 10 mol% **B3**, 4Å MS (150 mg), 0.3 mL of the corresponding solvent, 100 bar H_2, at the corresponding temperature for 18 h. Yields and diastereomeric ratios (dr) were determined by ^{1}H NMR spectroscopy.

Table S3. Influence of hydrogen pressure and time

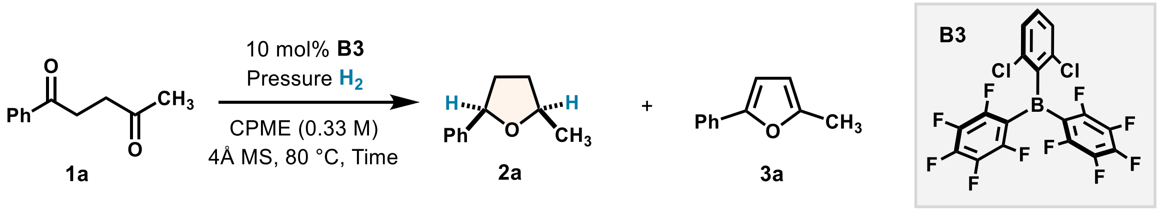

Entry	Pressure (bar)	Time (h)	**2a** %	**2a** dr	**3a**
1	100	18	93	10:1	2
2	50	18	73	10:1	6
3	25	18	67	10:1	6
4	25	4	21	10:1	3

Reaction conditions: **1a** (0.1 mmol), 10 mol% **B3**, 4Å MS (150 mg), 0.3 mL of CPME, corresponding H_2 pressure, at 50 °C for the corresponding time. Yields and diastereomeric ratios (dr) were determined by ^{1}H NMR spectroscopy.

Scheme S1. Reaction using a silane as reductant

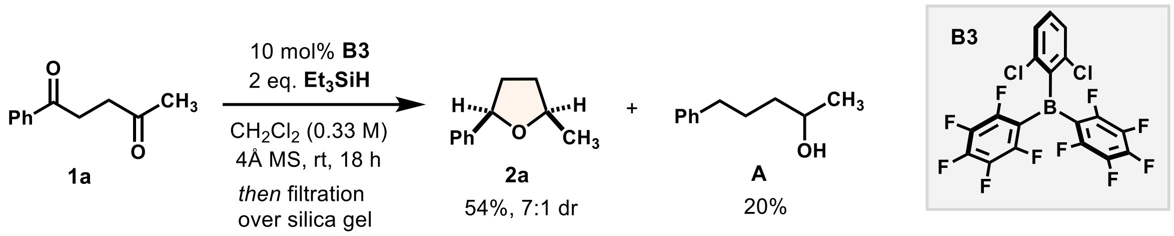

Reaction conditions: **1a** (0.2 mmol), 10 mol% **B3**, 4Å MS (300 mg), 0.6 mL of the CH_2Cl_2, Et_3SiH (2 eq.), at room temperature for 18 h. Then, crude reaction mixture filtered over a short plug of silica gel and concentrated under reduced pressure.

Quantitative ^{1}H NMR analysis of the crude reaction mixture using 1,3,5-trimethoxybenzene as an internal standard showed >95% consumption of starting material **1a** and formation of **2a** in 54% yield with a 7:1 dr. Additionally, c.a. 20% of a linear side product (**A**) was detected. The structure of **A** is tentatively assigned based on: i) literature reports of triarylborane-catalyzed reductive ring-opening of cyclic ethers to linear alcohols using silanes;[3] and ii) the presence of characteristic ^{1}H NMR signals at 3.85–3.79 ppm (m, 1H, C*H*OH) and 2.62 ppm (t, J = 7.7 Hz, 2H, PhCH_2R), which are consistent with reported data for **A**.[4] Partial *O*-silylation of **A** cannot be ruled out. Regardless, the key conclusion that using a silane as a reductant instead of H_2 leads to undesired side reactions and lower diastereoselectivity remains valid.

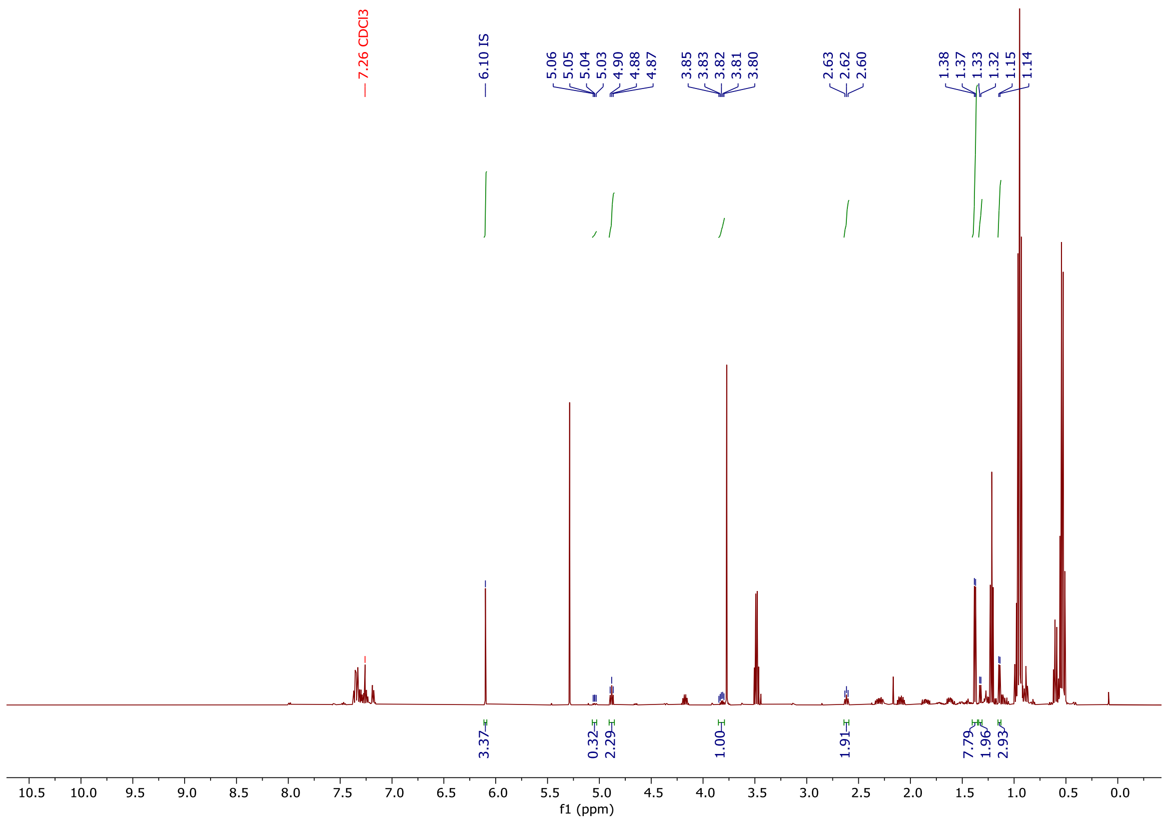

3. Synthesis and characterization of diketone substrates

General Procedure A: synthesis of 1,4-diketones

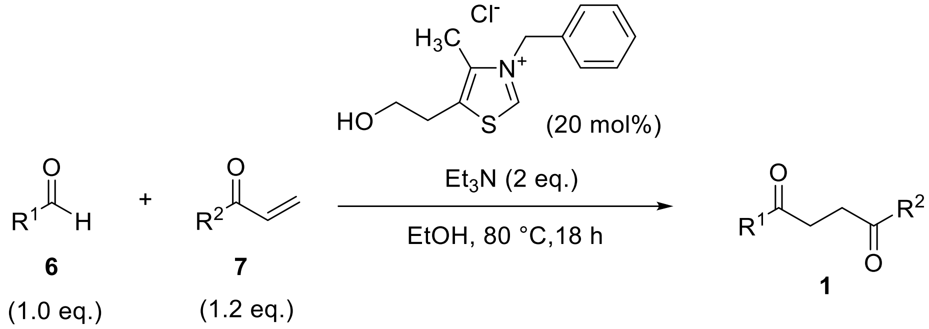

A flame-dried 20 mL microwave tube equipped with a stir bar was charged with the aldehyde **6** (5 mmol, 1 eq.), 3-benzyl-5-(2-hydroxyethyl)-4-methylthiazolium chloride (270 mg, 1 mmol, 20 mol%), and anhydrous EtOH (5 mL) under an argon atmosphere. Triethylamine (1.4 mL, 10 mmol, 2 eq.) and vinyl ketone **7** (6 mmol, 1.2 eq.) were added. The tube was sealed and the reaction mixture was heated to 80 °C stirring for 18 hours. After cooling the system to room temperature, water was added and the aqueous phase was extracted with CH_2Cl_2 (x3). The combined organic extracts were sequentially washed with 1 M aqueous solution of HCl, a saturated aqueous solution of $NaHCO_3$, and brine. The organic extracts were dried over Na_2SO_4, filtered, and concentrated under reduced pressure. The crude diketone products **1** were purified by flash chromatography on silica gel eluting with *n*-hexane/EtOAc mixtures.

1-(4-chlorophenyl)pentane-1,4-dione (1b)

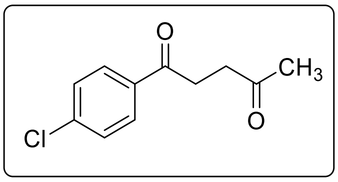

Synthesized from commercial 4-chlorobenzaldehyde (**6b**) and commercial methyl vinyl ketone (**7a**) according to the general procedure A. After chromatographic purification, the product was additionally recrystallized from *n*-hexane/EtOAc.

Beige solid (579 mg, 55%); $\mathbf{R}_f$ 0.25 (SiO_2; *n*-hexane/EtOAc 4:1); **^{1}H NMR** (400 MHz, $CDCl_3$) δ 7.94–7.88 (m, 2H), 7.46–7.39 (m, 2H), 3.25–3.20 (m, 2H), 2.90–2.85 (m, 2H), 2.25 (s, 3H); **^{13}C NMR** (101 MHz, $CDCl_3$) δ 207.2, 197.4, 139.7, 135.1, 129.6, 129.0, 37.1, 32.5, 30.2. The experimental data is in agreement with a previous report.[5]

methyl 4-(4-oxopentanoyl)benzoate (1c)

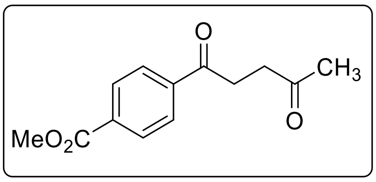

Synthesized from commercial methyl 4-formylbenzoate (**6c**) and commercial methyl vinyl ketone (**7a**) according to the general procedure A. After chromatographic purification, the product was additionally recrystallized from *n*-hexane/EtOAc.

Colorless solid (714 mg, 61%); **R*f*** 0.30 (SiO_2; *n*-hexane/EtOAc 2:1); **^{1}H NMR** (400 MHz, $CDCl_3$) δ 8.13–8.07 (m, 2H), 8.03–7.98 (m, 2H), 3.93 (s, 3H), 3.30–3.24 (m, 2H), 2.92–2.86 (m, 2H), 2.24 (s, 3H); **^{13}C NMR** (101 MHz, $CDCl_3$) δ 207.1, 198.2, 166.3, 140.0, 134.0, 129.9, 128.1, 52.5, 37.1, 32.8, 30.1. The experimental data is in agreement with a previous report.[6]

1-(4-(methylsulfonyl)phenyl)pentane-1,4-dione (1d)

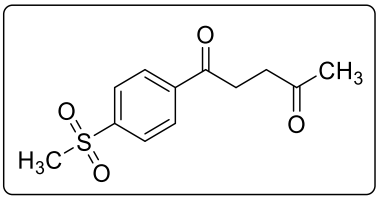

Synthesized from commercial 4-(methylsulfonyl)benzaldehyde (**6d**) and commercial methyl vinyl ketone (**7a**) according to the general procedure A. After chromatographic purification, the product was additionally recrystallized from *n*-hexane/EtOAc .

Colorless solid (850 mg, 67%); **R*f*** 0.25 (SiO_2; *n*-hexane/EtOAc 1:1); **^{1}H NMR** (500 MHz, $CDCl_3$) δ 8.17–8.11 (m, 2H), 8.07–8.01 (m, 2H), 3.29–3.25 (m, 2H), 3.07 (s, 3H), 2.95–2.91 (m, 2H), 2.26 (s, 3H); **^{13}C NMR** (126 MHz, $CDCl_3$) δ 206.9, 197.6, 144.3, 140.8, 129.1, 127.9, 44.5, 37.1, 32.9, 30.1; **HRMS** (ESI): *m/z* $[M+Na]^+$ calcd. for $C_{12}H_{14}NaO_4S^+$: 277.0505; found: 277.0501; **FTIR** ṽ (cm^{-1}): 3002, 2920, 1712, 1683, 1305, 1150.

1-(4-methoxyphenyl)pentane-1,4-dione (1e)

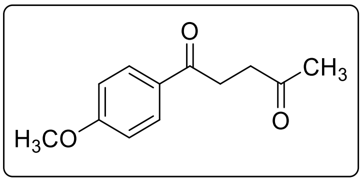

Synthesized from commercial 4-anisaldehyde (**6e**) and commercial methyl vinyl ketone (**7a**) according to the general procedure A.

Colorless solid (415 mg, 40%); **R*f*** 0.30 (SiO_2; *n*-hexane/EtOAc 2:1); **^{1}H NMR** (400 MHz, $CDCl_3$) δ 7.99–7.91 (m, 2H), 6.96–6.88 (m, 2H), 3.86 (s, 3H), 3.26–3.19 (m, 2H), 2.90–2.82 (m, 2H), 2.25 (s, 3H); **^{13}C NMR** (101 MHz, $CDCl_3$) δ 207.6, 197.1, 163.7, 130.4, 129.9, 113.8, 55.6, 37.3, 32.2, 30.3. The experimental data is in agreement with a previous report.[7]

1-(3-(4,4,5,5-tetramethyl-1,3,2-dioxaborolan-2-yl)phenyl)pentane-1,4-dione (1f)

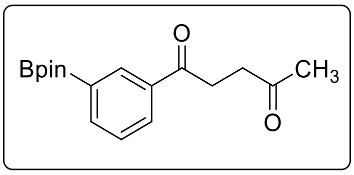

Synthesized from commercial 3-(4,4,5,5-Tetramethyl-1,3,2-dioxaborolan-2-yl)-benzaldehyde (**6f**) and commercial methyl vinyl ketone (**7a**) according to the general procedure A. After chromatographic purification, the product was additionally recrystallized from *n*-hexane/EtOAc.

Colorless solid (412 mg, 47%); **R*f*** 0.40 (SiO_2; *n*-hexane/EtOAc 2:1); **m.p.** = 95–97 °C; **^{1}H NMR** (400 MHz, $CDCl_3$) δ 8.43–8.37 (m, 1H), 8.06 (ddd, *J* = 7.8, 2.0, 1.3 Hz, 1H), 7.99 (dt, *J* = 7.3, 1.3 Hz, 1H), 7.50–7.43 (m, 1H), 3.35–3.30 (m, 2H), 2.89 (t, *J* = 6.3 Hz, 2H), 2.26 (s, 3H), 1.36 (s, 12H); **^{13}C NMR** (101 MHz, $CDCl_3$) δ 207.6, 198.8, 139.6, 136.2, 134.7, 130.7, 128.2, 84.3, 37.2, 32.7, 30.3, 25.0, the signal for the carbon directly attached to boron was not observed due to quadrupolar relaxation; **^{11}B NMR** (128 MHz, $CDCl_3$) δ 31.2; **HRMS** (ESI): *m/z* $[M+Na]^+$ calcd. for $C_{17}H_{23}BNaO_4^+$: 325.1582; found: 325.1580; **FTIR** ṽ (cm^{-1}): 2977, 2928, 1717, 1685, 1355, 1142.

4-(4-oxopentanoyl)benzonitrile (1g)

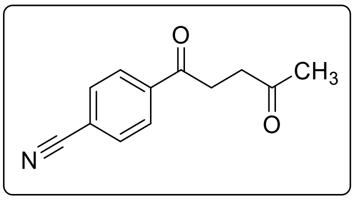

Synthesized from commercial 4-formylbenzonitrile (**6g**) and commercial methyl vinyl ketone (**7a**) according to the general procedure A. After chromatographic purification, the product was additionally recrystallized from *n*-hexane/EtOAc.

Colorless solid (586 mg, 58%); **R*f*** 0.35 (SiO_2; *n*-hexane/EtOAc 1:1); **^{1}H NMR** (500 MHz, $CDCl_3$) δ 8.09–8.03 (m, 2H), 7.80–7.74 (m, 2H), 3.25 (dd, *J* = 6.7, 5.5 Hz, 2H), 2.94–2.90 (m, 2H), 2.25 (s, 3H); **^{13}C NMR** (126 MHz, $CDCl_3$) δ 206.9, 197.4, 139.8, 132.6, 128.6, 118.1, 116.5, 37.1, 32.7, 30.1. The experimental data is in agreement with a previous report.[8]

***N*-(4-(4-oxopentanoyl)phenyl)acetamide (1h)**

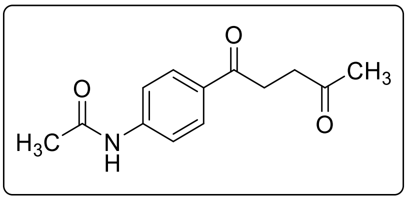

Synthesized from commercial *N*-(4-formylphenyl)acetamide (**6h**) and commercial methyl vinyl ketone (**7a**) according to the general procedure A. After chromatographic purification, the product was additionally recrystallized from EtOAc.

Yellowish solid (700 mg, 60%); **R_f** 0.20 (SiO_2; *n*-hexane/EtOAc 1:2); **^{1}H NMR** (500 MHz, $CDCl_3$) δ 8.02 (br. s, 1H), 7.89–7.86 (m, 2H), 7.58 (d, *J* = 8.6 Hz, 2H), 3.23–3.19 (m, 2H), 2.89–2.86 (m, 2H), 2.26 (s, 3H), 2.17 (s, 3H); **^{13}C NMR** (126 MHz, $CDCl_3$) δ 208.2, 197.3, 168.9, 142.7, 132.2, 129.5, 119.0, 37.2, 32.3, 30.3, 24.8; **HRMS** (ESI): *m/z* $[M+Na]^+$ calcd. for $C_{13}H_{15}NNaO_3^+$: 256.0944; found: 256.0940; **FTIR** ṽ (cm^{-1}): 3492, 3339, 2919, 1706, 1674, 1590.

1-(4-(hydroxymethyl)phenyl)pentane-1,4-dione (1i)

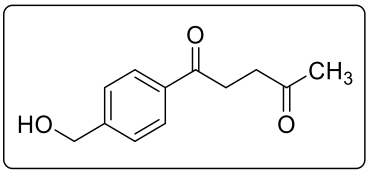

Synthesized from commercial 4-(hydroxymethyl)benzaldehyde (**6i**) and commercial methyl vinyl ketone (**7a**) according to the general procedure A. After chromatographic purification, the product was additionally recrystallized from *n*-hexane/EtOAc.

Colorless solid (412 mg, 40%); **R_f** 0.15 (SiO_2; *n*-hexane/EtOAc 1:1); **^{1}H NMR** (400 MHz, $CDCl_3$) δ 7.99–7.95 (m, 2H), 7.45 (d, *J* = 8.5 Hz, 2H), 4.77 (d, *J* = 5.9 Hz, 2H), 3.27 (dd, *J* = 6.8, 5.8 Hz, 2H), 2.91–2.87 (m, 2H), 2.26 (s, 3H), 1.93 (t, *J* = 6.0 Hz, 1H); **^{13}C NMR** (101 MHz, $CDCl_3$) δ 207.6, 198.3, 146.4, 136.0, 128.5, 126.8, 64.8, 37.2, 32.6, 30.3; **HRMS** (ESI): *m/z* $[M+Na]^+$ calcd. for $C_{12}H_{14}NaO_3^+$: 229.0835; found: 229.0833; **FTIR** ṽ (cm^{-1}): 3398, 2914, 1712, 1682, 1359, 1212.

1-(naphthalen-2-yl)pentane-1,4-dione (1j)

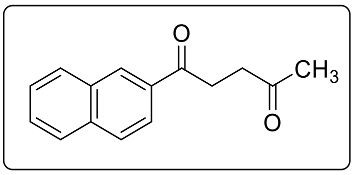

Synthesized from commercial 2-naphthaldehyde (**6j**) and commercial methyl vinyl ketone (**7a**) according to the general procedure A. After chromatographic purification, the product was additionally recrystallized from *n*-hexane/EtOAc.

Colorless solid (720 mg, 64%); **R_f** 0.30 (SiO_2; *n*-hexane/EtOAc 4:1); **^{1}H NMR** (400 MHz, $CDCl_3$) δ 8.55–8.50 (m, 1H), 8.03 (dd, *J* = 8.6, 1.8 Hz, 1H), 8.00–7.93 (m, 1H), 7.93–7.85 (m, 2H), 7.58 (dddd, *J* = 19.3, 8.1, 6.9, 1.4 Hz, 2H), 3.46–3.39 (m, 2H), 2.95 (t, *J* = 6.3 Hz, 2H), 2.29 (s, 3H); **^{13}C NMR** (101 MHz, $CDCl_3$) δ 207.6, 198.6, 135.8, 134.1, 132.7, 129.9, 129.7, 128.61, 128.58, 127.9, 126.9, 123.9, 37.3, 32.6, 30.3. The experimental data is in agreement with a previous report.[7]

1-(thiophen-3-yl)pentane-1,4-dione (1k)

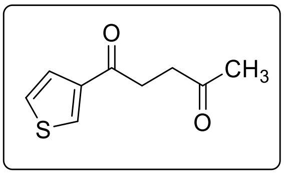

Synthesized from commercial 3-thiophenecarboxaldehyde (**6k**) and commercial methyl vinyl ketone (**7a**) according to the general procedure A.

Brownish oil (364 mg, 40%); **R*f*** 0.35 (SiO_2; *n*-hexane/EtOAc 2:1); **^{1}H NMR** (400 MHz, $CDCl_3$) δ 8.09 (dd, *J* = 2.9, 1.2 Hz, 1H), 7.55 (dd, *J* = 5.1, 1.2 Hz, 1H), 7.31 (dd, *J* = 5.1, 2.9 Hz, 1H), 3.21–3.17 (m, 2H), 2.86 (t, *J* = 6.3 Hz, 2H), 2.25 (s, 3H); **^{13}C NMR** (100 MHz, $CDCl_3$) δ 207.4, 193.0, 142.0, 132.1, 127.0, 126.5, 37.1, 33.6, 30.2; **HRMS** (ESI): *m/z* $[M+Na]^+$ calcd. for $C_9H_{10}NaO_2S^+$: 205.0294; found: 205.0292; **FTIR** ṽ (cm^{-1}): 3101, 2907, 1712, 1668, 1407, 1158.

1-(1-tosyl-1*H*-indol-3-yl)pentane-1,4-dione (1l)

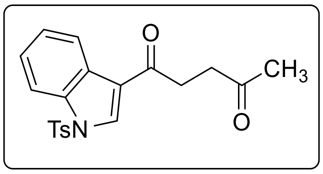

Synthesized from commercial 1-tosyl-1*H*-indole-3-carbaldehyde (**6l**) and commercial methyl vinyl ketone (**7a**) according to the general procedure A. After chromatographic purification, the product was additionally recrystallized from *n*-hexane/EtOAc.

Beige solid (1.09 g, 59%); **R*f*** 0.20 (SiO_2; *n*-hexane/EtOAc 2:1); **m.p.** = 123–125 °C; **^{1}H NMR** (500 MHz, $CDCl_3$) δ 8.30 (s, 1H), 8.29–8.26 (m, 1H), 7.95–7.91 (m, 1H), 7.86–7.82 (m, 2H), 7.38–7.27 (m, 4H), 3.22 (t, *J* = 6.3 Hz, 2H), 2.91 (t, *J* = 6.3 Hz, 2H), 2.37 (s, 3H), 2.26 (s, 3H); **^{13}C NMR** (126 MHz, $CDCl_3$) δ 207.4, 194.3, 146.1, 135.0, 134.7, 132.0, 130.4, 127.7, 127.3, 125.8, 124.9, 123.1, 121.0, 113.2, 37.0, 33.7, 30.2, 21.8; **HRMS** (ESI): *m/z* $[M+Na]^+$ calcd. for $C_{20}H_{19}NNaO_4S^+$: 392.0927; found: 392.0926; **FTIR** ṽ (cm^{-1}): 3124, 3054, 2911, 1713, 1665, 1369, 1164.

1-phenyloctane-1,4-dione (1m)

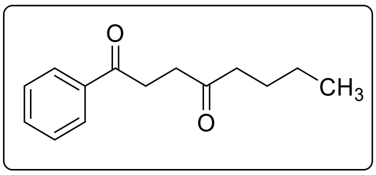

Synthesized from commercial valeraldehyde (**6m**) and commercial phenyl vinyl ketone (**7b**) according to the general procedure A. After chromatographic purification, the product was additionally recrystallized from *n*-hexane.

Colorless solid (371 mg, 34%); **R*f*** 0.50 (SiO_2; *n*-hexane/EtOAc 4:1); **^{1}H NMR** (400 MHz, $CDCl_3$) δ 8.02–7.94 (m, 2H), 7.60–7.51 (m, 1H), 7.50–7.40 (m, 2H), 3.31–3.23 (m, 2H), 2.89–2.81 (m, 2H), 2.52 (t, *J* = 7.5 Hz, 2H), 1.66–1.54 (m, 2H), 1.40–1.27 (m, 2H), 0.91 (t, *J* = 7.3 Hz, 3H); **^{13}C NMR** (101 MHz, $CDCl_3$) δ 209.9, 198.8, 136.8, 133.2, 128.7, 128.2, 42.8,

36.3, 32.5, 26.1, 22.5, 14.0. The experimental data is in agreement with a previous report.[9]

1,5-diphenylpentane-1,4-dione (1n)

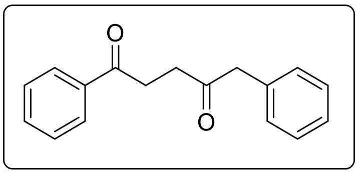

Synthesized from commercial phenylacetaldehyde (**6n**) and commercial phenyl vinyl ketone (**7b**) according to the general procedure A.

Beige solid (416 mg, 33%); **R*f*** 0.35 (SiO_2; *n*-hexane/EtOAc 4:1); **^{1}H NMR** (400 MHz, $CDCl_3$) δ 8.00–7.93 (m, 2H), 7.59–7.53 (m, 1H), 7.49–7.41 (m, 2H), 7.38–7.32 (m, 2H), 7.30–7.24 (m, 3H), 3.83 (s, 2H), 3.29–3.23 (m, 2H), 2.93–2.88 (m, 2H); **^{13}C NMR** (101 MHz, $CDCl_3$) δ 207.2, 198.6, 136.7, 134.4, 133.3, 129.6, 128.9, 128.7, 128.2, 127.2, 50.4, 35.8, 32.7. The experimental data is in agreement with a previous report.[10]

5-(benzyloxy)-1-phenylpentane-1,4-dione (1o)

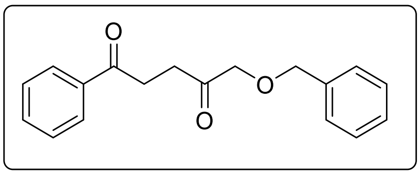

Synthesized from commercial benzyloxyacetaldehyde (**6o**) and commercial phenyl vinyl ketone (**7b**) according to the general procedure A.

Beige solid (960 mg, 68%); **R*f*** 0.25 (SiO_2; *n*-hexane/EtOAc 4:1); **^{1}H NMR** (400 MHz, $CDCl_3$) δ 8.01–7.95 (m, 2H), 7.61–7.52 (m, 1H), 7.51–7.43 (m, 2H), 7.42–7.28 (m, 5H), 4.65 (s, 2H), 4.22 (s, 2H), 3.38–3.29 (m, 2H), 2.95–2.87 (m, 2H); **^{13}C NMR** (101 MHz, $CDCl_3$) δ 207.8, 198.4, 137.4, 136.7, 133.3, 128.7, 128.6, 128.2, 128.11, 128.09, 75.3, 73.6, 32.8, 32.3. The experimental data is in agreement with a previous report.[7]

***rac*-6,10-dimethyl-1-phenylundec-9-ene-1,4-dione (1p)**

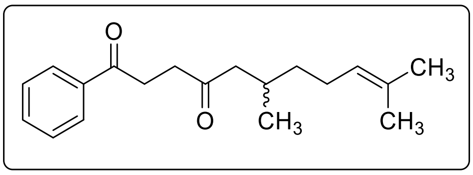

Synthesized from commercial (±)-citronellal (**6p**) and commercial phenyl vinyl ketone (**7b**) according to the general procedure A.

Yellowish oil (592 mg, 41%); **R*f*** 0.60 (SiO_2; *n*-hexane/EtOAc 8:1); **^{1}H NMR** (400 MHz, $CDCl_3$) δ 8.01–7.95 (m, 2H), 7.59–7.52 (m, 1H), 7.48–7.43 (m, 2H), 5.09 (th, *J* = 7.1, 1.4 Hz, 1H), 3.27 (t, *J* = 6.3 Hz, 2H), 2.92–2.77 (m, 2H), 2.52 (dd, *J* = 15.8, 5.6 Hz, 1H), 2.33 (dd, *J* = 15.8, 8.3 Hz, 1H), 2.11 – 1.90 (m, 3H), 1.69–1.65 (m, 3H), 1.63–1.58 (m, 3H), 1.40–1.27 (m, 1H), 1.27–1.15 (m, 1H), 0.92 (d, *J* = 6.7 Hz, 3H); **^{13}C NMR** (101 MHz, $CDCl_3$) δ 209.5,

198.8, 136.9, 133.2, 131.6, 128.7, 128.2, 124.5, 50.5, 37.2, 36.9, 32.4, 29.2, 25.8, 25.6, 19.9, 17.8. The experimental data is in agreement with a previous report.[7]

2-(2-oxo-2-phenylethyl)cyclohexan-1-one (1q)

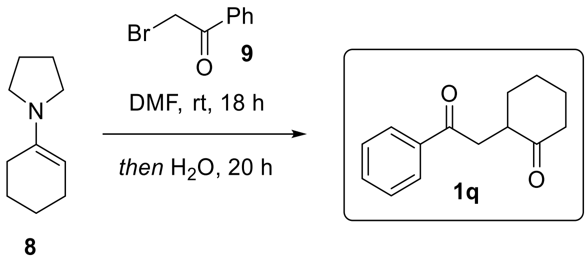

Synthesized from commercial 1-pyrrolidino-1-cyclohexene **8** (0.81 mL, 5.0 mmol) following a reported procedure.[11]

Colorless oil (527 mg, 49%); **R*f*** 0.40 (SiO_2; *n*-hexane/EtOAc 4:1); **^{1}H NMR** (400 MHz, $CDCl_3$) δ 8.02–7.96 (m, 2H), 7.59–7.51 (m, 1H), 7.50–7.40 (m, 2H), 3.60 (dd, *J* = 17.7, 6.6 Hz, 1H), 3.23–3.11 (m, 1H), 2.68 (dd, *J* = 17.7, 5.7 Hz, 1H), 2.46–2.38 (m, 2H), 2.24–2.10 (m, 2H), 1.95–1.85 (m, 1H), 1.84–1.60 (m, 2H), 1.45 (qd, *J* = 12.8, 3.9 Hz, 1H); **^{13}C NMR** (101 MHz, $CDCl_3$) δ 211.7, 198.8, 137.2, 133.1, 128.7, 128.2, 46.6, 42.1, 38.5, 34.5, 28.1, 25.5. The experimental data is in agreement with a previous report.[11]

(*S*)-2-(2-oxo-2-phenylethyl)cyclohexan-1-one (*S*-1q)

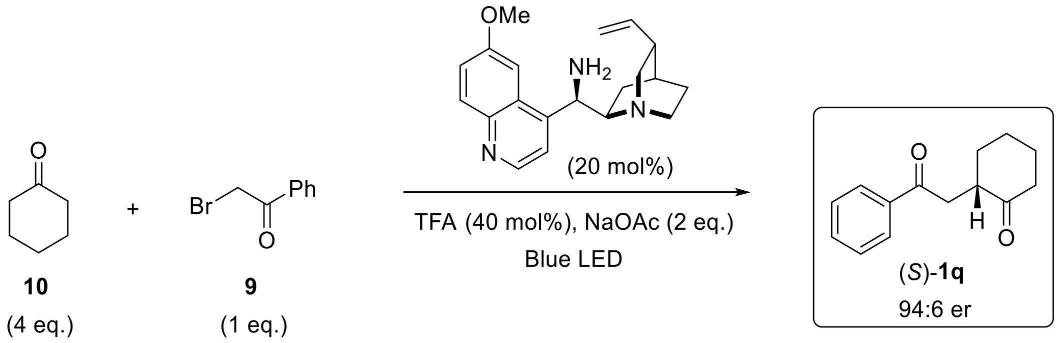

Synthesized from commercial cyclohexanone **10** (0.83 mL, 8.00 mmol) and 2-bromo-1-phenylethanone **9** (0.40 g, 2.00 mmol) following a reported procedure.[12] The reaction was conducted in an ETHos photoreactor[13] under blue LEDs. After purification, 170 mg of pure product were obtained (39% yield). NMR data matched the one described above for *rac*-**1q**.

The enantiomeric ratio was determined by chiral stationary phase HPLC with an IC column (*n*-hexane/^{i}PrOH 10%, 25 °C, 1 mL/min), UV detection at λ = 254 nm, t_R (major) = 27.3 min, t_R (minor) = 27.3 min, er = 94:6.

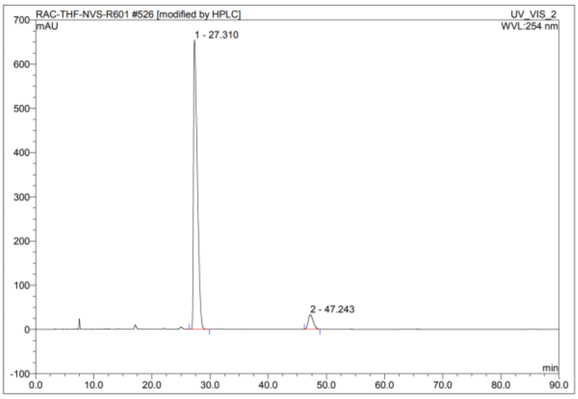

No.	Ret.Time min	Peak Name	Height mAU	Area mAU*min	Rel.Area %	Amount	Type
1	27.31	n.a.	653.738	493.947	93.77	n.a.	BMB*
2	47.24	n.a.	32.958	32.812	6.23	n.a.	BMB*
Total:			686.696	526.759	100.00	0.000	

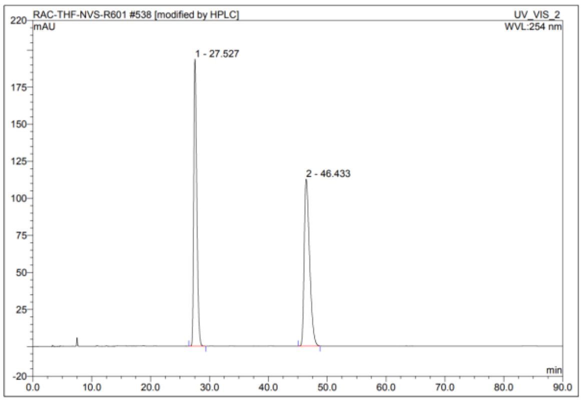

No.	Ret.Time min	Peak Name	Height mAU	Area mAU*min	Rel.Area %	Amount	Type
1	27.53	n.a.	193.535	116.449	49.53	n.a.	BMB*
2	46.43	n.a.	112.604	118.643	50.47	n.a.	BMB*
Total:			306.139	235.092	100.00	0.000	

Note: the absolute (*S*)-configuration for compound **1q** was assigned by comparing the elution order of its enantiomers with literature data using the same chiral stationary phase (Daicel Chiralpak IC column).[12]

1,1'-(1,4-phenylene)bis(pentane-1,4-dione) (1r)

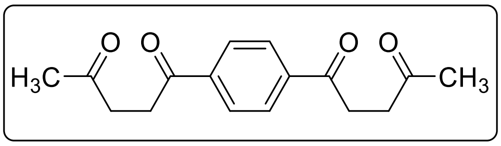

Synthesized from commercial terephthalaldehyde (**6r**) and commercial methyl vinyl ketone (**7a**) (2.4 eq.) according to the general procedure A. 40 mol% of the thiazolium catalyst and 4 eq. of Et_3N were used. After chromatographic purification, the product was additionally recrystallized from EtOAc.

Colorless solid (346 mg, 25%); $\mathbf{R_f}$ 0.25 (SiO_2; *n*-hexane/EtOAc 1:1); **m.p.** = 155–157 °C; **^{1}H NMR** (400 MHz, $CDCl_3$) δ 8.05 (s, 4H), 3.31–3.27 (m, 4H), 2.94–2.89 (m, 4H), 2.27 (s, 6H); **^{13}C NMR** (101 MHz, $CDCl_3$) δ 207.2, 198.2, 140.0, 128.4, 37.2, 32.9, 30.2; **HRMS** (ESI): *m/z* $[M+Na]^+$ calcd. for $C_{16}H_{18}NaO_4^+$: 297.1097; found: 297.1096; **FTIR** ṽ (cm^{-1}): 3044, 2906, 1705, 1673, 1404, 1318.

dodecane-3,6-dione (1s)

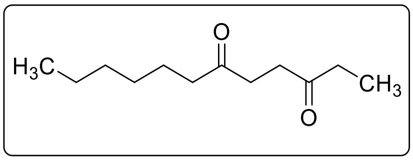

Synthesized from commercial heptanal (**6s**) and commercial ethyl vinyl ketone (**7c**) according to the general procedure A.

Colorless solid (502 mg, 51%); $\mathbf{R_f}$ 0.25 (SiO_2; *n*-hexane/EtOAc 8:1); **m.p.** = 40–42 °C; **^{1}H NMR** (500 MHz, $CDCl_3$) δ 2.69–2.66 (m, 4H), 2.51–2.42 (m, 4H), 1.61–1.53 (m, 1H), 1.33–1.24 (m, 7H), 1.06 (t, *J* = 7.4 Hz, 3H), 0.89–0.86 (m, 3H); **^{13}C NMR** (126 MHz, $CDCl_3$) δ 210.3, 210.0, 43.0, 36.2, 36.1, 35.7, 31.7, 29.0,

23.9, 22.6, 14.2, 7.9; **HRMS** (ESI): *m/z* $[M+Na]^+$ calcd. for $C_{12}H_{23}O_2^+$: 199.1693; found: 199.1691; **FTIR** ṽ (cm^{-1}): 2928, 2857, 2859, 1709, 1409, 1373.

1-phenylhexane-2,5-dione (1t)

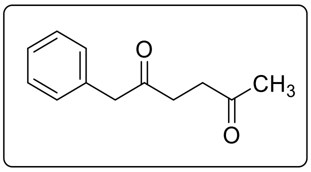

Synthesized from commercial phenylacetaldehyde (**6t**) and commercial methyl vinyl ketone (**7a**) according to the general procedure A.

Yellowish oil (390 mg, 41%); **R_f** 0.20 (SiO_2; *n*-hexane/EtOAc 4:1); **^{1}H NMR** (400 MHz, $CDCl_3$) δ 7.35–7.29 (m, 2H), 7.28–7.23 (m, 1H), 7.23–7.16 (m, 2H), 3.74 (s, 2H), 2.73–2.65 (m, 4H), 2.16 (s, 3H); **^{13}C NMR** (101 MHz, $CDCl_3$) δ 207.2, 207.1, 134.3, 129.6, 128.8, 127.1, 50.2, 37.1, 35.6, 30.0. The experimental data is in agreement with a previous report.[14]

1-phenylhexane-1,5-dione (1u)

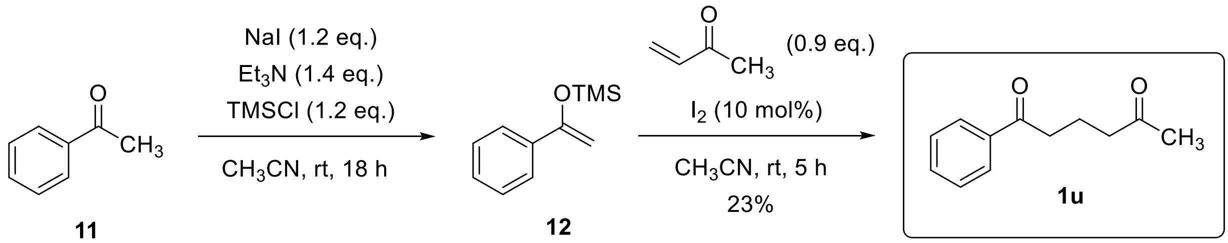

Step 1: In a flame-dried round-bottom flask equipped with a stir bar, under an argon atmosphere triethylamine (0.98 mL, 7 mmol, 1.4 eq.), acetophenone **11** (0.585 mL, 5 mmol, 1 eq.), and trimethylchlorosilane (0.762 mL, 6 mmol, 1.2 eq.) were successively added to a solution of NaI (0.9 g, 6 mmol, 1.2 eq.) in CH_3CN (5 mL) at room temperature. The mixture was stirred overnight and monitored. All volatiles were removed in vacuo. The residue was redissolved in *n*-hexane with rigorous stirring. The mixture was filtered and washed with *n*-hexane. The filtrate was concentrated. The resulting silyl enol ether **12** was used in the next step without further purification.

Step 2: Following a literature report,[15] in a flame-dried round-bottom flask equipped with a stir bar, under an argon atmosphere, a solution of crude 1-phenyl-1-trimethylsiloxyethylene **12** in CH_3CN (5 mL) was added dropwise to a solution of methyl vinyl ketone **7a** (5 mmol, 0.9 eq.) and iodine (10 mol%) in CH_3CN (5 mL) and stirred at room temperature for 5 h. Sodium thiosulfate was added to quench the reaction. The mixture was extracted with EtOAc (x3), the combined organic layers were dried over anhydrous Na_2SO_4, filtered, and the filtrate was

concentrated under reduced pressure. The residue was purified by silica gel flash column chromatography (eluting with *n*-hexane:EtOAc 100:0 to 80:20) to afford the product **1u** (0.22 g, 23% yield) as a yellowish solid. **R*f*** 0.25 (SiO_2; *n*-hexane/EtOAc 4:1); **^{1}H NMR** (400 MHz, $CDCl_3$) δ 8.00–7.92 (m, 2H), 7.60–7.51 (m, 1H), 7.50–7.41 (m, 2H), 3.02 (t, *J* = 7.0 Hz, 2H), 2.57 (t, *J* = 7.0 Hz, 2H), 2.15 (s, 3H), 2.02 (p, *J* = 7.0 Hz, 2H); **^{13}C NMR** (101 MHz, $CDCl_3$) δ 208.6, 199.9, 137.0, 133.2, 128.8, 128.2, 42.7, 37.5, 30.1, 18.3. The experimental data is in agreement with a previous report.[15]

1-cyclohexyl-4-phenylbutane-1,4-dione (1v)

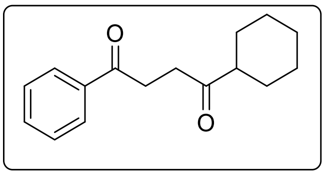

Synthesized from commercial cyclohexanecarboxaldehyde (**6v**) and commercial phenyl vinyl ketone (**7b**) according to the general procedure A.

Yellowish solid (721 mg, 59%); **R*f*** 0.55 (SiO_2; *n*-hexane/EtOAc 4:1); **^{1}H NMR** (400 MHz, $CDCl_3$) δ 8.02–7.94 (m, 2H), 7.58–7.52 (m, 1H), 7.49–7.41 (m, 2H), 3.30–3.23 (m, 2H), 2.93–2.85 (m, 2H), 2.46 (tt, *J* = 11.3, 3.5 Hz, 1H), 1.97–1.89 (m, 2H), 1.84–1.76 (m, 2H), 1.71–1.64 (m, 1H), 1.44–1.16 (m, 5H); **^{13}C NMR** (101 MHz, $CDCl_3$) δ 212.8, 198.9, 136.9, 133.2, 128.7, 128.2, 51.1, 34.4, 32.5, 28.7, 26.0, 25.8. The experimental data is in agreement with a previous report.[16]

benzyl 4-(4-oxo-4-phenylbutanoyl)piperidine-1-carboxylate (1w)

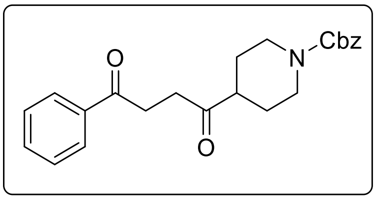

Synthesized from commercial benzyl 4-formylpiperidine-1-carboxylate (**6w**) and commercial phenyl vinyl ketone (**7b**) according to the general procedure A.

Yellowish oil (1130 mg, 60%); **R*f*** 0.25 (SiO_2; *n*-hexane/EtOAc 2:1); **^{1}H NMR** (500 MHz, $CDCl_3$) δ 7.98–7.96 (m, 2H), 7.58–7.53 (m, 1H), 7.48–7.44 (m, 2H), 7.38–7.29 (m, 5H), 5.13 (s, 2H), 4.20 (br. s, 2H), 3.33–3.27 (m, 2H), 2.95–2.86 (m, 4H), 2.65 (tt, *J* = 11.2, 3.7 Hz, 1H), 1.93 (br. s, 2H), 1.67–1.58 (m, 2H); **^{13}C NMR** (126 MHz, $CDCl_3$) δ 210.7, 198.6, 155.3, 136.9, 136.7, 133.3, 128.7, 128.6, 128.2, 128.1, 128.0, 67.2, 48.6, 43.6, 34.3, 32.5, 27.6; **HRMS** (ESI): *m/z* $[M+Na]^+$ calcd. for $C_{23}H_{25}NNaO_4^+$: 402.1676; found: 402.1672; **FTIR** ṽ (cm^{-1}): 3060, 2946, 2856, 1682, 1427, 1223.

5,5-dimethyl-1-phenylhexane-1,4-dione (1x)

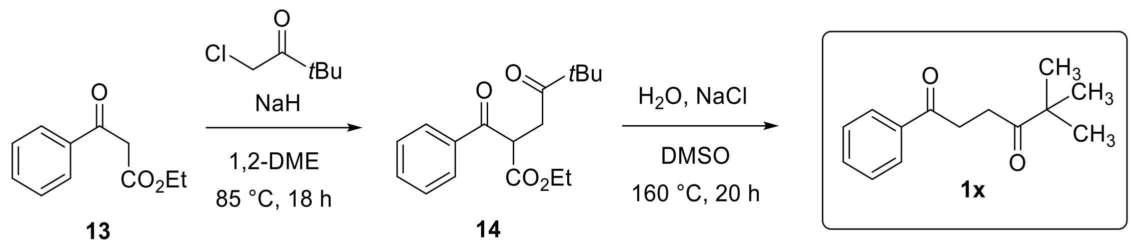

Synthesized from commercial ethyl 3-oxo-3-phenylpropanoate **13** (1.38 g, 6.842 mmol) following a reported two-step procedure.[17] Product **1x** purified by reversed-phase column chromatography with C18 as solid phase, eluting with a gradient from H_2O:CH_3CN 80:20 to 0:100.

Colorless liquid (0.49 g, 33% yield over 2 steps). **^{1}H NMR** (400 MHz, $CDCl_3$) δ 8.01–7.96 (m, 2H), 7.58–7.52 (m, 1H), 7.49–7.43 (m, 2H), 3.28–3.22 (m, 2H), 2.99–2.94 (m, 2H), 1.21 (s, 9H); **^{13}C NMR** (101 MHz, $CDCl_3$) δ 214.8, 199.1, 137.0, 133.2, 128.7, 128.2, 44.2, 32.6, 30.9, 26.8. The experimental data is in agreement with a previous report.[16]

1-(4-nitrophenyl)-4-phenylbutane-1,4-dione (1z)

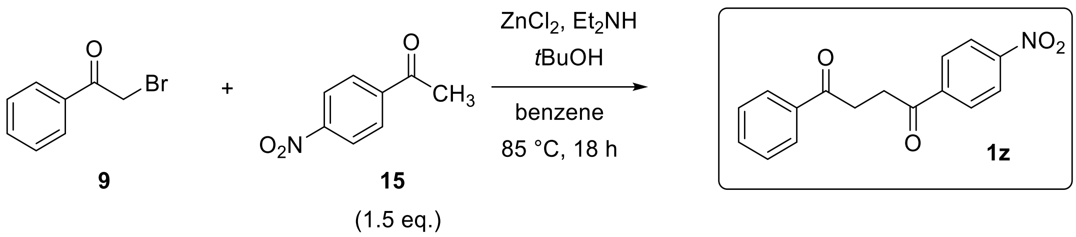

Synthesized from commercial 2-bromo-1-phenylethanone **9** (1.99 g, 10 mmol) and 1-(4-nitrophenyl)ethanone **15** (2.48 g, 15 mmol) following a reported procedure.[18] After chromatographic purification, the product was additionally recrystallized from *n*-hexane/EtOAc.

Yellow solid (1.25 g, 44%); **R*f*** 0.60 (SiO_2; *n*-hexane/EtOAc 1:1); **^{1}H NMR** (400 MHz, $CDCl_3$) δ 8.38–8.29 (m, 2H), 8.22–8.16 (m, 2H), 8.06–8.00 (m, 2H), 7.64–7.55 (m, 1H), 7.53–7.46 (m, 2H), 3.54–3.44 (m, 4H); **^{13}C NMR** (101 MHz, $CDCl_3$) δ 198.3, 197.5, 150.5, 141.4, 136.6, 133.5, 129.3, 128.8, 128.2, 124.0, 33.1, 32.7. The experimental data is in agreement with a previous report.[19]

1-phenyl-4-(4-(trifluoromethyl)phenyl)butane-1,4-dione (1aa)

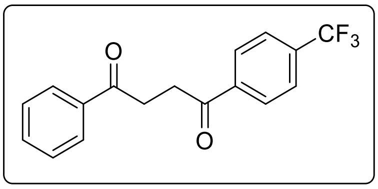

Synthesized from commercial 4-(trifluoromethyl)benzaldehyde (**6aa**) and commercial phenyl vinyl ketone (**7b**) according to the general procedure A.

Colourless solid (781 mg, 51%); **R*f*** 0.40 (SiO_2; *n*-hexane/EtOAc 4:1); **^{1}H NMR** (400 MHz, $CDCl_3$) δ 8.18–8.11 (m, 2H), 8.08–8.00 (m, 2H), 7.79–7.72 (m, 2H), 7.63–7.55 (m, 1H), 7.53–7.44 (m, 2H), 3.52–3.43 (m, 4H); **^{13}C NMR** (101 MHz, $CDCl_3$) δ 198.5, 198.0, 139.6, 136.7 134.6, (q, J = 32.7 Hz), 133.4, 128.8, 128.6, 128.3, 125.8 (q, J = 3.8 Hz), 32.9, 32.7, the carbon of the CF_3 group was not detected, likely because the large $^{1}J_{C–F}$ coupling (~270 Hz) splits the signal into a weak quartet that is buried in the noise/other signals; **^{19}F NMR** (377 MHz, $CDCl_3$) δ -63.1. The experimental data is in agreement with a previous report.[20]

1-(3-bromophenyl)-4-phenylbutane-1,4-dione (1ab)

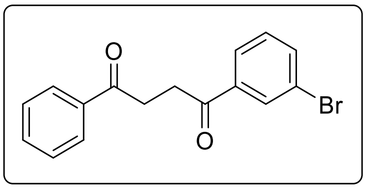

Synthesized from commercial 3-bromobenzaldehyde (**6ab**) and commercial phenyl vinyl ketone (**7b**) according to the general procedure A.

Beige solid (998 mg, 63%); **R*f*** 0.40 (SiO_2; *n*-hexane/EtOAc 4:1); **^{1}H NMR** (400 MHz, $CDCl_3$) δ 8.16 (t, J = 1.8 Hz, 1H), 8.06–8.01 (m, 2H), 7.98–7.94 (m, 1H), 7.70 (ddd, J = 8.0, 2.0, 1.0 Hz, 1H), 7.61–7.55 (m, 1H), 7.52–7.46 (m, 2H), 7.36 (t, J = 7.9 Hz, 1H), 3.49–3.45 (m, 2H), 3.44–3.39 (m, 2H); **^{13}C NMR** (101 MHz, $CDCl_3$) δ 198.5, 197.5, 138.7, 136.8, 136.1, 133.4, 131.4, 130.4, 128.8, 128.3, 126.8, 123.1, 32.8, 32.7. The experimental data is in agreement with a previous report.[21]

1-(2,6-difluorophenyl)-4-phenylbutane-1,4-dione (1ac)

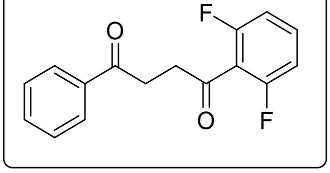

Synthesized from commercial 2,6-difluorobenzaldehyde (**6ac**) and commercial phenyl vinyl ketone (**7b**) according to the general procedure A. After chromatographic purification, the product was additionally recrystallized from *n*-hexane/EtOAc.

Colorless solid (790 mg, 58%); **R*f*** 0.40 (SiO_2; *n*-hexane/EtOAc 4:1); **^{1}H NMR** (400 MHz, $CDCl_3$) δ 8.04–7.98 (m, 2H), 7.60–7.53 (m, 1H), 7.52–7.44 (m, 2H), 7.44–7.34 (m, 1H), 7.02–6.91 (m, 2H), 3.49–3.44 (m, 2H), 3.37–3.32 (m, 2H); **^{13}C NMR** (101 MHz, $CDCl_3$) δ 198.0, 196.3, 161.5 (d, J = 7.3 Hz), 159.0 (d, J = 7.3 Hz), 136.7, 133.3, 132.6 (t, J = 10.5 Hz), 128.7,

128.2, 112.5–112.1 (m), 38.8 (t, J = 2.7 Hz), 32.8; **^{19}F NMR** (376 MHz, $CDCl_3$) δ -111.9; **HRMS** (ESI): m/z $[M+Na]^+$ calcd. for $C_{16}H_{12}F_2NaO_2^+$: 297.0698; found: 297.0695; **FTIR** ṽ (cm^{-1}): 3061, 2914, 1682, 1620, 1595, 1462.

1-phenyl-4-(*o*-tolyl)butane-1,4-dione (1ad)

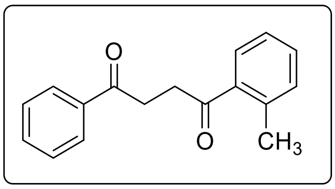

Synthesized from commercial 2-methylbenzaldehyde (**6ad**) and commercial phenyl vinyl ketone (**7b**) according to the general procedure A.

Colorless solid (631 mg, 50%); **R_f** 0.45 (SiO_2; *n*-hexane/EtOAc 4:1); **^{1}H NMR** (400 MHz, $CDCl_3$) δ 8.06–8.01 (m, 2H), 7.82 (dd, J = 7.7, 1.4 Hz, 1H), 7.61–7.53 (m, 1H), 7.52–7.45 (m, 2H), 7.43–7.34 (m, 1H), 7.32–7.24 (m, 2H), 3.48–3.43 (m, 2H), 3.38–3.34 (m, 2H), 2.51 (s, 3H); **^{13}C NMR** (101 MHz, $CDCl_3$) δ 202.8, 198.8, 138.3, 138.0, 136.9, 133.3, 132.0, 131.5, 128.7(×2), 128.3, 125.9, 35.5, 33.0, 21.4. The experimental data is in agreement with a previous report.[21]

7-(4-((tert-butyldimethylsilyl)oxy)phenyl)-1-(4-methoxyphenyl)heptane-1,5-dione (1ag)

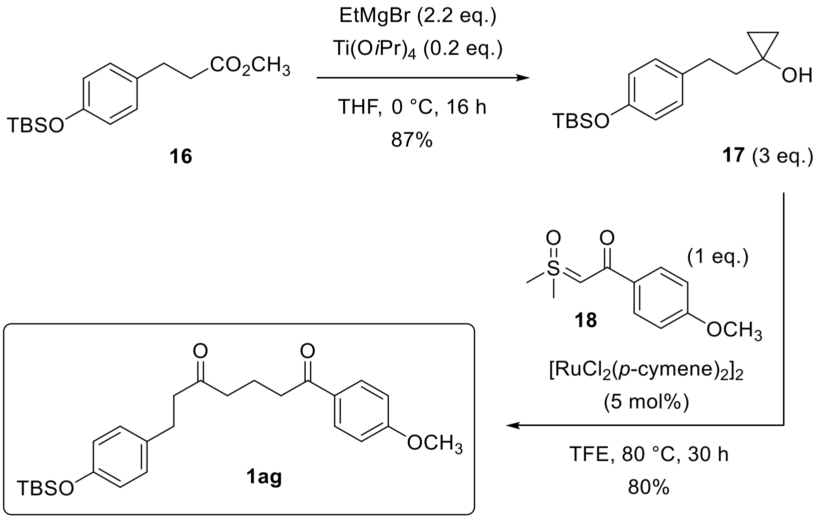

<u>Step 1</u>: In a flame-dried round-bottom flask equipped with a stir bar, under an argon atmosphere, EtMgBr (12.50 mL, 37.36 mmol, 3 M in Et_2O, 2.2 eq.) was added dropwise over 40 min to a solution of 3-[4-(*tert*-butyldimethylsiloxy)phenyl]propanoate[22] **16** (5.00 g, 16.98 mmol, 1.0 eq.) and Ti(O*i*Pr)$_4$ (1.03 mL, 3.40 mmol, 0.2 eq.) in 88 mL of dry THF at 0 °C. The mixture was warmed to room temperature and stirred for 16 h. A saturated aqueous solution of NH_4Cl was slowly added to quench the reaction. The precipitate was removed by filtration. The filtrate was diluted with 200 mL of water, and the aqueous phase was extracted in EtOAc (x3).

The combined organic layers were dried over anhydrous Na_2SO_4, filtered, and the filtrate was concentrated under reduced pressure to obtain a colourless oil as residue. The crude product was purified by silica gel flash column chromatography (eluting with *n*-hexane:EtOAc 100:0 to 90:10) to afford the pure cyclopropanol product **17** (4.30 g, 87%) as a colourless oil. **^{1}H NMR** (400 MHz, $CDCl_3$) δ 7.10–7.03 (m, 2H), 6.79–6.70 (m, 2H), 2.83–2.74 (m, 2H), 1.89–1.79 (m, 2H), 0.98 (s, 9H), 0.79–0.70 (m, 2H), 0.47–0.39 (m, 2H), 0.18 (s, 6H).

Step 2: Experimental procedure adapted from a literature report.[23] In a flame-dried Schlenk tube equipped with a magnetic stir bar, cyclopropanol **17** (1.75 g, 5.97 mmol, 3.0 eq.), sulfoxonium ylide **18**[24] (0.45 g, 1.99 mmol, 1.0 eq.), $[RuCl_2(p\text{-cymene})_2]_2$ (61 mg, 0.10 mmol, 5 mol%) and 40 mL of trifluoroethanol (pre-dried over 4 Å molecular sieves) were added. The mixture was degassed by vacuum-argon cycles (x5) backfilling last with argon. The mixture was heated to 80 °C and stirred for 30 h. The reaction was cooled to room temperature and concentrated under reduced pressure. The residue was purified by silica gel flash column chromatography (eluting with *n*-hexane:EtOAc 100:0 to 70:30) to afford the title product **1ag** (0.70 g, 80%) as a colourless solid. **R_f** 0.15 (SiO_2; *n*-hexane:EtOAc 85:15); **m.p.** = 103–105 °C; **^{1}H NMR** (500 MHz, $CDCl_3$) δ 7.95–7.91 (m, 2H), 7.03–6.98 (m, 2H), 6.95–6.90 (m, 2H), 6.75–6.71 (m, 2H), 3.87 (s, 3H), 2.92 (t, J = 7.1 Hz, 2H), 2.82 (t, J = 7.6 Hz, 2H), 2.71–2.67 (m, 2H), 2.50 (t, J = 7.0 Hz, 2H), 1.98 (p, J = 7.0 Hz, 2H), 0.97 (s, 9H), 0.17 (s, 6H); **^{13}C NMR** (126 MHz, $CDCl_3$) δ 210.2, 198.5, 163.6, 154.0, 133.8, 130.5, 130.1, 129.3, 120.2, 113.9, 55.6, 44.7, 42.2, 37.3, 29.2, 25.8, 18.6, 18.3, -4.3; **HRMS** (ESI): *m/z* $[M+Na]^+$ calcd. for $C_{26}H_{36}NaO_4Si^+$: 463.2275; found: 463.2274. **FTIR** $\tilde{v}$(cm^{-1}): 2929, 2857, 1701, 1668, 1600, 1508, 1248, 914, 778.

1-(quinolin-2-yl)pentane-1,4-dione (1ah)

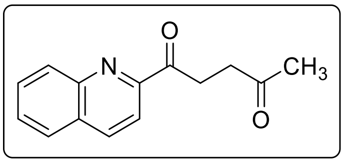

Synthesized from commercial 2-quinolinecarboxaldehyde (**6ah**) and commercial methyl vinyl ketone (**7a**) according to the general procedure A. After chromatographic purification, the product was additionally recrystallized from EtOAc.

Brownish solid (326 mg, 29%); **R_f** 0.45 (SiO_2; *n*-hexane/EtOAc 2:1); **m.p.** = 74–76 °C; **^{1}H NMR** (400 MHz, $CDCl_3$) δ 8.28–8.24 (m, 1H), 8.22–8.18 (m, 1H), 8.09 (d, J = 8.5 Hz, 1H), 7.88–7.85 (m, 1H), 7.80–7.75 (m, 1H), 7.67–7.62 (m, 1H), 3.73–3.68 (m, 2H), 2.97–2.93 (m, 2H), 2.28 (s, 3H); **^{13}C NMR** (101 MHz, $CDCl_3$) δ 207.5, 201.0, 152.9, 147.3, 137.0, 130.7, 130.1, 129.8, 128.7, 127.8, 118.2, 37.5, 31.9, 30.2; **HRMS** (ESI): *m/z* $[M + H]^+$

calcd. for $C_{14}H_{14}NO_2^+$: 228.1019; found: 228.1018. **FTIR** ṽ (cm^{-1}): 3060, 2917, 1716, 1695, 1359, 835.

methyl 2-acetyl-4-oxo-4-phenylbutanoate (1ai)

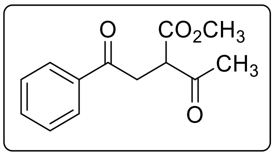

Synthesized according to a reported procedure.[25]

^{1}H NMR (400 MHz, $CDCl_3$) δ 7.98–7.93 (m, 2H), 7.59–7.53 (m, 1H), 7.48–7.42 (m, 2H), 4.22 (dd, *J* = 8.2, 5.6 Hz, 1H), 3.76 (s, 3H), 3.71 (dd, *J* = 18.4, 8.2 Hz, 1H), 3.51 (dd, *J* = 18.4, 5.6 Hz, 1H), 2.42 (s, 3H); **^{13}C NMR** (101 MHz, $CDCl_3$) δ 202.3, 197.1, 169.5, 136.1, 133.6, 128.8, 128.2, 53.7, 52.8, 37.5, 30.4. The experimental data is in agreement with the previous report.[25]

1-(4-methoxyphenyl)-4-phenylbutane-1,4-dione (1aj)

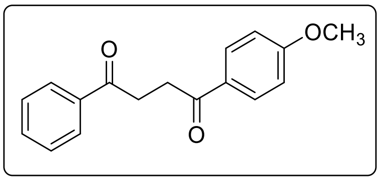

Synthesized from commercial 4-anisaldehyde (**6e**) and commercial phenyl vinyl ketone (**7b**) according to the general procedure A.

Yellowish solid (899 mg, 67%); **R*f*** 0.25 (SiO_2; *n*-hexane/EtOAc 4:1); **^{1}H NMR** (400 MHz, $CDCl_3$) δ 8.08–7.98 (m, 4H), 7.60–7.54 (m, 1H), 7.51–7.43 (m, 2H), 6.98–6.92 (m, 2H), 3.87 (s, 3H), 3.47–3.39 (m, 4H); **^{13}C NMR** (126 MHz, $CDCl_3$) δ 199.0, 197.3, 163.6, 137.0, 133.2, 130.5, 130.0, 128.7, 128.2, 113.8, 55.6, 32.8, 32.3. The experimental data is in agreement with a previous report.[16]

(2*R,3*R**)-2,3-dimethyl-1,4-diphenylbutane-1,4-dione (1ak)**

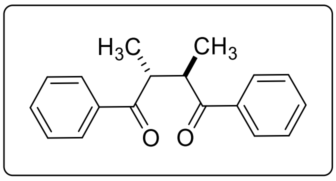

Synthesized following a reported procedure.[26]

^{1}H NMR (400 MHz, $CDCl_3$) δ 8.02–7.97 (m, 4H), 7.58–7.53 (m, 2H), 7.49–7.44 (m, 4H), 4.02–3.91 (m, 2H), 1.32–1.27 (m, 6H); **^{13}C NMR** (101 MHz, $CDCl_3$) δ 204.4, 136.3, 133.1, 128.7, 128.6, 43.8, 15.6. Relative stereochemistry assigned by comparison with a previous report.[27]

1-phenylheptane-1,6-dione (1al)

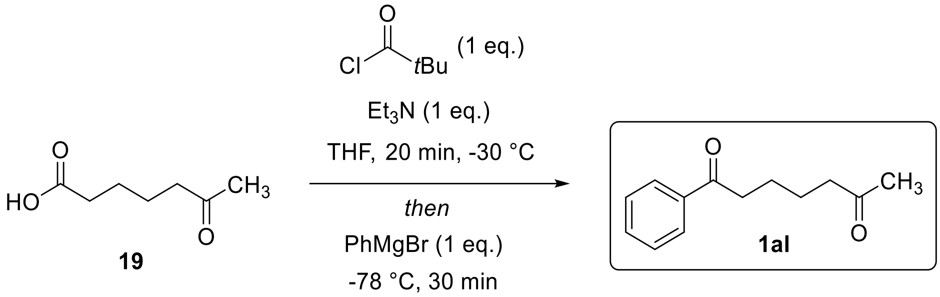

Experimental procedure adapted from a literature report.[28] A flame-dried Schlenk tube, equipped with a magnetic stir bar, was charged with 6-oxoheptanoic acid **19** (1.44 g, 10.0 mmol, 1 eq.) and dry THF (90 ml) under an argon atmosphere. The solution was cooled to -40 °C using a dry ice/acetonitrile cooling bath. Triethylamine (1.39 mL, 10.0 mmol, 1 eq.) and pivaloyl chloride (1.22 mL, 10.0 mmol. 1 eq.) were added in this order and the mixture was stirred for 20 minutes at -40°C. After cooling to -78 °C, phenylmagnesium bromide (3 M in Et_2O, 3.33 mL, 10.0 mmol, 1 eq.) was added dropwise. The reaction mixture was stirred for 30 minutes at -78 °C and quenched with a saturated aqueous solution of ammonium chloride. The aqueous phase was extracted in EtOAc (x2), and the combined organic extracts were washed with brine, dried over Na_2SO_4, filtered, and concentrated under reduced pressure. Purification by silica gel flash column chromatography, eluting with a gradient from *n*-hexane to *n*-hexane:EtOAc 70:30, afforded the product as a colorless solid (1.36 g, 67%). $\mathbf{R}_f$ 0.15 (SiO_2; *n*-hexane/EtOAc 5:1); **^{1}H NMR** (400 MHz, $CDCl_3$) δ 7.97–7.92 (m, 2H), 7.58–7.52 (m, 1H), 7.48–7.43 (m, 2H), 2.98 (t, *J* = 7.0 Hz, 2H), 2.49 (t, *J* = 7.1 Hz, 2H), 2.14 (s, 3H), 1.77–1.63 (m, 4H); **^{13}C NMR** (101 MHz, $CDCl_3$) δ 208.8, 200.1, 137.1, 133.1, 128.7, 128.1, 43.7, 38.4, 30.0, 23.8, 23.6. The experimental data is in agreement with a previous report.[29]

2-(2,5-dioxo-5-phenylpentyl)isoindoline-1,3-dione (1am)

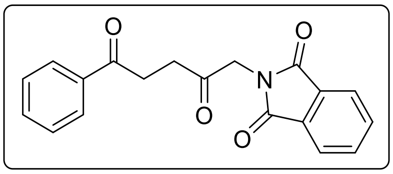

Synthesized from commercial phthalimidoacetaldehyde (**6am**) and commercial phenyl vinyl ketone (**7b**) according to the general procedure A. After chromatographic purification, the product was additionally recrystallized from EtOAc.

Colorless solid (1.19 g, 74%); $\mathbf{R}_f$ 0.55 (SiO_2; *n*-hexane/EtOAc 1:1); **m.p.** = 168–170 °C; **^{1}H NMR** (400 MHz, $CDCl_3$) δ 8.00–7.93 (m, 2H), 7.87 (dd, *J* = 5.5, 3.1 Hz, 2H), 7.76–7.69 (m, 2H), 7.60–7.51 (m, 1H), 7.49–7.40 (m, 2H), 4.65 (s, 2H), 3.36 (t, *J* = 6.4 Hz, 2H), 2.98 (t, *J* = 6.4 Hz, 2H); **^{13}C NMR** (101 MHz, $CDCl_3$) δ 201.5, 197.9, 167.8, 136.5, 134.3, 133.4,

132.2, 128.7, 128.2, 123.7, 46.8, 33.8, 32.5; **HRMS** (ESI): *m/z* $[M+H]^+$ calcd. for $C_{19}H_{16}NO_4^+$: 322.1074; found: 322.1093; **FTIR** ṽ (cm^{-1}): 2921, 1774, 1717, 1676, 1412, 1213.

2-(8-(5-fluoro-2-methoxyphenyl)-3,6-dioxooctyl)isoindoline-1,3-dione (1an)

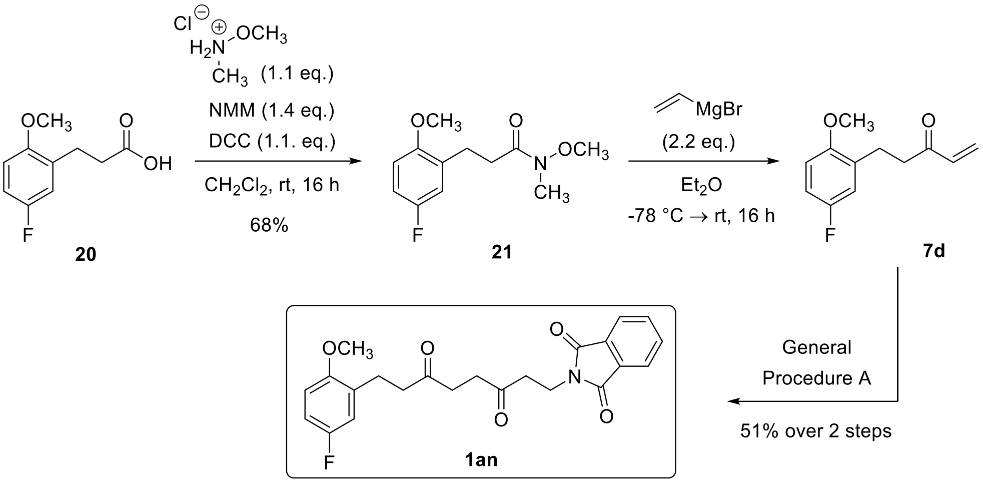

Step 1: A round-bottom flask equipped with a magnetic stir bar was charged with the carboxylic acid **20** (1.50 g, 7.57 mmol, 1.0 eq.) and *N,O*-dimethylhydroxylammmonium chloride (0.81 g, 8.33 mmol, 1.1 eq). An atmosphere of nitrogen was created and dry CH_2Cl_2 (25 mL) followed by *N*-methylmorpholine (NMM, 1.16 mL, 10.60 mmol, 1.4 eq.) were added. The mixture was cooled to 0 °C and DCC (1.72 g, 8.33 mmol, 1.1 eq.) was added in one portion. The reaction was allowed to warm to room temperature and stirred for 16 hours. The reaction was filtered over a pad of celite, eluting with multiple CH_2Cl_2 washes, and the combined organic eluent was washed twice with saturated aqueous $NaHCO_3$. The organic layer was washed with brine, dried over anhydrous Na_2SO_4, filtered, and concentrated under reduced pressure to give the crude product. Purification by silica gel flash column chromatography (eluting with EtOAc/*n*-hexane 1:1) provided the Weinreb amide **21** as a colourless solid (1.25 g, 68%). **^{1}H NMR** (400 MHz, $CDCl_3$) δ 6.93 – 6.81 (m, 2H), 6.75 (dd, *J* = 8.9, 4.5 Hz, 1H), 3.80 (s, 3H), 3.63 (s, 3H), 3.18 (s, 3H), 2.92 (dd, *J* = 8.9, 6.8 Hz, 2H), 2.70 (t, *J* = 8.0 Hz, 2H); **^{19}F NMR** (377 MHz, $CDCl_3$) δ -124.3.

Step 2: A flame-dried Schlenk tube equipped with a magnetic stir bar, under an argon atmosphere, was charged with the Weinreb amide **21** (1.04 g, 4.30 mmol, 1 eq.) and dry Et_2O (22 mL). Vinylmagnesium bromide (1.0 M in THF, 9.46 mL, 9.46 mmol, 2.2 eq.) was added dropwise at -78 °C. The reaction mixture was stirred for 15 min at -78 °C, then for 30 min at 0 °C, then allowed to warm gradually to room temperature. Stirring was maintained for a further 16 h. The reaction mixture was quenched at 0 °C with an aqueous solution of HCl (1 M, 50

mL). The aqueous phase was extracted in EtOAc (x3). The combined organic extracts were washed with brine, dried over Na_2SO_4, filtered, and concentrated under reduced pressure to give the crude vinyl ketone product **7d** as a pale yellow oil. **1H NMR** (400 MHz, $CDCl_3$) δ 6.91 – 6.80 (m, 2H), 6.79 – 6.70 (m, 1H), 6.35 (dd, J = 17.7, 10.5 Hz, 1H), 6.22 (dd, J = 17.7, 1.2 Hz, 1H), 5.83 (dd, J = 10.5, 1.2 Hz, 1H), 3.80 (s, 3H), 2.94 – 2.83 (m, 4H).

Step 3: The crude vinyl ketone **7d** (4.30 mmol theoretical) was reacted with commercial 3-phthalimidopropionaldehyde **6an** (728 mg, 3.6 mmol) according to the general procedure A. After chromatographic purification, the product **1an** was additionally recrystallized from EtOAc. Colorless solid (0.75 g, 51% over the last 2 steps); $\mathbf{R_f}$ 0.55 (SiO_2; *n*-hexane/EtOAc 1:1); **m.p.** = 103–105 °C; **^{1}H NMR** (400 MHz, $CDCl_3$) δ 7.88–7.78 (m, 2H), 7.73–7.67 (m, 2H), 6.87–6.80 (m, 2H), 6.75–6.70 (m, 1H), 3.96 (t, *J* = 7.3 Hz, 2H), 3.78 (s, 3H), 2.92 (t, *J* = 7.3 Hz, 2H), 2.84–2.79 (m, 2H), 2.74–2.67 (m, 6H); **^{13}C NMR** (101 MHz, $CDCl_3$) δ 208.4, 206.7, 168.2, 156.9 (d, *J* = 238.0 Hz), 153.7 (d, *J* = 2.1 Hz), 134.1, 132.2, 131.1 (d, *J* = 7.2 Hz), 123.4, 116.9 (d, *J* = 23.0 Hz), 113.1 (d, *J* = 22.6 Hz), 111.0 (d, *J* = 8.4 Hz), 55.9, 42.3, 40.9, 36.2, 33.1, 24.9, 24.9; **^{19}F NMR** (377 MHz, $CDCl_3$) δ -124.3; **HRMS** (ESI): *m/z* $[M+Na]^+$ calcd. for $C_{23}H_{22}FNNaO_5^+$: 434.1374; found: 434.1373; **FTIR** ṽ (cm^{-1}): 2943, 2908, 1771, 1708, 1496, 1369.

4. Synthesis and characterization of cyclic ether products

General Procedure B: Synthesis of Cyclic Ethers 2

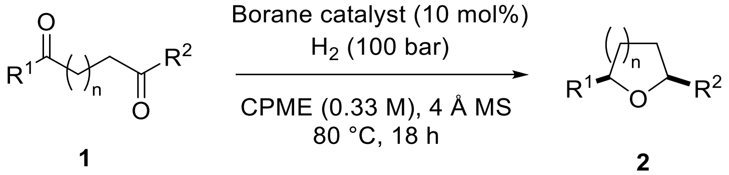

1 **2**

In a flame-dried 4 mL glass vial (screw cap with hole with PTFE/silicone septum) equipped with a magnetic stirring bar, 4 Å molecular sieves (~150 mg/mmol substrate), borane catalyst (0.02 mmol, 10 mol%), substrate **1** (0.2 mmol), and CPME (0.6 mL) were added under an atmosphere of argon. The mixture was stirred for 2 minutes, then the vial was pierced with a needle and placed in an autoclave. The system was sealed and purged three times with nitrogen (6 bar) and four times with hydrogen (3 bar), then pressurized to 100 bar with hydrogen gas. The reaction mixture was heated to 80 °C and stirred for 18 h. After reaction completion, the autoclave was allowed to cool to room temperature and slowly depressurized. The reaction mixture was filtered through a short silica plug, eluting with ethyl acetate. The filtrate was

concentrated under reduced pressure, and the crude product was purified by flash column chromatography on silica gel (gradient elution from *n*-hexane to *n*-hexane/EtOAc 4:1), affording the desired cyclic ether **2**.

Note: Prior to purification, the crude reaction mixture was analyzed by ^{1}H NMR to determine the diastereomeric ratio (dr). In most cases the dr remained unchanged after chromatography, indicating that the *cis/trans* isomers were not separated. When differences were observed, both dr values (before and after purification) are reported below. The NMR data is given for the major *cis*-diastereomer unless otherwise stated.

(2*R**,5*R**)-2-methyl-5-phenyltetrahydrofuran (2a)

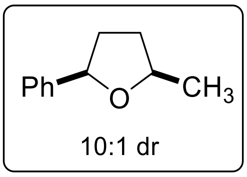

Synthesized from 1,4-diketone **1a** according to the general procedure B (10 mol% of catalyst **B3** was used).

Pale yellow oil (28 mg, 86%, 10:1 dr); **R*f*** 0.40 (SiO_2; *n*-hexane/EtOAc 8:1); **^{1}H NMR** (500 MHz, $CDCl_3$) δ 7.39–7.31 (m, 4H), 7.27–7.23 (m, 1H), 5.05 (dd, *J* = 8.2, 6.4 Hz, 1H, *trans*), 4.89 (t, *J* = 7.3 Hz, 1H), 4.22–4.14 (m, 1H), 2.35–2.26 (m, 1H), 2.14–2.05 (m, 1H), 1.89–1.81 (m, 1H), 1.68–1.56 (m, 1H), 1.38 (d, *J* = 6.1 Hz, 3H); **^{13}C NMR** (126 MHz, $CDCl_3$) δ 143.7, 128.4, 127.3, 126.0, 81.2, 76.1, 34.8, 33.2, 21.5; **HRMS** (ESI): *m/z* $[M+Na]^+$ calcd. for $C_{11}H_{14}NaO^+$: 185.0937; found: 185.0940; **FTIR** ṽ (cm^{-1}): 3026, 2927, 2859, 1450, 1048, 697.

The diastereomeric ratio (dr) was determined by ^{1}H NMR integration of the α-protons adjacent to the oxygen atom, with characteristic signals at 4.89 ppm (*cis*-isomer) and 5.05 ppm (*trans*-isomer).

Assignment of the stereochemistry:

The relative *cis*-configuration of product **2a** was confirmed by 1D NOE NMR spectroscopy through selective irradiation of the protons at the α-positions relative to the oxygen atom (see spectra below). The observed NOE enhancements are consistent with a *cis* relationship between the 2,5-substitutents. Additionally, the NMR data are in agreement with previously reported values for *cis*-**2a**.[30]

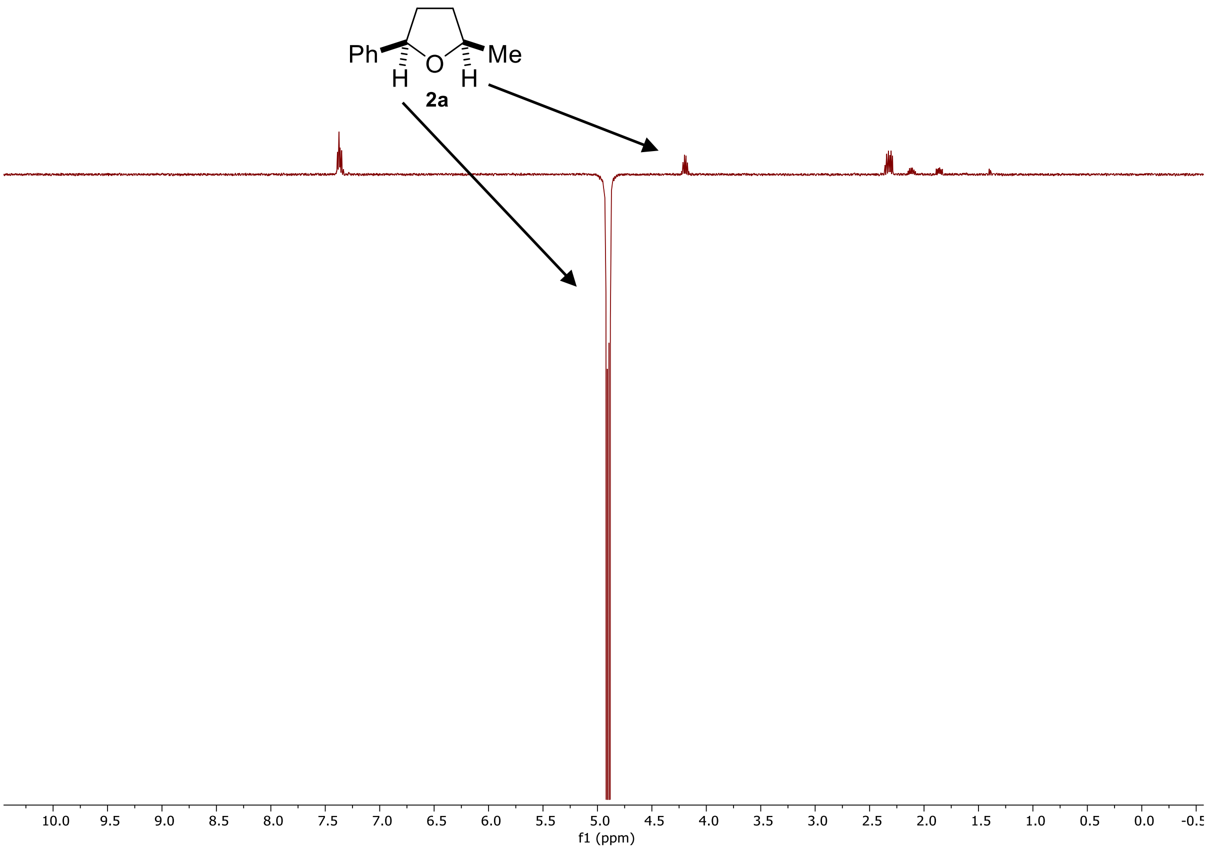
Ph
O
Me
H
H
2a
10.0
9.5
9.0
8.5
8.0
7.5
7.0
6.5
6.0
5.5
5.0
4.5
4.0
3.5
3.0
2.5
2.0
1.5
1.0
0.5
0.0
f1 (ppm)

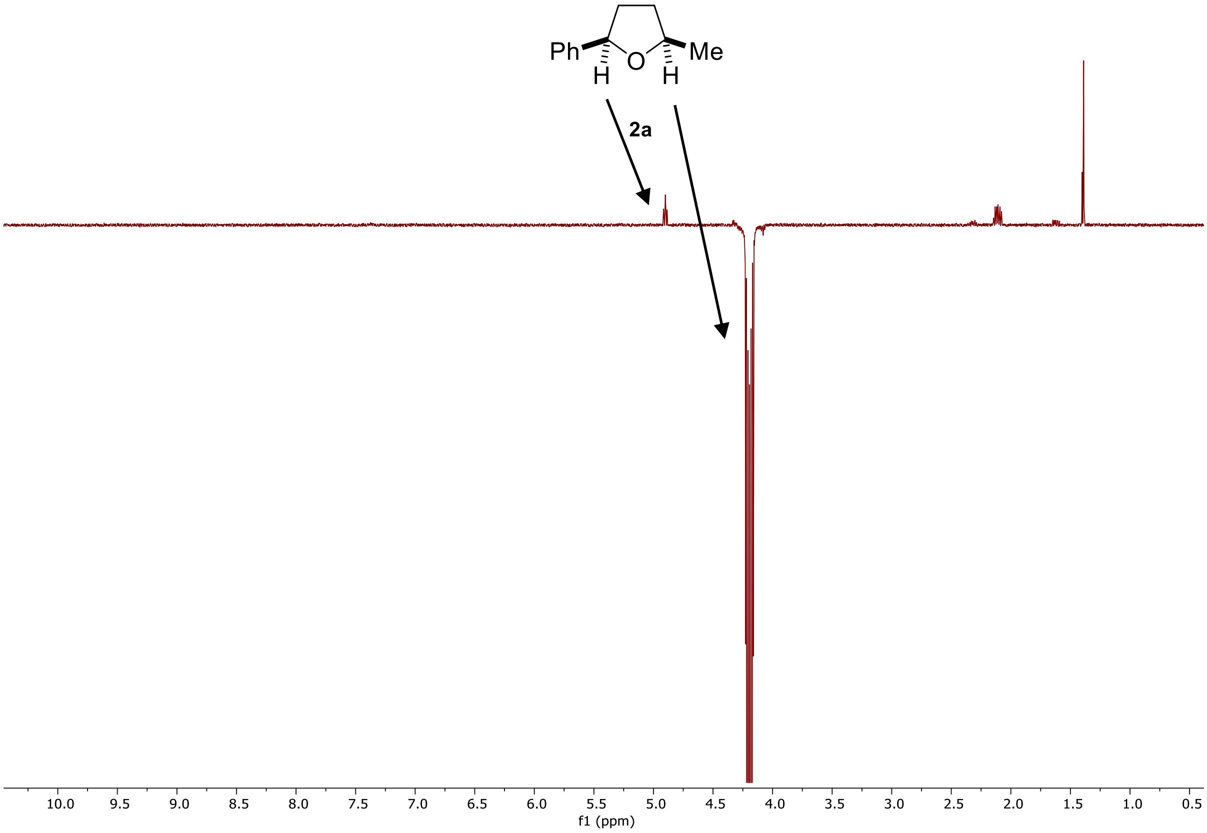
Ph
O
Me
H
H
2a
10.0
9.5
9.0
8.5
8.0
7.5
7.0
6.5
6.0
5.5
5.0
4.5
4.0
3.5
3.0
2.5
2.0
1.5
1.0
0.5
f1 (ppm)

(2*R,5*R**)-2-(4-chlorophenyl)-5-methyltetrahydrofuran (2b)**

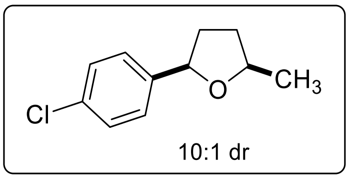

Synthesized from 1,4-diketone **1b** according to the general procedure B (10 mol% of catalyst **B3** was used).

Colorless oil (32 mg, 81%, 10:1 dr); **R*f*** 0.30 (SiO_2; *n*-hexane/EtOAc 12:1); **^{1}H NMR** (500 MHz, $CDCl_3$) δ 7.31–7.27 (m, 4H), 5.00 (dd, *J* = 8.2, 6.5 Hz, 1H, *trans*), 4.84 (t, *J* = 7.3 Hz, 1H), 4.20–4.11 (m, 1H), 2.33–2.25 (m, 1H), 2.13–2.04 (m, 1H), 1.85–1.73 (m, 1H), 1.67–1.53 (m, 1H), 1.36 (d, *J* = 6.1 Hz, 3H); **^{13}C NMR** (126 MHz, $CDCl_3$) δ 142.3, 132.8, 128.5, 127.3, 80.4, 76.2, 34.8, 33.1, 21.4; **HRMS** (ESI): *m/z* $[M + H]^+$ calcd. for $C_{11}H_{14}ClO^+$: 197.0728; found: 197.0725; **FTIR** ṽ (cm^{-1}): 2968, 2867, 1490, 1077, 817.

The diastereomeric ratio (dr) was determined by ^{1}H NMR integration of the α-protons adjacent to the oxygen atom, with characteristic signals at 4.84 ppm (*cis*-isomer) and 5.00 ppm (*trans*-isomer).

methyl 4-((2*R,5*R**)-5-methyltetrahydrofuran-2-yl)benzoate (2c)**

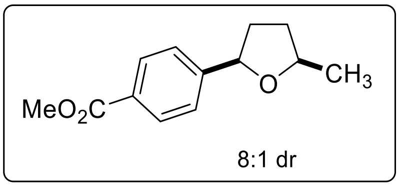

Synthesized from 1,4-diketone **1c** according to the general procedure B (10 mol% of catalyst **B3** was used).

Colorless solid (34 mg, 79%, 8:1 dr); **R*f*** 0.15 (SiO_2; *n*-hexane/EtOAc 12:1); **m.p.** = 32–34 °C; **^{1}H NMR** (500 MHz, $CDCl_3$) δ 8.03–7.96 (m, 2H), 7.44–7.38 (m, 2H), 5.08 (dd, *J* = 8.0, 6.8 Hz, 1H, *trans*), 4.92 (t, *J* = 7.3 Hz, 1H), 4.22–4.14 (m, 1H), 3.89 (s, 3H), 2.38–2.28 (m, 1H), 2.13–2.03 (m, 1H), 1.84–1.75 (m, 1H), 1.62–1.53 (m, 1H), 1.37 (d, *J* = 6.1 Hz, 3H); **^{13}C NMR** (126 MHz, $CDCl_3$) δ 167.1, 149.1, 129.7, 129.0, 125.7, 80.6, 76.4, 52.1, 34.8, 33.1, 21.3; **HRMS** (ESI): *m/z* $[M+Na]^+$ calcd. for $C_{13}H_{16}NaO_3^+$: 243.0992; found: 243.0992; **FTIR** ṽ (cm^{-1}): 2970, 1713, 1438, 1271, 1101, 763.

The diastereomeric ratio (dr) was determined by ^{1}H NMR integration of the α-protons adjacent to the oxygen atom, with characteristic signals at 4.92 ppm (*cis*-isomer) and 5.08 ppm (*trans*-isomer).

(2*R,5*R**)-2-methyl-5-(4-(methylsulfonyl)phenyl)tetrahydrofuran (2d)**

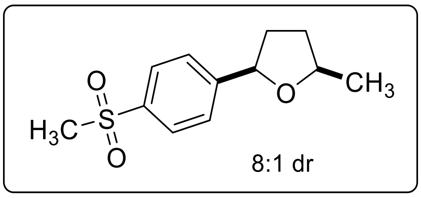

Synthesized from 1,4-diketone **1d** according to the general procedure B (10 mol% of catalyst **B3** was used) in CPME/1,2-DCE (3:1).

Colorless oil (35.1 mg, 73%, 8:1 dr); **R*f*** 0.20 (SiO_2; *n*-hexane/EtOAc 2:1); **^{1}H NMR** (500 MHz, $CDCl_3$) δ 7.91–7.87 (m, 2H), 7.56–7.53 (m, 2H), 5.13–5.08 (m, 1H, *trans*), 4.95 (t, *J* = 7.3 Hz, 1H), 4.25–4.15 (m, 1H), 3.03 (s, 3H), 2.42–2.32 (m, 1H), 2.15–2.07 (m, 1H), 1.84–1.75 (m, 1H), 1.62–1.53 (m, 1H), 1.37 (d, *J* = 6.1 Hz, 3H); **^{13}C NMR** (126 MHz, $CDCl_3$) δ 150.4, 139.2, 127.5, 126.7, 80.1, 76.6, 44.7, 34.8, 33.0, 21.3; **HRMS** (ESI): *m/z* $[M+Na]^+$ calcd. for $C_{12}H_{16}NaO_3S^+$: 263.0712; found: 263.0708; **FTIR** ṽ (cm^{-1}): 2968, 2928, 2870, 1305, 1148, 1086.

The diastereomeric ratio (dr) was determined by ^{1}H NMR integration of the α-protons adjacent to the oxygen atom, with characteristic signals at 4.95 ppm (*cis*-isomer) and 5.10 ppm (*trans*-isomer).

(2*R,5*R**)-2-(4-methoxyphenyl)-5-methyltetrahydrofuran (2e)**

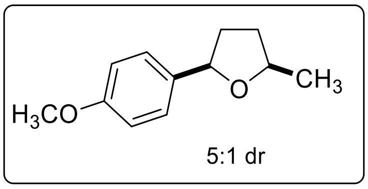

Synthesized from 1,4-diketone **1e** according to the general procedure B (10 mol% of catalyst **B3** was used).

Colorless oil (26 mg, 68%, 5:1 dr); **R*f*** 0.20 (SiO_2; *n*-hexane/EtOAc 12:1); **^{1}H NMR** (500 MHz, $CDCl_3$) δ 7.31–7.25 (m, 2H), 6.89–6.85 (m, 2H), 4.98 (dd, *J* = 8.3, 6.3 Hz, 1H, *trans*), 4.82 (t, *J* = 7.3 Hz, 1H), 4.18–4.10 (m, 1H), 3.80 (s, 3H), 2.29–2.21 (m, 1H), 2.13–2.04 (m, 1H), 1.91–1.77 (m, 1H), 1.67–1.55 (m, 1H), 1.36 (d, *J* = 6.1 Hz, 3H); **^{13}C NMR** (126 MHz, $CDCl_3$) δ 159.0, 135.6, 127.3, 113.8, 80.9, 75.9, 55.4, 34.6, 33.3, 21.5; **HRMS** (ESI): *m/z* $[M+Na]^+$ calcd. for $C_{12}H_{16}NaO_2^+$: 215.1043; found: 215.1042; **FTIR** ṽ (cm^{-1}): 2965, 1511, 1242, 1033, 825.

The diastereomeric ratio (dr) was determined by ^{1}H NMR integration of the α-protons adjacent to the oxygen atom, with characteristic signals at 4.82 ppm (*cis*-isomer) and 4.98 ppm (*trans*-isomer).

4,4,5,5-tetramethyl-2-(3-((2*R,5*R**)-5-methyltetrahydrofuran-2-yl)phenyl)-1,3,2-dioxaborolane (2f)**

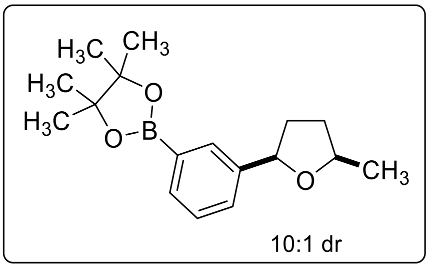

Synthesized from 1,4-diketone **1f** according to the general procedure B (10 mol% of catalyst **B3** was used).
Yellowish oil (36 mg, 85%, 10:1 dr) (9:1 dr in crude reaction mixture); **R*f*** 0.15 (SiO_2; *n*-hexane/EtOAc 12:1); **^{1}H NMR** (500 MHz, $CDCl_3$) δ 7.76–7.73 (m, 1H), 7.70 (dt, *J* = 7.3, 1.3 Hz, 1H), 7.53–7.49 (m, 1H), 7.37–7.32 (m, 1H), 5.04 (dd, *J* = 8.3, 6.4 Hz, 1H, *trans*), 4.88 (t, *J* = 7.4 Hz, 1H), 4.20–4.11 (m, 1H), 2.33–2.25 (m, 1H), 2.12–2.04 (m, 1H), 1.91–1.81 (m, 1H), 1.65–1.56 (m, 1H), 1.38 (d, *J* = 6.1 Hz, 3H), 1.34 (s, 12H); **^{13}C NMR** (126 MHz, $CDCl_3$) δ 142.8, 133.8, 132.4, 128.9, 127.9, 83.9, 81.2, 76.1, 34.7, 33.2, 25.0, 21.4, the signal for the carbon directly attached to boron was not observed due to quadrupolar relaxation; **^{11}B NMR** (160 MHz, $CDCl_3$) δ 31.0. **HRMS** (ESI): *m/z* $[M+Na]^+$ calcd. for $C_{17}H_{25}BNaO_3^+$: 311.1789; found: 311.1785. **FTIR** ṽ (cm^{-1}): 2972, 1355, 1141, 964, 708.

The diastereomeric ratio (dr) was determined by ^{1}H NMR integration of the α-protons adjacent to the oxygen atom, with characteristic signals at 4.88 ppm (*cis*-isomer) and 5.04 ppm (*trans*-isomer).

4-((2*R,5*R**)-5-methyltetrahydrofuran-2-yl)benzonitrile (2g)**

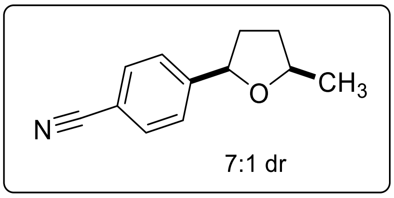

Synthesized from 1,4-diketone **1g** according to the general procedure B (20 mol% of catalyst **B3** was used).
Colorless oil (15.7 mg, 42%, 7:1 dr); **R*f*** 0.35 (SiO_2; *n*-hexane/EtOAc 4:1); **^{1}H NMR** (500 MHz, $CDCl_3$) δ 7.63–7.60 (m, 2H), 7.45 (d, *J* = 8.0 Hz, 2H), 5.09–5.05 (m, 1H, *trans*), 4.95–4.89 (m, 1H), 4.25–4.15 (m, 1H), 2.40– 2.30 (m, 1H), 2.14–2.06 (m, 1H), 1.82–1.74 (m, 1H), 1.62–1.54 (m, 2H), 1.37 (d, *J* = 6.1 Hz, 3H); **^{13}C NMR** (126 MHz, $CDCl_3$) δ 149.4, 132.3, 126.5, 119.2, 110.9, 80.2, 76.6, 34.8, 33.0, 21.3; **HRMS** (ESI): *m/z* $[M+Na]^+$ calcd. for $C_{12}H_{13}NNaO^+$: 210.0889; found: 210.0888; **FTIR** ṽ (cm^{-1}): 2970, 2870, 2227, 1609, 1080, 844.

The diastereomeric ratio (dr) was determined by ^{1}H NMR integration of the α-protons adjacent to the oxygen atom, with characteristic signals at 4.92 ppm (*cis*-isomer) and 5.07 ppm (*trans*-isomer).

***N*-(4-((2*R**,5*R**)-5-methyltetrahydrofuran-2-yl)phenyl)acetamide (2h)**

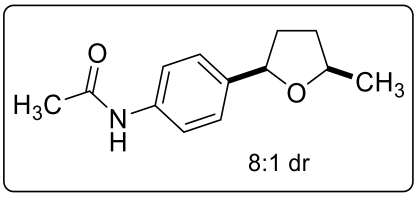

Synthesized from 1,4-diketone **1h** according to the general procedure B (20 mol% of catalyst **B3** was used) in CPME/1,2-DCE (3:1).

Colorless oil (23.1 mg, 53%, 8:1 dr); **R*f*** 0.15 (SiO_2; *n*-hexane/EtOAc 1:1); **^{1}H NMR** (500 MHz, $CDCl_3$) δ 7.50 (br. s, 1H), 7.43 (d, *J* = 8.5 Hz, 2H), 7.28 (d, *J* = 8.4 Hz, 2H), 5.01–4.97 (m, 1H, *trans*), 4.86–4.79 (m, 1H), 4.20–4.10 (m, 1H), 2.30–2.22 (m, 1H), 2.13 (s, 3H), 2.11–2.03 (m, 1H), 1.83–1.77 (m, 1H), 1.62–1.54 (m, 1H), 1.35 (d, *J* = 6.1 Hz, 3H); **^{13}C NMR** (126 MHz, $CDCl_3$) δ 168.6, 139.5, 137.0, 126.6, 120.0, 80.8, 76.1, 34.7, 33.2, 24.6, 21.5; **HRMS** (ESI): *m/z* $[M+Na]^+$ calcd. for $C_{13}H_{17}NNaO_2^+$: 242.1151; found: 242.1148; **FTIR** ṽ (cm^{-1}): 3297, 2967, 1666, 1602, 1537, 1316.

The diastereomeric ratio (dr) was determined by ^{1}H NMR integration of the α-protons adjacent to the oxygen atom, with characteristic signals at 4.83 ppm (*cis*-isomer) and 4.99 ppm (*trans*-isomer).

(4-((2*R,5*R**)-5-methyltetrahydrofuran-2-yl)phenyl)methanol (2i)**

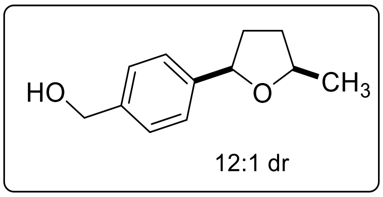

Synthesized from 1,4-diketone **1i** according to the general procedure B (20 mol% of catalyst **B3** was used).

Yellowish oil (18.1 mg, 47%, 12:1 dr); **R*f*** 0.30 (SiO_2; *n*-hexane/EtOAc 2:1); **^{1}H NMR** (500 MHz, $CDCl_3$) δ 7.36–7.28 (m, 4H), 5.05–5.02 (m, 1H, *trans*), 4.91–4.84 (m, 1H), 4.65 (s, 2H), 4.21–4.12 (m, 1H), 2.35–2.24 (m, 1H), 2.15–2.02 (m, 1H), 1.91–1.75 (m, 2H), 1.64–1.55 (m, 1H), 1.37 (d, *J* = 6.1 Hz, 3H); **^{13}C NMR** (126 MHz, $CDCl_3$) δ 143.1, 139.9, 127.1, 126.2, 80.9, 76.1, 65.3, 34.8, 33.2, 21.5; **HRMS** (ESI): *m/z* $[M+Na]^+$ calcd. for $C_{12}H_{16}NaO_2^+$: 215.1043; found: 215.1041; **FTIR** ṽ (cm^{-1}): 3360, 2967, 2866, 1418, 1072, 1013.

The diastereomeric ratio (dr) was determined by ^{1}H NMR integration of the α-protons adjacent to the oxygen atom, with characteristic signals at 4.87 ppm (*cis*-isomer) and 5.04 ppm (*trans*-isomer).

(2*R,5*R**)-2-methyl-5-(naphthalen-2-yl)tetrahydrofuran (2j)**

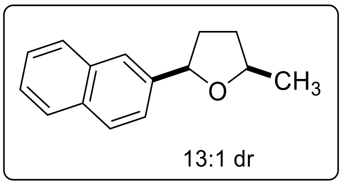

Synthesized from 1,4-diketone **1j** according to the general procedure B (10 mol% of catalyst **B3** was used).

Colorless oil (36 mg, 88%, 13:1 dr); **R*f*** 0.25 (SiO_2; *n*-hexane/EtOAc 12:1); **^{1}H NMR** (500 MHz, $CDCl_3$) δ 7.86–7.78 (m, 4H), 7.50–7.41 (m, 3H), 5.22 (dd, *J* = 8.1, 6.5 Hz, 1H, *trans*), 5.06 (t, *J* = 7.3 Hz, 1H), 4.28–4.19 (m, 1H), 2.42–2.33 (m, 1H), 2.18 – 2.09 (m, 1H), 1.99–1.87 (m, 1H), 1.72–1.60 (m, 1H), 1.43 (d, *J* = 6.1 Hz, 3H); **^{13}C NMR** (126 MHz, $CDCl_3$) δ 141.1, 133.4, 133.0, 128.2, 128.1, 127.8, 126.1, 125.7, 124.4, 124.3, 81.3, 76.3, 34.8, 33.3, 21.5; **HRMS** (ESI): *m/z* $[M+Na]^+$ calcd. for $C_{15}H_{16}NaO^+$: 235.1093; found: 235.1095; **FTIR** ṽ (cm^{-1}): 2966, 2865, 1372, 1078, 815.

The diastereomeric ratio (dr) was determined by ^{1}H NMR integration of the α-protons adjacent to the oxygen atom, with characteristic signals at 5.06 ppm (*cis*-isomer) and 5.22 ppm (*trans*-isomer).

(2*R,5*R**)-2-methyl-5-(thiophen-3-yl)tetrahydrofuran (2k)**

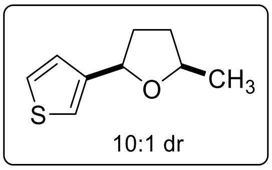

Synthesized from 1,4-diketone **1k** according to the general procedure B (10 mol% of catalyst **B3** was used).

Yellowish oil (25 mg, 73%, 10:1 dr) (8:1 dr in crude reaction mixture); **R*f*** 0.30 (SiO_2; *n*-hexane/EtOAc 12:1); **^{1}H NMR** (500 MHz, $CDCl_3$) δ 7.28 (dd, *J* = 5.0, 3.0 Hz, 1H), 7.21–7.17 (m, 1H), 7.05 (dd, *J* = 5.0, 1.3 Hz, 1H), 5.10 (t, *J* = 7.1 Hz, 1H, *trans*), 4.94 (t, *J* = 7.1 Hz, 1H), 4.16–4.08 (m, 1H), 2.30–2.21 (m, 1H), 2.13–2.03 (m, 1H), 1.97–1.87 (m, 1H), 1.65–1.55 (m, 1H), 1.34 (d, *J* = 6.1 Hz, 3H); **^{13}C NMR** (126 MHz, $CDCl_3$) δ 144.9, 126.01, 125.97, 120.8, 77.5, 76.0, 33.7, 33.2, 21.5; **HRMS** (ESI): *m/z* $[M+Na]^+$ calcd. for $C_9H_{12}NaOS^+$: 191.0501; found: 191.0503; **FTIR** ṽ (cm^{-1}): 2967, 2865, 1378, 1075, 779.

The diastereomeric ratio (dr) was determined by ^{1}H NMR integration of the α-protons adjacent to the oxygen atom, with characteristic signals at 4.94 ppm (*cis*-isomer) and 5.10 ppm (*trans*-isomer).

3-((2*R,5*R**)-5-methyltetrahydrofuran-2-yl)-1-tosyl-1*H*-indole (2l)**

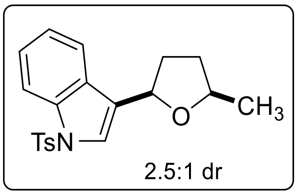

Synthesized from 1,4-diketone **1l** according to the general procedure B (10 mol% of catalyst **B3** was used). The product was purified by flash column chromatography on silica gel (gradient elution from *n*-hexane:CH_2Cl_2 80:20 to 0:100).

Purple oil (44 mg, 62%, 2.5:1 dr); **R*f*** 0.15 (SiO_2; *n*-hexane/EtOAc 8:1); **^{1}H NMR** (400 MHz, $CDCl_3$) δ 7.99–7.94 (m, 1H, *cis+trans*), 7.79–7.73 (m, 2H, *cis+trans*), 7.58–7.53 (m, 1H, *cis+trans*), 7.53–7.48 (m, 1H, *cis+trans*), 7.33–7.27 (m, 1H, *cis+trans*), 7.24–7.16 (m, 3H, *cis+trans*), 5.25 (ddd, *J* = 7.7, 6.5, 1.1 Hz, 1H, *trans*), 5.07 (td, *J* = 7.2, 1.1 Hz, 1H, *cis*), 4.33 (dp, *J* = 8.0, 6.0 Hz, 1H, *trans*), 4.15 (dp, *J* = 8.1, 6.1 Hz, 1H, *cis*), 2.44 – 2.24 (m, 4H, *cis+trans*), 2.23 – 1.94 (m, 2H, *cis+trans*), 1.71 – 1.55 (m, 1H, *cis+trans*), 1.38 (d, *J* = 6.1 Hz, 3H, *cis*), 1.32 (d, *J* = 6.1 Hz, 3H, *trans*); **^{13}C NMR** (101 MHz, $CDCl_3$) δ 145.0 (*cis+trans*), 135.8 (*trans*), 135.8 (*cis*), 135.4 (*cis+trans*), 130.0 (*cis+trans*), 129.2 (*trans*), 129.1 (*cis*), 126.99 (*cis*), 126.97 (*trans*), 125.1 (*trans*), 124.9 (*cis*), 124.8 (*cis+trans*), 123.2 (*cis+trans*), 122.8 (*cis*), 122.5 (*trans*), 120.4 (*trans*), 120.35 (*cis*), 113.9 (*cis+trans*), 76.1 (*cis*), 76.0 (*trans*), 74.8 (*cis*), 74.2 (*trans*), 34.1 (*trans*), 33.1 (*cis*), 33.0 (*trans*), 32.4 (*cis*), 21.7 (*cis+trans*), 21.5 (*trans*), 21.4 (*cis*). **HRMS** (ESI): *m/z* $[M+Na]^+$ calcd. for $C_{20}H_{21}NNaO_3S^+$: 378.1134; found: 378.1125. **FTIR** ṽ(cm^{-1}): 2968, 1719, 1445, 1364, 1169, 745.

The diastereomeric ratio (dr) was determined by ^{1}H NMR integration of the α-protons adjacent to the oxygen atom, with characteristic signals at 5.07 ppm (*cis*-isomer) and 5.25 ppm (*trans*-isomer).

(2*R,5*R**)-2-butyl-5-phenyltetrahydrofuran (2m)**

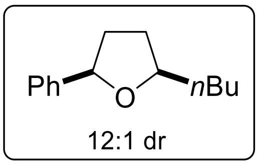

Synthesized from 1,4-diketone **1m** according to the general procedure B (20 mol% of catalyst **B3** was used).*

Yellowish oil (35 mg, 86%, 12:1 dr); **R*f*** 0.45 (SiO_2; *n*-hexane/EtOAc 12:1); **^{1}H NMR** (500 MHz, $CDCl_3$) δ 7.39–7.29 (m, 4H), 7.29–7.21 (m, 1H), 5.00 (dd, *J* = 8.3, 6.4 Hz, 1H, *trans*), 4.88 (t, *J* = 7.3 Hz, 1H), 4.07–3.98 (m, 1H), 2.34–2.24 (m, 1H), 2.11–2.03 (m, 1H), 1.85–1.73 (m, 2H), 1.68–1.56 (m, 2H), 1.51–1.36 (m, 4H), 0.96–0.92 (m, 3H); **^{13}C NMR** (126 MHz, $CDCl_3$) δ 143.8, 128.3, 127.2, 126.0, 80.9, 80.2, 35.9, 34.6, 31.5, 28.6, 23.0, 14.2; **HRMS** (ESI):

m/z $[M+Na]^+$ calcd. for $C_{14}H_{20}NaO^+$: 227.1406; found: 227.1405; **FTIR** ṽ (cm^{-1}): 2928, 2858, 1451, 1051, 697.
The diastereomeric ratio (dr) was determined by ^1H NMR integration of the α-protons adjacent to the oxygen atom, with characteristic signals at 4.88 ppm (*cis*-isomer) and 5.00 ppm (*trans*-isomer).
*Note: When the reaction was performed using 10 mol% of catalyst **B1**, the product was obtained in 89% yield and 7:1 dr.

(2*S**,5*R**)-2-benzyl-5-phenyltetrahydrofuran (2n)

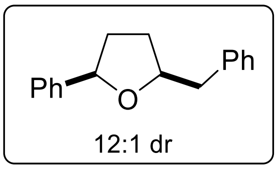

Synthesized from 1,4-diketone **1n** according to the general procedure B (20 mol% of catalyst **B3** was used).*
Yellowish oil (33 mg, 69%, 12:1 dr); **R*f*** 0.45 (SiO_2; *n*-hexane/EtOAc 12:1); **^{1}H NMR** (500 MHz, $CDCl_3$) δ 7.38–7.22 (m, 10H), 5.04 (dd, *J* = 7.9, 6.3 Hz, 1H, *trans*), 4.92 (t, *J* = 7.1 Hz, 1H), 4.32–4.24 (m, 1H), 3.14 (dd, *J* = 13.5, 6.0 Hz, 1H), 2.88 (dd, *J* = 13.5, 7.0 Hz, 1H), 2.34–2.25 (m, 1H), 2.04–1.98 (m, 1H), 1.86–1.73 (m, 2H); **^{13}C NMR** (126 MHz, $CDCl_3$) δ 143.5, 138.9, 129.5, 128.5, 128.4, 127.3, 126.4, 125.9, 81.1, 80.8, 42.4, 34.4, 30.9; **HRMS** (ESI): *m/z* $[M+Na]^+$ calcd. for $C_{17}H_{18}NaO^+$: 261.1250; found: 261.1255. **FTIR** ṽ (cm^{-1}): 2927, 2859, 1449, 1048, 696.
The diastereomeric ratio (dr) was determined by ^1H NMR integration of the α-protons adjacent to the oxygen atom, with characteristic signals at 4.92 ppm (*cis*-isomer) and 5.04 ppm (*trans*-isomer).
*Note: When the reaction was performed using 10 mol% of catalyst **B1**, the product was obtained in 88% yield and 7:1 dr.

(2*S**,5*R**)-2-((benzyloxy)methyl)-5-phenyltetrahydrofuran (2o)

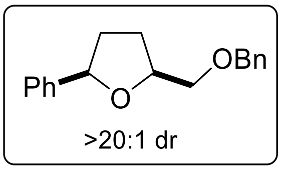

Synthesized from 1,4-diketone **1o** according to the general procedure B (20 mol% of catalyst **B3** was used).
Yellowish oil (45.5 mg, 85%, >20:1 dr) (16:1 dr in crude reaction mixture); **R*f*** 0.25 (SiO_2; *n*-hexane/EtOAc 8:1); **^{1}H NMR** (500 MHz, $CDCl_3$) δ 7.42–7.24 (m, 10H), 5.08–5.04 (m, 1H, *trans*), 4.98–4.91 (m, 1H), 4.65 (s, 2H), 4.36–4.27 (m, 1H), 3.70–3.60 (m, 2H), 2.35–2.25 (m, 1H), 2.13–2.05 (m, 1H), 1.93–1.81 (m, 2H); **^{13}C NMR** (126 MHz, $CDCl_3$) δ 143.1, 138.5, 128.5, 128.3, 127.8,

127.7, 127.3, 126.0, 81.5, 78.7, 73.5, 73.0, 34.4, 28.6; **HRMS** (ESI): *m/z* [M+Na]$^+$ calcd. for $C_{18}H_{20}NaO_2^+$: 291.1356; found: 291.1349; **FTIR** ṽ (cm^{-1}): 2858, 1451, 1082, 1059, 695.

The diastereomeric ratio (dr) was determined by ^{1}H NMR integration of the α-protons adjacent to the oxygen atom, with characteristic signals at 4.98–4.91 ppm (*cis*-isomer) and 5.08–5.04 ppm (*trans*-isomer).

(2*S,5*R**)-2-(2,6-dimethylhept-5-en-1-yl)-5-phenyltetrahydrofuran (2p)**

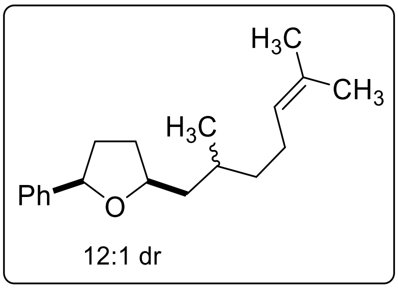

Synthesized from 1,4-diketone **1p** according to the general procedure B (20 mol% of catalyst **B3** was used).*

Colorless oil (28 mg, 51%, 12:1 dr); **R*f*** 0.50 (SiO_2; *n*-hexane/EtOAc 12:1); **^{1}H NMR** (500 MHz, $CDCl_3$) δ 7.37–7.31 (m, 4H), 7.27–7.22 (m, 1H), 5.13 (m, 1H), 5.04–4.98 (m, 1H, *trans*), 4.88 (t, *J* = 7.3 Hz, 1H), 4.16–4.08 (m, 1H), 2.34–2.25 (m, 1H), 2.11–1.96 (m, 3H), 1.85–1.73 (m, 2H), 1.72–1.69 (m, 3H), 1.68–1.55 (m, 6H), 1.46–1.33 (m, 1H), 1.29–1.17 (m, 1H), 1.00–0.96 (m, 3H); **^{13}C NMR** (126 MHz, $CDCl_3$) δ 143.9, 143.8, 131.25, 131.24, 128.35, 128.33, 127.18, 127.16, 125.95, 125.93, 125.04, 125.01, 80.85, 80.77, 78.7, 78.1, 43.7, 43.4, 37.9, 37.5, 34.7, 34.5, 32.2, 31.7, 30.4, 30.0, 25.88, 25.87, 25.60, 25.58, 20.2, 19.8, 17.8 (one carbon single is missing); **HRMS** (ESI): *m/z* [M+Na]$^+$ calcd. for $C_{19}H_{28}NaO^+$: 295.2032; found: 295.2031; **FTIR** ṽ (cm^{-1}): 2914, 2868, 1450, 1051, 697.

The diastereomeric ratio (dr) was determined by ^{1}H NMR integration of the α-protons adjacent to the oxygen atom, with characteristic signals at 4.88 ppm (*cis*-isomer) and 5.04–4.98 ppm (*trans*-isomer).

*Note: When the reaction was performed using 10 mol% of catalyst **B1**, the product was obtained in 89% yield and 7:1 dr.

(2*R,3a*R**,7a*R**)-2-phenyloctahydrobenzofuran (2q)**

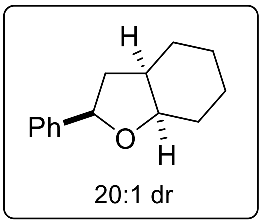

Synthesized from 1,4-diketone **1q** according to the general procedure B (10 mol% of catalyst **B3** was used).

Yellowish oil (26 mg, 63%, 20:1 dr); **R*f*** 0.35 (SiO_2; *n*-hexane/EtOAc 12:1); **^{1}H NMR** (500 MHz, $CDCl_3$) δ 7.42–7.39 (m, 2H), 7.36–7.31 (m, 2H), 7.26–7.21 (m, 1H), 5.18 (dd, *J* = 8.5, 6.9

Hz, 1H, *trans*), 4.97 (dd, J = 8.7, 7.0 Hz, 1H), 4.03 (q, J = 4.8 Hz, 1H), 2.49–2.39 (m, 1H), 2.27–2.15 (m, 1H), 2.01–1.92 (m, 1H), 1.79–1.71 (m, 1H), 1.70–1.61 (m, 2H), 1.60–1.50 (m, 2H), 1.47–1.38 (m, 2H), 1.34–1.22 (m, 1H); **^{13}C NMR** (126 MHz, $CDCl_3$) δ 144.6, 128.4, 126.9, 125.7, 79.6, 78.1, 40.7, 38.3, 29.1, 28.6, 23.9, 21.7; **HRMS** (ESI): *m/z* $[M+Na]^+$ calcd. for $C_{14}H_{18}NaO^+$: 225.1250; found: 225.1247; **FTIR** ṽ (cm^{-1}): 2927, 2851, 1447, 1024, 697.

Stereochemistry assigned by comparison with a previous report.[31] The diastereomeric ratio (dr) was determined by ^{1}H NMR integration of the α-protons adjacent to the oxygen atom, with characteristic signals at 4.97 ppm (*cis*-isomer) and 5.18 ppm (*trans*-isomer). All minor diastereomers are combined in a single number.

1-((2*R**,5*R**)-5-methyltetrahydrofuran-2-yl)-4-((2*S**,5*S**)-5-methyltetrahydrofuran-2-yl)benzene (2r) and 1,4-bis((2*S**,5*S**)-5-methyltetrahydrofuran-2-yl)benzene (2r′)

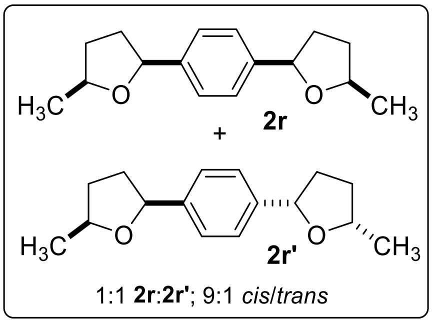

Synthesized from bis-1,4-diketone **1r** according to the general procedure B (20 mol% of catalyst **B3** was used). Colorless oil (30 mg, 61%, 1:1 **2r**:**2r′**, 9:1 *cis*/*trans*); **R*f*** 0.20 (SiO_2; *n*-hexane/EtOAc 8:1); **^{1}H NMR** (500 MHz, $CDCl_3$) δ 7.33–7.27 (m, 4H), 5.05–5.01 (m, 2H, *trans*), 4.89–4.84 (m, 2H), 4.19–4.12 (m, 2H), 2.32–2.23 (m, 2H), 2.12–2.03 (m, 2H), 1.87–1.78 (m, 2H), 1.63–1.55 (m, 2H), 1.36 (dd, J = 6.1, 1.0 Hz, 6H); **^{13}C NMR** (126 MHz, $CDCl_3$) δ 142.53, 142.51, 125.93, 125.90, 81.02, 81.00, 76.06, 76.05, 34.73, 34.67, 33.22, 33.21, 21.5 (x2); **HRMS** (ESI): *m/z* $[M+Na]^+$ calcd. for $C_{16}H_{22}NaO_2^+$: 269.1512; found: 269.1510; **FTIR** ṽ (cm^{-1}): 2969, 2868, 1383, 1074, 1021.

The total *cis/trans* ratio was determined by ^{1}H NMR integration of the α-protons adjacent to the oxygen atom, with characteristic signals at 4.89–4.84 ppm (*cis*-isomer) and 5.05–5.01 ppm (*trans*-isomer).

(2*R,5*S**)-2-ethyl-5-hexyltetrahydrofuran (2s)**

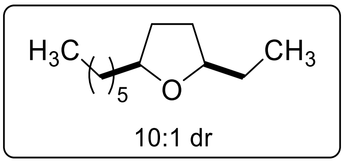

Synthesized from 1,4-diketone **1s** according to the general procedure B (20 mol% of catalyst **B3** was used).*

Colorless oil (29 mg, 79%, 10:1 dr) (8:1 dr in crude reaction mixture); **R_f** 0.30 (SiO_2; *n*-hexane/EtOAc 20:1); **^{1}H NMR** (500 MHz, $CDCl_3$) δ 3.93–3.82 (m, 2H, *trans*), 3.82–3.66 (m, 2H), 1.95–1.85 (m, 2H), 1.66–1.56 (m, 2H), 1.49–1.24 (m, 12H), 0.91 (t, J = 7.5 Hz, 3H), 0.87 (t, J = 7.0 Hz, 3H); **^{13}C NMR** (126 MHz, $CDCl_3$) δ 80.7, 79.6, 36.3, 32.0, 31.2, 30.7, 29.6, 29.0, 26.4, 22.8, 14.2, 10.5; **HRMS** (ESI): *m/z* $[M+Na]^+$ calcd. for $C_{12}H_{24}NaO^+$: 207.1719; found: 207.1718. **FTIR** ṽ (cm^{-1}): 2924, 2855, 1461, 1089, 1037.

The diastereomeric ratio (dr) was determined by ^{1}H NMR integration of the α-protons adjacent to the oxygen atom, with characteristic signals at 3.82–3.66 ppm (*cis*-isomer) and 3.93–3.82 ppm (*trans*-isomer).

*Note: When the reaction was performed using 10 mol% of catalyst **B1**, the product was obtained in 85% yield and 6:1 dr.

(2*R,5*R**)-2-benzyl-5-methyltetrahydrofuran (2t)**

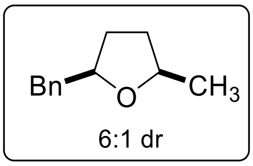

Synthesized from 1,4-diketone **1t** according to the general procedure B (10 mol% of catalyst **B3** was used).

Yellowish oil (27 mg, 78%, 6:1 dr); **R_f** 0.30 (SiO_2; *n*-hexane/EtOAc 12:1); **^{1}H NMR** (500 MHz, $CDCl_3$) δ 7.31–7.27 (m, 2H), 7.25–7.19 (m, 3H), 4.28–4.23 (m, 1H, *trans*), 4.11–4.04 (m, 1H), 4.02–3.94 (m, 1H), 2.98 (dd, J = 13.5, 5.8 Hz, 1H), 2.73 (dd, J = 13.6, 7.1 Hz, 1H), 1.97–1.83 (m, 2H), 1.67–1.58 (m, 1H), 1.49–1.36 (m, 1H), 1.25 (d, J = 6.1 Hz, 3H). **^{13}C NMR** (126 MHz, $CDCl_3$) δ 139.0, 129.5, 128.4, 126.3, 80.2, 75.6, 42.7, 32.8, 30.9, 21.6. **HRMS** (ESI): *m/z* $[M+H]^+$ calcd. for $C_{12}H_{17}O^+$: 177.1274; found: 177.1270. **FTIR** ṽ (cm^{-1}): 2966, 2863, 1452, 1083, 698.

The diastereomeric ratio (dr) was determined by ^{1}H NMR integration of the α-protons adjacent to the oxygen atom, with characteristic signals at 4.11–4.04 ppm (*cis*-isomer) and 4.28–4.23 ppm (*trans*-isomer).

(2*R,6*R**)-2-methyl-6-phenyltetrahydro-2*H*-pyran (2u)**

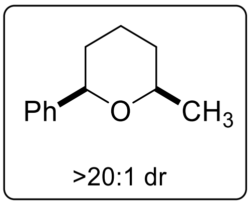

Synthesized from 1,5-diketone **1u** according to the general procedure B (10 mol% of catalyst **B3** was used).

Colorless oil (31 mg, 88%, >20:1 dr); **R*f*** 0.35 (SiO_2; *n*-hexane/EtOAc 20:1); **^{1}H NMR** (500 MHz, $CDCl_3$) δ 7.40–7.37 (m, 2H), 7.37–7.32 (m, 2H), 7.28–7.24 (m, 1H), 4.39 (dd, *J* = 11.3, 2.3 Hz, 1H), 3.65 (dqd, *J* = 12.4, 6.2, 2.0 Hz, 1H), 1.97–1.89 (m, 1H), 1.85–1.79 (m, 1H), 1.75–1.63 (m, 2H), 1.58–1.47 (m, 1H), 1.38–1.29 (m, 1H), 1.28 (d, *J* = 6.2 Hz, 3H); **^{13}C NMR** (126 MHz, $CDCl_3$) δ 143.7, 128.4, 127.3, 126.1, 80.0, 74.5, 33.7, 33.2, 24.3, 22.5; **HRMS** (ESI): *m/z* $[M+Na]^+$ calcd. for $C_{12}H_{16}NaO^+$: 199.1093; found: 199.1091; **FTIR** ṽ (cm^{-1}): 3029, 2931, 2847, 1451, 1086.

The experimental data is in agreement with the reported for the *cis*-isomer.[31] The *trans*-isomer was not detected by NMR.

(2*S,5*R**)-2-cyclohexyl-5-phenyltetrahydrofuran (2v)**

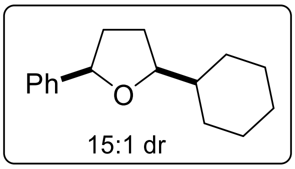

Synthesized from 1,4-diketone **1v** according to the general procedure B (10 mol% of catalyst **B1** was used).

Colorless oil (40 mg, 86%, 15:1 dr); **R*f*** 0.55 (SiO_2; *n*-hexane/EtOAc 12:1); **^{1}H NMR** (500 MHz, $CDCl_3$) δ 7.39–7.31 (m, 4H), 7.23–7.27 (m, 1H), 4.96 (dd, *J* = 8.4, 6.3 Hz, 1H, *trans*), 4.86 (t, *J* = 7.1 Hz, 1H), 3.75 (q, *J* = 7.2 Hz, 1H), 2.32–2.22 (m, 1H), 2.10–1.93 (m, 2H), 1.85–1.66 (m, 6H), 1.59–1.49 (m, 1H), 1.37–1.15 (m, 3H), 1.15–1.03 (m, 2H); **^{13}C NMR** (126 MHz, $CDCl_3$) δ 143.8, 128.3, 127.2, 125.9, 84.6, 80.6, 43.3, 34.7, 30.0, 29.3, 29.0, 26.8, 26.3, 26.2; **HRMS** (ESI): *m/z* $[M+Na]^+$ calcd. for $C_{16}H_{22}NaO^+$: 253.1563; found: 253.1562; **FTIR** ṽ (cm^{-1}): 2920, 2849, 1448, 1054, 696.

The diastereomeric ratio (dr) was determined by ^{1}H NMR integration of the α-protons adjacent to the oxygen atom, with characteristic signals at 4.86 ppm (*cis*-isomer) and 4.96 ppm (*trans*-isomer).

benzyl 4-((2*S,5*R**)-5-phenyltetrahydrofuran-2-yl)piperidine-1-carboxylate (2w)**

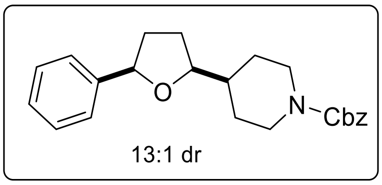

Synthesized from 1,4-diketone **1w** according to the general procedure B (20 mol% of catalyst **B1** was used). Colorless oil (58.1 mg, 79%, 13:1 dr); $\mathbf{R_f}$ 0.25 (SiO_2; *n*-hexane/EtOAc 4:1); ^{1}**H NMR** (500 MHz, $CDCl_3$) δ 7.38–7.30 (m, 9H), 7.28–7.23 (m, 1H), 5.14 (s, 2H), 4.95 (dd, *J* = 8.5, 6.2 Hz, 1H, *trans*), 4.86 (t, *J* = 7.2 Hz, 1H), 4.25 (s, 2H), 3.84–3.70 (m, 1H), 2.80 (s, 2H), 2.34–2.23 (m, 1H), 2.06–1.97 (m, 2H), 1.81–1.65 (m, 4H), 1.36–1.25 (m, 2H); ^{13}**C NMR** (126 MHz, $CDCl_3$) δ 155.4, 143.2, 137.1, 128.6, 128.4, 128.04, 127.97, 127.3, 125.9, 83.4, 80.7, 67.1, 44.2, 44.1, 41.6, 34.4, 28.9 (x2), 28.3; **HRMS** (ESI): *m/z* $[M+Na]^+$ calcd. for $C_{23}H_{27}NNaO_3^+$: 388.1883; found: 388.1881; **FTIR** ṽ (cm^{-1}): 3029, 2941, 2857, 1696, 1430, 1223.

The diastereomeric ratio (dr) was determined by ^{1}H NMR integration of the α-protons adjacent to the oxygen atom, with characteristic signals at 4.86 ppm (*cis*-isomer) and 4.95 ppm (*trans*-isomer).

(2*S,5*R**)-2-(*tert*-butyl)-5-phenyltetrahydrofuran (2x)**

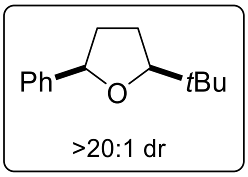

Synthesized from 1,4-diketone **1x** according to the general procedure B (10 mol% of catalyst **B1** was used).

Colorless oil (33 mg, 80%, >20:1 dr); $\mathbf{R_f}$ 0.50 (SiO_2; *n*-hexane/EtOAc 8:1); ^{1}**H NMR** (500 MHz, $CDCl_3$) δ 7.41–7.36 (m, 2H), 7.35–7.31 (m, 2H), 7.28–7.23 (m, 1H), 4.93–4.95 (m, 1H, *trans*), 4.81 (dd, *J* = 8.7, 6.6 Hz, 1H), 3.73 (t, *J* = 7.5 Hz, 1H), 2.30–2.20 (m, 1H), 1.96–1.86 (m, 1H), 1.85–1.76 (m, 1H), 1.72–1.62 (m, 1H), 1.00 (s, 9H); ^{13}**C NMR** (126 MHz, $CDCl_3$) δ 143.4, 128.3, 127.2, 126.0, 87.6, 80.8, 35.1, 34.0, 26.8, 26.1; **HRMS** (ESI): *m/z* $[M+Na]^+$ calcd. for $C_{14}H_{20}NaO^+$: 227.1406; found: 227.1407; **FTIR** ṽ (cm^{-1}): 2953, 2866, 1362, 1056, 697.

The diastereomeric ratio (dr) was determined by ^{1}H NMR integration of the α-protons adjacent to the oxygen atom, with characteristic signals at 4.81 ppm (*cis*-isomer) and 4.93–4.95 ppm (*trans*-isomer).

(2*R,5*S**)-2,5-diphenyltetrahydrofuran (2y)**

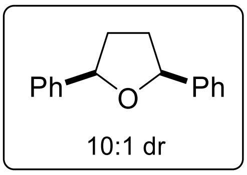

Synthesized from 1,4-diketone **1y** according to the general procedure B (10 mol% of catalyst **B1** was used).*

Yellowish oil (42 mg, 94%, 10:1 dr); **R*f*** 0.30 (SiO_2; *n*-hexane/EtOAc 8:1); **^{1}H NMR** (500 MHz, $CDCl_3$) δ 7.52–7.48 (m, 4H), 7.43–7.38 (m, 4H), 7.35–7.30 (m, 2H), 5.32–5.30 (m, 2H, *trans*), 5.14–5.06 (m, 2H), 2.51–2.43 (m, 2H), 2.06–1.98 (m, 2H); **^{13}C NMR** (126 MHz, $CDCl_3$) δ 143.1, 128.5, 127.4, 126.1, 81.3, 34.5; **HRMS** (ESI): *m/z* $[M+Na]^+$ calcd. for $C_{16}H_{16}NaO^+$: 247.1093.; found: 247.1093; **FTIR** ṽ (cm^{-1}): 2966, 2925, 2855, 1450, 1087, 697.

The diastereomeric ratio (dr) was determined by ^{1}H NMR integration of the α-protons adjacent to the oxygen atom, with characteristic signals at 5.14–5.06 ppm (*cis*-isomer) and 5.32–5.30 ppm (*trans*-isomer).

*Note: When the reaction was performed using 10 mol% of catalyst **B3**, the product was obtained in 31% yield and 20:1 dr.

(2*S,5*R**)-2-(4-nitrophenyl)-5-phenyltetrahydrofuran (2z)**

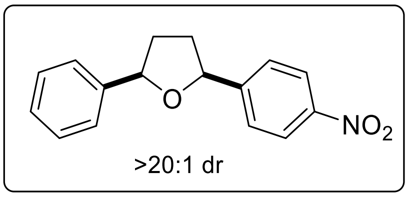

Synthesized from 1,4-diketone **1z** according to the general procedure B (10 mol% of catalyst **B1** was used) in CPME/1,2-DCE (3:1).

Yellow oil (39.2 mg, 73%, >20:1 dr) (6:1 dr in crude reaction mixture); **R*f*** 0.35 (SiO_2; *n*-hexane/EtOAc 4:1); **^{1}H NMR** (500 MHz, $CDCl_3$) δ 8.25–8.19 (m, 2H), 7.63–7.57 (m, 2H), 7.46–7.42 (m, 2H), 7.41–7.37 (m, 2H), 7.34–7.29 (m, 1H), 5.18–5.13 (m, 1H), 5.12–5.08 (m, 1H), 2.59–2.42 (m, 2H), 2.05–1.91 (m, 2H); **^{13}C NMR** (126 MHz, $CDCl_3$) δ 150.8, 147.3, 142.2, 128.6, 127.8, 126.7, 126.1, 123.8, 81.8, 80.2, 34.6, 34.0; **HRMS** (ESI): *m/z* $[M+Na]^+$ calcd. for $C_{16}H_{15}NNaO_3^+$: 292.0944; found: 292.0945; **FTIR** ṽ (cm^{-1}): 3029, 2945, 2869, 1602, 1515, 1341.

The diastereomeric ratio (dr) was determined by ^{1}H NMR integration of the α-protons adjacent to the oxygen atom, with characteristic signals at 5.15 ppm (*cis*-isomer) and 5.29 ppm (*trans*-isomer).

(2*R,5*S**)-2-phenyl-5-(4-(trifluoromethyl)phenyl)tetrahydrofuran (2aa)**

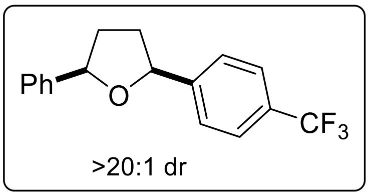

Synthesized from 1,4-diketone **1aa** according to the general procedure B (10 mol% of catalyst **B1** was used).

Yellowish oil (51 mg, 88%, >20:1 dr) (7:1 dr in crude reaction mixture); **R*f*** 0.35 (SiO_2; *n*-hexane/EtOAc 12:1); **^{1}H NMR** (500 MHz, $CDCl_3$) δ 7.63–7.55 (m, 4H), 7.47–7.43 (m, 2H), 7.41–7.36 (m, 2H), 7.33–7.28 (m, 1H), 5.34–5.26 (m, 2H, *trans*), 5.10 (dt, *J* = 14.4, 6.9 Hz, 2H), 2.57–2.40 (m, 2H), 2.06–1.90 (m, 2H); **^{13}C NMR** (126 MHz, $CDCl_3$) δ 147.3, 142.6, 129.6 (q, *J* = 32.2 Hz), 128.6, 127.6, 126.3, 126.1, 125.5 (q, *J* = 3.8 Hz), 81.6, 80.6, 34.6, 34.2, the carbon of the CF_3 group was not detected, likely because the large $^1J_{C–F}$ coupling (~270 Hz) splits the signal into a weak quartet that is buried in the noise/other signals; **^{19}F NMR** (471 MHz, $CDCl_3$) δ -62.4; **HRMS** (ESI): *m/z* $[M+Na]^+$ calcd. for $C_{17}H_{15}F_3NaO^+$: 315.0967; found: 315.0965. **FTIR** ṽ (cm^{-1}): 2975, 2873, 1322, 1062, 697.

The diastereomeric ratio (dr) was determined by ^{1}H NMR integration of the α-protons adjacent to the oxygen atom, with characteristic signals at 5.10 ppm (*cis*-isomer) and 5.34–5.26 ppm (*trans*-isomer).

(2*S,5*R**)-2-(3-bromophenyl)-5-phenyltetrahydrofuran (2ab)**

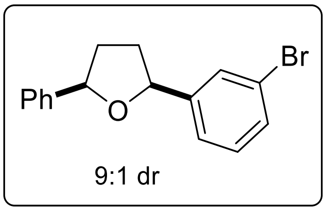

Synthesized from 1,4-diketone **1ab** according to the general procedure B (10 mol% of catalyst **B1** was used).

Colorless oil (55 mg, 91%, 9:1 dr); **R*f*** 0.45 (SiO_2; *n*-hexane/EtOAc 12:1); **^{1}H NMR** (400 MHz, $CDCl_3$) *δ* 7.62–7.60 (m, 1H), 7.47–7.36 (m, 6H), 7.33–7.29 (m, 1H), 7.24 (t, *J* = 7.8 Hz, 1H), 5.28–5.23 (m, 2H, *trans*), 5.10–5.00 (m, 2H), 2.52–2.38 (m, 2H), 2.06–1.90 (m, 2H); **^{13}C NMR** (100 MHz, $CDCl_3$) δ 145.5, 142.6, 130.4, 130.1, 129.2, 128.5, 127.6, 126.1, 124.7, 122.6, 81.5, 80.5, 34.5, 34.2; **HRMS** (ESI): *m/z* $[M+Na]^+$ calcd. for $C_{16}H_{15}BrNaO^+$: 325.0198; found: 325.0193; **FTIR** ṽ (cm^{-1}): 2943, 2869, 1474, 1049, 694.

The diastereomeric ratio (dr) was determined by ^{1}H NMR integration of the α-protons adjacent to the oxygen atom, with characteristic signals at 5.10–5.00 ppm (*cis*-isomer) and 5.28–5.23 ppm (*trans*-isomer).

(2*R,5*R**)-2-(2,6-difluorophenyl)-5-phenyltetrahydrofuran (2ac)**

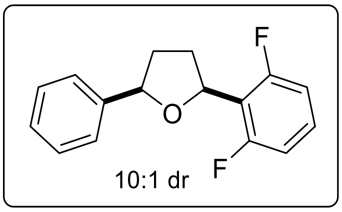

Synthesized from 1,4-diketone **1ac** according to the general procedure B (10 mol% of catalyst **B1** was used).

Colorless oil (50.3 mg, 97%, 10:1 dr); **R*f*** 0.40 (SiO_2; *n*-hexane/EtOAc 12:1); **^{1}H NMR** (500 MHz, $CDCl_3$) δ 7.50–7.45 (m, 2H), 7.39–7.34 (m, 2H), 7.30–7.21 (m, 3H), 6.93–6.87 (m, 2H), 5.64 (dd, J = 9.3, 6.8 Hz, 1H, *trans*), 5.46–5.37 (m, 1H), 4.92 (dd, J = 9.3, 5.7 Hz, 1H), 2.46–2.29 (m, 3H), 2.21–2.10 (m, 1H); **^{13}C NMR** (126 MHz, $CDCl_3$) δ 162.8 (d, *J* = 8.2 Hz), 160.8 (d, *J* = 8.2 Hz), 141.9, 129.6 (t, *J* = 10.8 Hz), 128.5, 127.7, 126.4, 112.4–111.1 (m), 82.4, 71.6 (t, *J* = 2.7 Hz), 35.1, 31.8 (t, *J* = 1.6 Hz); **^{19}F NMR** (471 MHz, $CDCl_3$) δ -113.5; **HRMS** (ESI): *m/z* $[M+Na]^+$ calcd. for $C_{16}H_{14}F_2NaO^+$: 283.0905; found: 283.0903; **FTIR** ṽ (cm^{-1}): 3030, 2954, 2873, 1625, 1591, 1470.

The diastereomeric ratio (dr) was determined by ^{1}H NMR integration of the α-protons adjacent to the oxygen atom, with characteristic signals at 5.41 ppm (*cis*-isomer) and 5.64 ppm (*trans*-isomer).

(2*R,5*S**)-2-phenyl-5-(*o*-tolyl)tetrahydrofuran (2ad)**

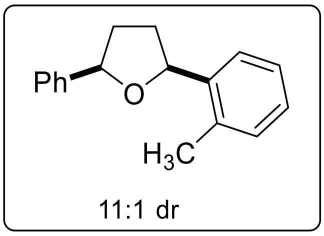

Synthesized from 1,4-diketone **1ad** according to the general procedure B (10 mol% of catalyst **B1** was used).

Colorless solid (42 mg, 89%, 11:1 dr); **R*f*** 0.45 (SiO_2; *n*-hexane/EtOAc 12:1); **m.p.** = 49–51 °C; **^{1}H NMR** (500 MHz, $CDCl_3$) δ 7.71 (dd, *J* = 7.7, 1.4 Hz, 1H), 7.56–7.52 (m, 2H), 7.45–7.40 (m, 2H), 7.37–7.31 (m, 1H), 7.30–7.25 (m, 1H), 7.25–7.17 (m, 2H), 5.49 (dd, *J* = 8.0, 6.2 Hz, 1H, *trans*) 5.26 (t, *J* = 7.3 Hz, 1H), 5.06 (dd, *J* = 8.3, 6.6 Hz, 1H), 2.57–2.43 (m, 2H), 2.40 (s, 3H), 2.05–1.97 (m, 1H), 1.96–1.87 (m, 1H); **^{13}C NMR** (126 MHz, $CDCl_3$) δ 142.8, 141.4, 134.4, 130.2, 128.5, 127.5, 127.0, 126.19, 126.16, 125.1, 81.2, 78.4, 34.3, 33.2, 19.4; **HRMS** (ESI): *m/z* $[M+Na]^+$ calcd. for $C_{17}H_{18}NaO^+$: 261.1250; found: 261.1246; **FTIR** ṽ (cm^{-1}): 2958, 2873, 1451, 1025, 697.

The diastereomeric ratio (dr) was determined by ^{1}H NMR integration of the α-protons adjacent to the oxygen atom, with characteristic signals at 5.26 ppm (*cis*-isomer) and 5.49 ppm (*trans*-isomer).

(1*R,3*S**)-1,3-diphenyl-1,3-dihydroisobenzofuran (2ae)**

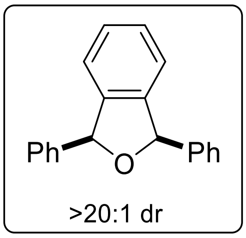

Synthesized from 1,4-diketone **1ae** according to the general procedure B (10 mol% of catalyst **B1** was used).

Green solid (31 mg, 58%, >20:1 dr); $\mathbf{R}_f$ 0.25 (SiO_2; *n*-hexane/EtOAc 12:1); **m.p.** = 90–92 °C; **^{1}H NMR** (500 MHz, $CDCl_3$) δ 7.50–7.46 (m, 4H), 7.45–7.39 (m, 4H), 7.39–7.35 (m, 2H), 7.31–7.26 (m, 2H), 7.10–7.04 (m, 2H), 6.48 (s, 2H, *trans*), 6.25 (s, 2H); **^{13}C NMR** (126 MHz, $CDCl_3$) δ 142.7, 141.4, 128.7, 128.4, 127.9 (x2), 122.3, 85.7; **HRMS** (ESI): *m/z* $[M+Na]^+$ calcd. for $C_{20}H_{16}NaO^+$: 295.1093; found: 295.1092; **FTIR** ṽ (cm^{-1}): 3027, 2852, 1451, 1004, 694.

The diastereomeric ratio (dr) was determined by ^{1}H NMR integration of the α-protons adjacent to the oxygen atom, with characteristic signals at 6.25 ppm (*cis*-isomer) and 6.48 ppm (*trans*-isomer).

(2*R,6*S**)-2,6-diphenyltetrahydro-2*H*-pyran (2af)**

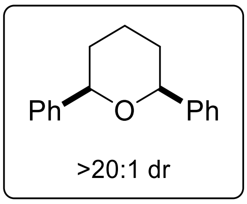

Synthesized from 1,5-diketone **1af** according to the general procedure B (10 mol% of catalyst **B1** was used).

Colorless oil (40 mg, 84%, >20:1 dr); $\mathbf{R}_f$ 0.40 (SiO_2; *n*-hexane/EtOAc 20:1); **^{1}H NMR** (500 MHz, $CDCl_3$) δ 7.48–7.43 (m, 4H), 7.38–7.33 (m, 4H), 7.29–7.24 (m, 2H), 4.88 (dd, *J* = 6.5, 4.2 Hz, 2H, *trans*), 4.59 (dd, *J* = 11.3, 2.1 Hz, 2H), 2.10–2.02 (m, 1H), 1.98–1.82 (m, 3H), 1.68–1.56 (m, 2H). **^{13}C NMR** (126 MHz, $CDCl_3$) δ 143.7, 128.4, 127.3, 126.0, 80.3, 34.0, 24.6. **HRMS** (ESI): *m/z* $[M+Na]^+$ calcd. for $C_{17}H_{18}NaO^+$: 261.1250; found: 261.1251. **FTIR** ṽ (cm^{-1}): 2934, 2850, 1450, 1043, 694.

The experimental data is in agreement with the reported for the *cis*-isomer.[32] The diastereomeric ratio (dr) was determined by ^{1}H NMR integration of the α-protons adjacent to the oxygen atom, with characteristic signals at 4.59 ppm (*cis*-isomer) and 4.88 ppm (*trans*-isomer).

***tert*-butyl(4-(2-((2*S**,6*R**)-6-(4-methoxyphenyl)tetrahydro-2*H*-pyran-2-yl)ethyl)phenoxy)dimethylsilane (2ag)**

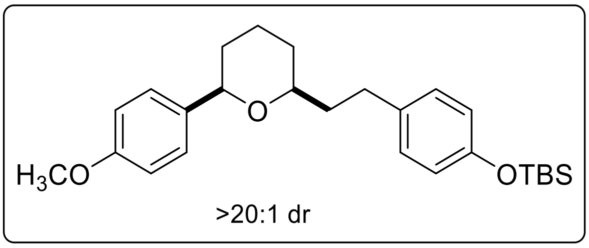

Synthesized from 1,5-diketone **1ag** according to the general procedure B (10 mol% of catalyst **B1** was used).

Colorless oil (51 mg, 60%, >20:1 dr); **R*f*** 0.45 (SiO_2; *n*-hexane/EtOAc 12:1); **^{1}H NMR** (500 MHz, $CDCl_3$) δ 7.34–7.30 (m, 2H), 7.06–7.02 (m, 2H), 6.90–6.87 (m, 2H), 6.77–6.72 (m, 2H), 4.30 (dd, *J* = 11.2, 2.2 Hz, 1H), 3.81 (s, 3H), 3.44 (dddd, *J* = 11.0, 7.7, 4.7, 1.9 Hz, 1H), 2.78–2.61 (m, 2H), 1.96–1.80 (m, 3H), 1.78–1.58 (m, 3H), 1.55–1.46 (m, 1H), 1.38–1.29 (m, 1H), 0.99 (s, 9H), 0.19 (s, 6H); **^{13}C NMR** (126 MHz, $CDCl_3$) δ 158.8, 153.6, 136.1, 135.3, 129.4, 127.2, 119.9, 113.7, 79.2, 77.3, 55.4, 38.4, 33.5, 31.4, 31.0, 25.9, 24.2, 18.3, -4.3; **HRMS** (ESI): *m/z* $[M+Na]^+$ calcd. for $C_{26}H_{38}NaO_3Si^+$: 449.2482; found: 449.2483; **FTIR** ṽ (cm^{-1}): 2930, 2856, 1610, 1510, 1248.

(±)-Centrolobine (2ag′)

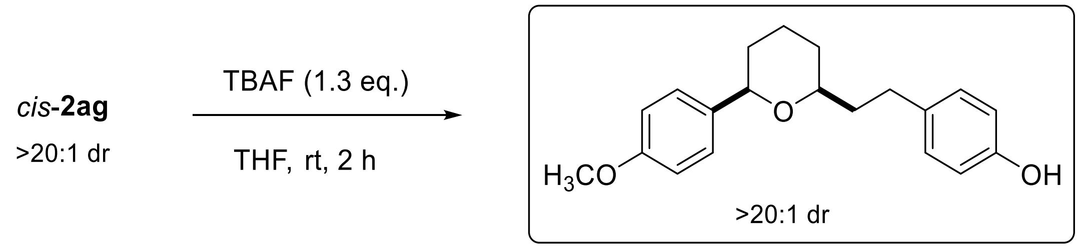

To a cooled (0 °C) stirred solution of (±)-**2ag** (42.7 mg, 0.01 mmol) in THF (1.00 mL) tetrabutylammonium fluoride (TBAF, 0.13 mL, 1 M solution in THF, 0.013 mmol) was added dropwise under argon atmosphere. The reaction was allowed to warm to room temperature and stirred for 2 hours. After completion, water was added and the aqueous phase was extracted with EtOAc (x3). The combined organic extracts were dried over Na_2SO_4, filtered, and concentrated under reduced pressure. The residue was purified by flash chromatography on silica gel eluting with *n*-hexane/EtOAc mixtures to afford (±)-Centrolobine (**2ag′**).

Colorless oil (30 mg, 95%, >20:1 dr); **R*f*** 0.30 (SiO_2; *n*-hexane/EtOAc 4:1); **^{1}H NMR** (500 MHz, $CDCl_3$) δ 7.35–7.30 (m, 2H), 7.07–7.03 (m, 2H), 6.91–6.86 (m, 2H), 6.74–6.69 (m, 2H), 4.31 (dd, *J* = 11.2, 2.2 Hz, 1H), 3.81 (s, 3H), 3.45 (dddd, *J* = 11.0, 7.8, 4.6, 1.9 Hz, 1H), 2.77–2.61 (m, 2H), 1.98–1.79 (m, 3H), 1.77–1.60 (m, 3H), 1.59–1.45 (m, 1H),

1.39–1.27 (m, 1H); **^{13}C NMR** (126 MHz, $CDCl_3$) δ 158.8, 153.6, 135.9, 134.7, 129.7, 127.3, 115.2, 113.8, 79.3, 77.4, 55.4, 38.4, 33.4, 31.4, 30.9, 24.2; **HRMS** (ESI): *m/z* $[M+Na]^+$ calcd. for $C_{20}H_{24}NaO_3^+$: 335.1618; found: 335.1618; **FTIR** ṽ (cm^{-1}): 3376, 2932, 2853, 1612, 1512,1247. The experimental data is in agreement with a previous report.[33]

methyl 2-methyl-5-phenylfuran-3-carboxylate (3ai)

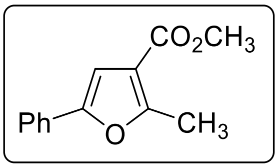

Synthesized from 1,4-diketone **1ai** following the general procedure B (10 mol% of catalyst **B1** was used).

77% NMR yield determined by analysis of the crude reaction mixture using 1,3,5-trimethoxybenzene as an internal standard; **^{1}H NMR** (500 MHz, $CDCl_3$) δ 7.65–7.62 (m, 2H), 7.40–7.36 (m, 2H), 7.29–7.25 (m, 1H), 6.87 (s, 1H), 3.84 (s, 3H), 2.64 (s, 3H). The NMR data is in agreement with a previous report.[34]

(2*S,5*R**)-2-(4-methoxyphenyl)-5-phenyltetrahydrofuran (2aj)**

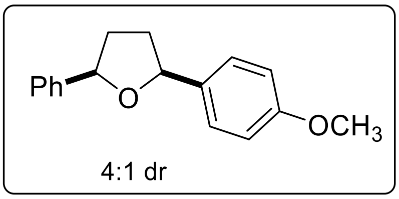

Synthesized from 1,4-diketone **1aj** according to the general procedure B (10 mol% of catalyst **B1** was used).

Yellowish oil (8 mg, 16%, 4:1 dr) (1:1 dr in crude reaction mixture); **Rf** 0.40 (SiO_2; *n*-hexane/EtOAc 12:1); **^{1}H NMR** (500 MHz, $CDCl_3$) δ 7.46–7.43 (m, 1H), 7.40–7.35 (m, 4H), 7.30–7.26 (m, 1H), 6.93–6.89 (m, 2H), 5.27–5.20 (m, 2H, *trans*) 5.08–4.96 (m, 2H), 3.82 (s, 3H), 2.53–2.34 (m, 2H), 2.06–1.92 (m, 2H). **^{13}C NMR** (126 MHz, $CDCl_3$) δ 159.1, 143.2, 135.1, 128.5, 127.5, 127.1, 126.1, 113.9, 81.22, 81.18, 55.4, 34.6, 34.4.; **HRMS** (ESI): *m/z* $[M+Na]^+$ calcd. for $C_{17}H_{18}NaO_2^+$: 277.1199.; found: 277.1196. **FTIR** ṽ (cm^{-1}): 2935, 2834, 1511, 1242, 1028, 698.

The diastereomeric ratio (dr) was determined by ^{1}H NMR integration of the α-protons adjacent to the oxygen atom, with characteristic signals at 5.08–4.96 ppm (*cis*-isomer) and 5.27–5.20 ppm (*trans*-isomer).

(2*R,3*R**,4*R**,5*S**)-3,4-dimethyl-2,5-diphenyltetrahydrofuran (2ak)**

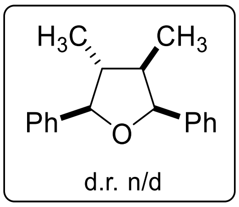

Synthesized from 1,4-diketone **1ak** according to the general procedure B (10 mol% of catalyst **B1** was used).

10% NMR yield determined by analysis of the crude reaction mixture using 1,3,5-trimethoxybenzene as an internal standard.

NMR data is in agreement with a previous report:[35] **^{1}H NMR** (lit.)[35] (400 MHz, $CDCl_3$)**:** δ 7.26–7.44 (m, 10H), 5.16 (d, 1H), 4.44 (d, 1 H), 3.32 (m, 2H), 1.16–1.00 (m, 6H).

Note: several additional minor diastereomers were detected by crude NMR.

(2*R,7*R**)-2-methyl-7-phenyloxepane (2al)**

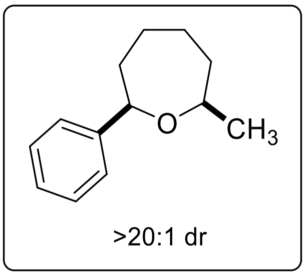

Synthesized from 1,6-diketone **1al** according to the general procedure B (10 mol% of catalyst **B3** was used).

Colorless oil (9 mg, 23%, >20:1 dr); **R*f*** 0.60 (SiO_2; *n*-hexane/EtOAc 12:1); **^{1}H NMR** (500 MHz, $CDCl_3$) δ 7.38–7.35 (m, 2H), 7.33–7.29 (m, 2H), 7.24–7.20 (m, 1H), 4.57 (dd, *J* = 9.0, 3.9 Hz, 1H), 3.88–3.82 (m, 1H), 2.09–2.02 (m, 1H), 1.89–1.73 (m, 4H), 1.68–1.59 (m, 3H), 1.24 (d, *J* = 6.3 Hz, 3H); **^{13}C NMR** (126 MHz, $CDCl_3$) δ 145.1, 128.2, 126.8, 125.8, 80.9, 76.1, 38.6, 38.2, 25.9, 24.9, 23.3; **HRMS** (ESI): *m/z* $[M+Na]^+$ calcd. for $C_{13}H_{18}NaO^+$: 213.1250; found: 213.1248; **FTIR** ṽ (cm^{-1}): 3027, 2925, 2854, 1449, 1099. The experimental data is in agreement with a previous report.[36]

2-(((2*S,5*R**)-5-phenyltetrahydrofuran-2-yl)methyl)isoindoline-1,3-dione (2am)**

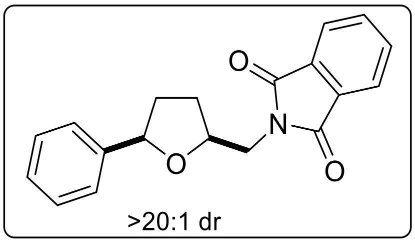

Synthesized from 1,4-diketone **1am** according to the general procedure B (20 mol% of catalyst **B3** was used) in CPME/1,2-DCE (3:1).

Colorless solid (57 mg, 93%, >20:1 dr); **m.p.** = 100–102 °C; **R*f*** 0.25 (SiO_2; *n*-hexane/EtOAc 4:1); **^{1}H NMR** (500 MHz, $CDCl_3$) δ 7.87–7.82 (m, 2H), 7.72–7.68 (m, 2H), 7.42–7.36 (m, 2H), 7.34–7.29 (m, 2H), 7.26–7.21 (m, 1H), 4.90 (t, *J* = 6.9 Hz, 1H), 4.50–4.42 (m, 1H), 4.01 (dd, *J* = 13.7, 7.4 Hz, 1H), 3.83 (dd, *J* = 13.7, 5.2 Hz, 1H), 2.36–2.25 (m, 1H), 2.19–2.08 (m, 1H), 1.92–1.82 (m, 2H); **^{13}C NMR** (126 MHz, $CDCl_3$) δ 168.5, 142.6, 134.0, 132.2, 128.4, 127.3, 125.8, 123.4, 81.7, 76.7, 42.7, 34.5, 29.6; **HRMS** (ESI): *m/z* $[M+Na]^+$

calcd. for $C_{19}H_{17}NNaO_3^+$: 330.1101; found: 330.1099; **FTIR** ṽ (cm^{-1}): 2927, 1772, 1713, 1393, 1053.

((2*S,5*R**)-5-phenyltetrahydrofuran-2-yl)methanamine (2am´)**

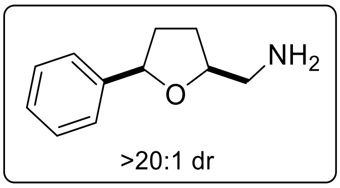

A 5 ml microwave tube equipped with a stir bar was charged with **2am** (0.1 mmol) and ethanol (1 mL). Hydrazine monohydrate (15 µL, 4.5 eq.) was added, the vial was sealed and the reaction mixture was heated to 80 °C stirring for 2 h. A white precipitate was formed during the reaction. After cooling to rt, water was added and the aqueous phase was extracted with EtOAc (x3). The combined organic extracts were dried over Na_2SO_4, filtered, and concentrated under reduced pressure to afford the deprotected product **2am´**.

Colorless oil (18 mg, >99%, >20:1 dr); **^{1}H NMR** (400 MHz, $CDCl_3$) δ 7.36–7.30 (m, 4H), 7.28–7.22 (m, 1H), 4.93–4.85 (m, 1H), 4.13–4.02 (m, 1H), 2.93 (dd, *J* = 13.1, 4.1 Hz, 1H), 2.85 (dd, *J* = 13.1, 7.0 Hz, 1H), 2.49 (br. s, 2H), 2.34–2.26 (m, 1H), 2.10–2.01 (m, 1H), 1.86–1.71 (m, 2H). The experimental data is in agreement with a previous report.[37]

2-(2-((2*S,5*S**)-5-(5-fluoro-2-methoxyphenethyl)tetrahydrofuran-2-yl)ethyl)isoindoline-1,3-dione (2an)**

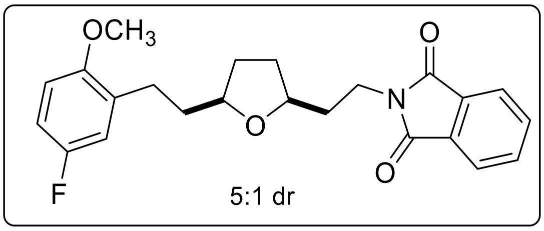

Synthesized from 1,4-diketone **1an** according to the general procedure B (20 mol% of catalyst **B1** was used) in CPME/1,2-DCE (3:1).

Colorless oil (72 mg, 91%, 5:1 dr); **R*f*** 0.30 (SiO_2; *n*-hexane/EtOAc 4:1); **^{1}H NMR** (500 MHz, $CDCl_3$) δ 7.85–7.80 (m, 2H), 7.70–7.64 (m, 2H), 6.85–6.67 (m, 3H), 3.92–3.82 (m, 2H), 3.81–3.72 (m, 5H), 2.62–2.43 (m, 2H), 2.12–1.78 (m, 4H), 1.75–1.46 (m, 4H); **^{13}C NMR** (126 MHz, $CDCl_3$) δ 168.6, 157.0 (d, *J* = 237.6 Hz), 153.6 (d, *J* = 2.0 Hz), 133.9, 132.5 (d, *J* = 7.2 Hz), 132.4, 123.2, 116.6 (d, *J* = 22.9 Hz), 112.4, 110.9 (d, *J* = 8.4 Hz), 79.1, 78.3 (*trans*), 77.3, 55.9, 35.9, 34.7, 31.3, 30.9, 27.0, 26.9; **^{19}F NMR** (471 MHz, $CDCl_3$) δ -124.59 (*trans*), -124.63; **HRMS** (ESI): *m/z* [M+Na]$^+$ calcd. for $C_{23}H_{24}FNNaO_4^+$: 420.1582; found: 420.1582; **FTIR** ṽ (cm^{-1}): 2937, 2861, 1771, 1705, 1497.

The diastereomeric ratio (dr) was determined by ^{19}F NMR, comparing the signals at -124.59 ppm (*trans*-isomer) and -124.63 ppm (*cis*-isomer).

2-((2*S,5*S**)-5-(5-fluoro-2-methoxyphenethyl)tetrahydrofuran-2-yl)ethan-1-amine (2an´)**

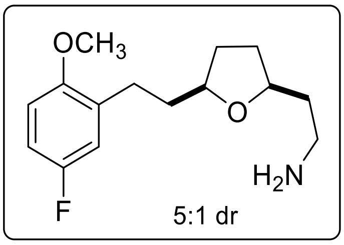

Synthesized from **2an** following the same procedure described for compound **2an´**.

Colourless oil (27 mg, >99%, 5:1 dr). **^{1}H NMR** (400 MHz, $CDCl_3$) δ 6.87–6.77 (m, 2H, *cis+trans*), 6.71 (dd, *J* = 8.8, 4.6 Hz, 1H, *cis+trans*), 4.06–3.99 (m, 1H, *trans*), 3.95 (d, *J* = 5.5 Hz, 1H, *trans*), 3.92–3.84 (m, 1H, *cis*), 3.84–3.78 (m, 1H, *cis*), 3.77 (s, 3H, *cis+trans*), 2.85 (t, *J* = 6.8 Hz, 2H, *cis+trans*), 2.73–2.47 (m, 4H, *cis+trans*), 2.04–2.01 (m, 2H, *trans*), 2.00–1.89 (m, 2H, *cis*), 1.88–1.77 (m, 1H, *cis+trans*), 1.75–1.44 (m, 5H, *cis+trans*); **^{19}F NMR** (377 MHz, $CDCl_3$) δ -124.57 *(trans*), -124.59 *(cis*). The experimental data is in agreement with a previous report.[38] The diastereomeric ratio (dr) was determined by ^{19}F NMR, comparing the signals at -124.57 ppm (*trans*-isomer) and -124.59 ppm (*cis*-isomer).

5. Gram-scale reaction

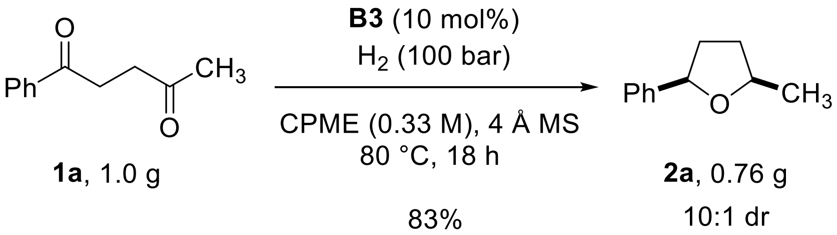

Without any precautions from air and moisture, a 50 mL high-pressure steel-autoclave vessel equipped with a stirring bar, was charged with pre-activated 4 Å MS (8.0 g), borane catalyst **B3** (280 mg, 10 mol%)**,** diketone substrate **1a** (1.0 g, 5.7 mmol) and CPME (17 mL). The vessel was then connected to the gas line, purged three times with nitrogen (6 bar) and four times with hydrogen (3 bar). The reactor was then pressurized at 100 bar with hydrogen gas and left stirring for 18 h at 80 °C. After 18 h, the autoclave was allowed to cool to room temperature and the gas slowly vented off to atmospheric pressure. The reaction mixture was then filtered on a short silica plug eluting with ethyl acetate. The filtrate was then concentrated under reduced pressure. Purification by silica gel flash column chromatography, using an eluent gradient from *n*-hexane to *n*-hexane/ethyl acetate 8:1, afforded **2a** (0.76 g, 83% yield) with a 10:1 dr. The compound's experimental data matched the one reported above.

6. Mechanistic experiments

Scheme S2. Deuterium labelling

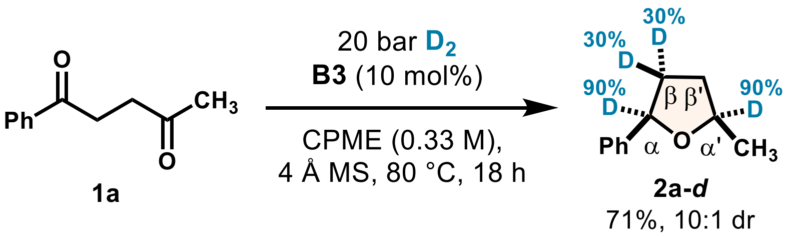

The reaction was performed following the General Procedure B using 20 bar of D_2 instead of H_2 gas. Deuterium incorporation was observed at the α-, α′-, and β-positions relative to the oxygen atom (90%, 90% and 30% respectively). The regioselectivity of the D-incorporation was assigned based on ^1H–^1H COSY NMR analysis of non-deuterated **2a**.

^{1}H–^{1}H COSY NMR spectrum of 2a

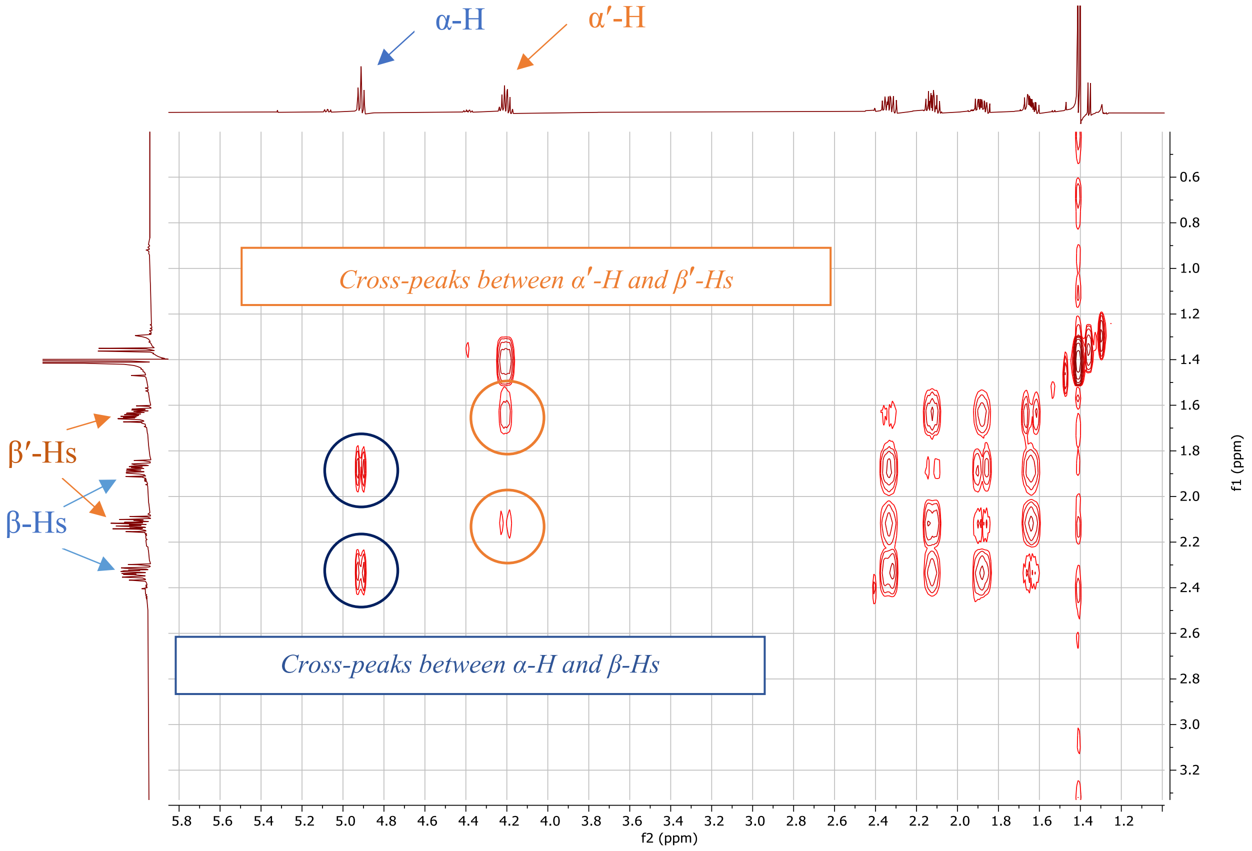

^{1}H NMR of 2a-*d*

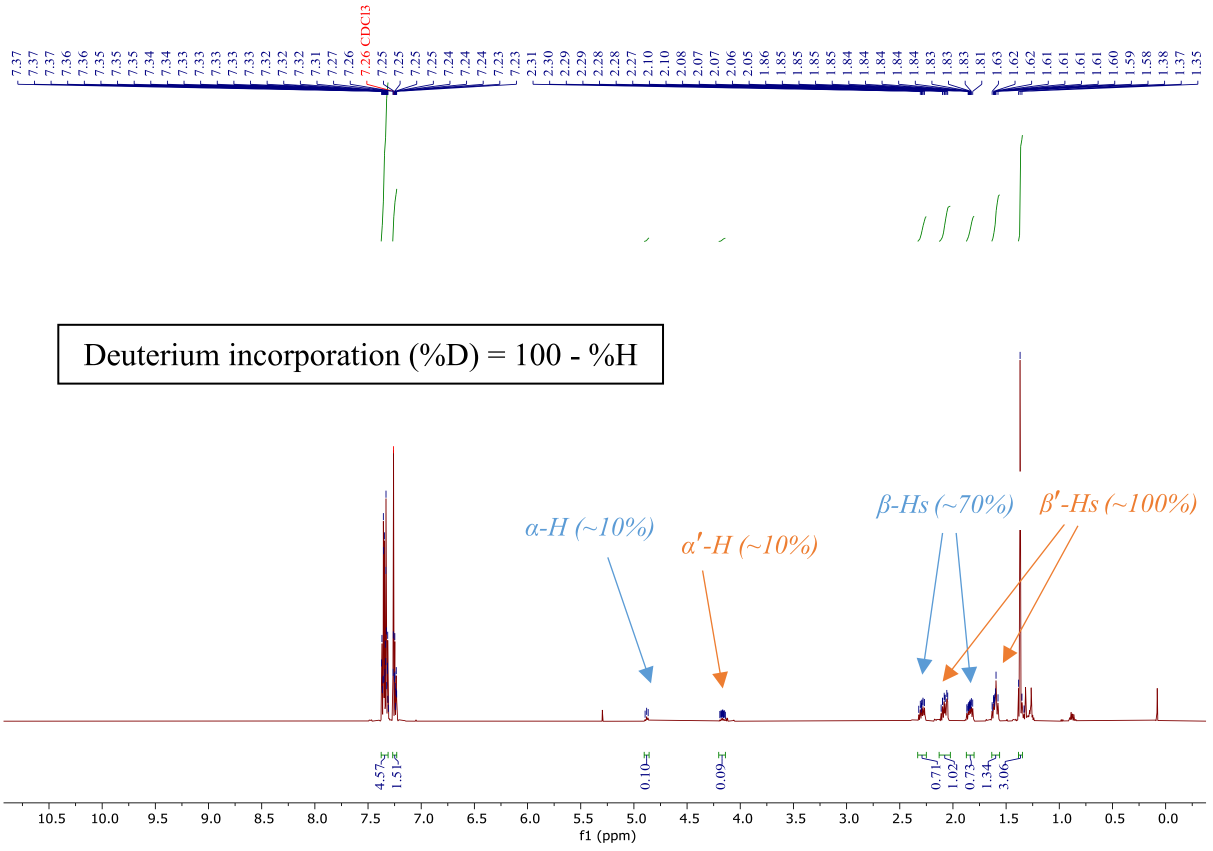

The observed deuteration at the α and α′ positions is fully consistent with the proposed mechanism, as it originates from "deuteride" transfer to the carbonyl group and to the oxocarbenium ion intermediate. Nevertheless, ~90% incorporation (rather than quantitative) suggests that some D/H exchange could occur during the reaction, for example, via enolization of the oxocarbenium ion which releases H^+ (consistent with the proposed mechanism), or due to the presence of trace moisture. Moreover, minor H-incorporation can also be explained by the isotopic purity of commercial D_2 gas (which contains ~5% H), or even by the intrinsic error of NMR-based quantification.

As discussed in the manuscript, β-deuteration suggests the existence of an equilibrium between oxocarbenium ion and enol ether intermediates, which interconvert faster than undergo reduction.

Scheme S3. Racemization test under standard conditions

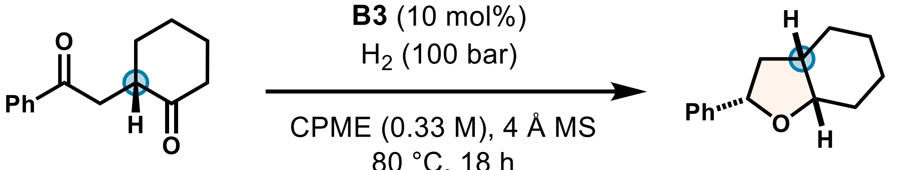

(*S*)-**1q**, 94:6 er

2q, 70%, 15:1 dr
56:44 er

The reaction was performed following the General Procedure B. Purification by silica gel flash column chromatography, using an eluent gradient from *n*-hexane to *n*-hexane/ethyl acetate 12:1, afforded **2q** (28.4 mg, 70% yield) with a 15:1 dr. The compound's experimental data matched the one reported above in section 4. The enantiomeric ratio was determined by chiral stationary phase HPLC with an IBN-5 column (*n*-hexane/^{i}PrOH 2%, 25 °C, 1 mL/min), UV detection at λ = 210 nm, t_R (minor) = 5.1 min, t_R (major) = 6.0 min, er = 44:56.

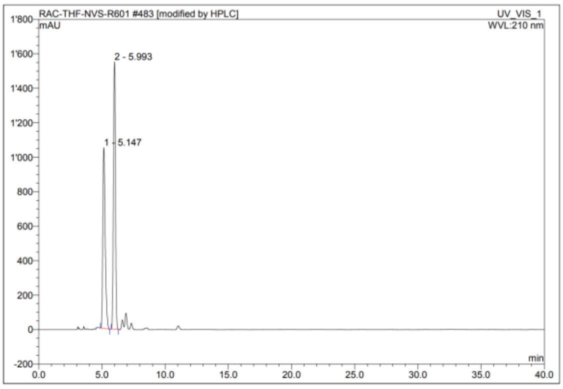

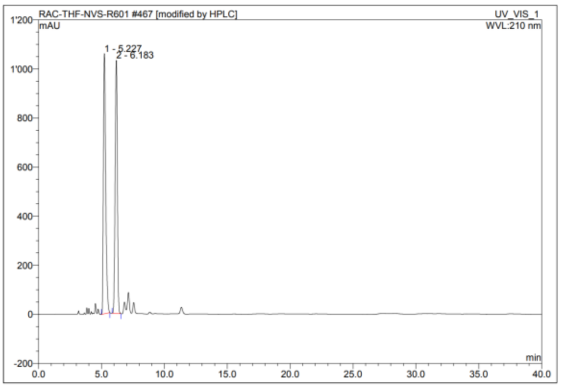

No.	Ret.Time min	Peak Name	Height mAU	Area mAU*min	Rel.Area %	Amount	Type
1	5.15	n.a.	1049.094	230.716	44.30	n.a.	BMB*
2	5.99	n.a.	1551.310	290.074	55.70	n.a.	BMB*
Total:			2600.403	520.791	100.00	0.000	

No.	Ret.Time min	Peak Name	Height mAU	Area mAU*min	Rel.Area %	Amount	Type
1	5.23	n.a.	1059.547	227.453	51.69	n.a.	BMB*
2	6.18	n.a.	1030.893	212.557	48.31	n.a.	BMB*
Total:			2090.440	440.010	100.00	0.000	

In a separate experiment, we tested whether racemization could occur *via* borane-catalyzed enolization of the starting material prior to hydrogenation/cyclization. To this end, the enantioenriched substrate *(S)*-**1q** was subjected to the standard reaction conditions in the absence of hydrogen (see below).

Scheme S4. Racemization test without hydrogen

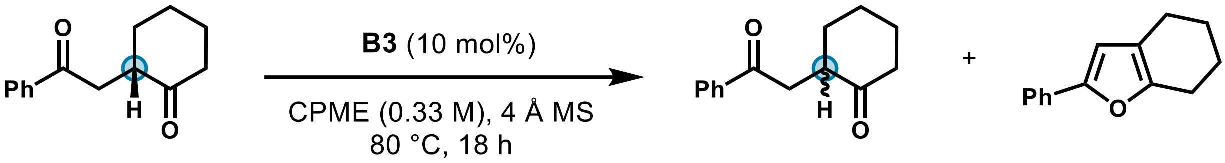

(*S*)-**1q** 94:6 er — **1q**, ~58% 50:50 er — **3q**, ~42%

The reaction was performed following the General Procedure B without H_2. ^{1}H NMR analysis of the crude reaction mixture showed a 1.4:1 ratio of starting material **1q** and the corresponding furan **3q**.[39]

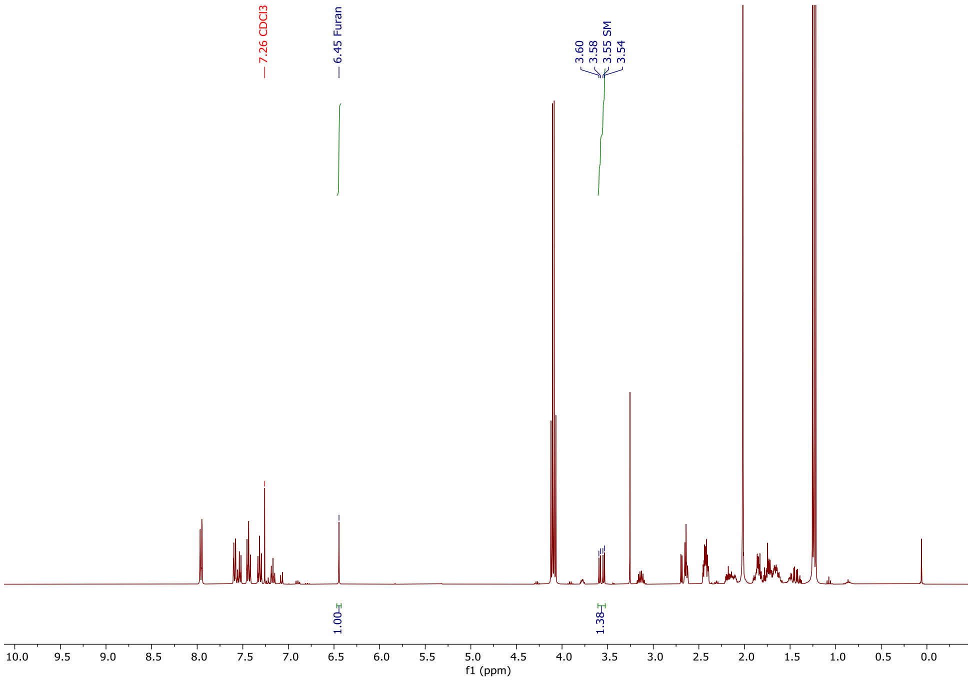

The enantiomeric ratio of the remaining **1q** was determined by chiral stationary phase HPLC with an IC column (*n*-hexane/^{i}PrOH 10%, 25 °C, 1 mL/min), UV detection at λ = 254 nm, t_R (major) = 27.3 min, t_R (minor) = 27.3 min, er = 50:50.

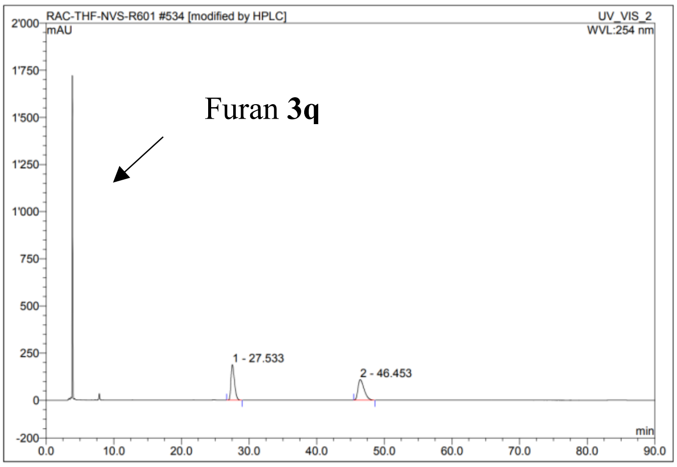

No.	Ret.Time min	Peak Name	Height mAU	Area mAU*min	Rel.Area %	Amount	Type
1	27.53	n.a.	188.679	113.428	49.74	n.a.	BMB*
2	46.45	n.a.	109.219	114.634	50.26	n.a.	BMB*
Total:			297.898	228.062	100.00	0.000	

The observed complete racemization of diketone (*S*)-**1q,** together with the partial formation of furan **3q,** supports that: i) the starting material can undergo Lewis acid-mediated enolization favouring Paal-Knorr cyclization; and that ii) the near-complete racemization observed after the hydrogenation reaction (Scheme S3) can be partially attributed to racemization of the starting material.

Scheme S5. γ-Hydroxy ketone hydrogenative cyclization.

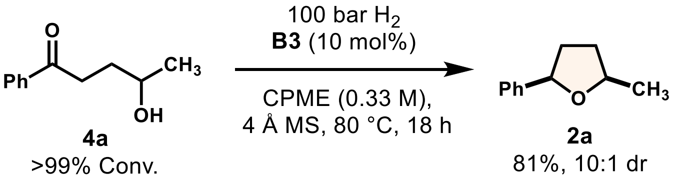

The reaction was performed following the General Procedure B using *rac*-4-hydroxy-1-phenylpentan-1-one **4a** as substrate. **4a**: **^{1}H NMR** (400 MHz, $CDCl_3$)[40] δ 8.01–7.91 (m, 2H), 7.55 (t, J = 7.4 Hz, 1H), 7.45 (t, J = 7.6 Hz, 2H), 4.00–3.74 (m, 1H), 3.22–2.97 (m, 2H), 1.95 (dtd, J = 11.1, 7.3, 4.1 Hz, 2H), 1.92–1.70 (m, 1H), 1.25 (d, J = 6.2 Hz, 3H).

Quantitative ^{1}H NMR analysis of the crude reaction mixture using 1,3,5-trimethoxybenzene as internal standard showed full consumption of the starting material and 81% of **2a** with a 10:1 dr (spectrum attached below). The diastereomeric ratio (dr) was determined by integration of the α-protons adjacent to the oxygen atom, with characteristic signals at 4.89 ppm (*cis*-isomer) and 5.05 ppm (*trans*-isomer).

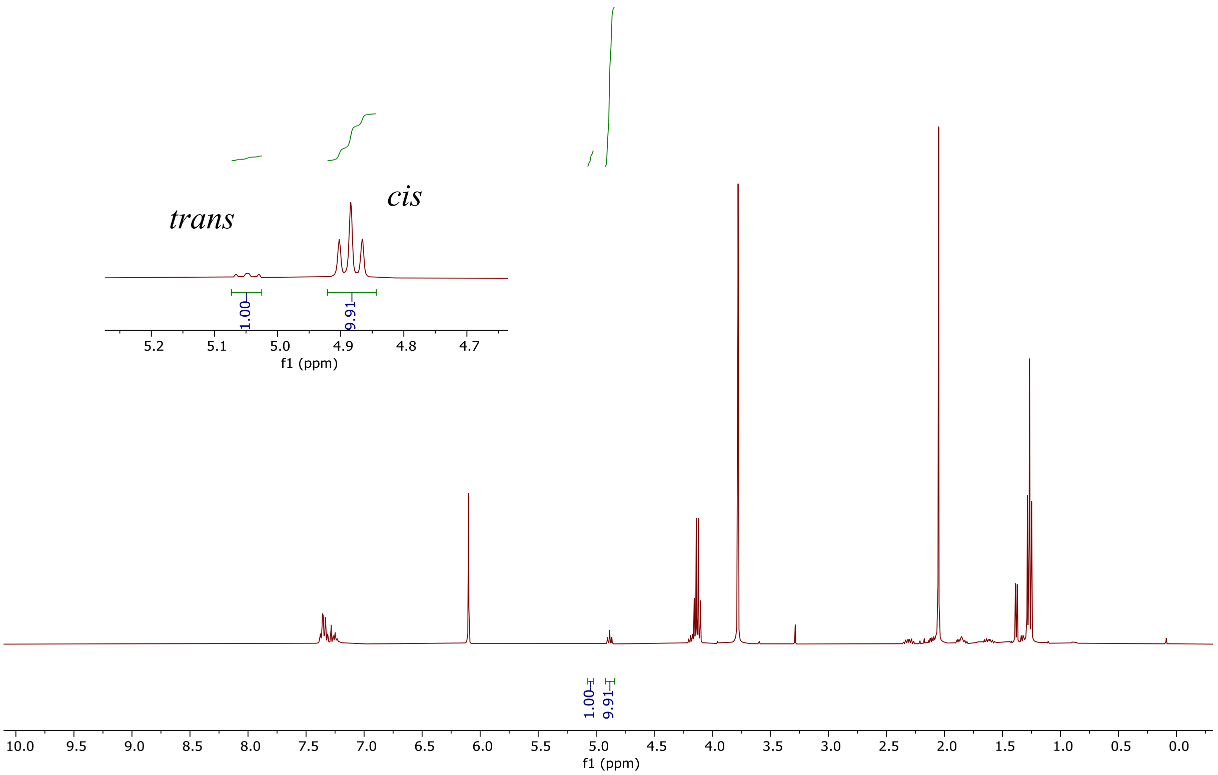

Scheme S6. Attempted furan hydrogenation

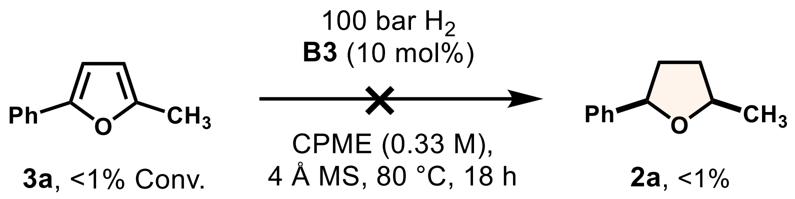

The reaction was performed following the General Procedure B using furan **3a** as substrate. **3a**: **^{1}H NMR** (300 MHz, $CDCl_3$)[39] δ 7.63 (d, J = 6 Hz, 2H), 7.35 (t, J = 6 Hz, 2H), 7.21 (t, J = 6 Hz, 1H), 6.53 (d, J = 3Hz, 1H), 6.05 (d, J = 3 Hz, 1H), 2.37 (s, 3H).

^{1}H NMR analysis of the crude reaction mixture showed only starting material present (1,3,5-trimethoxybenzene was used as internal standard; spectrum attached below). This result rules out a reaction pathway proceeding *via* Lewis acid-promoted cyclization to a furan intermediate followed by hydrogenation.

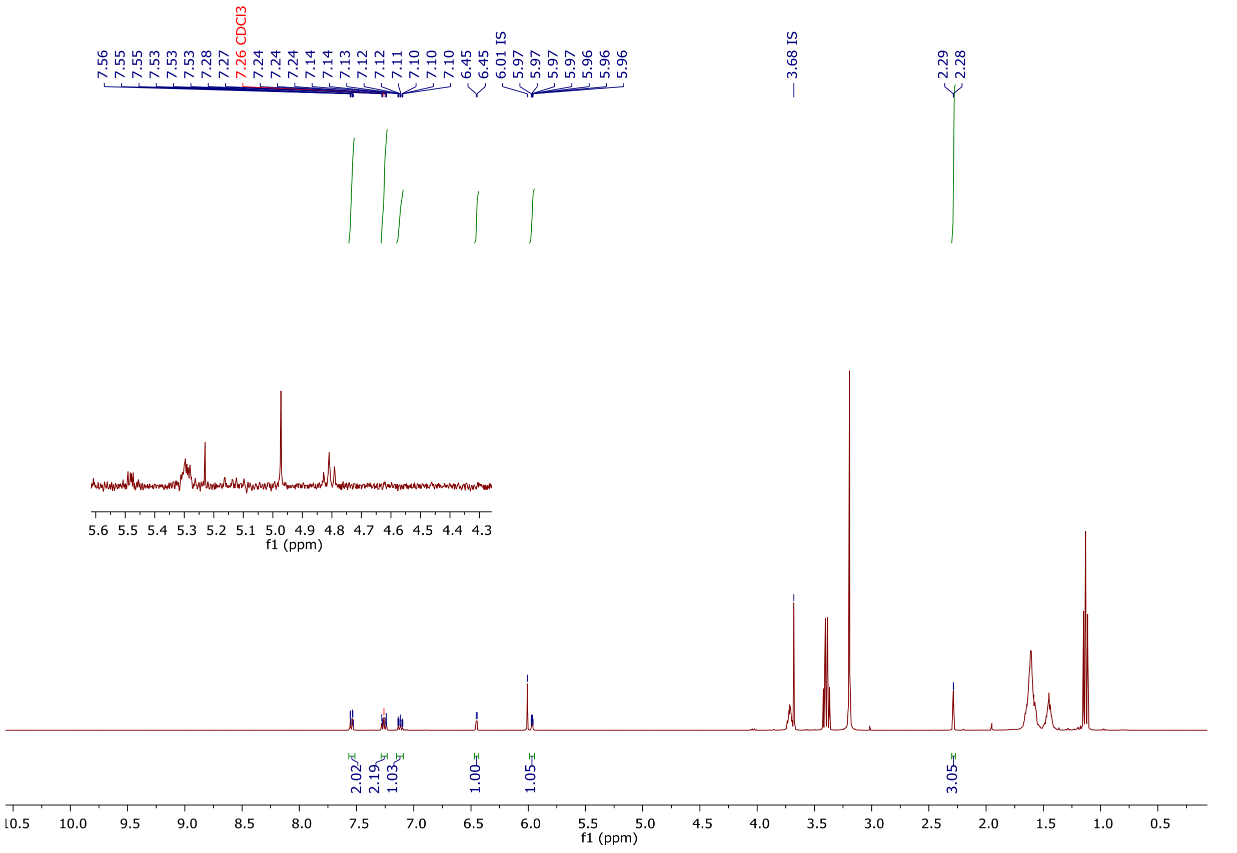

Scheme S7. Diol cyclodehydration.

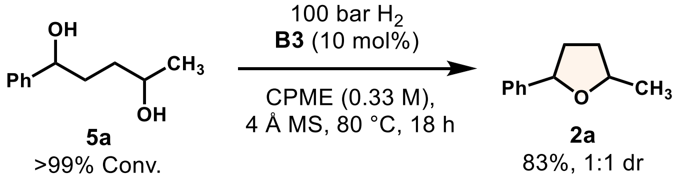

The reaction was performed following the General Procedure B using 1-phenylpentane-1,4-diol **5a** as substrate. **5a**: **^{1}H NMR** (400 MHz, $CDCl_3$)[41] δ 7.37–7.29 (m, 4H), 7.29–7.23 (m, 1H), 4.75–4.63 (m, 1H), 3.90–3.76 (m, 1H), 1.93–1.77 (m, 2H), 1.66–1.40 (m, 2H), 1.17 (dd, J = 6.2, 1.8 Hz, 3H).

Quantitative ^{1}H NMR analysis of the crude reaction mixture using 1,3,5-trimethoxybenzene as internal standard showed full consumption of the starting material and 83% of **2a** with a 1:1 dr (spectrum attached below). The diastereomeric ratio (dr) was determined by integration of the α-protons adjacent to the oxygen atom, with characteristic signals at 4.89 ppm (*cis*-isomer) and 5.05 ppm (*trans*-isomer).

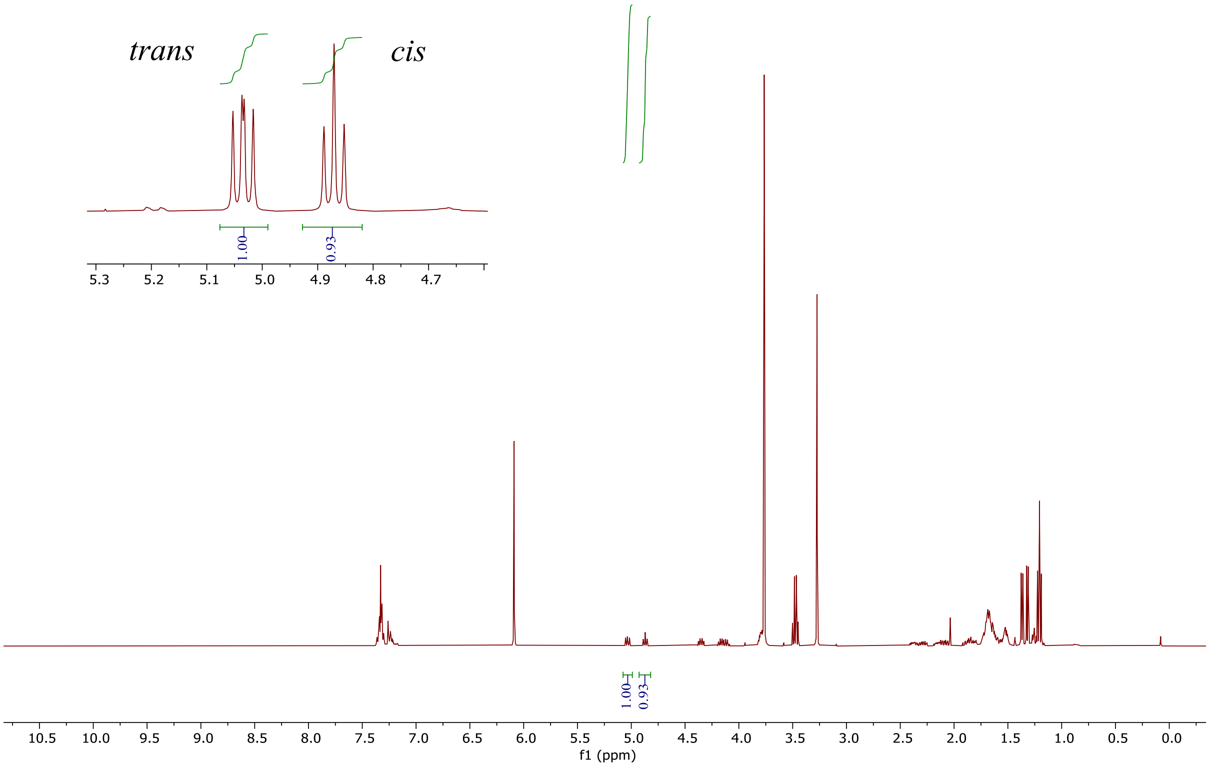

7. Computational details

7.1. General information

All calculations were performed on the Euler cluster at ETH Zurich.

The conformational space of the examined species was explored using xTB version 6.6.1,[42,43] CREST version 2.12,[44,45] and CENSO version 1.2.0.[46] For CREST and CENSO, the ALPB solvent model (ether) was employed.[47] For CENSO, steps 0–2 were used. Additional conformers were added manually if necessary.

The energetically most favorable structures were subjected to quantum chemical calculations. All DFT calculations were conducted using ORCA version 6.0.1.[48,49] Electronic structure calculations were carried out using the range-separated hybrid exchange-correlation functional ωB97X-D3 with dispersion correction.[50–53] The def2-TZVP basis set was employed for both geometry optimizations and single-point energy calculations.[54,55]

Solvent effects were considered by computing solvation free energies at the ωB97X-D3/def2-TZVP level using gas-phase optimized geometries and applying the CPCM solvation model (solvent = diethyl ether).[56]

Thermochemical data were computed using the ideal gas–rigid rotor–harmonic oscillator model at the experimental temperature of the catalytic hydrogenation reactions (T = 353.15 K).

The reported energies in the manuscript correspond to solution-phase Gibbs free energies, unless otherwise stated, obtained from ωB97X-D3/def2-TZVP electronic energies combined with all other contributions calculated at the same level of theory. Ground-state structures were confirmed by the absence of imaginary frequencies in frequency calculations, whereas all reported transition states exhibit exactly one imaginary frequency corresponding to the appropriate reaction coordinate.

Non-covalent interaction (NCI) analyses were performed using the NCIPLOT package (default settings),[57] and the results were visualized with VMD 1.9.3.[58]

All calculated structures and imaginary frequencies were visualized with Chemcraft 1.8.[59]

7.2. Calculation of the Lewis acidity of the boranes

To assess the Lewis-acidity of the borane catalysts, we computed their fluoride ion affinity (FIA) at the ωB97X-D3/def2-TZVP level of theory in the gas phase under standard conditions (298.15 K, 1.00 atm). Additionally, we evaluated their hydride ion affinity (HIA) —another established metric of Lewis acidity relevant for hydrogenation catalysts.[2] Both values were calculated as the negative reaction enthalpies of the following reactions:[60]

1) FIA: $LA + F^{-} \rightarrow [LA\text{-}F]^{-}$
2) HIA: $LA + H^{-} \rightarrow [LA\text{-}H]^{-}$

The results are summarized in Table S4.

Table S4. FIA and HIA values of boranes

**Catalyst**	**FIA**, kJ/mol	**%LA$_{FIA}$**	**HIA**, kJ/mol	**%LA$_{HIA}$**
**B1**	467	100	519	100
**B2**	409	88	464	89
**B3**	423	91	477	92

Higher values indicate stronger Lewis acidity. The relative Lewis acidity (%LA) values are calculated by normalizing to catalyst B1 (set at 100%) using the formula: (Bx / B1) × 100. The similar trends observed for both FIA- and HIA-based acidity scales support the consistency and validity of these descriptors for evaluating Lewis acidity in this system.

7.3. Calculations on the oxocarbenium ion reduction

For the DFT calculations, the symmetrical 1,4-diphenylbutane-1,4-dione (**1y**) was selected as the model substrate. The symmetry of **1y** ensures equivalent reactivity of both carbonyl groups, thereby reducing the number of possible intermediates and conformers that must be considered. This allows for a more straightforward computational analysis without compromising the validity of the conclusions. The energy diagram for the reduction of the **2y**-oxonium ion is shown below, with key findings discussed in the manuscript.

The zero level (0 kcal/mol) used as a reference for the estimation of relative stabilities corresponds to **B3** + **1y** + 2H_2; H_2O is included in all other energy points but is not depicted for simplicity.

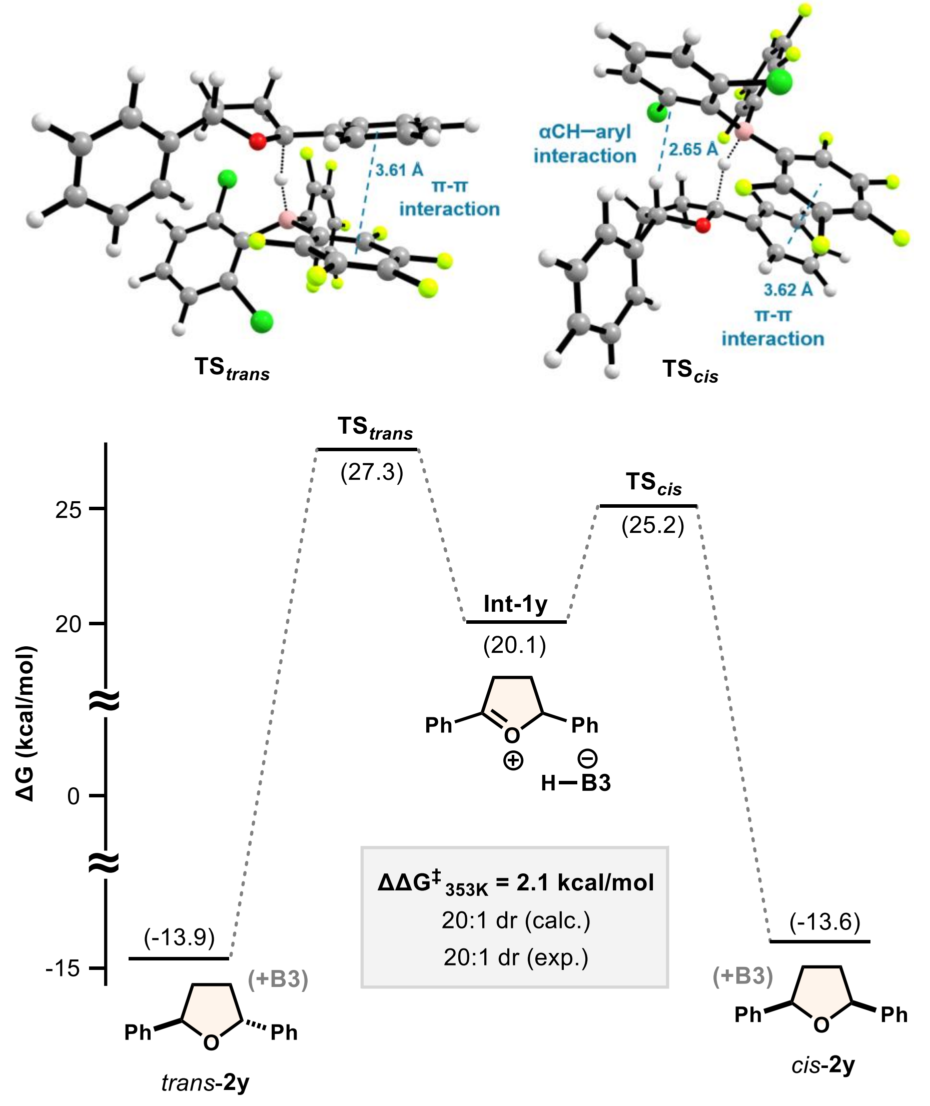

Analysis of non-covalent interactions

Contour plots of the density gradient isosurfaces for **TS*trans*** and **TS*cis*** of **Int-1y** with catalysts **B3** and **B1** catalysts are shown. The surface color code is: blue for strongly attractive, green for weak van der Waals interactions, and red for strongly repulsive interactions.

With catalyst **B3**, both the NCI analysis and interatomic distance analysis support a non-random C–H···aryl edge contact between the aliphatic *cis*-α-CH of the substrate and a C*sp*² carbon of the catalyst's aromatic ring (H···C distance = 2.65 Å, ~0.25 Å shorter than the vdW sum). This interaction is observed in the TS*cis* but not in the TS*trans*:

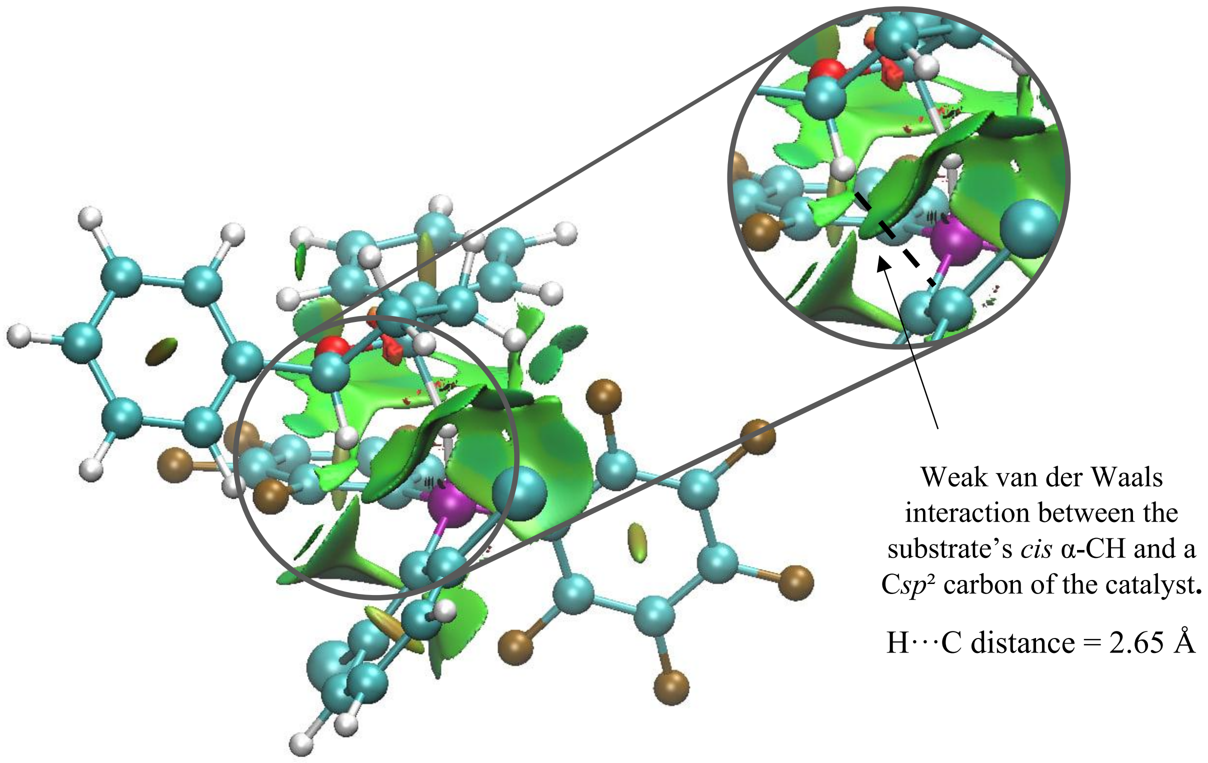

TS*cis* (with B3)

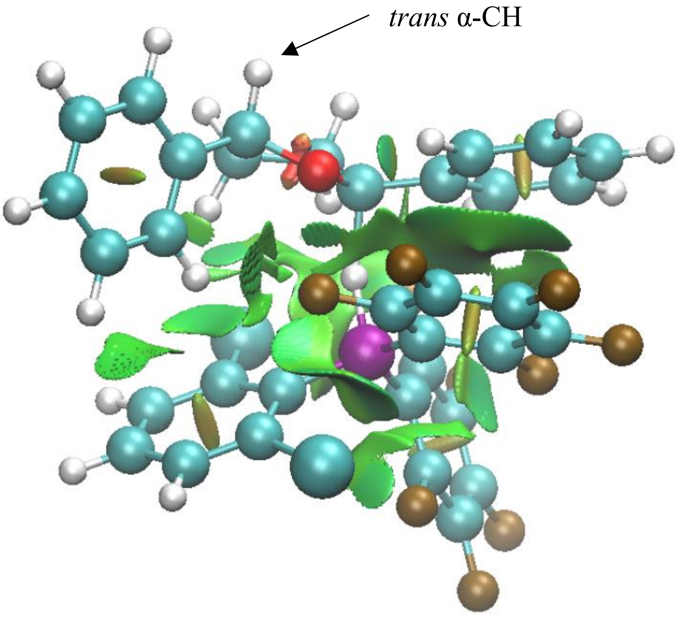

TS$_{trans}$ (with B3)

With catalyst **B1**, a similar interaction is detected (H···C distance = 2.76 Å, ~0.14 Å shorter than the vdW sum):

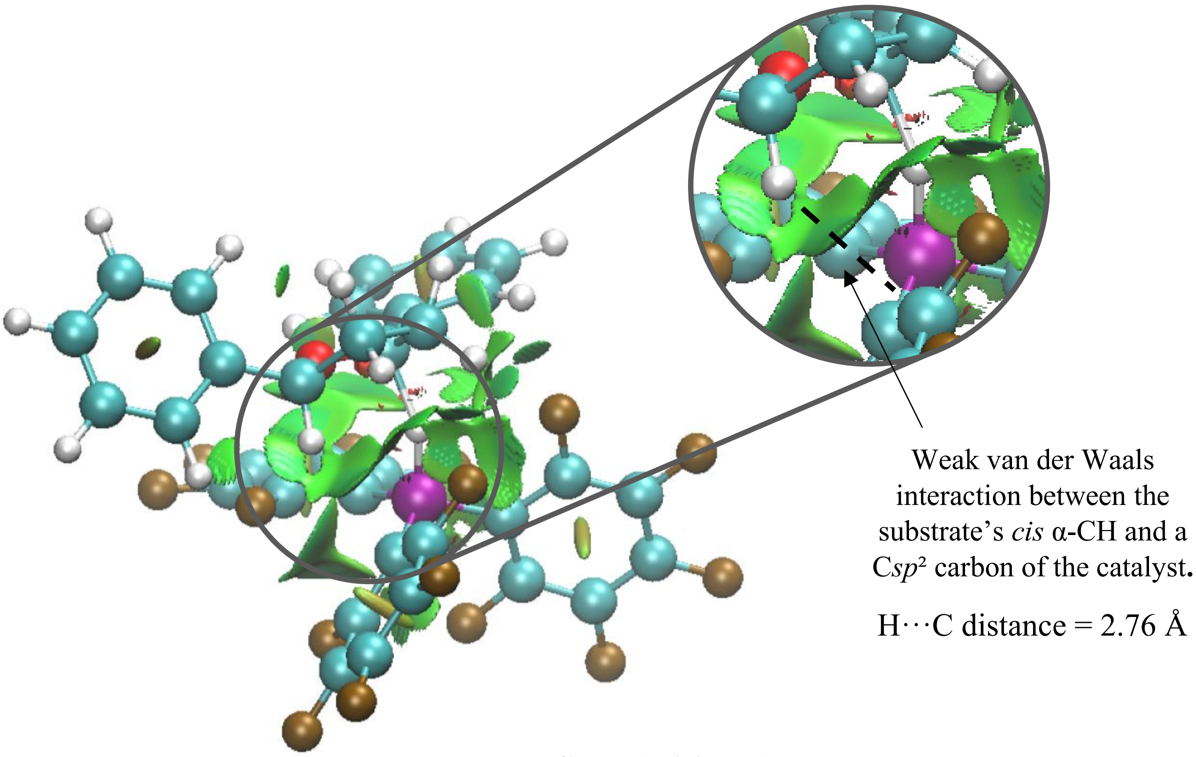

TS$_{cis}$ (with B1)

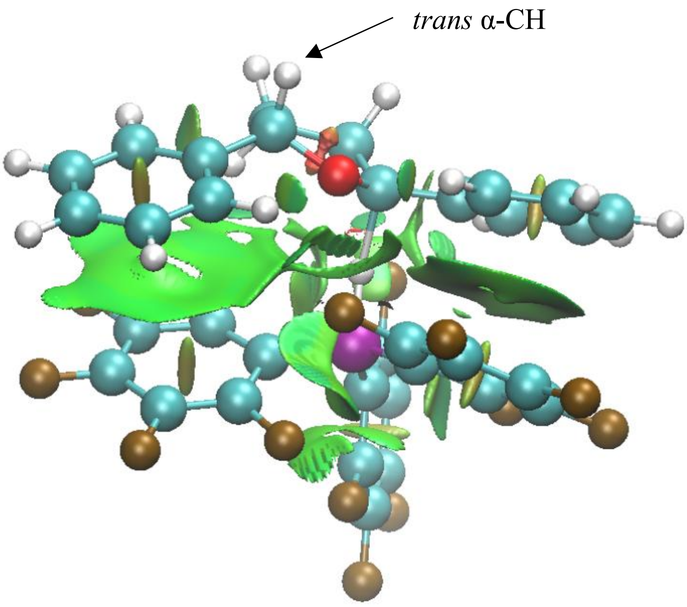

TS$_{\textbf{\textit{trans}}}$ (with B1)

In addition, arene−perfluoroarene offset π−π stacking interactions are observed between **Int-2y** and **B3** in both TS$_{cis}$ and TS$_{trans}$:

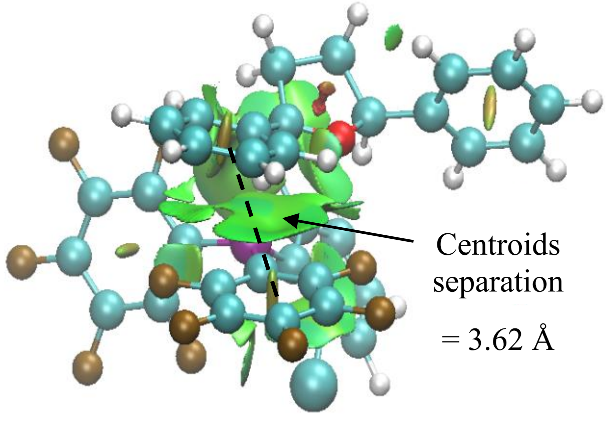

TS$_{\textbf{\textit{cis}}}$ (with B3)

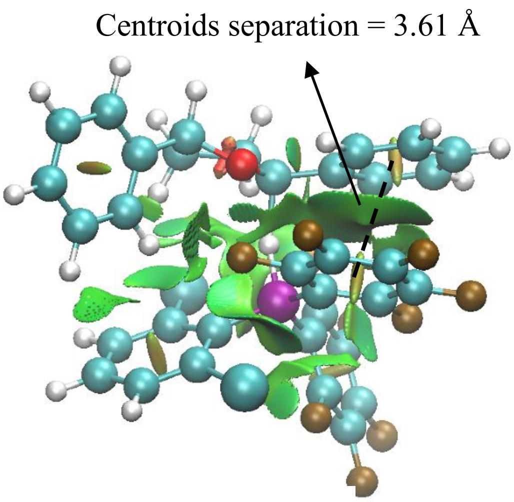

TS$_{\textbf{\textit{trans}}}$ (with B3)

The centroid separations (3.62 for TS$_{cis}$ and 3.61 Å for TS$_{trans}$) fall within the typical range for near-parallel aromatic stacking interactions (3.3–3.8 Å),[61] further supporting their presence.

Table S5. Comparison of energetic differences of cis/trans products according to ring size (ΔΔG) and energies of transition states (ΔΔG‡) with different borane catalysts.

Entry	Product	Ring size	Cat.	$\Delta\Delta G^{\ddagger}_{353K}$, kcal/mol	$\Delta\Delta G_{353K}$, kcal/mol	*cis:trans*$_{exp}$	*cis:trans*$_{calc}$
1	**2y**	5	**B3**	2.1	–0.3	20:1	~20:1
2	**2y**	5	**B1**	0.7	–0.3	10:1	~3:1
3	**2af**	6	**B1**	4.1	2.6	>20:1	~341:1

ΔΔG‡ and **ΔΔG** are reported as ΔG(trans) – ΔG(cis). Calculated cis:trans ratios (cis:trans$_{calc}$) were obtained based on the Boltzmann distribution as: cis/trans = exp(–ΔΔG‡/RT) at T = 353.15 K.[62]

Comparison of entries 1 and 2 shows the influence of the catalyst on the selectivity of the 5-membered oxonium ion reduction towards product **2y**. With the less sterically demanding catalyst **B1**, the energy difference between the diastereomeric transition states (ΔΔG‡ = 0.7 kcal/mol) is considerably smaller than with the bulkier catalyst **B3** (ΔΔG‡ = 2.1 kcal/mol). For **B3**, the computed ΔΔG‡ value matches the experimental cis/trans ratio (20:1). The slight discrepancy between *cis:trans*$_{\text{exp}}$ and *cis:trans*$_{\text{calc}}$ for **B1** (entry 2) likely stems from limitations in conformational sampling: for simplicity as we considered only the lowest-energy conformer of each transition state rather than an ensemble of low-lying conformers. Nevertheless, the trend between theory and experiment is consistent, supporting the mechanistic model and the hypothesis that bulkier catalysts provide enhanced stereoselectivity.

A comparison between entries 2 and 3 highlights the influence of the product's ring size. For the six-membered tetrahydropyran product **2af** (entry 3), both kinetic and thermodynamic factors favor formation of the *cis*-isomer (ΔΔG‡ = 4.1 kcal/mol, ΔΔG = 2.6 kcal/mol). In contrast, for the analogous five-membered tetrahydrofuran product **2y** (entry 2**)**, the *cis* and *trans* isomers are nearly isoenergetic (ΔΔG = –0.3 kcal/mol), indicating that stereoselectivity for these substrates is governed primarily by the kinetics of the reduction step (i.e., ΔΔG‡).

The transition-state geometries computed for the reduction of the **2af**-oxonium ion by the borohydride derived from catalyst **B1** (illustrated below) are consistent with these energetic trends: the TS$_{cis}$ adopts a favorable chair-like conformation, whereas the TS$_{trans}$ ends in a less favorable boat-like geometry, providing a structural rationale for the computed energy differences.

TS$_{cis}$

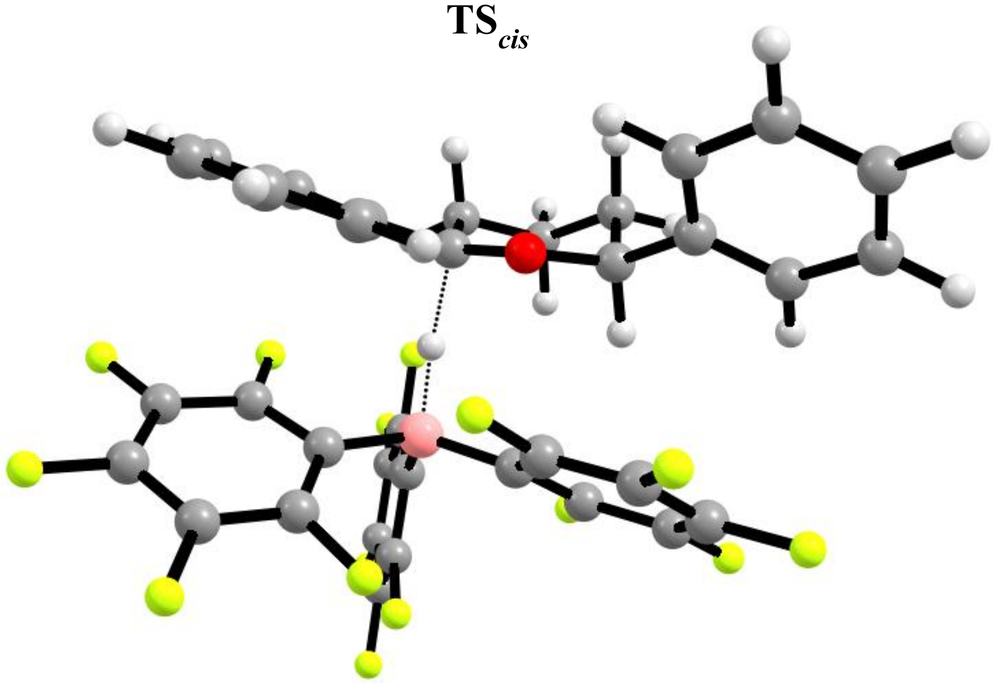

TS$_{cis}$ imaginary frequency – end point (chair-like conformation).

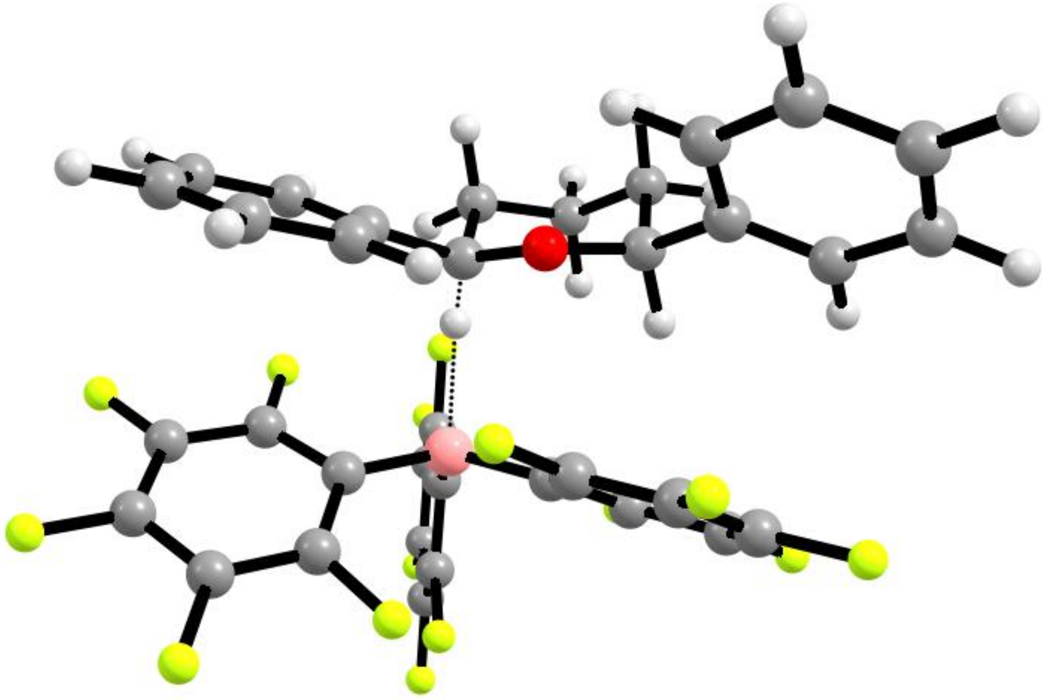

TS*trans*

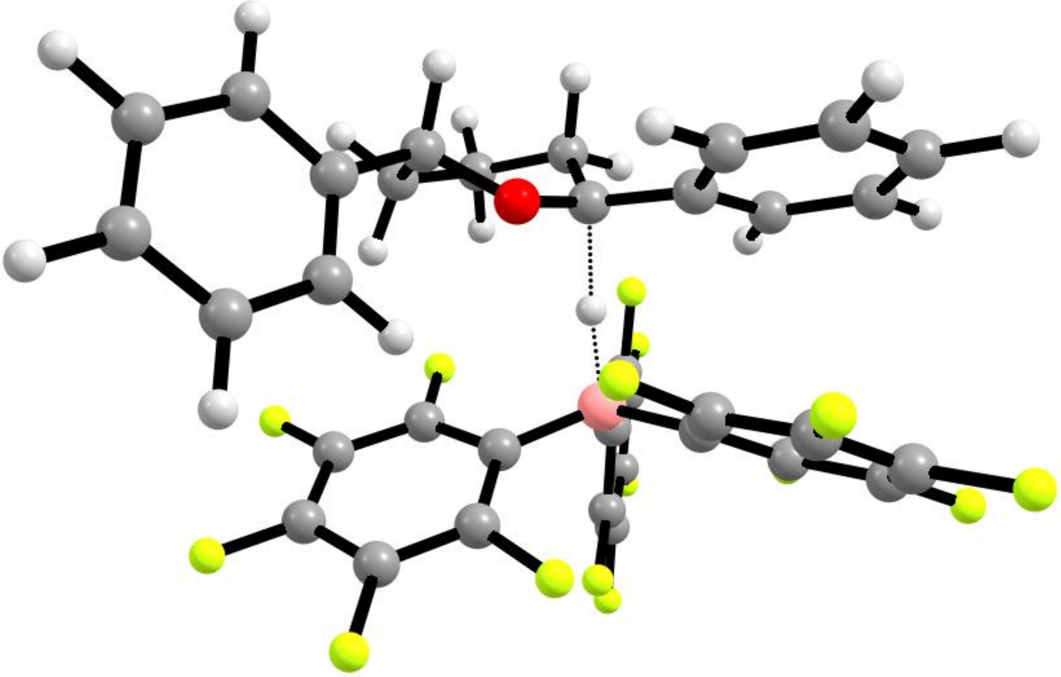

TS*trans* imaginary frequency – end point (boat-like conformation).

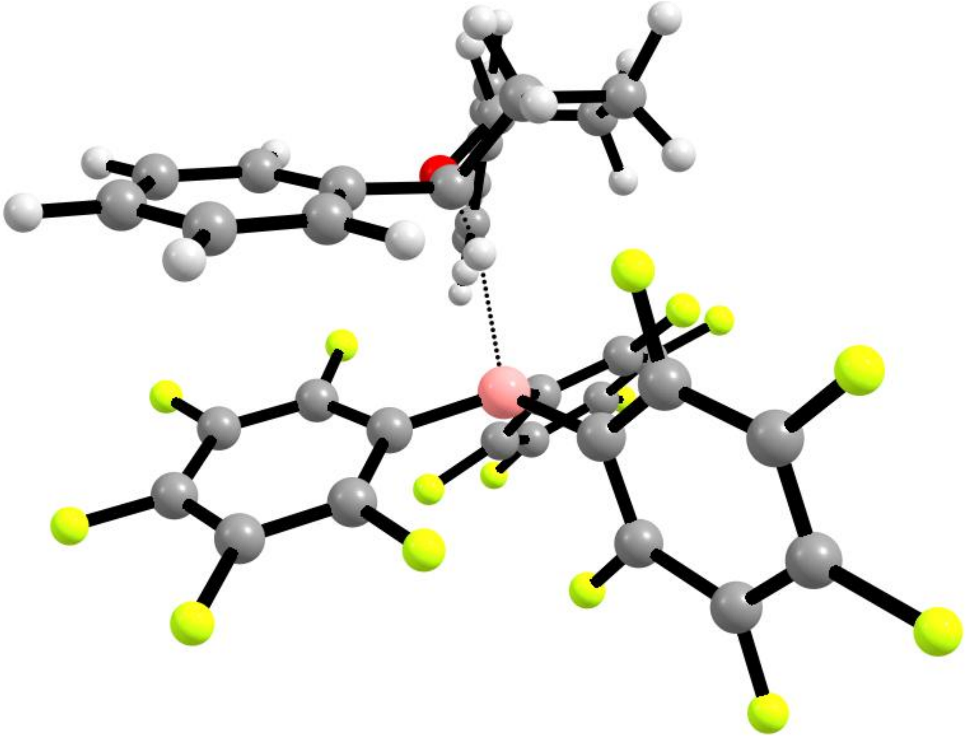

7.4. Cartesian coordinates of the calculated structures

Cartesian coordinates of the optimized geometries (ωB97X-D3/def2-TZVP/CPCM(diethyl ether)) are provided below in standard XYZ format (units are in Å). The first line contains the molecule name, the second line reports the solution-phase free energy at 353.15 K (G_{sol}, in Eh). For transition states, the imaginary frequency is provided in the third line ($\nu^{\ddagger}$ in cm^{-1})

H_2

G = –1.17988508

H	0.000000000000	0.000000000000	-0.002454000000
H	0.000000000000	0.000000000000	0.742454000000

B3

G = –2631.50464525

F	2.280371000000	1.248311000000	1.586932000000
C	2.425221000000	0.133439000000	0.873315000000
C	1.366627000000	-0.365233000000	0.118744000000
B	-0.000638000000	0.393873000000	-0.000900000000
C	-1.365804000000	-0.368977000000	-0.120590000000
C	-1.618828000000	-1.550938000000	0.572106000000
F	-0.676768000000	-2.090311000000	1.345615000000
C	-2.836733000000	-2.199678000000	0.531735000000
F	-3.038905000000	-3.315404000000	1.222822000000
C	-3.857864000000	-1.674194000000	-0.241480000000
F	-5.026856000000	-2.288105000000	-0.299210000000
C	-3.652184000000	-0.506143000000	-0.952676000000
F	-4.629161000000	-0.006661000000	-1.700634000000
C	-2.426490000000	0.127605000000	-0.873632000000
F	-2.285452000000	1.243694000000	-1.586106000000

C	-0.002863000000	1.970716000000	-0.000004000000
C	-0.477094000000	2.704011000000	1.080946000000
C	-0.484969000000	4.086650000000	1.104903000000
C	-0.006912000000	4.775170000000	0.001516000000
C	0.473175000000	4.089233000000	-1.102598000000
C	0.469279000000	2.706550000000	-1.080140000000
C	1.623554000000	-1.545644000000	-0.575145000000
F	0.683707000000	-2.086653000000	-1.350202000000
C	2.843193000000	-2.191083000000	-0.534317000000
F	3.049152000000	-3.305419000000	-1.226536000000
C	3.862117000000	-1.663722000000	0.240540000000
F	5.032698000000	-2.274555000000	0.298781000000
C	3.652573000000	-0.497045000000	0.952852000000
F	4.627449000000	0.004259000000	1.702332000000
H	0.843030000000	4.617919000000	-1.970995000000
H	-0.008506000000	5.857789000000	0.002093000000
H	-0.856424000000	4.613300000000	1.973854000000
Cl	1.039318000000	1.839913000000	-2.479050000000
Cl	-1.044576000000	1.834224000000	2.478930000000

1y

G = –768.45862233

C	2.945123000000	0.084982000000	-0.185120000000
C	3.610483000000	1.253220000000	-0.552981000000
C	3.653806000000	-0.927170000000	0.458226000000
C	4.958934000000	1.407231000000	-0.284029000000
C	5.005613000000	-0.774174000000	0.725950000000
C	5.659006000000	0.391992000000	0.356106000000
H	3.055088000000	2.036797000000	-1.052810000000

H	3.159262000000	-1.842998000000	0.755370000000
H	5.467624000000	2.318738000000	-0.573688000000
H	5.547814000000	-1.567712000000	1.225587000000
H	6.715343000000	0.511773000000	0.565974000000
C	1.488232000000	-0.041686000000	-0.505520000000
O	0.909499000000	0.841987000000	-1.101677000000
C	0.767576000000	-1.306554000000	-0.093318000000
H	1.237799000000	-2.142262000000	-0.619787000000
H	0.939750000000	-1.477824000000	0.971028000000
C	-0.715044000000	-1.270532000000	-0.396320000000
H	-1.142684000000	-2.272212000000	-0.290093000000
H	-0.890141000000	-0.969082000000	-1.430450000000
C	-1.488784000000	-0.349802000000	0.521874000000
O	-0.961176000000	0.157620000000	1.489003000000
C	-2.934808000000	-0.098788000000	0.226258000000
C	-3.602100000000	-0.743881000000	-0.812566000000
C	-3.632551000000	0.804993000000	1.025551000000
C	-4.945065000000	-0.490815000000	-1.046125000000
C	-4.971628000000	1.061488000000	0.789742000000
C	-5.630296000000	0.412437000000	-0.247216000000
H	-3.081866000000	-1.450450000000	-1.446413000000
H	-3.108933000000	1.302943000000	1.831875000000
H	-5.455829000000	-0.998423000000	-1.855314000000
H	-5.505898000000	1.767250000000	1.414242000000
H	-6.679089000000	0.612251000000	-0.432540000000

B3-H (borohydride)

G = –2632.23291939

F	0.524809000000	-1.880964000000	1.243433000000

C	1.550973000000	-1.403916000000	0.517057000000
C	1.381544000000	-0.309894000000	-0.315562000000
B	-0.022351000000	0.468521000000	-0.644107000000
C	-1.355074000000	-0.397203000000	-0.245306000000
C	-1.761518000000	-1.453773000000	-1.047866000000
F	-1.029273000000	-1.806792000000	-2.117677000000
C	-2.898717000000	-2.207501000000	-0.817915000000
F	-3.234102000000	-3.213442000000	-1.631910000000
C	-3.696985000000	-1.915071000000	0.271299000000
F	-4.798523000000	-2.627266000000	0.514086000000
C	-3.333623000000	-0.878602000000	1.106828000000
F	-4.091209000000	-0.590603000000	2.169878000000
C	-2.183217000000	-0.155052000000	0.838142000000
F	-1.892196000000	0.814899000000	1.721243000000
C	-0.098286000000	2.022583000000	-0.124832000000
C	0.557693000000	2.584908000000	0.976016000000
C	0.519795000000	3.929190000000	1.316168000000
C	-0.230961000000	4.802262000000	0.552243000000
C	-0.945322000000	4.310209000000	-0.523946000000
C	-0.868791000000	2.959890000000	-0.827704000000
C	2.539866000000	0.096267000000	-0.962766000000
F	2.509457000000	1.156839000000	-1.787445000000
C	3.770489000000	-0.516173000000	-0.808685000000
F	4.848579000000	-0.069716000000	-1.460632000000
C	3.882806000000	-1.607397000000	0.031222000000
F	5.057801000000	-2.217571000000	0.195539000000
C	2.760113000000	-2.054746000000	0.698265000000
F	2.855960000000	-3.103387000000	1.522098000000
H	-1.562644000000	4.962527000000	-1.127604000000
H	-0.271640000000	5.854885000000	0.803297000000
H	1.065843000000	4.278691000000	2.182633000000

Cl	-1.862433000000	2.455970000000	-2.181393000000
Cl	1.463862000000	1.582549000000	2.093336000000
H	-0.029105000000	0.532690000000	-1.854730000000

2y-oxonium

G = –693.61075337

C	-0.831366000000	-2.084084000000	0.613945000000
C	0.693038000000	-2.124903000000	0.530756000000
C	1.023548000000	-0.788696000000	-0.028511000000
O	0.013534000000	-0.214560000000	-0.545178000000
C	-1.220645000000	-1.058987000000	-0.442883000000
H	-1.291644000000	-3.047652000000	0.410253000000
H	1.054271000000	-2.866615000000	-0.191098000000
H	-1.311606000000	-1.506243000000	-1.432790000000
H	-1.149668000000	-1.750289000000	1.601153000000
C	2.298260000000	-0.138846000000	-0.060749000000
C	2.433437000000	1.123060000000	-0.660658000000
C	3.410931000000	-0.772148000000	0.508934000000
C	3.667067000000	1.735901000000	-0.685216000000
C	4.642166000000	-0.149960000000	0.476807000000
C	4.767284000000	1.100029000000	-0.117014000000
H	1.570989000000	1.606658000000	-1.100460000000
H	3.311790000000	-1.744914000000	0.973063000000
H	3.779288000000	2.708998000000	-1.145138000000
H	5.505159000000	-0.632846000000	0.916022000000
H	5.734806000000	1.587133000000	-0.138052000000
H	1.193507000000	-2.315959000000	1.477316000000
C	-2.387728000000	-0.169616000000	-0.152504000000
C	-3.469561000000	-0.149715000000	-1.022744000000

C	-2.407357000000	0.627918000000	0.988969000000
C	-4.570863000000	0.649708000000	-0.749329000000
C	-3.498964000000	1.436602000000	1.253174000000
C	-4.584930000000	1.444279000000	0.386252000000
H	-3.453879000000	-0.763400000000	-1.916572000000
H	-1.564107000000	0.626852000000	1.671229000000
H	-5.415109000000	0.654586000000	-1.427704000000
H	-3.506246000000	2.059422000000	2.139255000000
H	-5.441406000000	2.072773000000	0.598070000000

TS*cis* (for 2y with B3)

G = –3325.83548930

$\nu^{\ddagger}$ = –722.22

F	0.862910000000	-1.792902000000	-1.105342000000
C	0.370574000000	-0.965578000000	-2.036368000000
C	0.406125000000	0.413794000000	-1.849363000000
B	0.955233000000	1.075274000000	-0.472758000000
C	2.292875000000	0.404513000000	0.212268000000
C	2.559178000000	0.551226000000	1.582464000000
C	3.635761000000	-0.005895000000	2.252537000000
C	4.563940000000	-0.738642000000	1.541541000000
C	4.412641000000	-0.859066000000	0.175377000000
C	3.318782000000	-0.287351000000	-0.459375000000
C	1.125365000000	2.704856000000	-0.480765000000
C	2.120021000000	3.273480000000	-1.267039000000
C	2.387008000000	4.627609000000	-1.326477000000
C	1.634265000000	5.501555000000	-0.565261000000
C	0.633832000000	4.991380000000	0.234583000000
C	0.405274000000	3.625010000000	0.259817000000

C	-0.155852000000	1.139632000000	-2.891881000000
F	-0.239863000000	2.476169000000	-2.842629000000
C	-0.671009000000	0.564469000000	-4.042087000000
F	-1.200224000000	1.325060000000	-4.999648000000
C	-0.656754000000	-0.806710000000	-4.185607000000
F	-1.163371000000	-1.377403000000	-5.272943000000
C	-0.131917000000	-1.578660000000	-3.167366000000
F	-0.125084000000	-2.906741000000	-3.280177000000
H	-0.122908000000	0.800736000000	0.323943000000
H	-2.488990000000	2.540916000000	0.171624000000
C	-2.810293000000	1.788249000000	-0.533869000000
H	-3.986522000000	3.162531000000	-1.672387000000
C	-3.653707000000	2.136982000000	-1.573348000000
C	-4.071341000000	1.175936000000	-2.484268000000
H	-4.729433000000	1.452030000000	-3.299239000000
C	-3.651331000000	-0.139234000000	-2.347503000000
H	-3.981588000000	-0.893400000000	-3.051110000000
C	-2.797916000000	-0.490912000000	-1.316187000000
H	-2.472141000000	-1.516331000000	-1.206352000000
C	-2.363056000000	0.474986000000	-0.410969000000
C	-1.477529000000	0.079878000000	0.700272000000
H	-2.729036000000	0.789951000000	2.255086000000
C	-1.670161000000	0.568149000000	2.120118000000
H	-1.113388000000	1.474404000000	2.323332000000
O	-1.109480000000	-1.176369000000	0.693528000000
C	-0.465359000000	-1.508506000000	1.960918000000
C	-1.211825000000	-0.631348000000	2.960843000000
H	-2.069133000000	-1.168414000000	3.365865000000
H	-0.563316000000	-0.343182000000	3.784980000000
H	0.572454000000	-1.187126000000	1.857241000000
C	-0.506263000000	-2.991847000000	2.161299000000

C	0.681598000000	-3.688562000000	2.337187000000
C	-1.714364000000	-3.682677000000	2.174523000000
C	0.666438000000	-5.061967000000	2.538675000000
C	-1.730144000000	-5.054491000000	2.363560000000
C	-0.539196000000	-5.745863000000	2.550714000000
H	1.624224000000	-3.151701000000	2.311711000000
H	-2.646164000000	-3.147388000000	2.028033000000
H	1.598319000000	-5.596829000000	2.676539000000
H	-2.673175000000	-5.587842000000	2.366393000000
H	-0.553760000000	-6.818699000000	2.701409000000
F	2.874503000000	2.490779000000	-2.047737000000
F	3.354010000000	5.097007000000	-2.113088000000
F	1.870836000000	6.808494000000	-0.603489000000
F	-0.104687000000	5.812714000000	0.981039000000
F	-0.589926000000	3.233854000000	1.073008000000
H	3.741316000000	0.146753000000	3.318161000000
H	5.410957000000	-1.190451000000	2.041820000000
H	5.145787000000	-1.391593000000	-0.415284000000
Cl	1.513035000000	1.517818000000	2.593122000000
Cl	3.368605000000	-0.469512000000	-2.196692000000

TS*trans* (for 2y with B3)

G = –3325.83217549

$\nu^{\ddagger}$ = –730.61

F	0.582284000000	-1.680422000000	1.413552000000
C	-0.669448000000	-1.931506000000	1.014359000000
C	-1.498464000000	-0.890733000000	0.605101000000
B	-0.960532000000	0.641408000000	0.522885000000
C	-2.111228000000	1.761516000000	0.187912000000

C	-3.083580000000	1.999321000000	1.152912000000
F	-3.090530000000	1.270490000000	2.276172000000
C	-4.086507000000	2.941336000000	1.033799000000
F	-4.988165000000	3.102806000000	2.000856000000
C	-4.155433000000	3.717326000000	-0.107783000000
F	-5.109784000000	4.631155000000	-0.248327000000
C	-3.214018000000	3.526289000000	-1.096288000000
F	-3.252644000000	4.268994000000	-2.202755000000
C	-2.226005000000	2.568999000000	-0.928993000000
F	-1.353011000000	2.480564000000	-1.946062000000
C	-0.029312000000	1.216630000000	1.749386000000
C	0.022858000000	0.734570000000	3.071168000000
C	0.879371000000	1.231208000000	4.043952000000
C	1.715398000000	2.290996000000	3.756900000000
C	1.647767000000	2.873318000000	2.508186000000
C	0.784146000000	2.346508000000	1.561903000000
C	-2.759137000000	-1.307339000000	0.194368000000
F	-3.664133000000	-0.439700000000	-0.278404000000
C	-3.184698000000	-2.625986000000	0.226668000000
F	-4.408800000000	-2.950503000000	-0.188312000000
C	-2.328129000000	-3.611574000000	0.667724000000
F	-2.712459000000	-4.882886000000	0.688447000000
C	-1.052940000000	-3.256879000000	1.062478000000
F	-0.202742000000	-4.195884000000	1.477458000000
H	2.254139000000	3.733617000000	2.259078000000
H	2.392993000000	2.675640000000	4.508403000000
H	0.872305000000	0.785280000000	5.029371000000
Cl	0.751521000000	3.236582000000	0.060509000000
Cl	-1.042406000000	-0.513002000000	3.671439000000
H	-0.132301000000	0.513520000000	-0.568891000000
H	0.248236000000	-2.907106000000	-1.694176000000

C	-0.540958000000	-2.394531000000	-2.226464000000
H	-1.585337000000	-4.195363000000	-2.714884000000
C	-1.568413000000	-3.116518000000	-2.809077000000
C	-2.574850000000	-2.459317000000	-3.501135000000
H	-3.384079000000	-3.023431000000	-3.948802000000
C	-2.540047000000	-1.077117000000	-3.627751000000
H	-3.318290000000	-0.560575000000	-4.175433000000
C	-1.513197000000	-0.351980000000	-3.050640000000
H	-1.494203000000	0.722220000000	-3.155299000000
C	-0.515908000000	-1.004967000000	-2.330743000000
C	0.613608000000	-0.256413000000	-1.737320000000
H	0.727148000000	1.812934000000	-2.421782000000
C	1.298379000000	0.892175000000	-2.436764000000
H	1.419467000000	0.581596000000	-3.479321000000
O	1.506201000000	-1.002599000000	-1.127522000000
C	2.868918000000	-0.483210000000	-1.308365000000
C	2.640654000000	0.961014000000	-1.719615000000
H	2.580747000000	1.599389000000	-0.840083000000
H	3.443523000000	1.322475000000	-2.358562000000
H	3.269047000000	-1.061250000000	-2.144823000000
C	3.703734000000	-0.736937000000	-0.088917000000
C	3.319056000000	-0.267798000000	1.162450000000
C	4.900755000000	-1.430308000000	-0.215841000000
C	4.117108000000	-0.494657000000	2.270608000000
C	5.709838000000	-1.646803000000	0.891349000000
C	5.317227000000	-1.181476000000	2.136759000000
H	2.382462000000	0.262655000000	1.275247000000
H	5.204272000000	-1.803858000000	-1.187868000000
H	3.798465000000	-0.133268000000	3.241030000000
H	6.643159000000	-2.185570000000	0.780546000000
H	5.942661000000	-1.356807000000	3.003958000000

cis-**2y**

G = –694.39267811

H	1.363270000000	2.304477000000	0.967608000000
H	1.069157000000	-1.049400000000	-0.690060000000
C	2.059860000000	-0.721913000000	-0.397700000000
H	2.995240000000	-2.557948000000	-0.982913000000
C	3.143012000000	-1.571312000000	-0.559318000000
C	4.415381000000	-1.162107000000	-0.180145000000
H	5.261199000000	-1.827716000000	-0.305327000000
C	4.594688000000	0.100135000000	0.364998000000
H	5.581983000000	0.425289000000	0.671577000000
C	3.506560000000	0.947204000000	0.528266000000
H	3.648503000000	1.930843000000	0.963842000000
C	2.231762000000	0.548416000000	0.145661000000
C	1.072520000000	1.506300000000	0.277129000000
H	1.336607000000	1.956096000000	-1.858440000000
C	0.607032000000	2.116181000000	-1.065963000000
H	0.462202000000	3.191484000000	-0.952167000000
O	-0.062213000000	0.829620000000	0.817734000000
C	-1.230795000000	1.178426000000	0.091792000000
C	-0.725962000000	1.423754000000	-1.327606000000
H	-0.585624000000	0.464024000000	-1.830567000000
H	-1.416064000000	2.024648000000	-1.919235000000
H	-1.644824000000	2.113623000000	0.495401000000
C	-2.274698000000	0.099473000000	0.208042000000
C	-3.605501000000	0.413146000000	-0.051901000000
C	-1.941149000000	-1.211961000000	0.524660000000
C	-4.584861000000	-0.567061000000	-0.008531000000
C	-2.921814000000	-2.193372000000	0.575350000000
C	-4.245071000000	-1.876110000000	0.305946000000

H	-3.876582000000	1.437253000000	-0.287875000000
H	-0.910249000000	-1.459392000000	0.743751000000
H	-5.616809000000	-0.308165000000	-0.214957000000
H	-2.649618000000	-3.211394000000	0.829004000000
H	-5.009527000000	-2.642819000000	0.348181000000

***trans*-2y**

G = –694.39312778

H	-0.897814000000	-1.752447000000	0.367219000000
H	-1.846203000000	1.703457000000	-0.573206000000
C	-2.656379000000	0.988333000000	-0.494364000000
H	-4.126028000000	2.335039000000	-1.279050000000
C	-3.937235000000	1.339846000000	-0.893413000000
C	-4.975200000000	0.421766000000	-0.802480000000
H	-5.975615000000	0.697681000000	-1.114150000000
C	-4.721984000000	-0.852837000000	-0.316835000000
H	-5.523909000000	-1.578546000000	-0.247851000000
C	-3.437538000000	-1.204674000000	0.073948000000
H	-3.240523000000	-2.206506000000	0.441730000000
C	-2.396168000000	-0.287165000000	-0.004735000000
C	-1.024400000000	-0.669629000000	0.489081000000
H	-1.127526000000	-1.040733000000	2.641993000000
C	-0.759885000000	-0.285977000000	1.947503000000
H	-1.253457000000	0.662241000000	2.174975000000
O	-0.004368000000	-0.013760000000	-0.264055000000
C	0.997502000000	0.529686000000	0.594686000000
C	0.751780000000	-0.120653000000	1.956736000000
H	1.249195000000	-1.092976000000	1.996520000000
H	1.124785000000	0.492711000000	2.776449000000

H	0.836027000000	1.612247000000	0.674763000000
C	2.376165000000	0.290324000000	0.035668000000
C	2.685675000000	-0.886190000000	-0.638848000000
C	3.375656000000	1.235263000000	0.235956000000
C	3.973145000000	-1.113437000000	-1.100946000000
C	4.667135000000	1.006879000000	-0.218583000000
C	4.969122000000	-0.168677000000	-0.890099000000
H	1.908648000000	-1.621831000000	-0.810177000000
H	3.140337000000	2.161088000000	0.750918000000
H	4.200367000000	-2.031325000000	-1.630645000000
H	5.435803000000	1.753126000000	-0.054735000000
H	5.974693000000	-0.346742000000	-1.252376000000

H_2O

G = –694.39312778

O	-2.555652000000	3.891492000000	-0.589380000000
H	-2.555652000000	4.652175000000	-0.004104000000
H	-2.555652000000	3.130809000000	-0.004104000000

TS*cis* (for 2y with B1)

G = –2902.90836978

$\nu^{\ddagger}$ = –539.49

F	1.217347000000	-1.882901000000	-0.850934000000
C	0.657491000000	-1.193607000000	-1.855128000000
C	0.508965000000	0.187263000000	-1.770267000000
B	0.932201000000	1.023212000000	-0.451132000000
C	2.255211000000	0.503533000000	0.354746000000
C	2.406268000000	0.739975000000	1.712645000000

F	1.425411000000	1.353003000000	2.394825000000
C	3.521451000000	0.381653000000	2.446827000000
F	3.583056000000	0.624569000000	3.754878000000
C	4.581157000000	-0.230026000000	1.806296000000
F	5.665874000000	-0.582569000000	2.485915000000
C	4.498608000000	-0.460275000000	0.446855000000
F	5.517101000000	-1.038456000000	-0.186371000000
C	3.358535000000	-0.089747000000	-0.246647000000
F	3.376169000000	-0.337007000000	-1.560775000000
C	1.071594000000	2.638671000000	-0.616500000000
C	1.945939000000	3.158378000000	-1.562346000000
C	2.185814000000	4.507878000000	-1.730757000000
C	1.536561000000	5.418365000000	-0.916441000000
C	0.667370000000	4.952627000000	0.048001000000
C	0.460788000000	3.588697000000	0.180741000000
C	-0.120674000000	0.757465000000	-2.870267000000
F	-0.399327000000	2.066429000000	-2.901625000000
C	-0.528887000000	0.040553000000	-3.982179000000
F	-1.134759000000	0.654290000000	-4.997192000000
C	-0.338528000000	-1.325123000000	-4.020884000000
F	-0.735398000000	-2.032700000000	-5.071424000000
C	0.257241000000	-1.948752000000	-2.940996000000
F	0.437214000000	-3.268186000000	-2.955066000000
H	-0.122481000000	0.817140000000	0.334472000000
H	-2.620018000000	2.551828000000	0.732784000000
C	-2.899012000000	1.939710000000	-0.113868000000
H	-4.179316000000	3.436097000000	-0.945898000000
C	-3.776557000000	2.437491000000	-1.059172000000
C	-4.142675000000	1.656398000000	-2.148120000000
H	-4.830969000000	2.048290000000	-2.887191000000
C	-3.635149000000	0.373086000000	-2.287264000000

H	-3.922263000000	-0.239188000000	-3.133074000000
C	-2.749707000000	-0.127403000000	-1.348321000000
H	-2.354521000000	-1.129398000000	-1.452899000000
C	-2.373732000000	0.656398000000	-0.259093000000
C	-1.453797000000	0.113630000000	0.752141000000
H	-2.526452000000	0.723270000000	2.469748000000
C	-1.489318000000	0.506231000000	2.211499000000
H	-0.905447000000	1.396525000000	2.411641000000
O	-1.142592000000	-1.149925000000	0.618389000000
C	-0.409776000000	-1.614233000000	1.797569000000
C	-0.966325000000	-0.747386000000	2.924583000000
H	-1.776536000000	-1.265416000000	3.436017000000
H	-0.191023000000	-0.520026000000	3.652552000000
H	0.637242000000	-1.378152000000	1.601434000000
C	-0.572360000000	-3.098426000000	1.919637000000
C	0.552663000000	-3.910928000000	1.945630000000
C	-1.836165000000	-3.674257000000	2.012333000000
C	0.421668000000	-5.286822000000	2.075207000000
C	-1.968729000000	-5.047536000000	2.129932000000
C	-0.838851000000	-5.855996000000	2.165770000000
H	1.536965000000	-3.463705000000	1.859174000000
H	-2.721158000000	-3.047759000000	1.984450000000
H	1.305830000000	-5.912365000000	2.096435000000
H	-2.955135000000	-5.490717000000	2.195513000000
H	-0.944527000000	-6.930023000000	2.261386000000
F	2.605552000000	2.326543000000	-2.379792000000
F	3.031183000000	4.940471000000	-2.664028000000
F	1.751223000000	6.721540000000	-1.059075000000
F	0.038586000000	5.813403000000	0.847425000000
F	-0.386115000000	3.224398000000	1.157282000000

TS$_{trans}$ (for 2y with B1)

G = –2902.90727344

$\nu^{\ddagger}$ = –548.09

F	2.813964000000	-1.681876000000	-0.803530000000
C	1.623777000000	-1.910586000000	-1.370201000000
C	0.628962000000	-0.942146000000	-1.371290000000
B	0.711410000000	0.547746000000	-0.722213000000
C	1.796338000000	0.818660000000	0.453659000000
C	2.104133000000	-0.127624000000	1.425306000000
F	1.489559000000	-1.316148000000	1.430857000000
C	3.007304000000	0.075226000000	2.451144000000
F	3.243880000000	-0.883580000000	3.345877000000
C	3.636524000000	1.297779000000	2.570509000000
F	4.489686000000	1.524243000000	3.562071000000
C	3.339578000000	2.290210000000	1.659446000000
F	3.909650000000	3.488254000000	1.777929000000
C	2.437780000000	2.039449000000	0.638757000000
F	2.202018000000	3.078282000000	-0.173244000000
C	0.769332000000	1.609529000000	-1.960214000000
C	1.895815000000	1.608782000000	-2.772535000000
C	2.052577000000	2.425984000000	-3.875236000000
C	1.039694000000	3.304215000000	-4.215358000000
C	-0.102818000000	3.339719000000	-3.442674000000
C	-0.212340000000	2.500186000000	-2.346348000000
C	-0.500565000000	-1.287173000000	-2.098814000000
F	-1.489182000000	-0.388794000000	-2.248143000000
C	-0.691128000000	-2.510755000000	-2.710734000000
F	-1.816989000000	-2.776635000000	-3.371227000000
C	0.306168000000	-3.462140000000	-2.630310000000
F	0.147632000000	-4.653124000000	-3.196231000000

C	1.475923000000	-3.152007000000	-1.965324000000
F	2.455971000000	-4.052583000000	-1.903319000000
H	-0.459816000000	0.699445000000	-0.089813000000
H	-0.104941000000	0.330184000000	3.460403000000
C	-0.121530000000	1.363728000000	3.140290000000
H	1.004713000000	2.065653000000	4.818313000000
C	0.495123000000	2.341755000000	3.903446000000
C	0.469035000000	3.665303000000	3.490497000000
H	0.960873000000	4.427126000000	4.083101000000
C	-0.185063000000	4.015848000000	2.315697000000
H	-0.205062000000	5.048548000000	1.991216000000
C	-0.803056000000	3.044237000000	1.551029000000
H	-1.294043000000	3.319382000000	0.626845000000
C	-0.767989000000	1.712018000000	1.958176000000
C	-1.445853000000	0.677634000000	1.155107000000
H	-2.733617000000	1.541975000000	-0.374365000000
C	-2.785054000000	0.916175000000	0.508448000000
H	-3.383720000000	1.440936000000	1.258484000000
O	-1.364865000000	-0.550633000000	1.595259000000
C	-2.535917000000	-1.356757000000	1.229964000000
C	-3.312911000000	-0.489424000000	0.245271000000
H	-3.110603000000	-0.791413000000	-0.778117000000
H	-4.384230000000	-0.560553000000	0.419396000000
H	-3.080948000000	-1.456800000000	2.172214000000
C	-2.104681000000	-2.718328000000	0.752305000000
C	-2.905860000000	-3.430915000000	-0.133300000000
C	-0.936890000000	-3.302524000000	1.229750000000
C	-2.537575000000	-4.703657000000	-0.545378000000
C	-0.561826000000	-4.567391000000	0.805521000000
C	-1.361460000000	-5.273130000000	-0.082039000000
H	-3.821533000000	-2.994919000000	-0.515150000000

H	-0.309029000000	-2.760017000000	1.922280000000
H	-3.167840000000	-5.245523000000	-1.240091000000
H	0.361038000000	-5.001349000000	1.171489000000
H	-1.066002000000	-6.260776000000	-0.414749000000
F	2.916492000000	0.788467000000	-2.483217000000
F	3.163468000000	2.380374000000	-4.607988000000
F	1.165017000000	4.101018000000	-5.271382000000
F	-1.091187000000	4.176583000000	-3.756953000000
F	-1.357315000000	2.604628000000	-1.650562000000

TS*cis* (for 2af with B1)

G = –2942.19672560

$\nu^{\ddagger}$ = –663.05

F	1.562463000000	-2.129230000000	2.379498000000
C	2.526501000000	-2.318832000000	1.467426000000
C	2.521496000000	-1.615616000000	0.279059000000
B	1.488095000000	-0.447964000000	-0.202524000000
C	2.326935000000	0.943681000000	-0.188378000000
C	2.812803000000	1.404943000000	1.028758000000
F	2.596992000000	0.686251000000	2.140939000000
C	3.537285000000	2.570827000000	1.182950000000
F	3.951124000000	2.965475000000	2.385988000000
C	3.843355000000	3.324045000000	0.065861000000
F	4.537189000000	4.450453000000	0.183294000000
C	3.440872000000	2.876008000000	-1.177012000000
F	3.768620000000	3.570783000000	-2.265320000000
C	2.713966000000	1.701714000000	-1.284678000000
F	2.430265000000	1.311258000000	-2.532043000000
C	0.597806000000	-0.824825000000	-1.505986000000

C	-0.129514000000	0.120148000000	-2.222621000000
C	-0.949395000000	-0.178953000000	-3.295353000000
C	-1.081826000000	-1.490305000000	-3.704477000000
C	-0.394536000000	-2.474674000000	-3.022248000000
C	0.411215000000	-2.132211000000	-1.949834000000
C	3.582250000000	-1.912470000000	-0.567552000000
F	3.671310000000	-1.294948000000	-1.755120000000
C	4.570903000000	-2.831639000000	-0.276206000000
F	5.556876000000	-3.071009000000	-1.138601000000
C	4.527952000000	-3.510752000000	0.927585000000
F	5.466524000000	-4.399998000000	1.233730000000
C	3.495748000000	-3.250015000000	1.805178000000
F	3.438046000000	-3.892475000000	2.971421000000
H	0.527622000000	-0.242108000000	0.746098000000
F	-0.086802000000	1.411298000000	-1.881242000000
F	-1.626311000000	0.781385000000	-3.919202000000
F	-1.874289000000	-1.803607000000	-4.720454000000
F	-0.528889000000	-3.747632000000	-3.388295000000
F	0.988906000000	-3.167196000000	-1.326882000000
C	-1.539917000000	-1.929544000000	2.120965000000
C	-0.938310000000	-0.594475000000	2.516432000000
C	-0.775915000000	0.355417000000	1.358848000000
C	-2.378441000000	-0.913730000000	0.037921000000
C	-2.781100000000	-1.675815000000	1.282546000000
H	-1.778665000000	-2.498001000000	3.019680000000
H	-0.817821000000	-2.516223000000	1.546903000000
H	0.005517000000	-0.700991000000	3.039933000000
H	-1.625015000000	-0.073968000000	3.194852000000
H	-1.703042000000	-1.538613000000	-0.547394000000
H	-3.250073000000	-2.607360000000	0.962465000000
H	-3.525421000000	-1.104101000000	1.844729000000

O	-1.632764000000	0.292715000000	0.366744000000
C	-3.532183000000	-0.485572000000	-0.827388000000
C	-4.160185000000	0.741466000000	-0.654785000000
C	-3.999397000000	-1.361080000000	-1.801240000000
C	-5.239968000000	1.089747000000	-1.453493000000
C	-5.081473000000	-1.015469000000	-2.595293000000
C	-5.703271000000	0.213419000000	-2.423942000000
H	-3.799558000000	1.433401000000	0.096646000000
H	-3.506721000000	-2.317393000000	-1.944358000000
H	-5.718487000000	2.052492000000	-1.319046000000
H	-5.432332000000	-1.703841000000	-3.354623000000
H	-6.543826000000	0.489025000000	-3.049401000000
C	-0.352052000000	1.751987000000	1.649308000000
C	0.249093000000	2.069894000000	2.865629000000
C	-0.600783000000	2.768063000000	0.727879000000
C	0.618630000000	3.374500000000	3.142995000000
C	-0.223436000000	4.069967000000	1.004690000000
C	0.394124000000	4.375363000000	2.209274000000
H	0.446104000000	1.304015000000	3.602160000000
H	-1.092789000000	2.538052000000	-0.204282000000
H	1.091758000000	3.606235000000	4.088845000000
H	-0.414662000000	4.848300000000	0.276427000000
H	0.692505000000	5.394416000000	2.424160000000

TS$_{trans}$ (for 2af with B1)

G = –2942.19023092

$\nu^{‡}$ = –680.58

F	4.114076000000	-0.948301000000	0.522800000000
C	3.275010000000	-1.525074000000	-0.347068000000

C	2.039935000000	-0.962999000000	-0.649422000000
B	1.451899000000	0.391115000000	0.015127000000
C	2.493796000000	1.176035000000	0.990648000000
C	2.496367000000	1.187720000000	2.370925000000
F	1.593120000000	0.470920000000	3.059494000000
C	3.400131000000	1.908468000000	3.133270000000
F	3.339541000000	1.880039000000	4.464076000000
C	4.371691000000	2.660703000000	2.504612000000
F	5.250191000000	3.358422000000	3.216466000000
C	4.421686000000	2.672816000000	1.122840000000
F	5.363356000000	3.378632000000	0.499984000000
C	3.495874000000	1.939108000000	0.406356000000
F	3.621037000000	1.960641000000	-0.929179000000
C	0.759289000000	1.481695000000	-0.977360000000
C	0.805905000000	1.494503000000	-2.367180000000
C	0.184268000000	2.458587000000	-3.143397000000
C	-0.498578000000	3.495523000000	-2.538295000000
C	-0.516513000000	3.568018000000	-1.159872000000
C	0.119697000000	2.584289000000	-0.425599000000
C	1.279572000000	-1.717457000000	-1.537044000000
F	0.052355000000	-1.305065000000	-1.877834000000
C	1.690068000000	-2.911727000000	-2.093141000000
F	0.895363000000	-3.585340000000	-2.922675000000
C	2.932104000000	-3.420520000000	-1.764372000000
F	3.343343000000	-4.574636000000	-2.274282000000
C	3.729787000000	-2.717138000000	-0.886770000000
F	4.924141000000	-3.199946000000	-0.546705000000
H	0.401795000000	-0.076983000000	0.776068000000
F	1.502525000000	0.578777000000	-3.047849000000
F	0.246278000000	2.399278000000	-4.472079000000
F	-1.110629000000	4.417189000000	-3.270432000000

F	-1.138935000000	4.575505000000	-0.551843000000
F	0.107581000000	2.749860000000	0.905529000000
C	-1.671179000000	0.942589000000	2.684952000000
C	-1.162456000000	-0.487970000000	2.574458000000
C	-0.865209000000	-0.946116000000	1.179808000000
C	-2.927576000000	0.133184000000	0.630553000000
C	-2.567318000000	1.326873000000	1.498502000000
H	-0.820840000000	1.619032000000	2.738999000000
H	-2.213508000000	1.036669000000	3.625337000000
H	-1.948993000000	-1.189854000000	2.884738000000
H	-0.317841000000	-0.683377000000	3.226090000000
H	-3.564932000000	-0.559002000000	1.188682000000
H	-3.503557000000	1.771266000000	1.837886000000
H	-2.083886000000	2.069634000000	0.867480000000
O	-1.749236000000	-0.635117000000	0.260438000000
C	-3.618592000000	0.538299000000	-0.642389000000
C	-2.898823000000	0.831706000000	-1.793809000000
C	-4.999849000000	0.691930000000	-0.644044000000
C	-3.551963000000	1.282347000000	-2.930990000000
C	-5.653050000000	1.147781000000	-1.779515000000
C	-4.929643000000	1.446661000000	-2.925589000000
H	-1.825037000000	0.695835000000	-1.804245000000
H	-5.569662000000	0.455006000000	0.248394000000
H	-2.978359000000	1.508307000000	-3.822435000000
H	-6.730026000000	1.265493000000	-1.769513000000
H	-5.438624000000	1.802232000000	-3.813368000000
C	-0.280583000000	-2.302267000000	1.032620000000
C	-0.934839000000	-3.242541000000	0.242561000000
C	0.858575000000	-2.668882000000	1.745125000000
C	-0.450213000000	-4.538453000000	0.164147000000
C	1.346070000000	-3.959159000000	1.654171000000

C	0.692124000000	-4.896331000000	0.863573000000
H	-1.820457000000	-2.956749000000	-0.308549000000
H	1.385725000000	-1.935509000000	2.343504000000
H	-0.964024000000	-5.266281000000	-0.451316000000
H	2.242826000000	-4.233750000000	2.195507000000
H	1.077827000000	-5.906145000000	0.792445000000

***cis*-2af**

G = –733.68795431

C	0.000215000000	-2.915259000000	0.642210000000
C	1.250080000000	-2.045909000000	0.719065000000
C	1.182448000000	-0.920708000000	-0.312634000000
C	-1.182324000000	-0.920624000000	-0.312188000000
C	-1.249660000000	-2.045941000000	0.719439000000
H	0.000345000000	-3.658566000000	1.442001000000
H	0.000096000000	-3.466373000000	-0.304797000000
H	2.152751000000	-2.636131000000	0.545326000000
H	1.338738000000	-1.594040000000	1.712485000000
H	-1.165027000000	-1.369372000000	-1.317150000000
H	-2.152344000000	-2.636185000000	0.545890000000
H	-1.338003000000	-1.594154000000	1.712926000000
O	0.000103000000	-0.155977000000	-0.142481000000
C	-2.372770000000	-0.001330000000	-0.220085000000
C	-2.369215000000	1.106420000000	0.620927000000
C	-3.520418000000	-0.289093000000	-0.950758000000
C	-3.494862000000	1.911055000000	0.726873000000
C	-4.648642000000	0.510376000000	-0.842007000000
C	-4.638494000000	1.614910000000	-0.001836000000
H	-1.477465000000	1.342201000000	1.188668000000

H	-3.529526000000	-1.147169000000	-1.615126000000
H	-3.478115000000	2.775040000000	1.381138000000
H	-5.534113000000	0.274340000000	-1.420546000000
H	-5.516534000000	2.244480000000	0.080888000000
C	2.372978000000	-0.001482000000	-0.221099000000
C	2.369723000000	1.106508000000	0.619596000000
C	3.520434000000	-0.289590000000	-0.951934000000
C	3.495488000000	1.911045000000	0.725066000000
C	4.648773000000	0.509776000000	-0.843654000000
C	4.638930000000	1.614558000000	-0.003804000000
H	1.478125000000	1.342552000000	1.187465000000
H	3.529306000000	-1.147872000000	-1.616040000000
H	3.478983000000	2.775219000000	1.379085000000
H	5.534094000000	0.273465000000	-1.422310000000
H	5.517063000000	2.244049000000	0.078545000000
H	1.164611000000	-1.369532000000	-1.317551000000

***trans*-2af**

G = –733.68381307

C	0.633473000000	2.503873000000	0.941297000000
C	1.304897000000	2.474430000000	-0.427859000000
C	1.276394000000	1.074420000000	-1.035886000000
C	-0.696742000000	0.518475000000	0.194364000000
C	-0.763621000000	1.901065000000	0.839021000000
H	0.582637000000	3.528215000000	1.315895000000
H	1.225004000000	1.930561000000	1.662326000000
H	2.326706000000	2.853615000000	-0.384591000000
H	0.757202000000	3.130786000000	-1.109844000000
H	-0.112062000000	-0.142008000000	0.849003000000

H	-1.230609000000	1.814060000000	1.822888000000
H	-1.407193000000	2.539430000000	0.224557000000
O	-0.062082000000	0.589566000000	-1.077906000000
C	-2.062027000000	-0.089104000000	0.004421000000
C	-2.860482000000	0.268658000000	-1.077643000000
C	-2.559608000000	-0.988481000000	0.939892000000
C	-4.132193000000	-0.265573000000	-1.219937000000
C	-3.834839000000	-1.519619000000	0.803664000000
C	-4.624420000000	-1.160071000000	-0.278633000000
H	-2.478075000000	0.964034000000	-1.815134000000
H	-1.940473000000	-1.279341000000	1.782273000000
H	-4.742644000000	0.016842000000	-2.069866000000
H	-4.207629000000	-2.221914000000	1.540082000000
H	-5.617762000000	-1.578343000000	-0.390708000000
C	2.211211000000	0.050012000000	-0.397735000000
C	1.963287000000	-1.306581000000	-0.602402000000
C	3.335793000000	0.410070000000	0.336505000000
C	2.809943000000	-2.273227000000	-0.085016000000
C	4.188697000000	-0.556462000000	0.855193000000
C	3.929253000000	-1.901650000000	0.648274000000
H	1.089093000000	-1.602865000000	-1.169835000000
H	3.560617000000	1.453736000000	0.517133000000
H	2.595938000000	-3.321823000000	-0.256548000000
H	5.057416000000	-0.252005000000	1.427274000000
H	4.593743000000	-2.655457000000	1.053595000000
H	1.558854000000	1.150117000000	-2.089695000000

B1 (gas phase)

H = –2208.45423327

F	-2.410510000000	0.394084000000	1.540882000000
C	-2.029345000000	1.407823000000	0.764351000000
C	-0.874042000000	1.303910000000	-0.004894000000
B	0.002683000000	0.004565000000	-0.010634000000
C	-0.686941000000	-1.403395000000	-0.010109000000
C	-0.215812000000	-2.453697000000	0.771639000000
F	0.841875000000	-2.276520000000	1.563138000000
C	-0.819456000000	-3.696471000000	0.799608000000
F	-0.350968000000	-4.663897000000	1.575307000000
C	-1.929457000000	-3.926532000000	0.003776000000
F	-2.513197000000	-5.110634000000	0.010700000000
C	-2.427359000000	-2.913575000000	-0.799291000000
F	-3.484102000000	-3.138947000000	-1.567106000000
C	-1.810132000000	-1.677178000000	-0.784713000000
F	-2.318399000000	-0.736928000000	-1.580992000000
C	1.566528000000	0.108521000000	-0.010224000000
C	2.362839000000	-0.745385000000	-0.768123000000
C	3.742458000000	-0.667300000000	-0.783422000000
C	4.374069000000	0.284431000000	0.000085000000
C	3.622705000000	1.149737000000	0.778136000000
C	2.244237000000	1.055583000000	0.752193000000
C	-0.545183000000	2.423486000000	-0.763773000000
F	0.533705000000	2.408497000000	-1.545711000000
C	-1.312573000000	3.572834000000	-0.774917000000
F	-0.973463000000	4.610881000000	-1.526216000000
C	-2.449339000000	3.634230000000	0.014325000000
F	-3.188850000000	4.727674000000	0.022732000000
C	-2.809729000000	2.547914000000	0.794259000000
F	-3.892957000000	2.611260000000	1.555578000000
F	1.565475000000	1.902440000000	1.525458000000
F	4.229470000000	2.054297000000	1.533537000000

F	5.691531000000	0.366514000000	0.005609000000
F	4.463210000000	-1.488081000000	-1.534316000000
F	1.801185000000	-1.669969000000	-1.546288000000

B2 (gas phase)

H = –2432.89802416

F	2.253958000000	1.028882000000	1.633825000000
C	2.410898000000	-0.075114000000	0.904841000000
C	1.366228000000	-0.556260000000	0.120555000000
B	-0.000349000000	0.207479000000	-0.000520000000
C	-1.365890000000	-0.558115000000	-0.121289000000
C	-2.411752000000	-0.078059000000	-0.904686000000
F	-2.256874000000	1.026501000000	-1.633243000000
C	-3.629262000000	-0.731594000000	-0.983522000000
F	-4.589360000000	-0.236823000000	-1.763642000000
C	-3.855567000000	-1.883397000000	-0.262103000000
C	-2.840032000000	-2.374165000000	0.531873000000
F	-3.033341000000	-3.481394000000	1.247303000000
C	-1.619982000000	-1.727498000000	0.591440000000
F	-0.682965000000	-2.242195000000	1.388860000000
C	-0.001376000000	1.783977000000	-0.000305000000
C	0.486376000000	2.517284000000	-1.075047000000
C	0.490963000000	3.899937000000	-1.096290000000
C	-0.003019000000	4.586500000000	0.000090000000
C	-0.496203000000	3.899043000000	1.096267000000
C	-0.490009000000	2.516404000000	1.074634000000
C	1.622389000000	-1.724929000000	-0.592593000000
F	0.686569000000	-2.240509000000	-1.390856000000
C	2.843299000000	-2.369919000000	-0.532541000000

F	3.038653000000	-3.476511000000	-1.248406000000
C	3.857627000000	-1.878137000000	0.262358000000
C	3.629258000000	-0.727004000000	0.984190000000
F	4.588144000000	-0.231294000000	1.765205000000
H	-0.879224000000	4.423638000000	1.961094000000
H	-0.003655000000	5.669110000000	0.000246000000
H	0.873346000000	4.425233000000	-1.960974000000
Cl	-1.078806000000	1.650881000000	2.462945000000
Cl	1.076143000000	1.652830000000	-2.463622000000
H	-4.808632000000	-2.391608000000	-0.316267000000
H	4.811347000000	-2.385074000000	0.316925000000

B3 (gas phase)

H = –2631.40044726

F	2.282472000000	1.249219000000	1.581001000000
C	2.425724000000	0.132816000000	0.872601000000
C	1.366198000000	-0.367188000000	0.119439000000
B	-0.000041000000	0.393012000000	-0.000334000000
C	-1.366037000000	-0.367646000000	-0.119894000000
C	-2.425936000000	0.132078000000	-0.872725000000
F	-2.283278000000	1.248632000000	-1.581003000000
C	-3.652431000000	-0.501925000000	-0.953865000000
F	-4.628560000000	-0.001986000000	-1.699735000000
C	-3.857687000000	-1.672270000000	-0.244425000000
F	-5.025699000000	-2.285853000000	-0.304117000000
C	-2.836841000000	-2.200091000000	0.529483000000
F	-3.039880000000	-3.315653000000	1.217390000000
C	-1.618594000000	-1.550727000000	0.571710000000
F	-0.677085000000	-2.089425000000	1.344310000000

C	-0.000323000000	1.969933000000	-0.000020000000
C	-0.474867000000	2.702578000000	1.080914000000
C	-0.480405000000	4.085176000000	1.102962000000
C	-0.000905000000	4.772543000000	0.000551000000
C	0.478876000000	4.085826000000	-1.102146000000
C	0.473910000000	2.703216000000	-1.080663000000
C	1.619346000000	-1.550090000000	-0.572256000000
F	0.678214000000	-2.089023000000	-1.345154000000
C	2.837821000000	-2.199012000000	-0.529821000000
F	3.041441000000	-3.314409000000	-1.217824000000
C	3.858284000000	-1.670925000000	0.244410000000
F	5.026502000000	-2.284098000000	0.304317000000
C	3.652429000000	-0.500754000000	0.953963000000
F	4.628192000000	-0.000567000000	1.700144000000
H	0.850214000000	4.611430000000	-1.971430000000
H	-0.001132000000	5.855117000000	0.000774000000
H	-0.851980000000	4.610266000000	1.972454000000
Cl	1.044986000000	1.838048000000	-2.477011000000
Cl	-1.045626000000	1.836594000000	2.476882000000

H^- (gas phase)

H = –0.506971670816

H	0.000000000000	0.000000000000	0.000000000000

[B1-H]$^-$ (gas phase)

H = –2209.15880162

F	-1.751430000000	1.135313000000	-1.440704000000
C	-2.283137000000	0.168367000000	-0.676932000000

C	-1.546458000000	-0.421598000000	0.337253000000
B	-0.033385000000	-0.004978000000	0.807179000000
C	0.384639000000	1.517671000000	0.370040000000
C	1.147873000000	1.855306000000	-0.736685000000
F	1.556481000000	0.909824000000	-1.596422000000
C	1.518554000000	3.152609000000	-1.049811000000
F	2.256745000000	3.416291000000	-2.133550000000
C	1.108712000000	4.191400000000	-0.237014000000
F	1.450984000000	5.450808000000	-0.522860000000
C	0.334280000000	3.910566000000	0.871690000000
F	-0.072913000000	4.910526000000	1.661776000000
C	-0.008976000000	2.597251000000	1.147665000000
F	-0.775543000000	2.407914000000	2.230193000000
C	1.104988000000	-1.102755000000	0.377157000000
C	2.294962000000	-1.154234000000	1.090290000000
C	3.306669000000	-2.064742000000	0.838729000000
C	3.150408000000	-2.987601000000	-0.177769000000
C	1.988596000000	-2.973339000000	-0.923547000000
C	1.005004000000	-2.038817000000	-0.639548000000
C	-2.212761000000	-1.434411000000	1.014611000000
F	-1.595794000000	-2.100380000000	2.000546000000
C	-3.508319000000	-1.830143000000	0.731369000000
F	-4.097200000000	-2.812828000000	1.422126000000
C	-4.204325000000	-1.198368000000	-0.281970000000
F	-5.457145000000	-1.561373000000	-0.571517000000
C	-3.584595000000	-0.190373000000	-0.992269000000
F	-4.244685000000	0.421186000000	-1.981762000000
F	-0.075741000000	-2.074739000000	-1.434173000000
F	1.831966000000	-3.856010000000	-1.916195000000
F	4.111804000000	-3.878625000000	-0.437003000000
F	4.430377000000	-2.066494000000	1.564494000000

F	2.521280000000	-0.283155000000	2.083413000000
H	-0.053689000000	-0.014116000000	2.016889000000

[B2-H]$^{-}$ (gas phase)

H = –2433.58180395

F	0.577955000000	-2.064804000000	1.302032000000
C	1.593030000000	-1.571609000000	0.572073000000
C	1.398670000000	-0.487843000000	-0.271660000000
B	-0.025305000000	0.245407000000	-0.610876000000
C	-1.335888000000	-0.627938000000	-0.160290000000
C	-1.725920000000	-1.724796000000	-0.919399000000
F	-0.992134000000	-2.106640000000	-1.976474000000
C	-2.850426000000	-2.477796000000	-0.636401000000
F	-3.166604000000	-3.526862000000	-1.418009000000
C	-3.656486000000	-2.171297000000	0.439488000000
C	-3.290769000000	-1.093989000000	1.215619000000
F	-4.043231000000	-0.764864000000	2.281343000000
C	-2.159357000000	-0.351902000000	0.921035000000
F	-1.877128000000	0.660884000000	1.756472000000
C	-0.126892000000	1.818021000000	-0.155676000000
C	0.530323000000	2.438493000000	0.912354000000
C	0.467288000000	3.794968000000	1.194921000000
C	-0.310393000000	4.619200000000	0.405597000000
C	-1.025598000000	4.066950000000	-0.639486000000
C	-0.925815000000	2.707217000000	-0.888424000000
C	2.544216000000	-0.059427000000	-0.932399000000
F	2.481964000000	0.990852000000	-1.765965000000
C	3.780703000000	-0.655243000000	-0.769187000000
F	4.845503000000	-0.180141000000	-1.440979000000

C	3.941273000000	-1.734652000000	0.073933000000
C	2.823310000000	-2.183658000000	0.741868000000
F	2.934292000000	-3.229151000000	1.581737000000
H	-1.664001000000	4.677870000000	-1.264109000000
H	-0.371269000000	5.680912000000	0.612803000000
H	1.016309000000	4.187876000000	2.040588000000
Cl	-1.925109000000	2.128649000000	-2.203568000000
Cl	1.471911000000	1.505391000000	2.055164000000
H	-0.045492000000	0.259987000000	-1.820907000000
H	4.904064000000	-2.207515000000	0.205713000000
H	-4.535442000000	-2.757058000000	0.668287000000

[B3-H]$^-$ (gas phase)

H = –2632.08904127

F	0.526764000000	-1.921933000000	1.208199000000
C	1.552914000000	-1.419772000000	0.503007000000
C	1.381305000000	-0.313531000000	-0.313313000000
B	-0.026300000000	0.456627000000	-0.645112000000
C	-1.357634000000	-0.404307000000	-0.231287000000
C	-1.770741000000	-1.463152000000	-1.028474000000
F	-1.046919000000	-1.824123000000	-2.097163000000
C	-2.909911000000	-2.211336000000	-0.787032000000
F	-3.256923000000	-3.221891000000	-1.593431000000
C	-3.700145000000	-1.910760000000	0.306094000000
F	-4.805062000000	-2.619835000000	0.558990000000
C	-3.328852000000	-0.872201000000	1.135543000000
F	-4.082011000000	-0.578798000000	2.202156000000
C	-2.176975000000	-0.153069000000	0.857252000000
F	-1.878679000000	0.819142000000	1.730996000000

C	-0.104424000000	2.015089000000	-0.138537000000
C	0.555991000000	2.590830000000	0.952309000000
C	0.513400000000	3.938533000000	1.277426000000
C	-0.246275000000	4.799705000000	0.510528000000
C	-0.964263000000	4.292696000000	-0.555322000000
C	-0.884464000000	2.940192000000	-0.846908000000
C	2.540284000000	0.113897000000	-0.947682000000
F	2.506383000000	1.183802000000	-1.754107000000
C	3.774982000000	-0.491132000000	-0.793097000000
F	4.857101000000	-0.026275000000	-1.428853000000
C	3.890751000000	-1.594412000000	0.031029000000
F	5.072648000000	-2.197983000000	0.194207000000
C	2.767652000000	-2.062816000000	0.682560000000
F	2.868991000000	-3.126098000000	1.489161000000
H	-1.589495000000	4.932441000000	-1.164119000000
H	-0.290767000000	5.855124000000	0.751074000000
H	1.064563000000	4.295290000000	2.137527000000
Cl	-1.882281000000	2.419767000000	-2.186898000000
Cl	1.476879000000	1.608710000000	2.071663000000
H	-0.035953000000	0.510655000000	-1.853804000000

F^{-} (gas phase)

H = –99.847305079635

F	0.000000000000	0.000000000000	0.000000000000

[B1-F]$^{-}$ (gas phase)

H = –2308.47948248

F	0.776073000000	2.239956000000	-2.160751000000

C	1.596143000000	1.818490000000	-1.191494000000
C	1.295563000000	0.674520000000	-0.463697000000
B	-0.075442000000	-0.183344000000	-0.858923000000
C	-1.361306000000	0.745347000000	-0.382015000000
C	-2.388573000000	1.150983000000	-1.220051000000
F	-2.456699000000	0.747060000000	-2.490731000000
C	-3.419169000000	1.985172000000	-0.806536000000
F	-4.385954000000	2.348732000000	-1.656134000000
C	-3.453189000000	2.444644000000	0.493880000000
F	-4.439440000000	3.246061000000	0.904025000000
C	-2.455612000000	2.058336000000	1.369080000000
F	-2.483800000000	2.484163000000	2.635948000000
C	-1.449273000000	1.227036000000	0.915445000000
F	-0.530383000000	0.862922000000	1.823751000000
C	-0.088493000000	-1.716899000000	-0.227727000000
C	0.941134000000	-2.579749000000	-0.584437000000
F	1.958146000000	-2.138638000000	-1.334223000000
C	1.012727000000	-3.902294000000	-0.189322000000
F	2.038553000000	-4.677683000000	-0.554309000000
C	0.009241000000	-4.428027000000	0.603422000000
F	0.056575000000	-5.702417000000	0.997802000000
C	-1.041443000000	-3.617612000000	0.976979000000
F	-2.022926000000	-4.116185000000	1.735981000000
C	-1.072545000000	-2.295218000000	0.554751000000
F	-2.145841000000	-1.598451000000	0.958723000000
C	2.197360000000	0.382055000000	0.545867000000
F	2.014109000000	-0.672470000000	1.353360000000
C	3.329530000000	1.136867000000	0.808143000000
F	4.166285000000	0.799400000000	1.794750000000
C	3.594433000000	2.254771000000	0.043324000000
F	4.679264000000	2.996069000000	0.280663000000

C	2.716337000000	2.601414000000	-0.965168000000
F	2.955795000000	3.691214000000	-1.702110000000
F	-0.080793000000	-0.356973000000	-2.265859000000

[B2-F]$^{-}$ (gas phase)

H = –2532.90122375

C	1.009847000000	2.700041000000	-0.759939000000
C	0.152472000000	1.813284000000	-0.093202000000
B	0.029846000000	0.236346000000	-0.609278000000
C	-1.410793000000	-0.490042000000	-0.222091000000
C	-1.588440000000	-1.609128000000	0.575207000000
F	-0.557976000000	-2.158508000000	1.240516000000
C	-2.822185000000	-2.209231000000	0.766539000000
F	-2.916090000000	-3.294548000000	1.555524000000
C	-3.956133000000	-1.710686000000	0.166169000000
C	-3.811647000000	-0.592103000000	-0.626760000000
F	-4.895967000000	-0.061995000000	-1.220020000000
C	-2.573339000000	-0.005347000000	-0.810598000000
F	-2.539339000000	1.101509000000	-1.564012000000
C	1.340956000000	-0.659224000000	-0.131998000000
C	2.180652000000	-0.348599000000	0.926187000000
F	1.929390000000	0.704111000000	1.721545000000
C	3.304289000000	-1.094778000000	1.241070000000
F	4.075944000000	-0.728028000000	2.279738000000
C	3.642817000000	-2.209957000000	0.508934000000
C	2.814415000000	-2.555657000000	-0.537165000000
F	3.098036000000	-3.650367000000	-1.265530000000
C	1.694080000000	-1.802762000000	-0.839071000000
F	0.923945000000	-2.249987000000	-1.838978000000

C	-0.551815000000	2.421093000000	0.949976000000
C	-0.508216000000	3.776062000000	1.243919000000
C	0.302760000000	4.607122000000	0.497921000000
C	1.085759000000	4.059650000000	-0.499189000000
F	0.024462000000	0.264395000000	-2.035862000000
H	4.516708000000	-2.797969000000	0.751332000000
H	-4.921075000000	-2.175079000000	0.312433000000
H	-1.101567000000	4.160619000000	2.062862000000
H	0.344610000000	5.668910000000	0.709443000000
H	1.766760000000	4.671993000000	-1.075489000000
Cl	2.148823000000	2.131791000000	-1.958609000000
Cl	-1.526289000000	1.483414000000	2.063755000000

[B3-F]$^{-}$ (gas phase)

H = –2731.40879741

C	0.967617000000	2.932704000000	-0.710709000000
C	0.129286000000	2.006078000000	-0.074328000000
B	0.036097000000	0.446071000000	-0.645249000000
C	-1.387188000000	-0.321870000000	-0.273544000000
C	-2.562840000000	0.159533000000	-0.835256000000
F	-2.556110000000	1.284367000000	-1.558651000000
C	-3.798725000000	-0.437902000000	-0.661867000000
F	-4.899701000000	0.078800000000	-1.218771000000
C	-3.898594000000	-1.580687000000	0.108715000000
C	-2.759632000000	-2.096713000000	0.691579000000
F	-2.844532000000	-3.199089000000	1.444972000000
C	-1.542147000000	-1.463747000000	0.492686000000
F	-0.500725000000	-2.019863000000	1.131842000000
C	1.366854000000	-0.438427000000	-0.202287000000

C	1.731713000000	-1.558175000000	-0.938598000000
F	0.962021000000	-1.993997000000	-1.941490000000
C	2.863229000000	-2.311703000000	-0.677746000000
F	3.165521000000	-3.381817000000	-1.421620000000
C	3.685936000000	-1.962456000000	0.376002000000
C	3.354011000000	-0.871329000000	1.151701000000
F	4.135701000000	-0.531790000000	2.182895000000
C	2.212352000000	-0.143359000000	0.853711000000
F	1.958389000000	0.883220000000	1.678326000000
C	-0.581917000000	2.562992000000	0.992387000000
C	-0.562357000000	3.906995000000	1.335202000000
C	0.230467000000	4.779381000000	0.617731000000
C	1.019888000000	4.283009000000	-0.400800000000
F	0.023315000000	0.520224000000	-2.069428000000
H	-1.159811000000	4.250229000000	2.169295000000
H	0.253470000000	5.833239000000	0.867941000000
H	1.687864000000	4.927722000000	-0.956542000000
Cl	2.112041000000	2.430584000000	-1.933623000000
Cl	-1.535478000000	1.570432000000	2.077507000000
F	-5.081580000000	-2.174796000000	0.288325000000
F	4.780895000000	-2.678604000000	0.646192000000

8. Crystallographic data

For single crystal X-ray diffraction analysis, the single crystals of **2ad** and **2ae** were grown by slow evaporation of methanol solution. Suitable crystals were selected and measured on a **XtaLAB Synergy**, **Dualflex**, **Pilatus 300K** diffractometer. The crystals were kept at 100.0(1) K during data collection. Using Olex2,[63] the structures were solved with the SHELXT structure solution program[64] using Intrinsic Phasing and refined with the SHELXL refinement package[65] using Least Squares minimisation.

**Crystal data**	**2ad**	**2ae**
CCDC code	2444861	2444863
Identification code	w010225_4_2	w170225_2_1
Empirical formula	$C_{17}H_{18}O$	$C_{20}H_{16}O$
Formula weight	238.31	272.33
Temperature/K	100.0(1)	100.0(1)
Crystal system	orthorhombic	orthorhombic
Space group	Pnma	Fmm2
a, Å	6.69380(10)	9.8889(4)
b, Å	24.4670(5)	18.9697(6)
c, Å	8.1899(2)	7.5927(2)
α, °	90	90
β, °	90	90
γ, °	90	90
Volume, Å^3	1341.32(5)	1424.31(8)
Z	4	4
ρ_{calc}, g/cm^3	1.180	1.270
μ, mm^{-1}	0.550	0.593
F(000)	512.0	576.0
Crystal size, mm^3	0.291 × 0.176 × 0.026	0.218 × 0.139 × 0.059
Radiation	Cu Kα (λ = 1.54184)	Cu Kα (λ = 1.54184)
2Θ range for data collection, °	7.226 to 149.524	9.324 to 145.818
Index ranges	$-8 \le h \le 7$, $-30 \le k \le 30$, $-10 \le l \le 10$	$-12 \le h \le 11$, $-23 \le k \le 22$, $-7 \le l \le 8$
Reflections collected	40088	7368
Independent reflections	1385 [R_{int} = 0.0479, R_{sigma} = 0.0129]	714 [R_{int} = 0.0302, R_{sigma} = 0.0153]
Data/ restraints/ parameters	1385/229/143	714/4/100
Goodness-of-fit on F^2	1.087	1.102
Final R indexes [I≥2σ (I)]	R_1 = 0.0723, wR_2 = 0.1842	R_1 = 0.0302, wR_2 = 0.0744
Final R indexes [all data]	R_1 = 0.0789, wR_2 = 0.1896	R_1 = 0.0313, wR_2 = 0.0751
Largest diff. peak/hole / e·Å^{-3}	0.29/–0.33	0.08/–0.16

Crystal Structure of 2ad (CCDC 2444861)

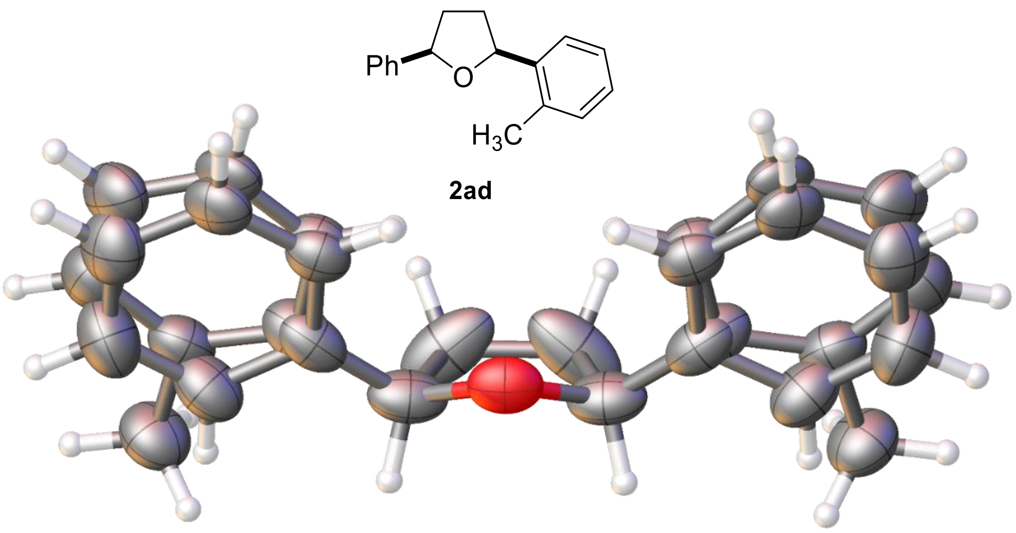

Note: the crystallographic model includes disorder, which was modelled as described. For clarity, only the major component is shown in the manuscript.

Crystal Structure of 2ae (CCDC 2444863)

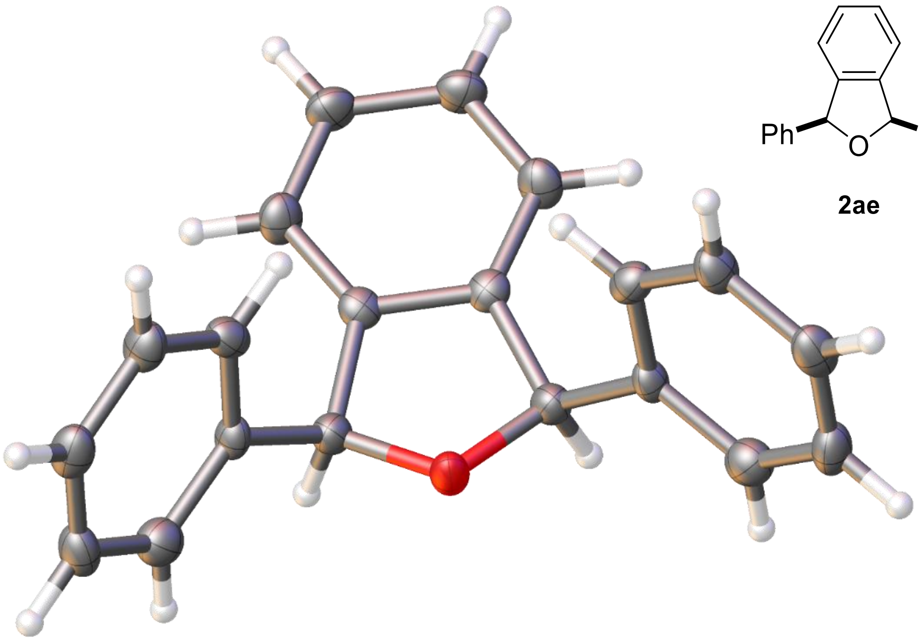

9. NMR spectra of diketone substrates and cyclic ether products

^{1}H NMR (400 MHz, $CDCl_3$) of **1b**

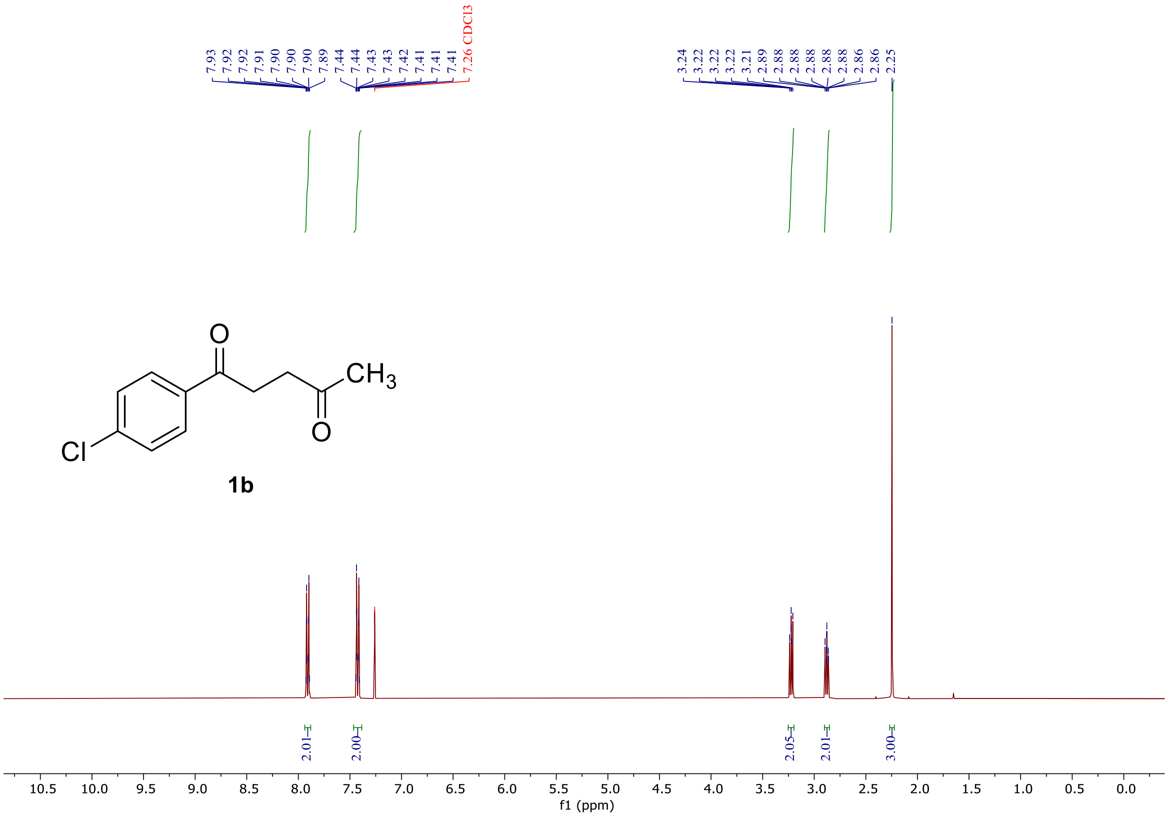

$^{13}C\{^1H\}$ NMR (101 MHz, $CDCl_3$) of **1b**

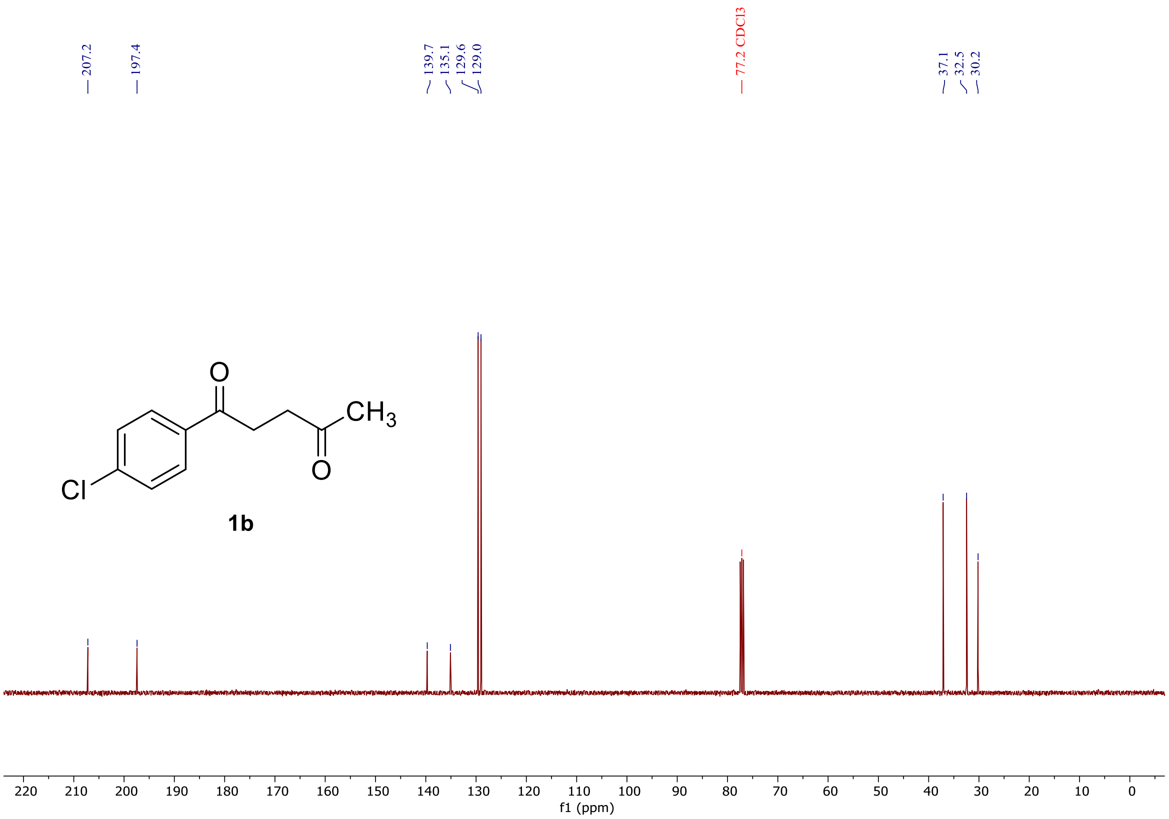

^{1}H NMR (400 MHz, $CDCl_3$) of **1c**

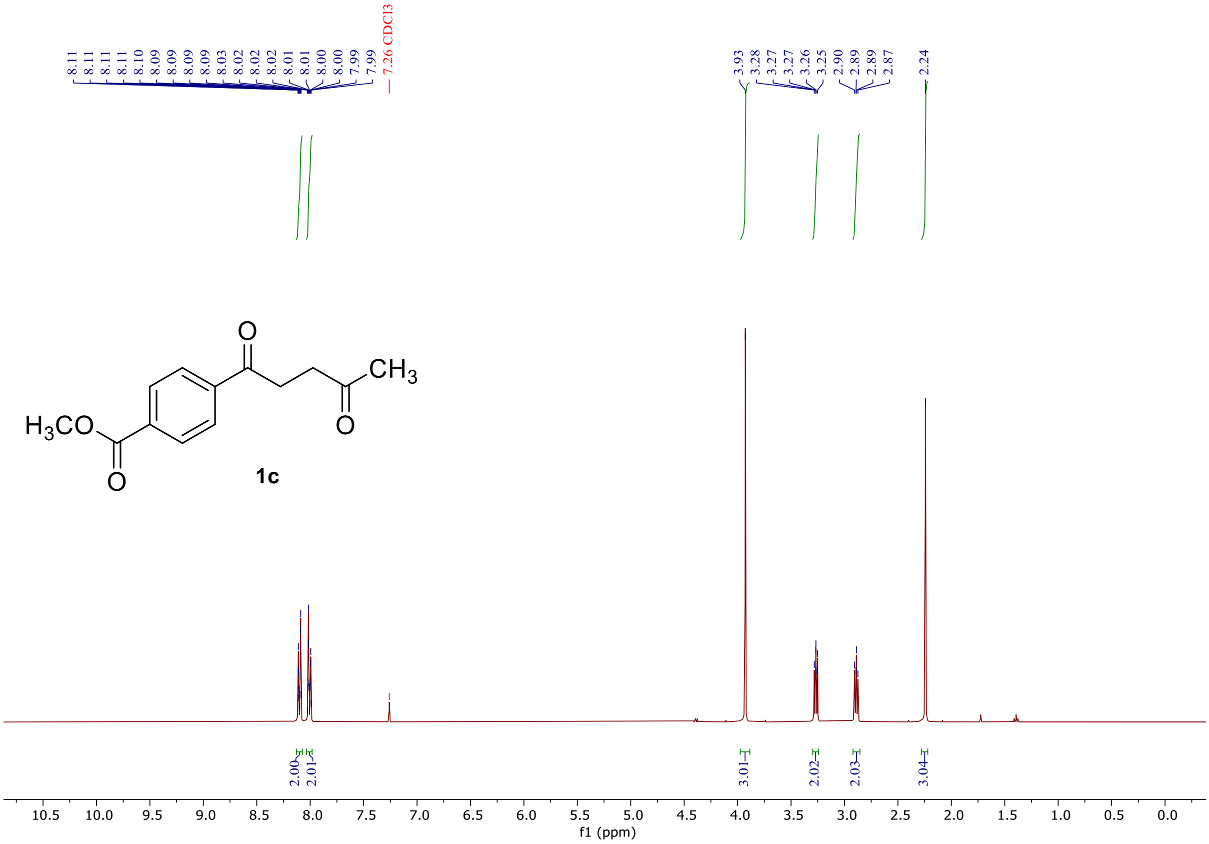

^{13}C{^{1}H} NMR (101 MHz, $CDCl_3$) of **1c**

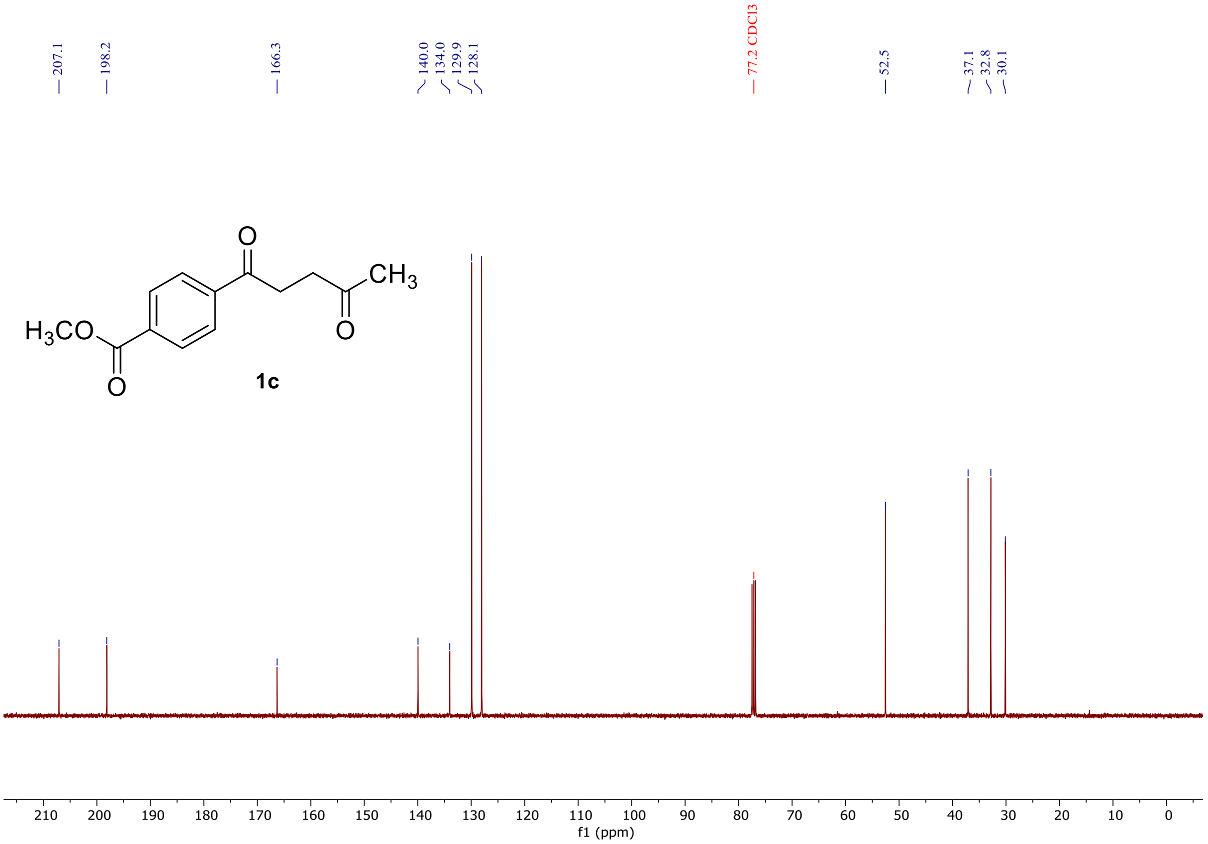

^{1}H NMR (500 MHz, $CDCl_3$) of **1d**

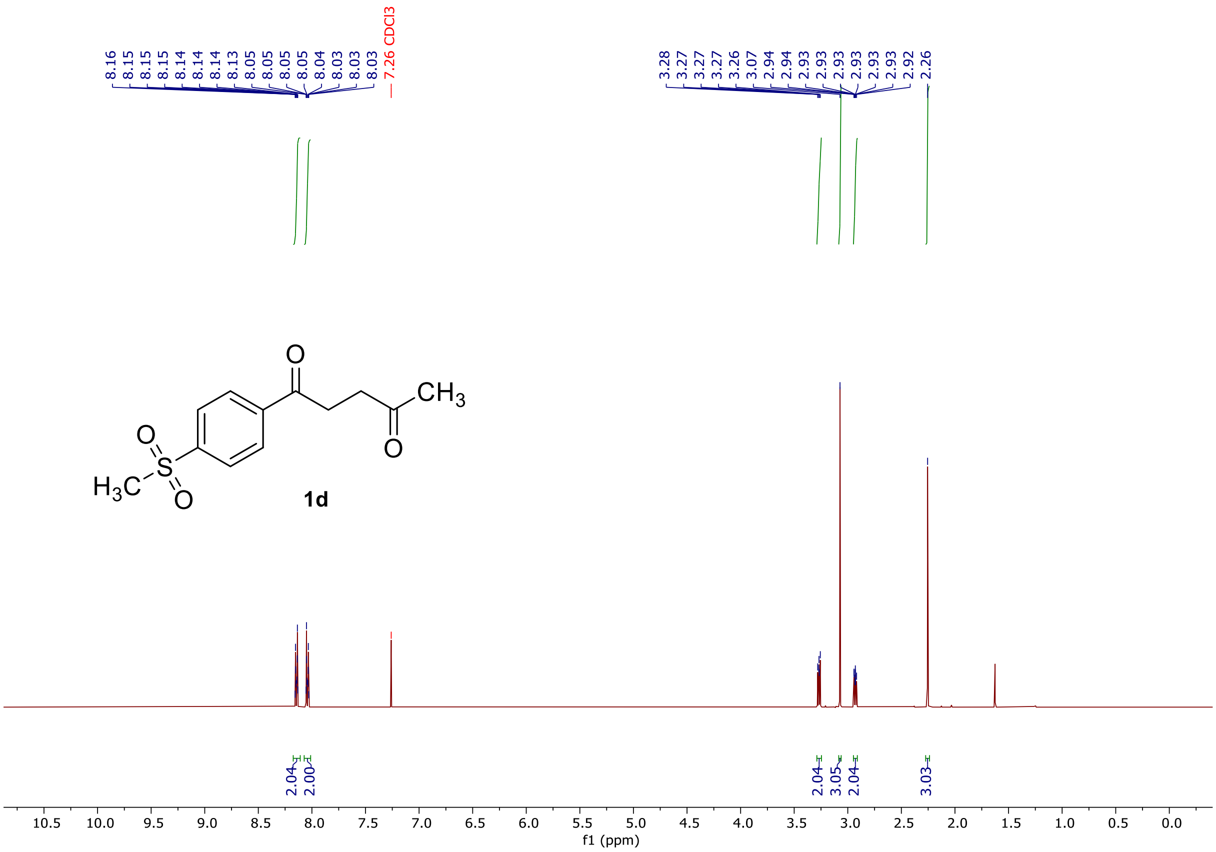

$^{13}C\{^{1}H\}$ NMR (126 MHz, $CDCl_3$) of **1d**

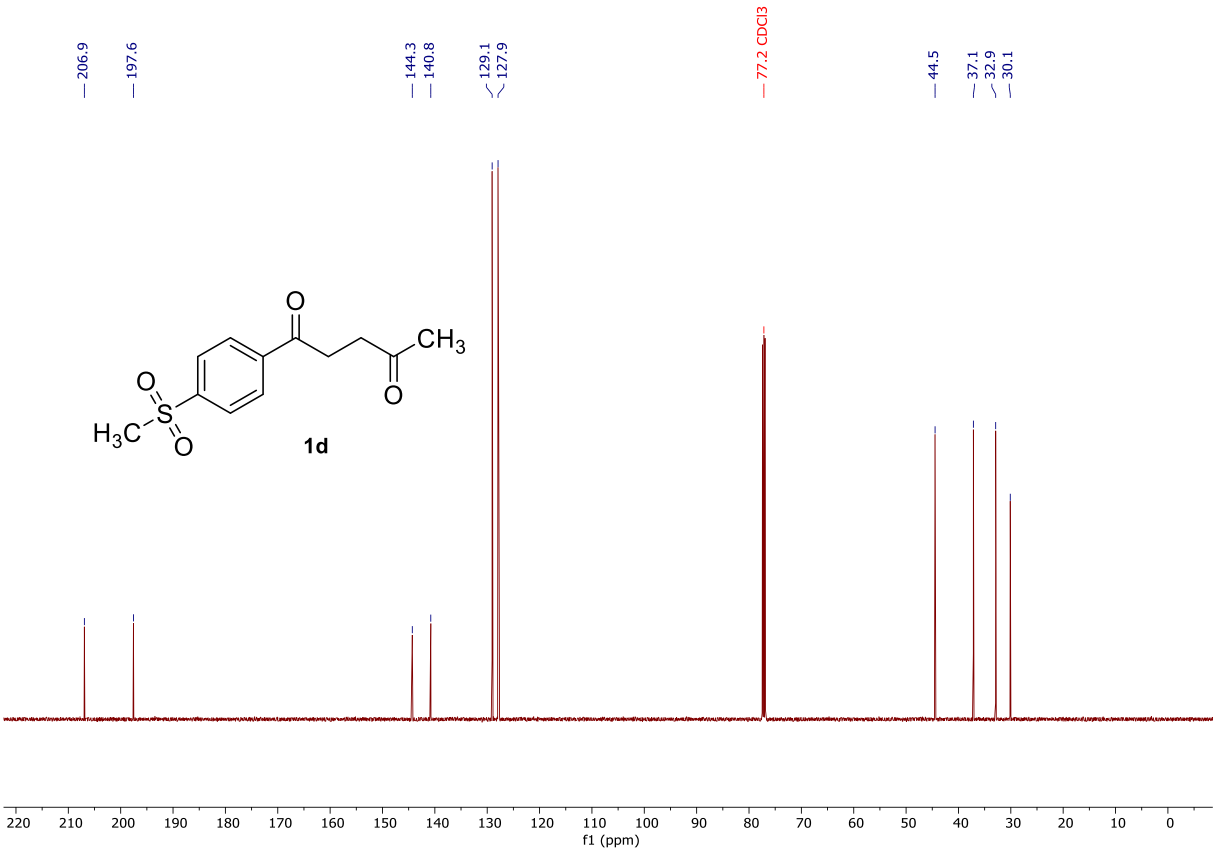

^{1}H NMR (400 MHz, $CDCl_3$) of **1e**

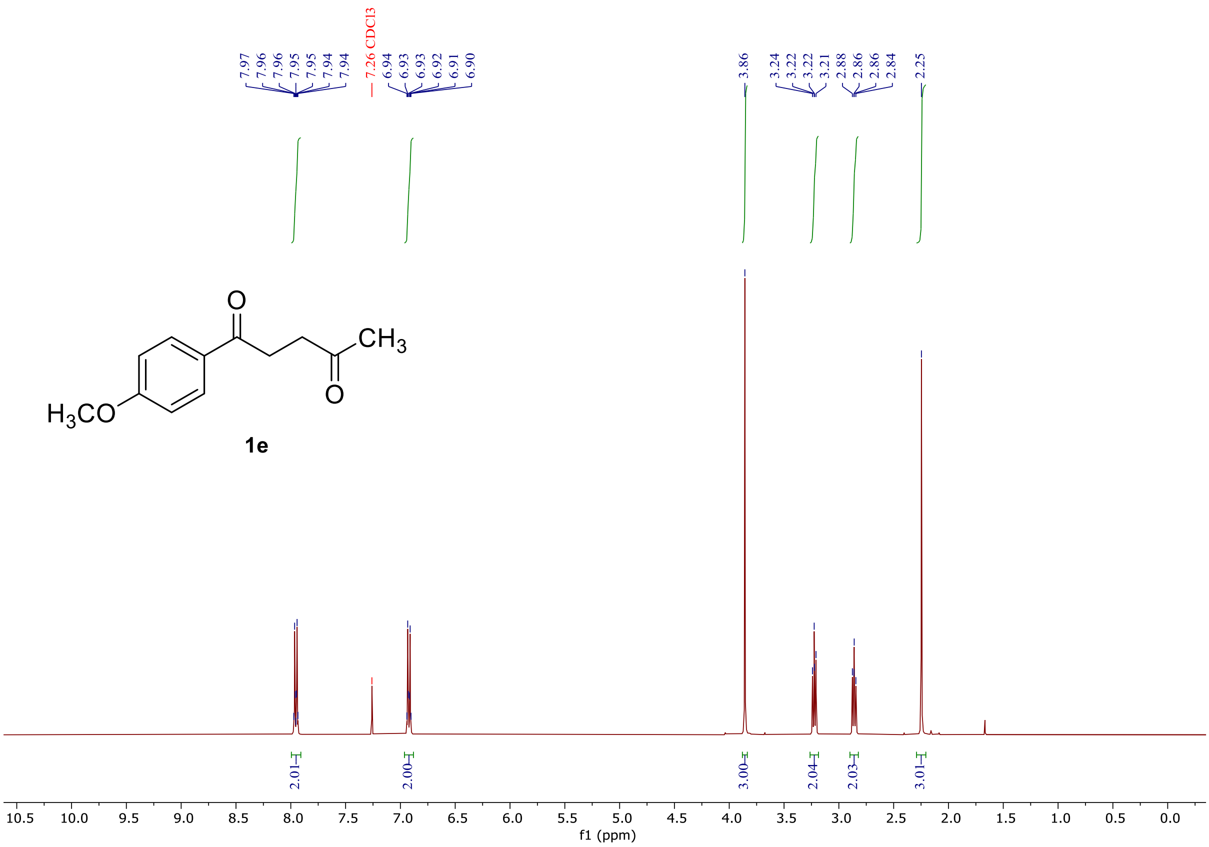

$^{13}C\{^{1}H\}$ NMR (101 MHz, $CDCl_3$) of **1e**

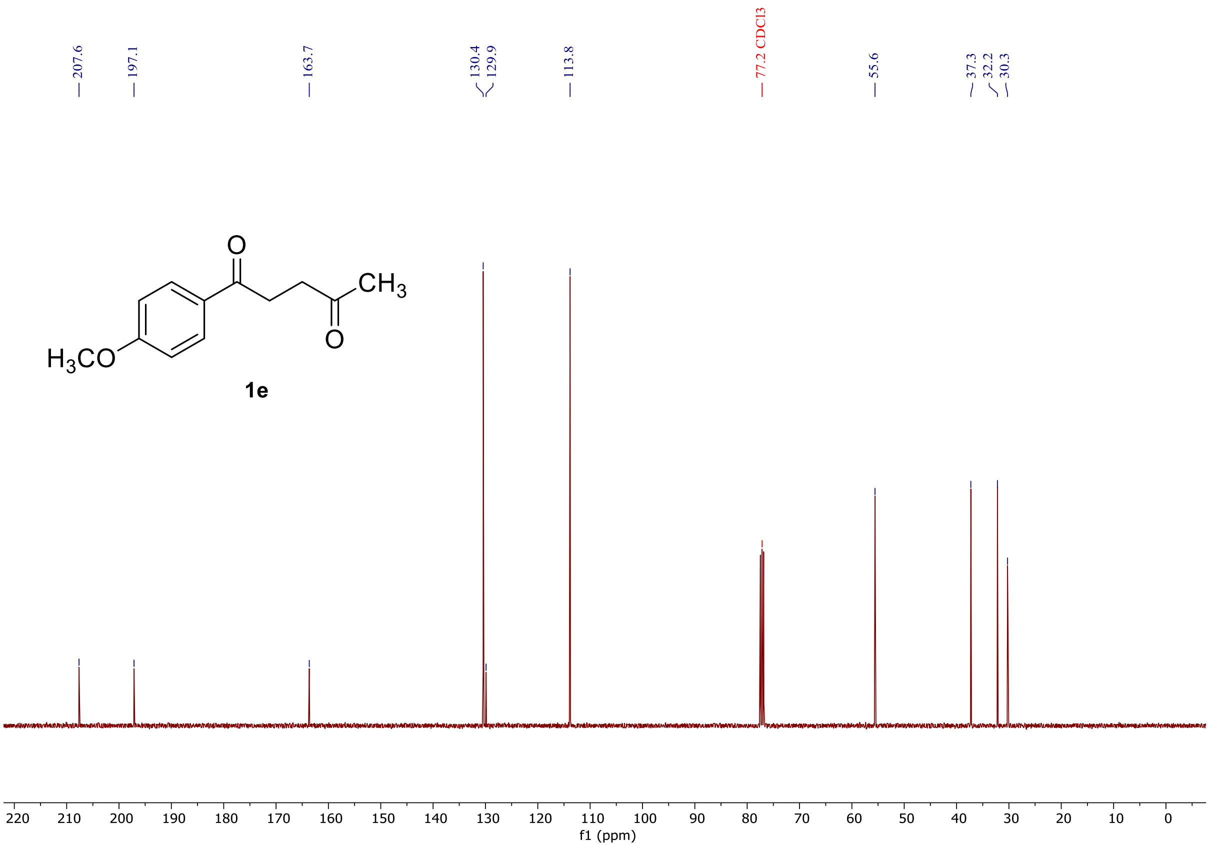

^{1}H NMR (400 MHz, $CDCl_3$) of **1f**

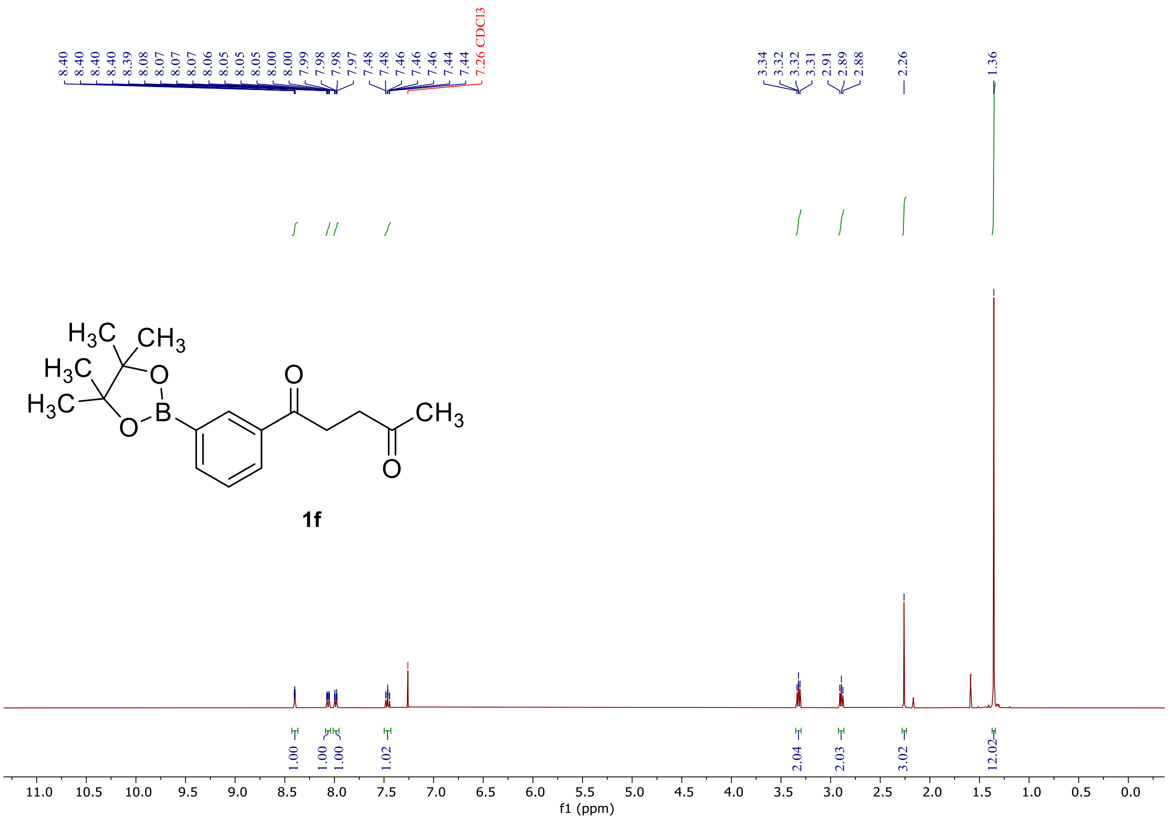

^{13}C{^{1}H} NMR (101 MHz, $CDCl_3$) of **1f**

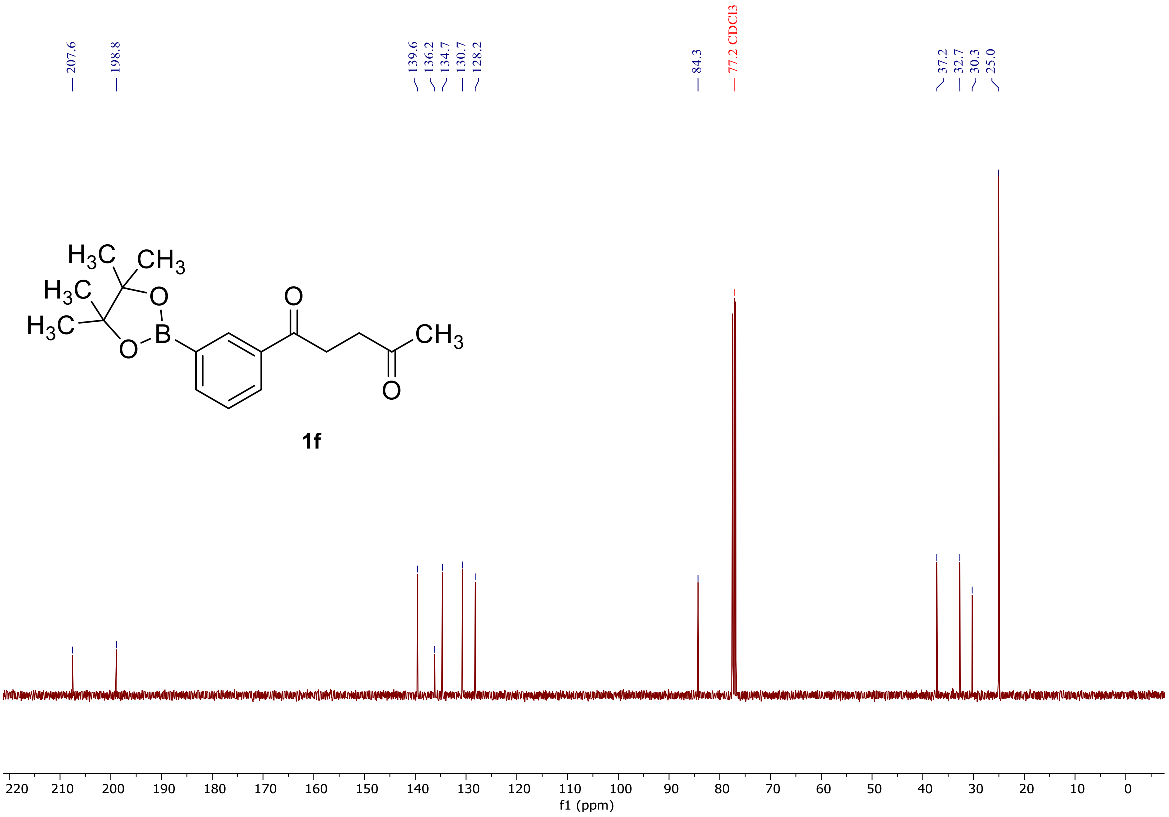

^{11}B NMR (128 MHz, $CDCl_3$) of **1f**

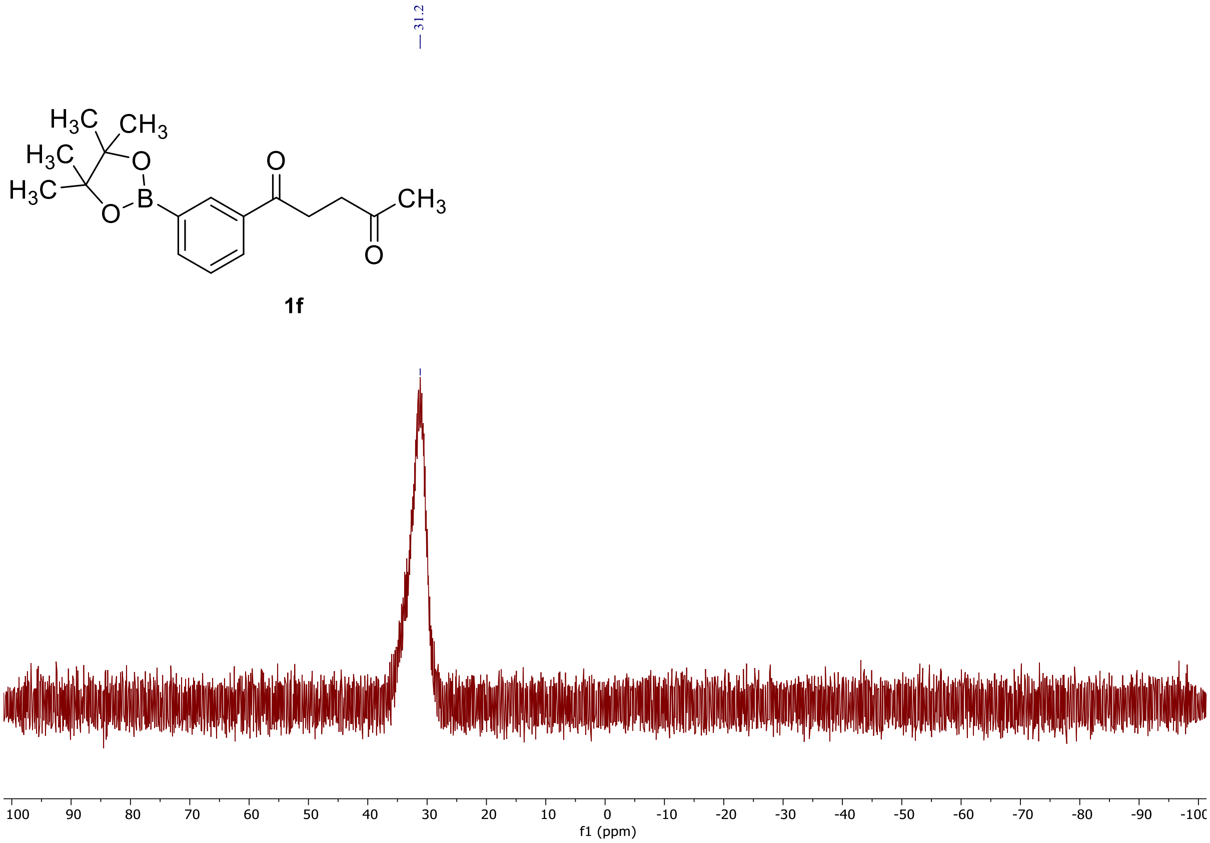

^{1}H NMR (500 MHz, $CDCl_3$) of **1g**

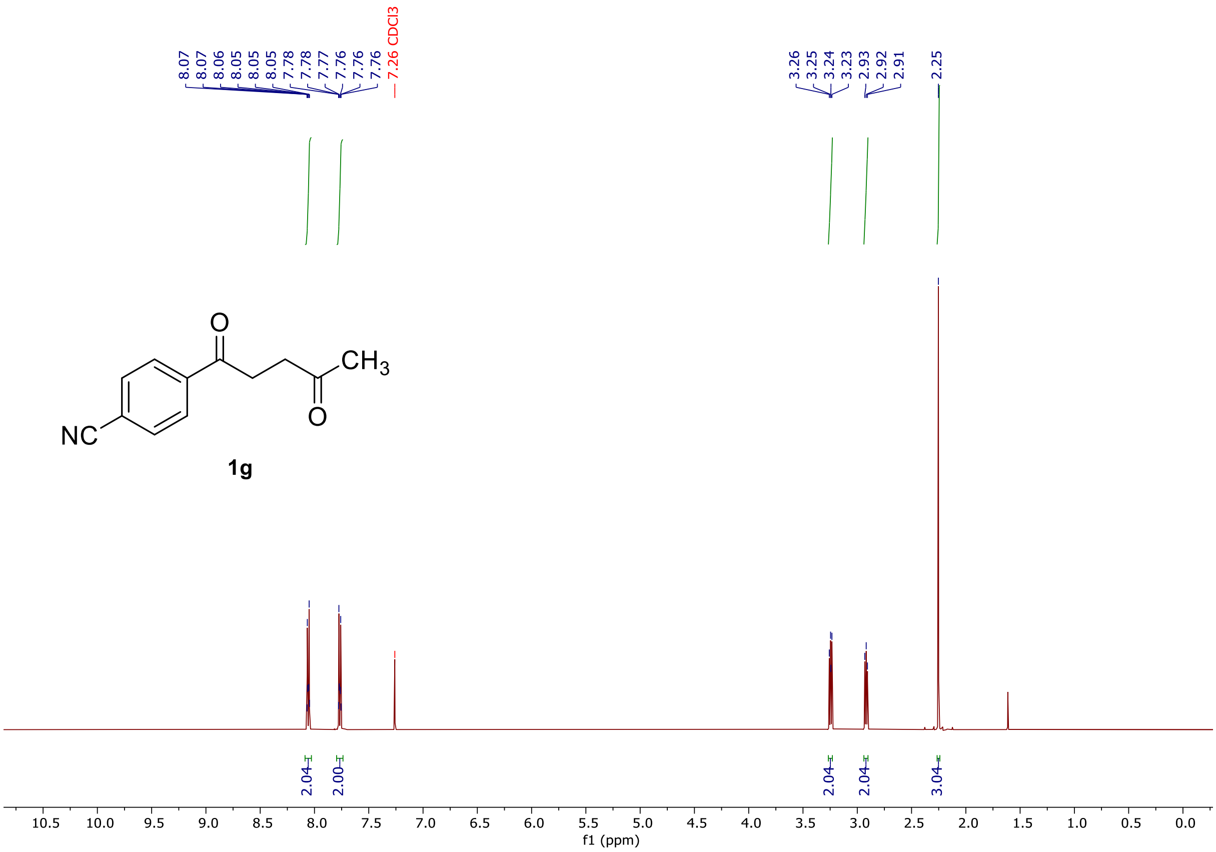

^{13}C{^{1}H} NMR (126 MHz, $CDCl_3$) of **1g**

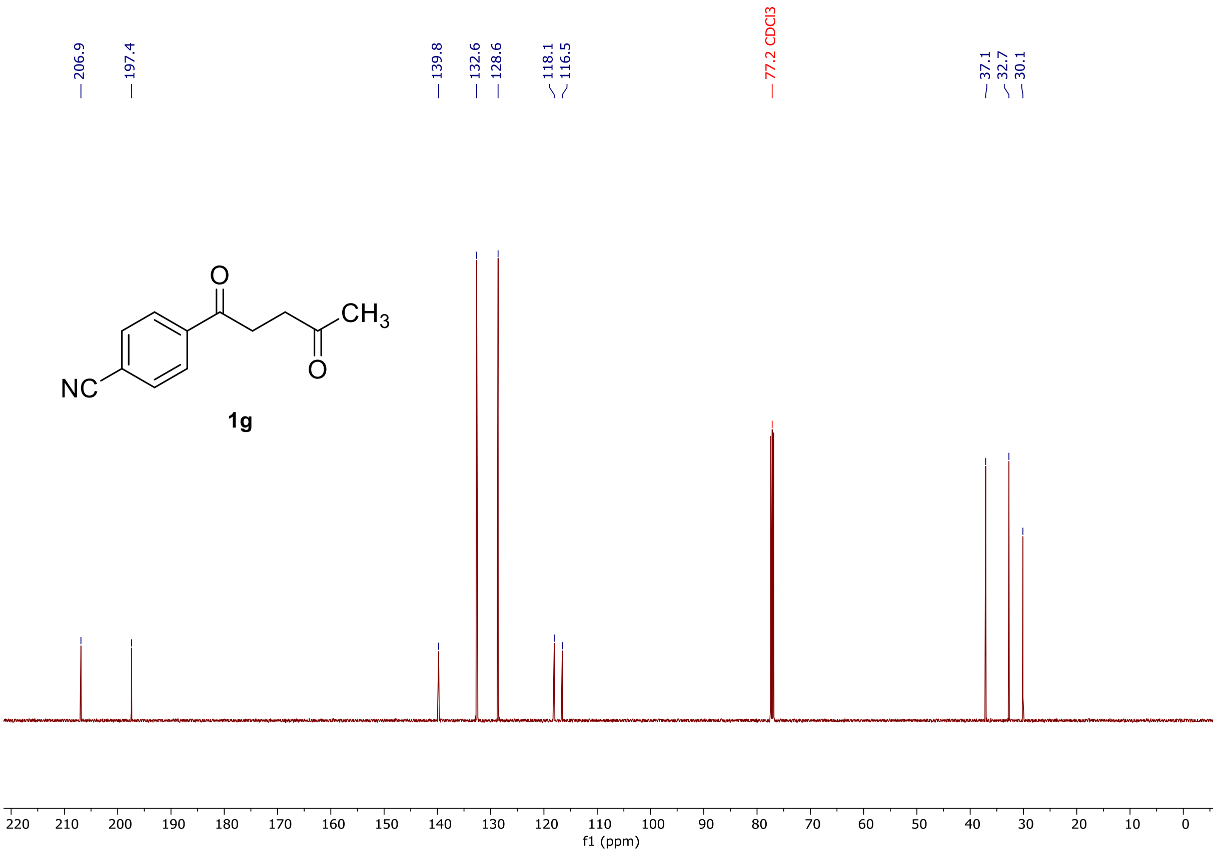

^{1}H NMR (500 MHz, $CDCl_3$) of **1h**

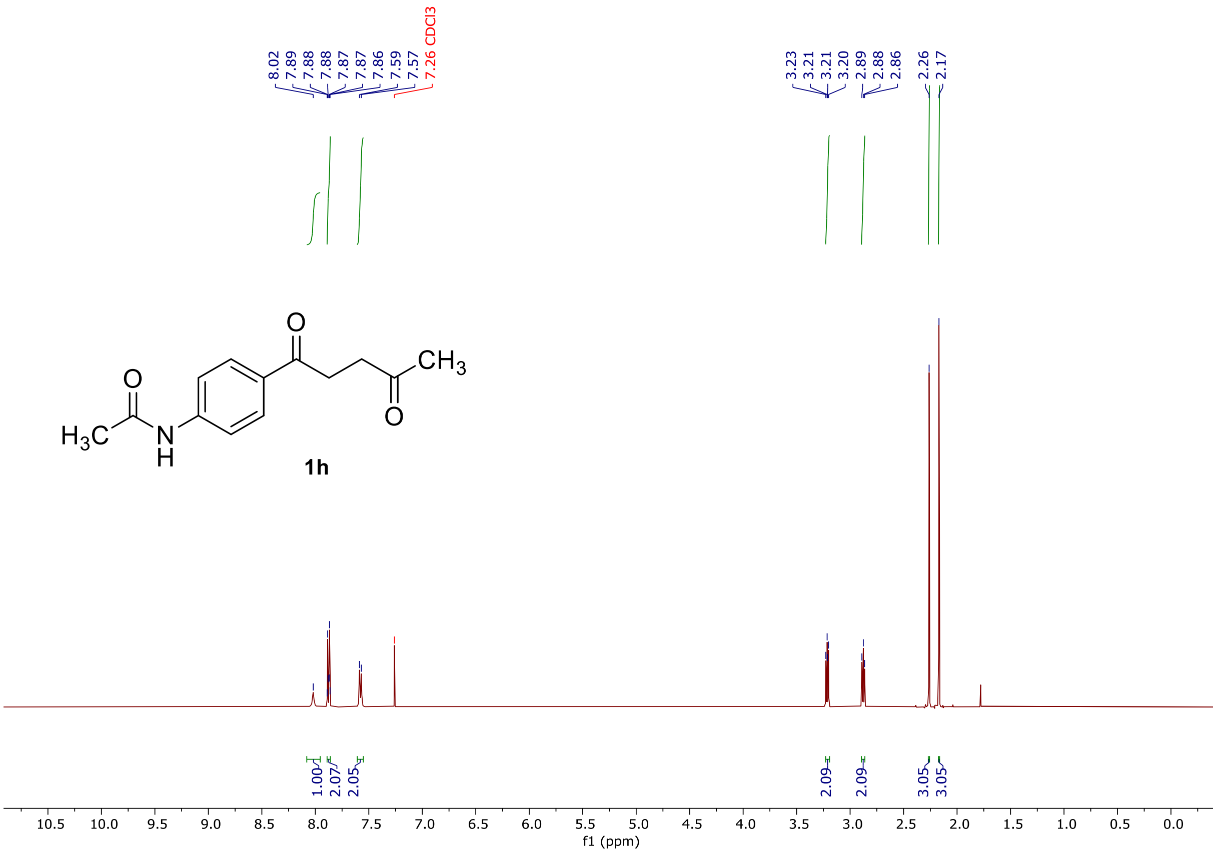

$^{13}C\{^{1}H\}$ NMR (126 MHz, $CDCl_3$) of **1h**

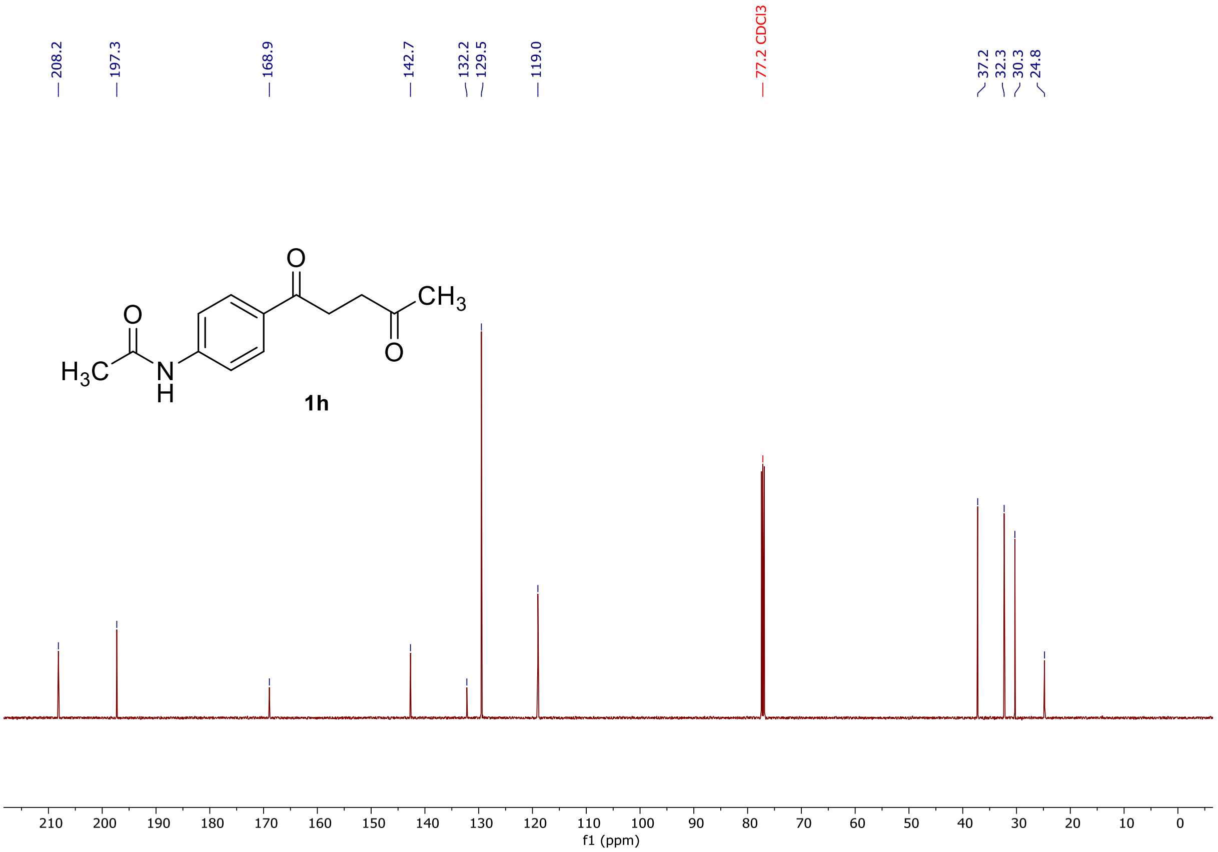

^{1}H NMR (400 MHz, $CDCl_3$) of **1i**

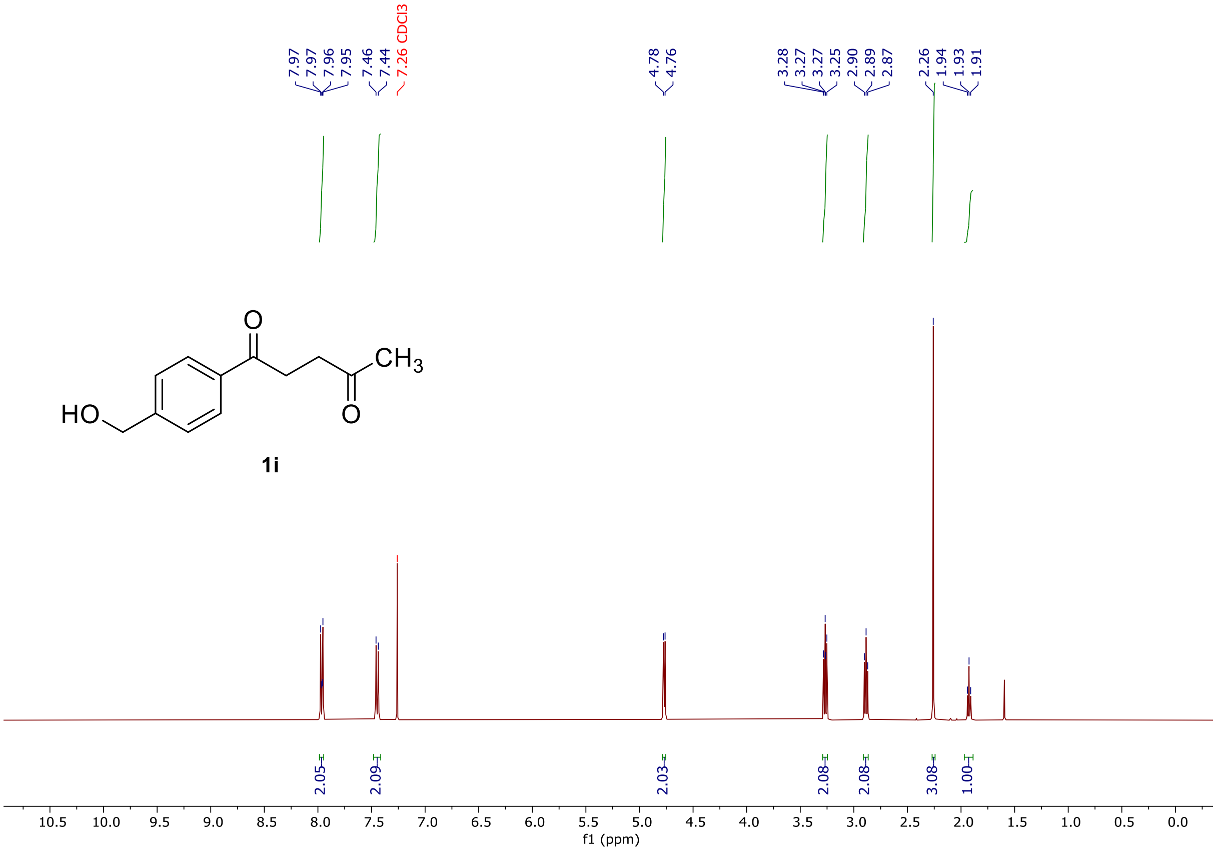

$^{13}C\{^{1}H\}$ NMR (101 MHz, $CDCl_3$) of **1i**

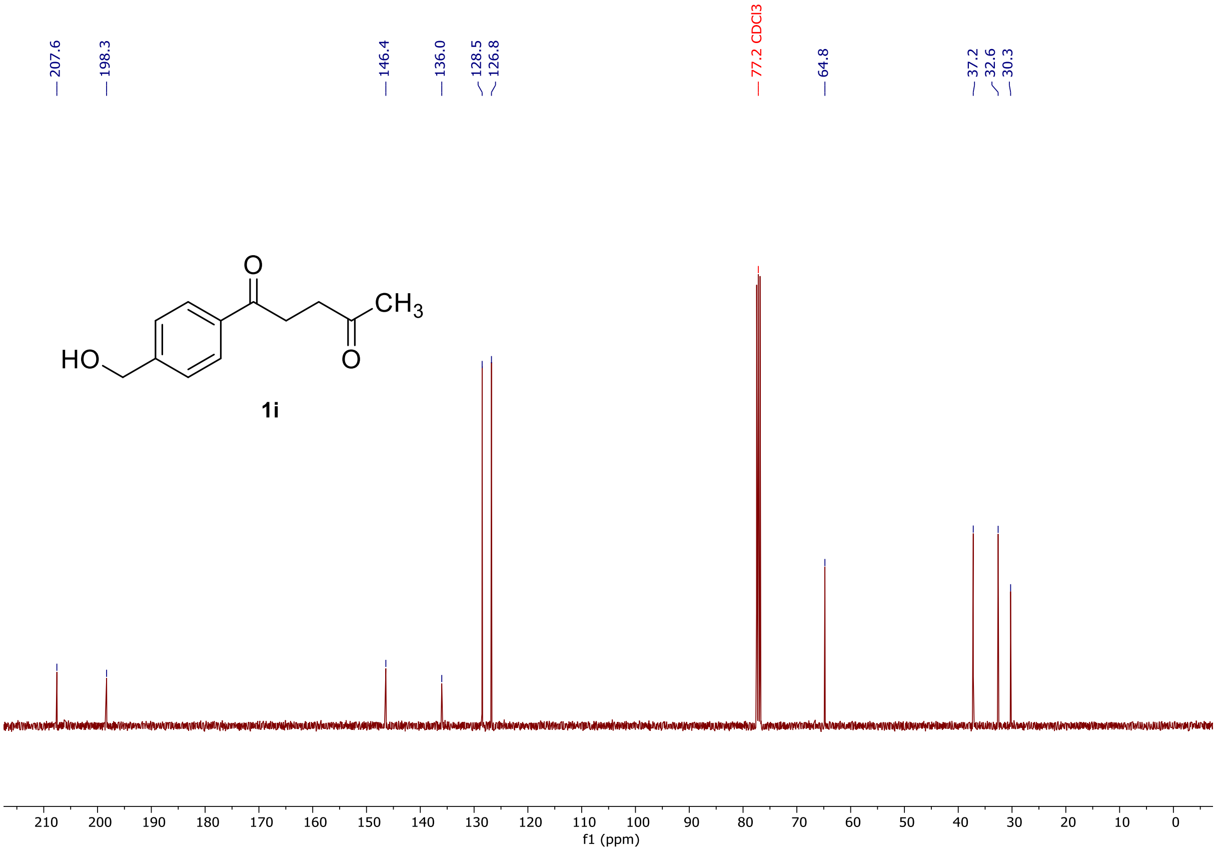

^{1}H NMR (400 MHz, $CDCl_3$) of **1j**

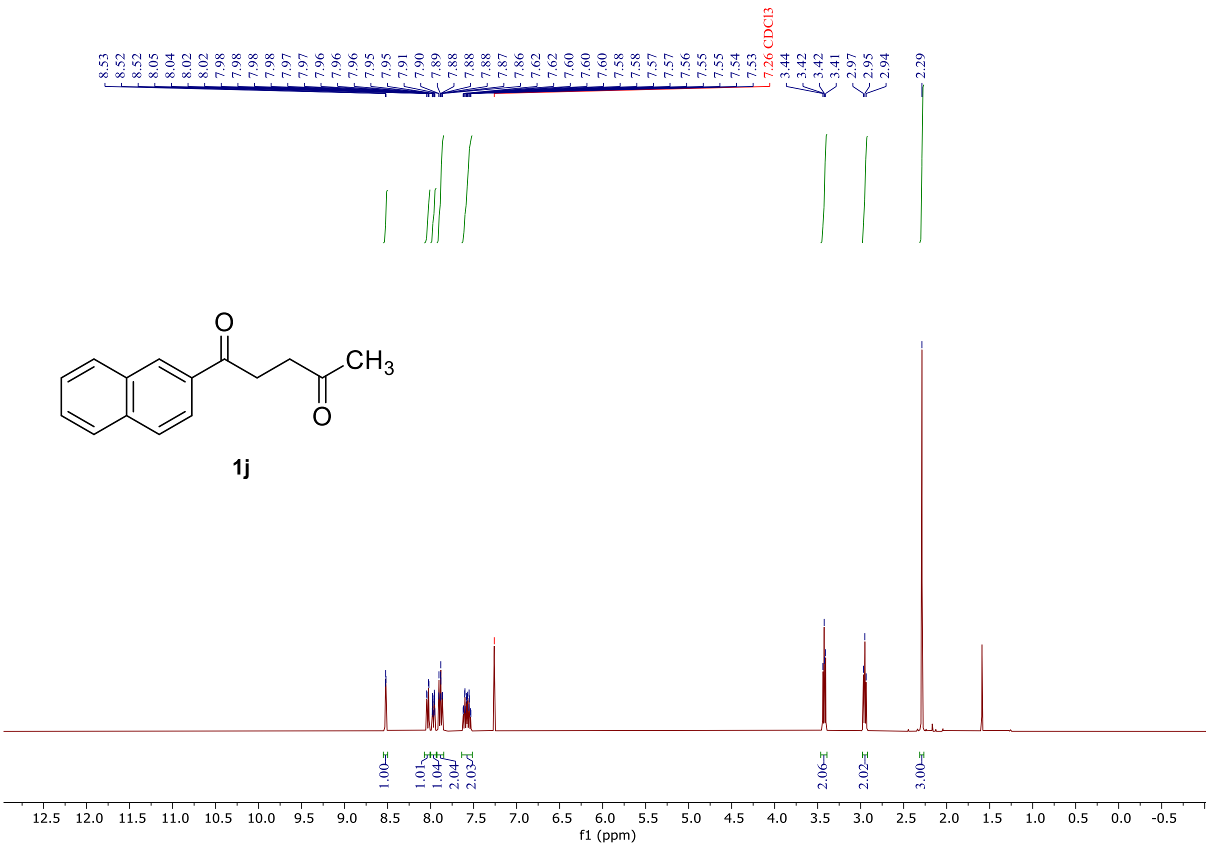

^{13}C{^{1}H} NMR (101 MHz, $CDCl_3$) of **1j**

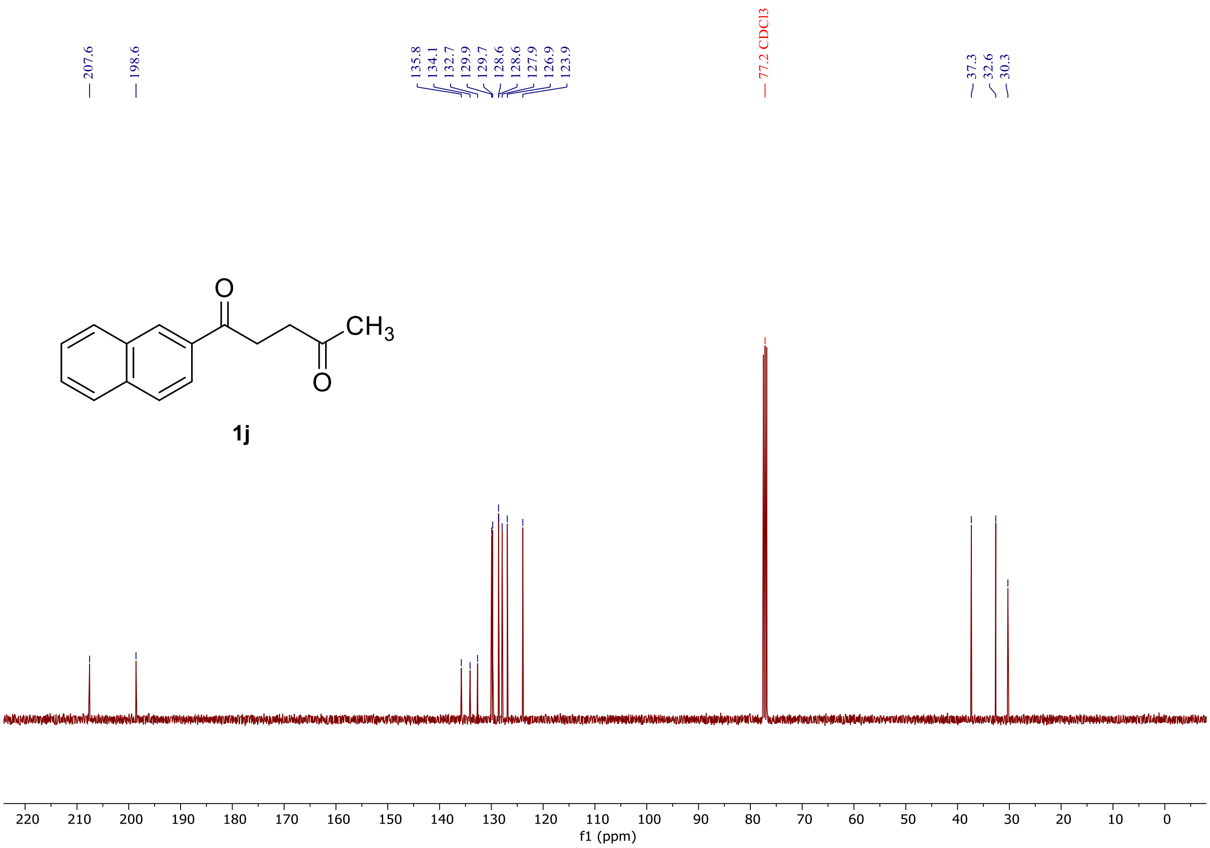

^{1}H NMR (400 MHz, $CDCl_3$) of **1k**

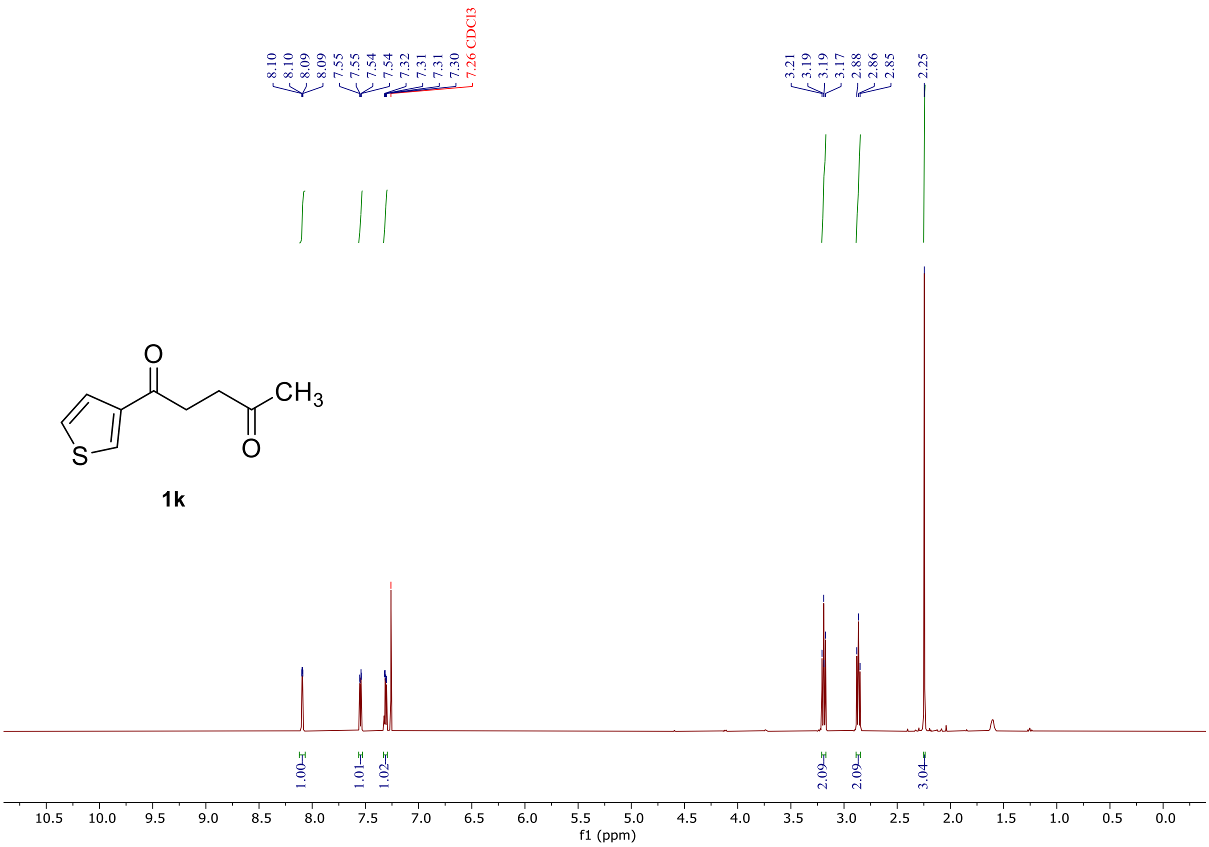

^{13}C{^{1}H} NMR (101 MHz, $CDCl_3$) of **1k**

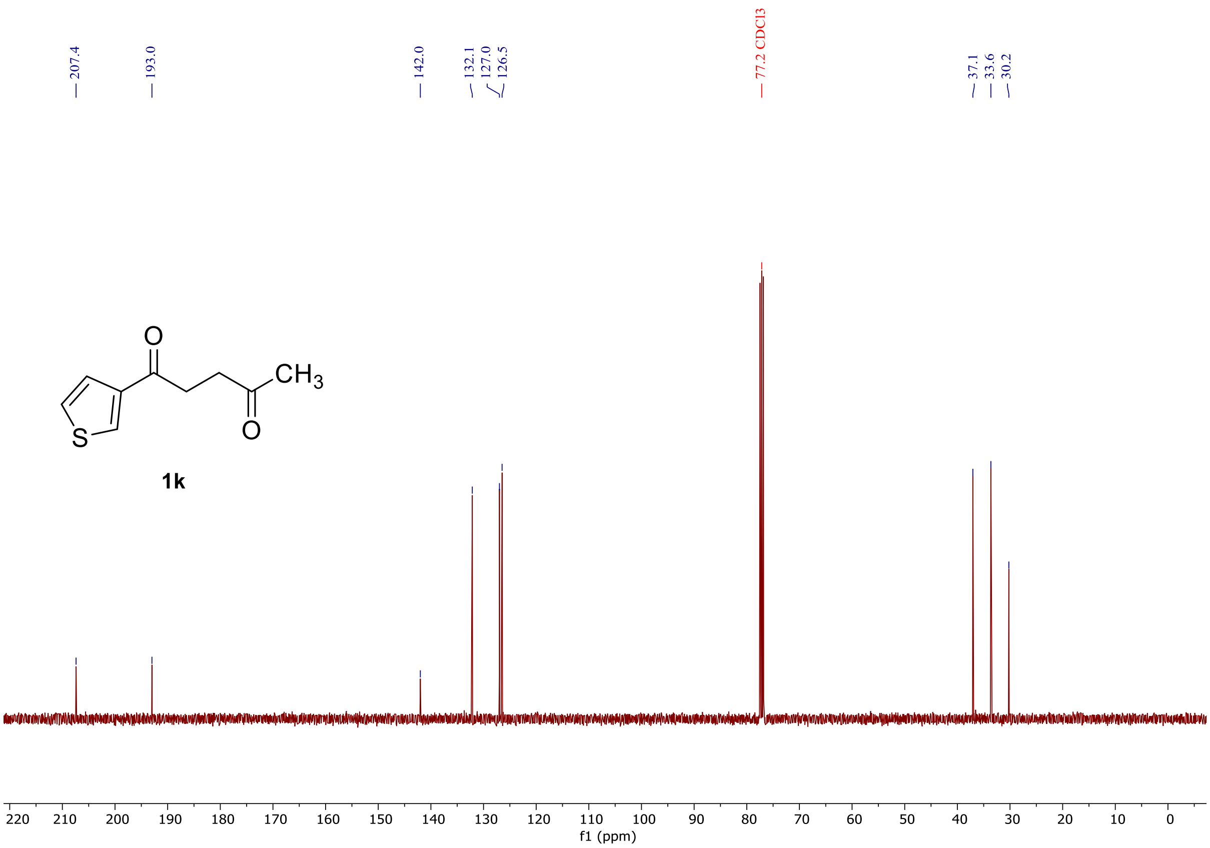

^{1}H NMR (500 MHz, $CDCl_3$) of **1l**

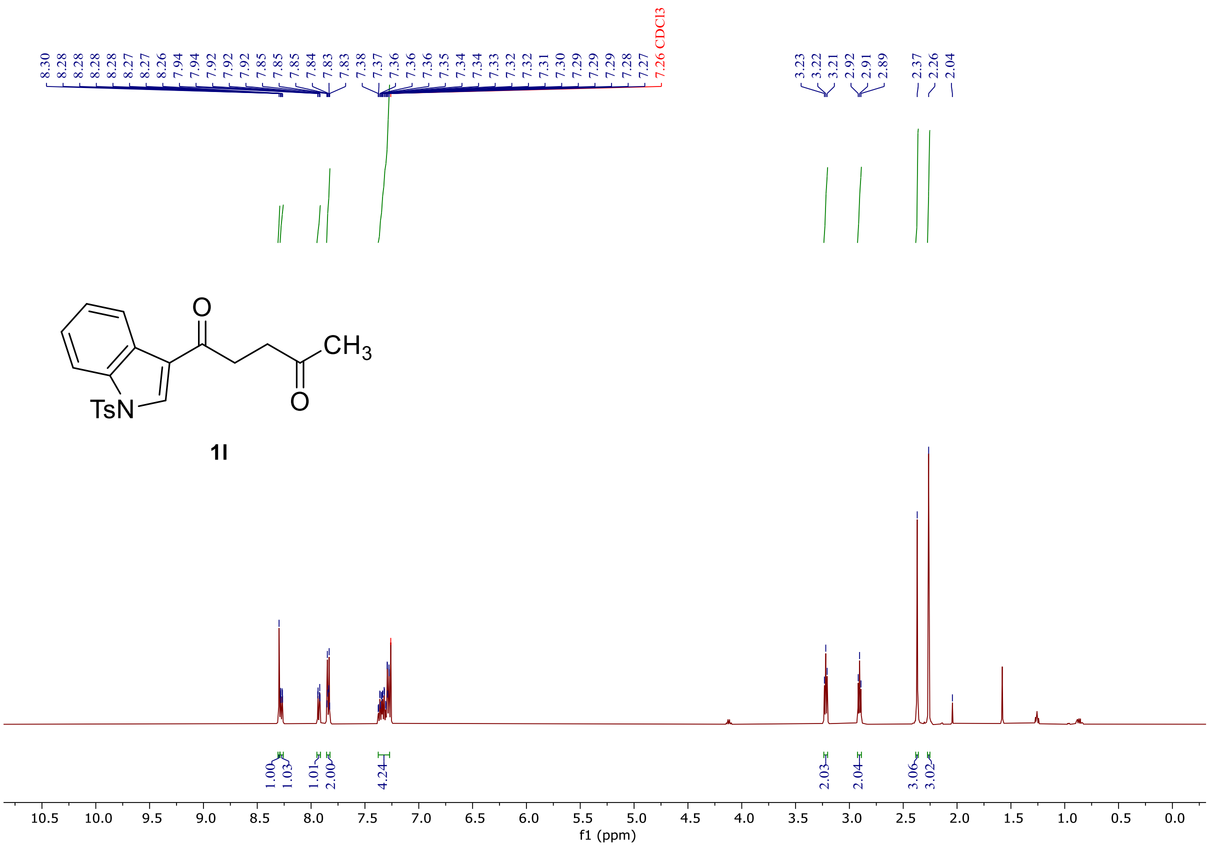

$^{13}C\{^{1}H\}$ NMR (126 MHz, $CDCl_3$) of **1l**

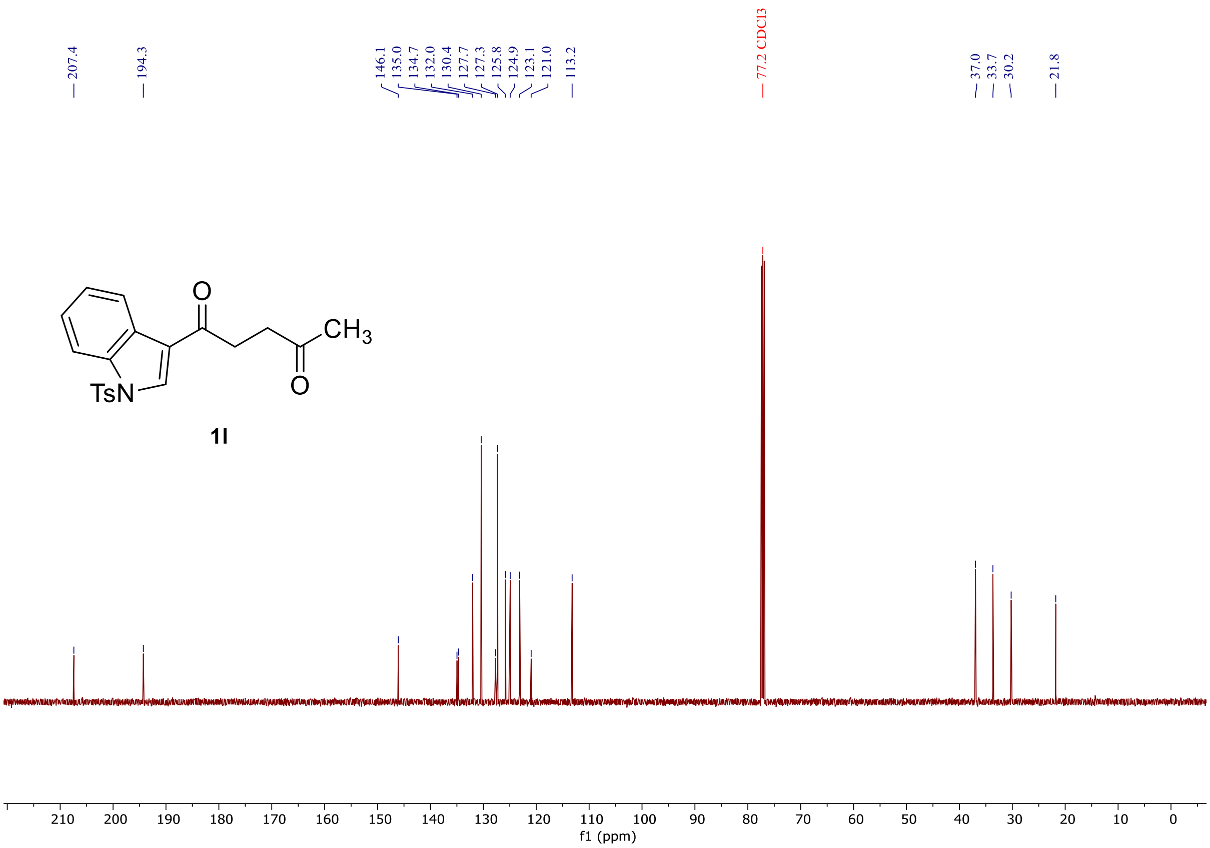

^{1}H NMR (400 MHz, $CDCl_3$) of **1m**

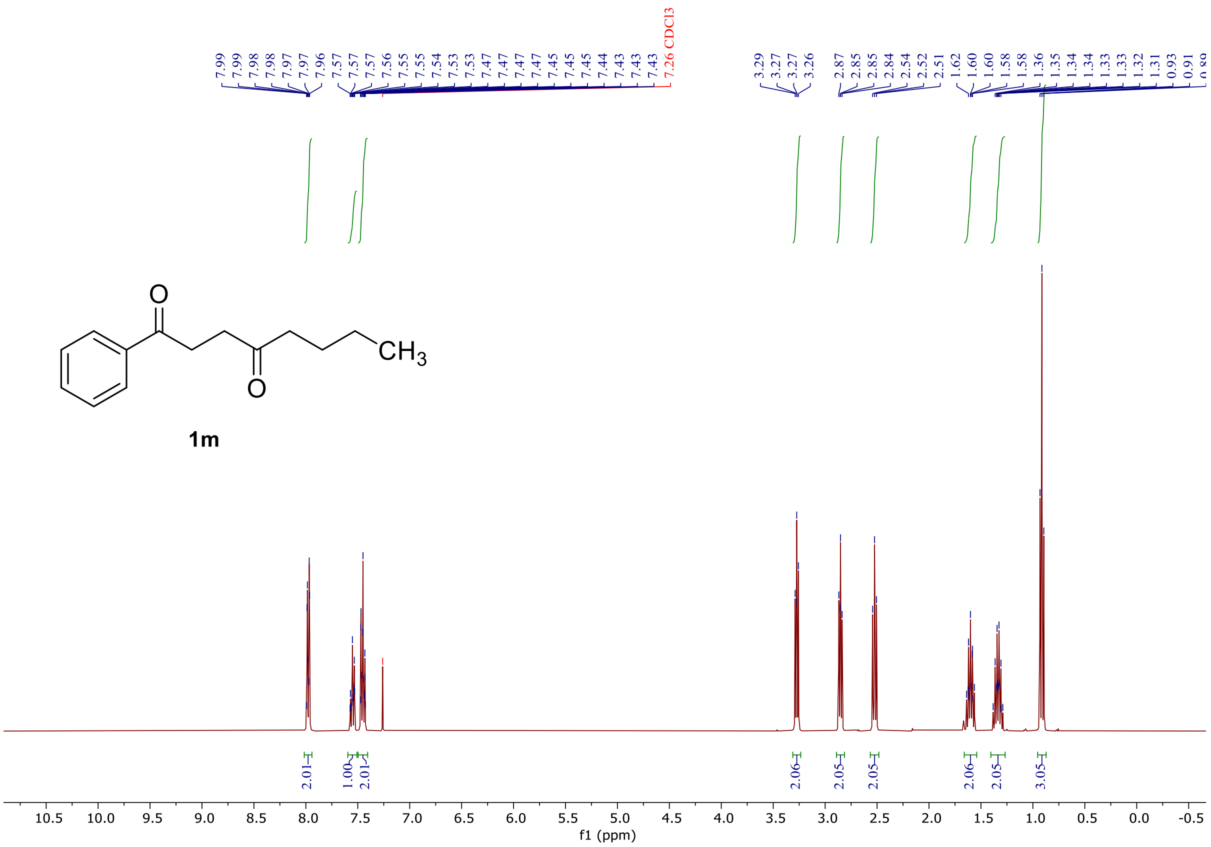

^{13}C{^{1}H} NMR (101 MHz, $CDCl_3$) of **1m**

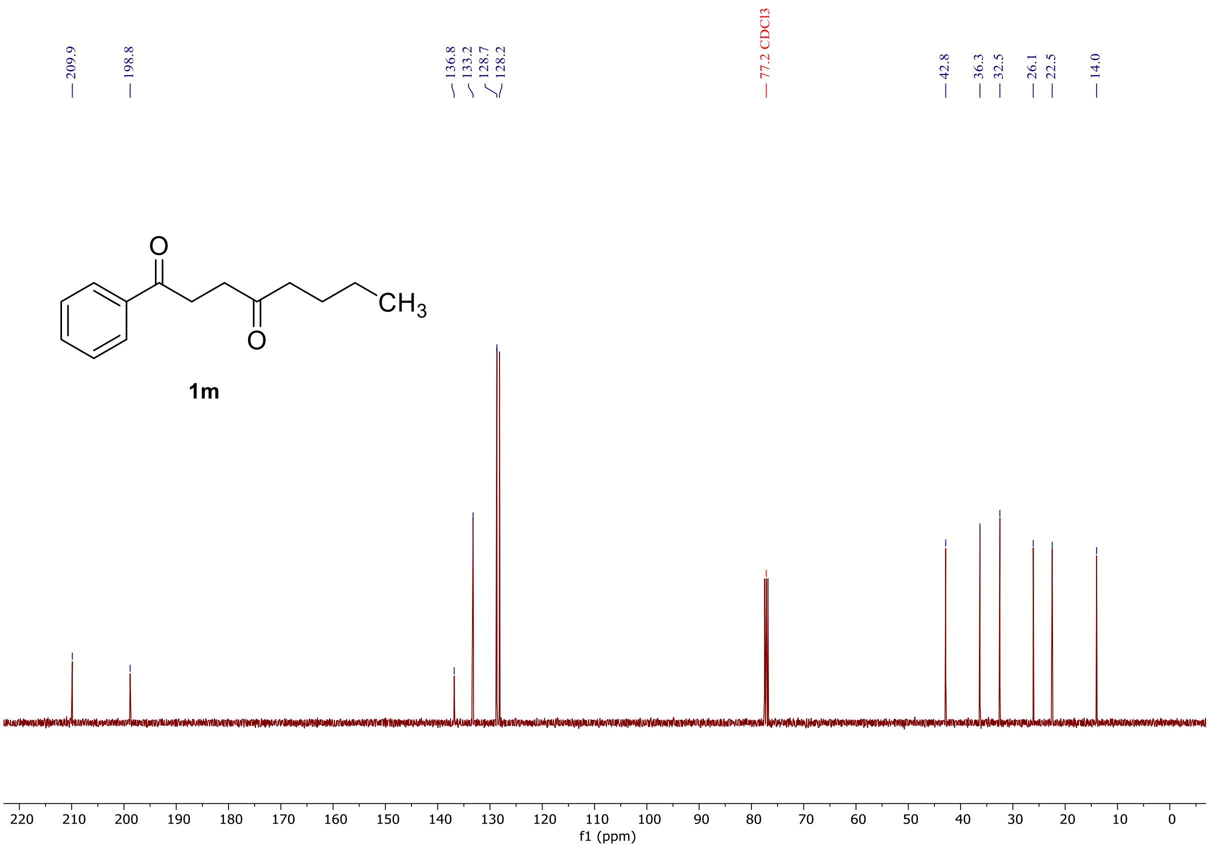

^{1}H NMR (400 MHz, $CDCl_3$) of **1n**

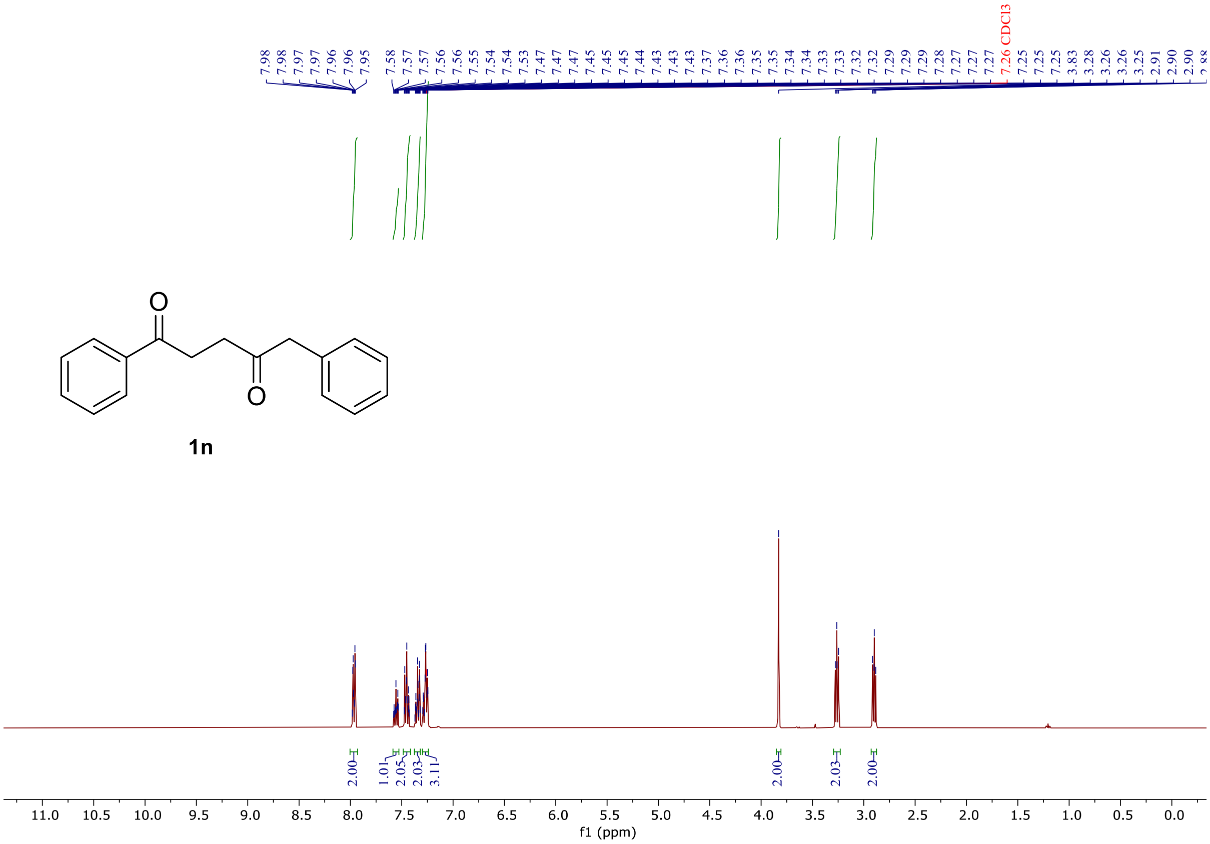

^{13}C{^{1}H} NMR (101 MHz, $CDCl_3$) of **1n**

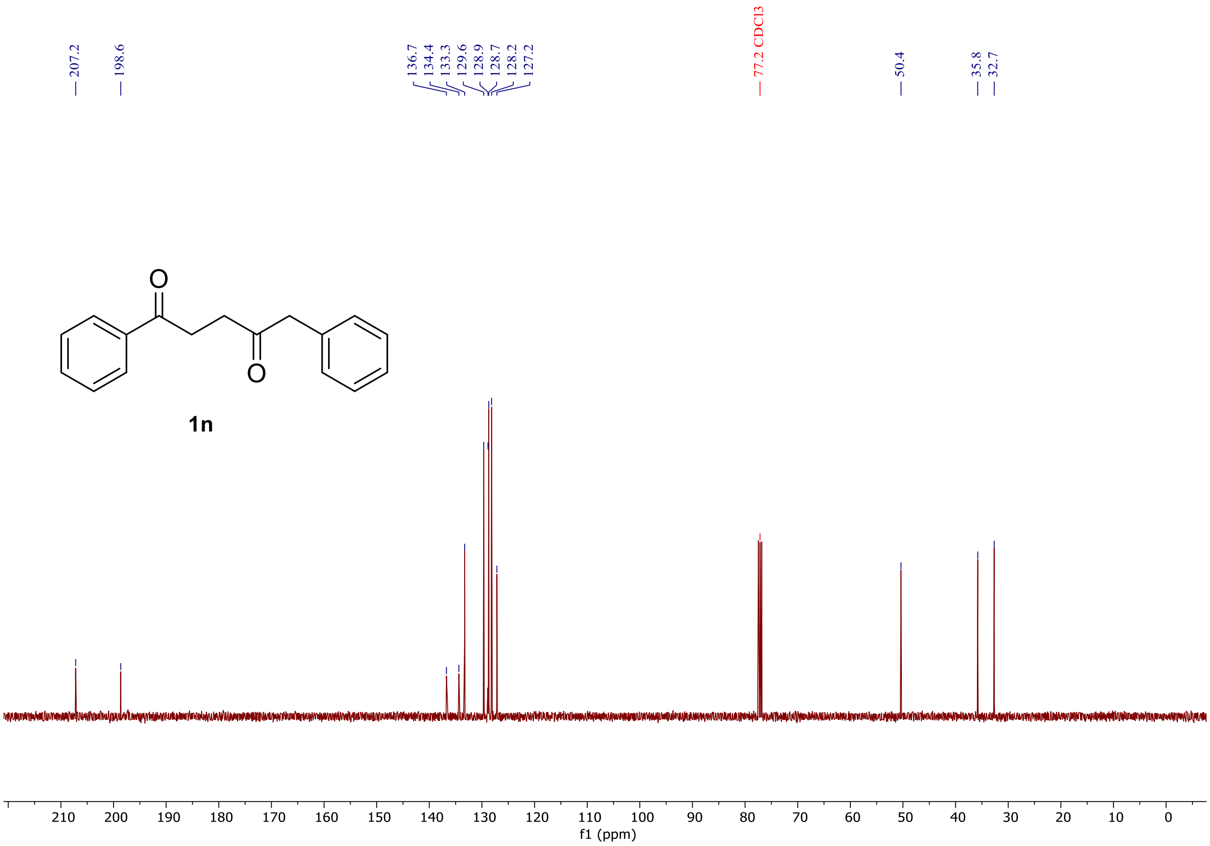

^{1}H NMR (400 MHz, $CDCl_3$) of **1o**

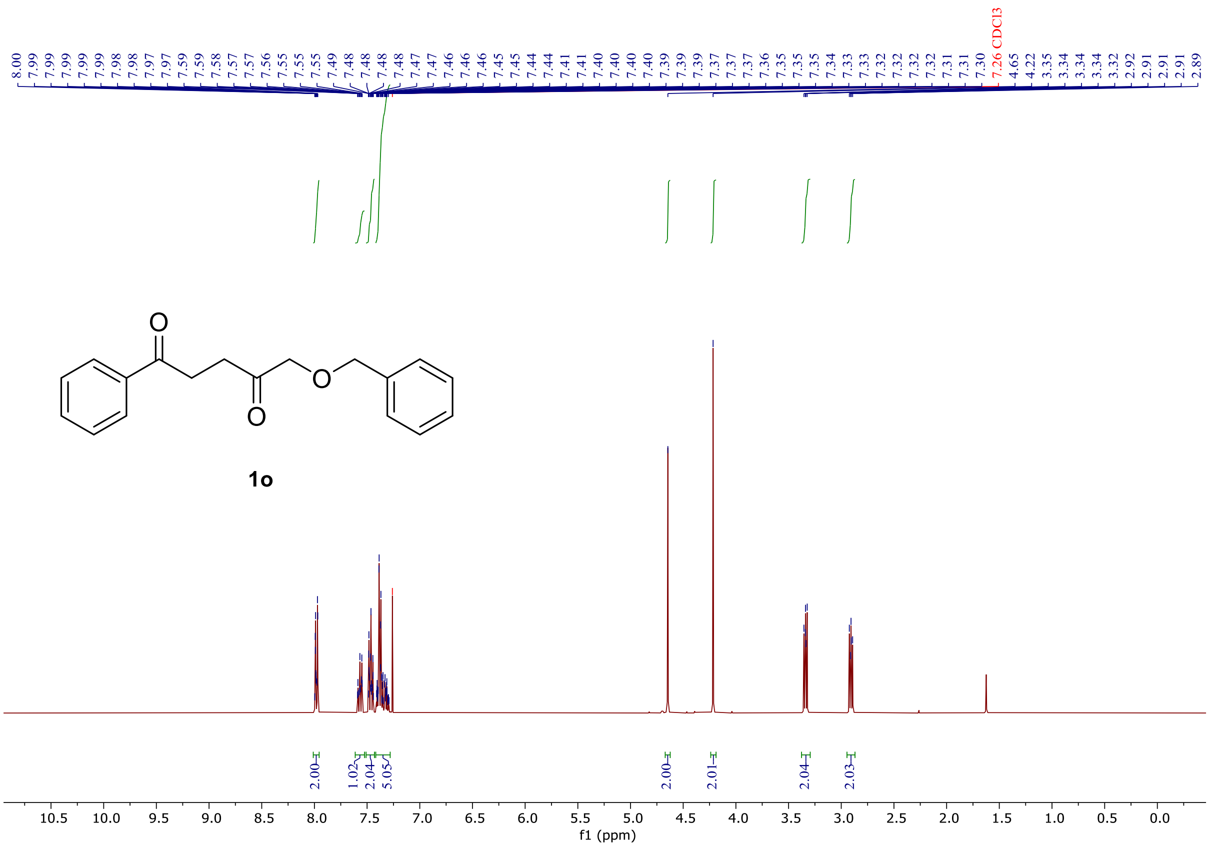

^{13}C{^{1}H} NMR (101 MHz, $CDCl_3$) of **1o**

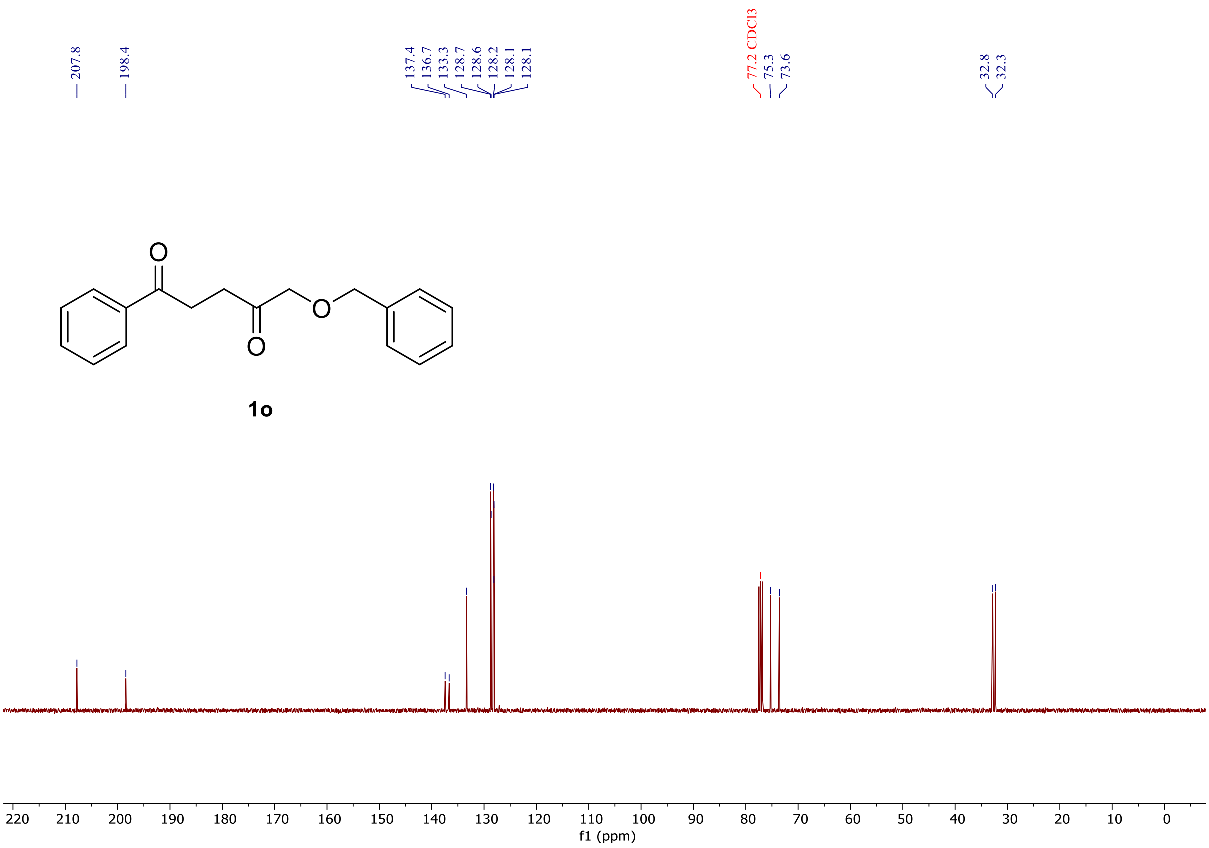

^{1}H NMR (400 MHz, $CDCl_3$) of **1p**

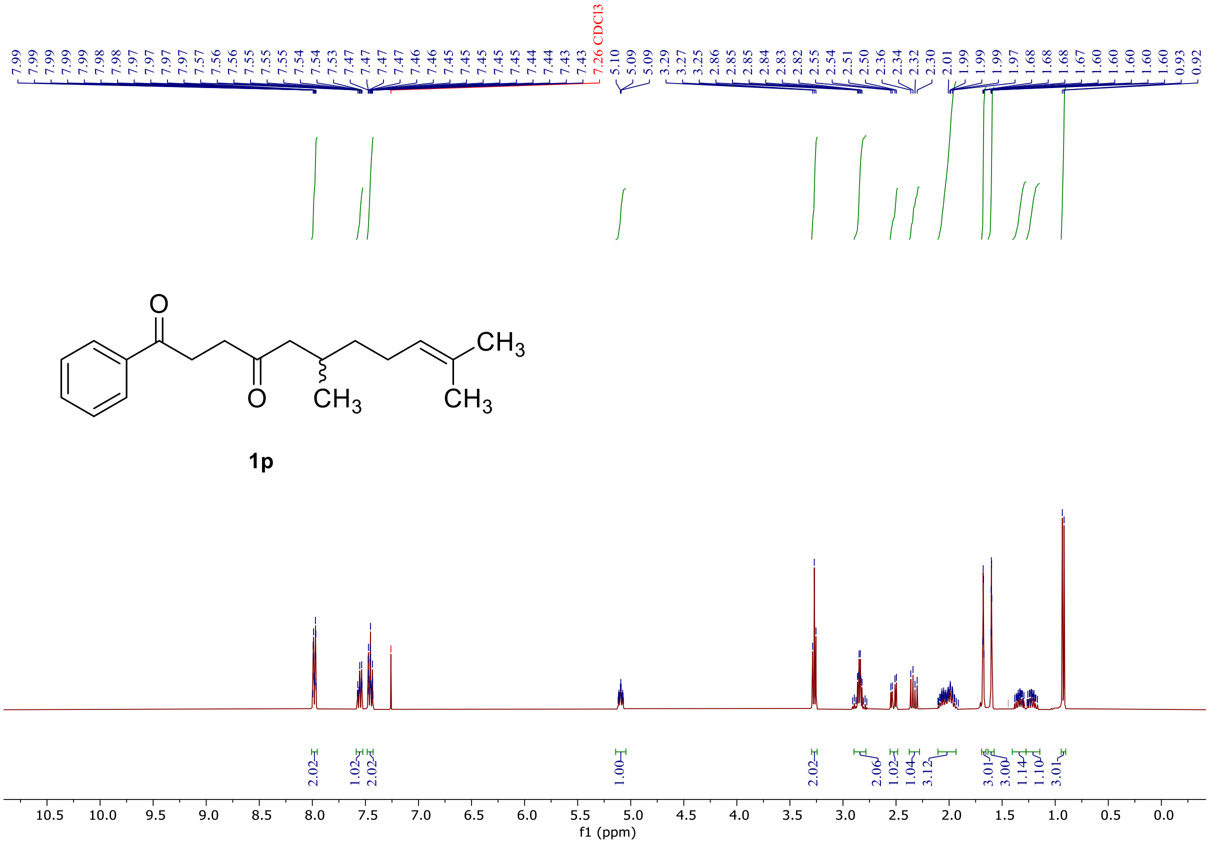

^{13}C{^{1}H} NMR (101 MHz, $CDCl_3$) of **1p**

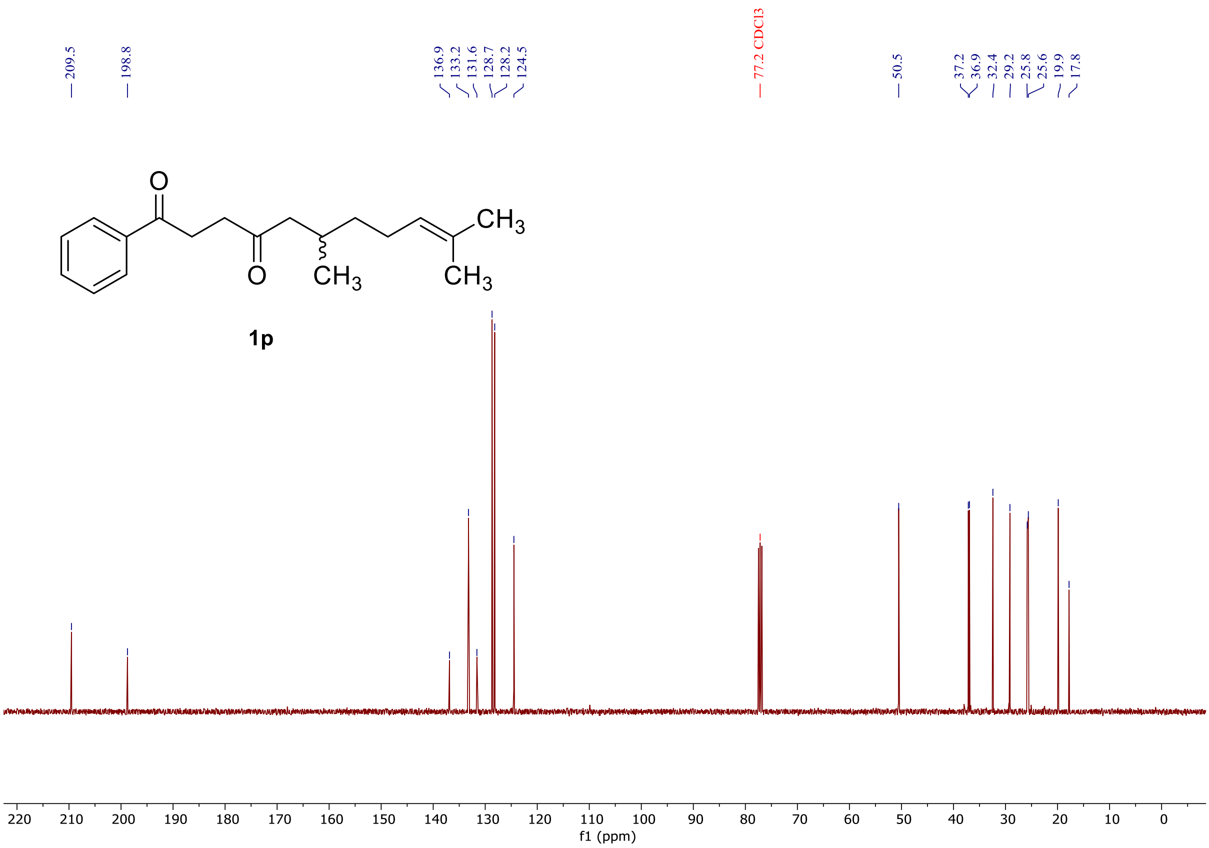

^{1}H NMR (400 MHz, $CDCl_3$) of **1q**

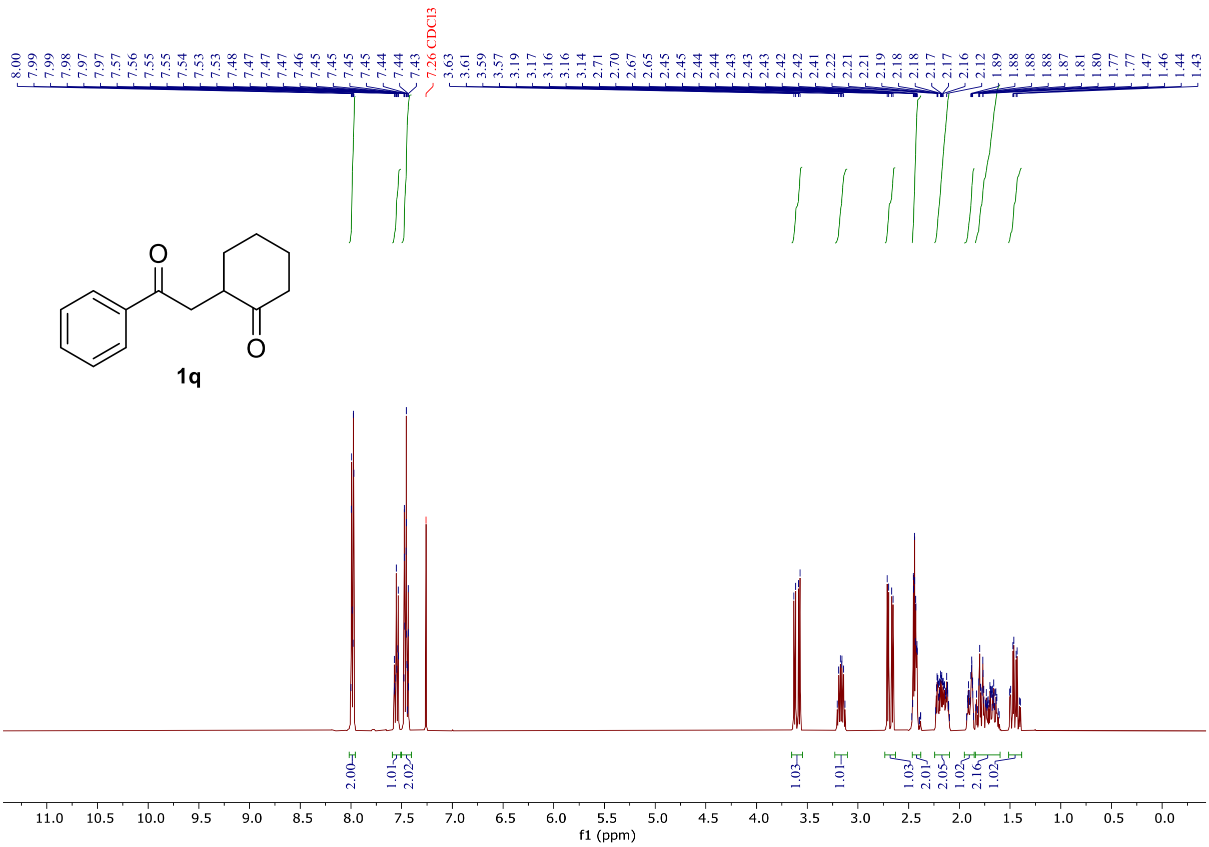

$^{13}C\{^{1}H\}$ NMR (101 MHz, $CDCl_3$) of **1q**

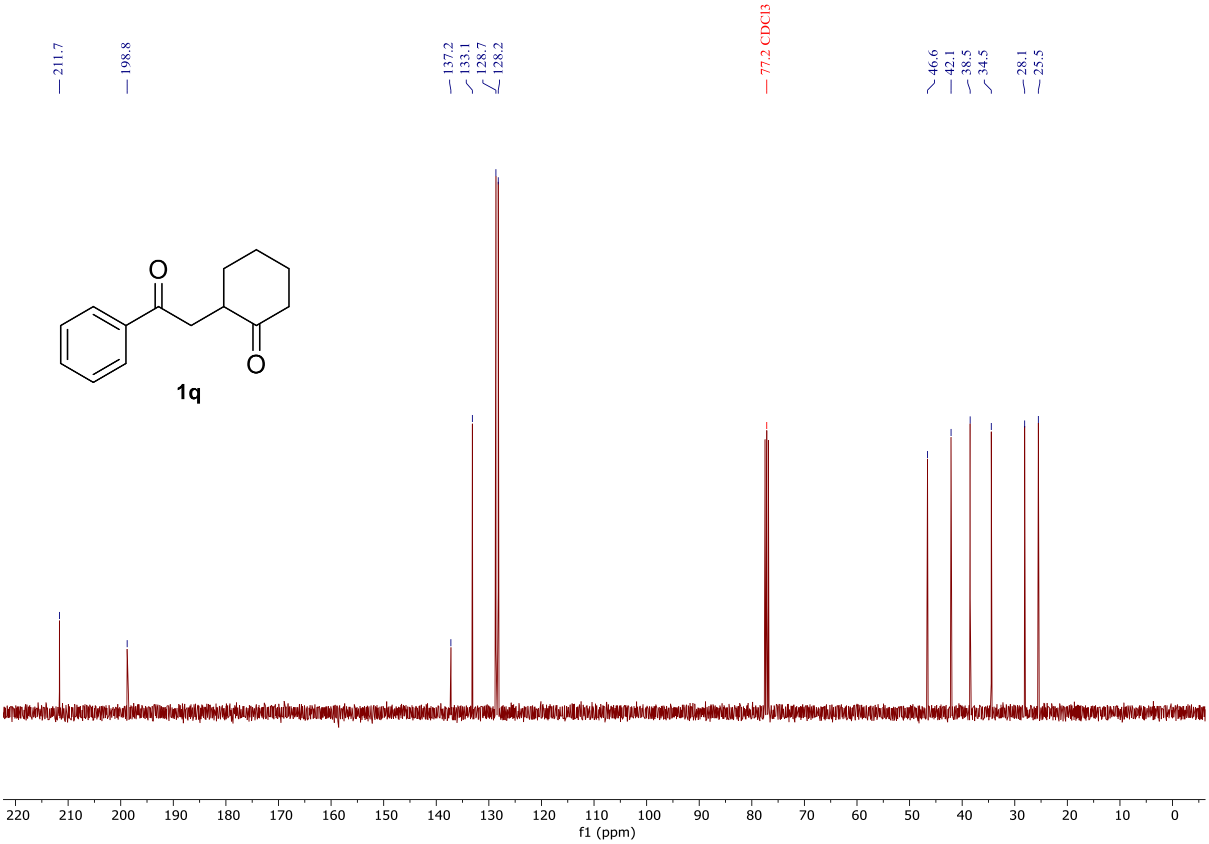

^{1}H NMR (400 MHz, $CDCl_3$) of **1r**

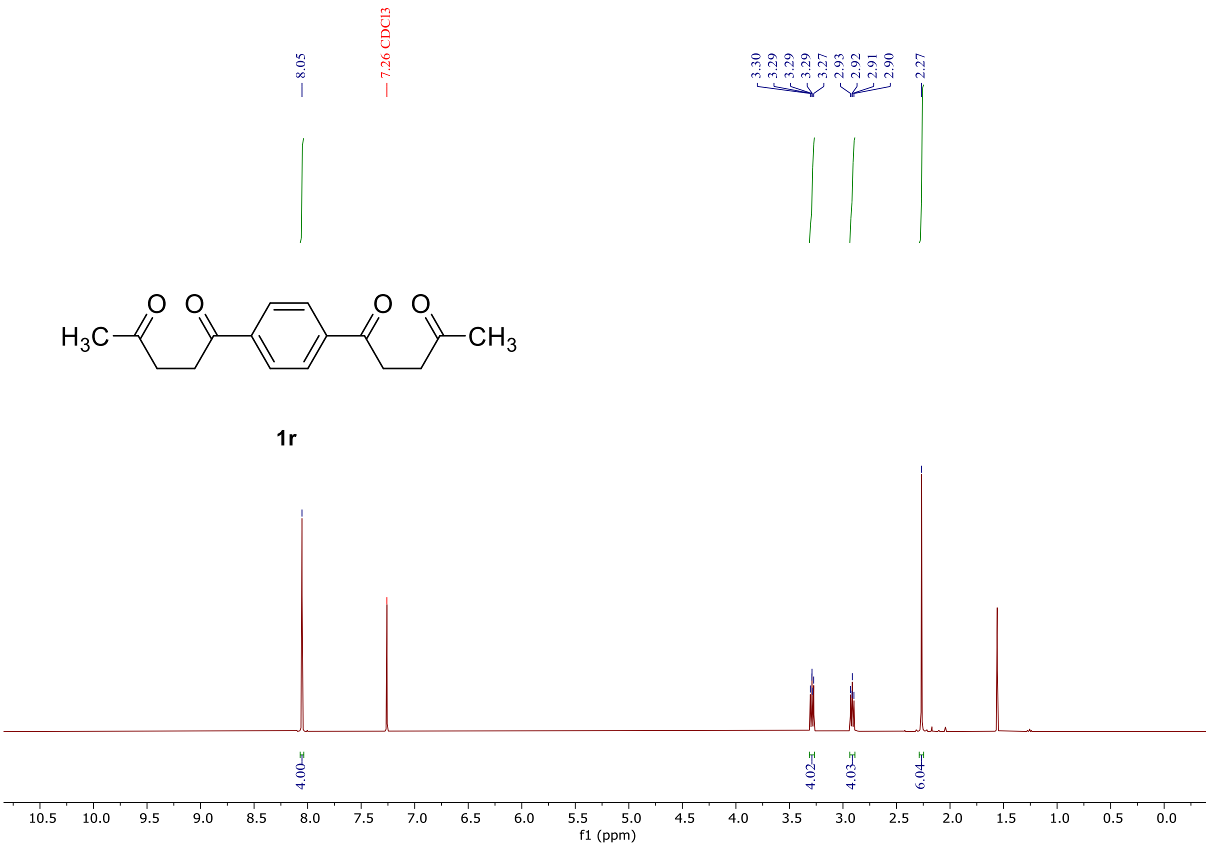

^{13}C{^{1}H} NMR (101 MHz, $CDCl_3$) of **1r**

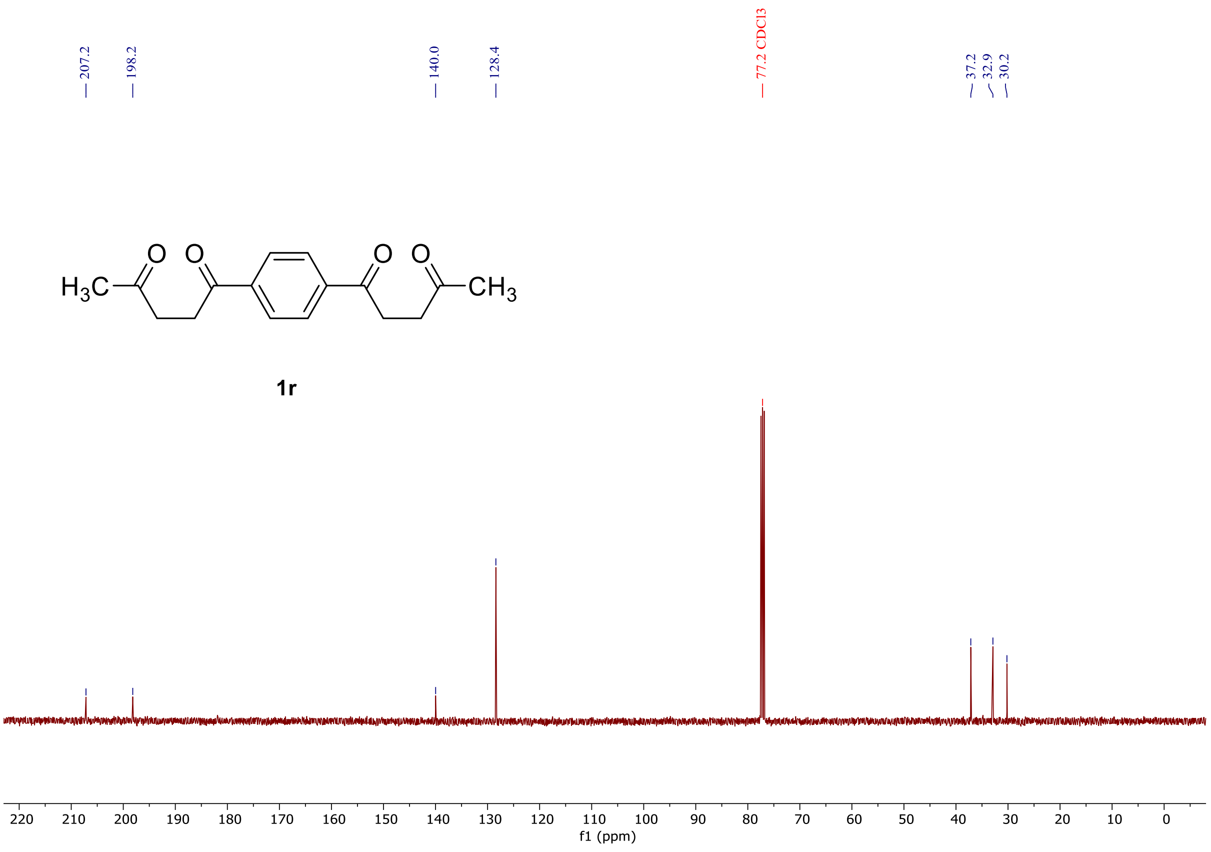

^{1}H NMR (500 MHz, $CDCl_3$) of **1s**

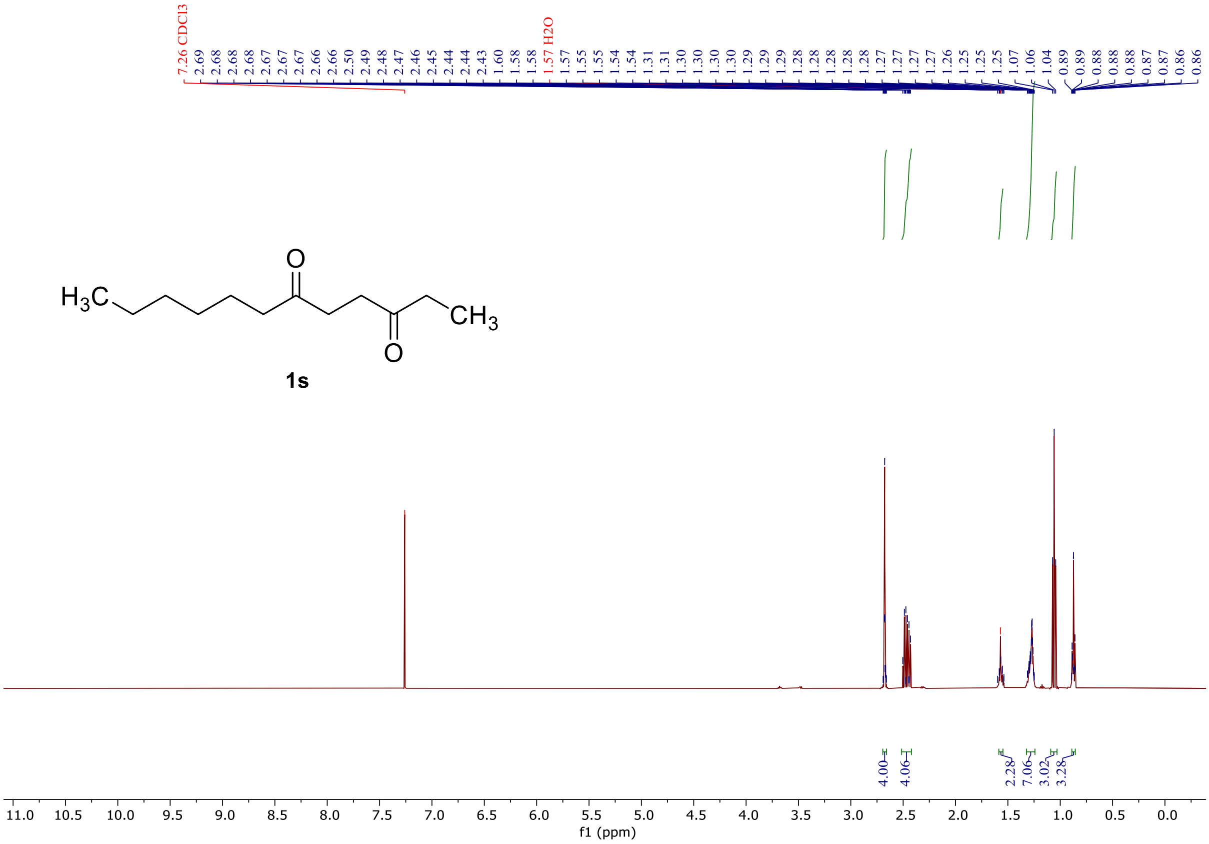

$^{13}C\{^{1}H\}$ NMR (126 MHz, $CDCl_3$) of **1s**

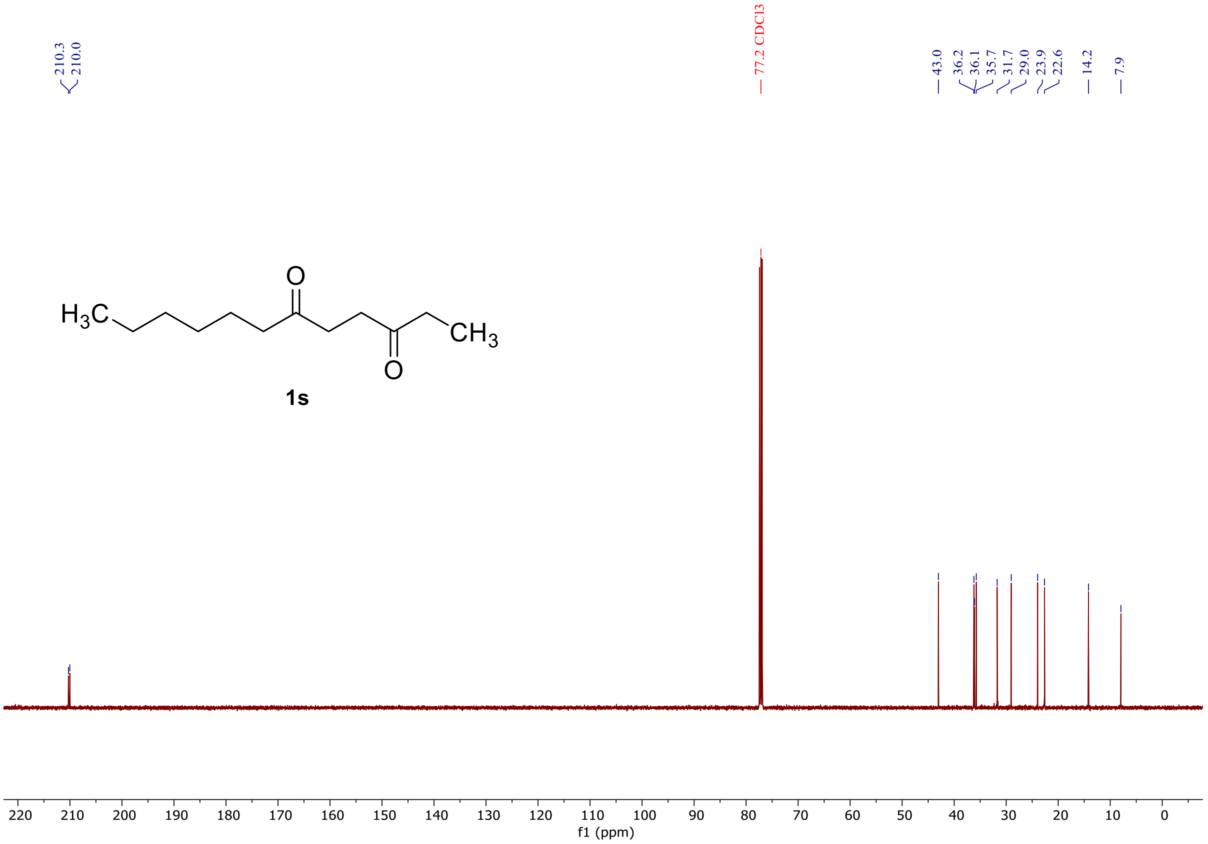

^{1}H NMR (400 MHz, $CDCl_3$) of **1t**

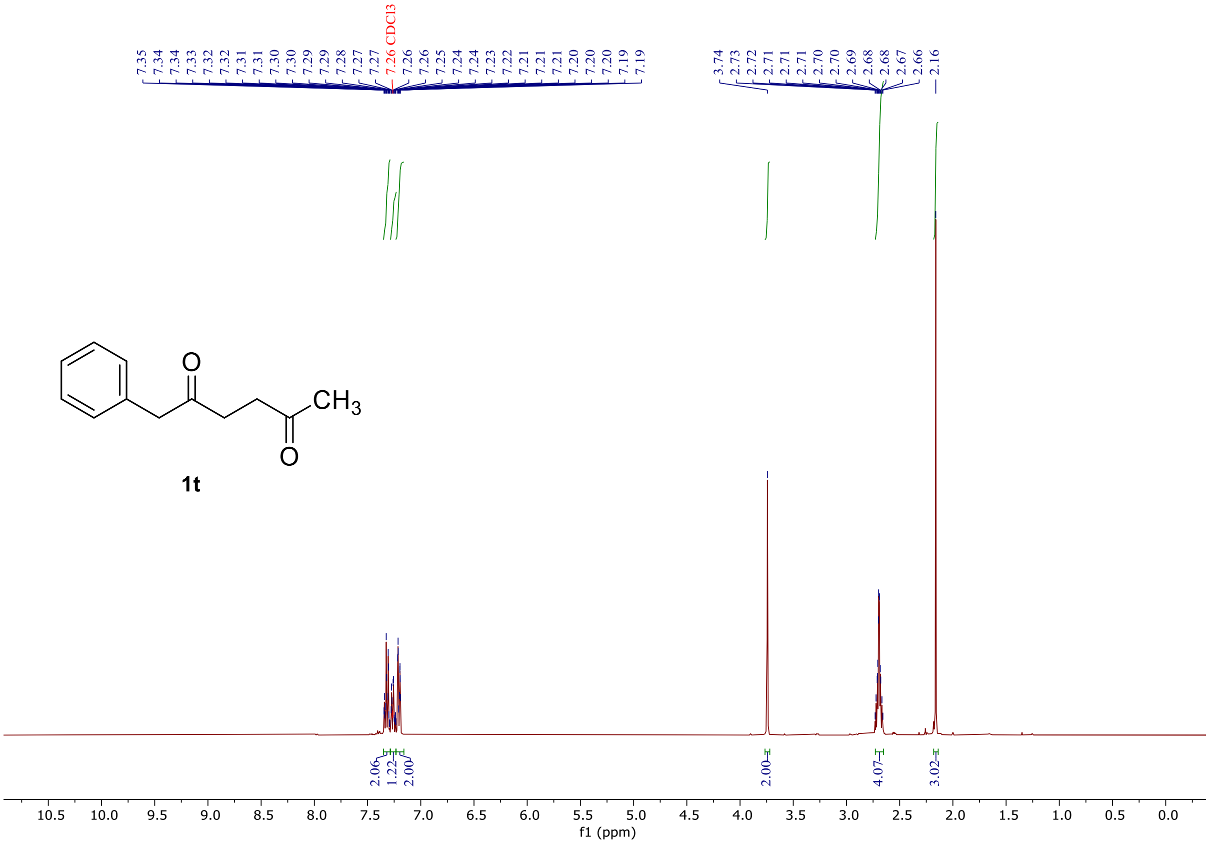

^{13}C{^{1}H} NMR (101 MHz, $CDCl_3$) of **1t**

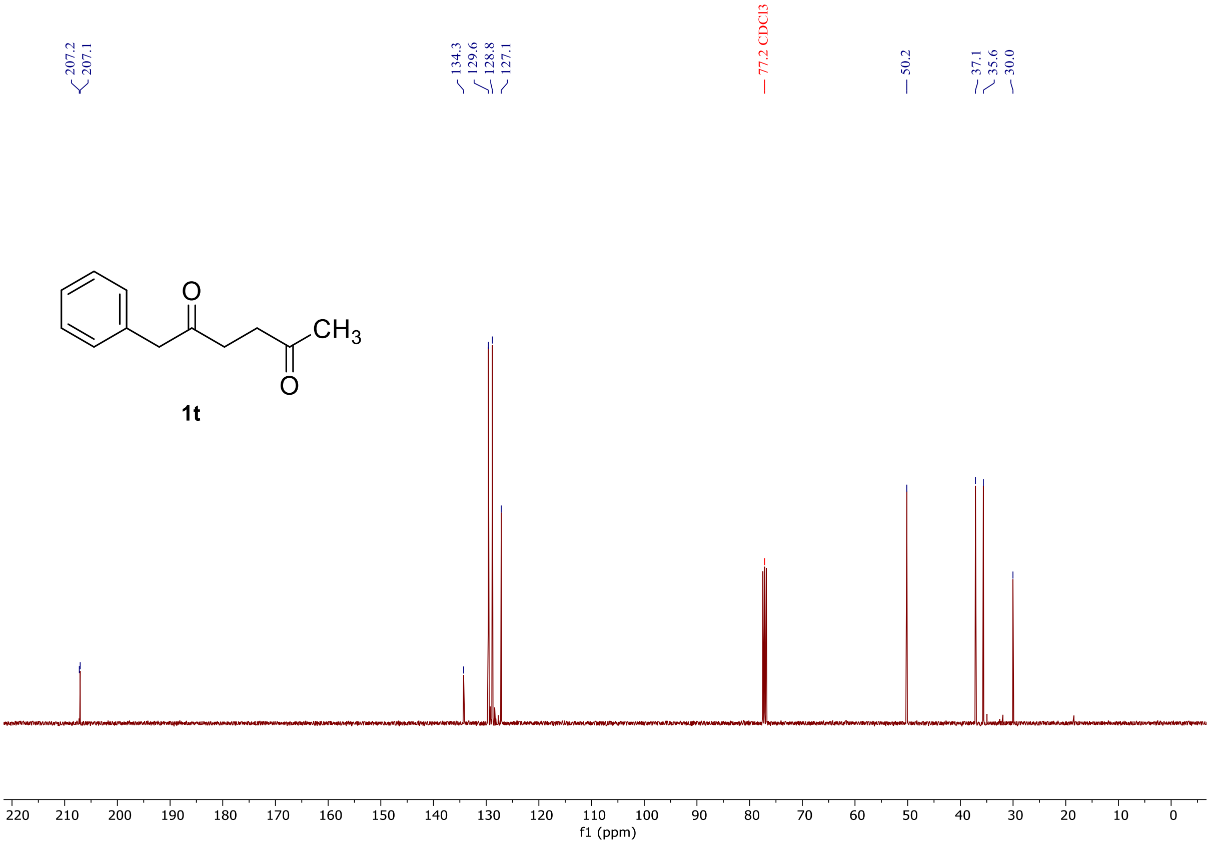

^{1}H NMR (400 MHz, $CDCl_3$) of **1u**

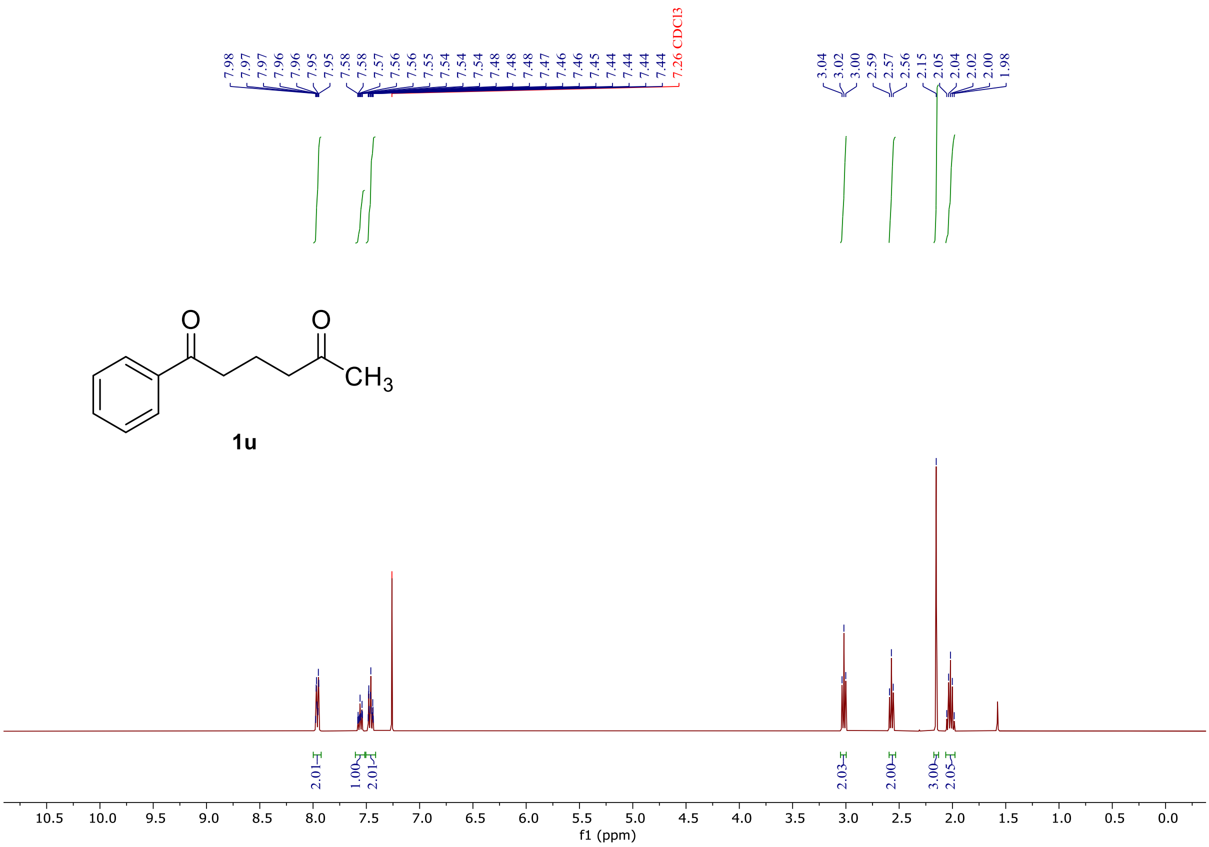

$^{13}C\{^{1}H\}$ NMR (101 MHz, $CDCl_3$) of **1u**

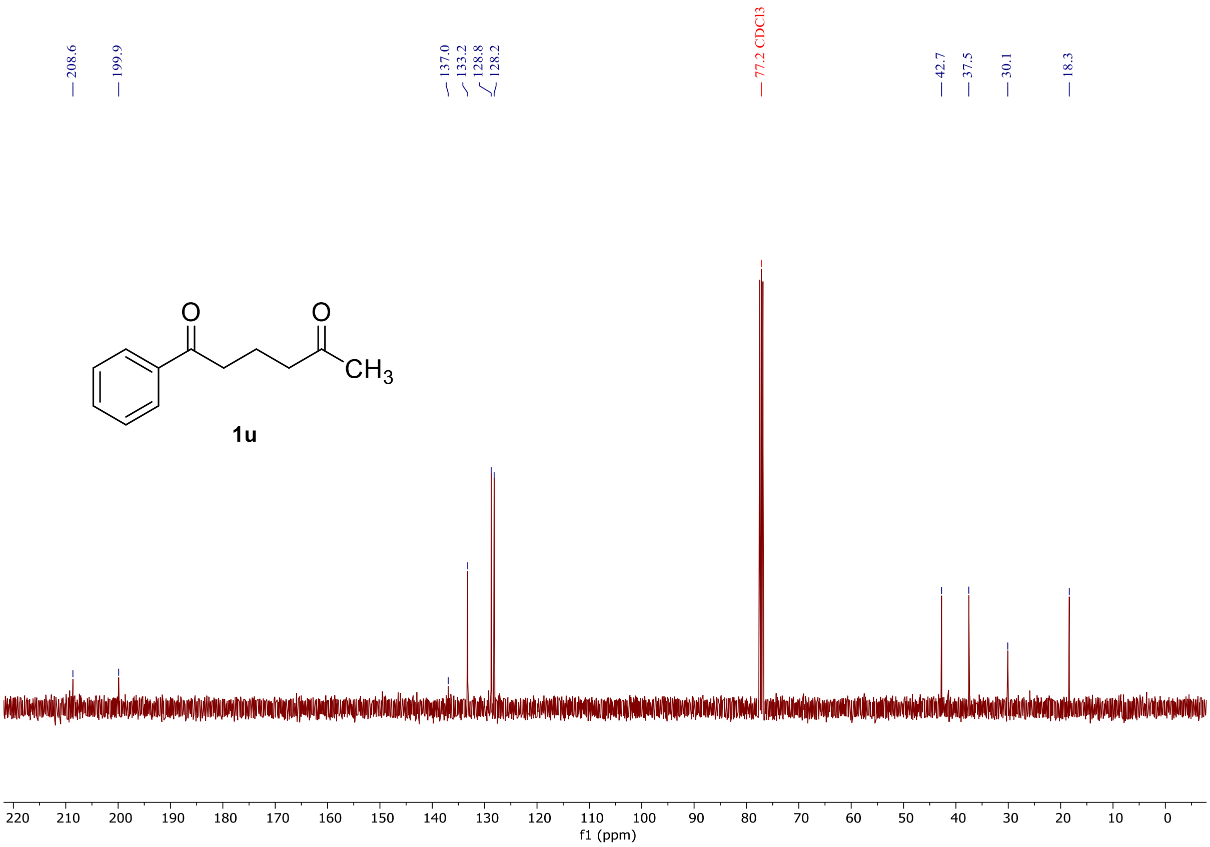

^{1}H NMR (400 MHz, $CDCl_3$) of **1v**

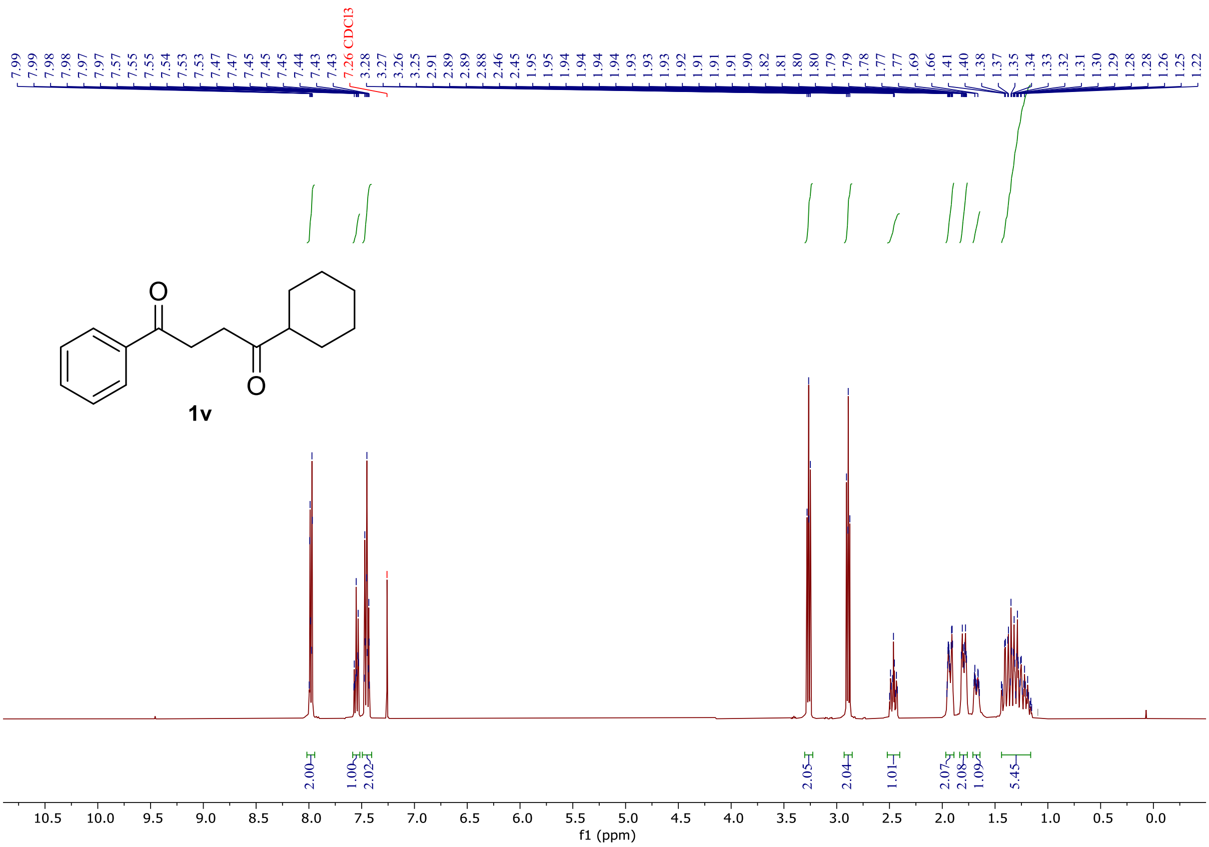

^{13}C{^{1}H} NMR (101 MHz, $CDCl_3$) of **1v**

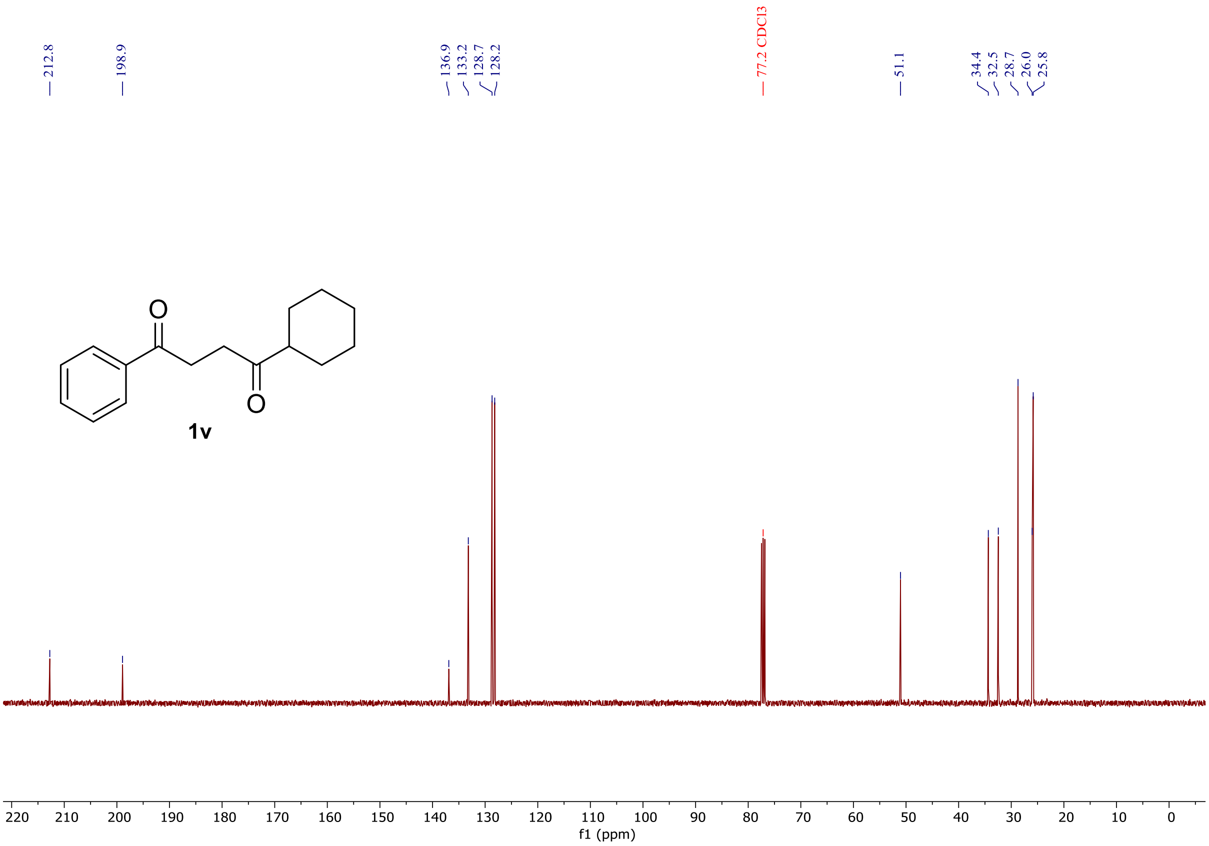

^{1}H NMR (500 MHz, $CDCl_3$) of **1w**

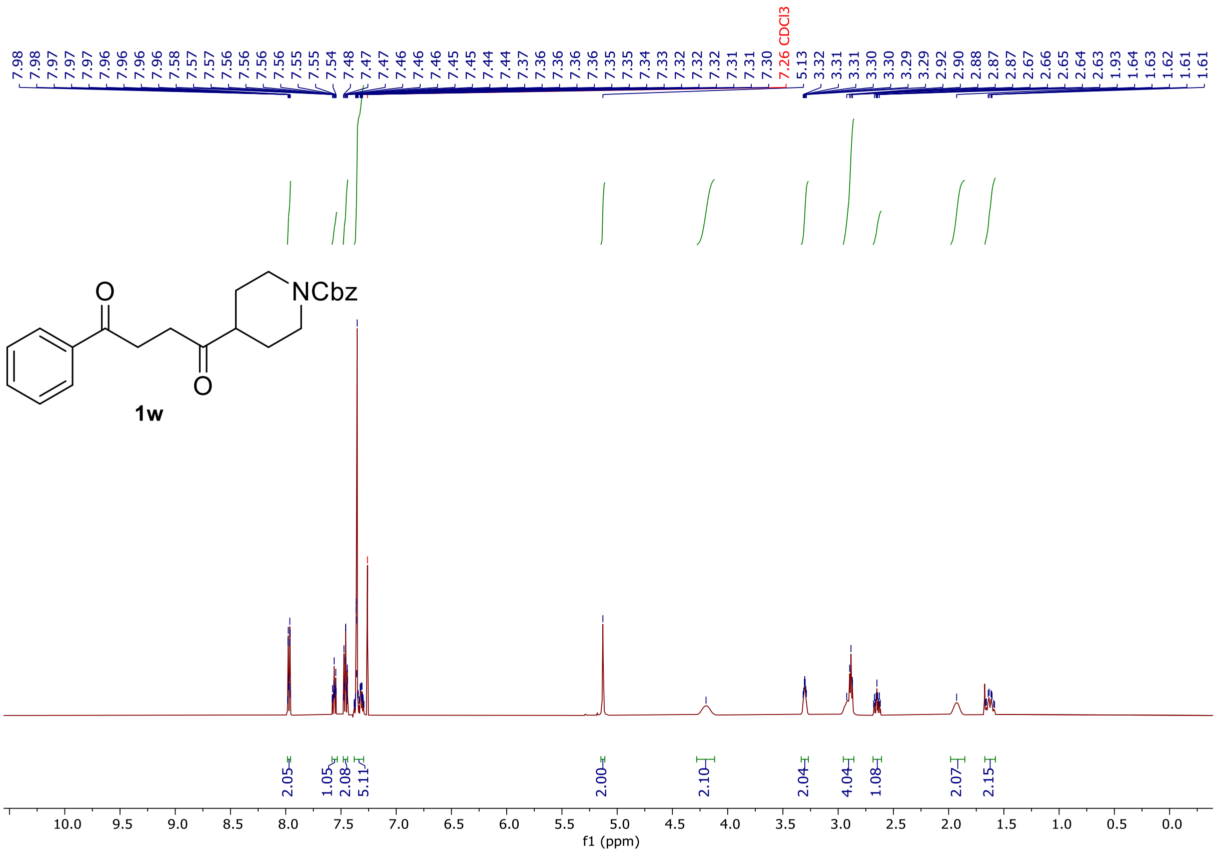

^{13}C{^{1}H} NMR (126 MHz, $CDCl_3$) of **1w**

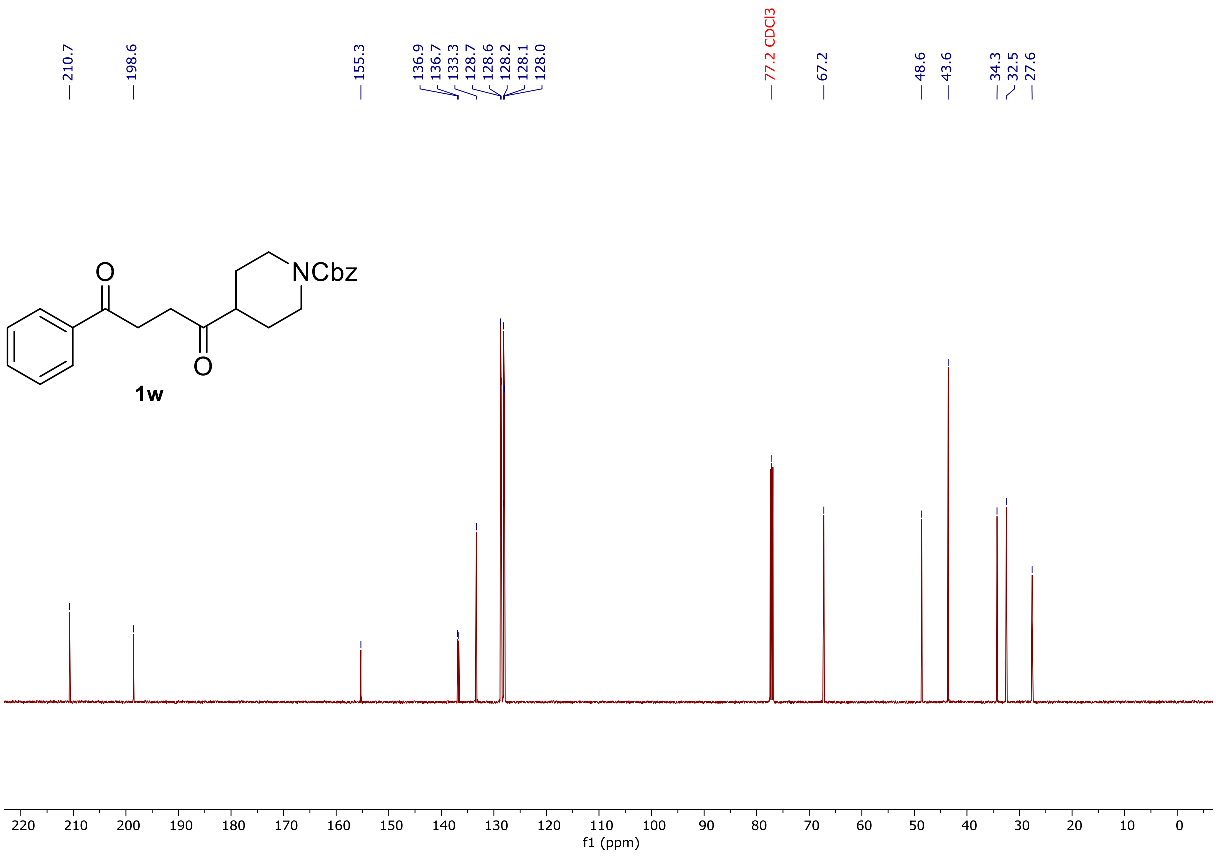

^{1}H NMR (400 MHz, $CDCl_3$) of **1x**

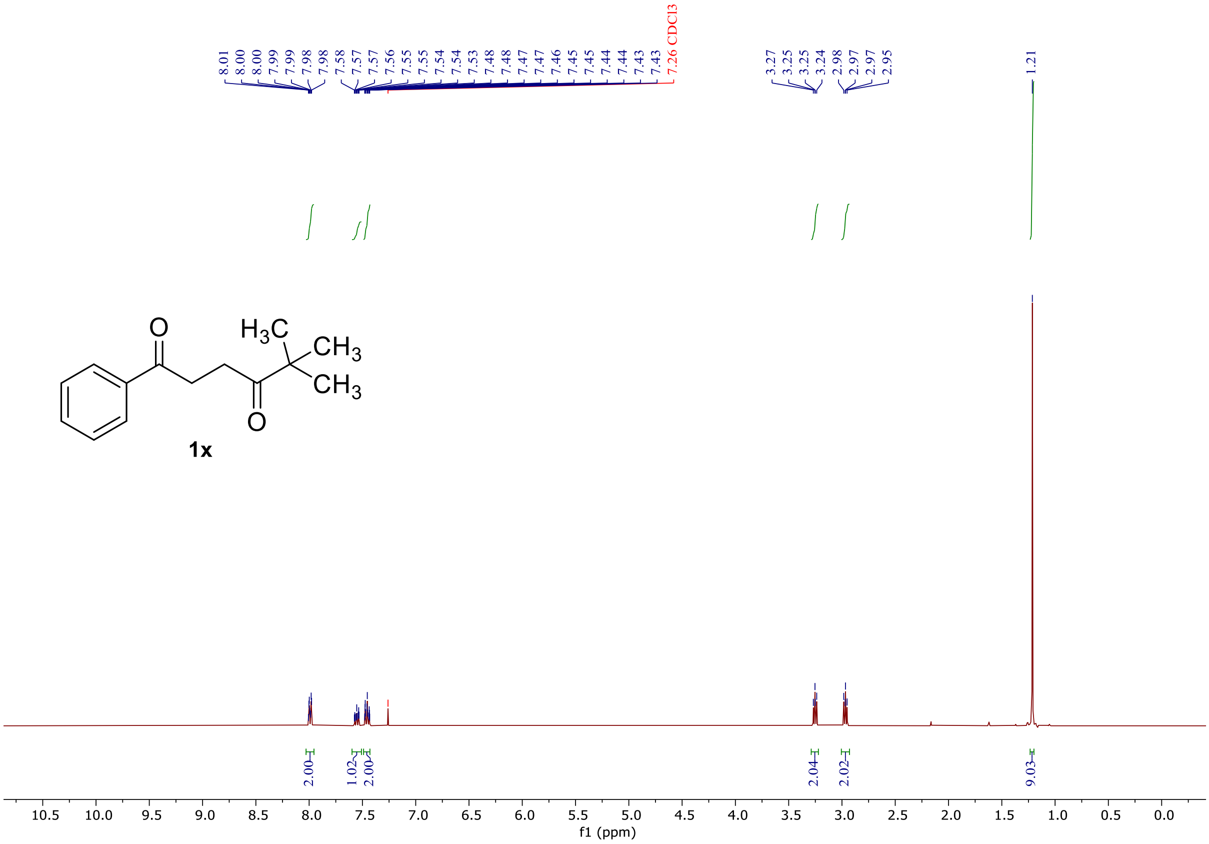

$^{13}C\{^{1}H\}$ NMR (101 MHz, $CDCl_3$) of **1x**

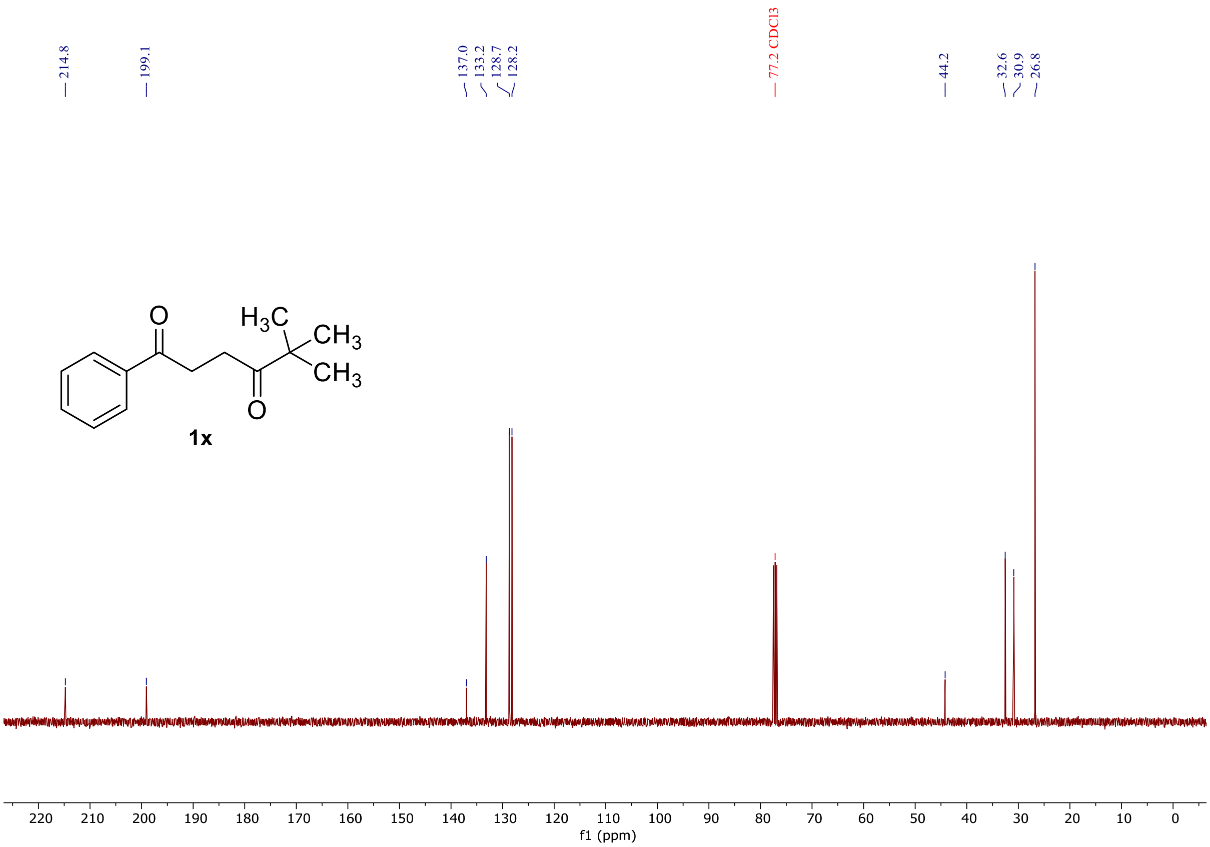

^{1}H NMR (400 MHz, $CDCl_3$) of **1z**

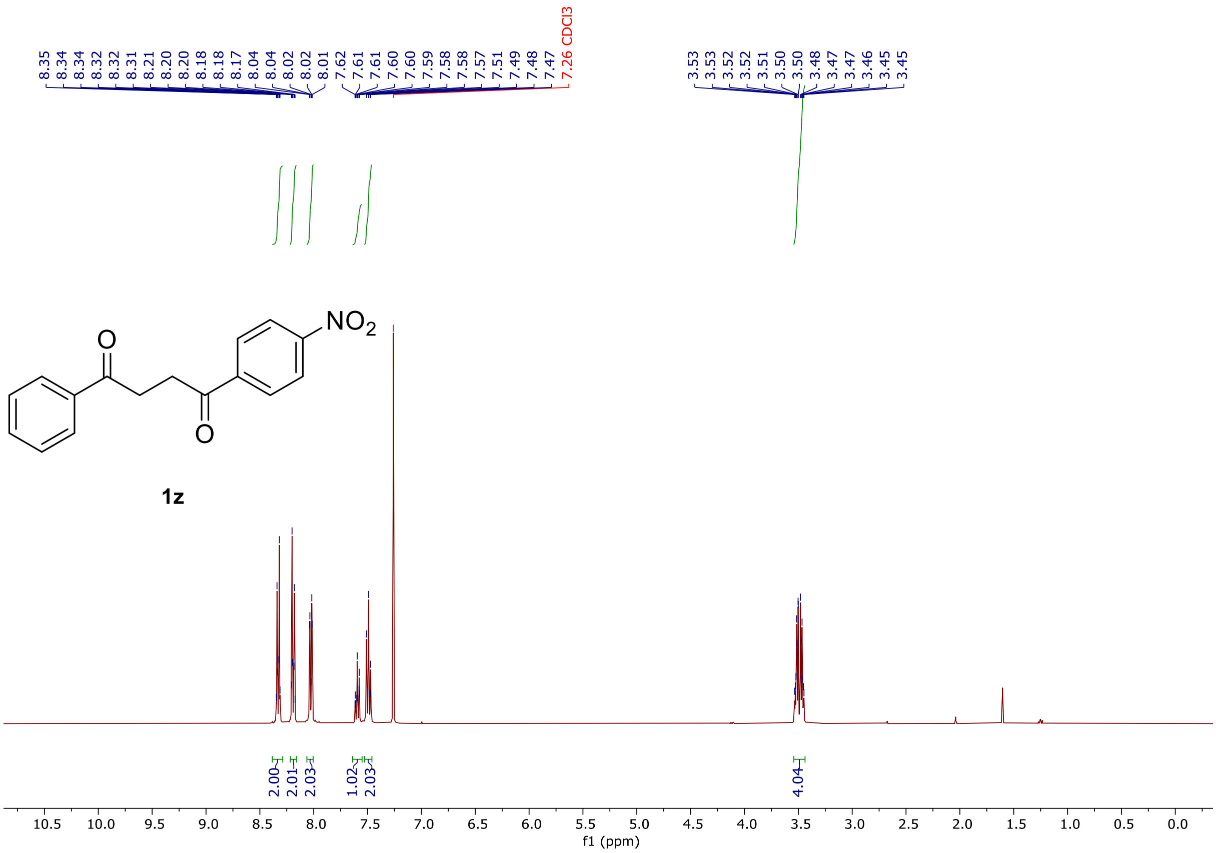

$^{13}C\{^{1}H\}$ NMR (101 MHz, $CDCl_3$) of **1z**

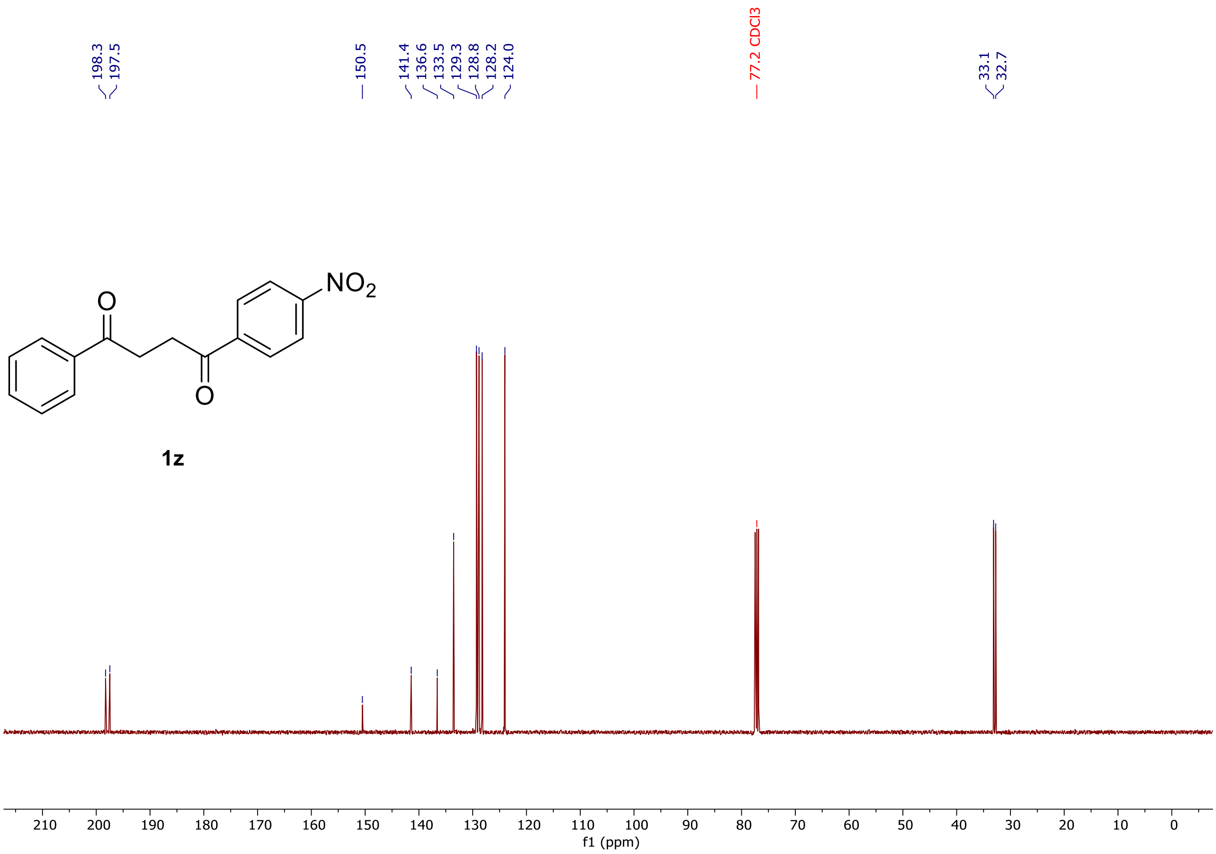

^{1}H NMR (400 MHz, $CDCl_3$) of **1aa**

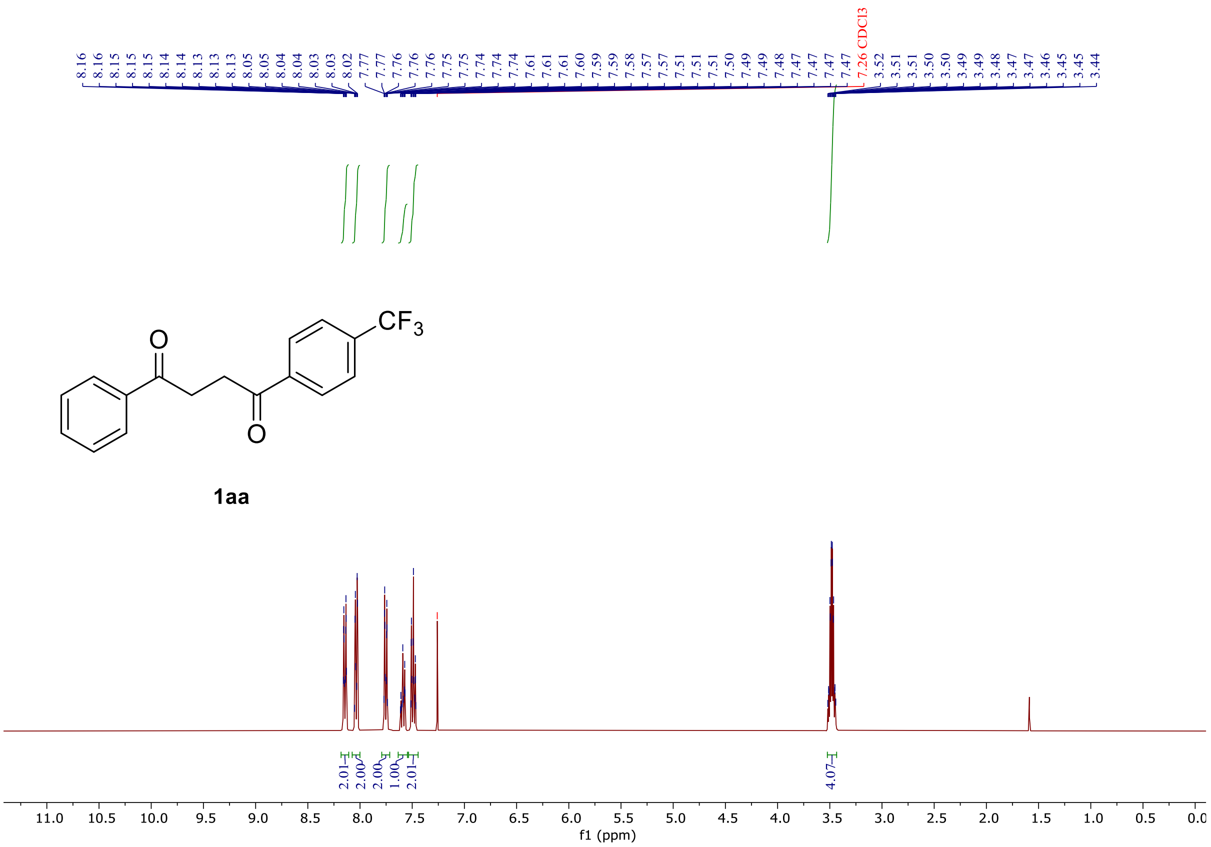

^{13}C{^{1}H} NMR (101 MHz, $CDCl_3$) of **1aa**

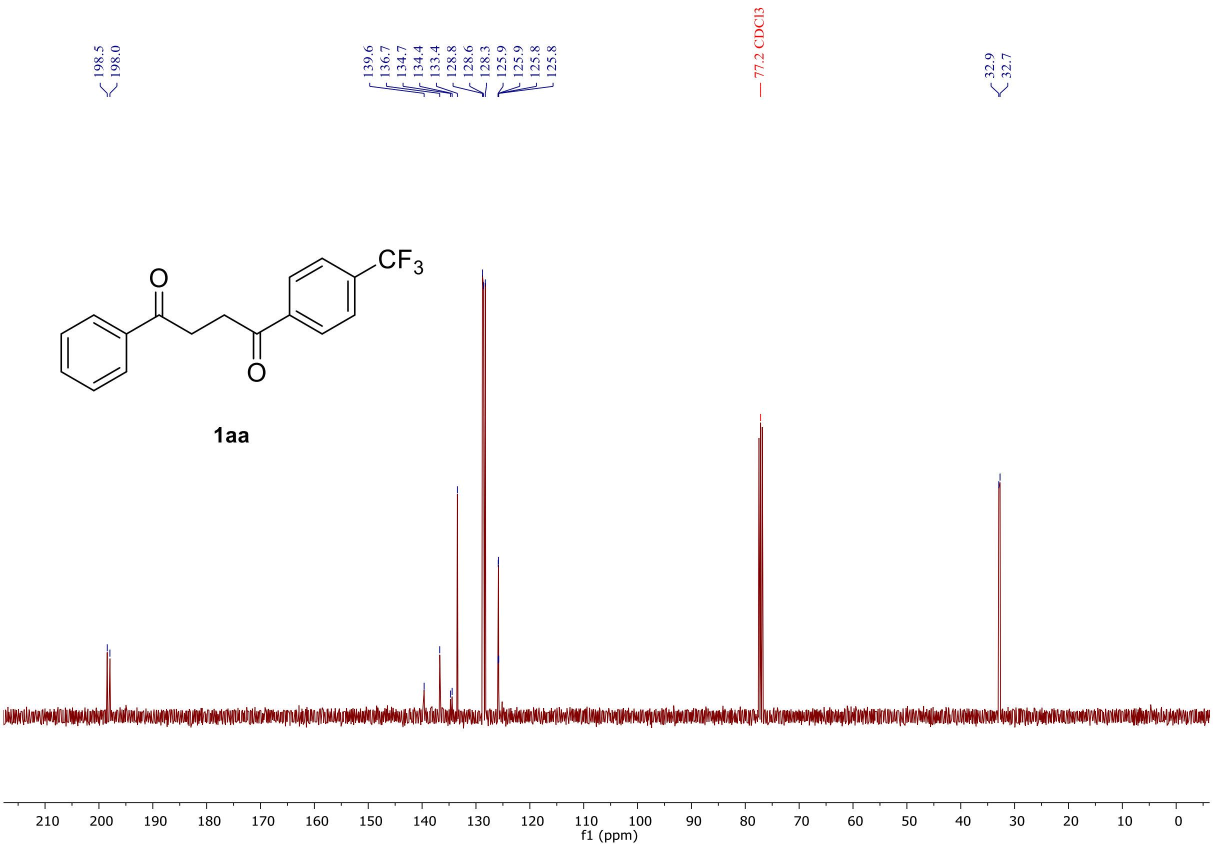

$^{19}F\{^1H\}$ NMR (377 MHz, $CDCl_3$) of **1aa**

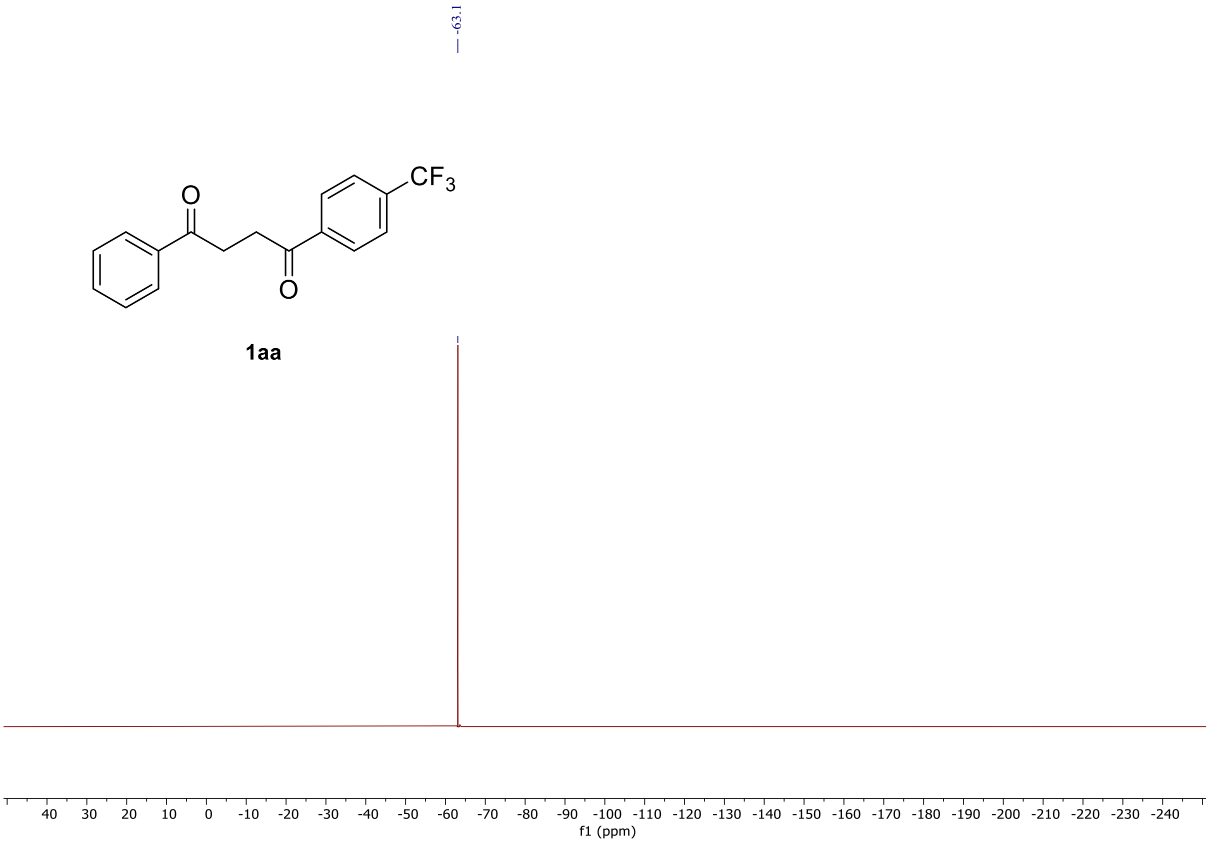

^{1}H NMR (400 MHz, $CDCl_3$) of **1ab**

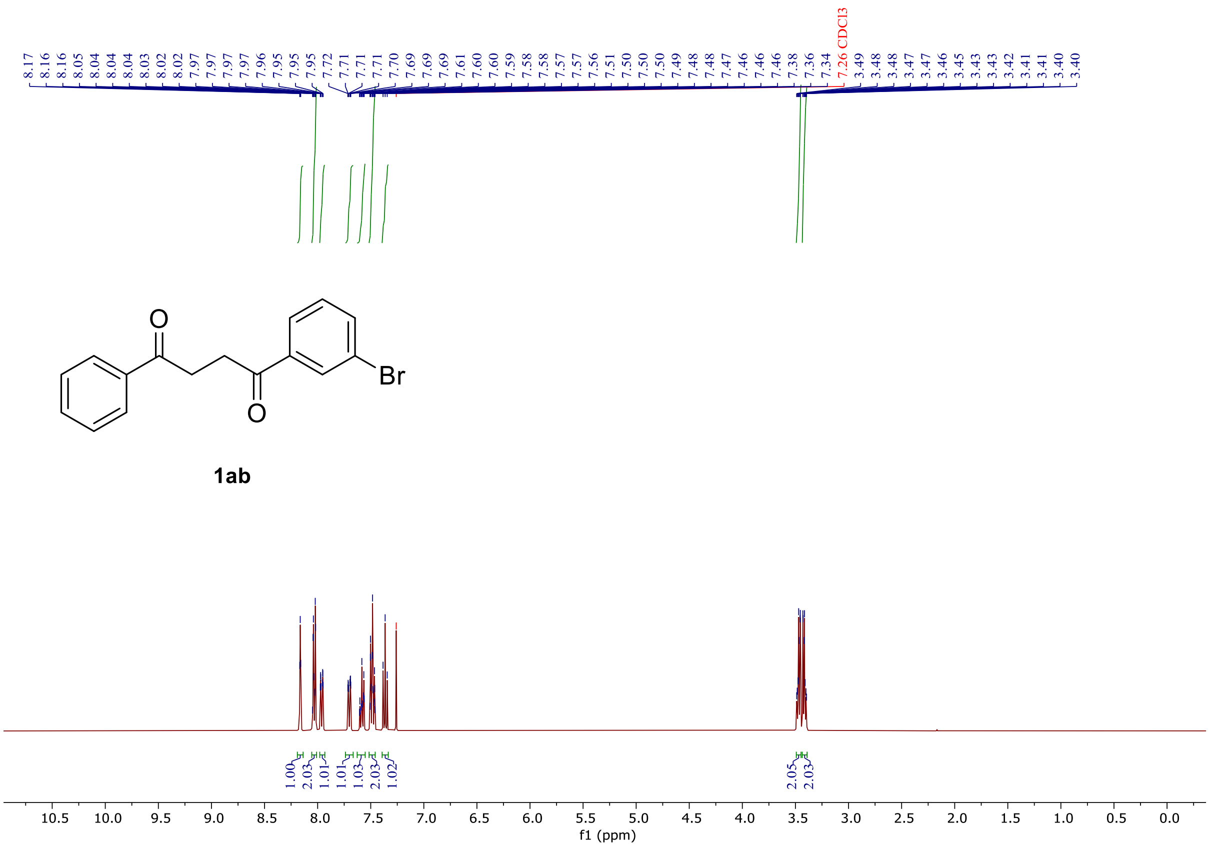

^{13}C{^{1}H} NMR (101 MHz, $CDCl_3$) of **1ab**

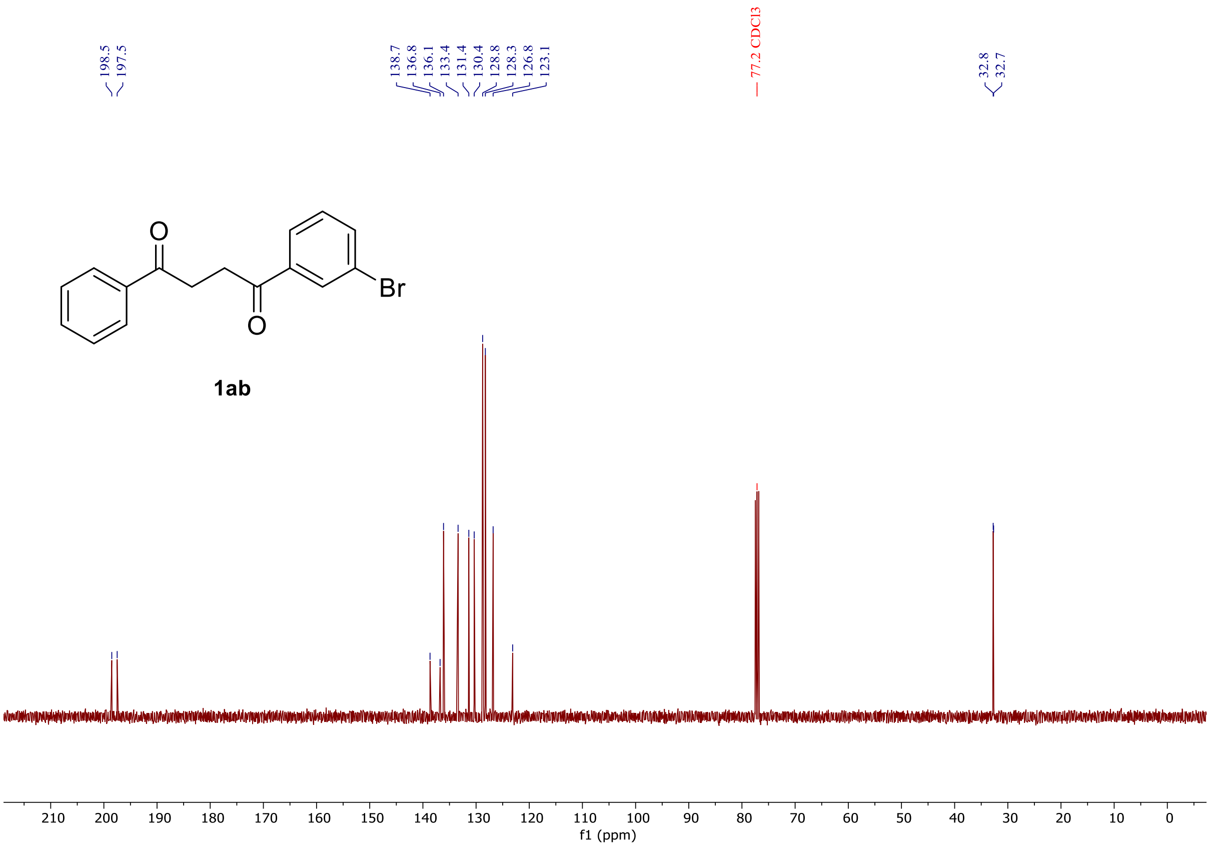

^{1}H NMR (400 MHz, $CDCl_3$) of **1ac**

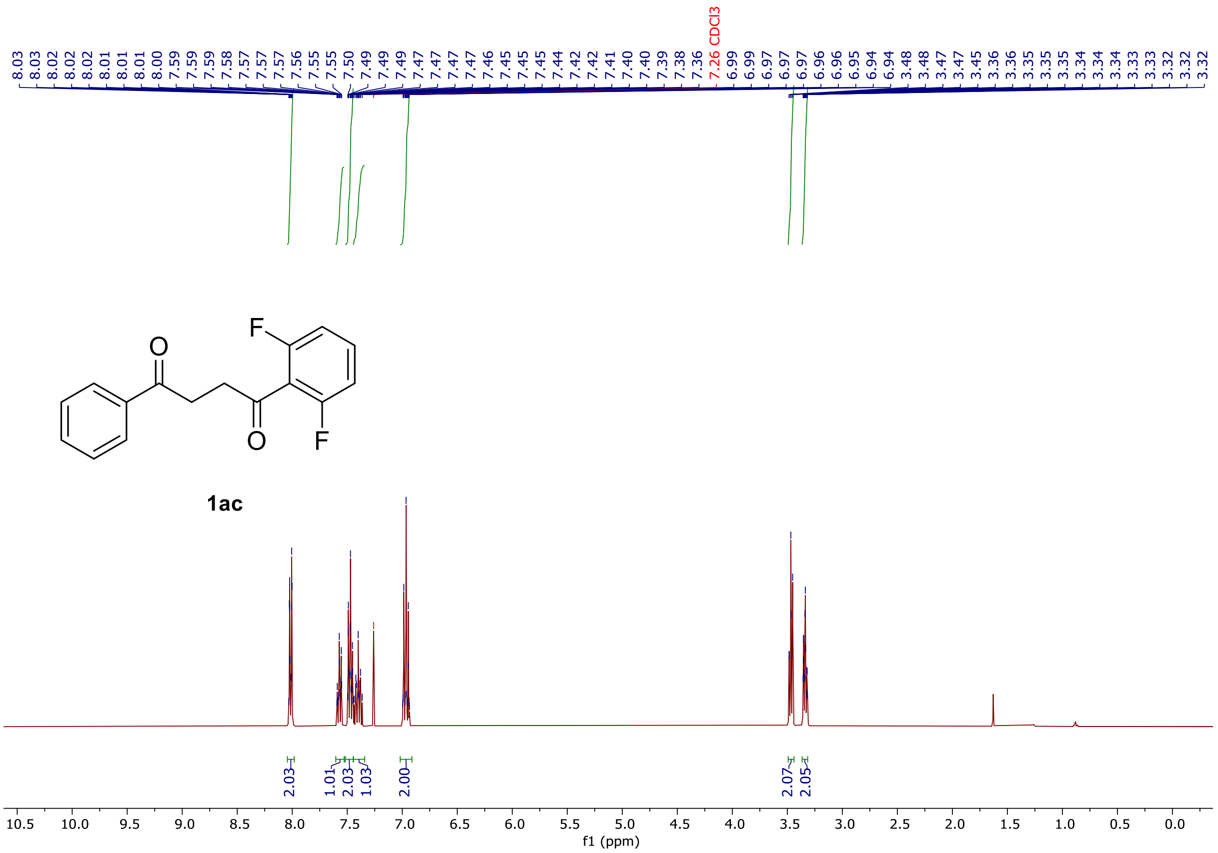

$^{13}C\{^{1}H\}$ NMR (101 MHz, $CDCl_3$) of **1ac**

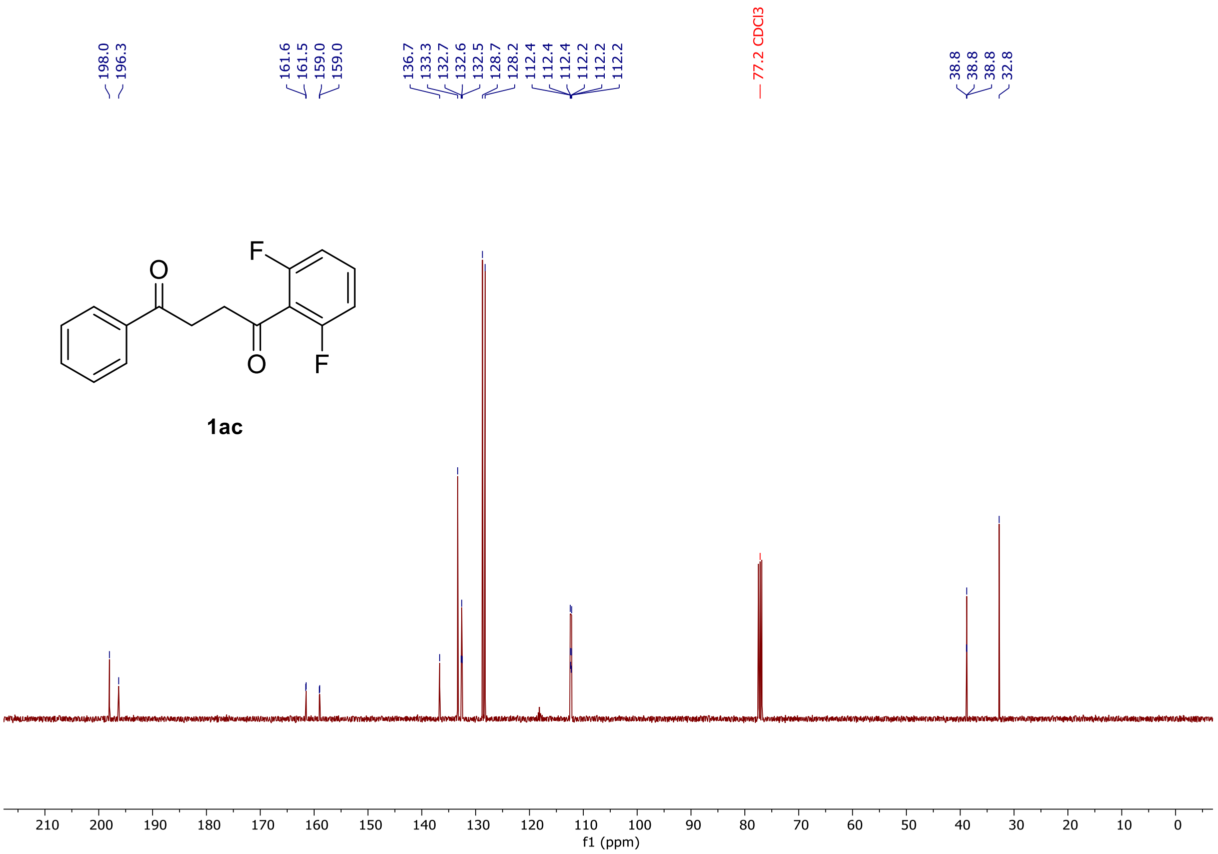

$^{19}F\{^{1}H\}$ NMR (377 MHz, $CDCl_3$) of **1ac**

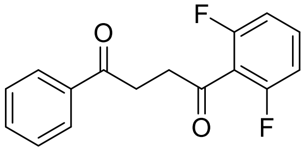

1ac

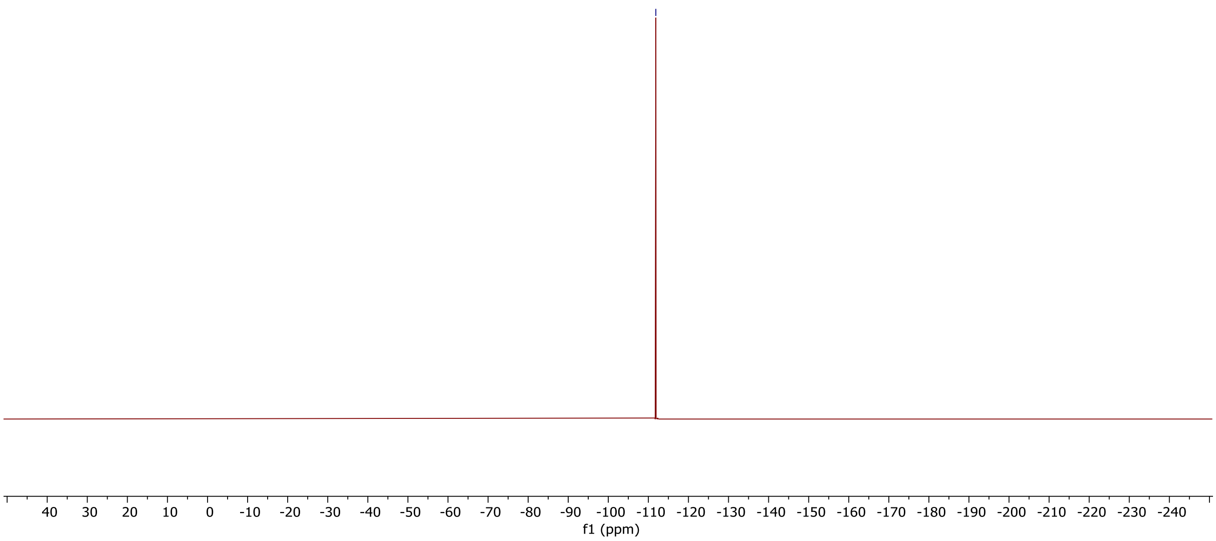

^{1}H NMR (400 MHz, $CDCl_3$) of **1ad**

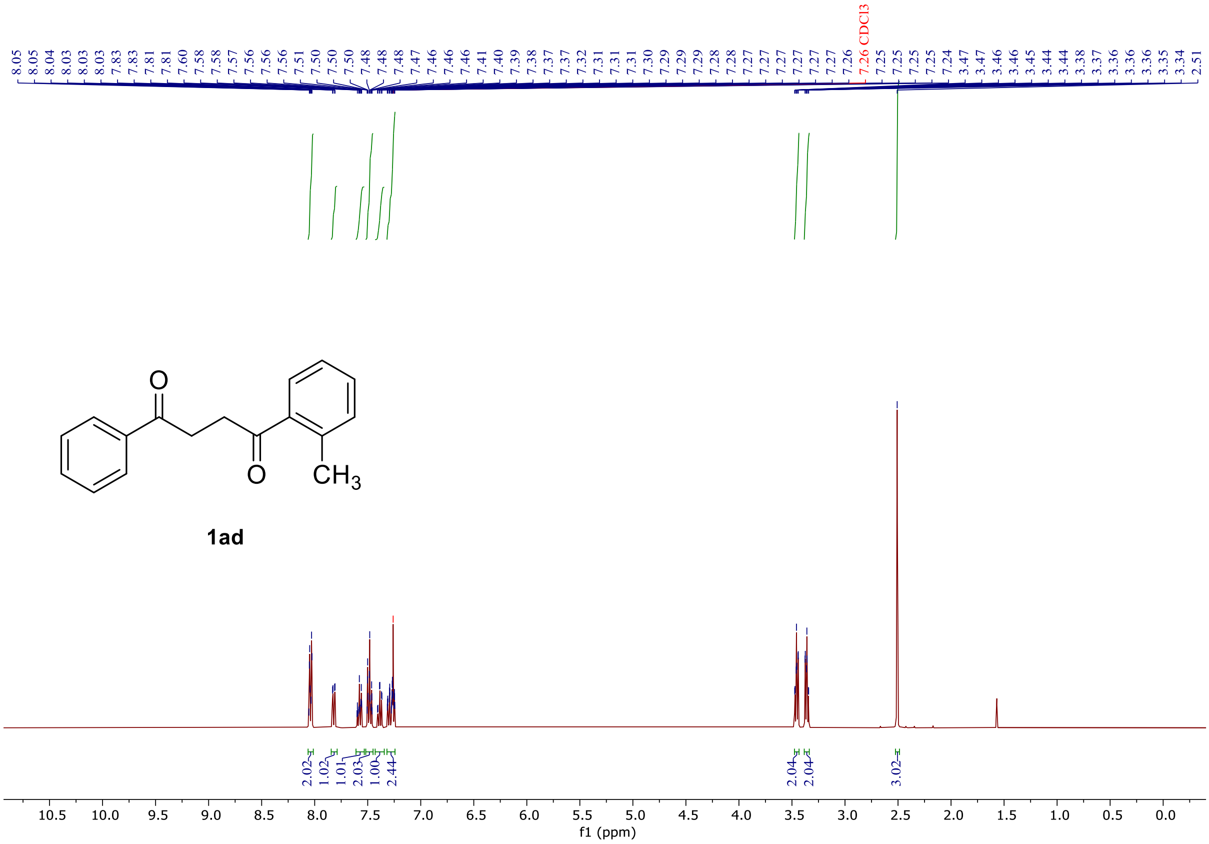

^{13}C{^{1}H} NMR (101 MHz, $CDCl_3$) of **1ad**

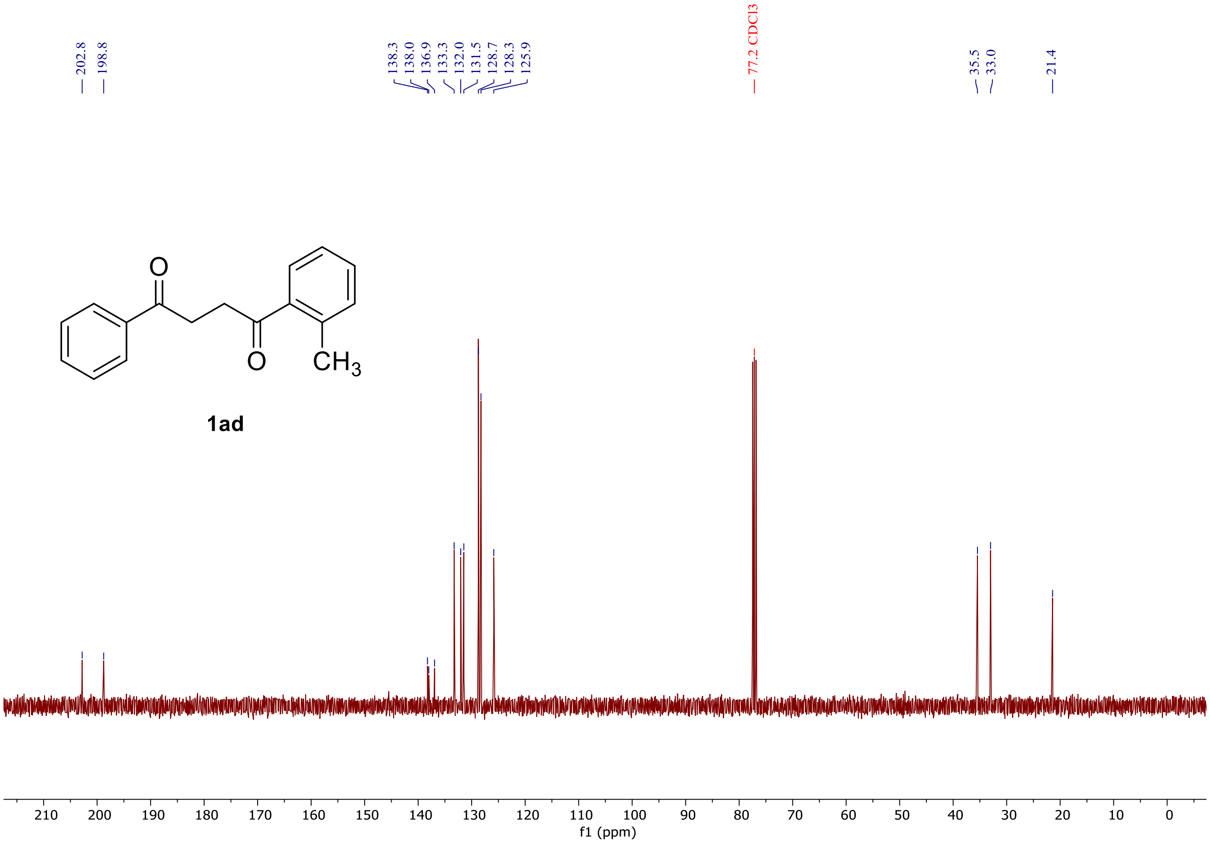

^{1}H NMR (500 MHz, CDCl$_3$) of **1ag**

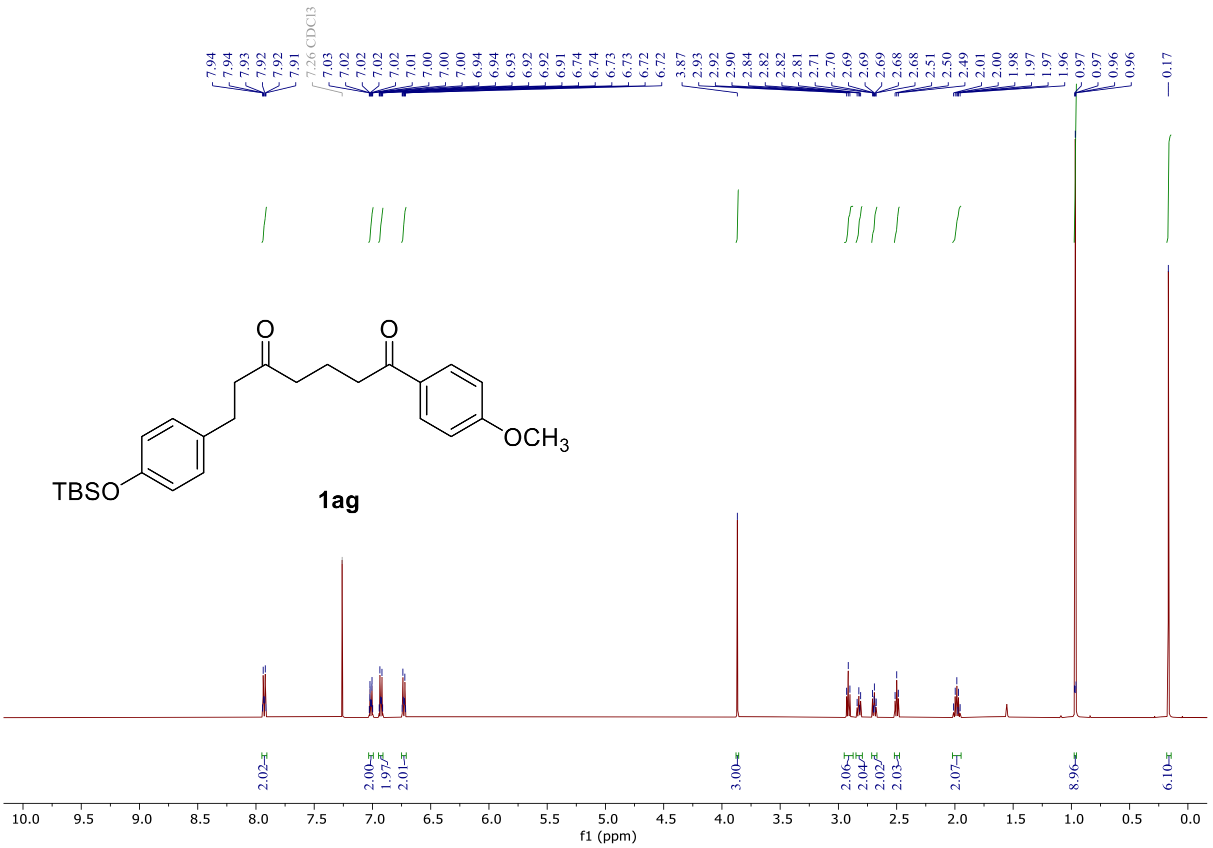

^{13}C{^{1}H} NMR (126 MHz, CDCl$_3$) of **1ag**

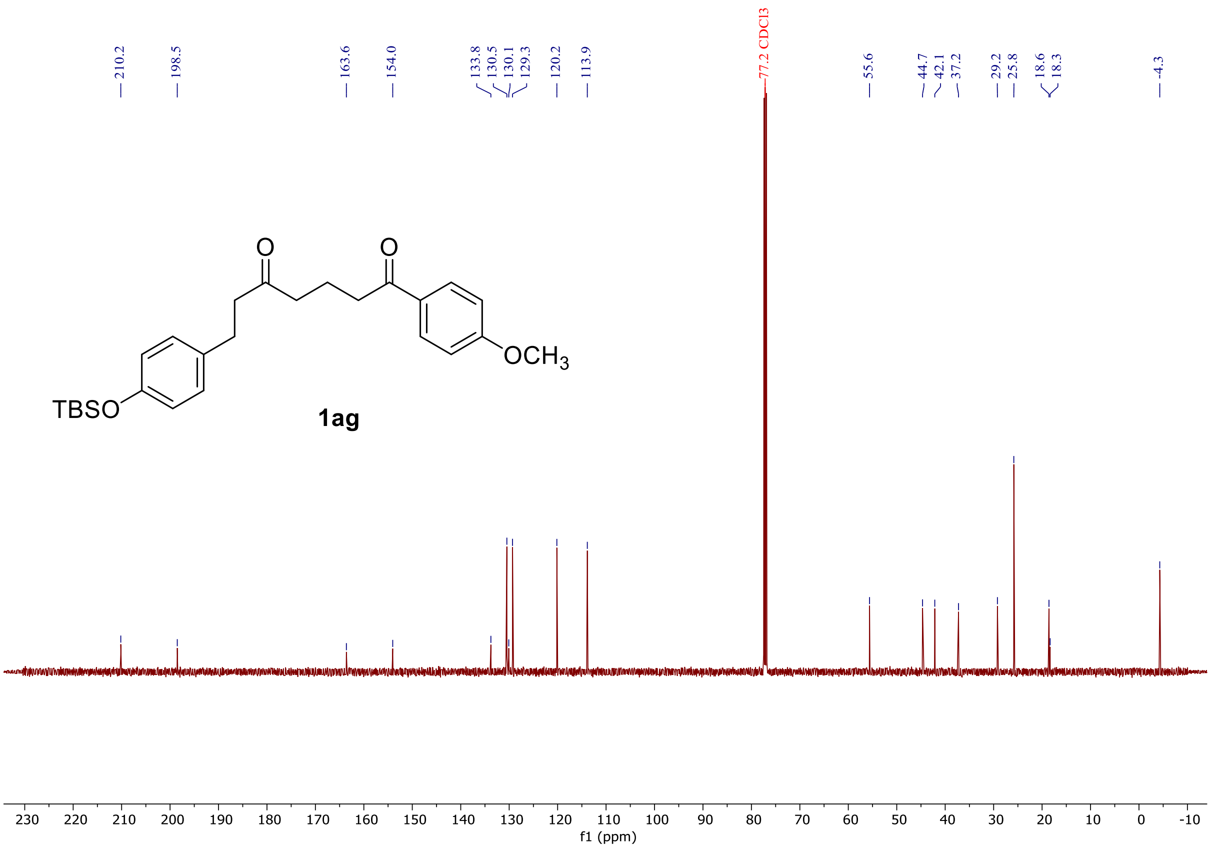

^{1}H NMR (400 MHz, $CDCl_3$) of **1ah**

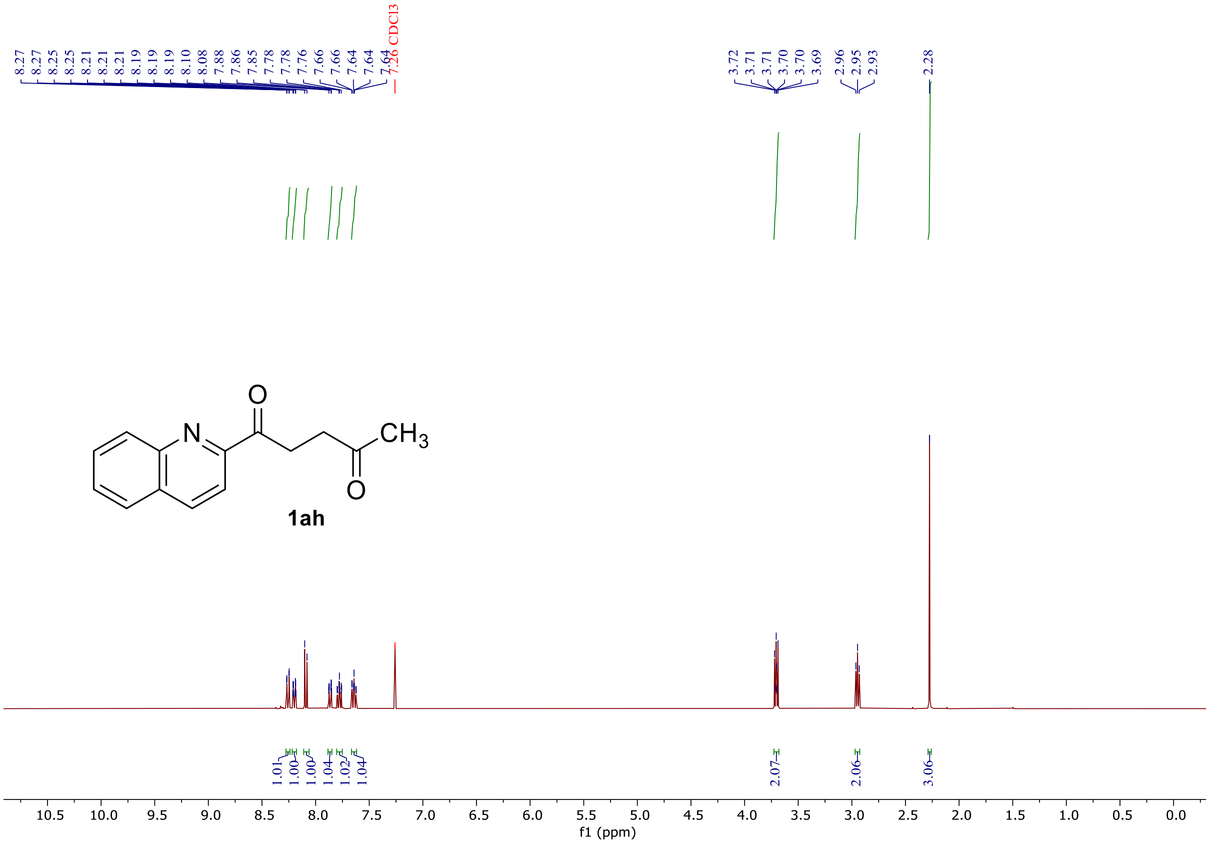

^{13}C{^{1}H} NMR (101 MHz, $CDCl_3$) of **1aa**

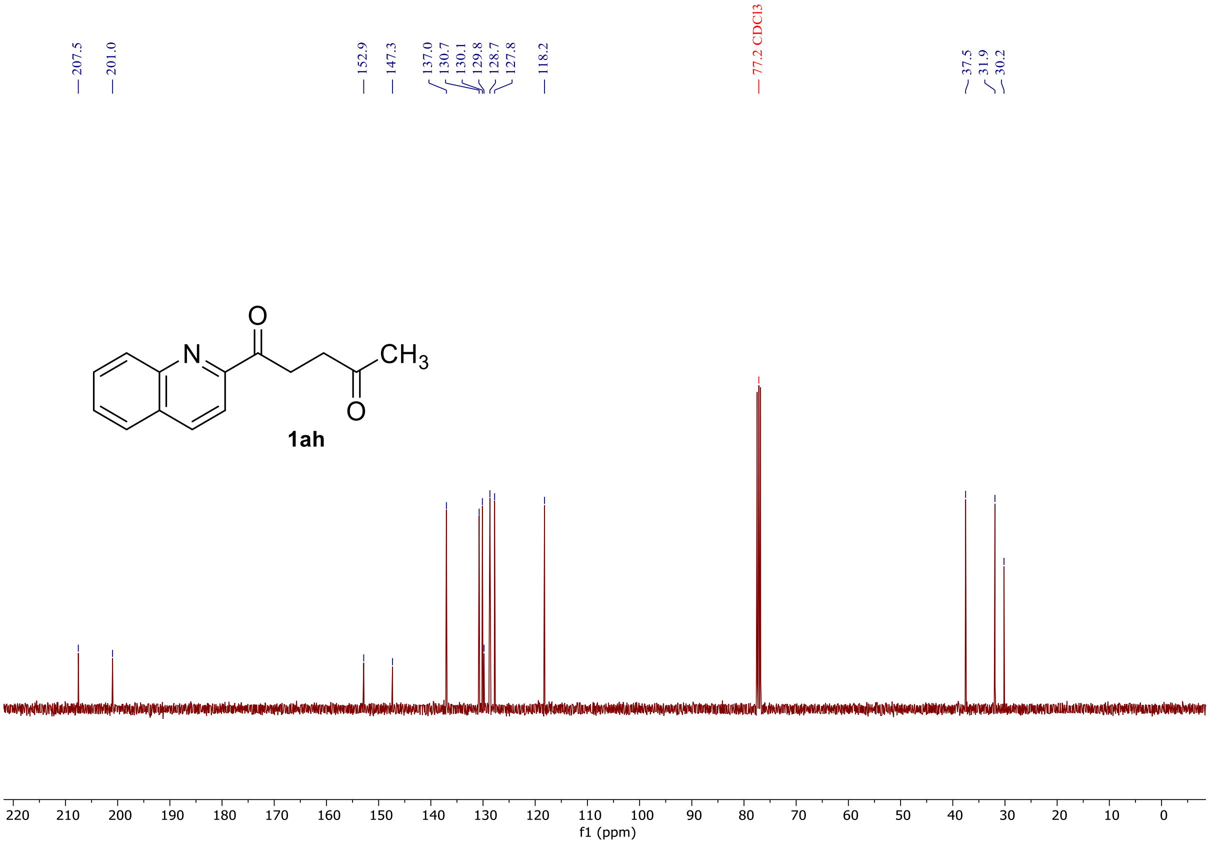

^{1}H NMR (400 MHz, $CDCl_3$) of **1ai**

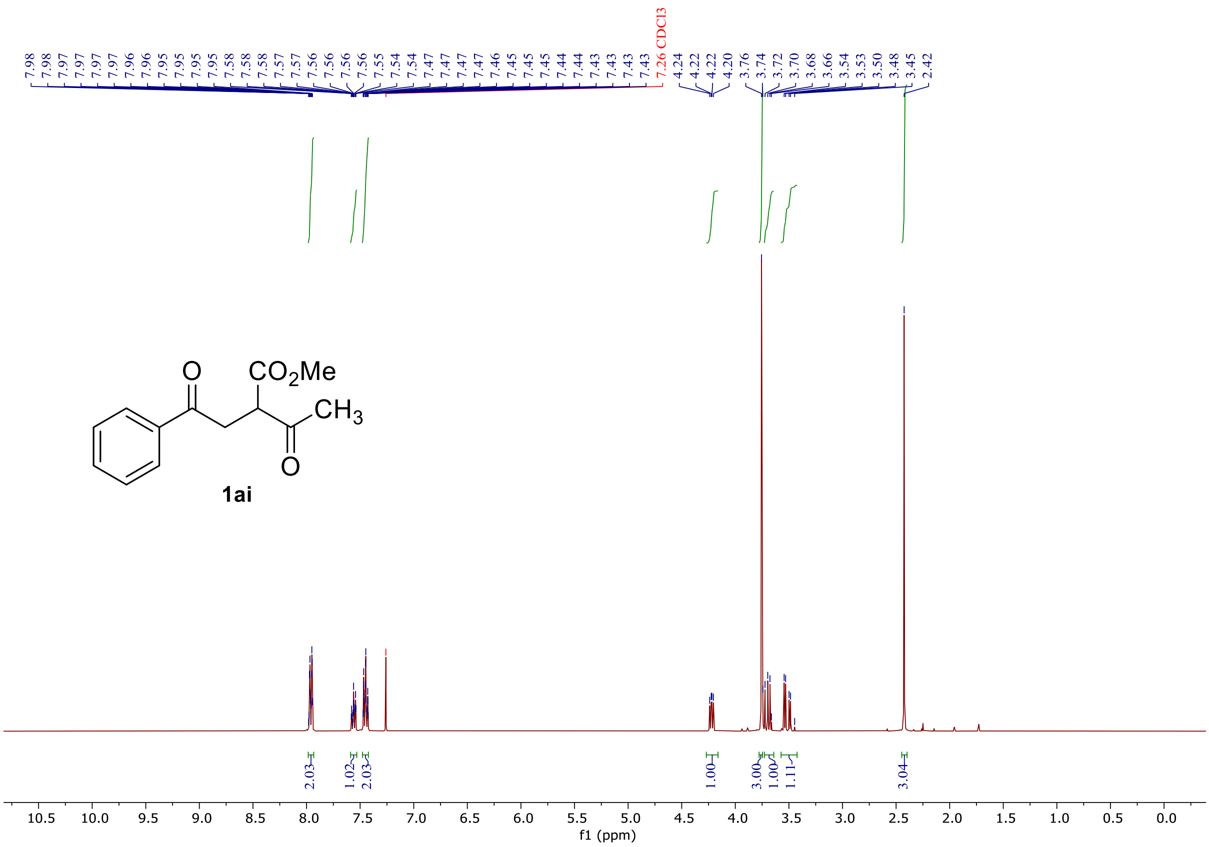

$^{13}C\{^{1}H\}$ NMR (101 MHz, $CDCl_3$) of **1ai**

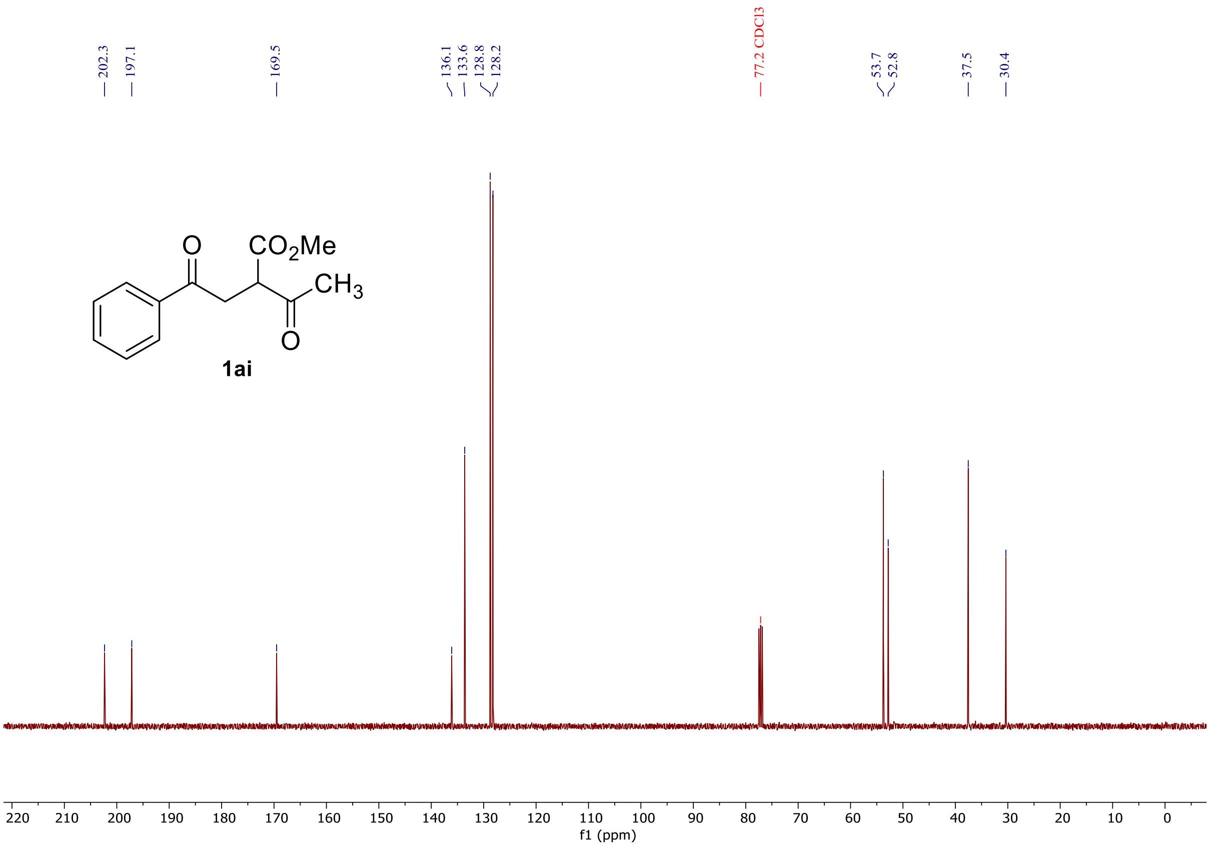

^{1}H NMR (400 MHz, $CDCl_3$) of **1aj**

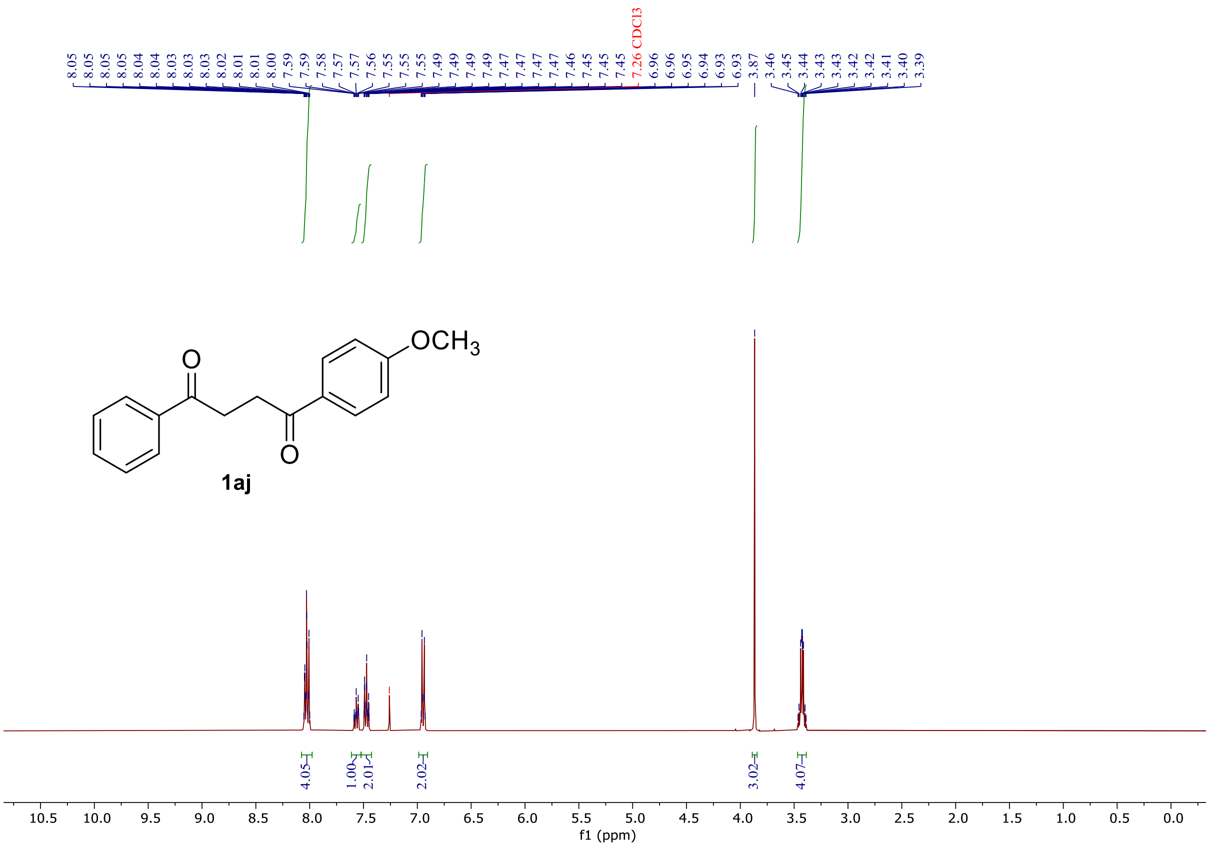

$^{13}C\{^1H\}$ NMR (101 MHz, $CDCl_3$) of **1aj**

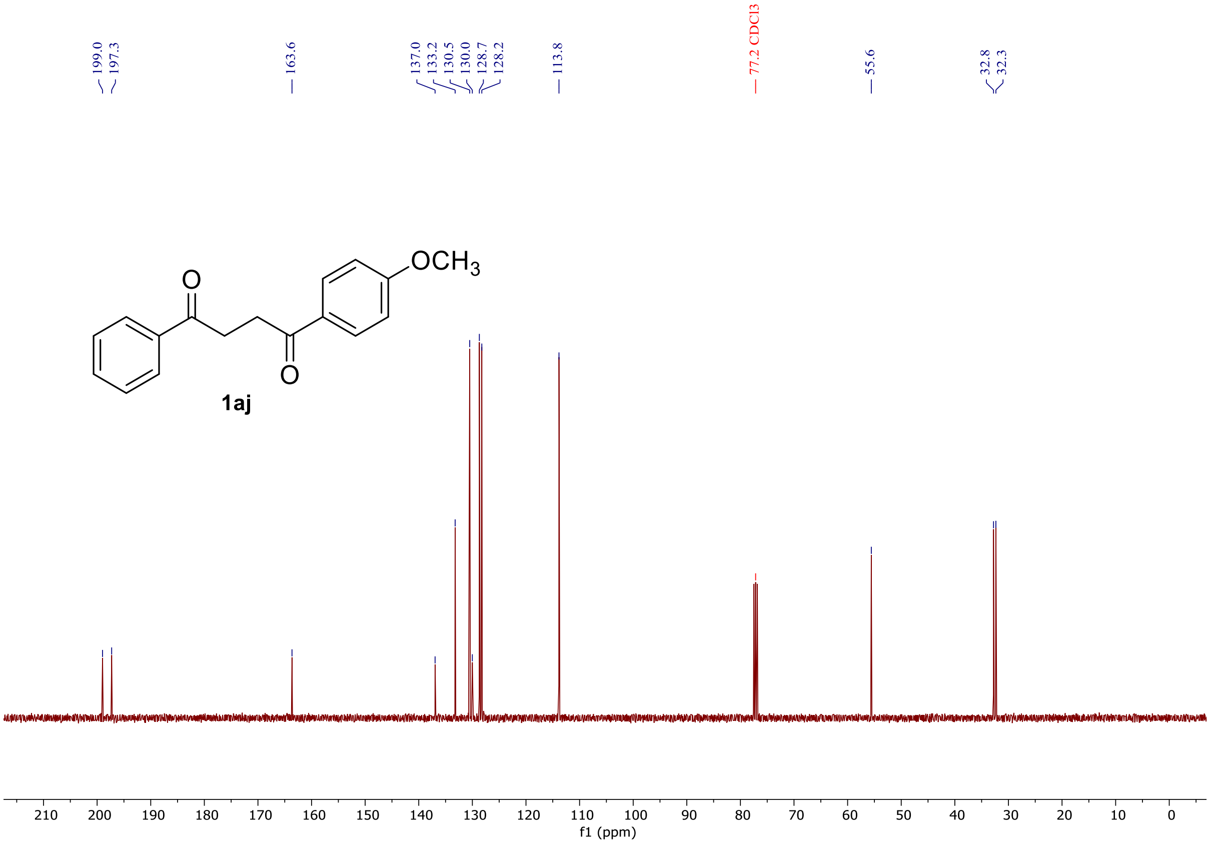

^{1}H NMR (400 MHz, $CDCl_3$) of **1ak**

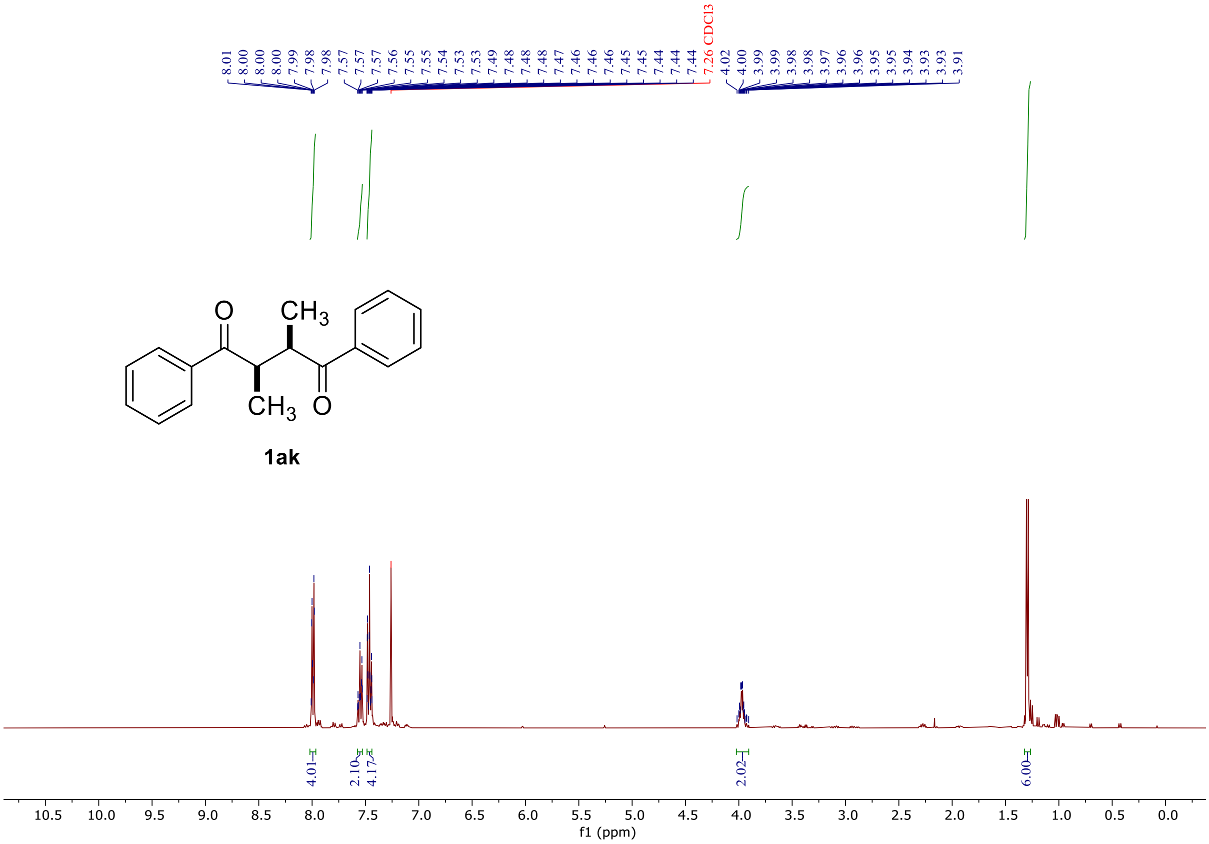

^{13}C{^{1}H} NMR (101 MHz, $CDCl_3$) of **1ak**

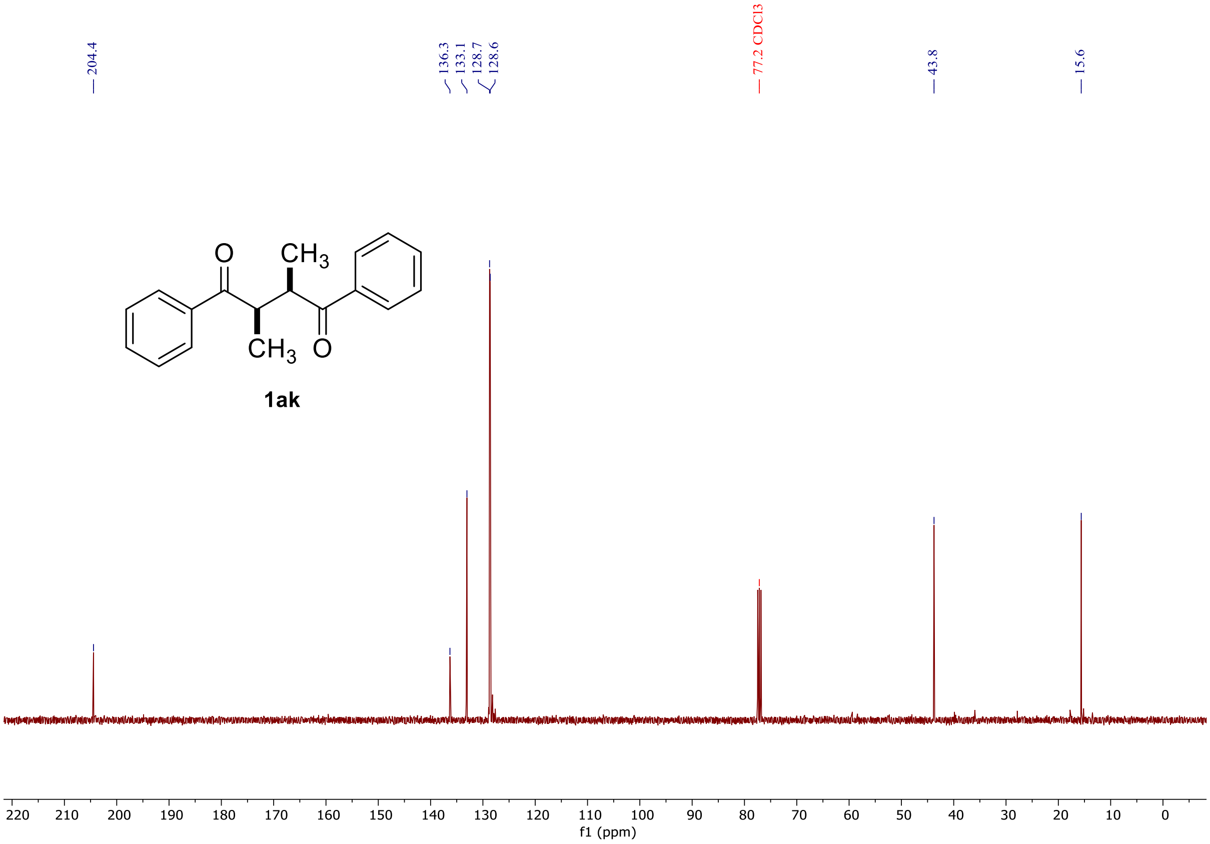

^1H NMR (400 MHz, $CDCl_3$) of **1al**

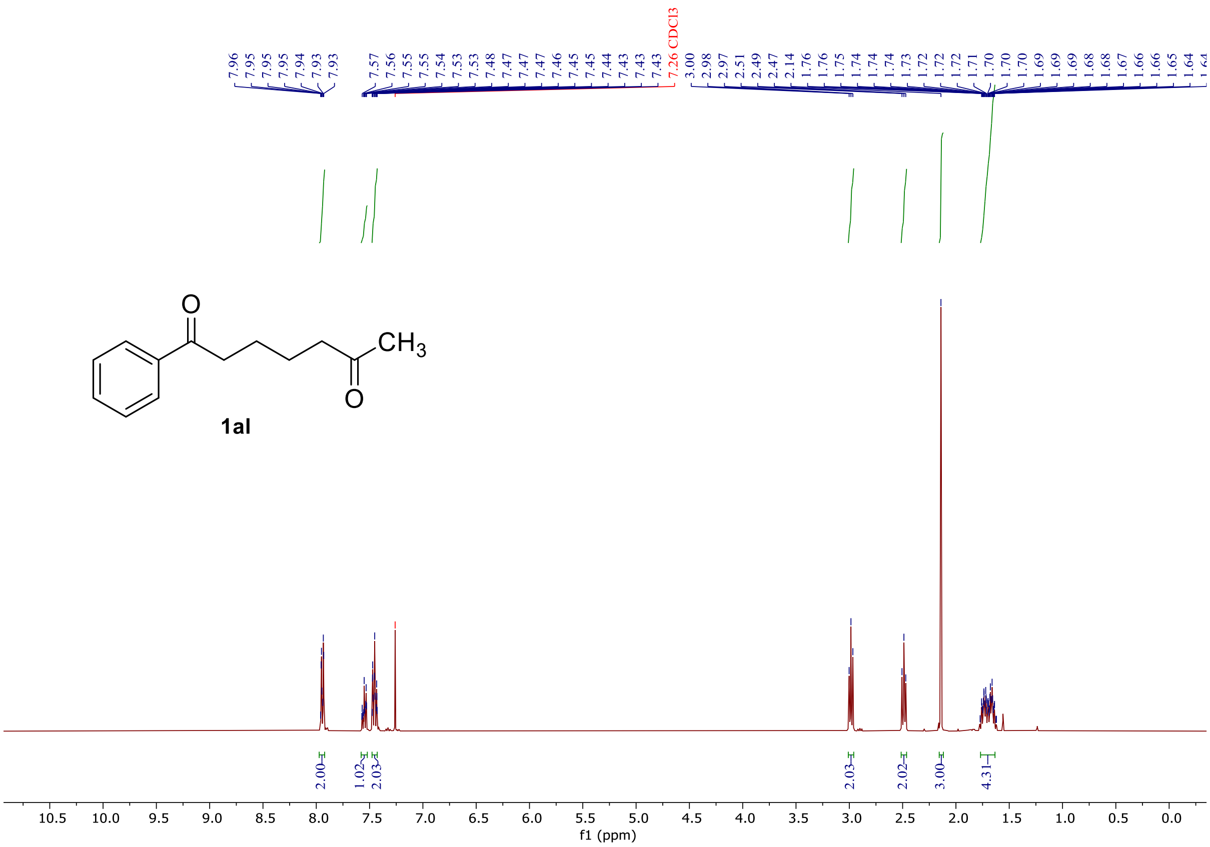

$^{13}C\{^1H\}$ NMR (101 MHz, $CDCl_3$) of **1al**

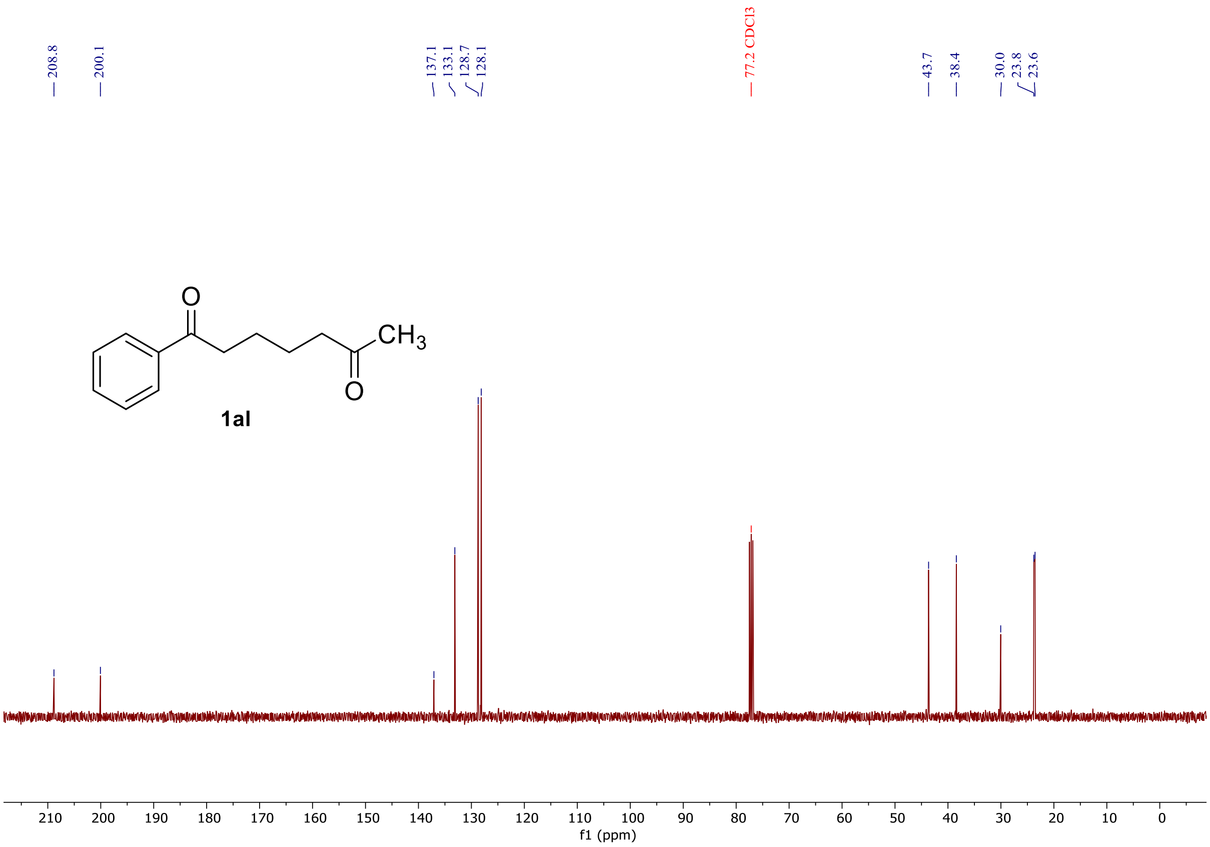

^{1}H NMR (400 MHz, $CDCl_3$) of **1am**

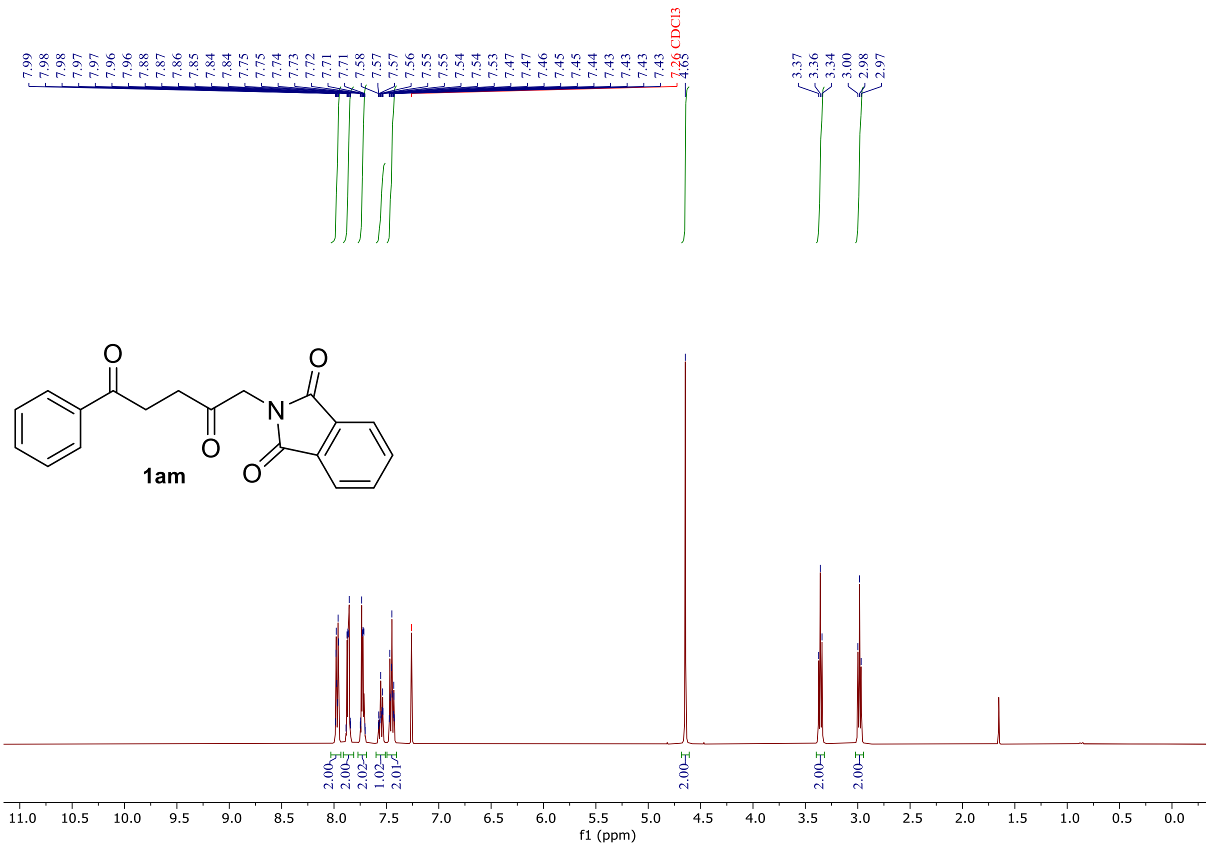

$^{13}C\{^{1}H\}$ NMR (101 MHz, $CDCl_3$) of **1am**

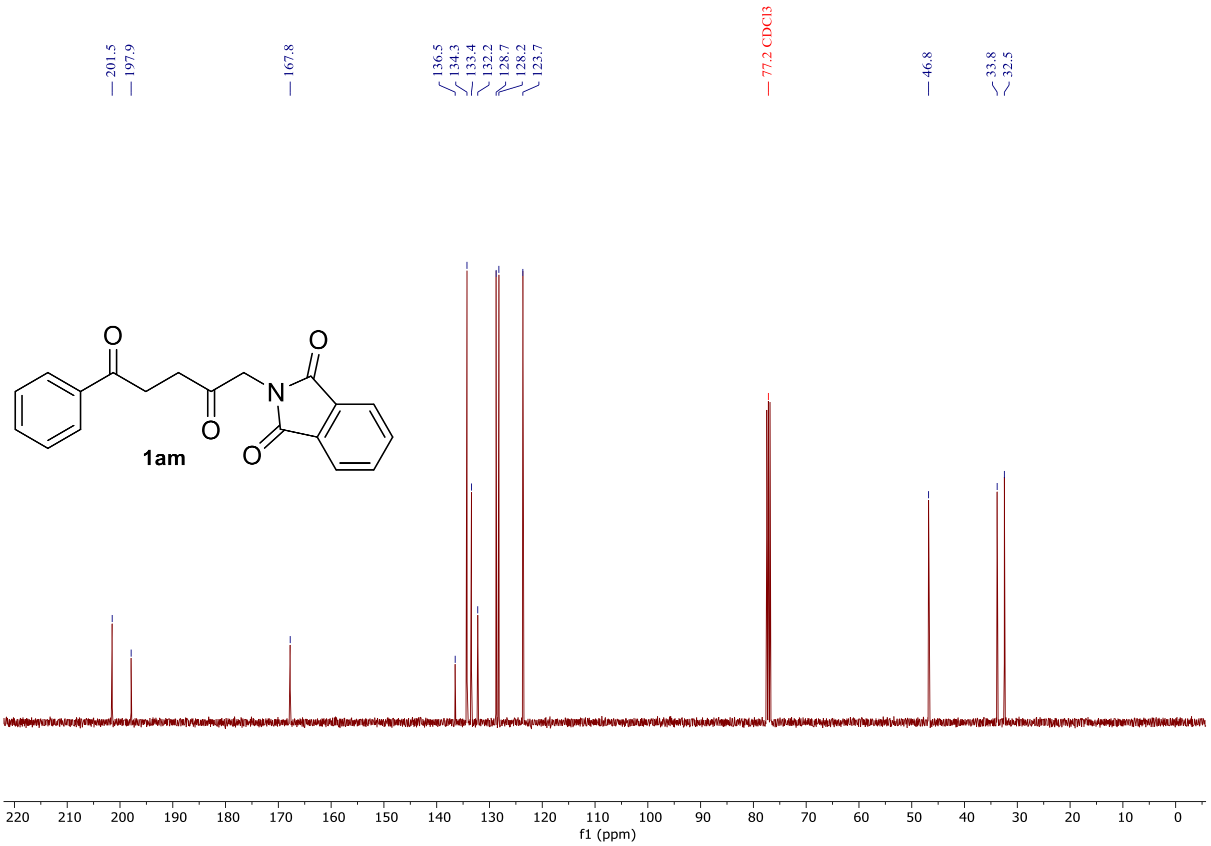

^{1}H NMR (400 MHz, $CDCl_3$) of **1an**

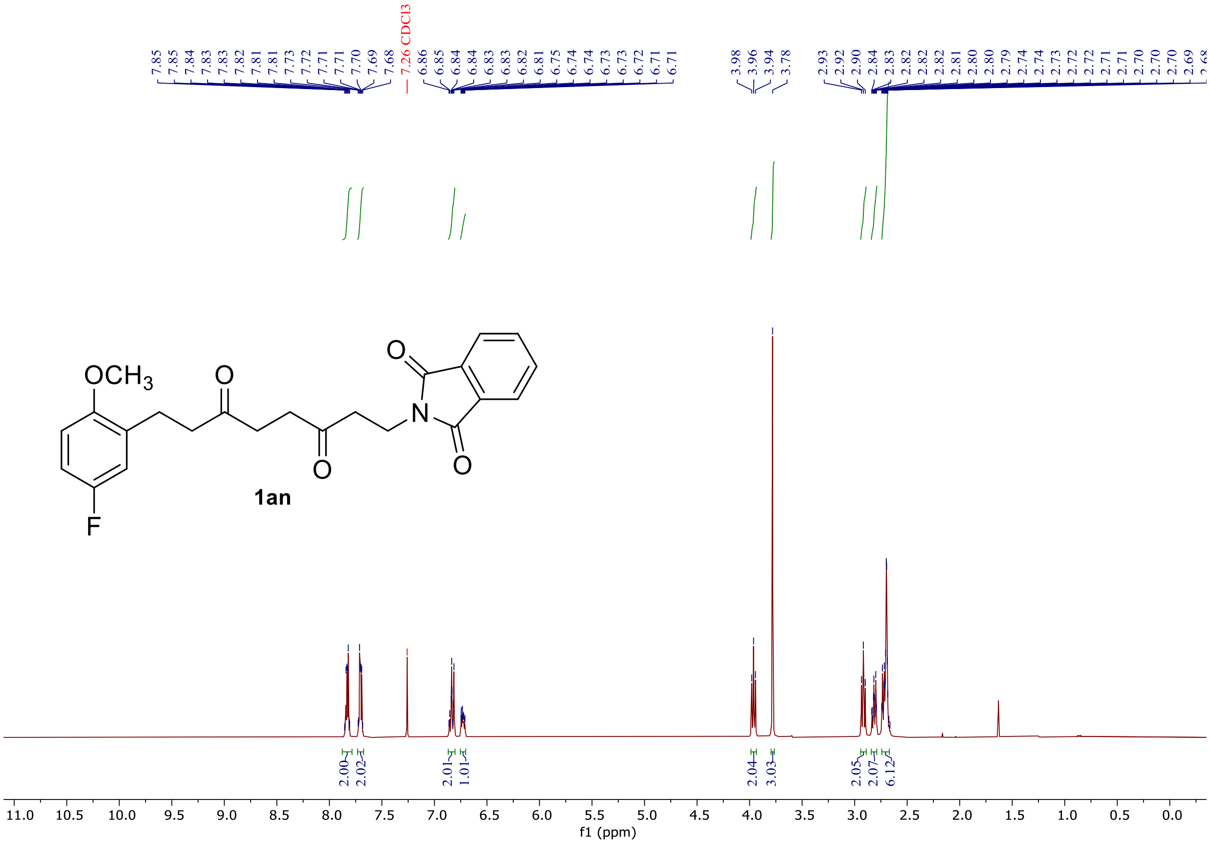

$^{13}C\{^{1}H\}$ NMR (101 MHz, $CDCl_3$) of **1an**

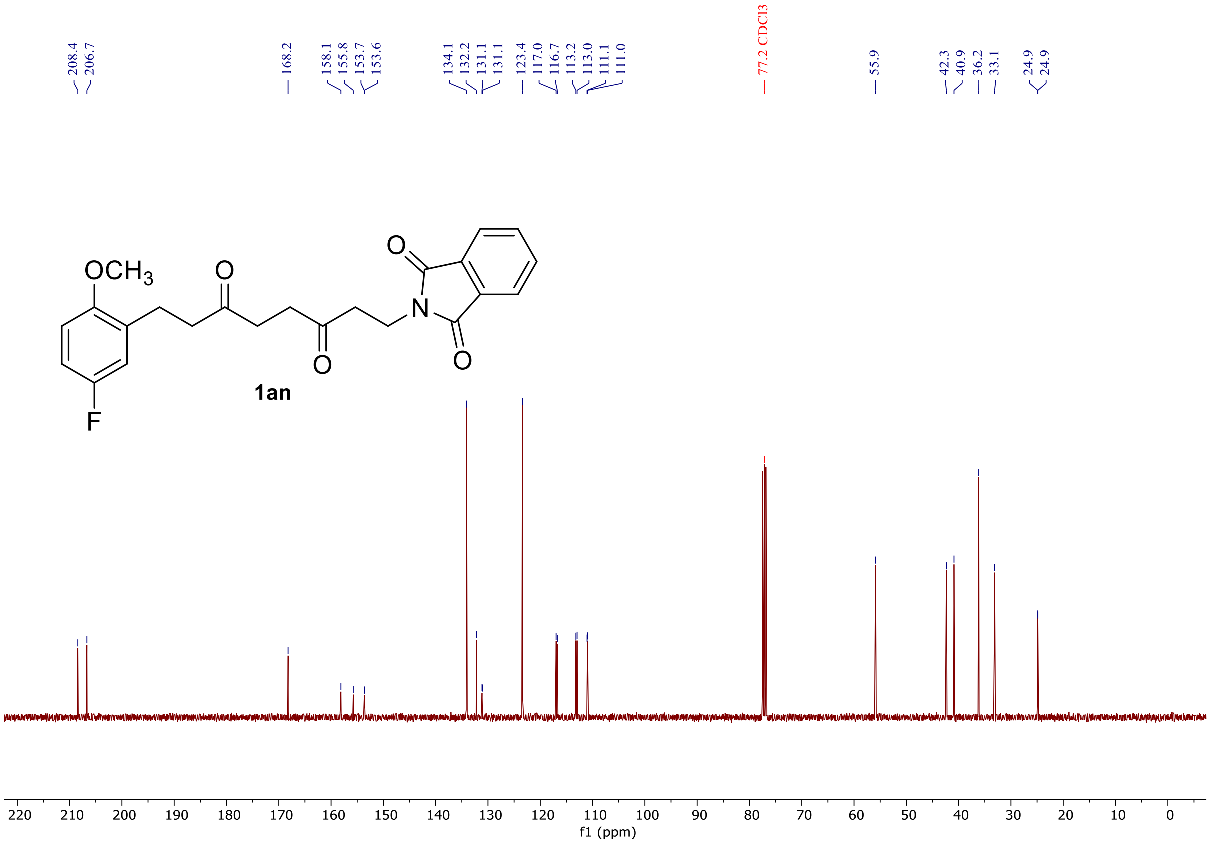

$^{19}F\{^{1}H\}$ NMR (377 MHz, $CDCl_3$) of **1an**

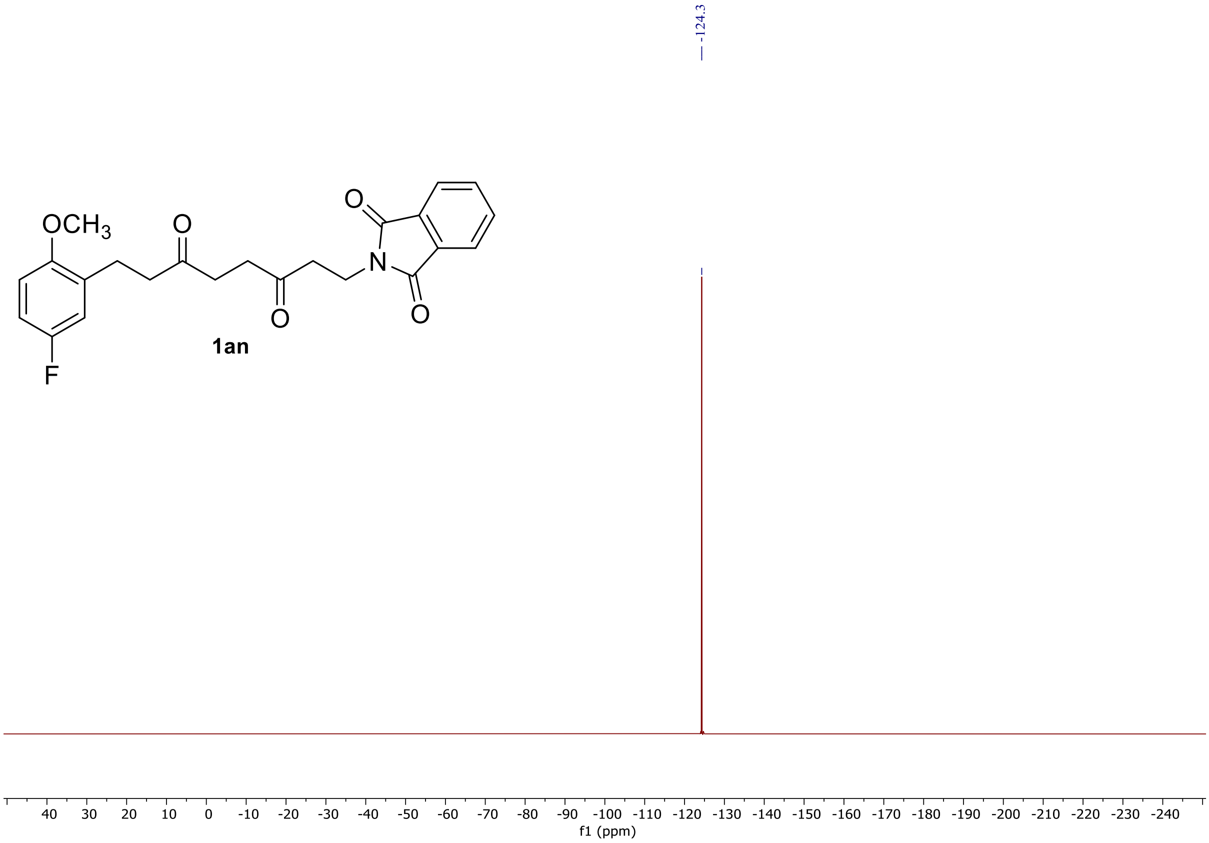

^{1}H NMR (500 MHz, $CDCl_3$) of **2a**

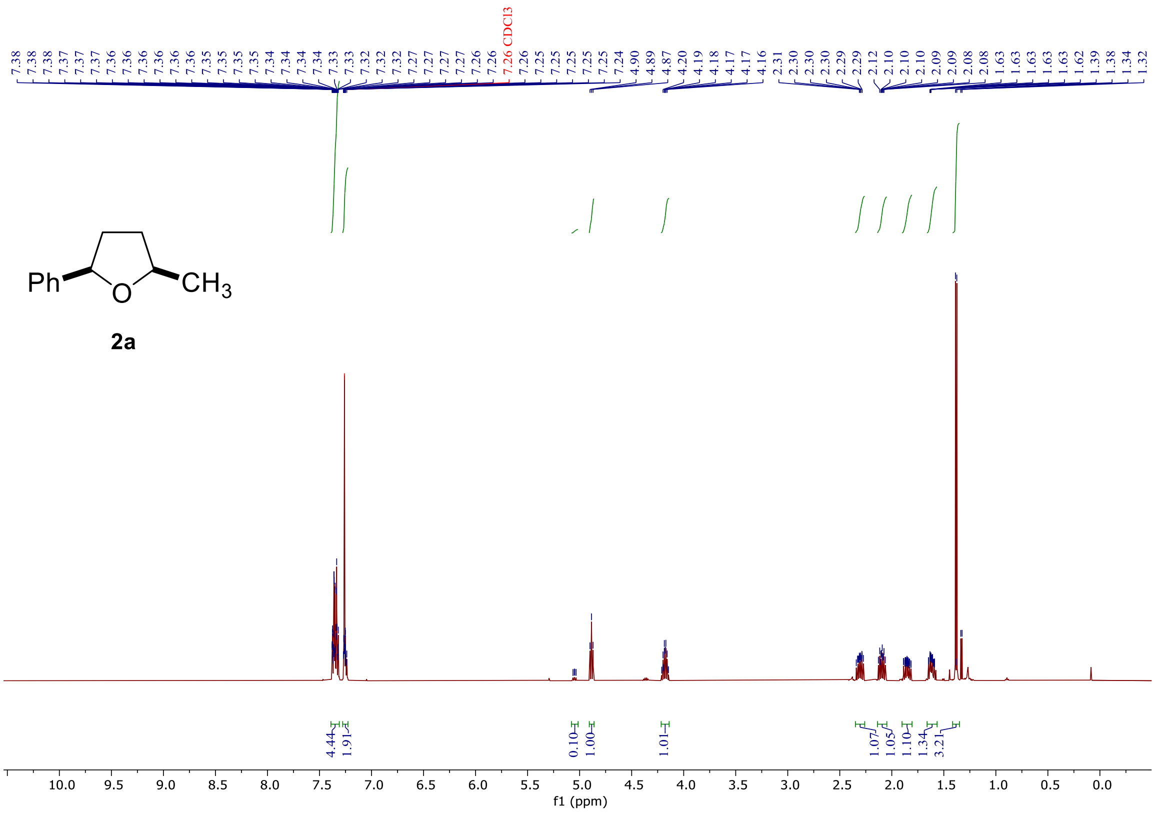

$^{13}C\{^{1}H\}$ NMR (126 MHz, $CDCl_3$) of **2a**

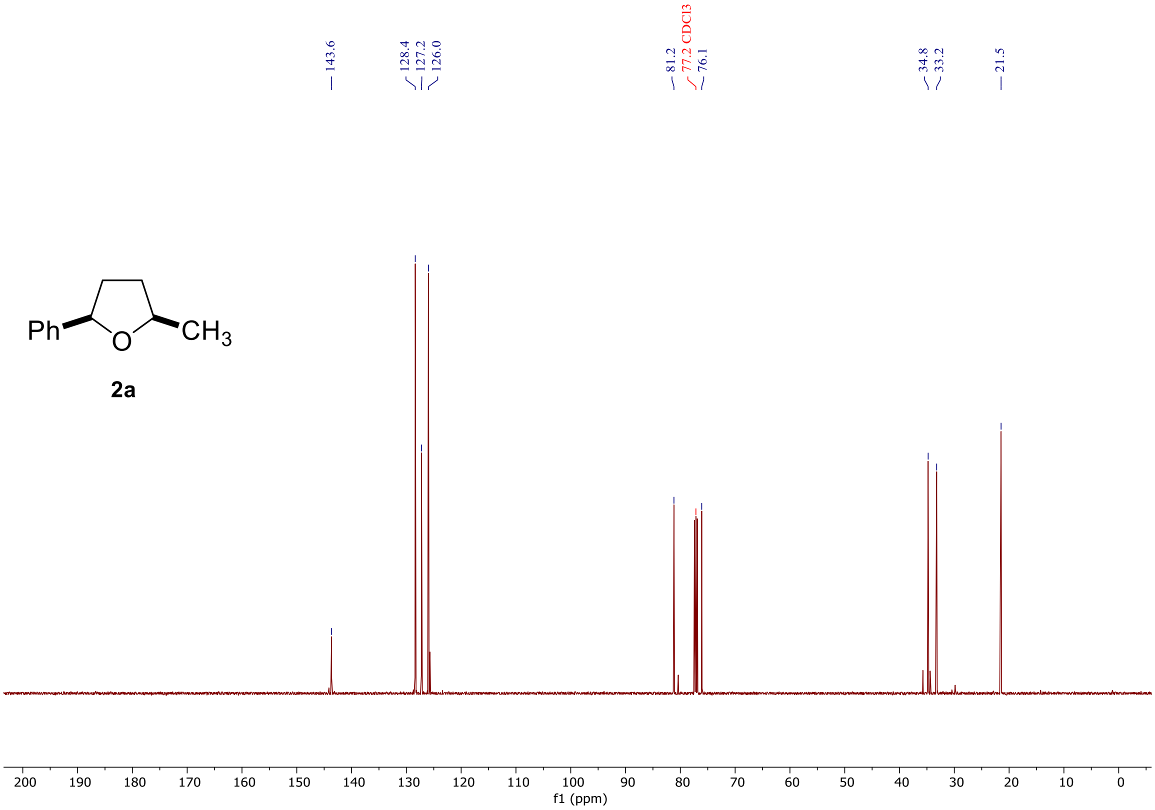

^{1}H NMR (500 MHz, $CDCl_3$) of **2b**

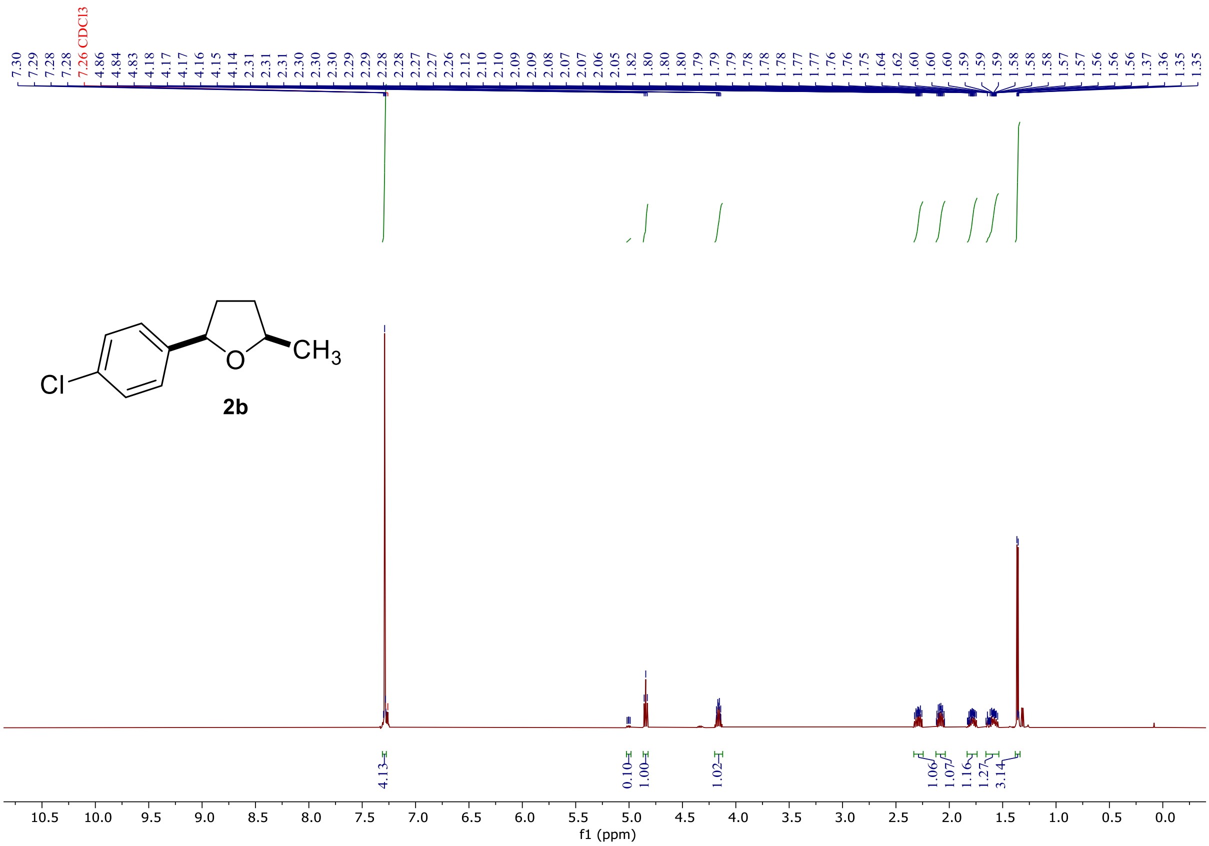

$^{13}C\{^{1}H\}$ NMR (126 MHz, $CDCl_3$) of **2b**

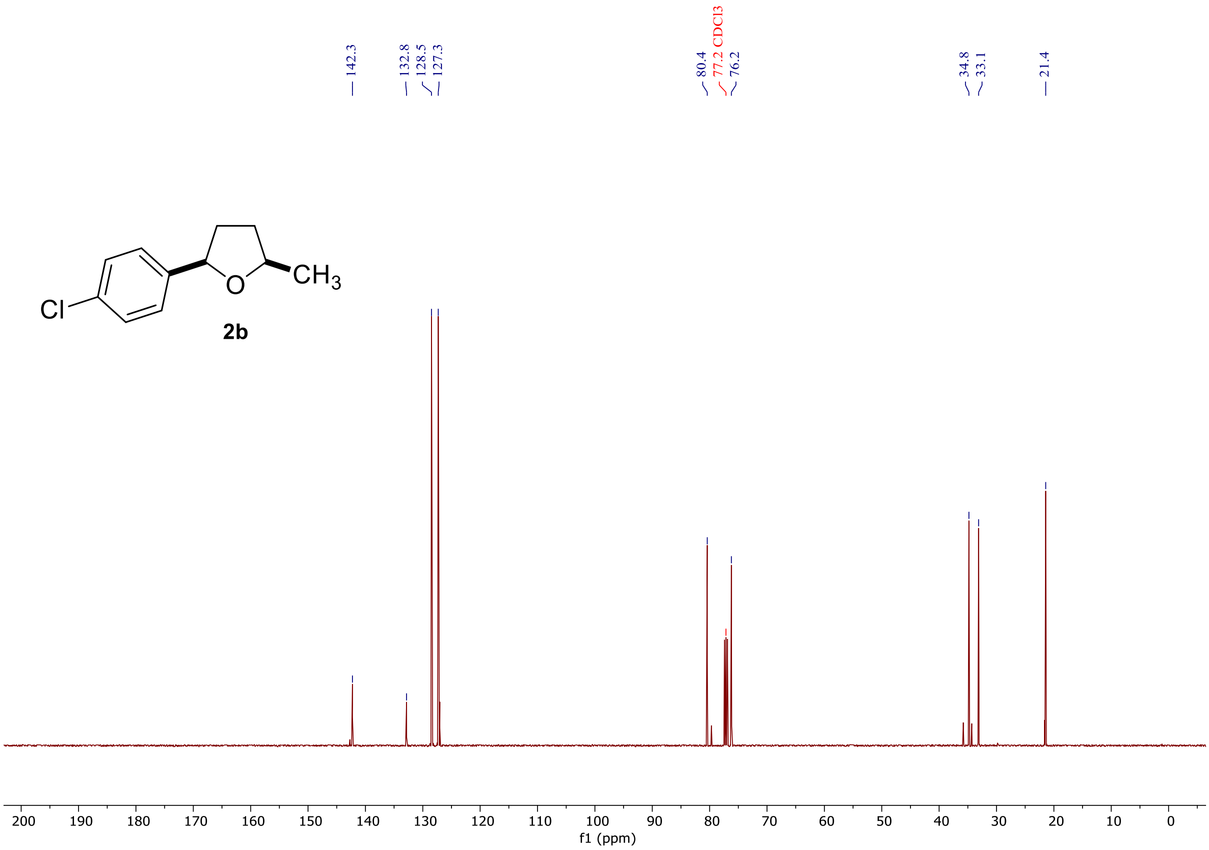

^{1}H NMR (500 MHz, $CDCl_3$) of **2c**

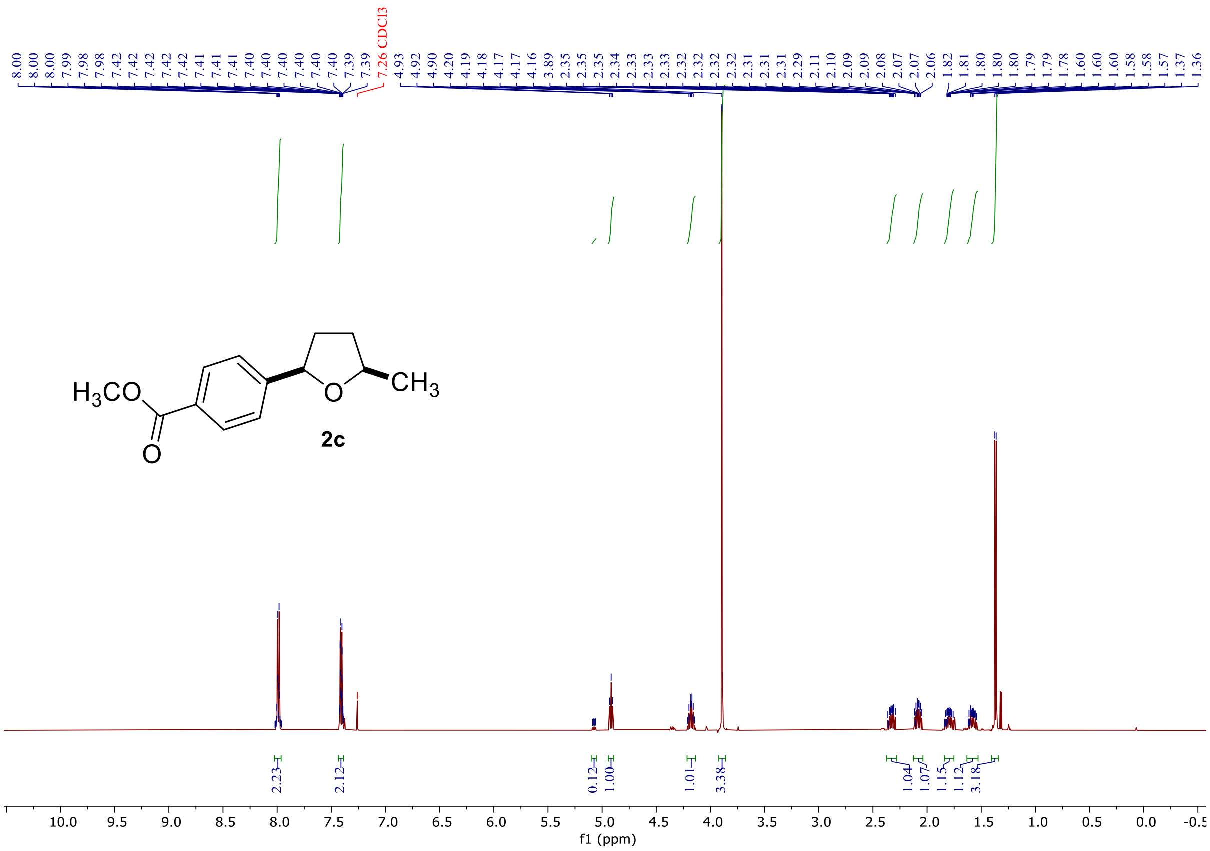

^{13}C{^{1}H} NMR (126 MHz, $CDCl_3$) of **2c**

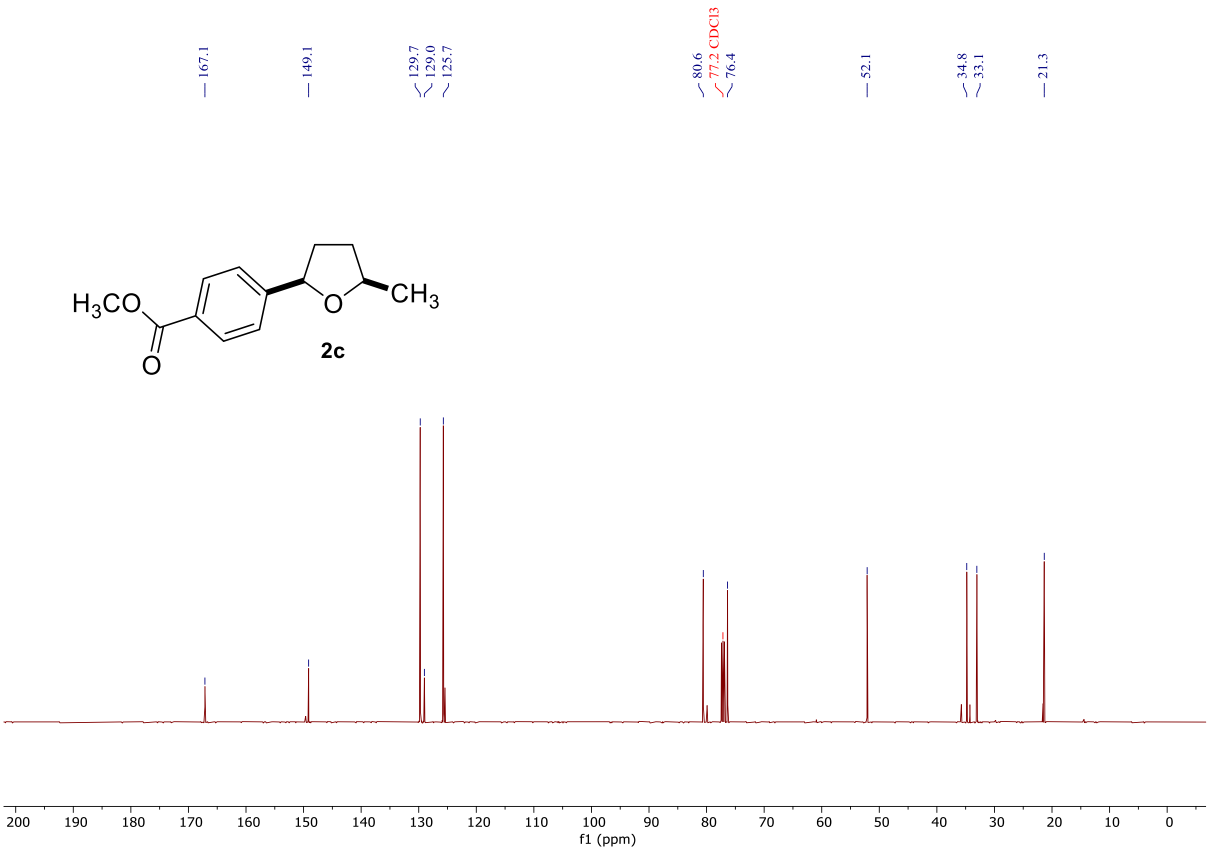

^{1}H NMR (500 MHz, $CDCl_3$) of **2d**

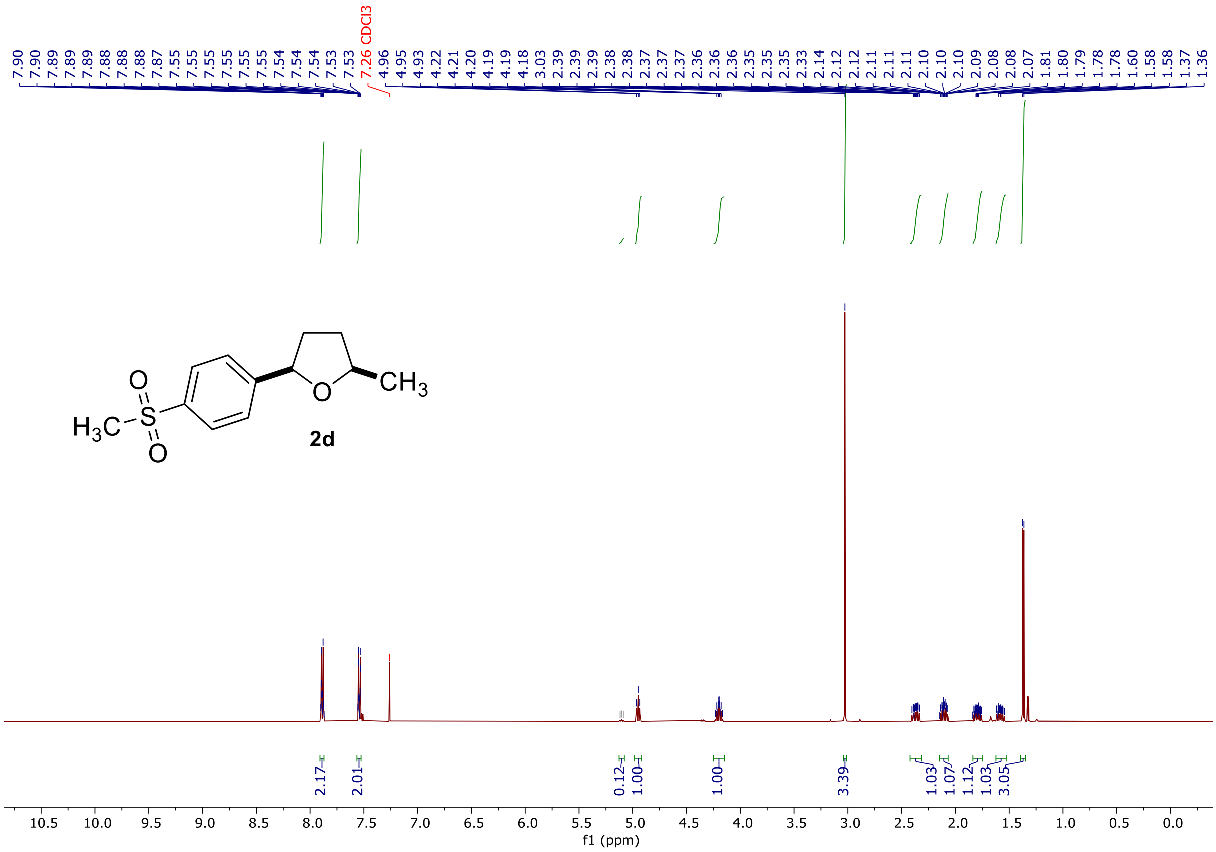

^{13}C{^{1}H} NMR (126 MHz, $CDCl_3$) of **2d**

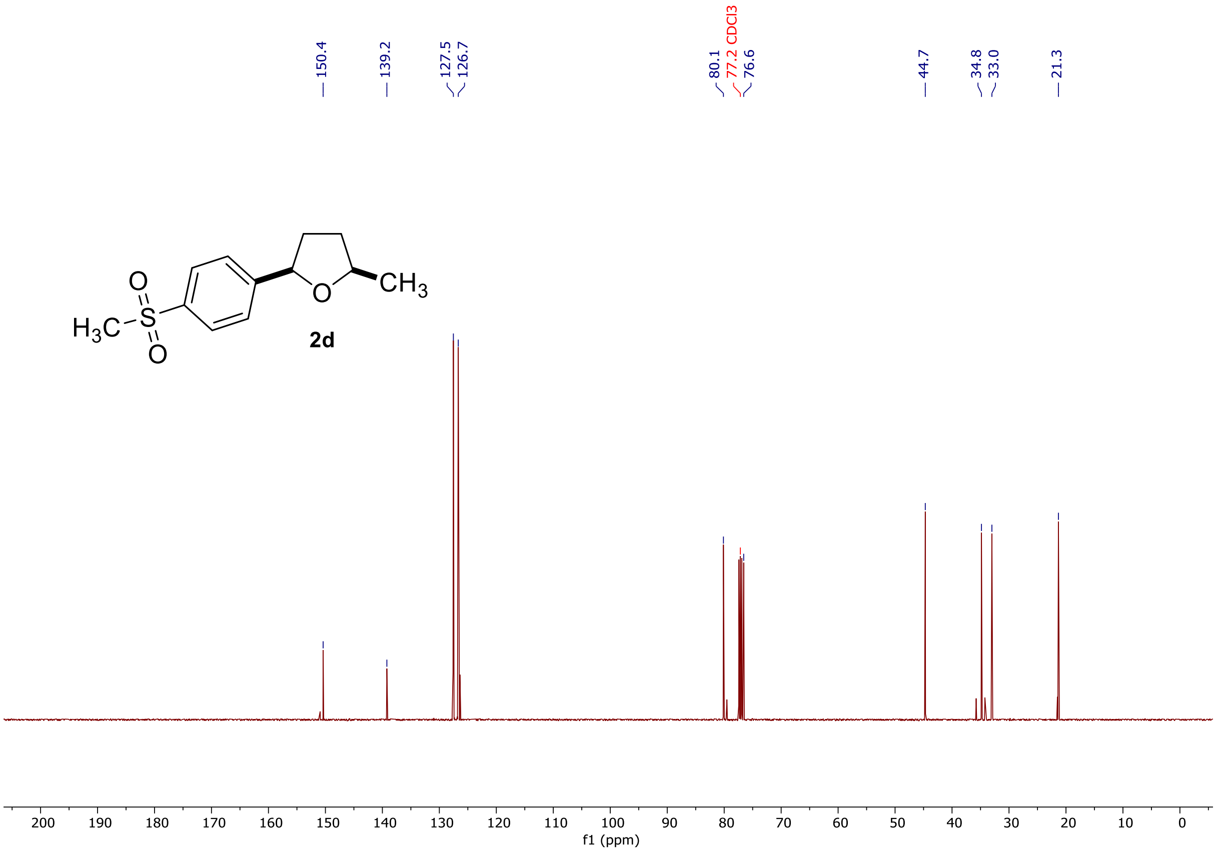

^{1}H NMR (500 MHz, $CDCl_3$) of **2e**

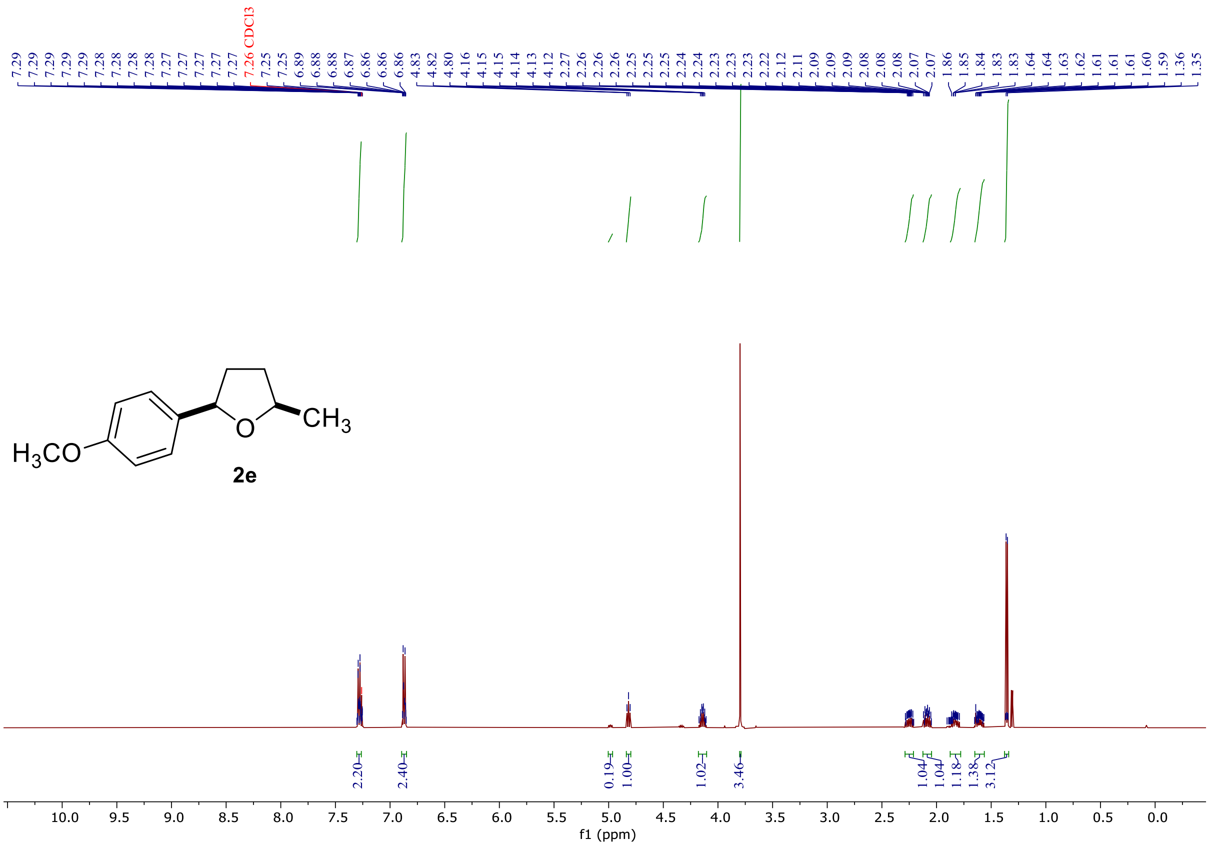

$^{13}C\{^1H\}$ NMR (126 MHz, $CDCl_3$) of **2e**

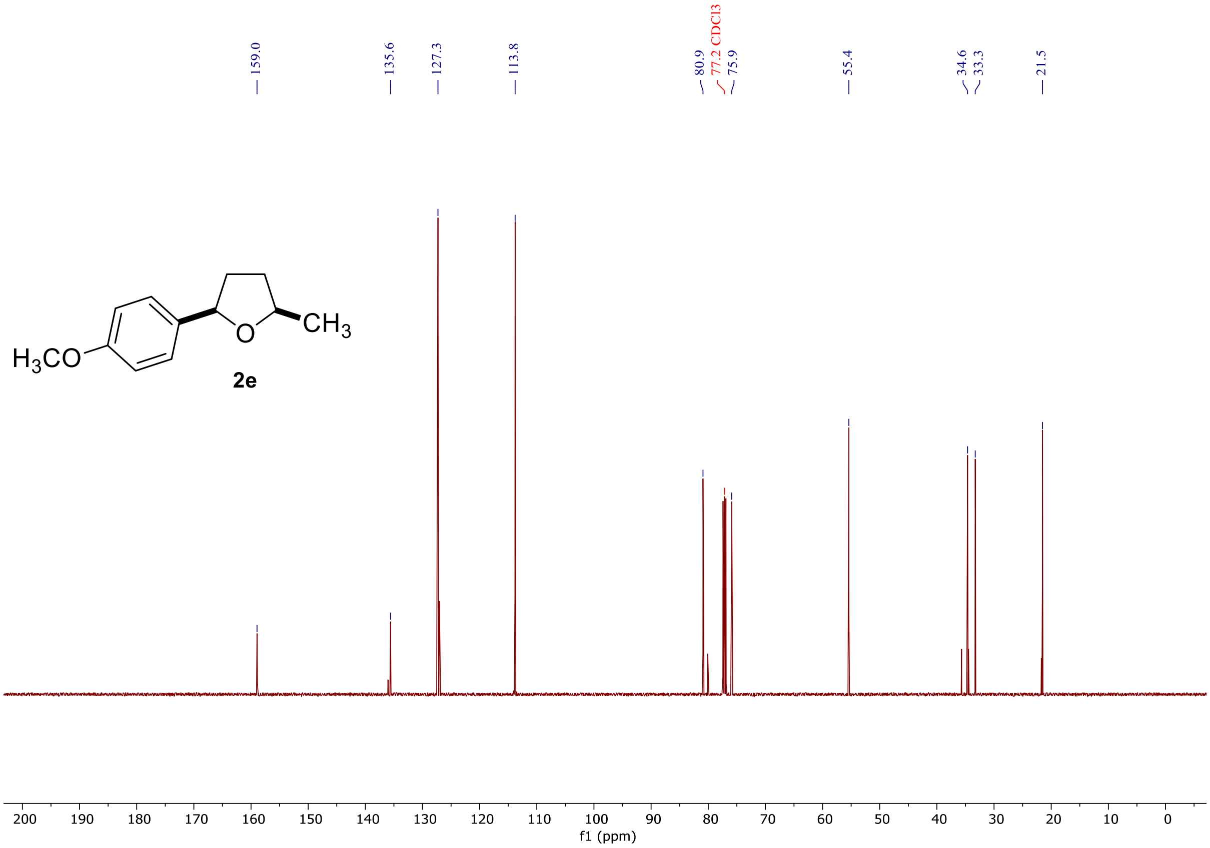

^{1}H NMR (500 MHz, $CDCl_3$) of **2f**

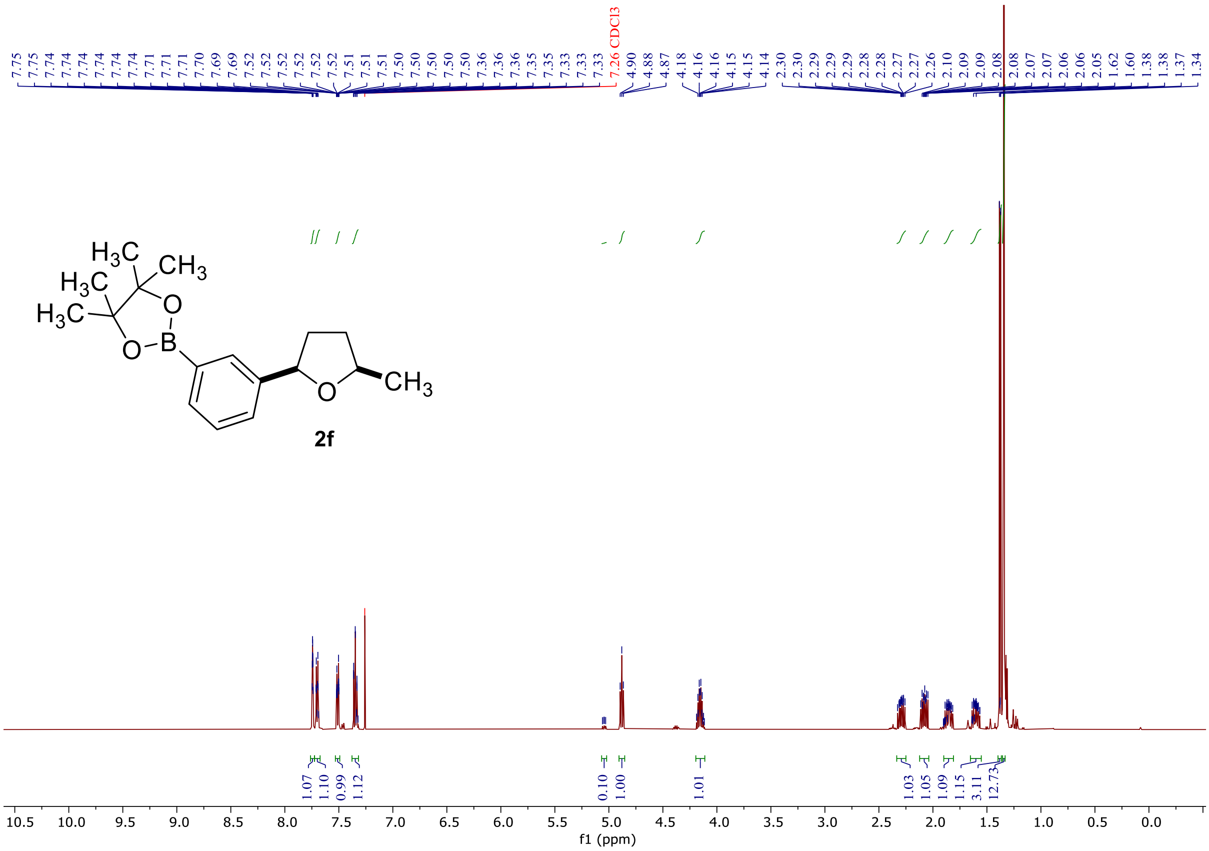

^{13}C{^{1}H} NMR (126 MHz, $CDCl_3$) of **2f**

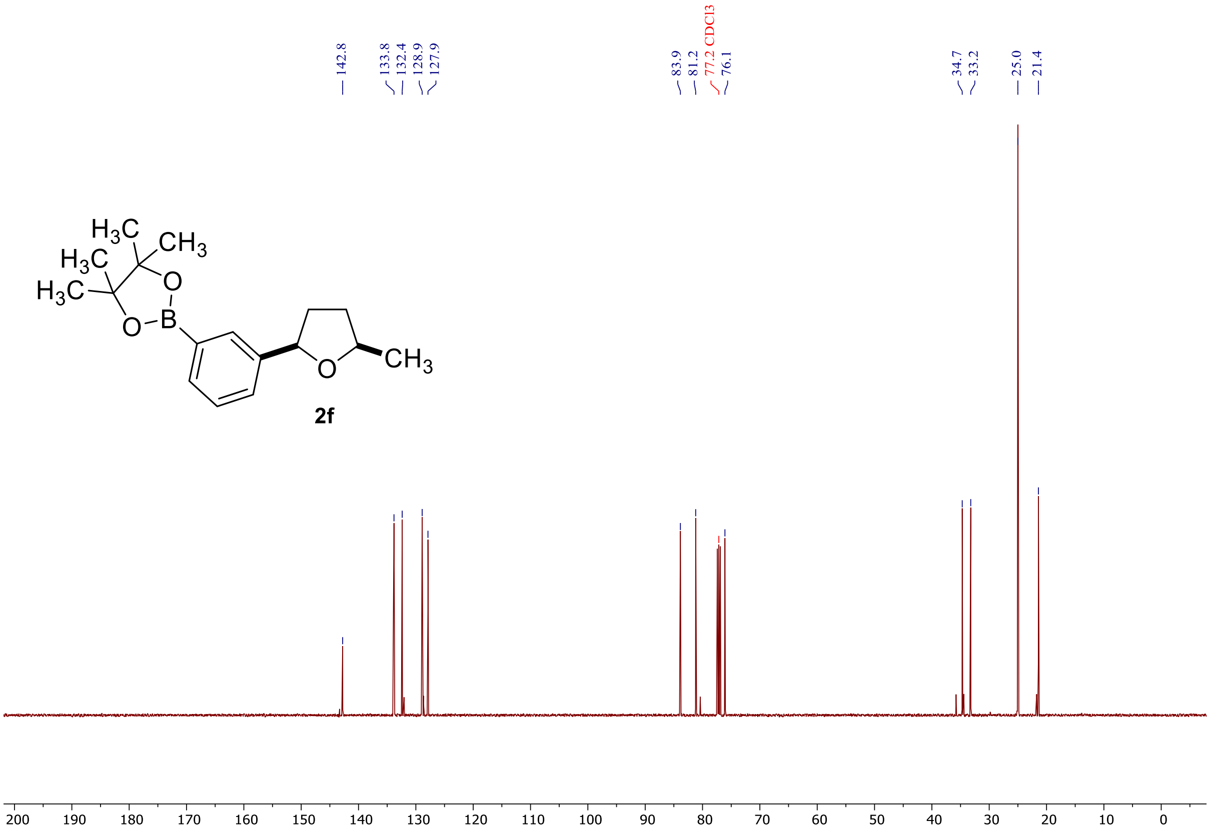

^{11}B NMR (160 MHz, $CDCl_3$) of **2f**

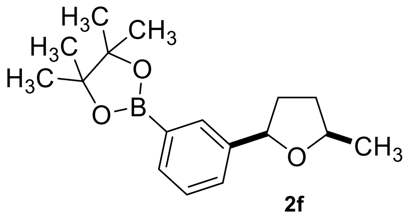

2f

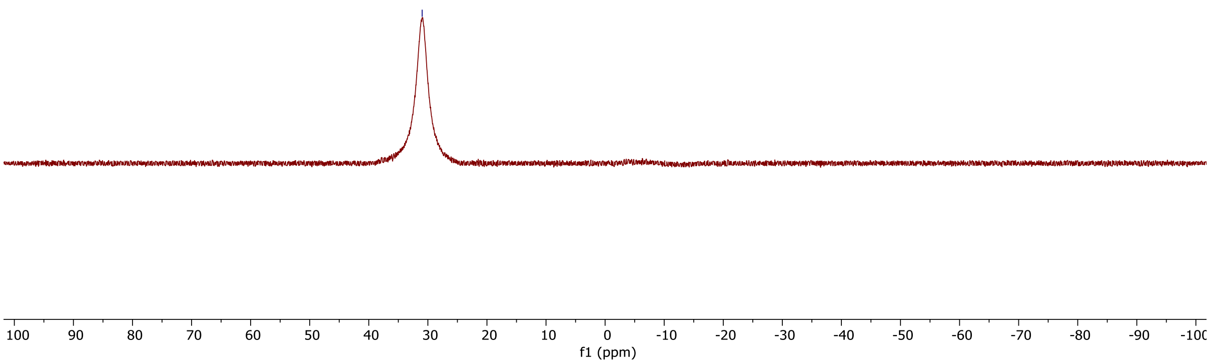

^{1}H NMR (500 MHz, $CDCl_3$) of **2g**

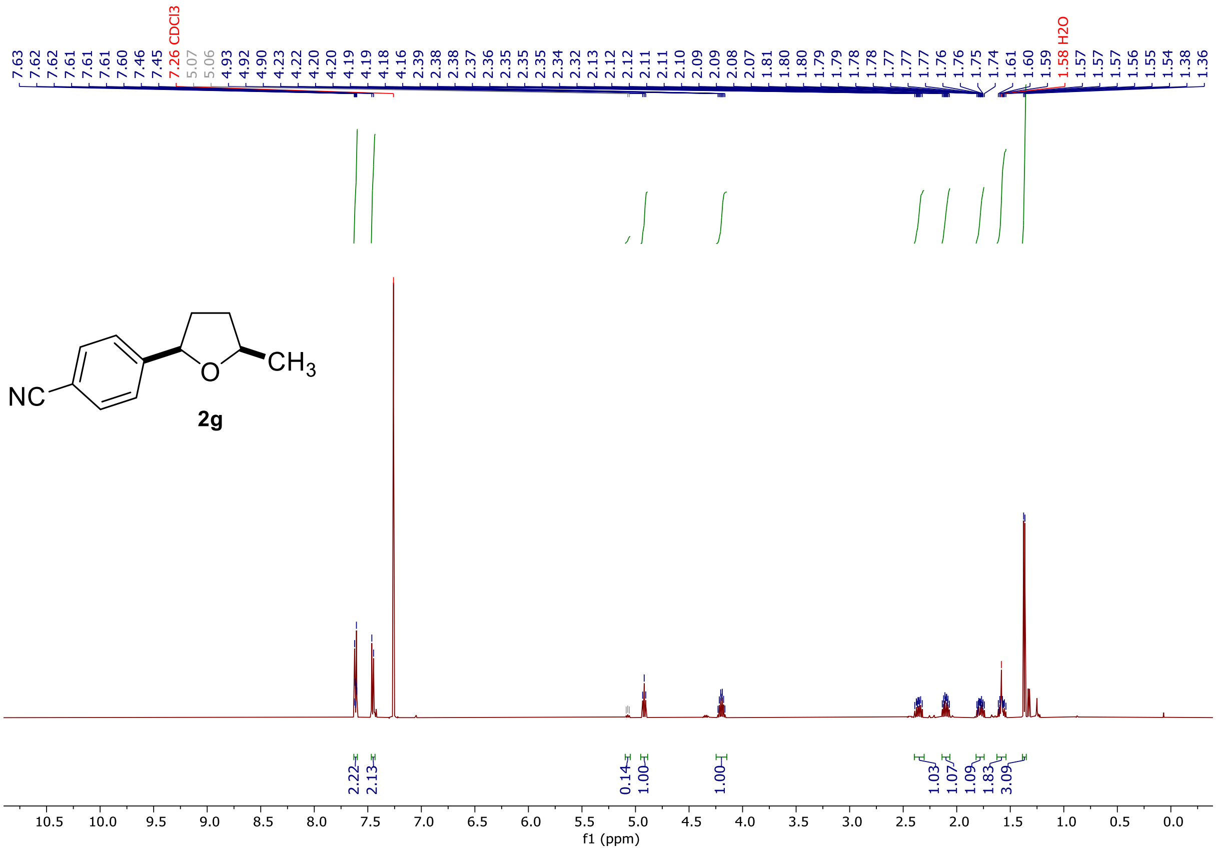

^{13}C{^{1}H} NMR (126 MHz, $CDCl_3$) of **2g**

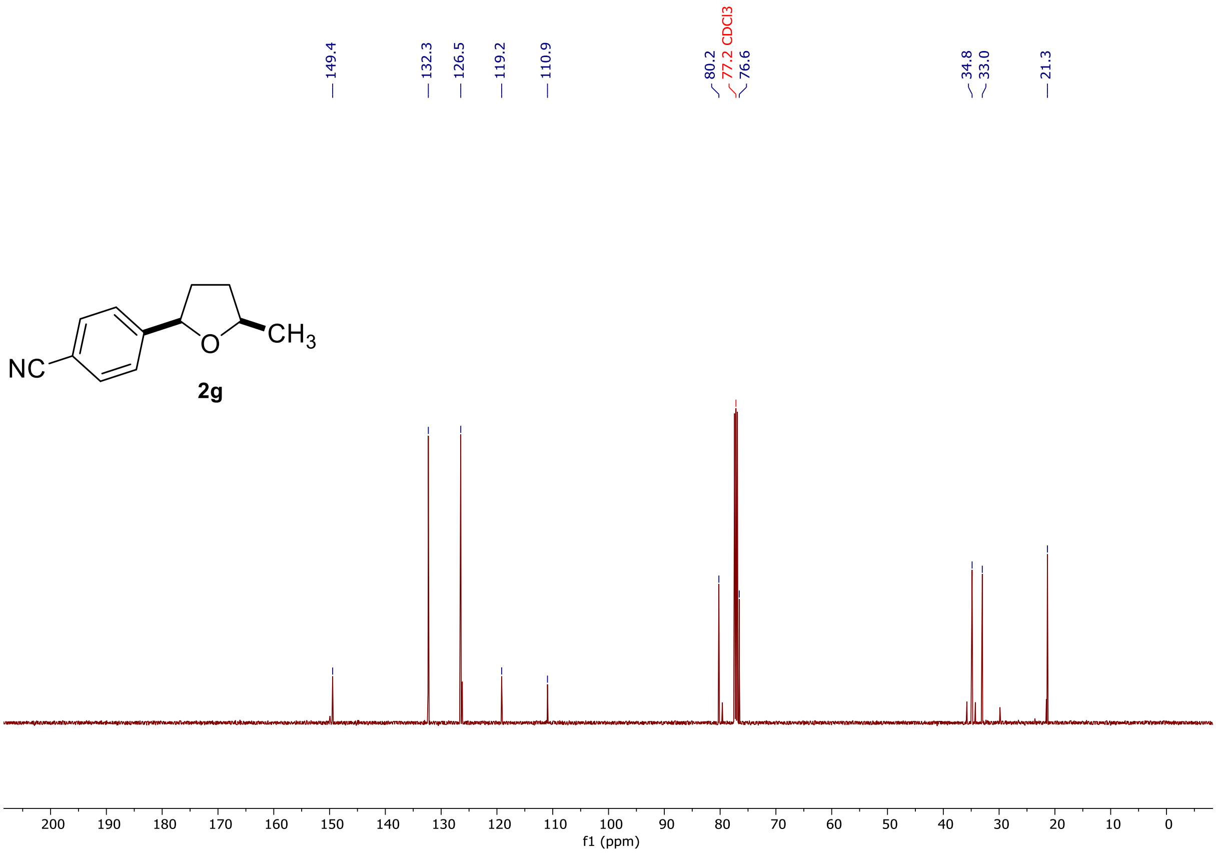

^{1}H NMR (500 MHz, $CDCl_3$) of **2h**

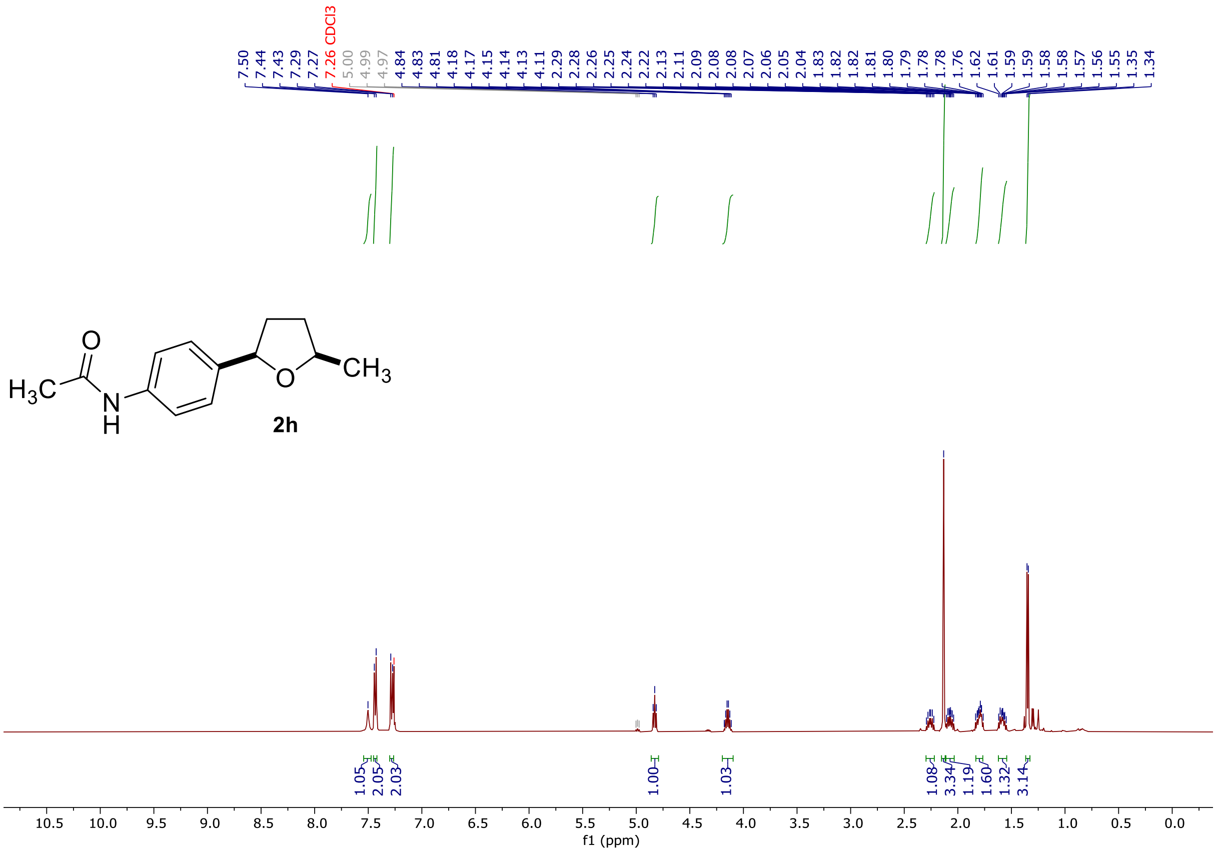

^{13}C{^{1}H} NMR (126 MHz, $CDCl_3$) of **2h**

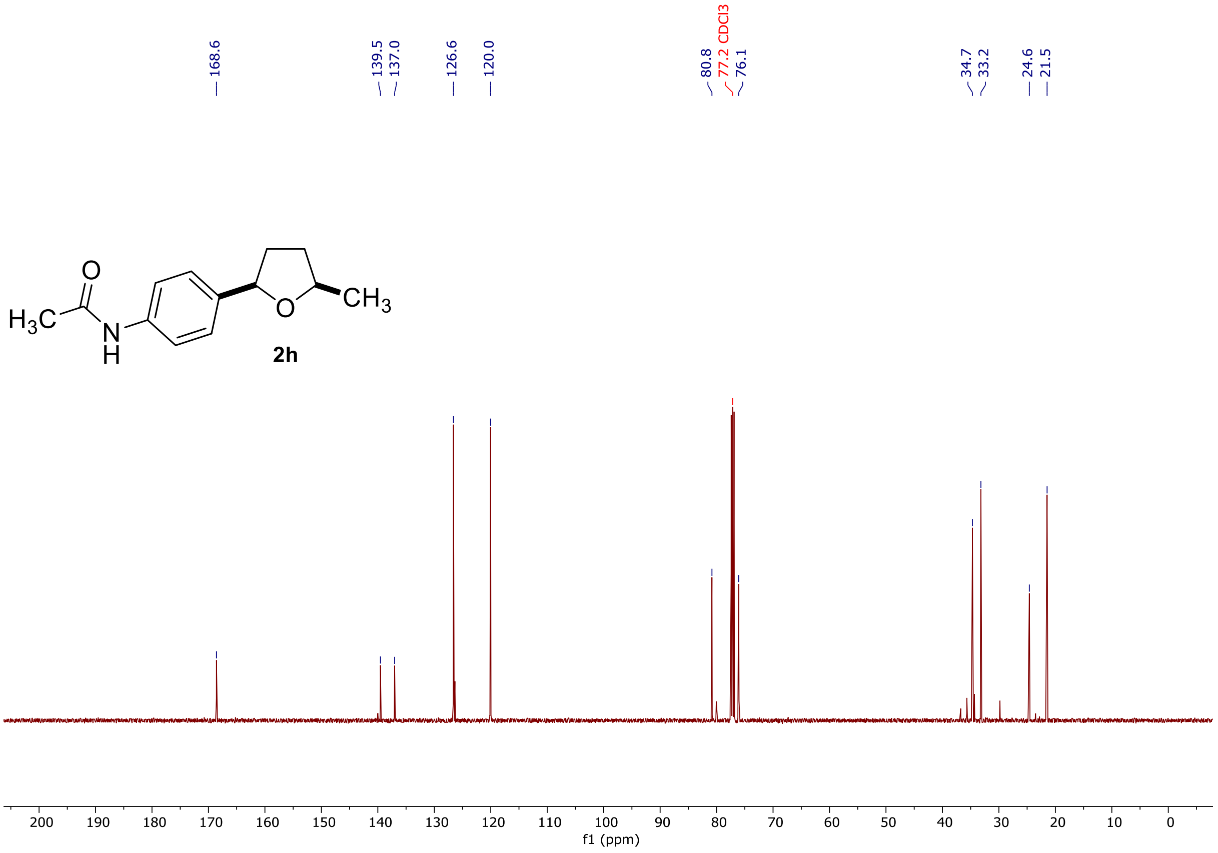

^{1}H NMR (500 MHz, $CDCl_3$) of **2i**

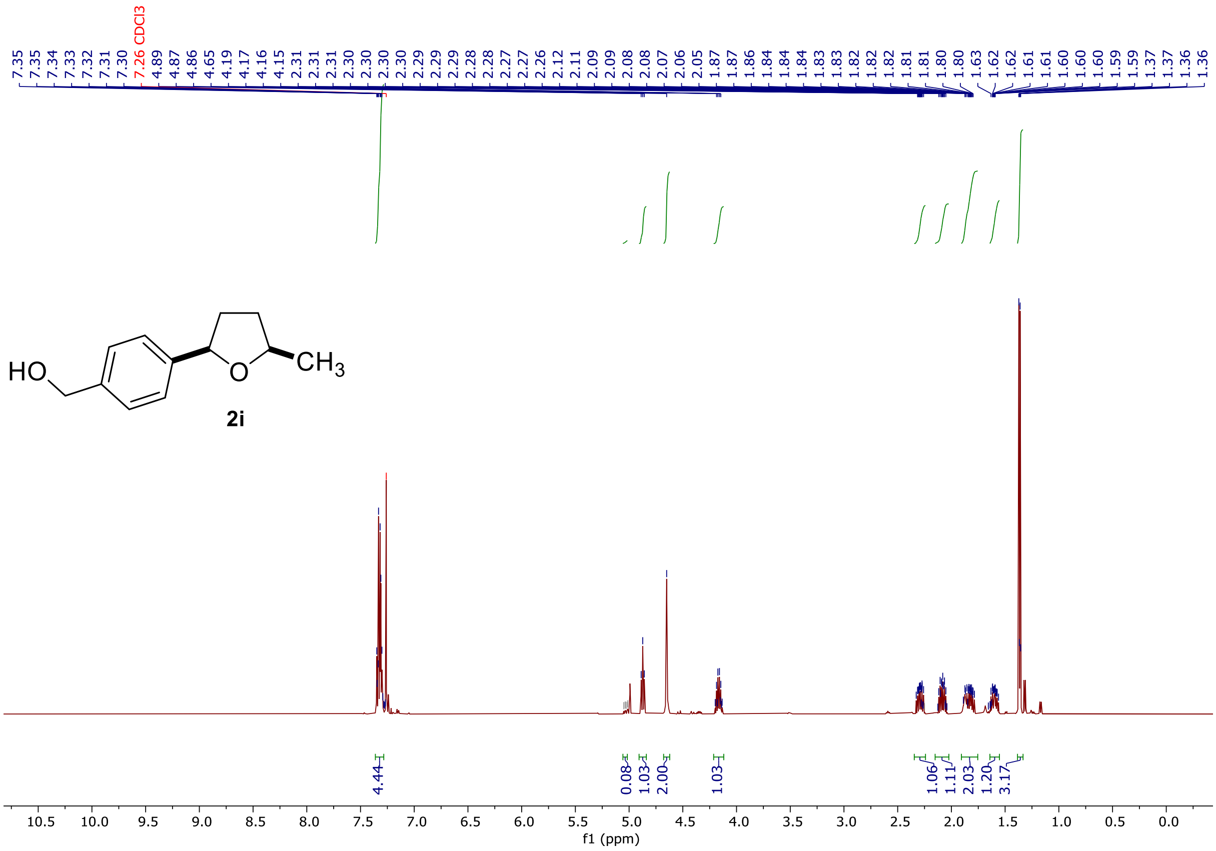

^{13}C{^{1}H} NMR (126 MHz, $CDCl_3$) of **2i**

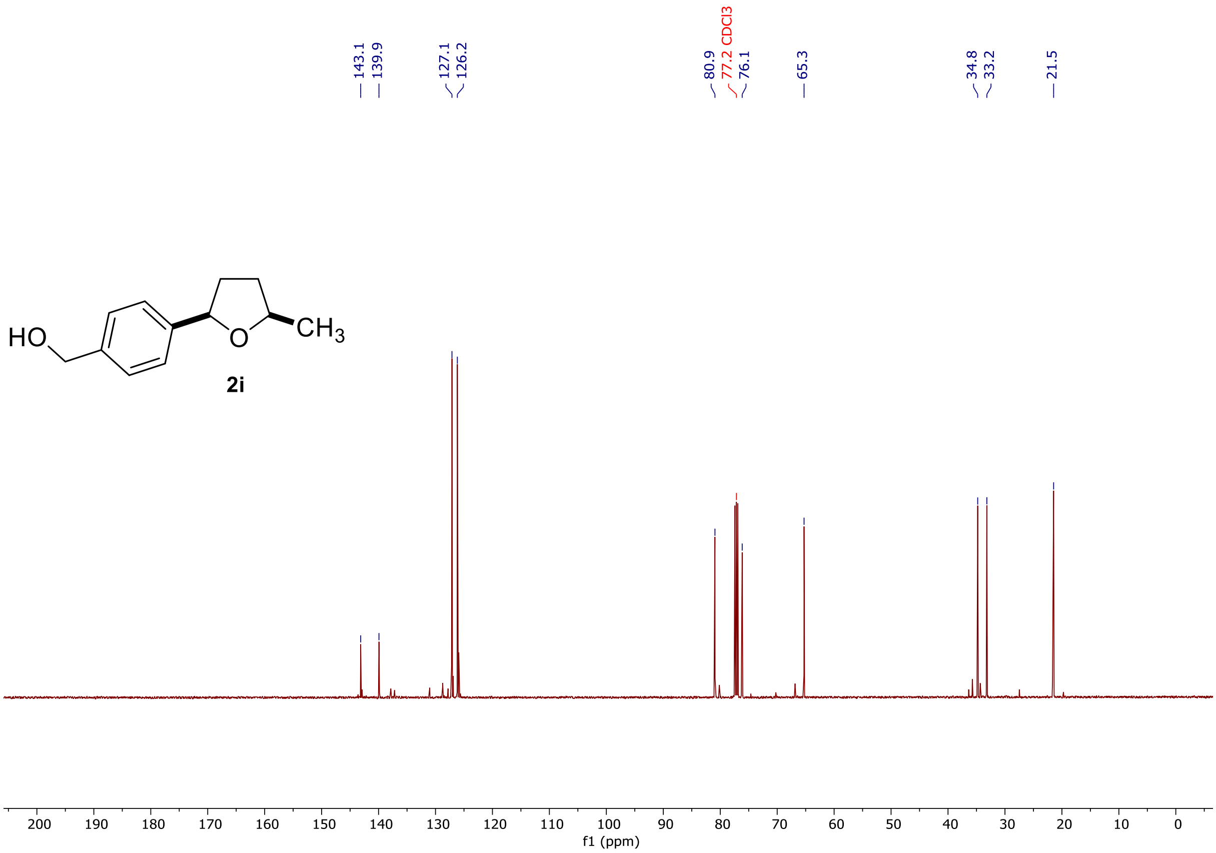

^1H NMR (500 MHz, $CDCl_3$) of **2j**

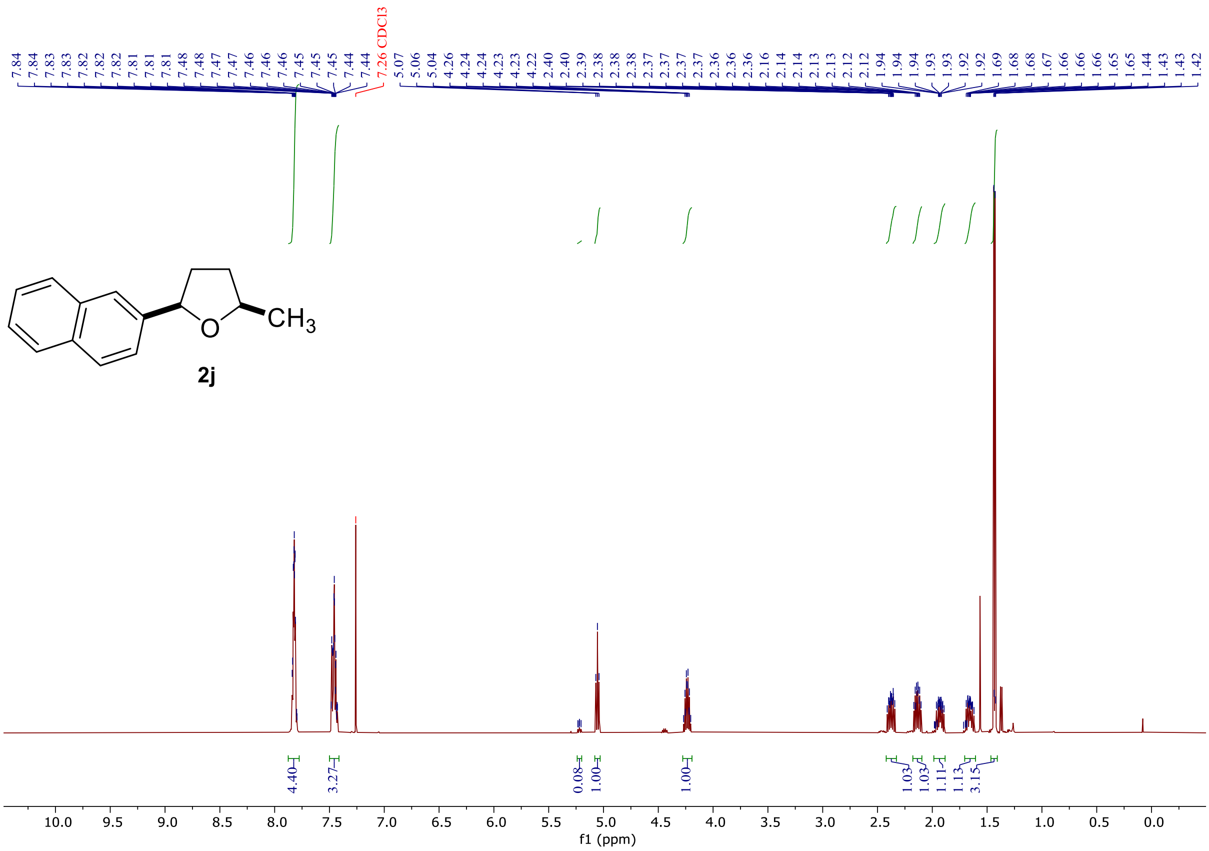

$^{13}C\{^1H\}$ NMR (126 MHz, $CDCl_3$) of **2j**

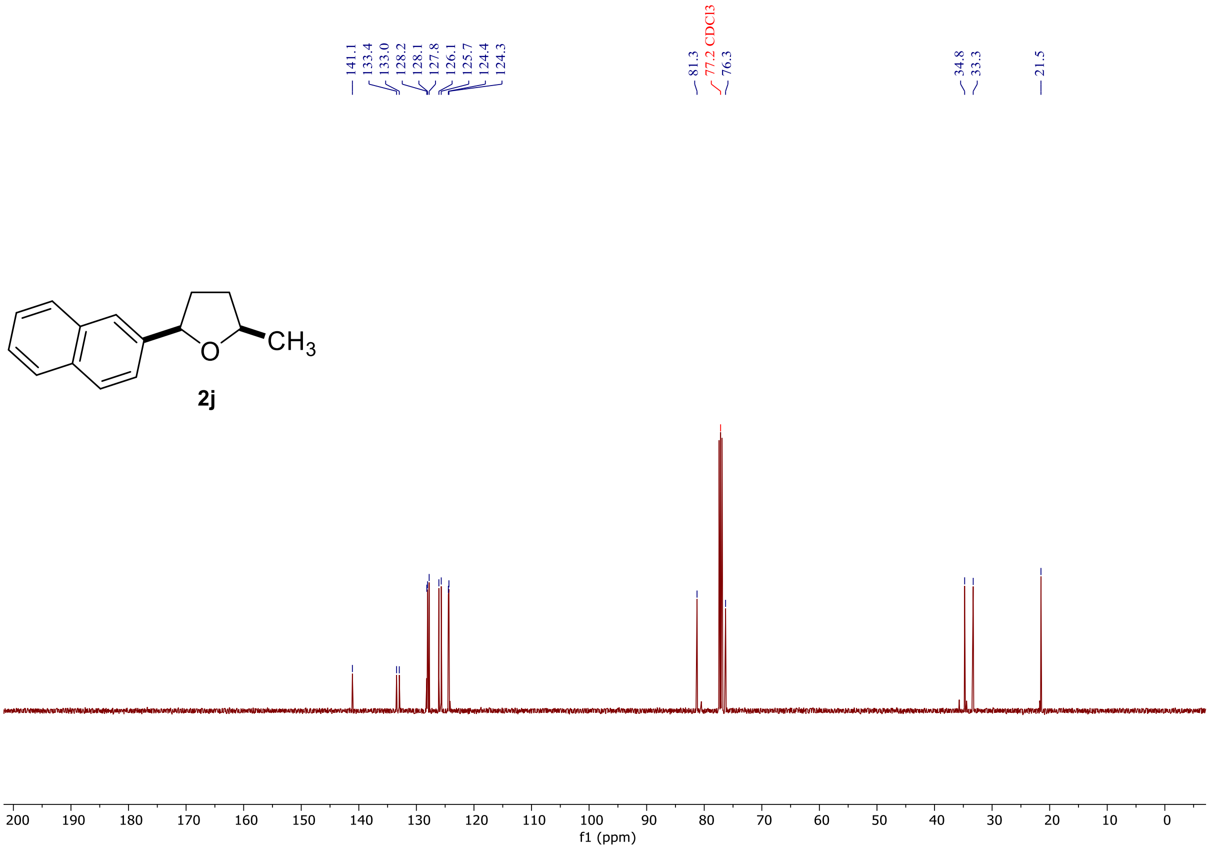

^{1}H NMR (500 MHz, $CDCl_3$) of **2k**

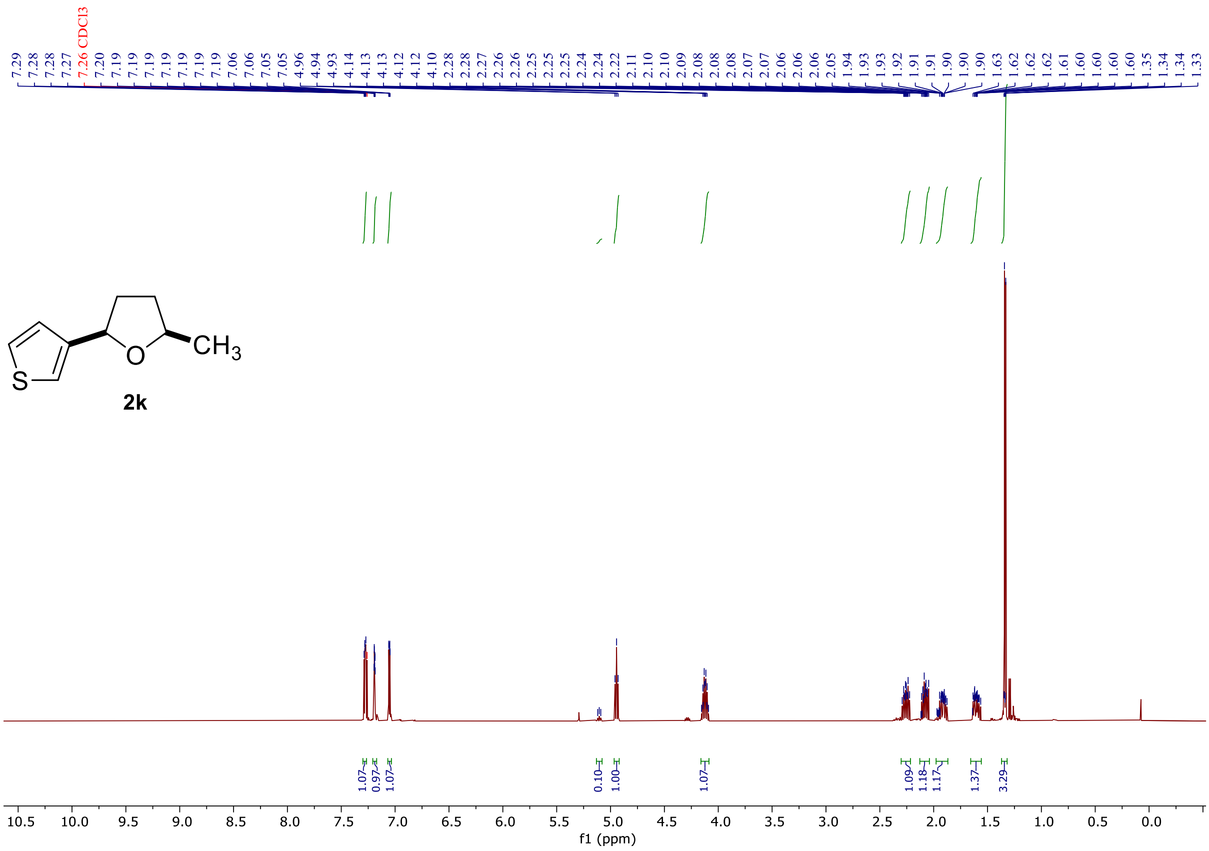

^{13}C{^{1}H} NMR (126 MHz, $CDCl_3$) of **2k**

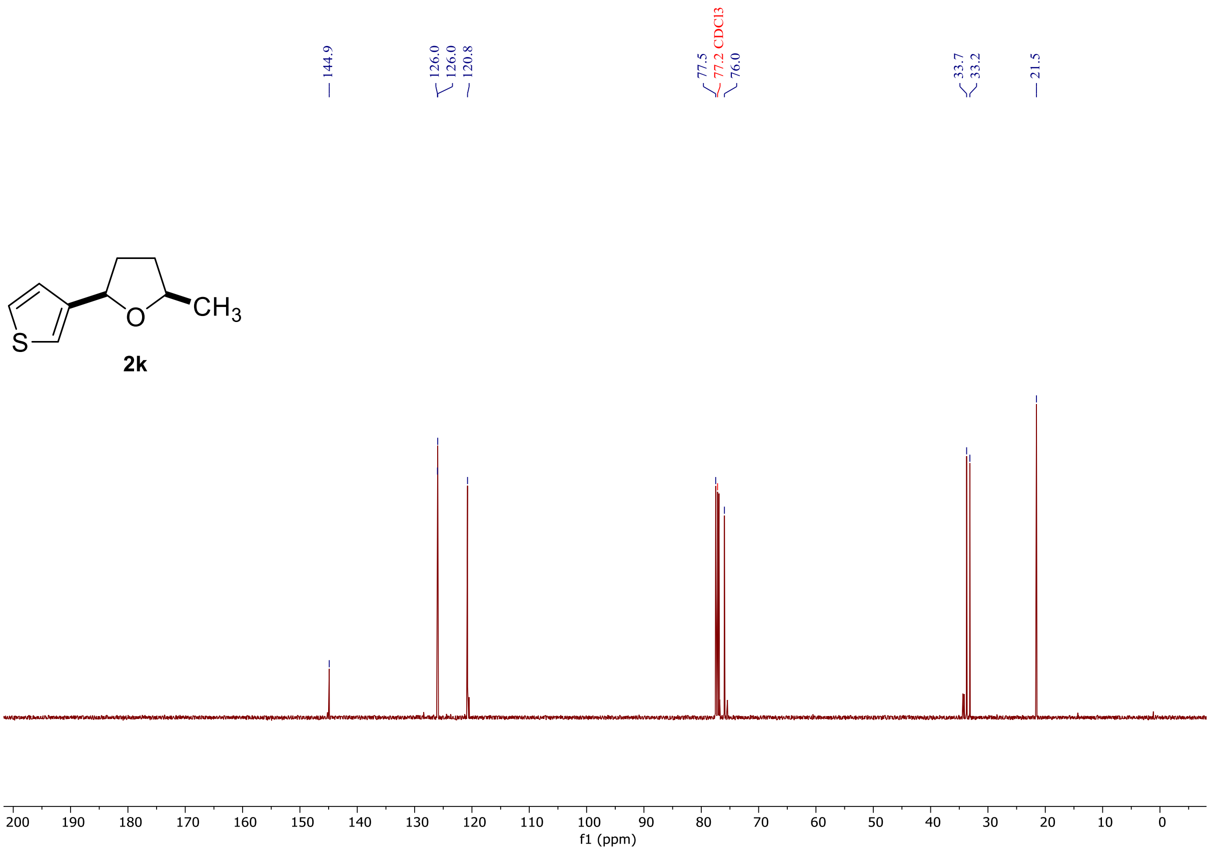

^{1}H NMR (500 MHz, $CDCl_3$) of **2l**

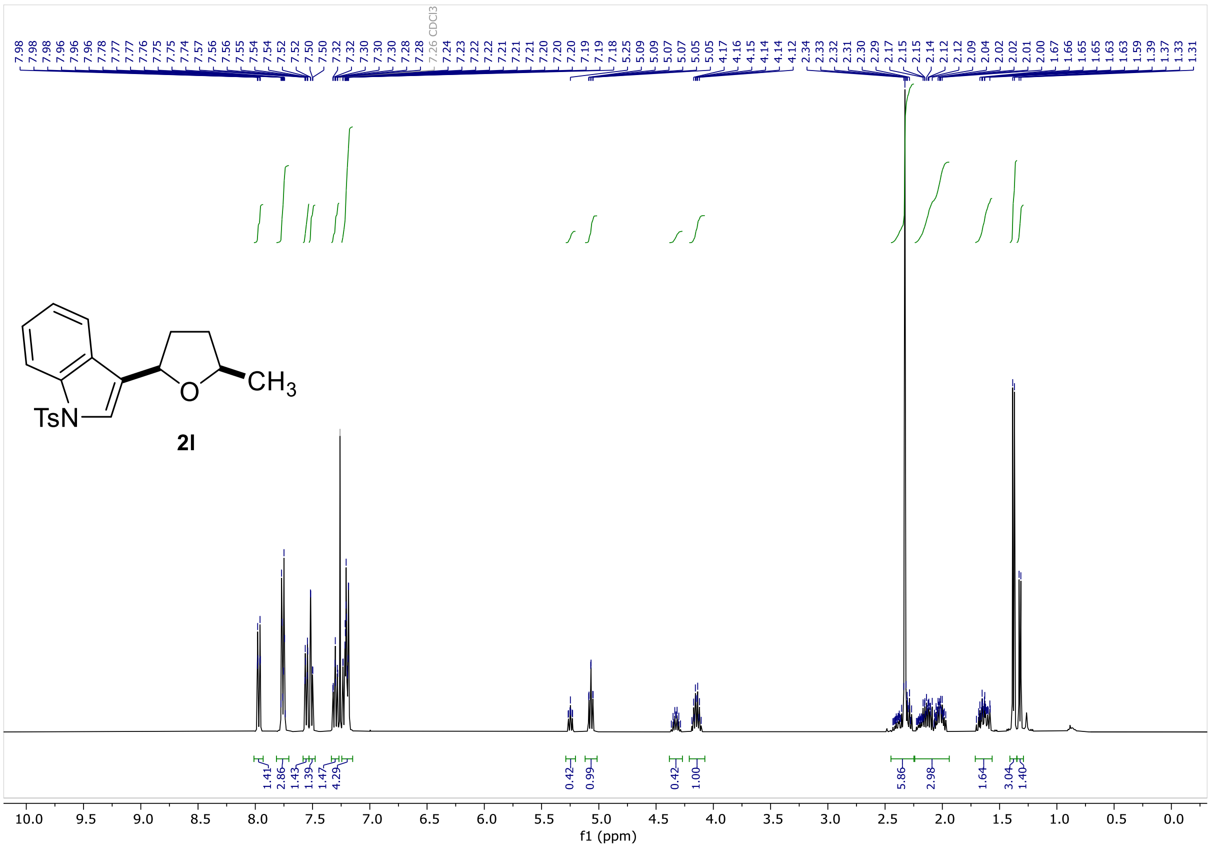

$^{13}C\{^{1}H\}$ NMR (126 MHz, $CDCl_3$) of **2l**

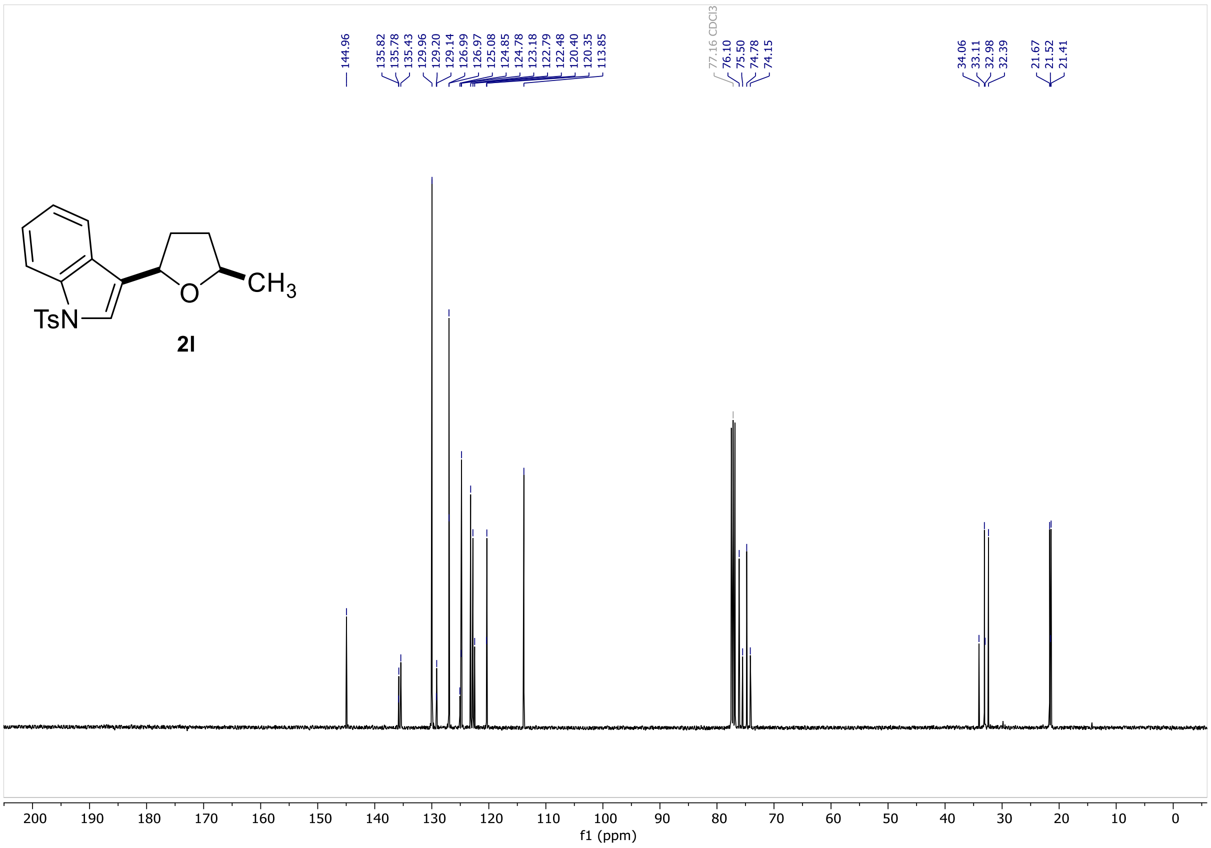

^{1}H NMR (500 MHz, $CDCl_3$) of **2m**

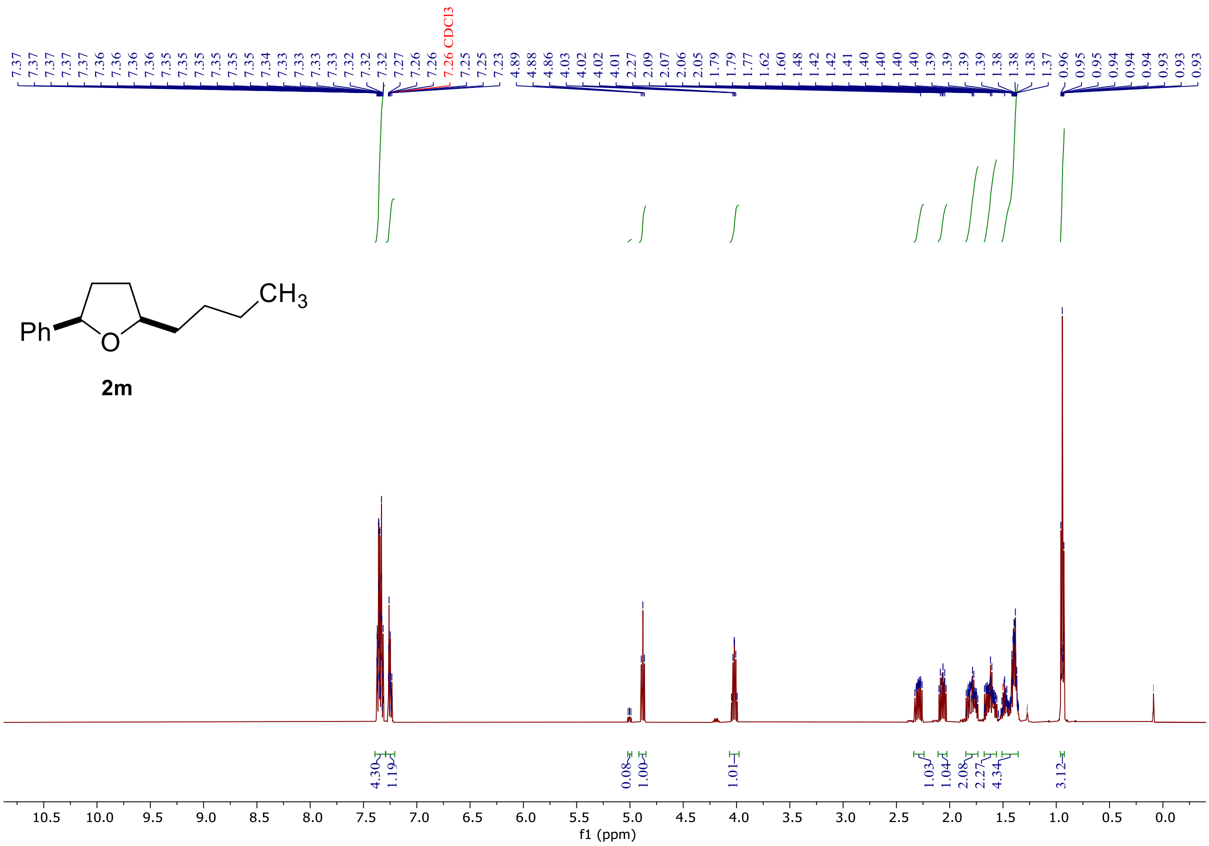

^{13}C{^{1}H} NMR (126 MHz, $CDCl_3$) of **2m**

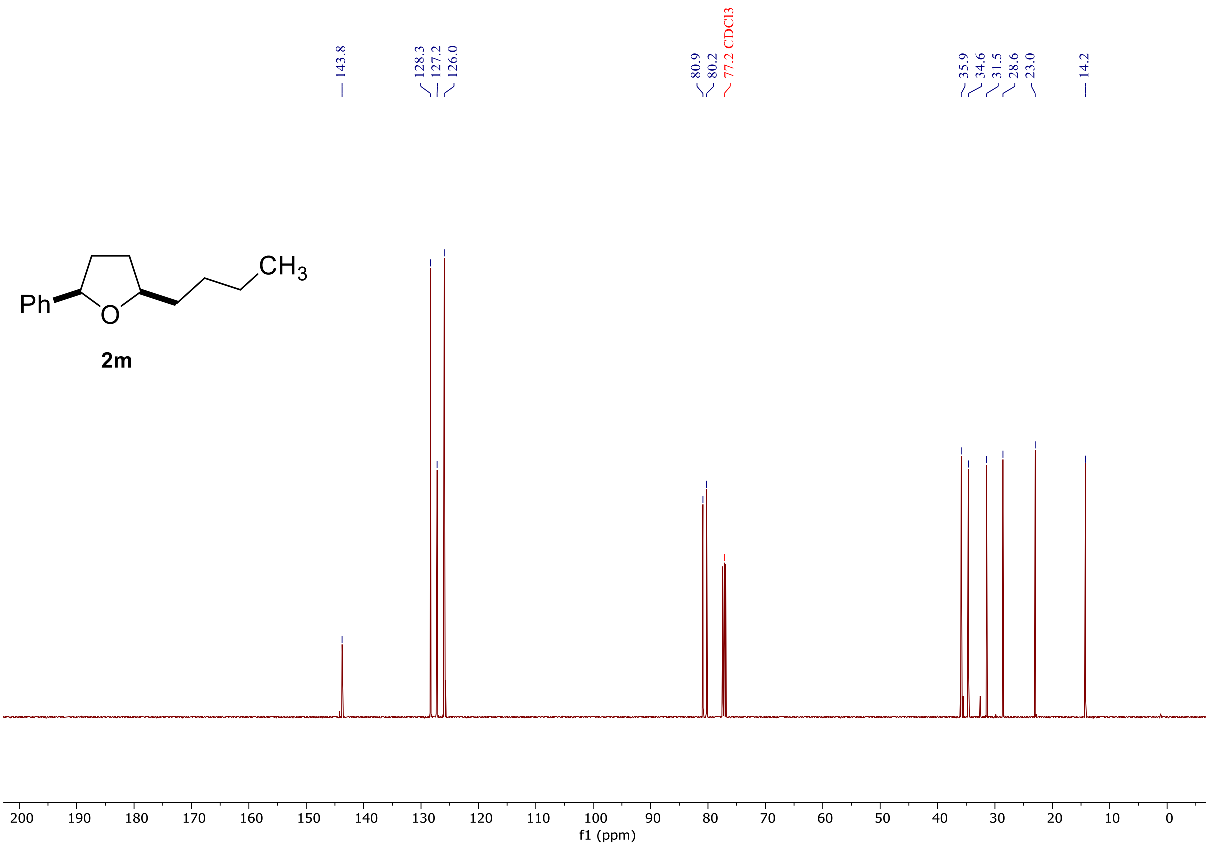

^{1}H NMR (500 MHz, $CDCl_3$) of **2n**

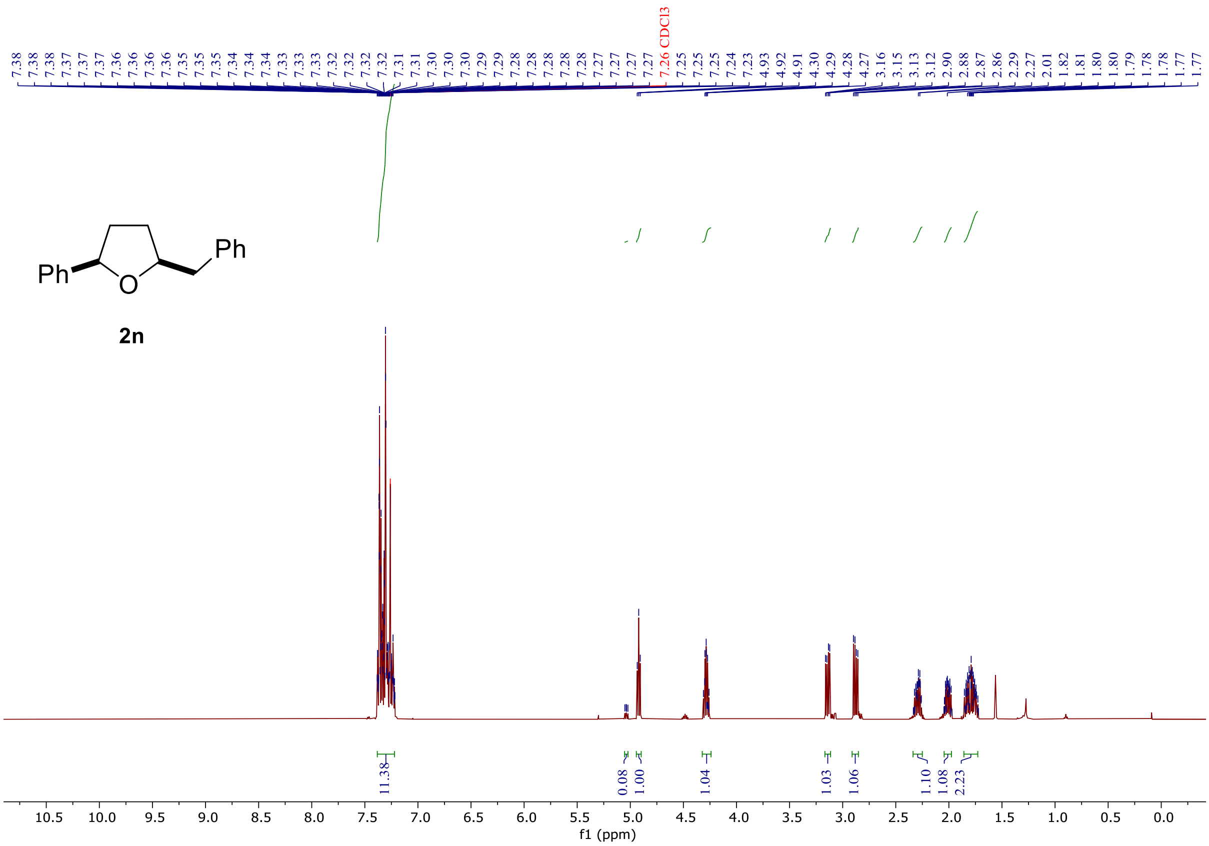

^{13}C{^{1}H} NMR (126 MHz, $CDCl_3$) of **2n**

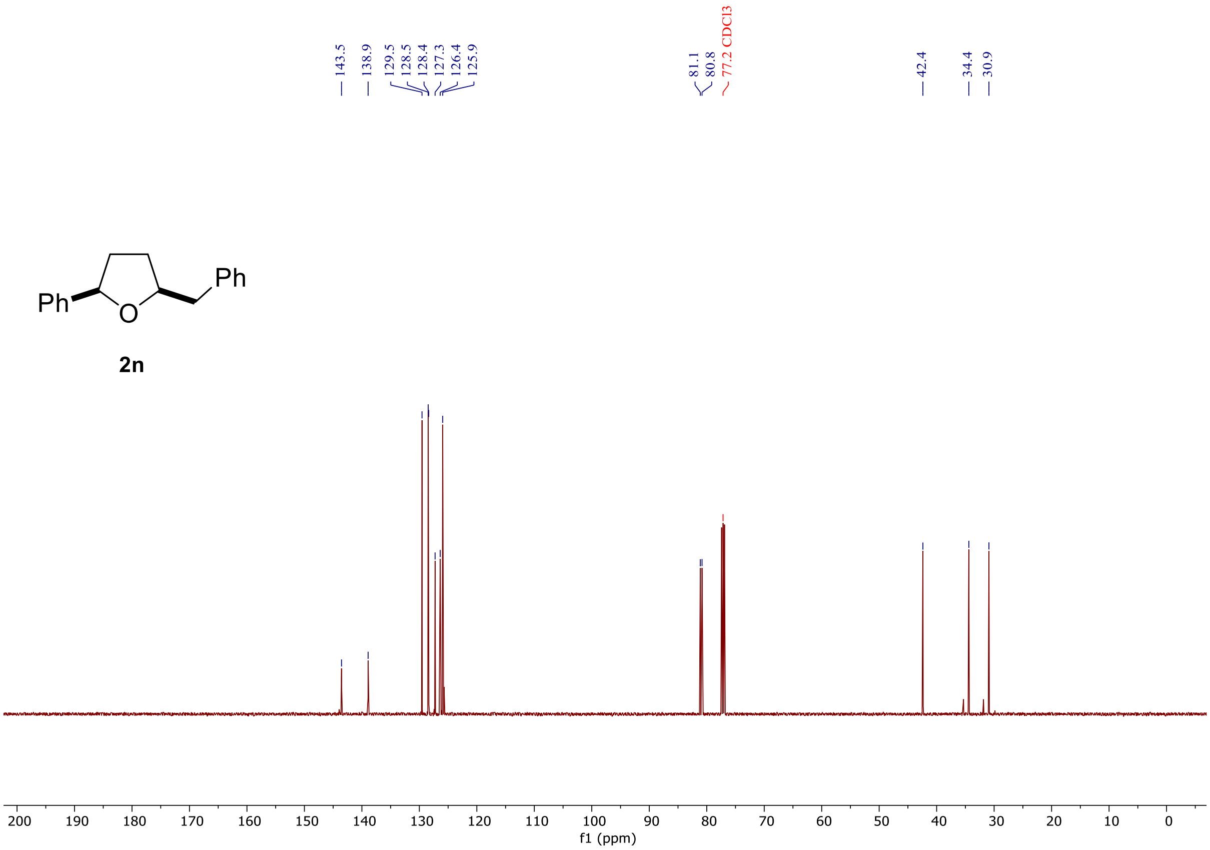

^{1}H NMR (500 MHz, CDCl$_3$) of **2o**

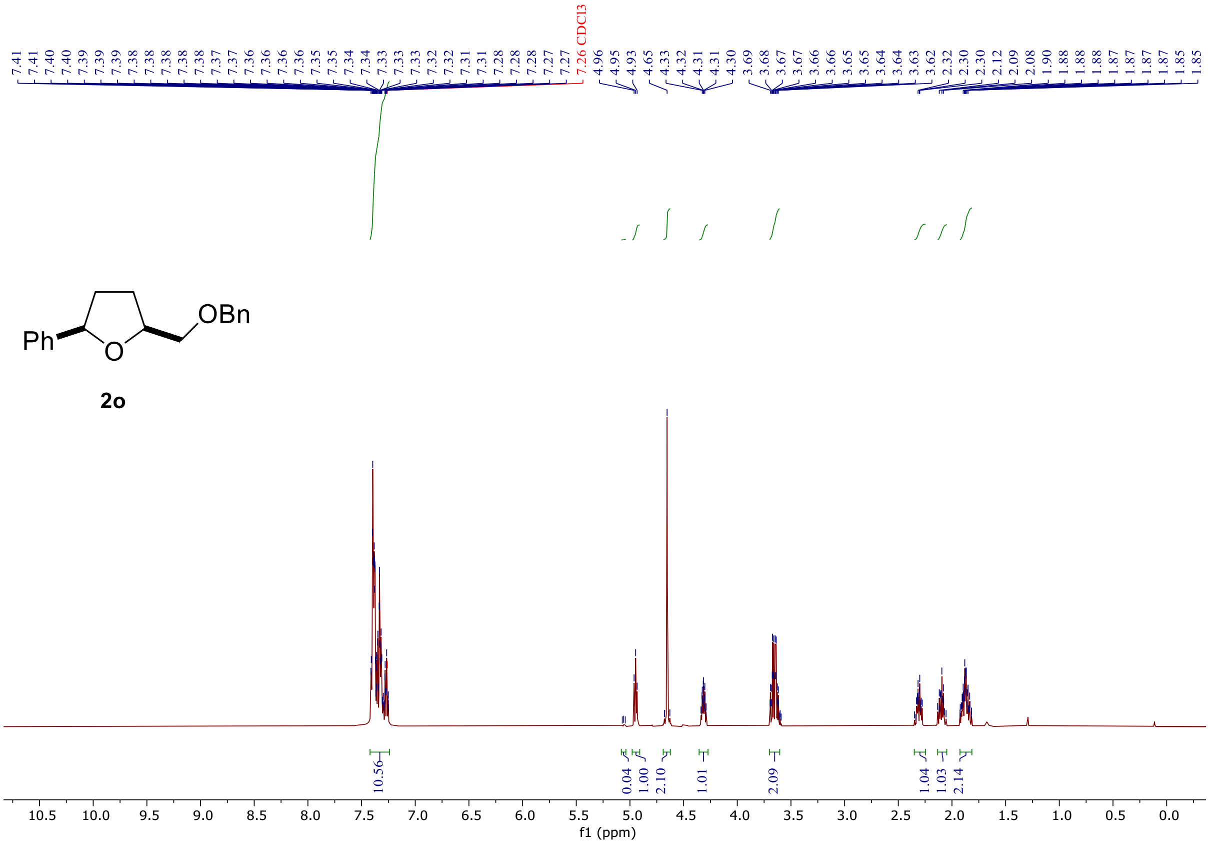

^{13}C{^{1}H} NMR (126 MHz, CDCl$_3$) of **2o**

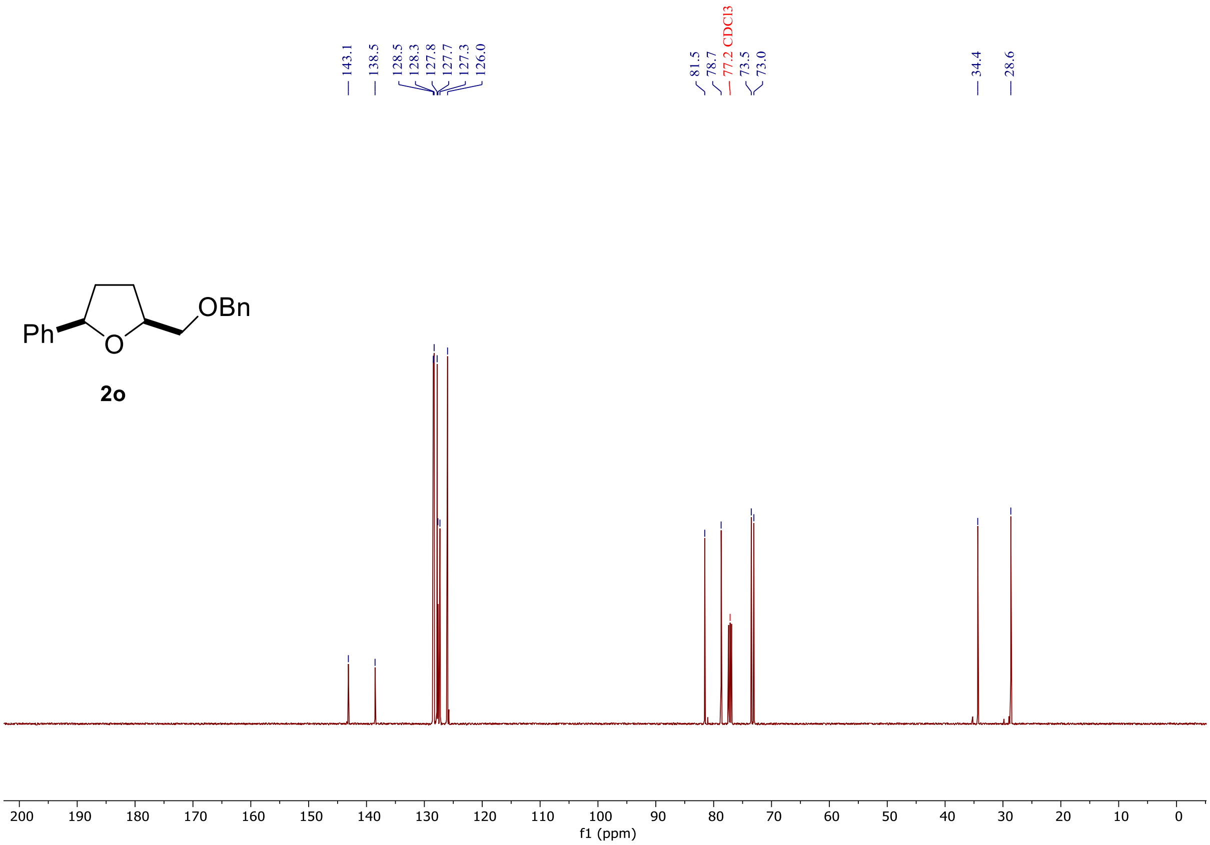

^{1}H NMR (500 MHz, $CDCl_3$) of **2p**

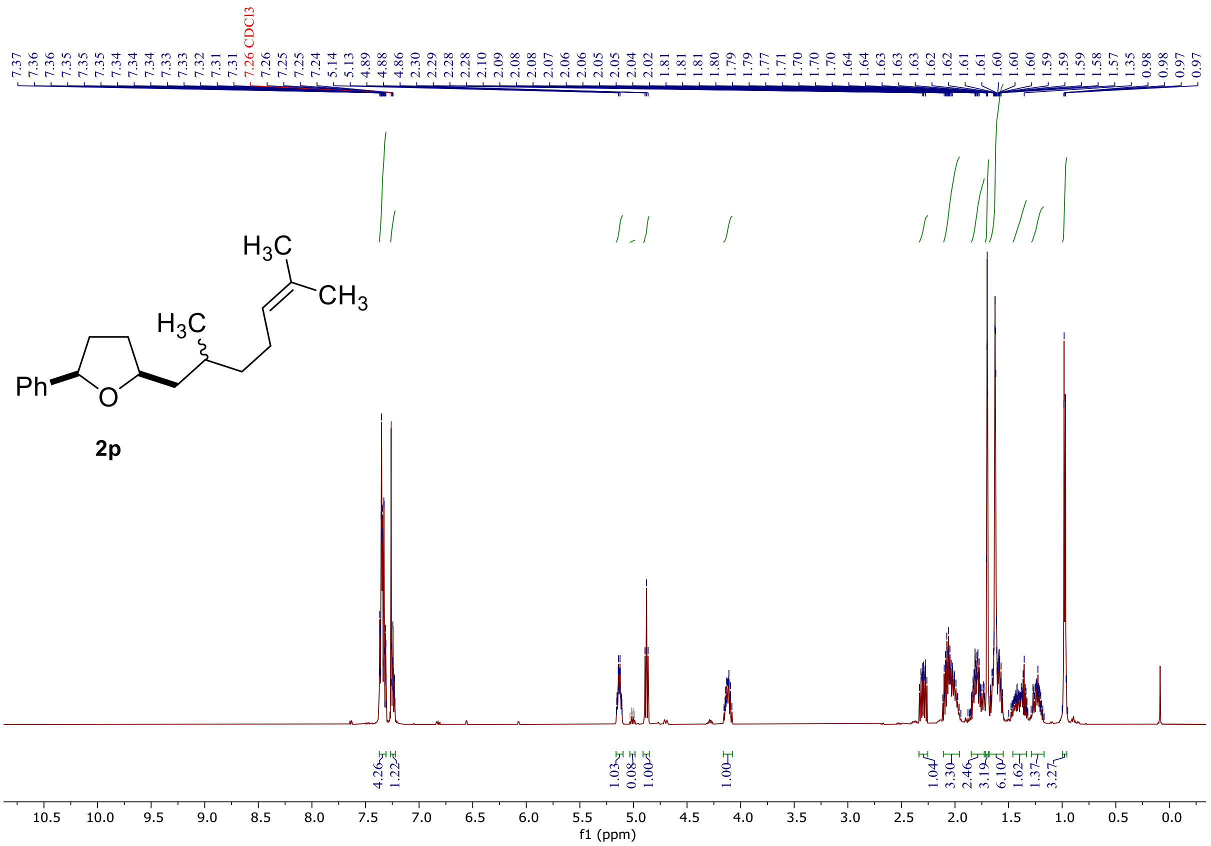

^{13}C{^{1}H} NMR (126 MHz, $CDCl_3$) of **2p**

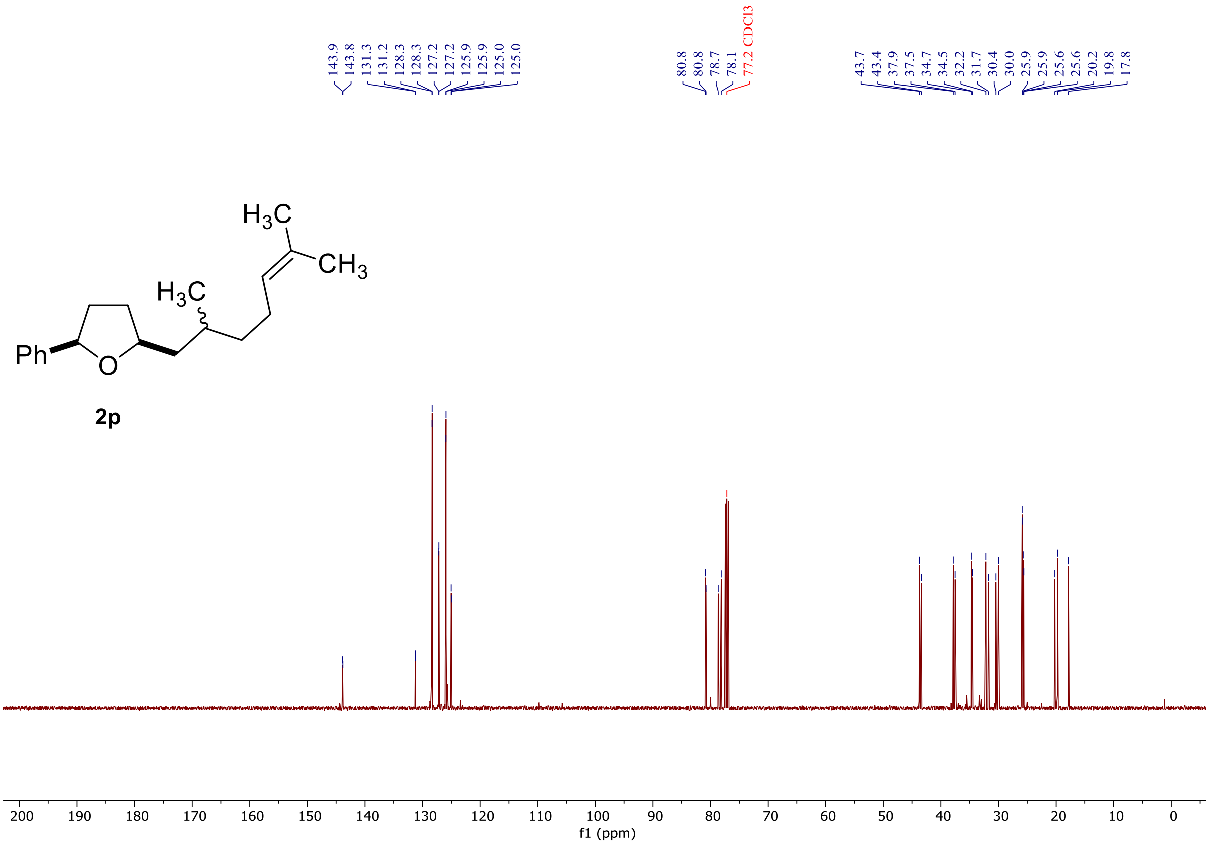

^{1}H NMR (500 MHz, $CDCl_3$) of **2q**

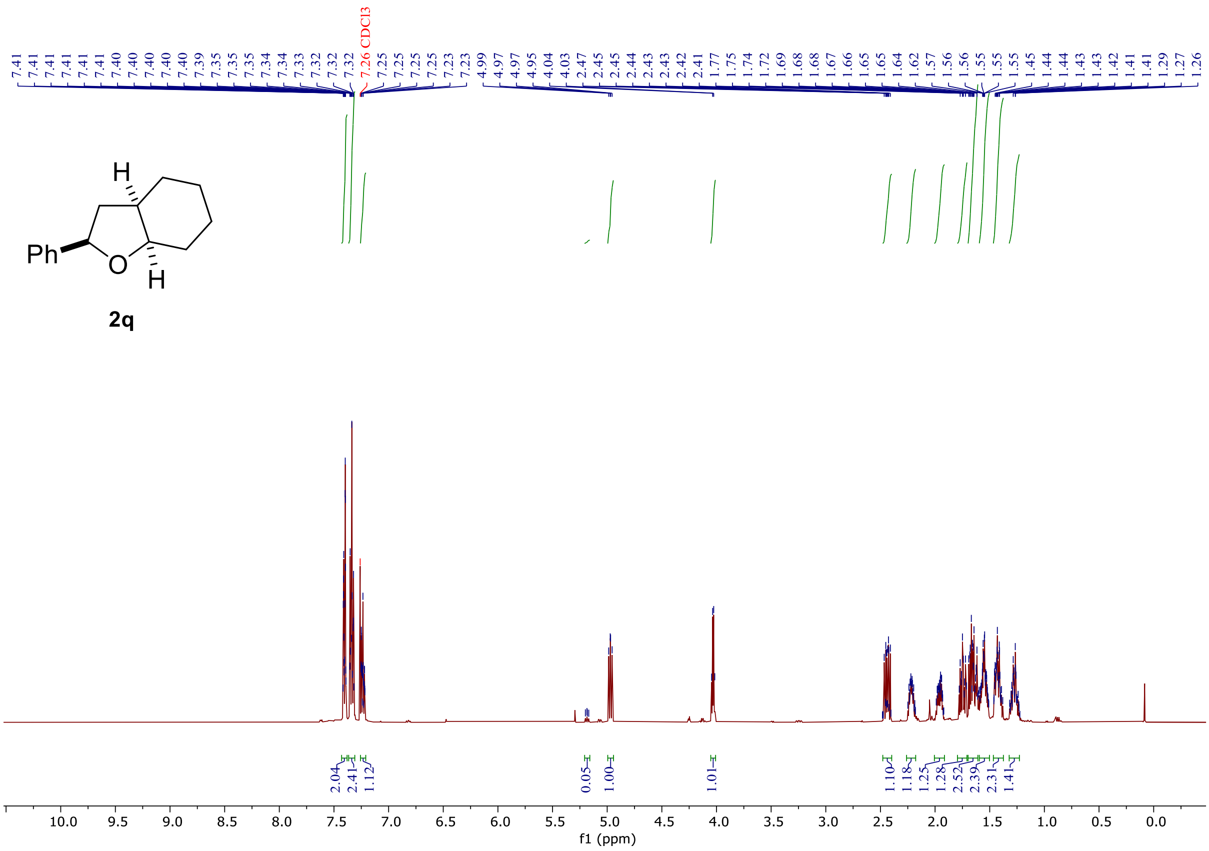

^{13}C{^{1}H} NMR (126 MHz, $CDCl_3$) of **2q**

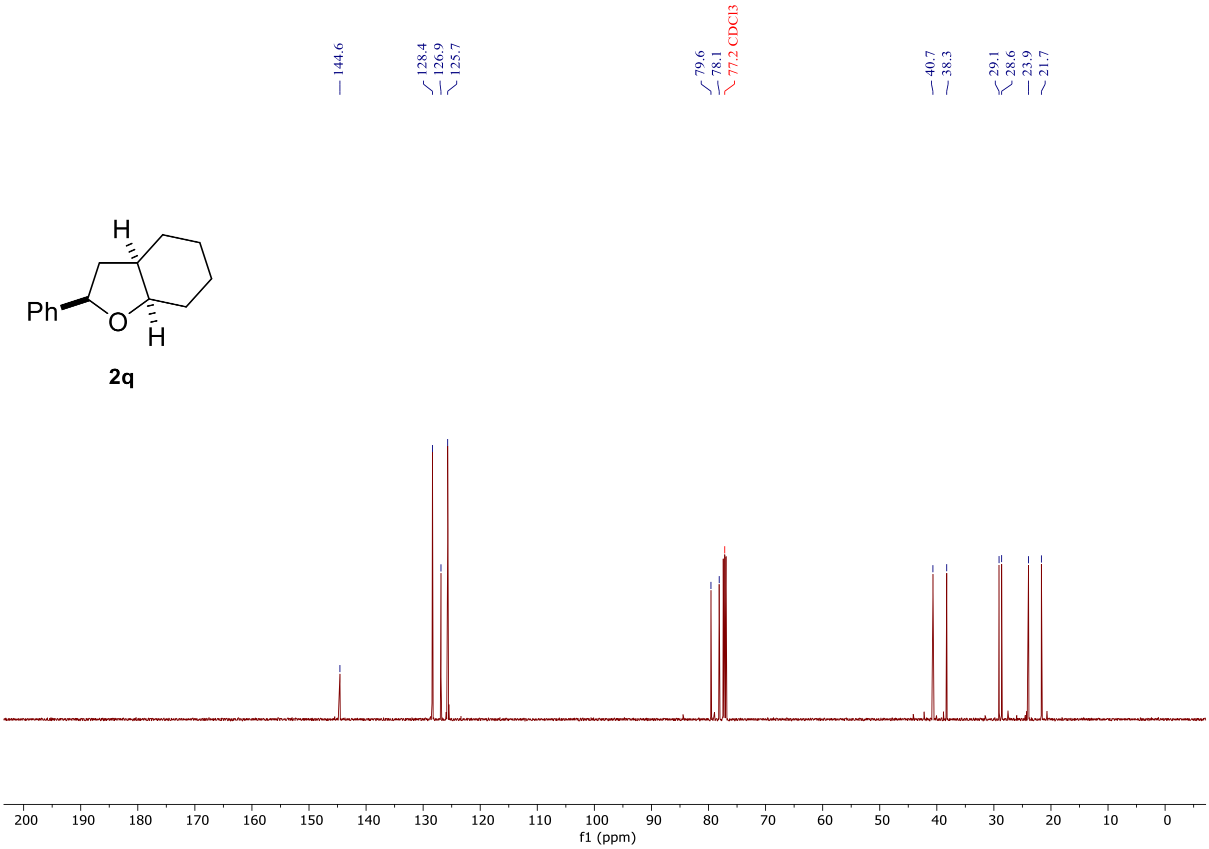

^{1}H NMR (500 MHz, $CDCl_3$) of **2r**+**2r′**

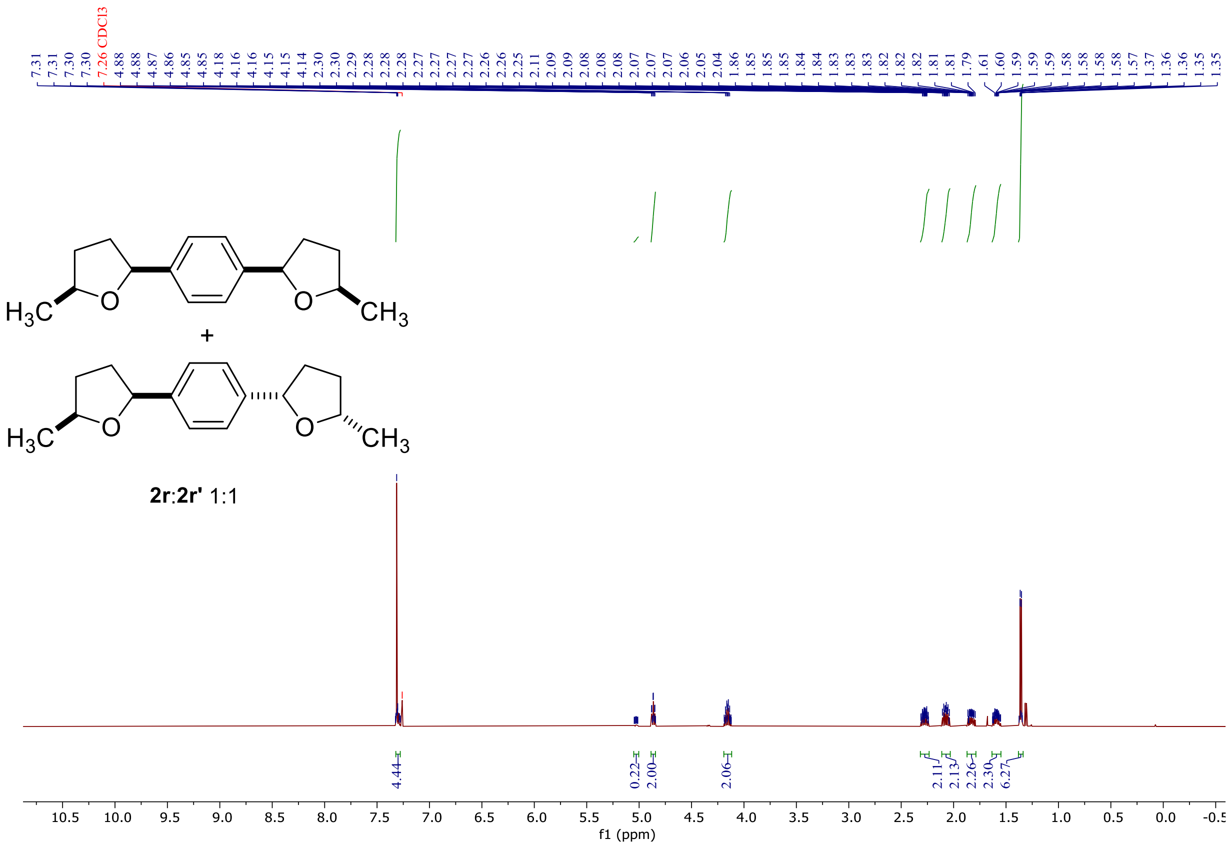

$^{13}C\{^{1}H\}$ NMR (126 MHz, $CDCl_3$) of **2r**+**2r′**

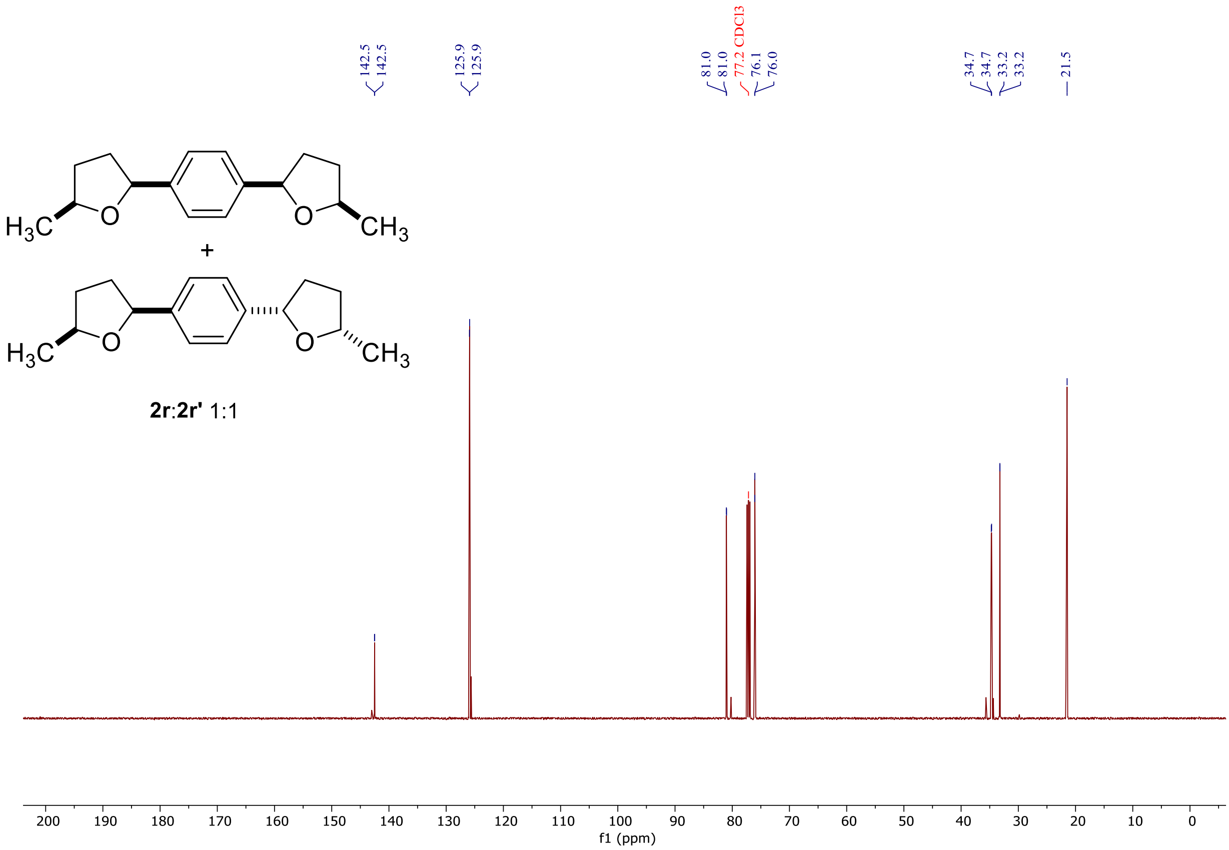

^{1}H NMR (500 MHz, $CDCl_3$) of **2s**

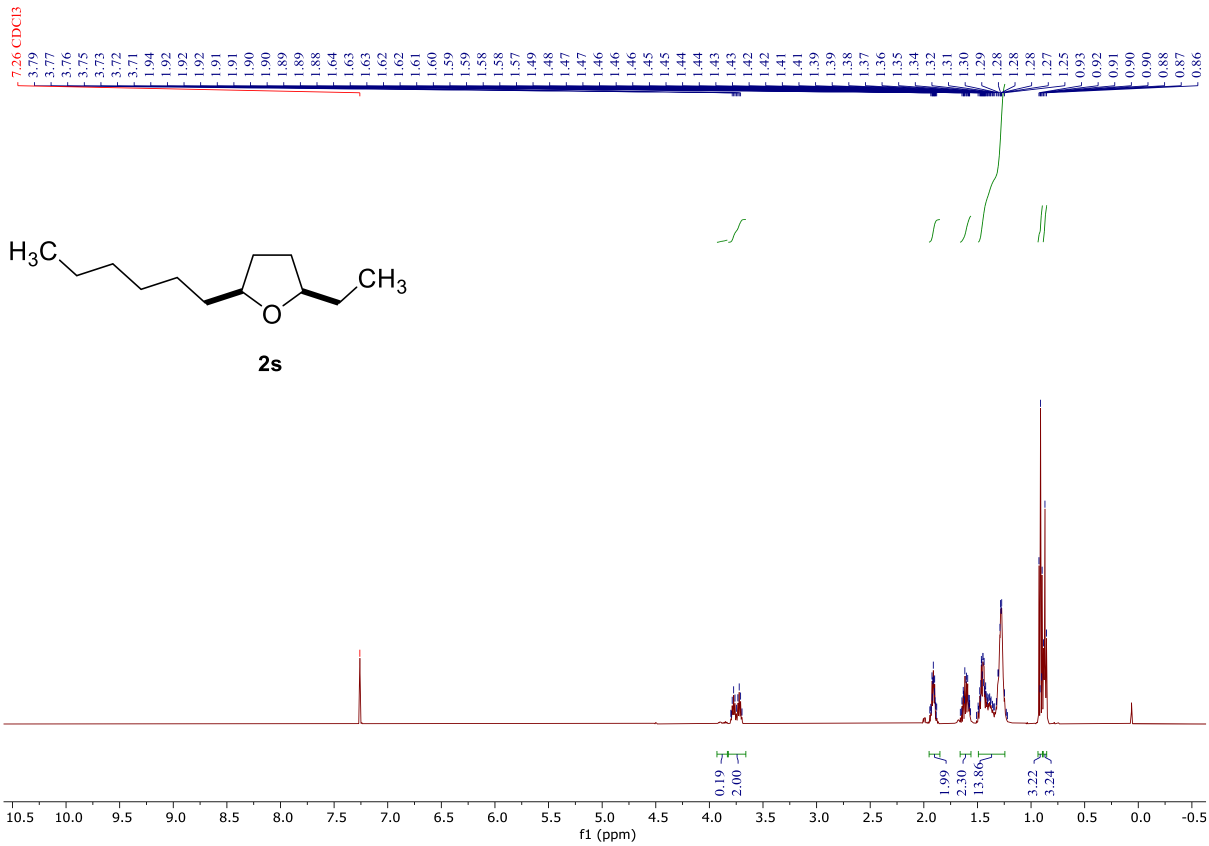

$^{13}C\{^{1}H\}$ NMR (126 MHz, $CDCl_3$) of **2s**

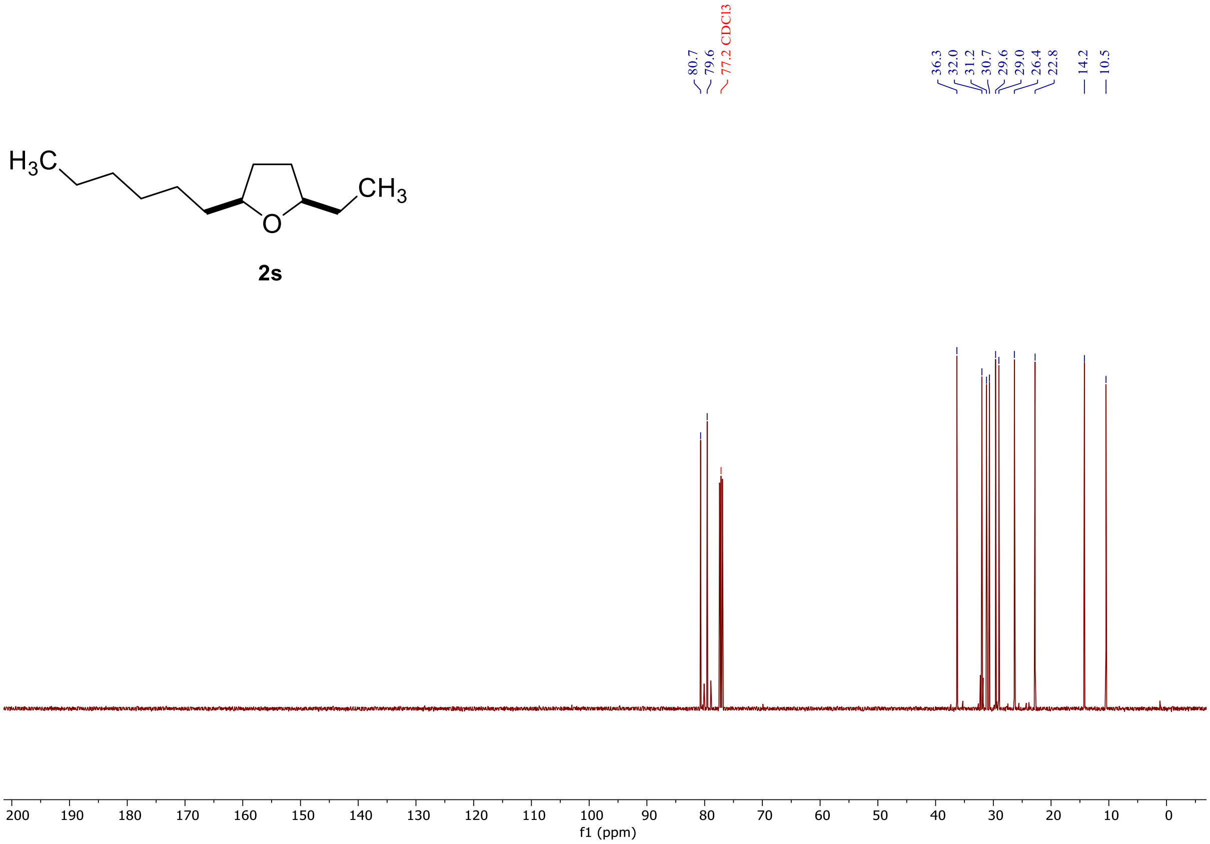

^{1}H NMR (500 MHz, $CDCl_3$) of **2t**

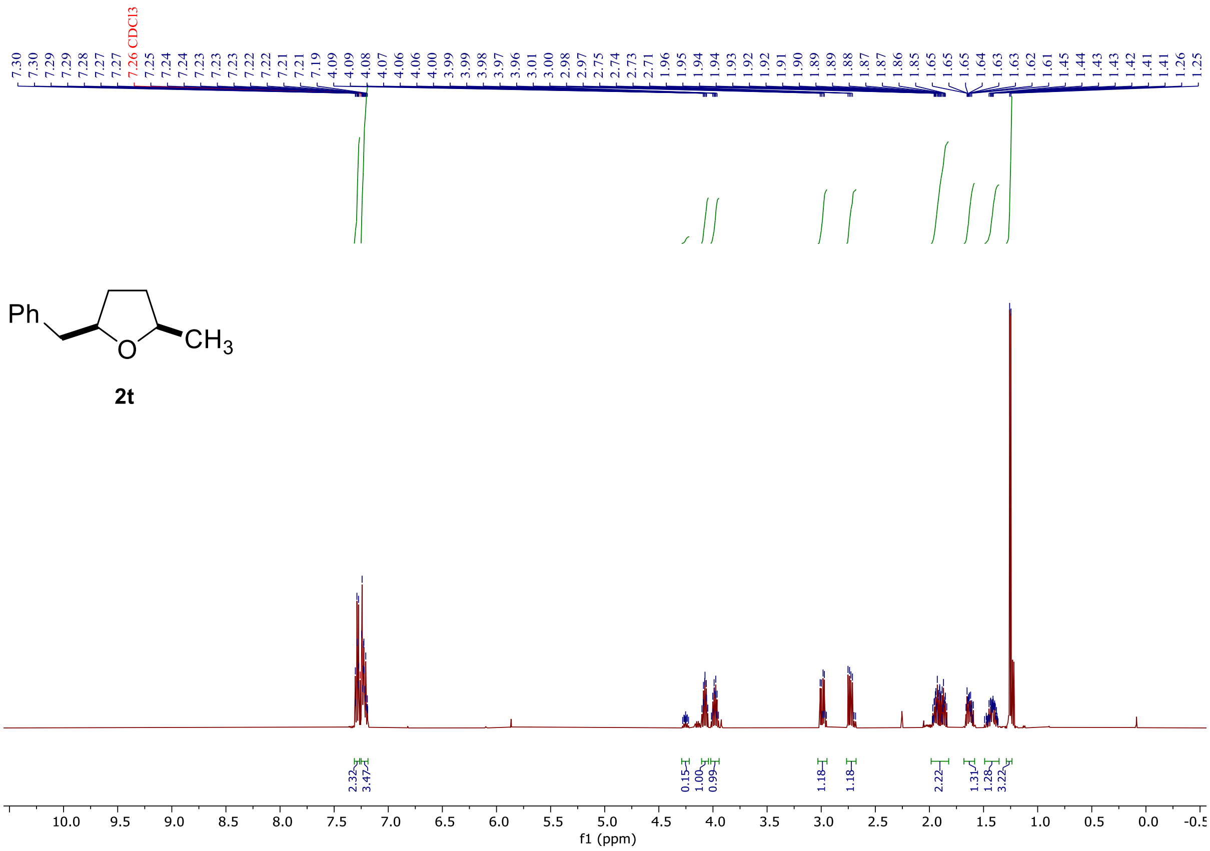

^{13}C{^{1}H} NMR (126 MHz, $CDCl_3$) of **2t**

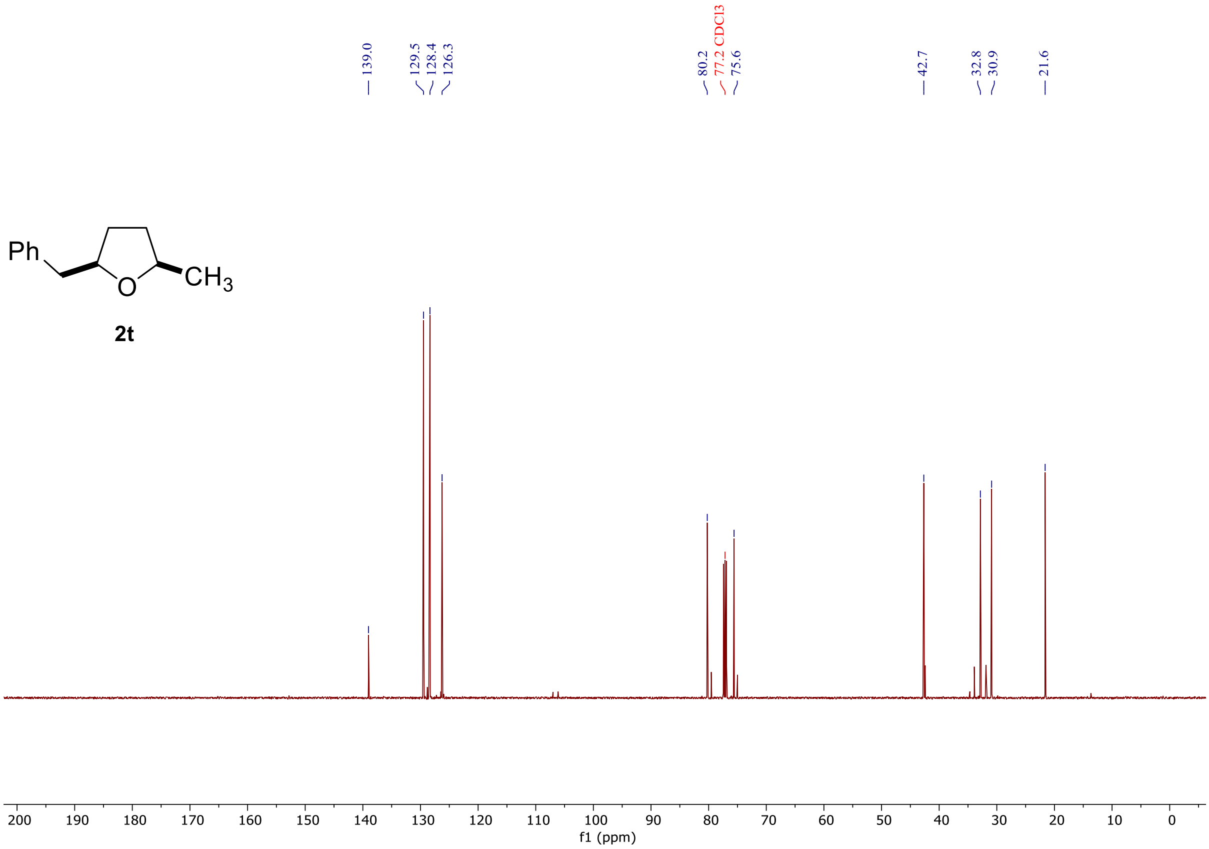

^{1}H NMR (500 MHz, $CDCl_3$) of **2u**

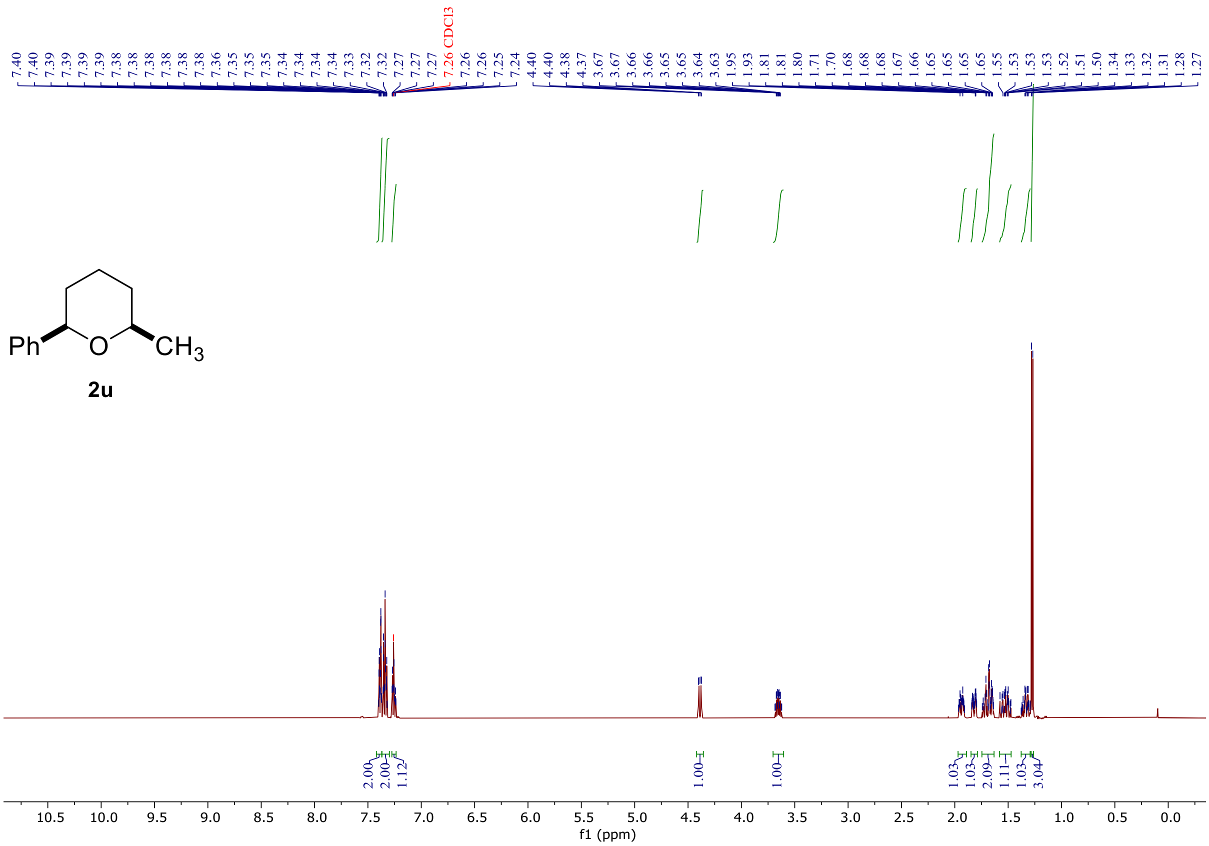

$^{13}C\{^{1}H\}$ NMR (126 MHz, $CDCl_3$) of **2u**

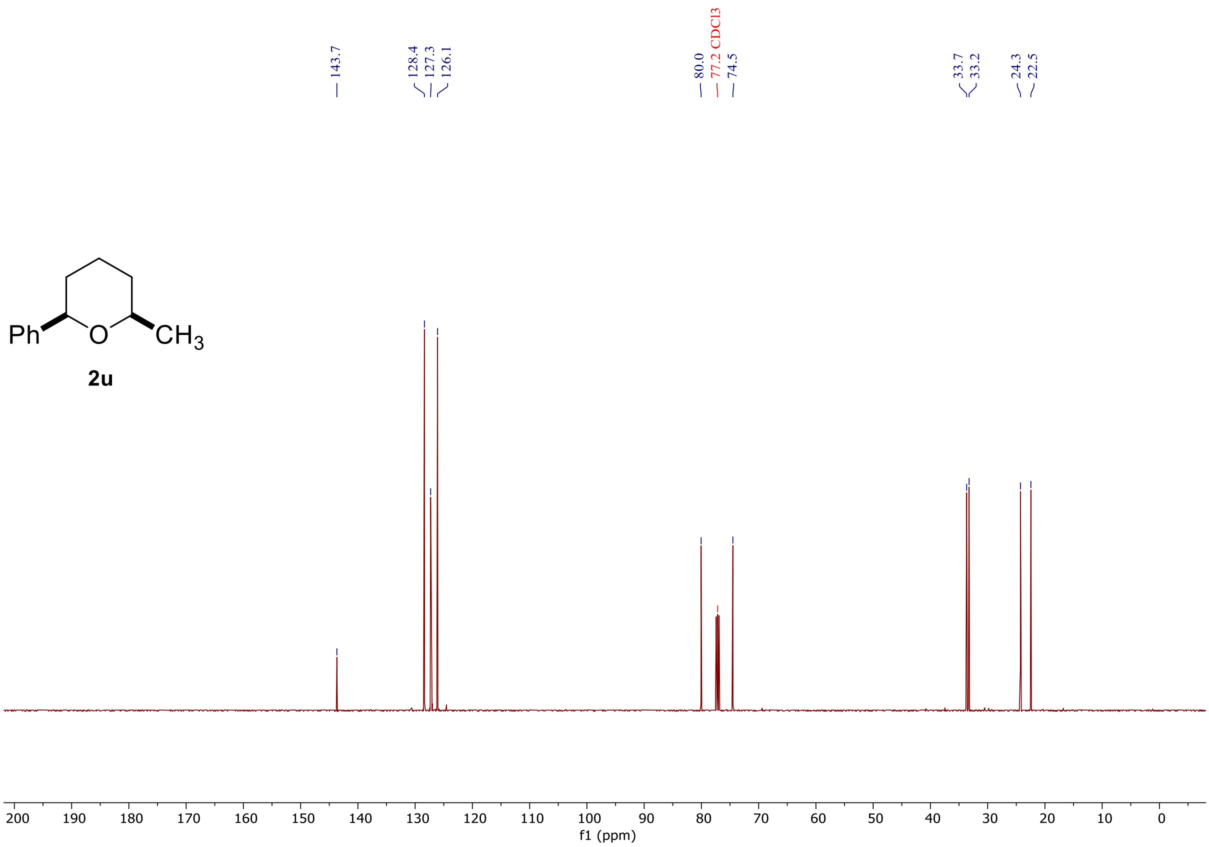

^{1}H NMR (500 MHz, $CDCl_3$) of **2v**

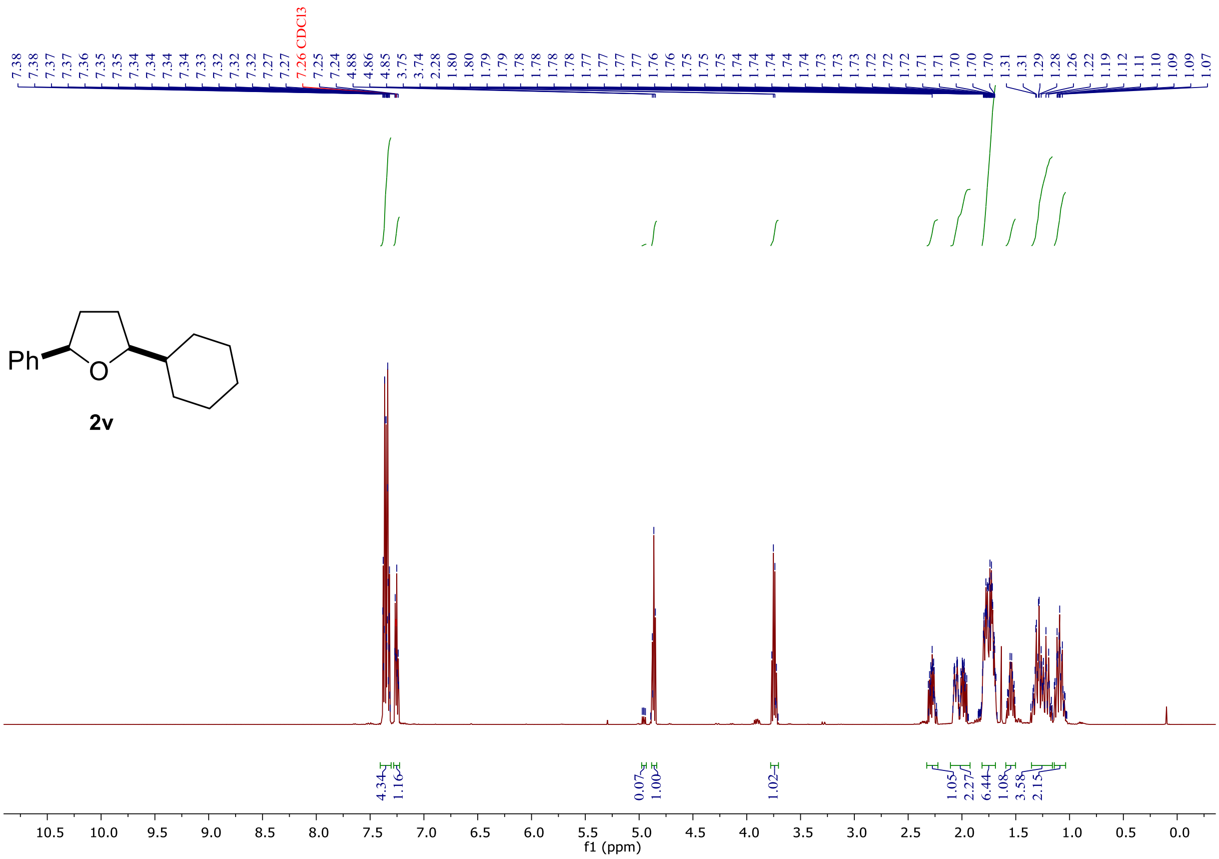

^{13}C{^{1}H} NMR (126 MHz, $CDCl_3$) of **2v**

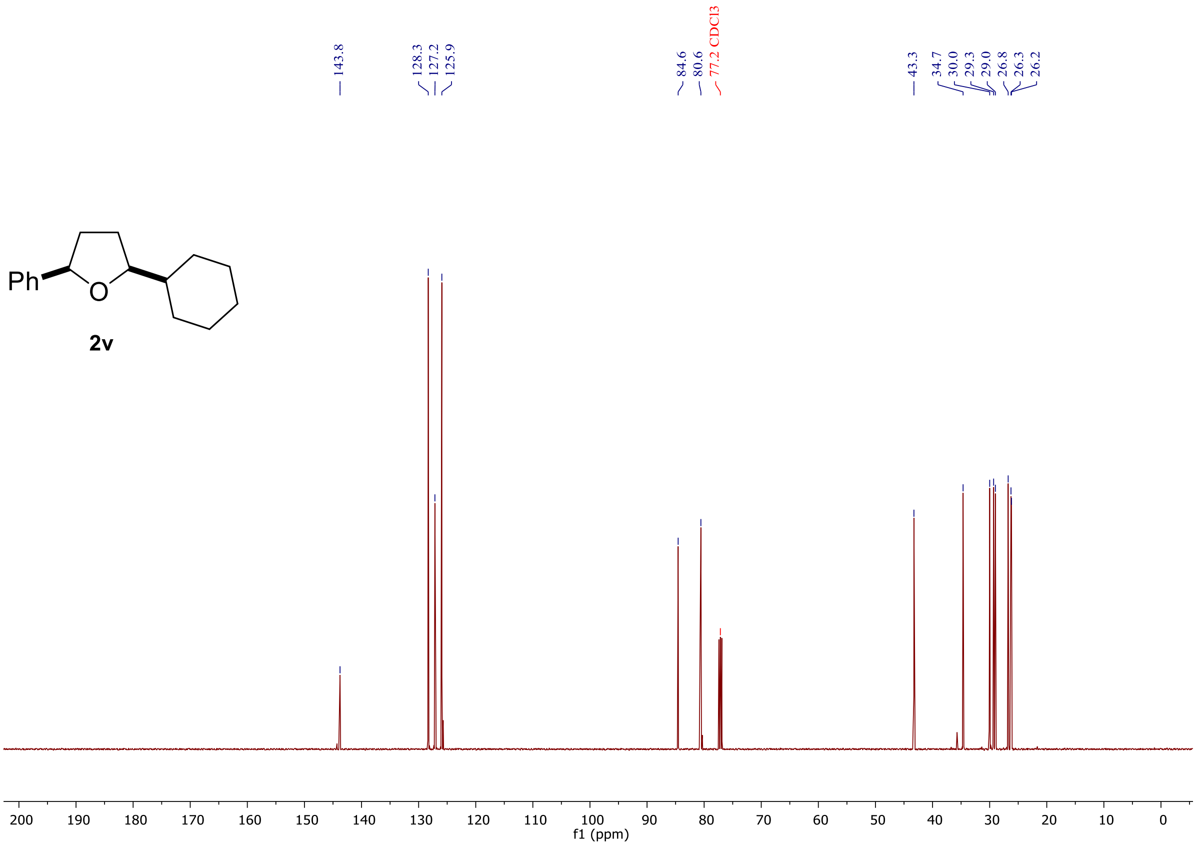

^{1}H NMR (500 MHz, $CDCl_3$) of **2w**

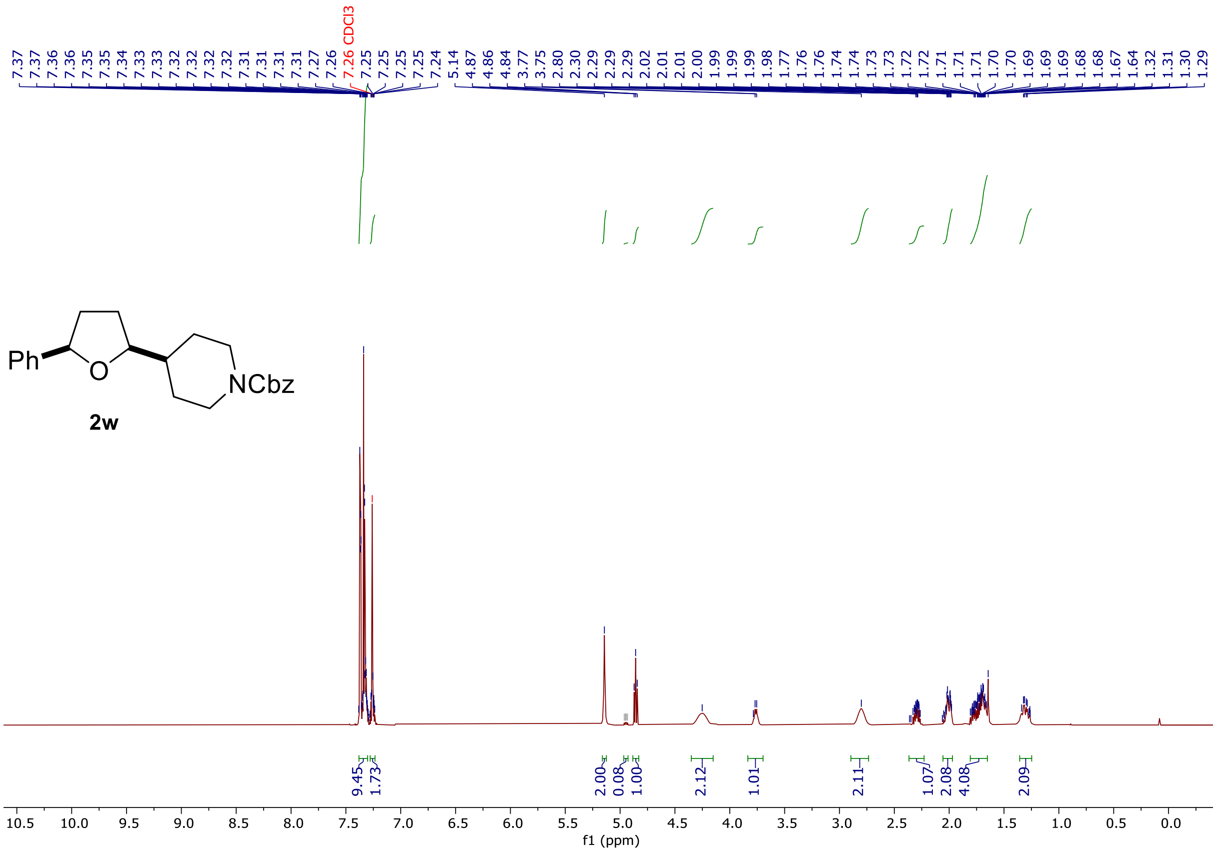

$^{13}C\{^{1}H\}$ NMR (126 MHz, $CDCl_3$) of **2w**

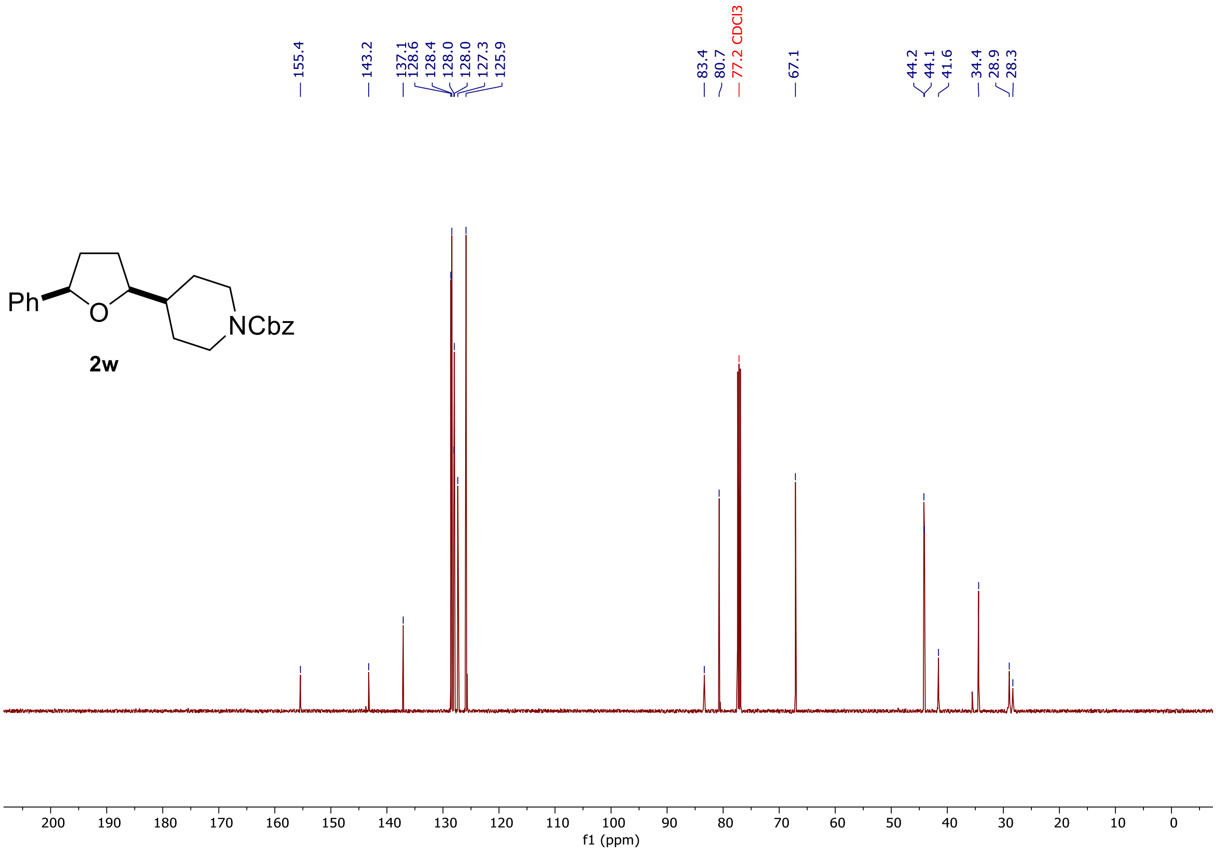

^{1}H NMR (500 MHz, $CDCl_3$) of **2x**

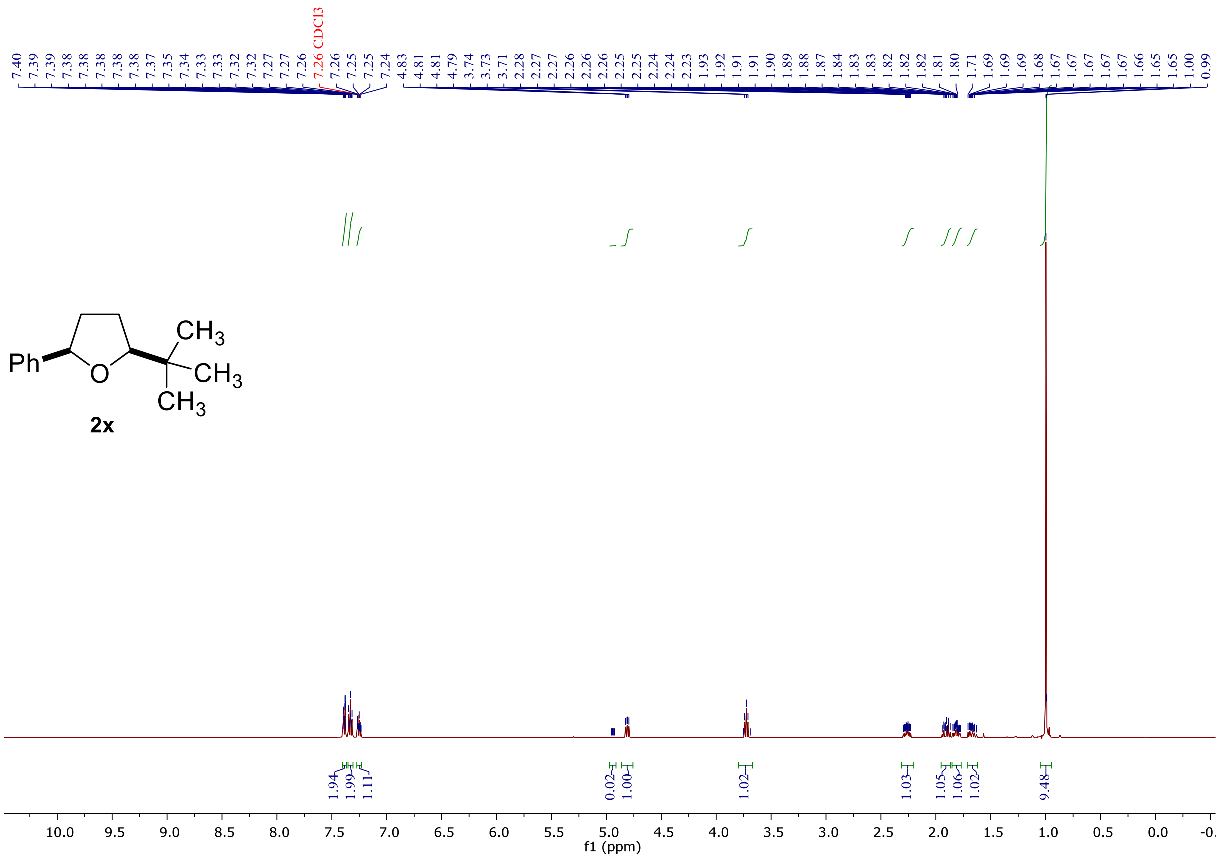

$^{13}C\{^1H\}$ NMR (126 MHz, $CDCl_3$) of **2x**

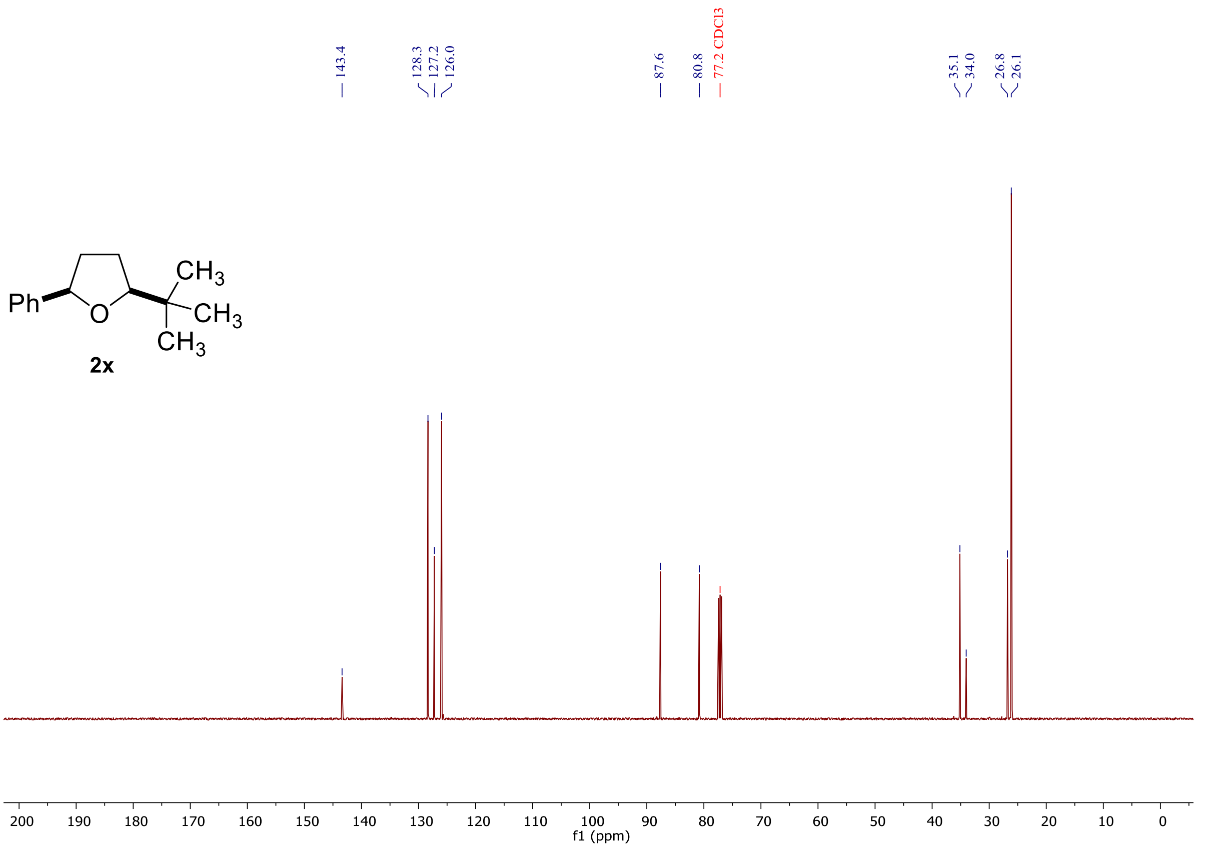

^{1}H NMR (500 MHz, $CDCl_3$) of **2y**

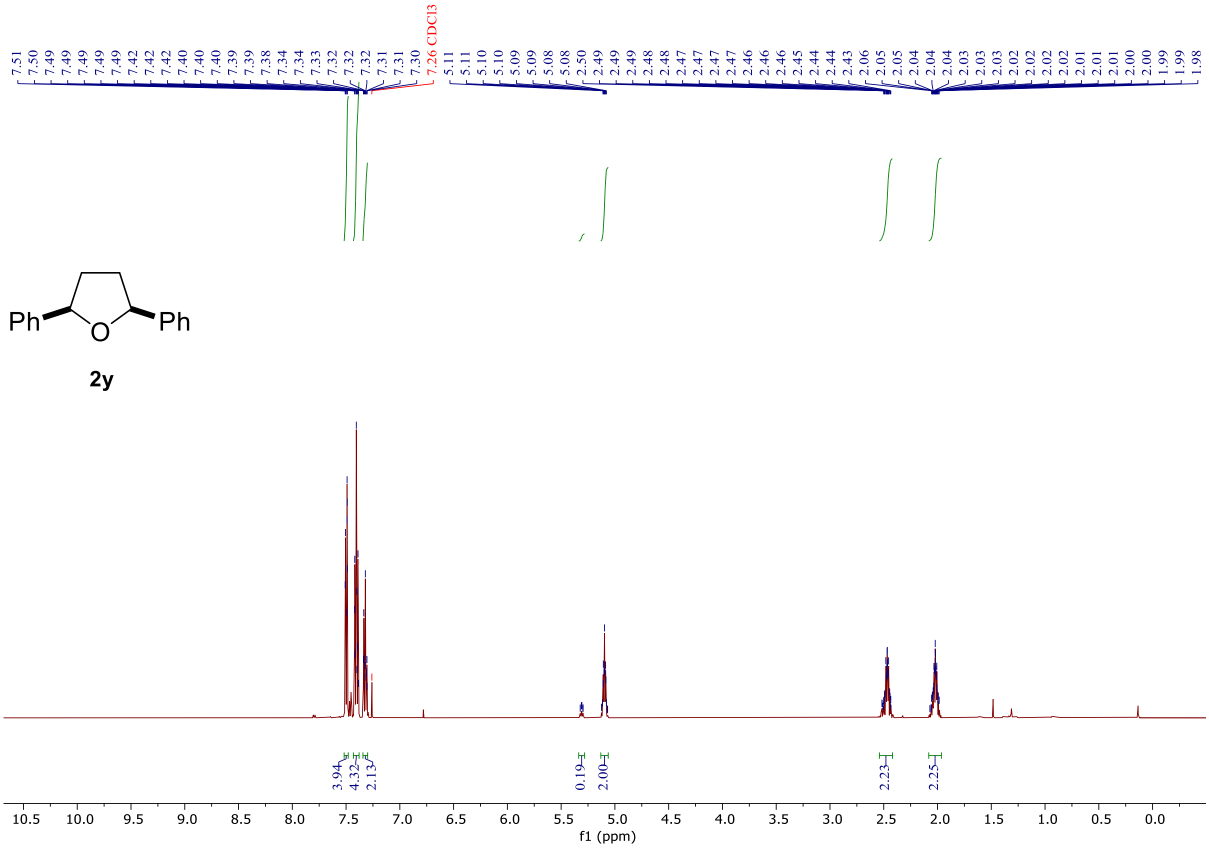

$^{13}C\{^{1}H\}$ NMR (126 MHz, $CDCl_3$) of **2y**

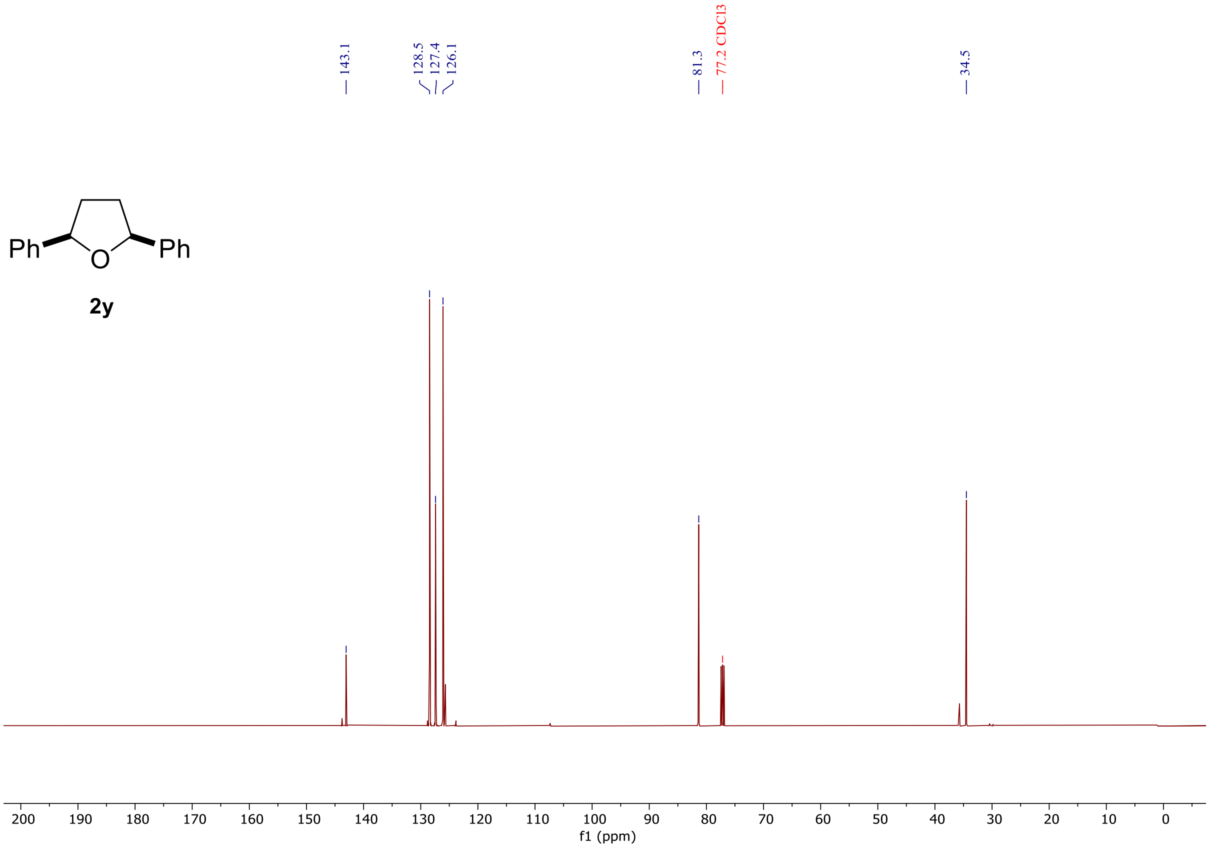

^{1}H NMR (500 MHz, $CDCl_3$) of **2z**

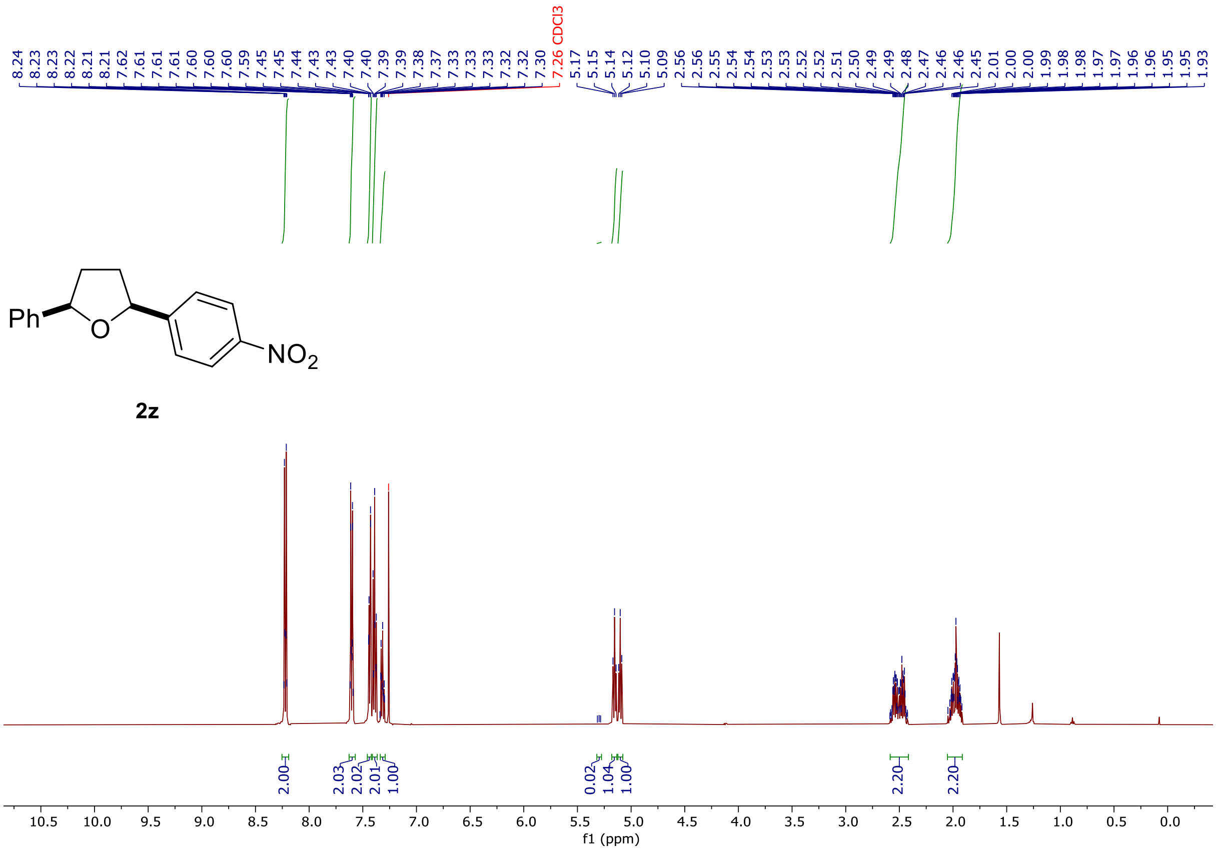

^{13}C{^{1}H} NMR (126 MHz, $CDCl_3$) of **2z**

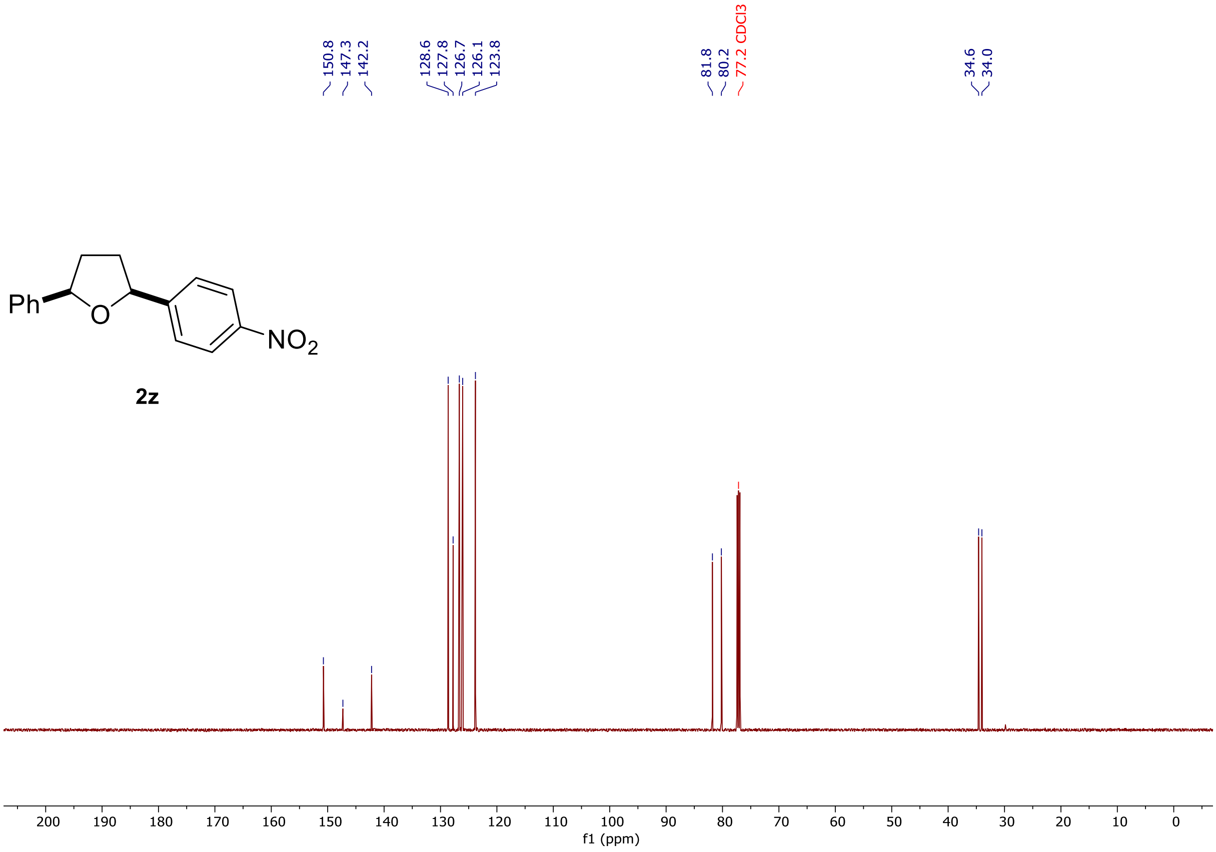

^{1}H NMR (500 MHz, $CDCl_3$) of **2aa**

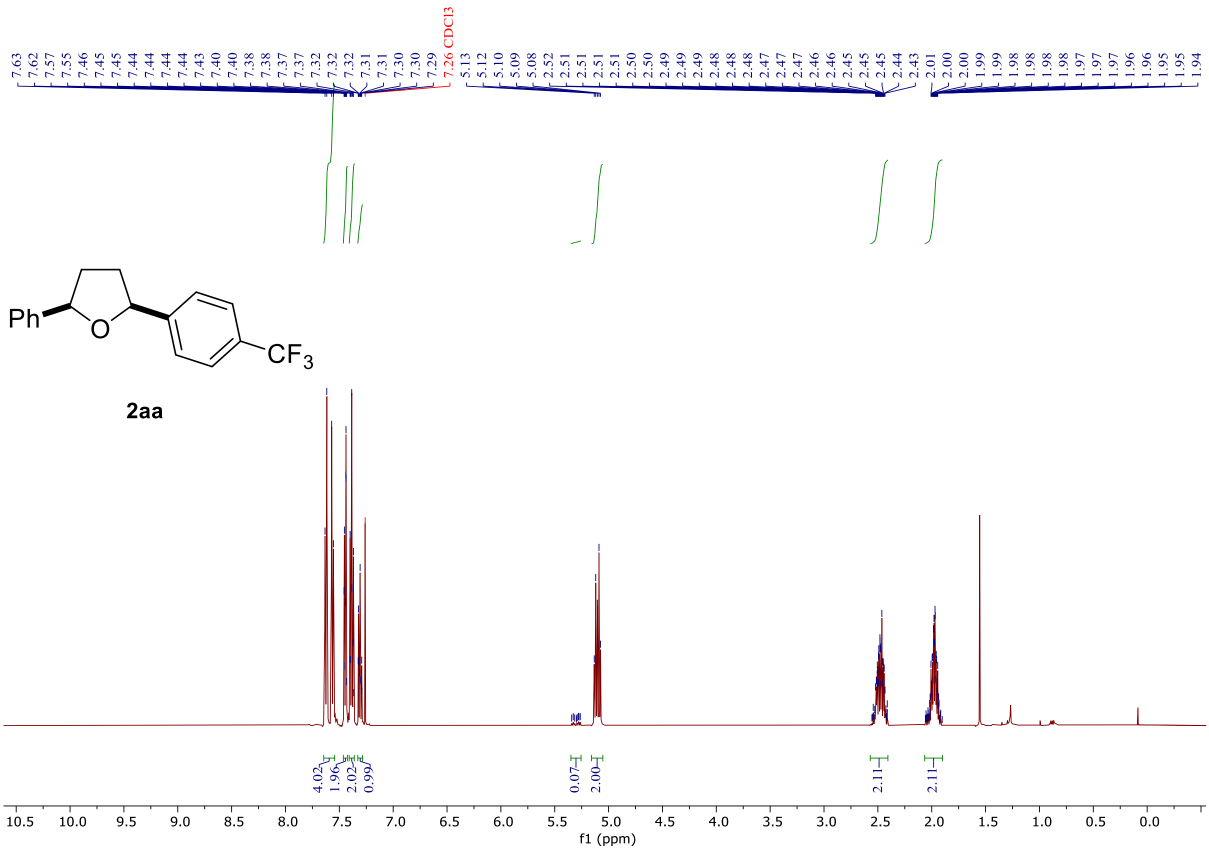

$^{13}C\{^{1}H\}$ NMR (126 MHz, $CDCl_3$) of **2aa**

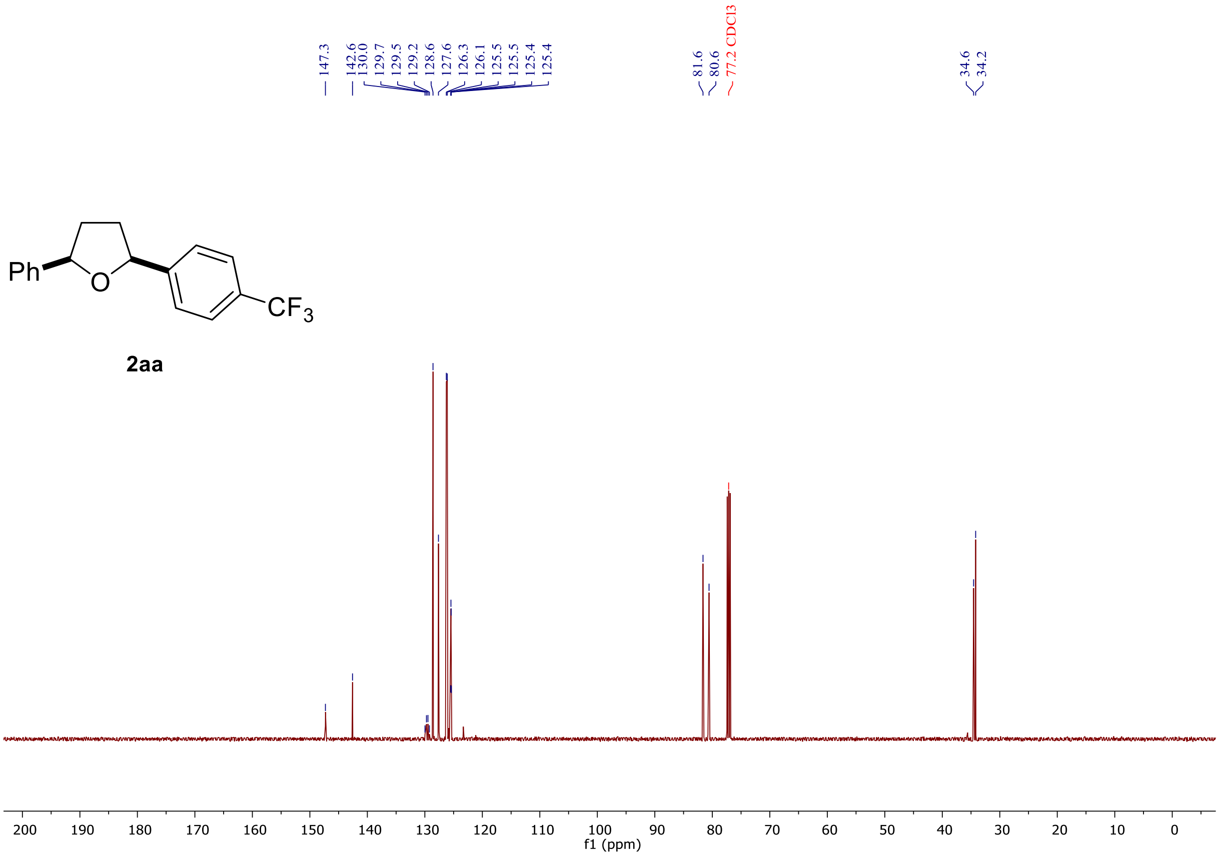

^{19}F NMR (471 MHz, $CDCl_3$) of **2aa**

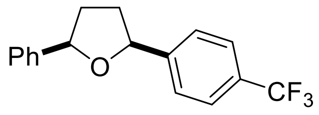

2aa

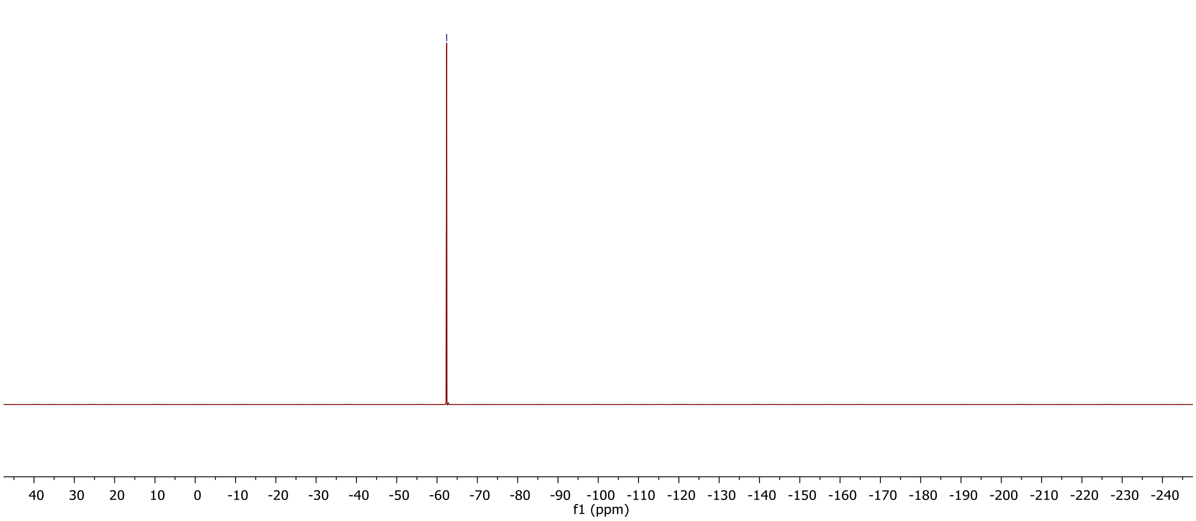

^{1}H NMR (500 MHz, $CDCl_3$) of **2ab**

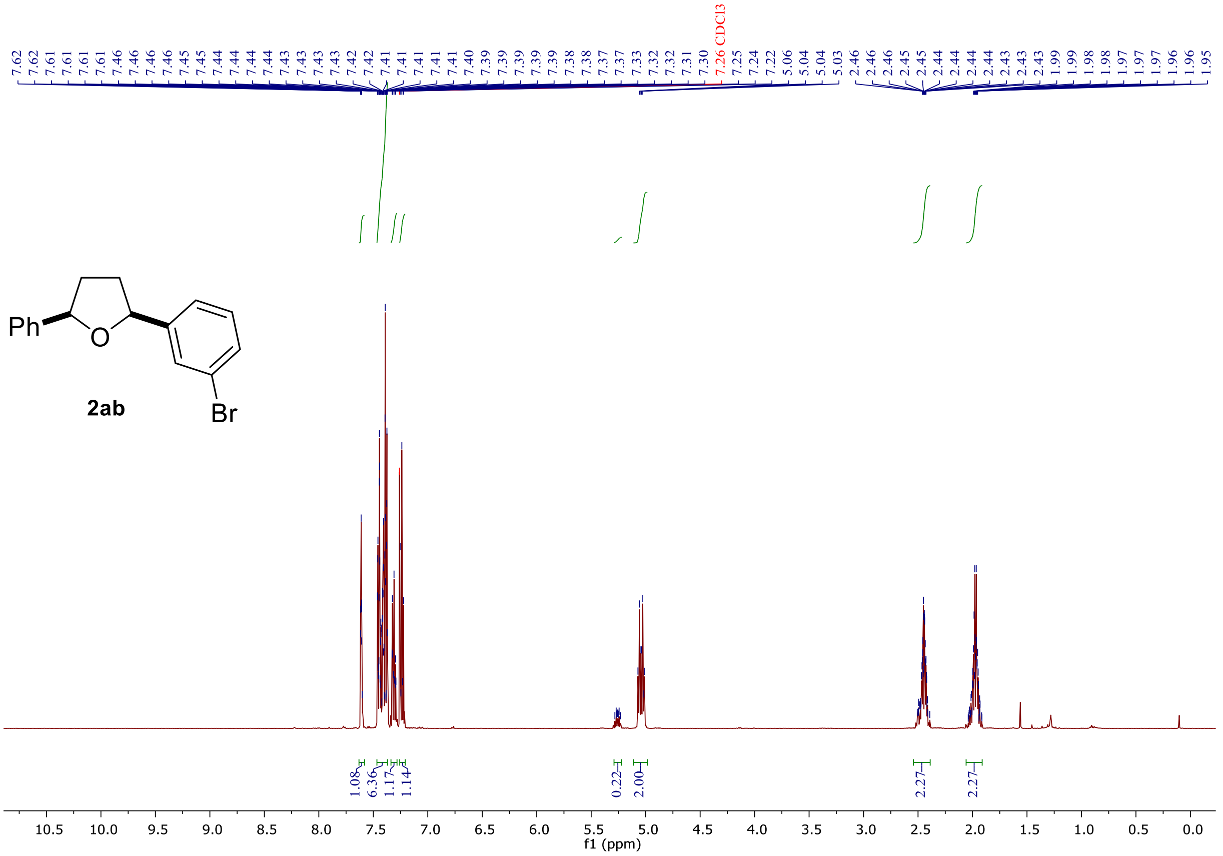

$^{13}C\{^{1}H\}$ NMR (126 MHz, $CDCl_3$) of **2ab**

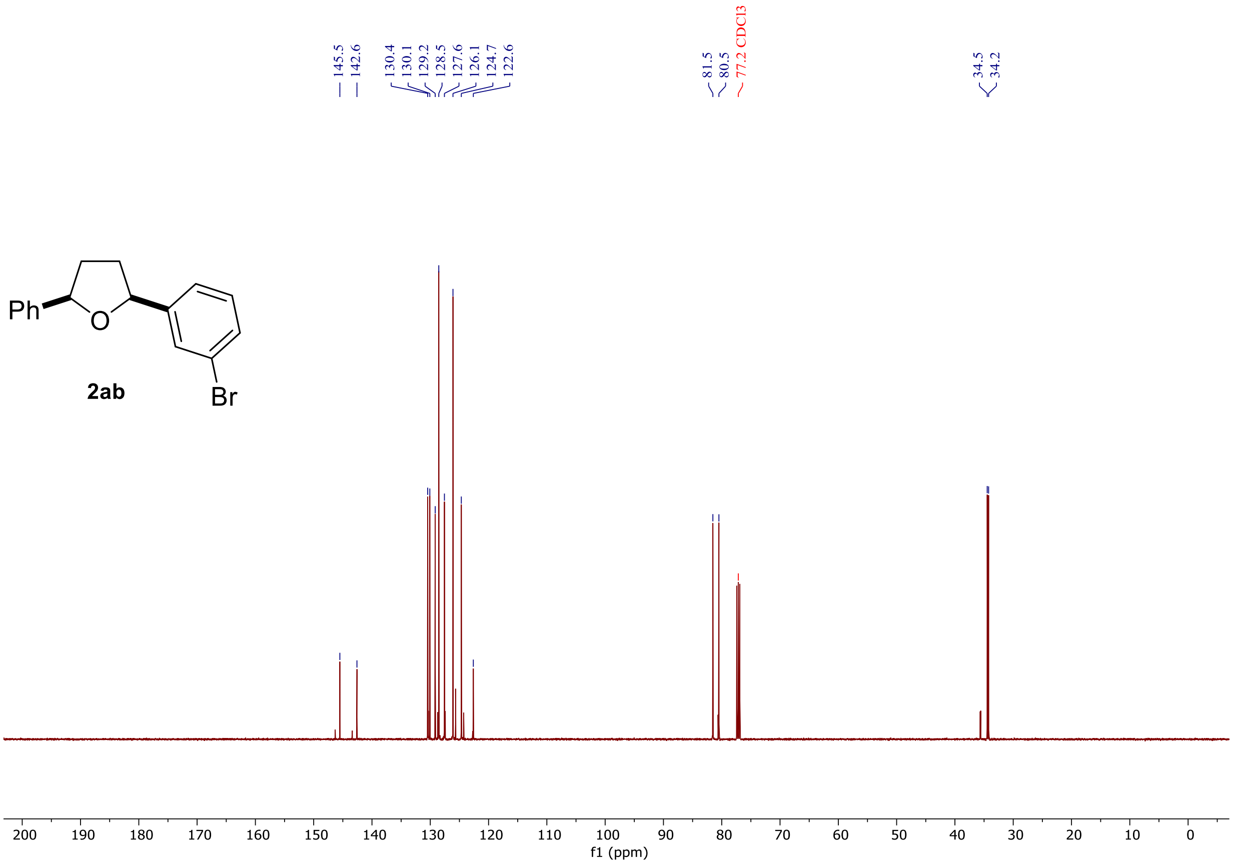

^{1}H NMR (500 MHz, $CDCl_3$) of **2ac**

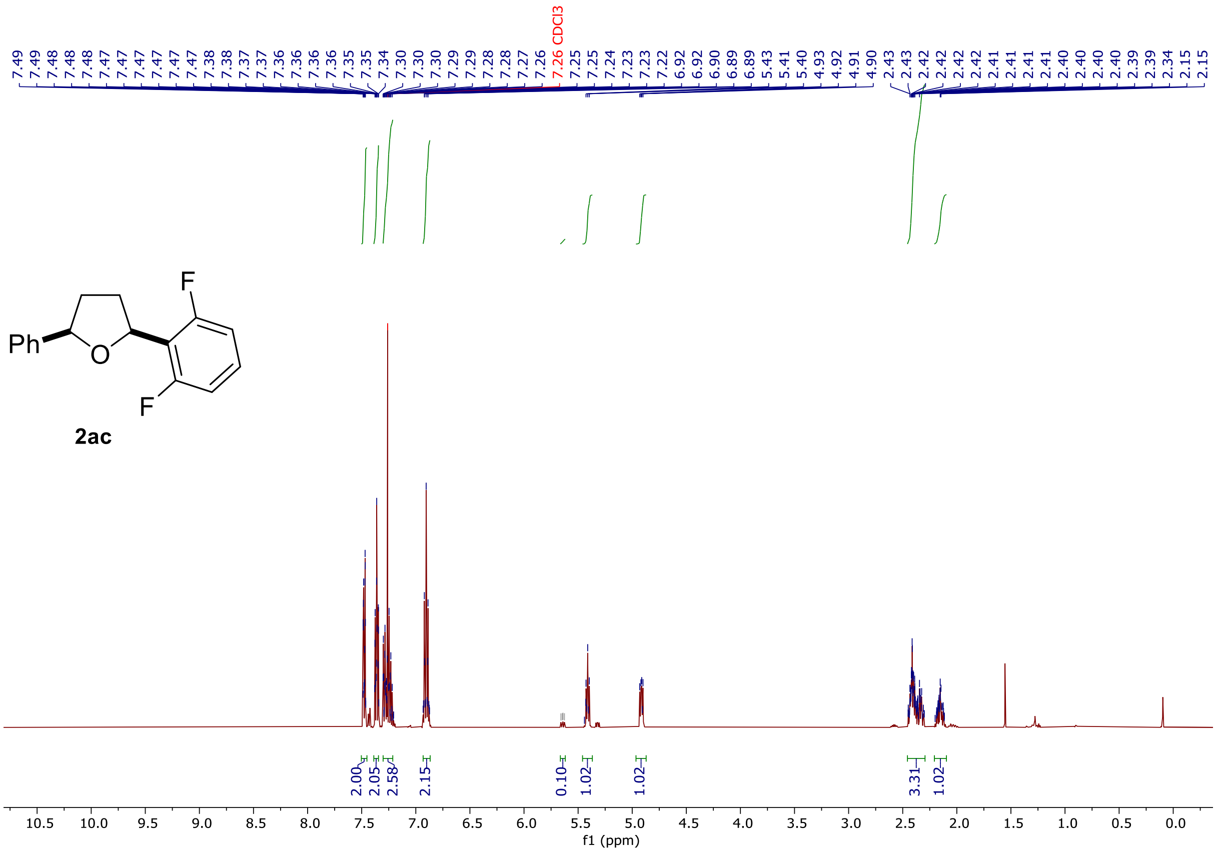

$^{13}C\{^{1}H\}$ NMR (126 MHz, $CDCl_3$) of **2ac**

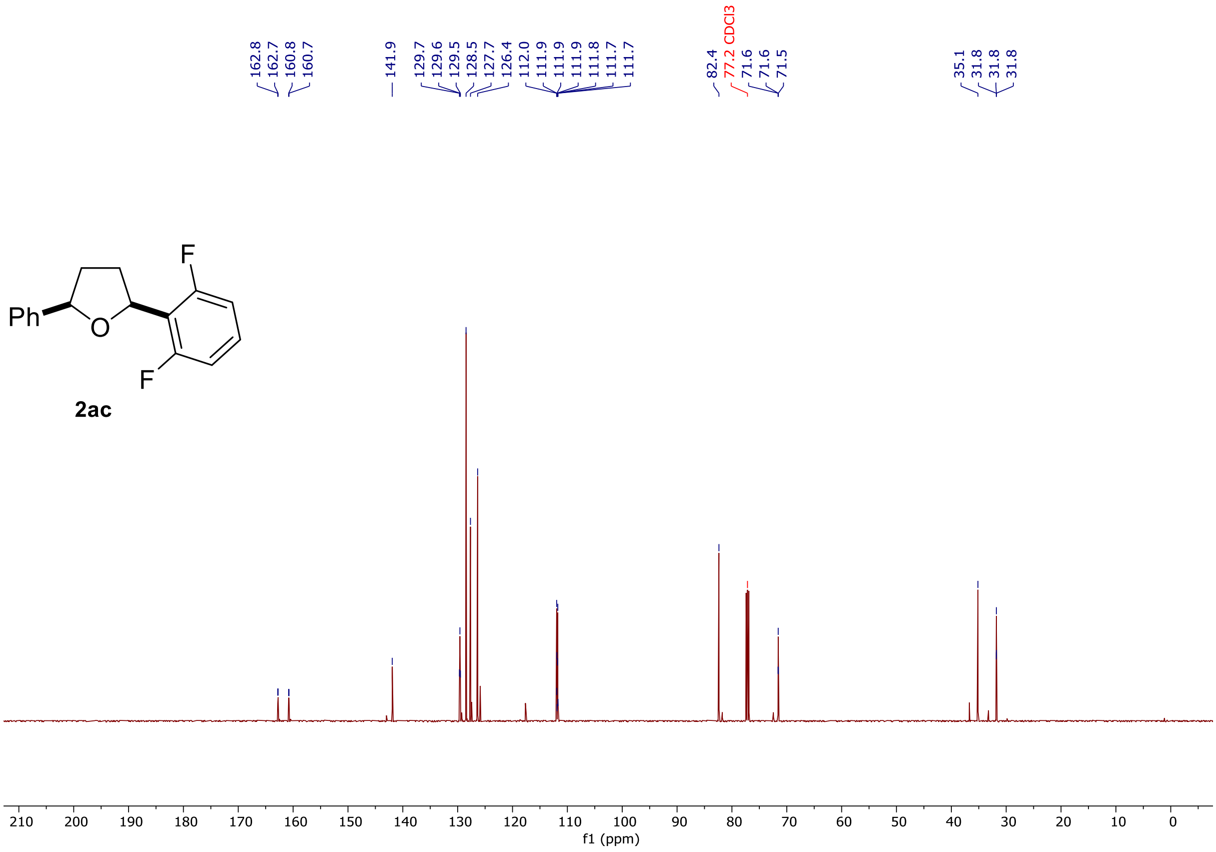

^{19}F NMR (471 MHz, $CDCl_3$) of **2ac**

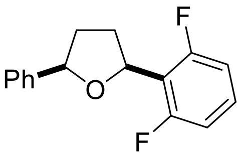

2ac

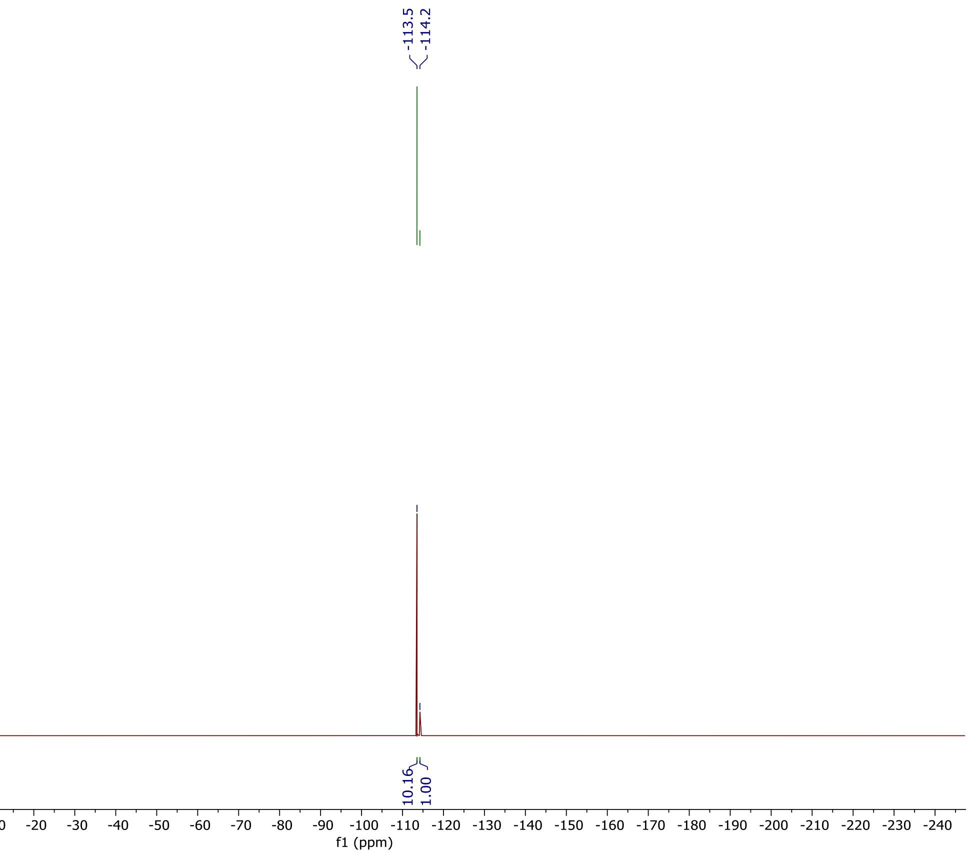

^{1}H NMR (500 MHz, $CDCl_3$) of **2ad**

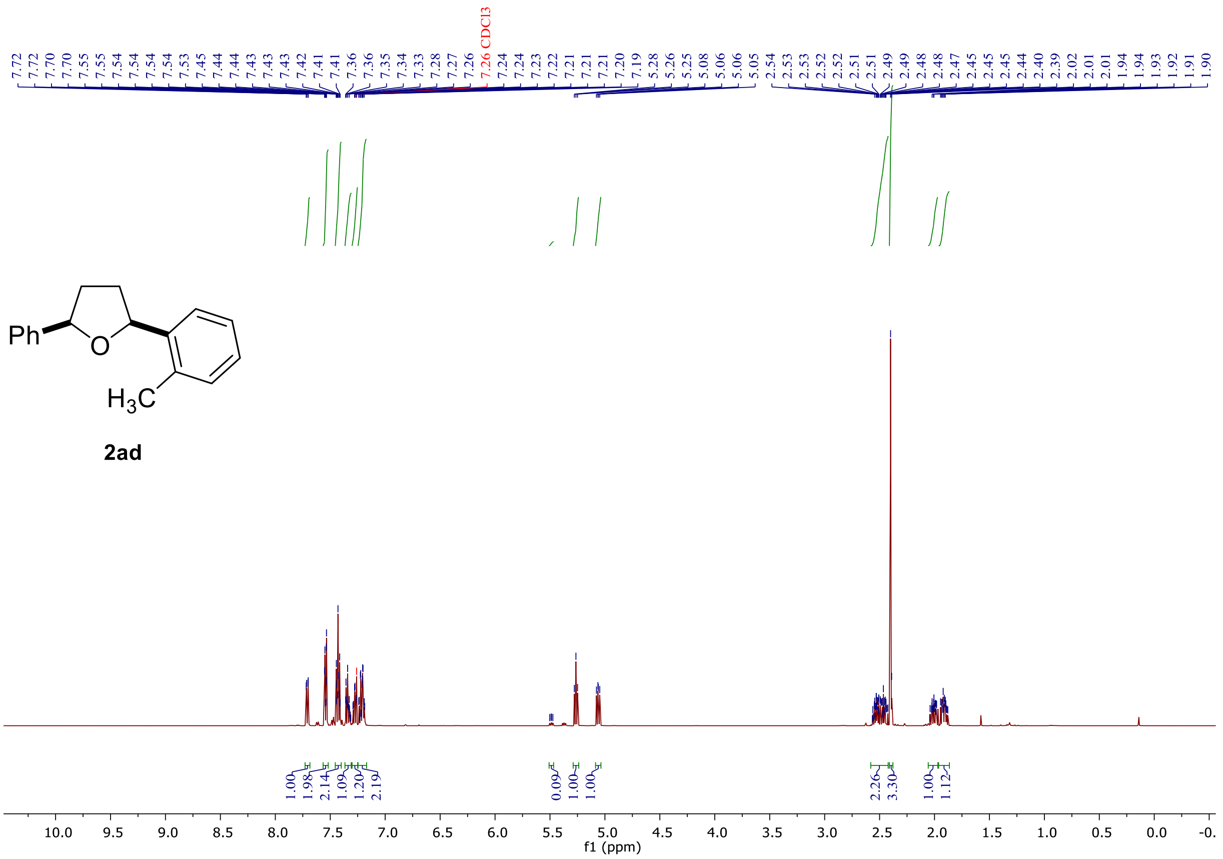

^{13}C{^{1}H} NMR (126 MHz, $CDCl_3$) of **2ad**

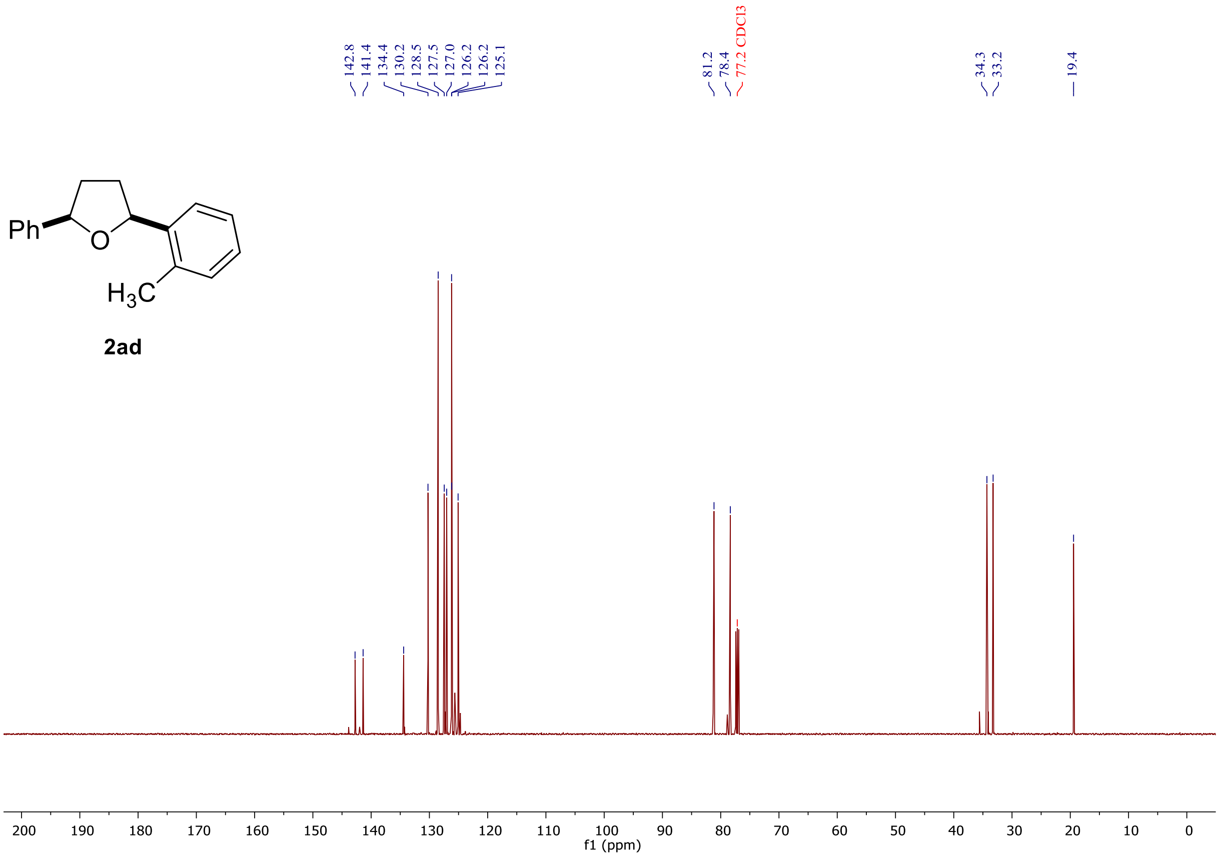

^{1}H NMR (500 MHz, $CDCl_3$) of **2ae**

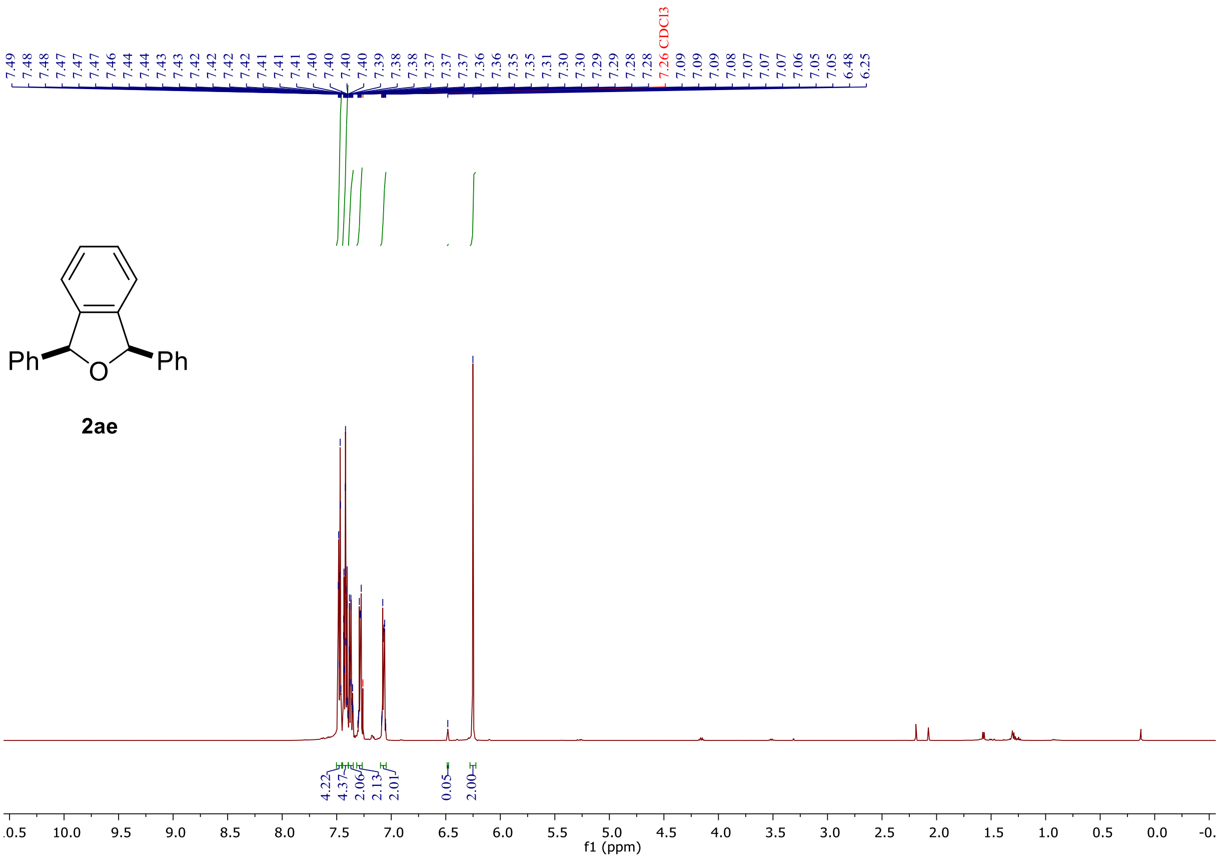

^{13}C{^{1}H} NMR (126 MHz, $CDCl_3$) of **2ae**

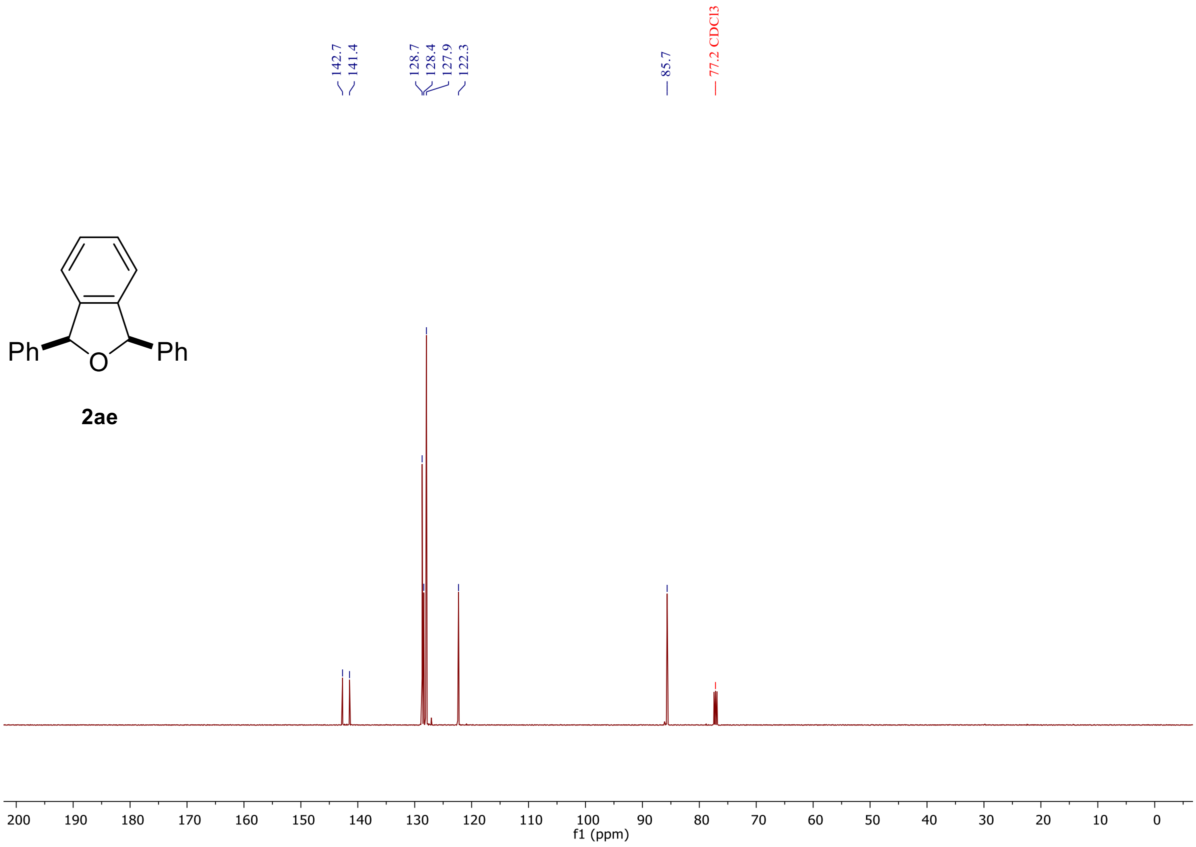

^{1}H NMR (500 MHz, $CDCl_3$) of **2af**

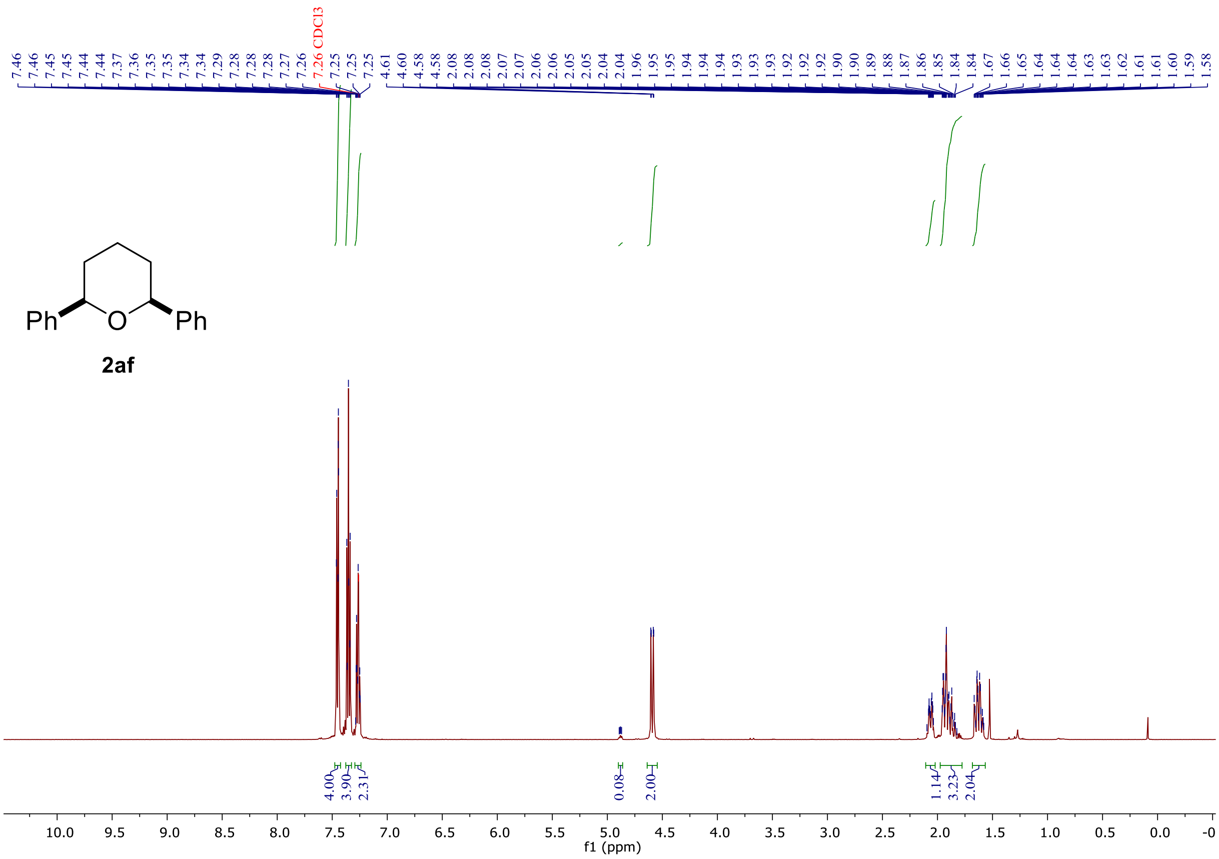

^{13}C{^{1}H} NMR (126 MHz, $CDCl_3$) of **2af**

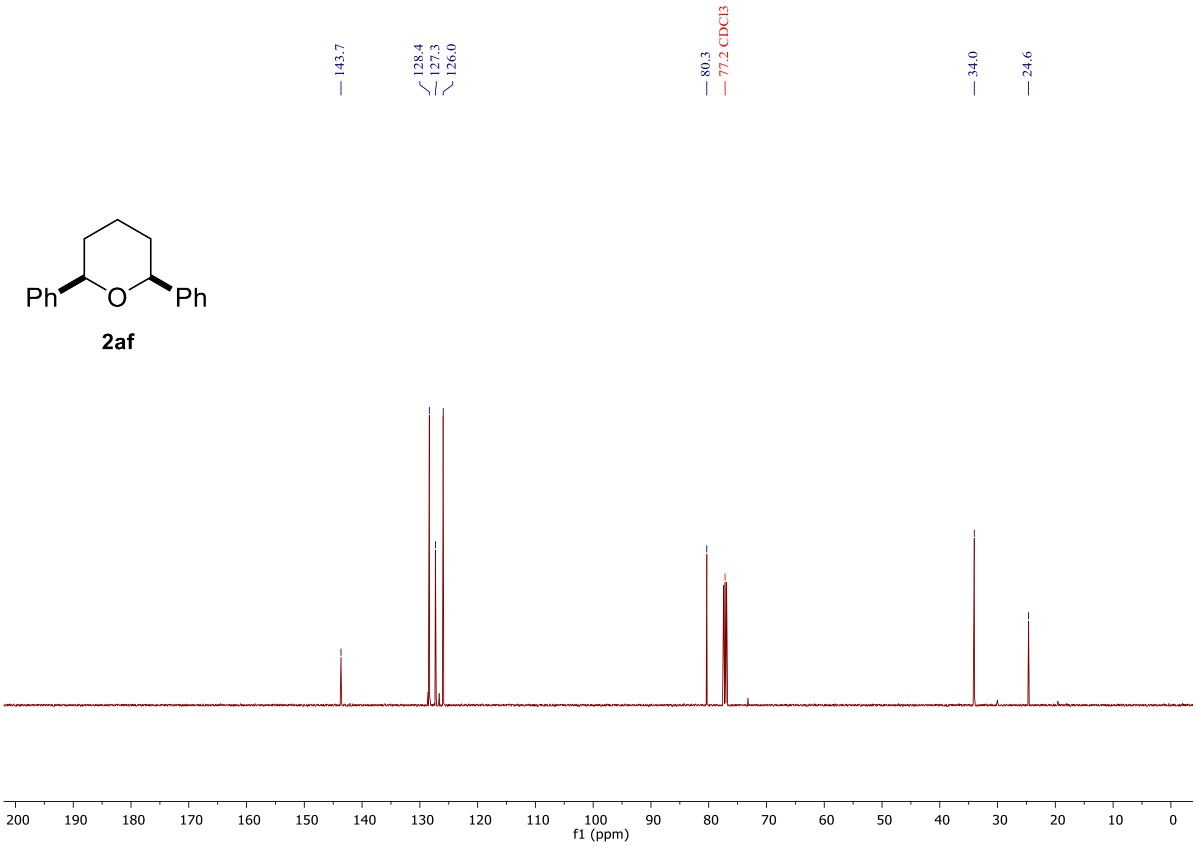

^{1}H NMR (500 MHz, $CDCl_3$) of **2ag**

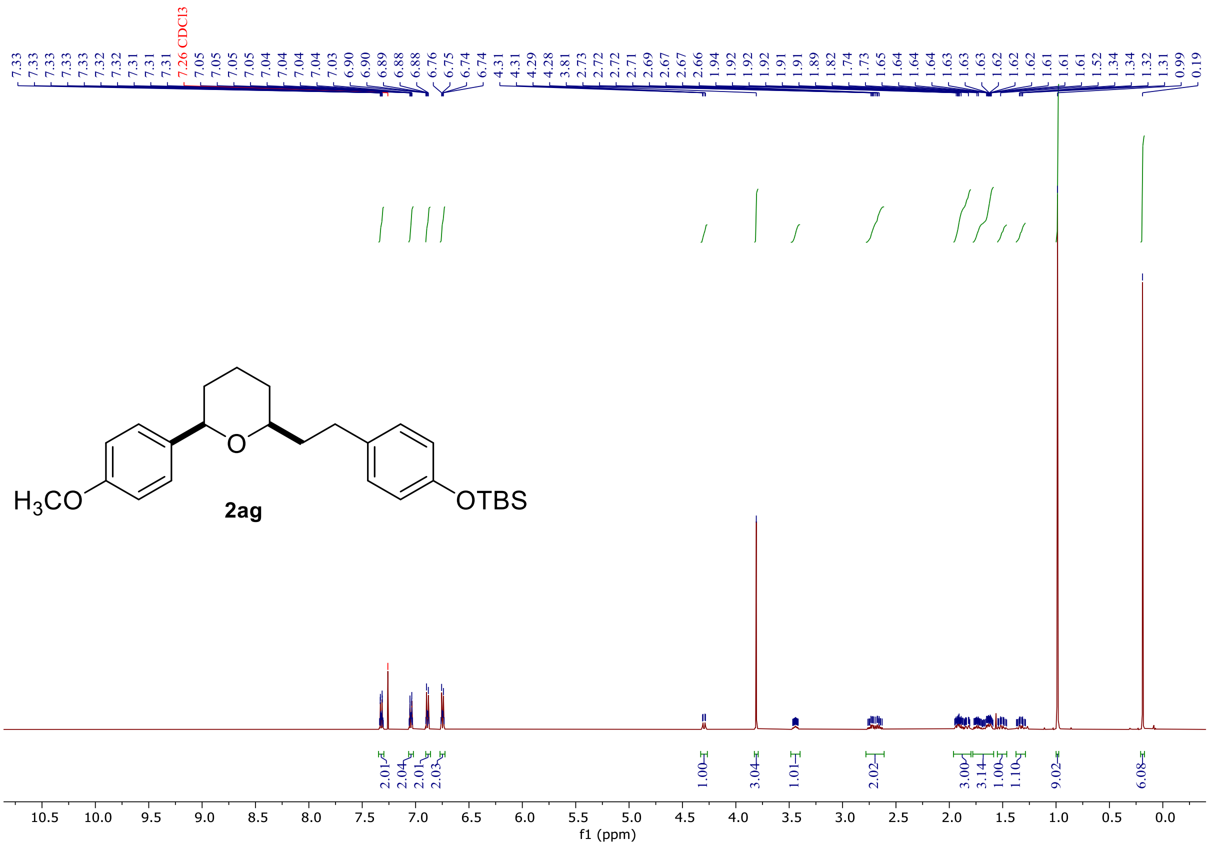

^{13}C{^{1}H} NMR (126 MHz, $CDCl_3$) of **2ag**

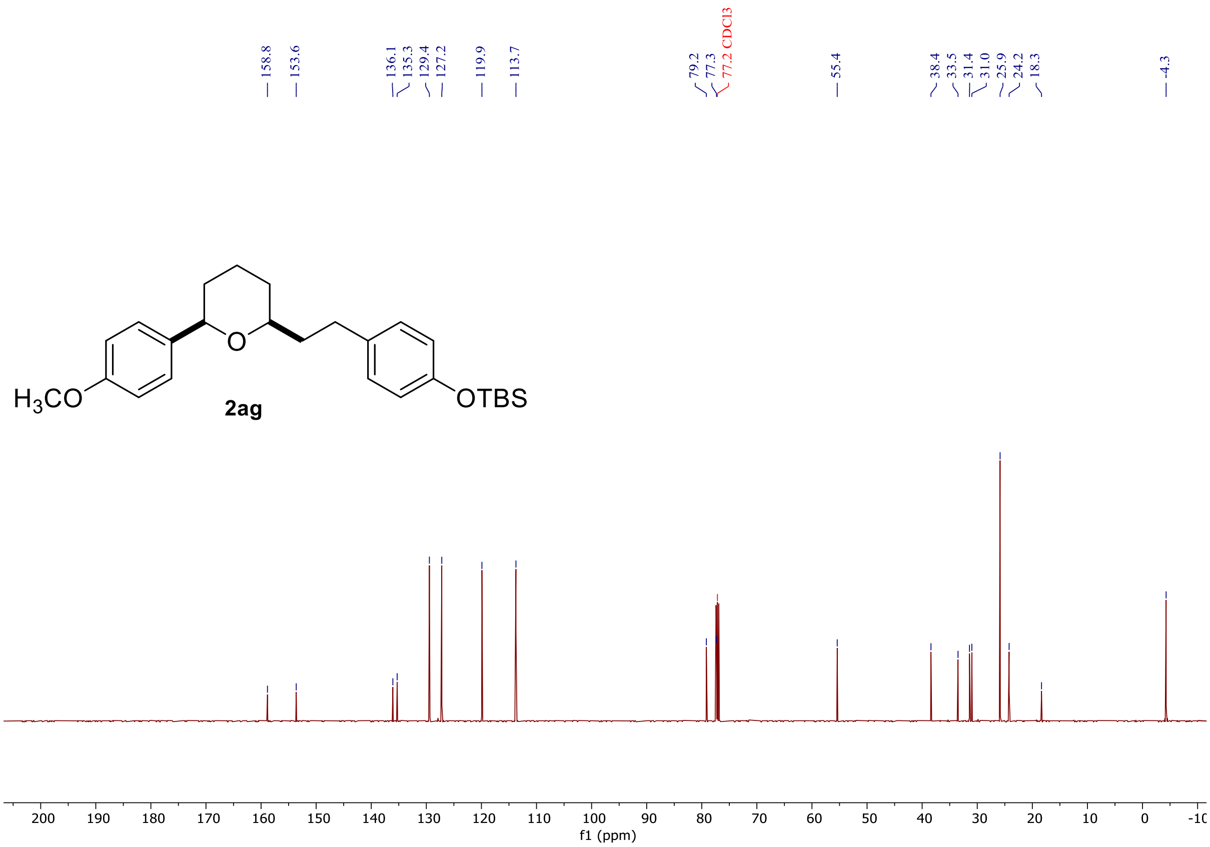

^{1}H NMR (500 MHz, $CDCl_3$) of **2ag′**

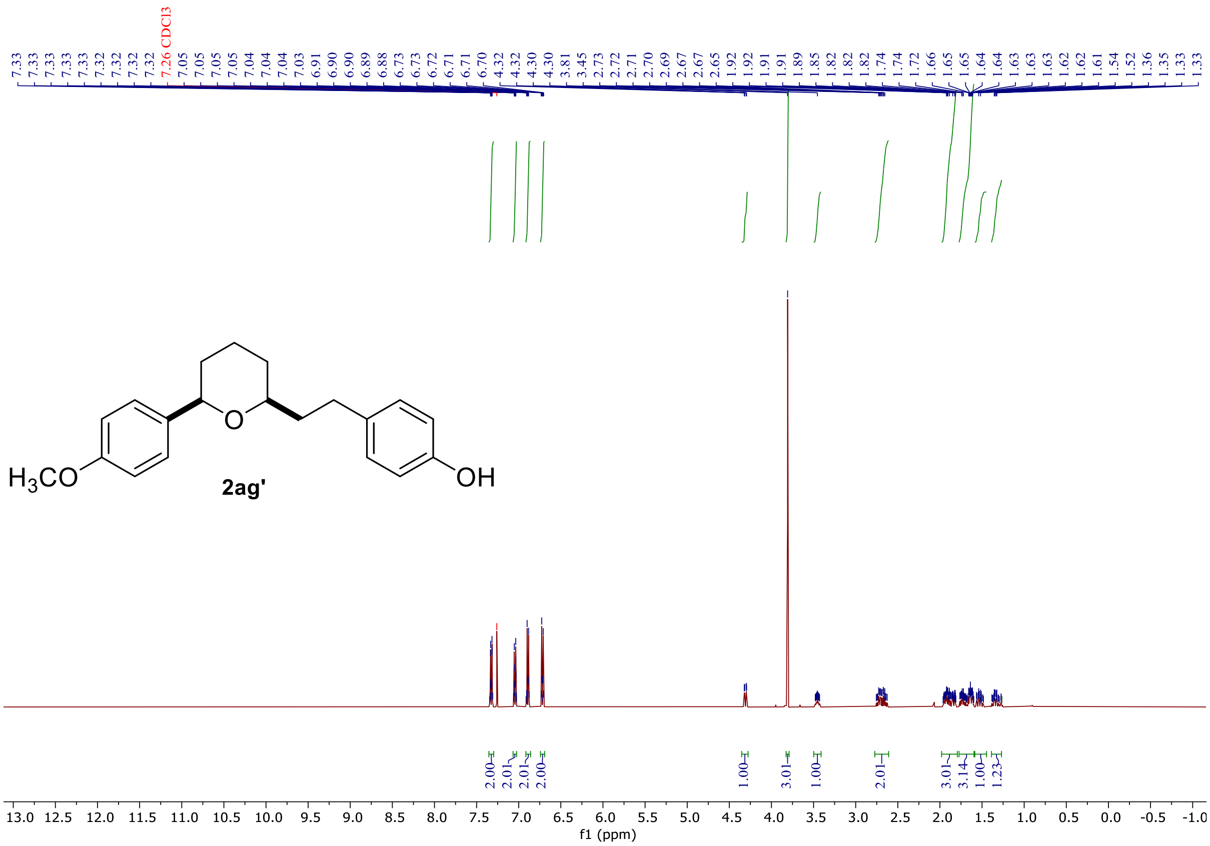

^{13}C{^{1}H} NMR (126 MHz, $CDCl_3$) of **2ag′**

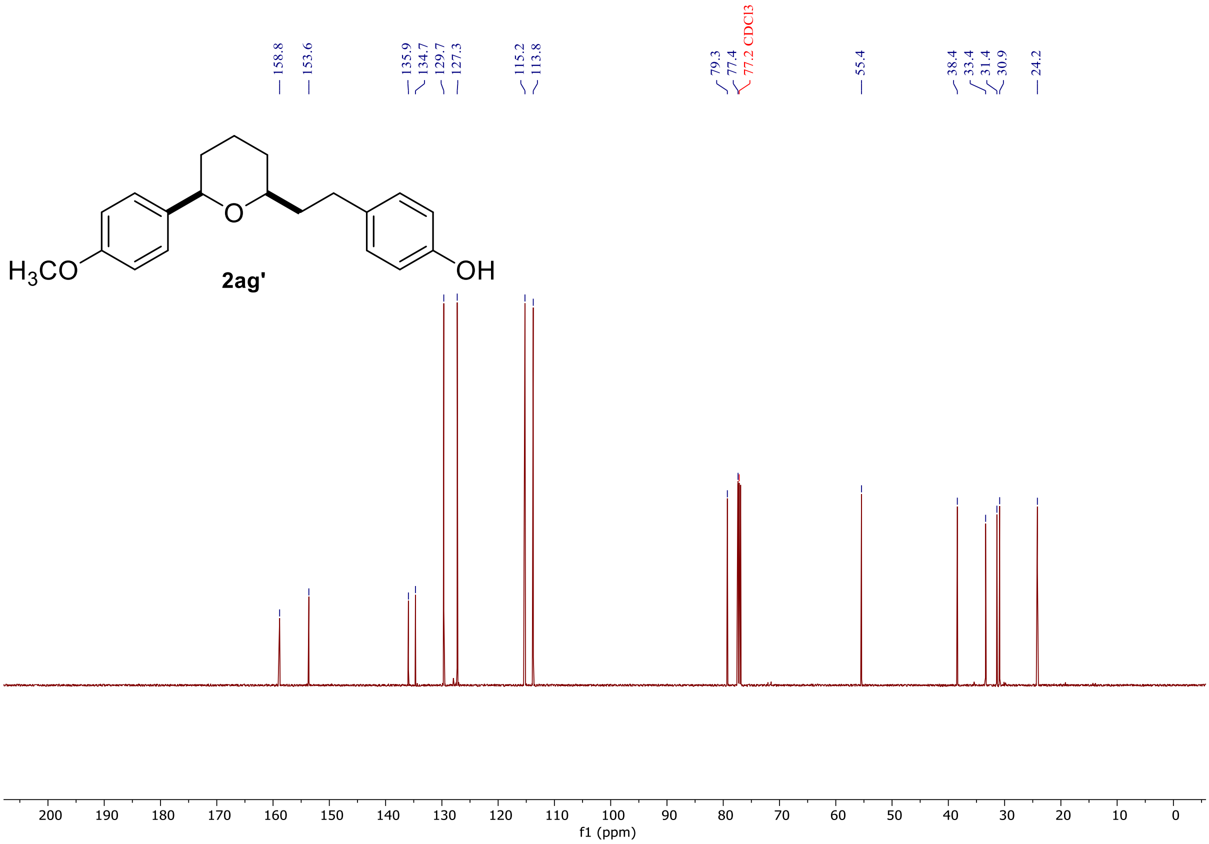

^1H NMR (500 MHz, $CDCl_3$) of **3ai** (crude)

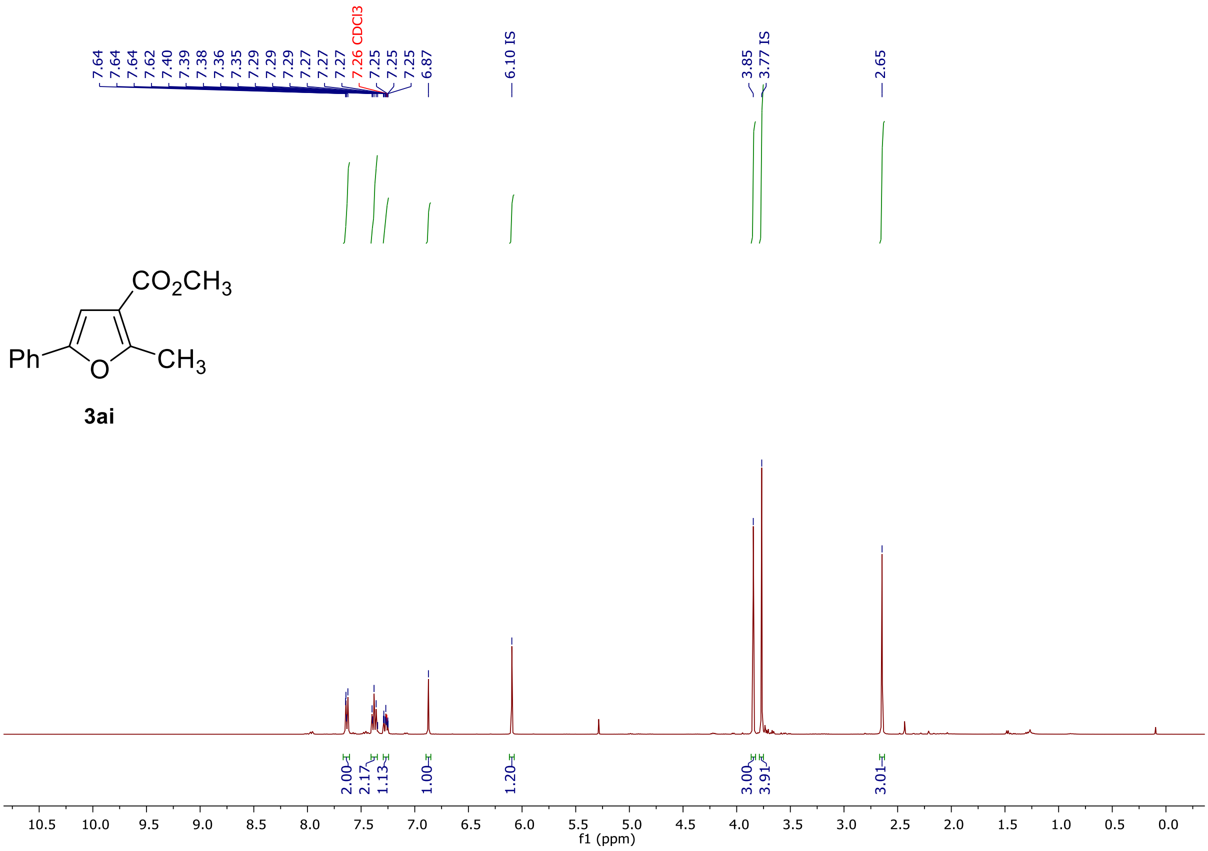

^{1}H NMR (500 MHz, $CDCl_3$) of **2aj**

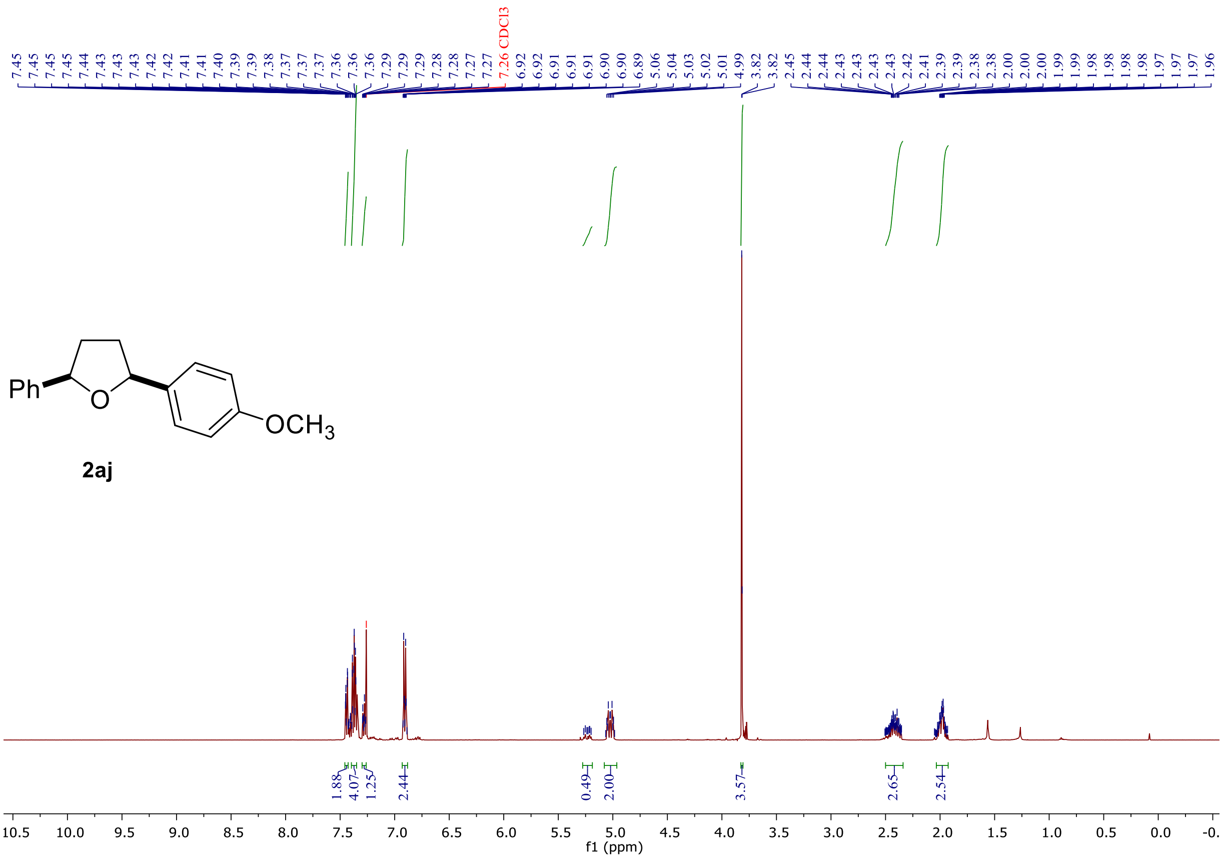

$^{13}C\{^{1}H\}$ NMR (126 MHz, $CDCl_3$) of **2aj**

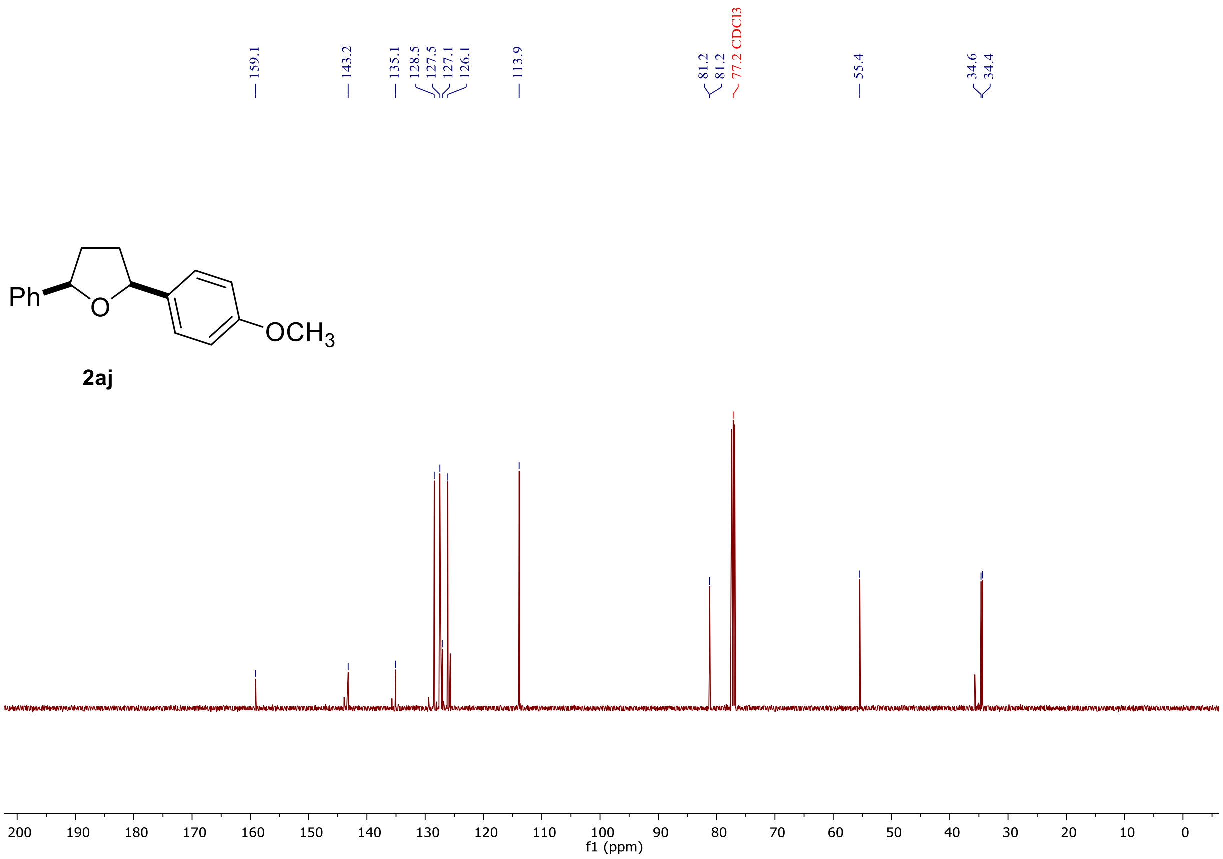

^{1}H NMR (500 MHz, $CDCl_3$) of **2ak** (crude)

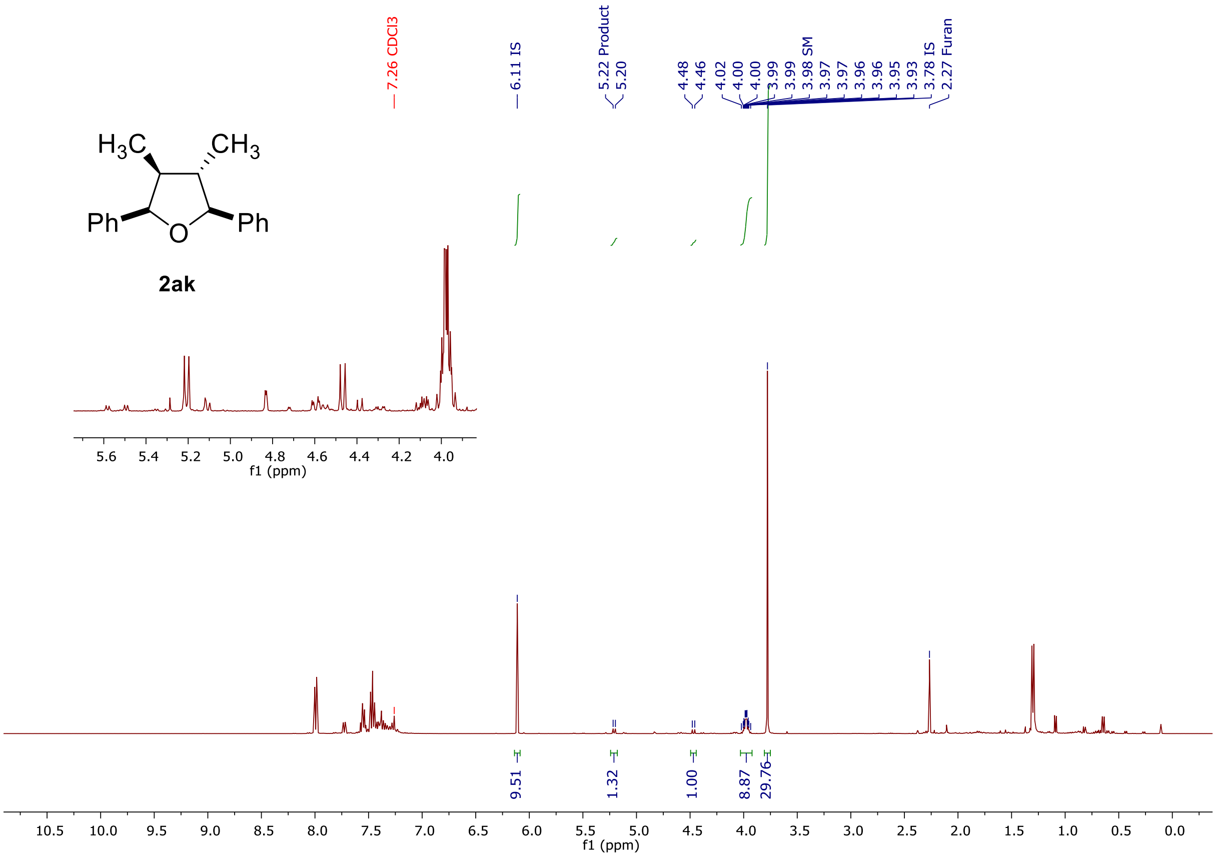

^{1}H NMR (500 MHz, $CDCl_3$) of **2al**

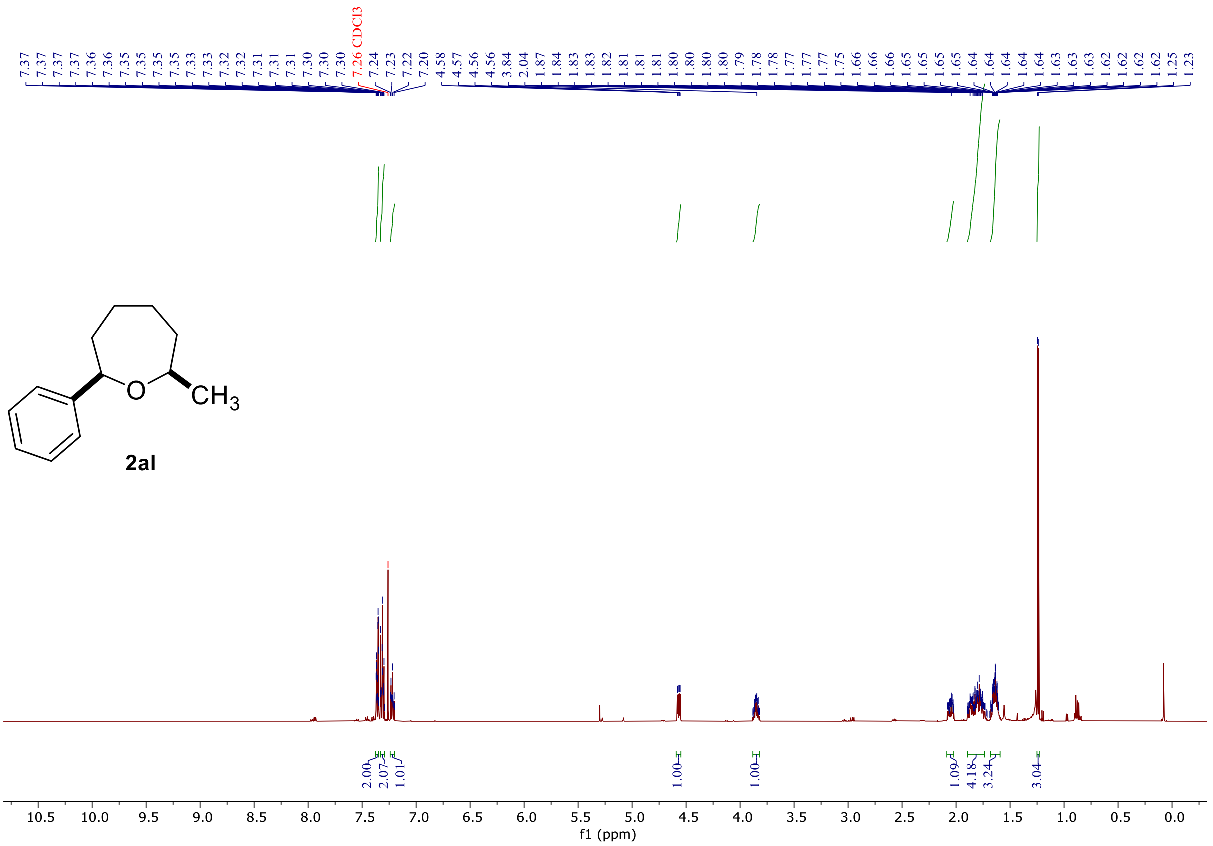

$^{13}C\{^{1}H\}$ NMR (126 MHz, $CDCl_3$) of **2al**

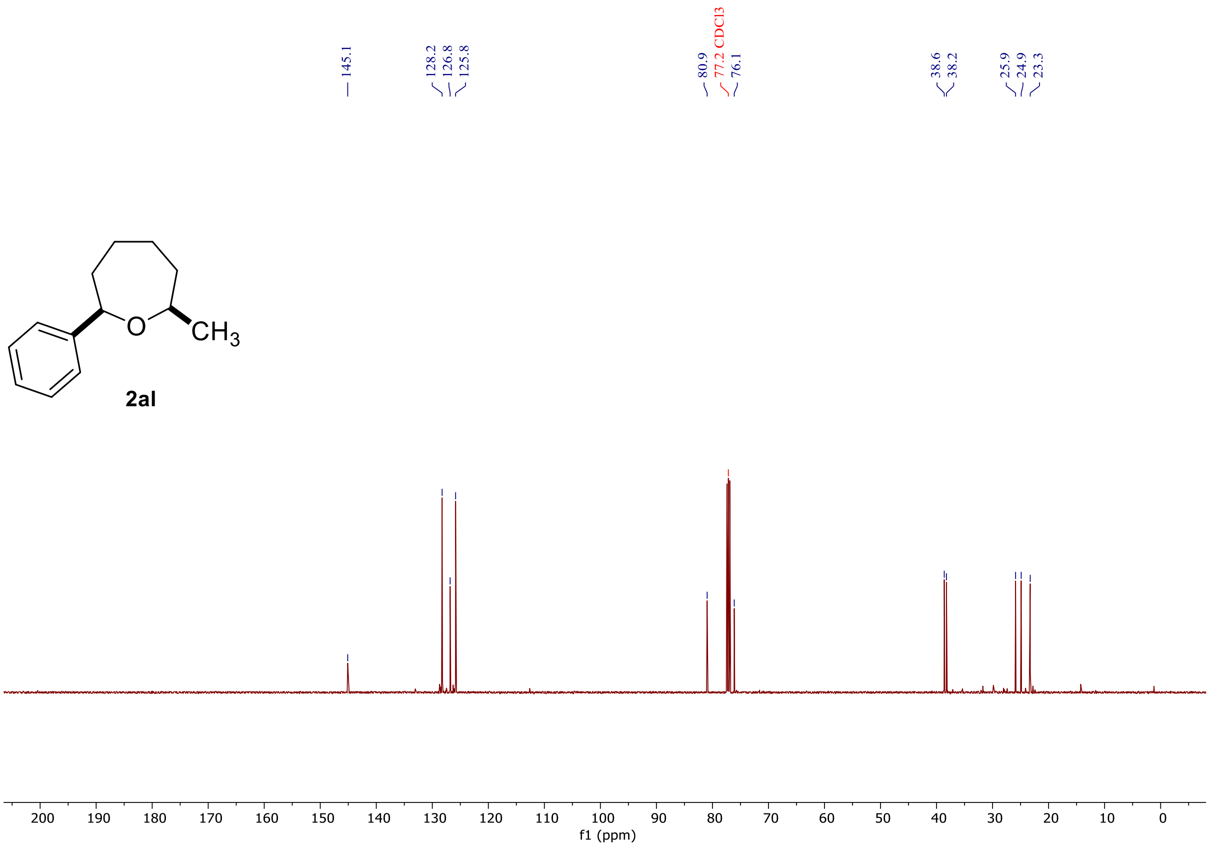

^{1}H NMR (500 MHz, $CDCl_3$) of **2am**

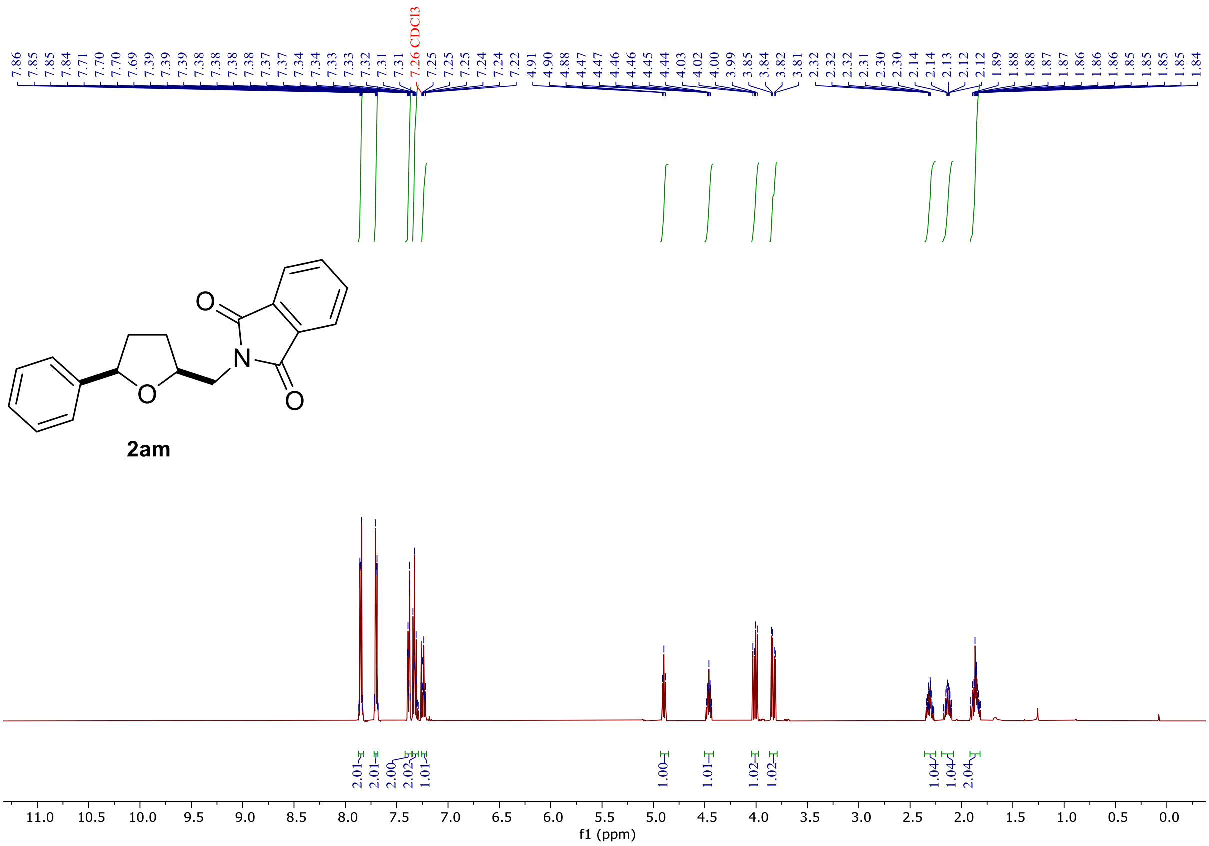

^{13}C{^{1}H} NMR (126 MHz, $CDCl_3$) of **2am**

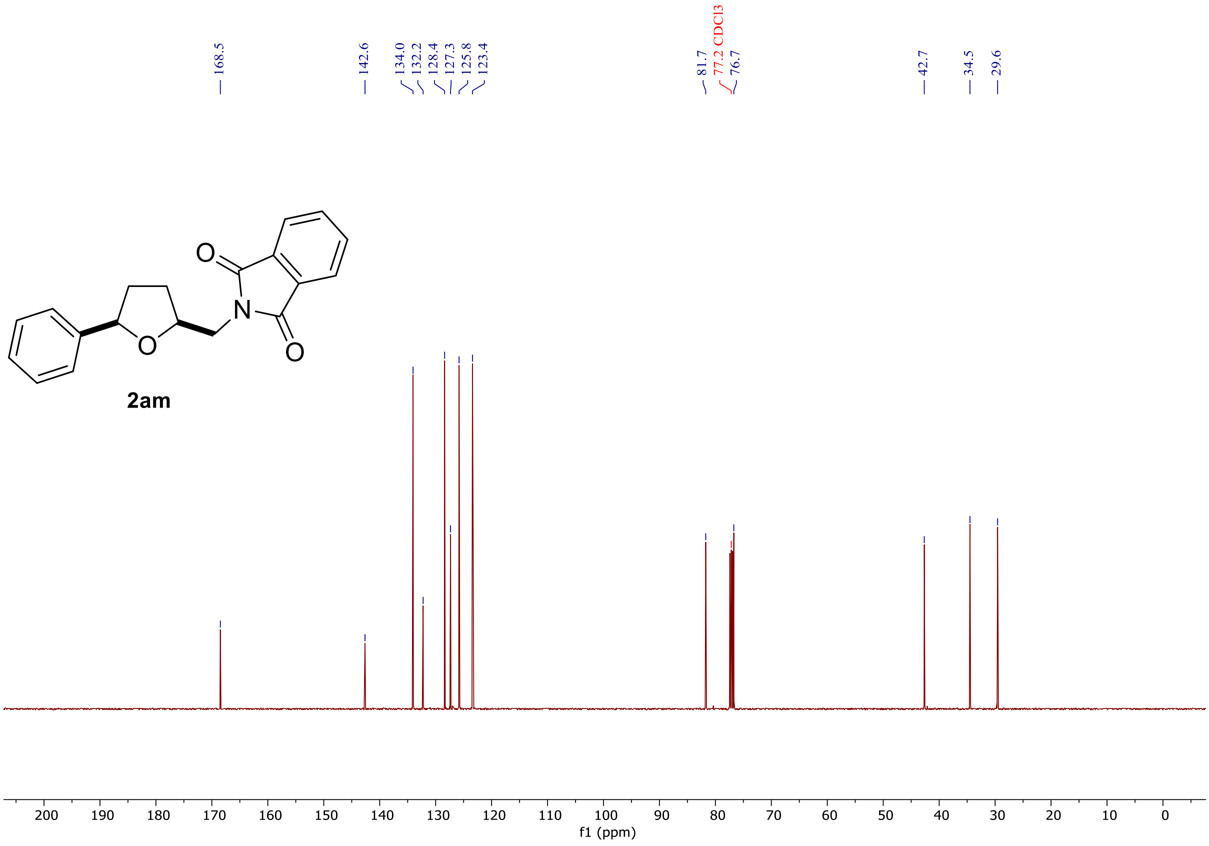

^{1}H NMR (400 MHz, $CDCl_3$) of **2am′**

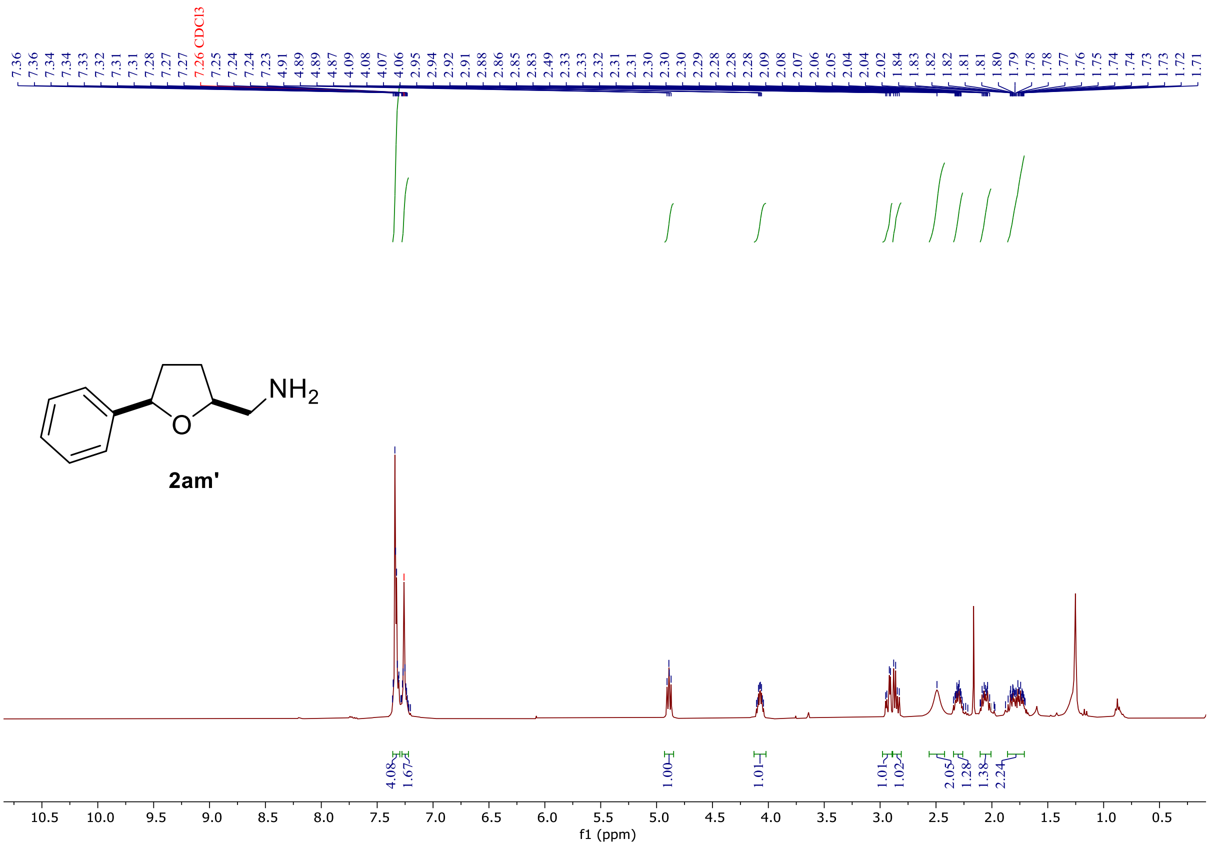

^{1}H NMR (500 MHz, $CDCl_3$) of **2an**

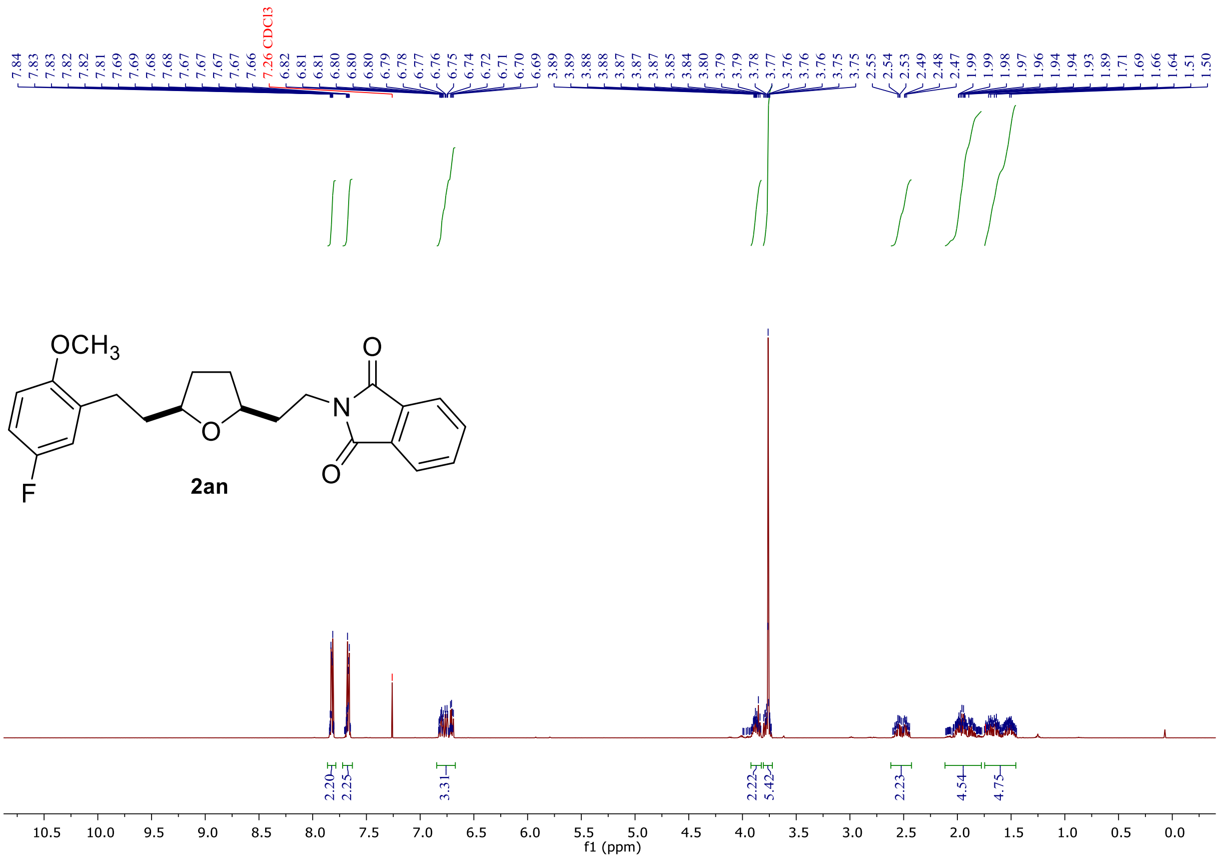

$^{13}C\{^{1}H\}$ NMR (126 MHz, $CDCl_3$) of **2an**

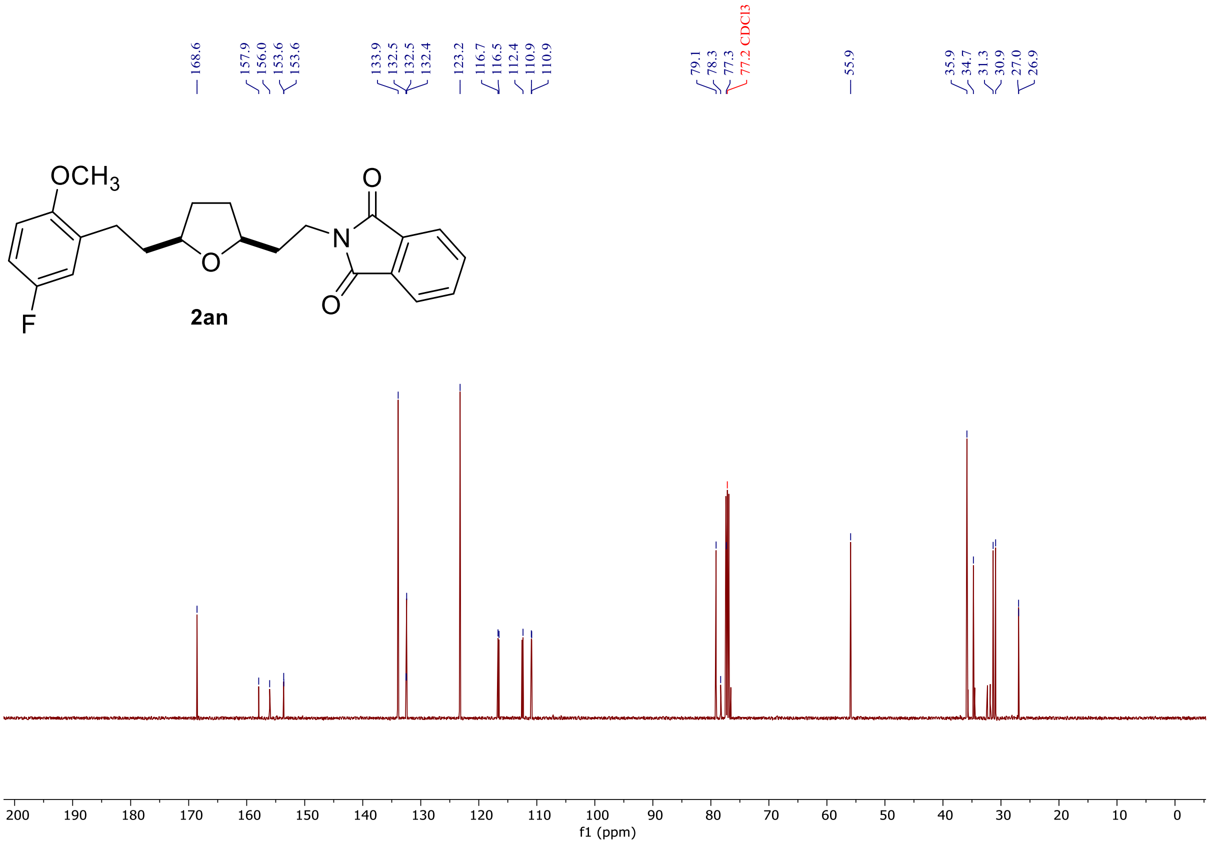

^{19}F NMR (471 MHz, $CDCl_3$) of **2an**

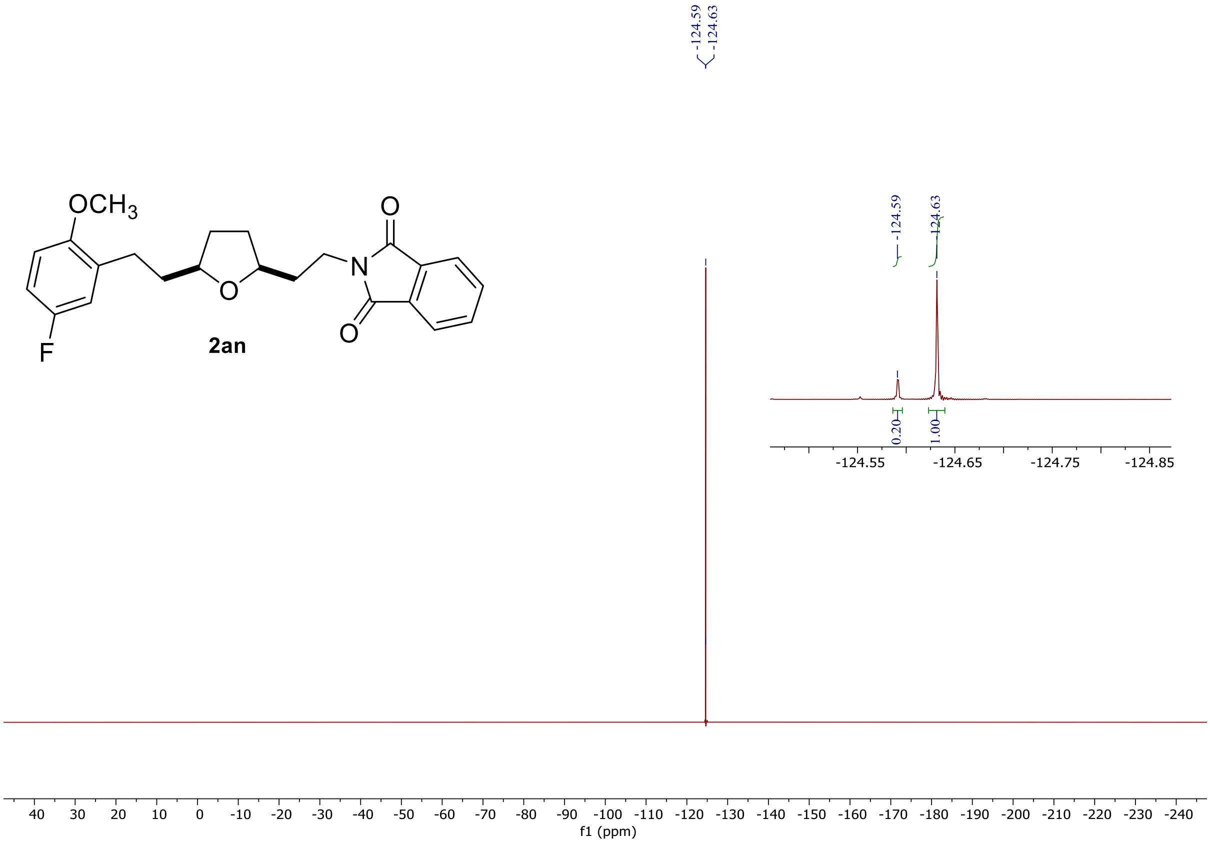

^{1}H NMR (400 MHz, $CDCl_3$) of **2an´**

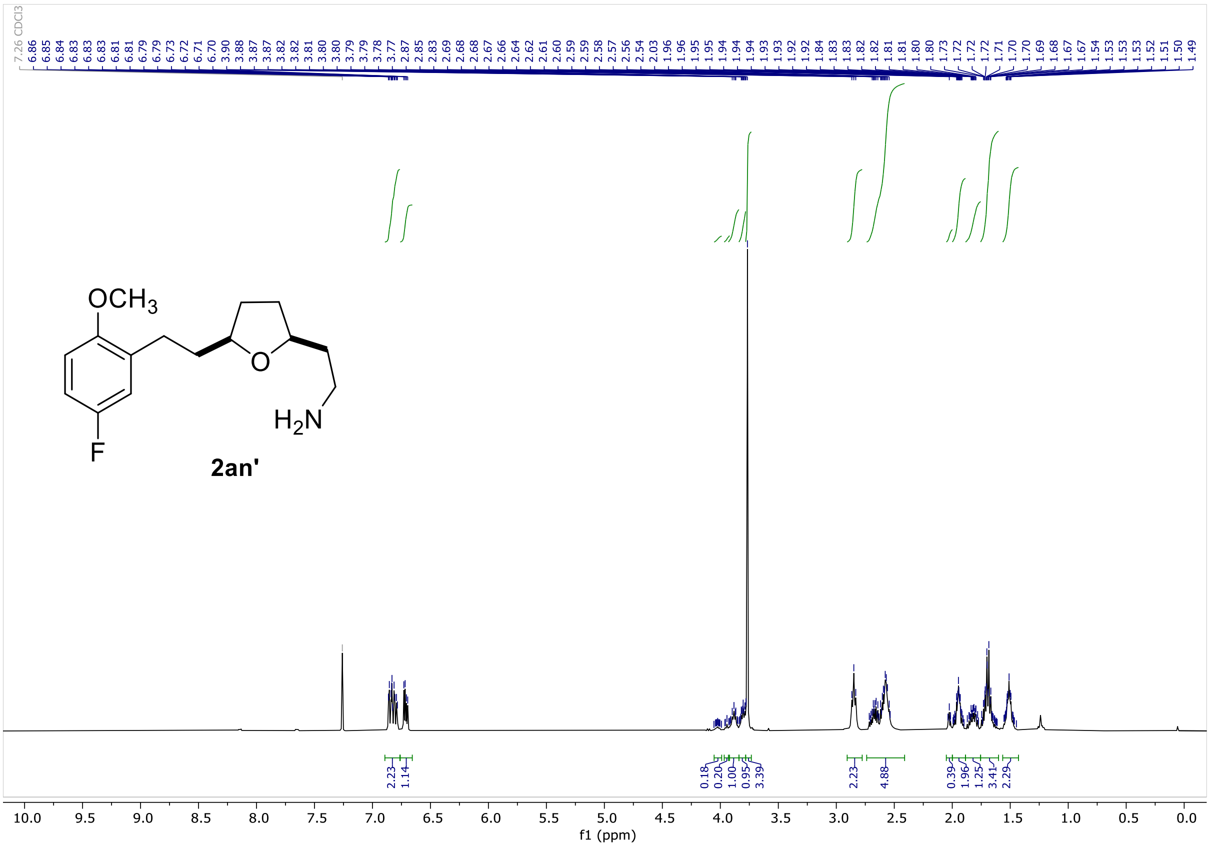

^{19}F NMR (377 MHz, $CDCl_3$) of **2an´**

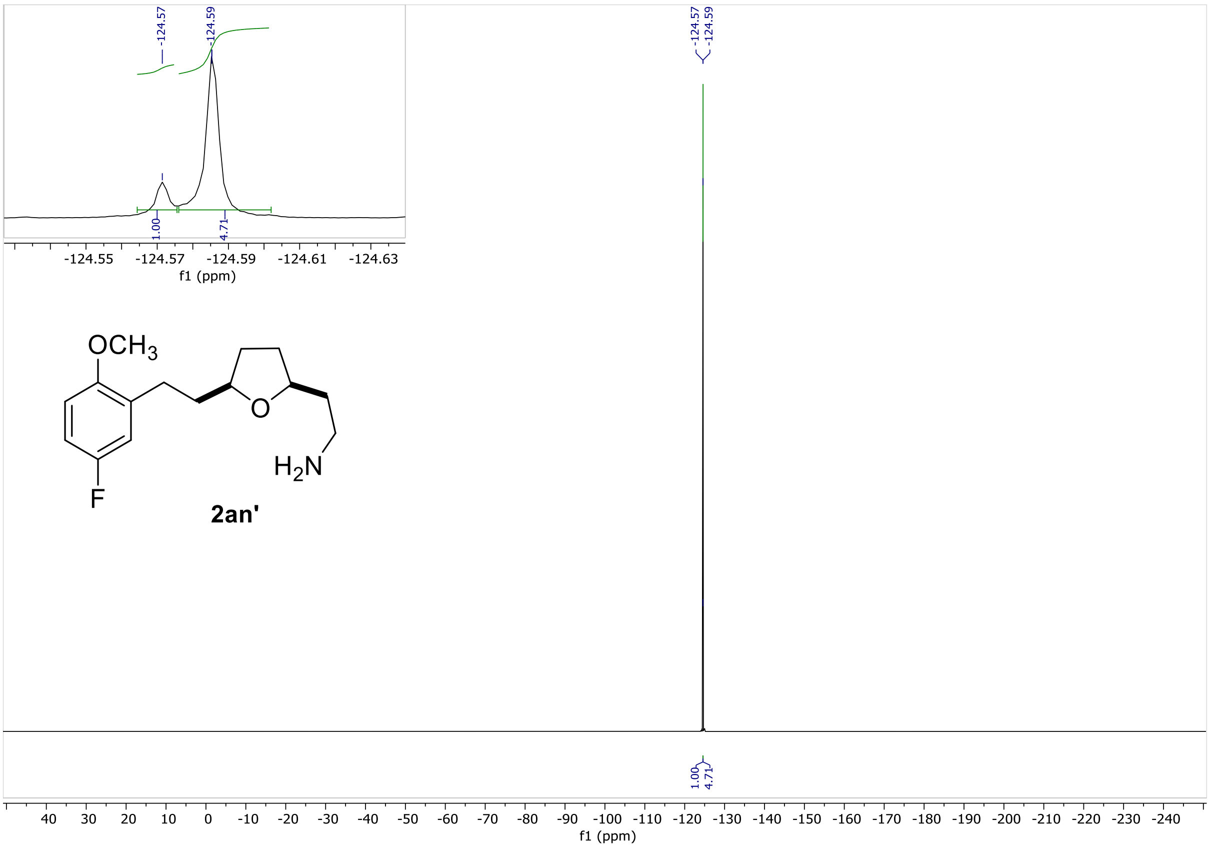

10. References

[1] Á. Gyömöre, M. Bakos, T. Földes, I. Pápai, A. Domján, T. Soós, "Moisture-Tolerant Frustrated Lewis Pair Catalyst for Hydrogenation of Aldehydes and Ketones" *ACS Catal.* **2015**, *5*, 5366.

[2] M. Bakos, Z. Dobi, D. Fegyverneki, Á. Gyömöre, I. Fernández, T. Soós, "Janus Face of the Steric Effect in a Lewis Acid Catalyst with Size-Exclusion Design: Steric Repulsion and Steric Attraction in the Catalytic Exo-Selective Diels–Alder Reaction" *ACS Sustain. Chem. Eng.* **2018**, *6*, 10869.

[3] S. Park, "Catalytic Reduction of Cyclic Ethers with Hydrosilanes" *Chem. Asian J.* **2019**, *14*, 2048.

[4] L. R. Jefferies, S. P. Cook, "Iron-Catalyzed Arene Alkylation Reactions with Unactivated Secondary Alcohols" *Org. Lett.* **2014**, *16*, 2026.

[5] S. Xue, L.-Z. Li, Y.-K. Liu, Q.-X. Guo, "Zinc-Mediated Chain Extension Reaction of 1,3-Diketones to 1,4-Diketones and Diastereoselective Synthesis of *Trans*-1,2-Disubstituted Cyclopropanols" *J. Org. Chem.* **2006**, *71*, 215.

[6] U. Karotsina, M. Pilicheva, A. Hurski, D. Astashko, "Ligand Enabled Manganese-Catalyzed Oxidation of 1,2-Disubstituted Cyclobutanols" *ChemistrySelect* **2024**, *9*, e202402709.

[7] T. Rigotti, T. Bach, "Bicyclo[2.1.1]Hexanes by Visible Light-Driven Intramolecular Crossed [2 + 2] Photocycloadditions" *Org. Lett.* **2022**, *24*, 8821.

[8] Y. Li, J.-Q. Shang, X.-X. Wang, W.-J. Xia, T. Yang, Y. Xin, Y.-M. Li, "Copper-Catalyzed Decarboxylative Oxyalkylation of Alkynyl Carboxylic Acids: Synthesis of γ-Diketones and γ-Ketonitriles" *Org. Lett.* **2019**, *24*, 2227.

[9] J. Zhou, M. Jia, M. Song, Z. Huang, A. Steiner, Q. An, J. Ma, Z. Guo, Q. Zhang, H. Sun, C. Robertson, J. Bacsa, J. Xiao, C. Li, "Chemoselective Oxyfunctionalization of Functionalized Benzylic Compounds with a Manganese Catalyst" *Angew. Chem. Int. Ed.* **2022**, *61*, e202205983.

[10] F.-P. Wu, X.-F. Wu, "Catalyst-Controlled Selective Borocarbonylation of Benzylidenecyclopropanes: Regiodivergent Synthesis of γ-Vinylboryl Ketones and β-Cyclopropylboryl Ketones" *Chem. Sci.* **2022**, *13*, 4321.

[11] K. Maeda, R. Matsubara, M. Hayashi, "Synthesis of Substituted Anilines from Cyclohexanones Using Pd/C–Ethylene System and Its Application to Indole Synthesis" *Org. Lett.* **2021**, *23*, 1530.

[12] E. Arceo, A. Bahamonde, G. Bergonzini, P. Melchiorre, "Enantioselective direct α-alkylation of cyclic ketones by means of photo-organocatalysis" *Chem. Sci.* **2014**, *5*, 2438.

[13] J. W. Rackl, A. F. Müller, C. Bärtschi, H. Wennemers, "ETHos – A Swiss-Made Open-Source Modular Photoreactor for Laboratory-Scale Photochemical Reactions" *Helv. Chim. Acta* **2024**, *107*, e202400154.

[14] L. Kersten, G. Hilt, “Regioselective Cobalt-Catalysed Hydrovinylation for the Synthesis of Non-Conjugated Enones and 1,4-Diketones” *Adv. Synth. Catal.* **2012**, *354*, 863.

[15] G. González Miera, A. Bermejo López, E. Martínez-Castro, P. Norrby, B. Martín-Matute, “Nonclassical Mechanism in the Cyclodehydration of Diols Catalyzed by a Bifunctional Iridium Complex” *Chem. Eur. J.* **2019**, *25*, 2631.

[16] Y. Liu, S. Liu, D. Li, N. Zhang, L. Peng, J. Ao, C. E. Song, Y. Lan, H. Yan, “Kinetic Resolution of Allylic Alcohol with Chiral BINOL-Based Alkoxides: A Combination of Experimental and Theoretical Studies” *J. Am. Chem. Soc.* **2019**, *141*, 1150.

[17] J. O. Smith, B. K. Mandal, “A Convenient Synthesis of 8-substituted Indolizines as Precursors to 5-substituted Cycl{3.2.2}azine Derivatives” *J. Heterocycl. Chem.* **1997**, *34*, 1441.

[18] N. M. Nevar, A. V. Kel'in, O. G. Kulinkovich, “One Step Preparation of 1,4-Diketones from Methyl Ketones and α-Bromomethyl Ketones in the Presence of $ZnCl_2$·t-BuOH·Et_2NR as a Condensation Agent” *Synthesis* **2000**, *9*, 1259.

[19] L. Fang, S. Jia, S. Fana, J. Zhu, “Palladium-catalyzed coupling of amides and cyclopropanols for the synthesis of γ-diketones” *Chem. Commun.* **2023**, *59*, 10392.

[20] M. Reinhold, J. Steinebach, C. Golz, J. C. L. Walker, “Synthesis of Polysubstituted Bicyclo[2.1.1]Hexanes Enabling Access to New Chemical Space” *Chem. Sci.* **2023**, *14*, 9885.

[21] M. Zhao, Y. Liu, X. Chen, M. Peng, Y. Wang, X. Liu, H. Jiang, R. Tan, J. Li, “Photocatalyst-Free Formate-Mediated C–O Cleavage by the EDA Complex and SCS Strategy for the Synthesis of Diaryl 1,4-Diketone in Air” *Org. Biomol. Chem.* **2025**, *23*, 2079.

[22] F. Allais, P.-H. Ducrot, “Stereoselective Total Synthesis of (+)-Dodoneine” *Synthesis* **2010**, *2010*, 1649.

[23] X. Huang, J. Li, H. He, K. Yan, R. Lai, Y. Luo, M. Guan, Y. Wu, “Ruthenium-Catalyzed Alkylation of Cyclopropanols with Sulfoxonium Ylides via C–C Bond Cleavage: Formation of Diverse 1,5-Diketones” *Synthesis* **2022**, *54*, 779.

[24] R. M. P. Dias, A. C. B. Burtoloso, “Catalyst-Free Insertion of Sulfoxonium Ylides into Aryl Thiols. A Direct Preparation of β-Keto Thioethers.” *Org. Lett.* **2016**, *18*, 3034.

[25] L. Zhang, J. Zhang, J. Ma, D.-J. Cheng, B. Tan, “Highly Atroposelective Synthesis of Arylpyrroles by Catalytic Asymmetric Paal–Knorr Reaction” *J. Am. Chem. Soc.* **2017**, *139*, 1714.

[26] M. Ceylan, M. Gürdere, Y. Budak, C. Kazaz, H. Seçen, “One-Step Preparation of Symmetrical 1,4-Diketones from α-Halo Ketones in the Presence of Zn-I_2 as a Condensation Agent” *Synthesis* **2004**, *2004*, 1750.

[27] P. R. Jagtap, I. Císařová, U. Jahn, “Bioinspired Total Synthesis of Tetrahydrofuran Lignans by Tandem Nucleophilic Addition/Redox Isomerization/Oxidative Coupling and Cycloetherification Reactions as Key Steps” *Org. Biomol. Chem.* **2018**, *16*, 750.

[28] M. Araki, S. Sakata, H. Takei, T. Mukaiyama, "T. REACTION OF MIXED CARBOXYLIC ANHYDRIDES WITH GRIGNARD REAGENTS. A CONVENIENT METHOD FOR THE PREPARATION OF 1,4- OR 1,6-DIKETONES" *Chem. Lett.* **1974**, *3*, 687.

[29] G. G. Bagkavou, C. I. Stathakis, "Iron(III) Catalyzed Aerobic C_α–C_β Cleavage of Allylic Alcohols" *Eur. J. Org. Chem.* **2024**, *27*, e202400556.

[30] J. Wysocki, N. Ortega, F. Glorius, "Asymmetric Hydrogenation of Disubstituted Furans" *Angew. Chem. Int. Ed.* **2014**, *53*, 8751.

[31] S. J. Gharpure, D. S. Vishwakarma, S. K. Nanda, "Lewis Acid Mediated "*Endo-Dig*" Hydroalkoxylation–Reduction on Internal Alkynols for the Stereoselective Synthesis of Cyclic Ethers and 1,4-Oxazepanes" *Org. Lett.* **2017**, *19*, 6534.

[32] X. Jiang, E. K. London, D. J. Morris, G. J. Clarkson, M. Wills, "Gold-Catalysed Cyclic Ether Formation from Diols" *Tetrahedron* **2010**, *66*, 9828.

[33] J. P. Schmidt, B. Breit, "Rhodium-Catalyzed Cyclization of Terminal and Internal Allenols: An Atom Economic and Highly Stereoselective Access Towards Tetrahydropyrans" *Angew. Chem. Int. Ed.* **2020**, *59*, 23485.

[34] S. Tang, K. Liu, Y. Long, X. Qi, Y. Lan, A. Lei, "Tuning Radical Reactivity Using Iodine in Oxidative C(Sp^3)–H/C(Sp)–H Cross-Coupling: An Easy Way toward the Synthesis of Furans and Indolizines" *Chem. Commun.* **2015**, *51*, 8769.

[35] P. Clawson, P. M. Lunn, D. A. Whiting, "Synthetic Studies on O-Heterocycles via Cycloadditions. Part 1. Photochemical (Electron Transfer Sensitised) C-C Cleavage of Diaryloxiranes" *J. Chem. Soc. Perkin 1* **1990**, No. 1, 153.

[36] K. C. Nicolaou, C. K. Hwang, M. E. Duggan, D. A. Nugiel, Y. Abe, K. B. Reddy, S. A. DeFrees, D. R. Reddy, R. A. Awartani, S. R. Conley, F. P. J. T. Ruties, E. A. Theodorakis, "Total Synthesis of Brevetoxin B. 1. First Generation Strategies and New Approaches to Oxepane Systems" *J. Am. Chem. Soc.* **1995**, *117*.

[37] D. Rasina, M. Otikovs, J. Leitans, R. Recacha, O. V. Borysov, I. Kanepe-Lapsa, I. Domraceva, T. Pantelejevs, K. Tars, M. J. Blackman, K. Jaudzems, A. Jirgensons, "Fragment-Based Discovery of 2-Aminoquinazolin-4(3*H*)-Ones As Novel Class Nonpeptidomimetic Inhibitors of the Plasmepsins I, II, and IV" *J. Med. Chem.* **2016**, *59*, 374.

[38] T. Voelker, H. Xia, K. Fandrick, R. Johnson, A. Janowsky, J. R. Cashman, "2,5-Disubstituted Tetrahydrofurans as Selective Serotonin Re-Uptake Inhibitors" *Bioorg. Med. Chem.* **2009**, *17*, 2047.

[39] S. Sau, K. M. Das, B. Mondal, A. Thakur, "Cobalt(II)-Catalyzed Synthesis of γ-Diketones from Aryl Alkenes and Its Utilization in the Synthesis of Various Heterocyclic Compounds" *J. Org. Chem.* **2024**, *89*, 7095.

[40] B. Brutiu, G. Iannelli, M. Riomet, D. Kaiser, N. Maulide, "Stereodivergent 1,3-Difunctionalization of Alkenes by Charge Relocation" *Nature* **2024**, *626*, 92.

[41] H. Suzuki, S. Yoshioka, A. Igesaka, H. Nishioka, Y. Takeuchi, "Palladium-Catalyzed Hydrogenation with Use of Ionic Liquid Bis(2-Hydroxyethyl)Ammonium Formate [BHEA][HCO2] as a Solvent and Hydrogen Source" *Tetrahedron* **2013**, *69*, 6399.

[42] C. Bannwarth, S. Ehlert, S. Grimme, "GFN2-XTB—An Accurate and Broadly Parametrized Self-Consistent Tight-Binding Quantum Chemical Method with Multipole Electrostatics and Density-Dependent Dispersion Contributions" *J. Chem. Theory. Comput.* **2019**, *15*, 1652.

[43] C. Bannwarth, E. Caldeweyher, S. Ehlert, A. Hansen, P. Pracht, J. Seibert, S. Spicher, S. Grimme, "Extended tight-binding quantum chemistry methods" *WIREs Comput. Mol. Sci.* **2021**, *11*, e1493.

[44] P. Pracht, F. Bohle, S. Grimme, "Automated Exploration of the Low-Energy Chemical Space with Fast Quantum Chemical Methods" *Phys. Chem. Chem. Phys.* **2020**, *22*, 7169.

[45] S. Grimme, "Exploration of Chemical Compound, Conformer, and Reaction Space with Meta-Dynamics Simulations Based on Tight-Binding Quantum Chemical Calculations" *J. Chem. Theory Comput.* **2019**, *15*, 2847.

[46] S. Grimme, F. Bohle, A. Hansen, P. Pracht, S. Spicher, M. Stahn, "Efficient Quantum Chemical Calculation of Structure Ensembles and Free Energies for Nonrigid Molecules" *J. Phys. Chem. A* **2021**, *125*, 4039.

[47] S. Ehlert, M. Stahn, S. Spicher, S. Grimme, "Robust and Efficient Implicit Solvation Model for Fast Semiempirical Methods" *J. Chem. Theory Comput.* **2021**, *17*, 4250.

[48] F. Neese, F. Wennmohs, U. Becker, C. Riplinger, "The ORCA Quantum Chemistry Program Package" *J. Chem. Phys.* **2020**, *152*. https://doi.org/10.1063/5.0004608.

[49] F. Neese, "Software Update: The ORCA Program System—Version 5.0" *WIREs Comput. Mol. Sci.* **2022**, *12*. https://doi.org/10.1002/wcms.1606.

[50] J.-D. Chai, M. Head-Gordon, "Long-Range Corrected Hybrid Density Functionals with Damped Atom–Atom Dispersion Corrections" *Phys. Chem. Chem. Phys.* **2008**, *10*, 6615.

[51] Y.-S. Lin, G.-D. Li, S.-P. Mao, J.-D. Chai, "Long-Range Corrected Hybrid Density Functionals with Improved Dispersion Corrections" *J. Chem. Theory Comput.* **2013**, *9*, 263.

[52] S. Grimme, J. Antony, S. Ehrlich, H. Krieg, "A Consistent and Accurate *Ab Initio* Parametrization of Density Functional Dispersion Correction (DFT-D) for the 94 Elements H-Pu" *J. Chem. Phys.* **2010**, *132*, 154104.

[53] S. Grimme, S. Ehrlich, L. Goerigk, "Effect of the Damping Function in Dispersion Corrected Density Functional Theory" *J. Comput. Chem.* **2011**, *32*, 1456.

[54] F. Weigend, "Accurate Coulomb-Fitting Basis Sets for H to Rn" *Phys. Chem. Chem. Phys.* **2006**, *8*, 1057.

[55] F. Weigend, R. Ahlrichs, “Balanced Basis Sets of Split Valence, Triple Zeta Valence and Quadruple Zeta Valence Quality for H to Rn: Design and Assessment of Accuracy” *Phys. Chem. Chem. Phys.* **2005**, *7*, 3297.

[56] V. Barone, M. Cossi, “Quantum Calculation of Molecular Energies and Energy Gradients in Solution by a Conductor Solvent Model” *J. Phys. Chem. A* **1998**, *102*, 1995.

[57] E. R. Johnson, S. Keinan, P. Mori-Sánchez, J. Contreras-García, A. J. Cohen, W. Yang, “Revealing Noncovalent Interactions” *J. Am. Chem. Soc.* **2010**, *132*, 6498.

[58] W. Humphrey, A. Dalke, K. Schulten, “VMD: Visual Molecular Dynamics” *J. Mol. Graph.* **1996**, *14*, 33.

[59] *Chemcraft - graphical software for visualization of quantum chemistry computations. Version 1.8, build 682.* https://www.chemcraftprog.com.

[60] C. Stoian, M. Olaru, T. A. Cucuiet, K. T. Kegyes, A. Sava, A. Y. Timoshkin, C. I. Raţ, J. Beckmann, “Bulky Polyfluorinated Terphenyldiphenylboranes: Water Tolerant Lewis Acids” *Chem. Eur. J.* **2021**, *27*, 4327.

[61] C. Janiak, “A critical account on π–π stacking in metal complexes with aromatic nitrogen-containing ligands” *J. Chem. Soc., Dalton Trans.* **2000***,* 3885.

[62] Q. Peng, F. Duarte, R. S. Paton, “Computing Organic Stereoselectivity-from Concepts to Quantitative Calculations and Predictions” *Chem. Soc. Rev.* **2016**, *45*, 6093.

[63] O. V. Dolomanov, L. J. Bourhis, R. J. Gildea, J. A. K. Howard, H. Puschmann, “*OLEX2*: A Complete Structure Solution, Refinement and Analysis Program” *J. Appl. Crystallogr.* **2009**, *42*, 339.

[64] G. M. Sheldrick, “*SHELXT* – Integrated Space-Group and Crystal-Structure Determination” *Acta Cryst.* **2015**, A*71*, 3.

[65] G. M. Sheldrick, “Crystal Structure Refinement with *SHELXL*” *Acta Cryst.* **2015**, C*71*, 3.
